# Palladium-catalyzed α-arylation for the addition of small rings to aromatic compounds

**DOI:** 10.1038/s41467-019-12090-z

**Published:** 2019-09-09

**Authors:** Zhi-Tao He, John F. Hartwig

**Affiliations:** Department of Chemistry, University of California, Berkeley, CA 94720 United States

**Keywords:** Catalytic mechanisms, Homogeneous catalysis, Synthetic chemistry methodology

## Abstract

Small, strained rings have rigid, defined conformations and unique electronic properties. For these reasons, many groups seek to use these subunits to form biologically active molecules. We report a generally applicable approach to attach small rings to a wide range of aromatic compounds by palladium-catalyzed α-arylation of cyclopropyl, cyclobutyl and azetidinyl esters. The direct α-arylation of cyclopropyl esters and cyclobutyl esters is achieved in high yield by ensuring that the rate of coupling exceeds the rate of Claisen condensation. The α-arylation of azetidines is achieved without ring opening of the strained saturated heterocycle by conducting the reactions with an azetidine derivative bearing a benzyl protecting group on nitrogen. Mechanistic studies show that the α-arylation of small rings is challenging because of the weak acidity of α C-H bond (cyclopropanes), strong sensitivity of the strained esters to Claisen condensation (cyclobutatanes), or facile decomposition of the enolates (azetidinyl esters).

## Abstract (web summary)

Methods to prepare small, strained rings are sought after due to the importance of such scaffolds in medicinal chemistry. Here, the authors report a palladium-catalyzed α-arylation of cyclopropyl, cyclobutyl and azetidinyl esters.

## Introduction

**Fig. 1:**
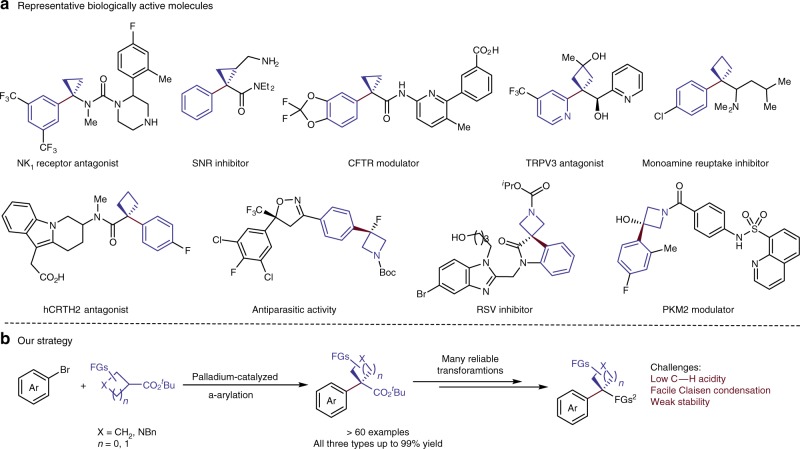
Representative biologically active molecules containing small rings and our strategy for their synthesis. **a** Selected biologically active molecules containing aryl-substituted small rings. **b** Our strategy for the introduction of small rings to aromatic compounds. FG: functional group

The unique electronic, steric, and conformational properties of small rings have led to an increased interest in methods to incorporate such structures into pharmaceutical and agrochemical candidates^1–5^. For example, the rigid conformation and strong C–H bonds of cyclopropanes have been stated to help achieve a range of properties, including enhanced binding and potency, increased metabolic stability, increased brain permeability, decreased plasma clearance, and reduced off-target effects^2^. The combination of small rings and aromatic fragments is particularly attractive because aryl and heteroaryl units are prevalent in compounds valuable for medicinal chemistry and agrochemistry (Fig. 1a)^6–15^.

Palladium-catalyzed α-arylation reactions have been studied extensively, since the initial work published by Buchwald, Miura, and our group^16–23^. However, documented efforts to develop direct methods to couple aryl halides with the esters of small rings (Fig. 1b), whether small carbocyclic or heterocyclic esters, have given no product or have been reported in two examples in patents to give few turnovers and modest yields that we have not been able to reproduce (vide infra)^24–27^. Reactions of Reformatsky reagents generated from alpha-bromo esters requiring multiple steps for preparation have been published^28–30^, and the reactions of small-ring nitriles as surrogates of the esters have been reported^24,25,31^, but the direct reactions of three-membered or four-membered ring carbocyclic or heterocyclic esters with aryl halides in the presence of base and a catalyst have not been published^26,27^. Because esters can be converted easily into a variety of functional groups, the coupling of esters with aryl halides would be the most valuable version of the coupling of a small-ring enolate.

The same properties of small rings that make them valuable substructures in biologically active molecules make the reactions of such structures difficult to develop. The acidities of C–H bonds and electrophilicities of attached carbonyl groups are distinct from those of unstrained analogs, and the propensity to undergo ring opening under conditions of synthetic chemistry is well known. In particular, the lower acidity of cyclopropyl esters and the greater electrophilicity of cyclopropyl and cyclobutyl ester carbonyl groups, relative to those of unstrained esters, cause Claisen condensation to compete with coupling. The propensity of azetidines protected with typical carbamate substituents makes the enolates of such azetidines unstable. To overcome these challenges, ester derivatives that form the enolates in high yield, conditions to generate the enolates without condensation, catalysts with high activity to outcompete competing processes, and azetidine derivatives that resist ring opening are needed. Further, an assessment of the rates of deprotonation and rates of side reactions are needed to determine the properties of these ring systems that limit catalytic couplings.

Here, we report a widely applicable, one-step approach with readily accessible reagents for the attachment of small rings to aromatic compounds by palladium-catalyzed α-arylation. The reactions of cyclopropyl esters occur in high yield with a catalyst precursor not used previously for the α-arylation of esters. Broadly applicable reactions of cyclobutyl and azetidinyl esters occur after identification of the origins of the low yields of the one prior example of each reaction and redesign of the reagents and conditions. The arylations of all three types of small rings now occur with broad scope in yields up to 99%. Finally, an unusual combination of debenzylation and alkylation of the azetidine nitrogen occurs, and broadly applicable derivatizations of the nitrogen in the product from coupling of azetidines can be conducted. The synthesis of three biologically relevant molecules containing small rings demonstrates the value of these valuable α-arylations.

## Results

### Development of the α-arylation of cyclopropyl esters

The coupling of cyclopropyl esters has not been reported previously, and recent literature documented the inability to identify conditions for this process, even by modern high-throughput methods^24,25^. Thus, less efficient, alternative routes have been used to produce α-aryl cyclopropyl esters, such as reactions of unstable Reformatsky reagents requiring three steps for preparation of even the simplest zinc enolates^28–30^, or reactions of cyclopropyl nitriles as surrogates in moderate yield^24,25^. Due to the lack of direct α-arylations of cyclopropyl esters and the highest value of ester units for subsequent transformations, we sought to discover a method to accomplish this transformation.

Consistent with the prior literature^24,25^, our initial efforts to achieve the α-arylation of a cyclopropyl methyl ester under the conditions reported for common esters gave no coupled product^32,33^. Only the side product of Claisen condensation was observed. We considered that a *t*-butyl ester should undergo condensation less rapidly and allow formation of the enolate in higher yield. However, most α-arylation reactions of α,α-disubstituted esters have been conducted with esters that are less hindered than *t*-butyl esters because the steric hindrance of the *t*-butyl ester prevents reaction at the hindered α-position^34–36^. It was unclear if the small ring esters would behave more like α,α-disubstituted esters or less hindered esters.

The reaction of 4-bromo-1-fluoro-2-methoxybenzene (**2a**) with cyclopropyl *t*-butyl ester **1a** was used to evaluate catalysts and conditions that would lead to the α-arylation of cyclopropyl esters, and the results of these experiments are shown in Supplementary Table 1. This reaction gave a measurable, but low, 38% yield of coupled product **3a** under the conditions reported for coupling of acyclic esters (Supplementary Table 1, entry 1). Reactions with varied ligands, bases, and temperatures gave less than 63% yield of the coupled product (Supplementary Table 1, entry 2–12).

In most cases, substantial quantities of the bromoarene remained unreacted, even though the same bromoarene is known to undergo rapid α-arylation of enolates, including acyclic esters^32,33^. We presumed the ester enolate decomposed or underwent Claisen condensation, leaving unreacted bromoarene. In this case, a higher proportion of active catalyst was needed for the rate of coupling to be higher than the rate of decomposition of the enolate. Thus, we tested alternative palladium sources and found that reactions catalyzed by the Pd precursors (**Pd-4**, **Pd-5**) recently reported by Hazari occurred to full conversion of the bromoarene (Supplementary Table 1, entry 16–17)^37,38^. Because a cationic metal center having a lower coordination number would be more electrophilic and susceptible to nucleophilic attack at the sacrificial ligand^39^, AgBF_4_ was added prior to the enolate to generate a cationic Pd complex in situ. With this further change to the method for generating the active catalyst, the α-aryl ester was obtained in 99% isolated yield (Supplementary Table 1, entry 18).

### Scope of the α-arylation of cyclopropyl esters

**Fig. 2:**
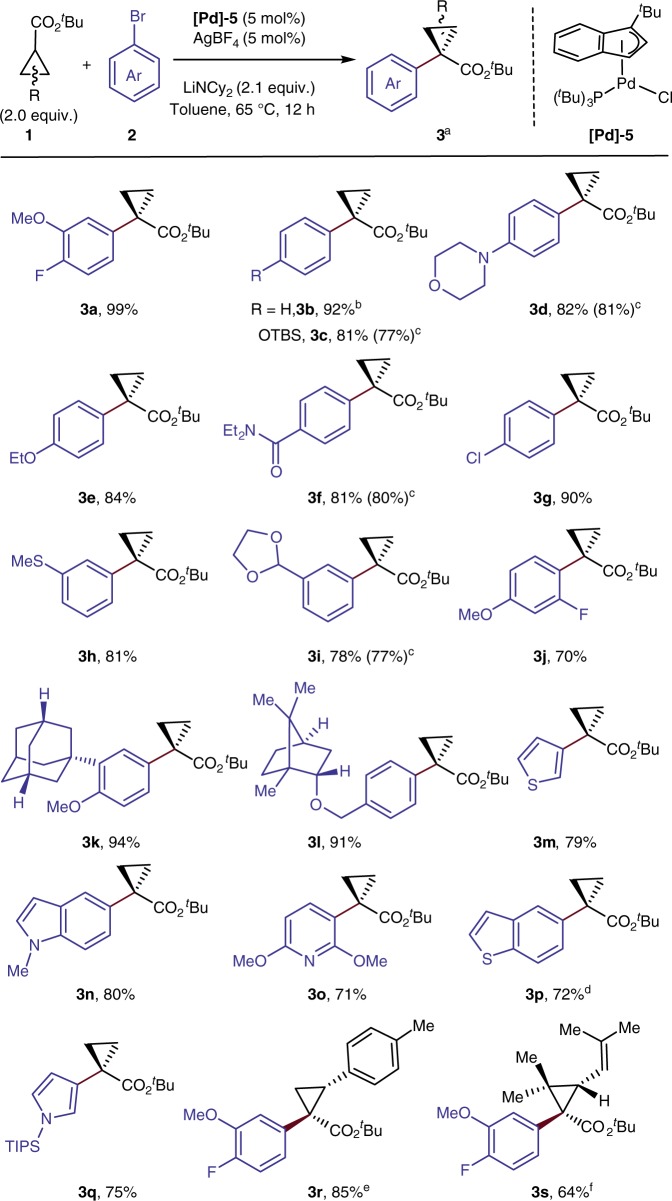
Scope of α-arylation of cyclopropyl *t*-butyl esters. ^a^Isolated yields, dr was determined by crude ^19^F NMR. ^b^With PhCl or PhOTf used instead of PhBr, no product was observed; with PhI used instead, 64% yield. ^c^Yield in the parentheses in 10 min. ^d^7 mol% [Pd] cat and AgBF_4_ were used. ^e^50 ^°^C, dr 12:1, major isomer is shown. ^f^Major isomer is shown; dr = 2:1. TIPS: triisopropylsilyl

The scope of the α-arylation of *t*-butyl cyclopropyl esters under the developed conditions is illustrated by the examples in Fig. 2. Aryl bromides containing a variety of functional groups at varying positions reacted with 2.0 equiv. of cyclopropyl *t*-butyl ester and 2.1 equiv. of lithium dicyclohexylamide (LiNCy_2_) as base in 70–99% yield when catalyzed by 5% of **Pd-5** in toluene at 65 ^°^C for 12 h (**3a**–**3j**). Both strongly electron-donating (morpholine in **3d**) and electron-withdrawing substituents (amide and acetal in **3f** and **3i**, respectively) on the aryl bromides were tolerated, along with potentially coordinating thioethers (**3h**) and potentially reactive silyl ethers (**3c**). The coupling also occurred with aryl bromides containing polycyclic substituents, such as **2k** and **2l**, providing **3k** and **3l** in 94% and 91% yield, respectively. Coupling of heteroaryl electrophiles, such as bromothiophenes, -indoles, -pyridines, -benzothiophenes, and -pyrroles occurred in >70% yield (**3m**–**3q**). Reaction of an aryl-substituted cyclopropyl ester **1r** gave **3r** in 85% yield with high diastereoselectivity. Reaction of a highly substituted cyclopropyl ester, ethyl chrysanthemumate **1s**, even occurred smoothly to give 64% isolated yield of the coupled product **3s**.

So far, the reactions of phenyl chloride and phenyl triflate as a representative member of these types of aryl electrophiles have not given coupled product, even though that of phenyl bromide gave the arylated product **3b** in 92% yield. The reaction of phenyl iodide did occur to give the coupled product in 64% yield. The 12-h reaction time used for convenience when assessing reaction scope was not necessary. The arylation of cyclopropyl esters with both electron-rich and electron-poor aryl bromides (**3c**, **3d**, **3f**, **3i**) afforded the coupled products after just 10 min in yields that were comparable to reactions conducted for the longer times.

### Development of the α-arylation of cyclobutyl esters

Just one example of the direct α-arylation of cyclobutyl methyl ester was disclosed in a patent and stated to occur in 53% yield^26^. Because of the modest yield and narrow scope of this reaction, the products have been prepared with alternative reactants, such as silyl ketene acetals or nitriles^24,25,31,40^. However, the versatility of esters for further derivatization and the simplicity of using an ester for the coupling reaction make it important to identify conditions and catalyst for the direct α-arylation of cyclobutyl esters.

To test the veracity and potential scope of the patented procedure^26^, we studied reactions of arylbromide **5** with cyclobutyl methyl ester **4a** (Supplementary Table 2). However, <30% yield of **6a** was observed after a series of initial studies. The major products in all cases resulted from Claisen condensation of the cyclobutyl methyl ester **4a** and hydrodehalogenation of the aryl bromide **5**.

To suppress the Claisen condensation and to avoid formation of a reducing alcohol, we tested reactions with a bulkier ester. Indeed, the reaction of cyclobutyl *t*-butyl ester **4b** (Supplementary Figure 14) gave a much higher 76% yield of coupled product **6b** without any detectable product from Claisen condensation. Yet, Pd-catalyzed hydrodehalogenation of aryl bromide **4b**, possibly from the α-proton of ester **5** or trace water, was still observed. Instead, reaction conducted with just 1% of indenyl complex **[Pd]−5** (Supplementary Fig. 14) gave product **6c** in 99% yield without any observed side reactions. Thus, the coupling of a *t*-butyl cyclobutyl ester with an aryl halide occurs in high yield with 1% **[Pd]−5** as catalyst, and 2% AgOTf as additive in toluene at 50 ^°^C.

### Scope of α-arylation of cyclobutyl *t*-butyl esters

**Fig. 3:**
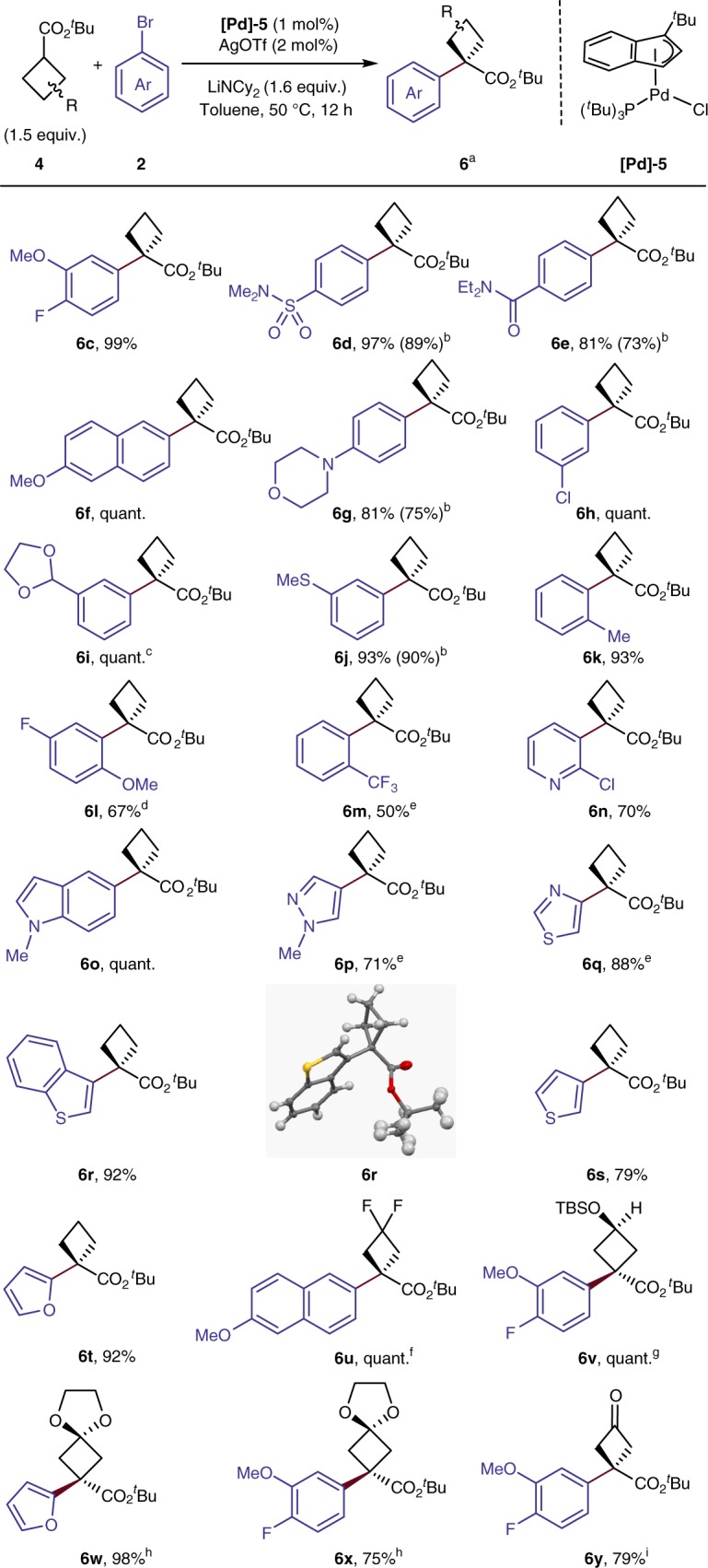
Scope of α-arylation of cyclobutyl *t*-butyl esters. ^a^Isolated yields. ^b^Yield in the parentheses in 10 min. ^c^2 mol% [Pd] cat and AgOTf were used. ^d^2 mol% [Pd] cat was used. ^e^5 mol% [Pd] cat was used. ^f^5 mol% [Pd] cat was used without AgOTf. ^g^5 mol% [Pd] cat and AgBF_4_ were used, dr 4:1, major isomer is shown. ^h^5 mol% [Pd] cat and AgBF_4_, ester (2 equiv.), LiNCy_2_ (2.1 equiv.) at 65 ^°^C were used. ^i^From **6u** through hydrolysis. TBS: *tert*-butyldimethylsilyl

Experiments on the scope of the α-arylation of cyclobutyl *t*-butyl esters with a series of aryl bromides is summarized in Fig. 3. Both electron-rich and electron-poor aryl bromides underwent coupling in 81–99% yield with 1 mol% **[Pd]−5**. Functional groups, including aryl chlorides, acetals, sulfonamides, amides, morpholino groups, and a thioether at varying positions were all tolerated (**6c**–**6j**). When 1-bromo-2-methylbenzene was used as substrate, product **6k** formed in 93% yield. Aryl halides containing more hindered *ortho* substituents reacted in lower yet still moderate yields with a higher catalyst loading (**6l**–**6m**). The coupling of a range of heteroaryl bromides also occurred. The coupling of brominated pyridine, indole, pyrrole, thiazole, thiophene, furan and benzothiazole with *t*-butyl ester **4b** occurred in 70% to quantitative yield (**6n**–**6t**). The structure of **6r** was confirmed by X-ray crystallographic analysis.

The α-arylation of cyclobutyl esters extends beyond reactions of the parent cyclobutyl carboxylic esters. The results in Fig. 3 show that the coupling of cyclobutyl esters containing a variety of substitution patterns on the cyclobutane ring occurred in high yield. The increased structural complexity of the corresponding products could allow further transformations or derivatization. For example, the cyclobutyl ester containing an acetal group coupled efficiently with both aryl and heteroaryl bromides (75% yield for **6x**, 98% yield for **6w**). These structures serve as sources of the cyclobutanones after hydrolysis. For example, the direct hydrolysis of **6x** gave the cyclobutanone **6y** in 79% yield. A TBS-protected hydroxyl group was well tolerated, furnishing arylated product **6v** in quantitative yield with 4:1 diastereoselectivity. A substrate containing a *gem*-difluoromethylene group also coupled in quantitative yield (**6u**). Again, the yields of these arylation reactions with both electron-rich and electron-poor aryl bromides after 10 min were comparable to those obtained from standard 12-h reaction times (**6d**, **6e**, **6g**, **6j**).

### Development of the α-arylation of azetidine *t*-butyl esters

Small saturated heterocycles are known to undergo ring opening in the presence of nucleophiles^41,42^, and this reactivity could interfere with the α-arylations of azetidine carboxylates. Consistent with this assertion, the α-arylations of azetidine esters have been limited to a single reaction of a Cbz-protected azetidine *t*-butyl ester in a patent stated to occur in 69% yield with 16 mol% [Pd] loading^27^, and even this one example has not been reproducible after extensive experimentation.

Our attempts to couple the Cbz-protected azetidine with an aryl bromide showed that reactions of the two coupling partners in the patent disclosure under conditions identical to those described gave only 17% yield of the product with 16 mol% of catalyst, far less than the reported 69% (Supplementary Table 3). Only 7% of the azetidine ester remained after 1 h at room temperature, indicating that the ester is unstable to the reaction conditions. The reaction of aryl bromide **2a**, with other azetidine esters containing a Boc or trifluoroacetyl group on nitrogen consumed all of the starting azetidine, but formed the coupled product in low yield. These results indicate that azetidine esters protected by electron-withdrawing groups on nitrogen are unsuitable for coupling with aryl halides with useful scope and yields under the reported conditions because they decompose, even at room temperature, in the presence of a base sufficiently strong to generate the enolate.

To achieve couplings of azetidines in high yield, we proposed that azetidines containing a less electron-withdrawing benzyl group on nitrogen should be more stable but suitable for subsequent deprotection (Supplementary Table 4). Although the reaction of Bn-protected azetidine **7d** with lithium bis(trimethylsilyl)amide (LiHMDS) as base gave no product, 32% of **7d** did remain unreacted after 12 h at 50 ^°^C, indicating that this azetidine derivative is more stable under the conditions of the α-arylation than those containing a carbonyl group on nitrogen. The reaction with LiNCy_2_ in place of LiHMDS gave 43% yield of product **8o**, and further experimentation showed that the reaction with lithium 2,2,6,6-tetramethylpiperidide (LiTMP) as base gave 88% yield of the coupled product when 1.5 equiv. of azetidine **7d** were used. Reaction conditions that are similar to those used for the α-arylation of cycloproyl ester gave a yield that was comparable (82% yield) to that from the combination of Pd(dba)_2_ + ^*t*^Bu_3_P as catalyst (88% yield) for the generation of compound **8o**. The use of Pd(P^*t*^Bu_3_)_2_ as catalyst also gave a comparable 82% yield of product **8o**.

### Scope of α-arylation of azetidine *t*-butyl esters

**Fig. 4:**
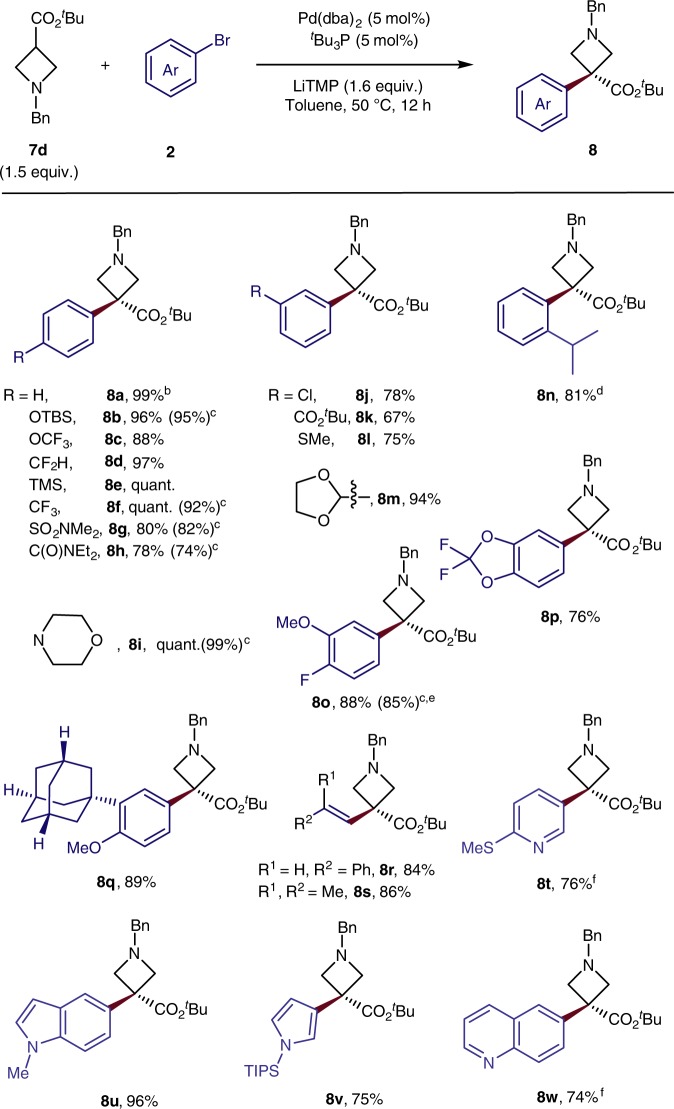
Scope of α-arylation of azetidinyl *t*-butyl esters. ^a^Isolated yields. ^b^For PhCl use instead, 40% yield; For PhI used instead, 70% yield; For PhOTf used instead, no product. ^c^Yield shown in the parentheses after 10 min. ^d^**7d** (2.0 equiv.), LiTMP (2.1 equiv.), [Pd] (10 mol%), ^*t*^Bu_3_P (10 mol%) were used instead. ^e^Half-gram scale test gave 85% yield. ^f^**7d** (2.0 equiv.), LiTMP (2.1 equiv.) were used instead

A wide range of aryl- and heteroaryl-bromides, as well as alkenyl bromides coupled with 3-*t*-butyl azetidine carboxylate under the developed conditions (Fig. 4). A series of functional groups at varying positions on the aryl ring were tolerated (**8a**-**8n**, 67–99% yield). For example, aryl bromides containing electron-donating groups, such as OTBS, SMe, or morpholino groups, gave 75–99% yield of coupled product (**8b**, **8l**, **8i**); those containing electron-withdrawing groups, such as CF_3_, sulfonamide, amide, Cl, and acetal, gave 78–99% yield of coupled product (**8f**–**8h**, **8j**, **8m**). Strongly base-sensitive groups were not tolerated, but an aryl bromide containing a *t*-butyl ester reacted in good yield (**8k**). Bulky substituents, like an *i*-propyl group, at the *ortho*-position of the aryl bromide were tolerated; *ortho*-isopropyl product **8n** formed in 81% isolated yield. Multi-substituted aryl bromides also reacted with the *t*-butyl azetidine carboxylate to give the coupled product in 76–89% yields (**8o**–**8q**). The reaction of a half-gram of bromide **2a** with 3-*t*-butyl azetidine carboxylate occurred in a yield that was comparable with that on a smaller scale (**8o**, 85% for half-gram scale vs 88% for 0.1 mmol scale).

Additional sp^2^ carbon electrophiles also reacted with the azetidine ester. For example, the alkenyl bromide *trans*-β-bromostyrene or 1-bromo-2-methylprop-1-ene reacted with 3-*tert*-butyl azetidine carboxylate to give the coupled product **8r** and **8** **s** in 84% and 86% yield, respectively. Likewise, a series of heteroaryl bromides coupled in high yield. For example, coupling of 3-*t*-butyl azetidine **7d** with a bromo-pyridine, -indole, -pyrrole and -quinoline all occurred in 74–96% yield (**8t**-**8w**).

In contrast to the α-arylation of small carbocycles, the α-arylation of *t-*butyl azetidine carboxylate occurred with phenyl chloride, affording the arylated product **8a** in a moderate 40% yield under the standard reaction conditions without further optimization. For comparison, the reaction of phenyl bromide and iodide occurred in 99% and 70% yield respectively. Similar to the α-arylation of small carbocycles, the α-arylation of *t-*butyl azetidine carboxylate with phenyl triflate did not occur. The reactions of *t-*butyl azetidine carboxylate with both electron-rich and electron-poor aryl bromides **8b**, **8f**-**8i**, and **8o** occurred to completion in 10 min, giving yields of coupled product that were comparable to those obtained after 12 h.

### Debenzylation-alkylation of the α-aryl azetidine product

The value of the *N*-benzyl azetidine products rests upon removal of the benzyl group. Thus, procedures were developed to remove or replace this group, and these studies revealed an unusual alkylation of the azetidine under typical conditions for hydrogenolysis of an *N*-benzyl group^43^. Initial experiments to identify conditions for cleavage of the *N*-benzyl group by classical procedures with Pd/C as catalyst, H_2_ as reagent and MeOH as solvent gave no N–H azetidine; instead, the corresponding *N*-methyl azetidine **9a** was obtained in 70% yield (Supplementary Fig. 18). The same reaction of three additional *N*-benzyl aziridines gave the analogous methylated azetidines in good yield (**9b**–**9d**).

To assess the applicability of this reaction to the formation of other *N*-alkyl aziridines, we conducted the debenzylation in a series of alcohols. The reaction in ethanol gave the ethylated azetidine **9e** in 84% yield, and the reaction in *i-*propanol gave the *N*-isopropyl product **9f** in a moderate 62% yield. The same process with cyclopropylmethanol led to reductive ring-opening, followed by alkylation of the azetidine to give **9g** in 50% yield. Discussion of the mechanism of these processes can be found in Supplementary Fig. 19 and accompanying legend.

### Derivatizations of *N*-benzyl azetidines

**Fig. 5:**
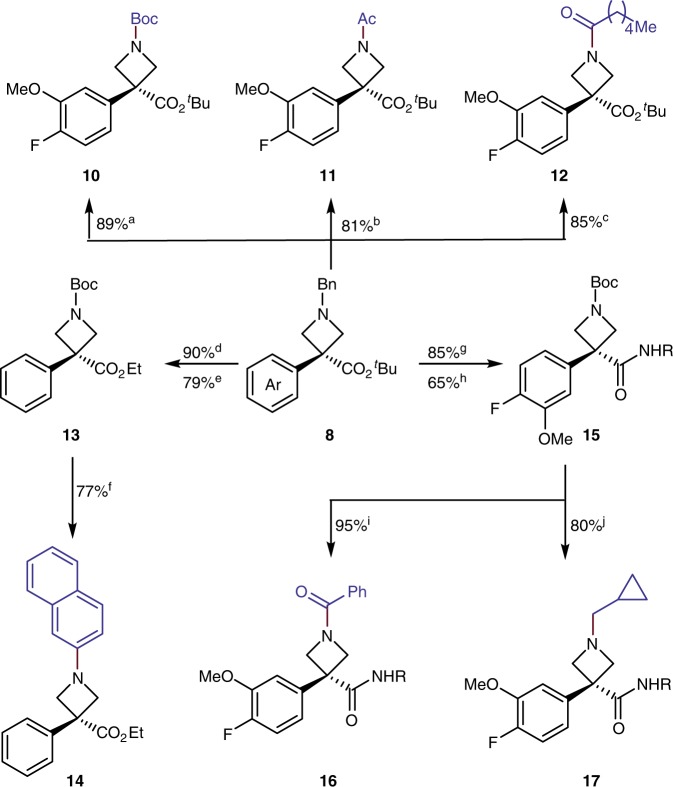
Derivatizations of arylated azetidine products. ^a^Pd(OH)_2_/C, Boc_2_O, MeOH, H_2_, RT. ^b^Pd(OH)_2_/C, Ac_2_O, MeOH, H_2_, RT. ^c^Pd(OH)_2_/C, hexanoic anhydride, MeOH, H_2_, RT. ^d^Step 1: HCl·dioxane, RT. Step 2: (COCl)_2_, DMF, CH_2_Cl_2_/EtOH, RT. ^e^Pd(OH)_2_/C, Boc_2_O, H_2_, MeOH, RT. ^f^Step 1: TFA/ CH_2_Cl_2_, RT. Step 2: 2-bromonaphthalene, Pd_2_(dba)_3_, Xantphos, Cs_2_CO_3_, dioxane, 100 ^°^C. ^g^Step 1: HCl·dioxane, RT. Step 2: 2-(4-methoxyphenyl)ethan-1-amine, HBTU, HOBt, DIPEA, DMF, RT. ^h^Pd(OH)_2_/C, Boc_2_O, H_2_, MeOH, RT. ^i^Step 1: TFA/ CH_2_Cl_2_, RT. Step 2: PhCOCl, Et_3_N, CH_2_Cl_2_, RT. ^j^Step 1: TFA/CH_2_Cl_2_, RT. Step 2: cyclopropanecarbaldehyde, Na(AcO)_3_BH, CH_2_Cl_2_, RT

Although this alkylation of the azetidine during hydrogenolysis of the *N*-benzyl linkage can be useful for installation of simple alkyl groups, a method was sought to form an azetidine with a conventional carbamate group that would allow for more diverse subsequent transformations. Fortunately, the alkylation process could be interrupted with an anhydride to capture the free azetidine. As shown in Fig. 5, hydrogenolysis of the *N*-benzyl group in the presence of carboxylic acid anhydrides led to the acylated azetidines. The carbamate, acetamide, and longer chain amides **10**–**12** were isolated in high yields (81–89%).

The Boc-protected azetidine proved to be a valuable precursor to the free amine as a synthetic intermediate. For example, the *N*-aryl azetidine **14** was obtained by cleaving the Boc group to form the free azetidine and conducting a palladium-catalyzed cross coupling of the resulting amine with an aryl halide. The combination of acid-catalyzed cleavage of the *N*-Boc linkage and acylation with benzoyl chloride gave the *N*-benzoyl azetidine **16**, whereas cleavage and reductive amination with cyclopropanecarbaldehyde gave the *N*-cyclopropylmethyl azetidine **17**. Overall, the combination of α-arylation, conversion of the *t*-butyl ester to any of the functional groups accessible from an ester, and derivatization of the free nitrogen after deprotection generates an azetidine core containing a wide range of substituents at three positions.

### Application to biologically relevant molecules

**Fig. 6:**
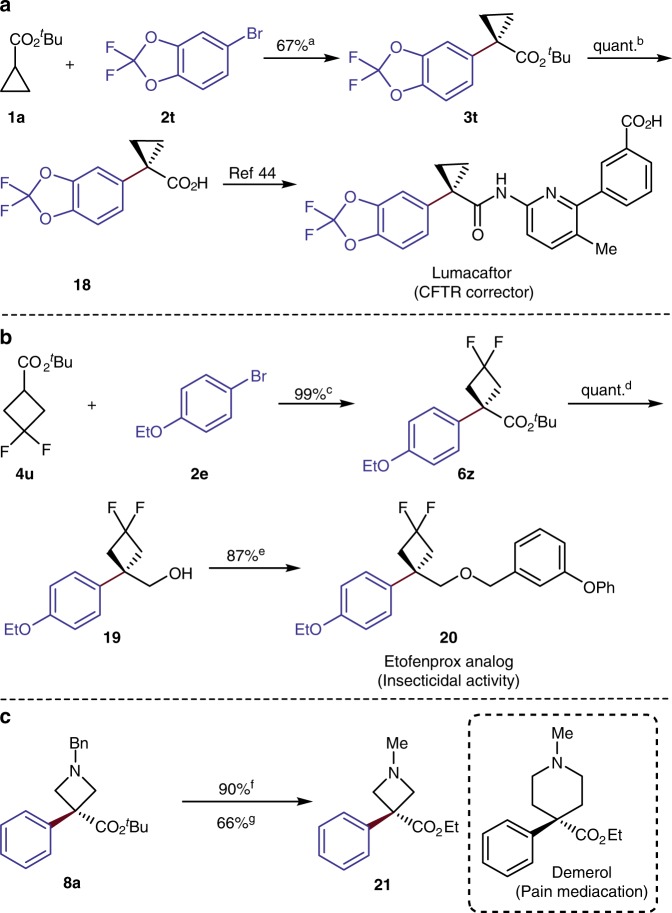
Applications to biologically relevant molecules. **a** Synthesis of Lumacaftor. **b** Synthesis of Etofenprox analog. **c** Synthesis of Demerol analog. ^a^Standard condition as shown in Fig. 2 without using AgBF_4_. ^b^TFA, Et_3_SiH, CH_2_Cl_2_, RT. ^c^Standard condition as shown in Fig. 3 without using AgOTf. ^d^LiAlH_4_, Et_2_O, 0 ^°^C – RT. ^e^NaH, 1-(bromomethyl)-3-phenoxybenzene, THF, 0 ^°^C – RT. ^f^Step 1: HCl·dioxane, RT. Step 2: (COCl)_2_, DMF, CH_2_Cl_2_/EtOH, RT. ^g^Pd/C, H_2_, CH_3_OH, RT. quant.: quantative

To illustrate the synthetic potential of these α-arylations of small rings, we prepared three biologically relevant molecules by short sequences that include an α-arylation (Fig. 6). The commercial drug and CFTR corrector Lumacaftor illustrates the value of the α-arylation of a cyclopropane^9^. The reported synthesis required four steps to reach the key intermediate **18**. Our method afforded **18** in high yield over just two steps (Fig. 6a)^44^. The analog **20** of Etofenprox, studied for potential insecticidal activity illustrates the value of the α-arylation of a cyclobutane. Sequential α-arylation, reduction, and nucleophilic substitution reactions gave **20** in 87% overall yield (Fig. 6b)^45^. Likewise, preparation of compound **21**, an analog of the commercial pain medication named Demerol^46^, illustrated one value of the α-arylation of an azetidine. Compound **21** was easily prepared from α-arylation product **8a** by ester exchange and the one-step debenzylation-methylation reaction (Fig. 6c).

### Preliminary mechanistic studies

**Fig. 7:**
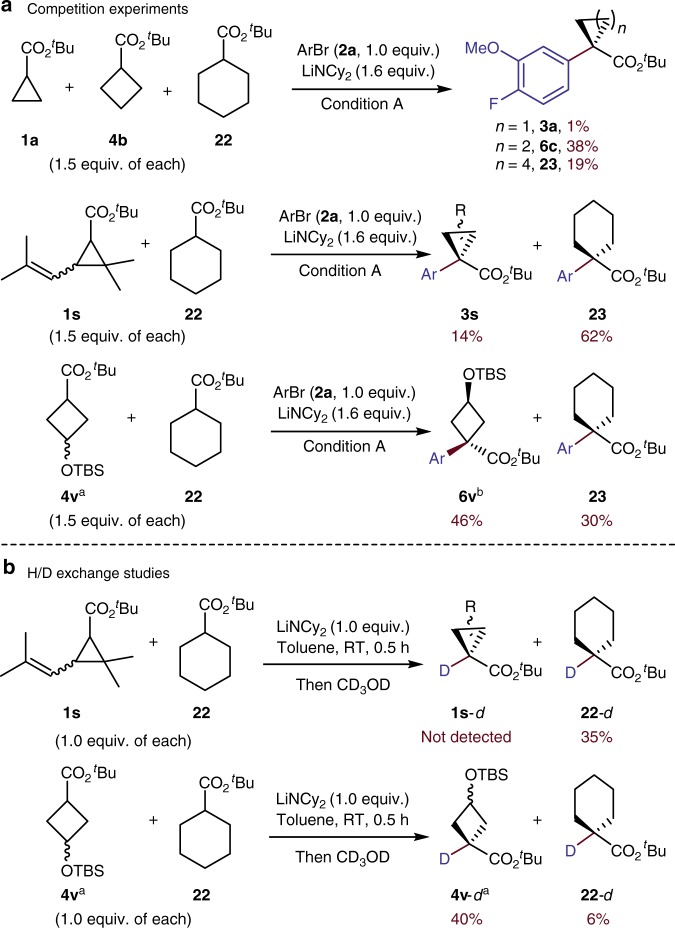
Competition experiments and H/D exchange studies. **a** Competition experiments. **b** H/D exchange studies. Condition A: 4-bromo-1-fluoro-2-methoxybenzene (**2a**, 1.0 equiv.), **[Pd]−5** (5 mol%), AgBF_4_ (5 mol%), LiNCy_2_ (1.6 equiv.), toluene, 50 ^°^C, 12 h. ^a^Mixtures of two isomers, dr 9:1. ^b^Major isomer is shown, dr 3:1

Because reproducible, high-yield α-arylation reactions of the small-ring carboxylic acids had not been reported previously, we conducted a series of mechanistic experiments to reveal more clearly the reaction parameters that led to the successful development of these reactions. We first compared the relative rates of reactions of the esters containing different ring sizes. To do so, we conducted the reactions of the cyclopropyl ester **1a**, cyclobutyl ester **4b**, and cyclohexyl ester **22** (Fig. 7a) in the same vessel. The yield of the α-arylation of cyclobutyl ester **4b** was twice that of cyclohexyl ester **22**, and only trace amounts of product from arylation of cyclopropyl ester **1a** were detected. This trend in reactivity (cyclobutyl > cyclohexyl > cyclopropyl ester) was also observed from a pairwise competition of the cyclohexyl ester with substituted, small-ring carboxylic acid esters. The reaction of 4-bromo-1-fluoro-2-methoxybenzene **2a** with the combination of the substituted cyclopropane **1s** and cyclohexane carboxylate **22** gave the cyclopropyl product **3s** in 14% yield and the cyclohexyl product **23** in 62% yield; the arylation of the combination of siloxy-substituted **4v** and **22** gave cyclobutyl product **6v** in 46% yield and cyclohexyl product **23** in 30% yield. Thus, the relative reactivity observed for the parent ring systems translated to substituted versions of the carbocyclic esters.

Previously, our group showed that a series of arylpalladium enolates underwent reductive elimination with nearly identical rates^47^. This result implies that the trend in reactivity of the different sizes of carbocyclic esters more likely reflects the trend in stability of the corresponding enolates than the reactivity of the palladium enolates^23^. H/D exchange experiments were conducted to test this hypothesis. The relative propensities of the cyclopropyl, cyclobutyl, and cyclohexyl carboxylic esters to undergo deprotonation were assessed by addition of limiting base to an excess of two esters, followed by quenching with CD_3_OD. This experiment with equimolar amounts of LiNCy_2_, cyclohexyl ester **22** and cyclopropyl ester **1s** at room temperature in a glovebox, gave the deuterated cyclohexyl ester **22-***d* in 35% yield and no detectable deuterated cyclopropyl ester **1s**-*d* (Fig. 7b). The same experiment conducted with cyclohexyl ester **22** and cyclobutyl ester **4v** gave more deuterated cyclobutyl ester **4v**-*d* (40%) than cyclohexyl ester **22**-*d* (6%). These results imply that the rate or equilibrium for deprotonation of these enolates follows the order: cyclobutyl > cyclohexyl > cyclopropyl. This order reflects their corresponding acidity and matches the order of reactivity toward the α-arylation described above. Thus, the low acidity of the cyclopropyl ester is one property of this ester that makes its coupling reactions challenging.

**Fig. 8:**
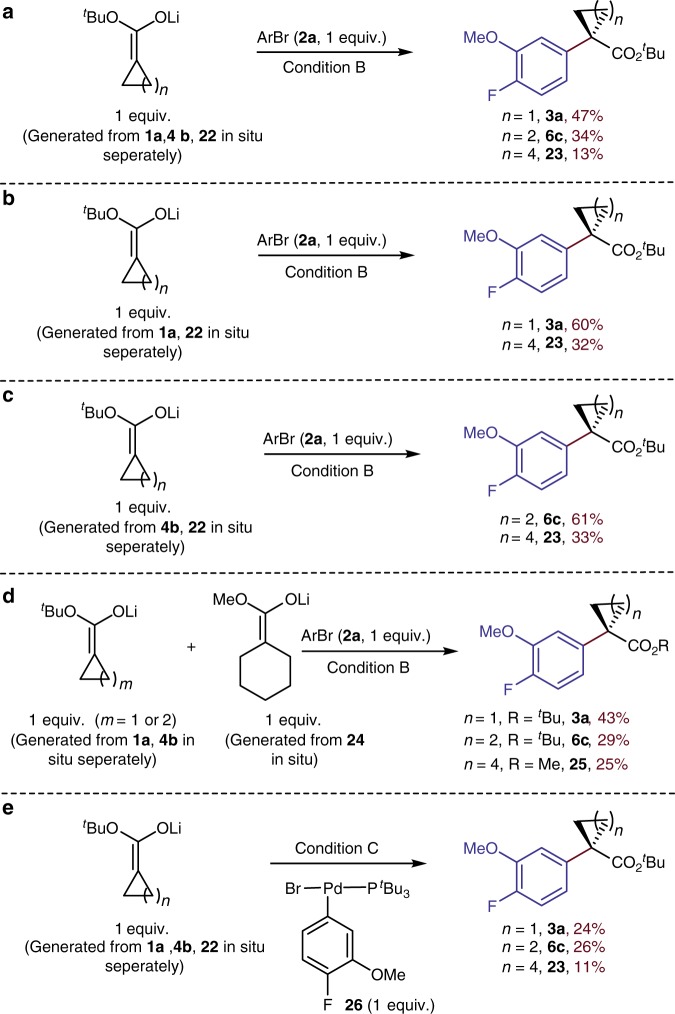
Studies on transmetallation. **a** Competition experiments among enolates of cyclopropyl, cyclobutyl and cyclohexyl *t*-butyl esters. **b** Competition experiments among enolates of cyclopropyl, and cyclohexyl *t*-butyl esters. **c** Competition experiments among enolates of cyclobutyl and cyclohexyl *t*-butyl esters. **d** Competition experiments among enolates of cyclopropyl, cyclobutyl *t*-butyl esters and cyclohexyl methyl esters. **e** Competition experiments among enolates of cyclopropyl, cyclobutyl and cyclohexyl *t*-butyl esters with **26** as catalyst. Condition B: ester (**1a**, or **4b**, or **22**, or **24**, 1 equiv.) with LiNCy_2_ (1.05 equiv.) and toluene, 4-bromo-1-fluoro-2-methoxybenzene (**2a**, 1 equiv.), **[Pd]−5** (5 mol%), AgBF_4_ (5 mol%) at 65 ^°^C for 6 h. Condition C: ester (**1a**, or **4b**, or **22**, 1 equiv.) with LiNCy_2_ (1.05 equiv.) and toluene, **26** (1 equiv.) at 65 ^°^C for 6 h

Additional experiments revealed the effect of the ring size on the rates of transmetallation of the ester enolates (Fig. 8). Three different *O*-bound lithium enolates^48^ were generated in situ separately from 1 equiv. of *t*-butyl ester (**1a**, or **4b**, or **22**) and a slight excess of LiNCy_2_ in toluene for 15 min. Reaction of a mixture of the three enolates with 1 equiv. of aryl bromide **2a** and 5 mol % catalyst at 65 ^°^C for 6 h formed the products from cyclopropane and cyclobutane in 47% and 34% yield, respectively, and only 13% of the product from the cyclohexyl ester (Fig. 8a). This trend was corroborated by similar reactions of aryl bromide **2a** with enolates generated from cyclopropyl ester **1a** and cyclohexyl ester **22**, which gave the corresponding products in 60% and 32% yield respectively (Fig. 8b), and by reactions of **2a** with enolates from cyclobutyl ester **4b** and cyclohexyl ester **22**, which gave the corresponding products in 61% and 33% yield, respectively (Fig. 8c). Even the reaction of the less hindered enolate from the cyclohexyl methyl ester **24**, instead of that from the cyclohexyl *t*-butyl ester **22**, formed less of the product from this ester (25%) than that from the enolates of the smaller-ring esters (Fig. 8d, 43% and 29%).

Single-turnover experiments involving the reaction of the lithium enolates with the arylpalladium complex **26** were also conducted to obtain direct information on the rates of transmetallation. Like the catalytic reactions, these reactions of the isolated arylpalladium halide complex with the combination of the lithium enolates from the three esters formed the products of the small ring esters in larger amounts than the products from the larger-ring ester (Fig. 8e).

The final set of experiments on the mechanism of the coupling of the small-ring carbocycles involved assessing the stability of the esters in the presence of the bases and solvents that are typically used to generate the enolates during the α-arylation reactions. As noted in the introduction, prior α-arylations of α,α-disubstituted esters required the smaller methyl ester, rather than the larger *t*-butyl ester. Thus, we tested the stabilities of the methyl and *t*-butyl versions of the cyclic esters in this study. The esters can decompose in the presence of base by Claisen condensation or formation of ketene.

**Fig. 9:**
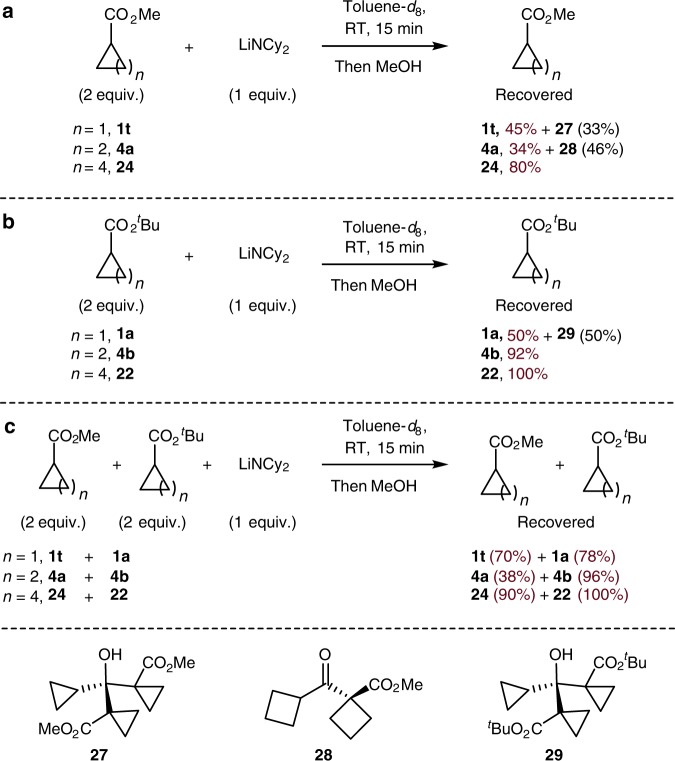
Relative rates for Claisen condensation. **a** Claisen condensation test for cyclopropyl, cyclobutyl or cyclohexyl methyl esters individually. **b** Claisen condensation test for cyclopropyl, cyclobutyl or cyclohexyl *t*-butyl esters individually. **c** Claisen condensation test between methyl ester and related *t*-butyl ester

Two equiv. of the methyl esters and one equiv. of base in toluene-*d*_8_ were mixed at RT for 15 min and then quenched with an excess of methanol (Fig. 9a). Cyclopropyl methyl ester **1t** was recovered in 45% yield, along with 33% of side product **27** from condensation. Cyclobutyl methyl ester **4a** was recovered in an even lower 34% yield, with a higher 46% yield of side product **28** from condensation. In contrast, cyclohexyl methyl ester **24** was recovered in 80% yield. These data show that the methyl esters of small carbocycles are sensitive to base and are not well suited for direct α-arylation.

The same experiments of the *t*-butyl esters showed stabilities that matched with the difficulty in identifying conditions for the coupling reactions (Fig. 9b). Only 50% of the cyclopropyl *t*-butyl ester **1a** was recovered by quenching of the enolate after the same 15 min at room temperature; the other 50% of the material was product **29** formed from condensation of the ester. This observation underscores the propensity of cyclopropyl esters to undergo condensation under the conditions of the coupling process and why the reaction required the most efficient generation of a highly active catalyst. In contrast, cyclobutyl *t*-butyl ester **4b** was recovered in a high 92% yield from the same experiment, implying that reactions of cyclobutyl *t*-butyl esters are more forgiving than those of the cyclopropyl esters. As expected, the cyclohexyl *t*-butyl ester was fully recovered.

The same type of experiment with a mixture of the two esters was consistent with the stability of the individual esters (Fig. 9c). In these mixtures, the cyclopropyl methyl and *t*-butyl esters were the least stable (70% remaining of methyl ester **1t**, 78% of the *t*-butyl ester **1a**). The cyclobutyl *t*-butyl ester was almost completely stable (96% of **4b** recovered), but the cyclobutyl methyl ester was unstable (38% of **4a** recovered); Both the cyclohexyl methyl and cyclohexyl *t*-butyl esters were relatively stable (over 90% of each was recovered).

**Fig. 10:**
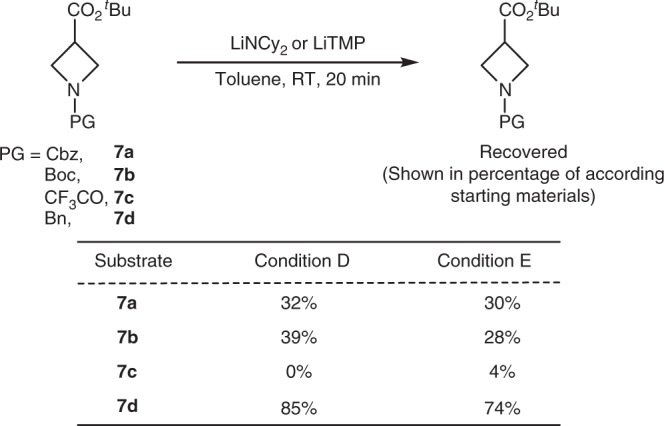
Stability of different azetidine esters. Condition D: PG-azetidine (1 equiv.), LiNCy_2_ (2 equiv.), toluene, RT, 20 min. Then MeOH; Condition E: PG-azetidine (1 equiv.), LiTMP (1.05 equiv.), toluene, RT, 20 min. Then MeOH. PG: protecting group

Related experiments with the azetidine carboxylic ester showed the importance of electron-donating protecting groups on the nitrogen (Fig. 10). When the azetidine bears an electron-withdrawing acyl or carbamoyl group on nitrogen, it is unstable to the basic conditions, presumably by a process involving ring opening. For example, when the azetidine ester was mixed with LiNCy_2_ in toluene at RT for 20 min, azetidines **7a**–**7c** containing a carbonyl group on nitrogen were recovered in less than 39% yield while Bn-protected azetidine (**7d**) was recovered in 85% yield (Fig. 10, condition D). Similar results were observed with the LiTMP base that we used in the reaction conditions to obtain the highest yields of the product from α-arylation of azetidine. The combination of the azetidine carboxylic ester **7d** containing a more electron-donating Bn group as the protecting group and the LiTMP base led to 74% recovery of the azetidine. In contrast, the same experiment with **7a**–**7c** containing electron-withdrawing protecting groups led to less than 30% recovery of the azetidine (Fig. 10, condition E).

These data showing the instability of the cyclopropyl ester, the cyclobutyl methyl ester, and the azetidines containing carbonyl groups on nitrogen help explain the absence of these α-arylation reactions in prior literature. To achieve these coupling reactions, the coupling should be conducted with a *t*-butyl ester of cyclopropane and cyclobutane and an *N*-alkyl azetidine, and they should be conducted with catalysts that are sufficiently reactive to form the coupled product with a rate that exceeds that of the decomposition of the ester and competing Claisen condensation. Overall, these data highlight the challenge of coupling small ring esters and why highly active catalysts and substituents that minimize decomposition are necessary.

## Discussion

In summary, systems and conditions have been identified for broadly applicable, direct α-arylation of aromatic and heteroaromatic electrophiles with small ring esters, including cyclopropyl esters, cyclobutyl esters and azetidine esters. All three classes of arylations occurred in yields up to 99%. Mechanistic studies indicate that the weak acidity of the α C-H bond in cyclopropyl esters, strong sensitivity of the strained esters to Claisen condensation, particularly for cyclobutyl esters, and facile decomposition of the enolates of azetidinyl esters were the major challenges we overcame when developing these couplings with small ring esters. Deprotection of the *N*-benzyl group led to an interesting debenzylation-alkylation process of the arylated azetidine. The reliability and generality of this coupling method were showcased by three short syntheses of biologically relevant molecules. Further studies on the coupling of small-ring enolates are in progress.

## Methods

### General procedure for α-arylations of cyclopropyl esters

In a dry and N_2_-filled glovebox, the small ring ester **1** (0.20 mmol) in toluene (0.3 mL) was added dropwise at room temperature to a 4-mL vial containing solid LiNCy_2_ (39 mg, 0.21 mmol). The resulting mixture was allowed to stir for 15 min at RT. To a second vial containing **[Pd]−5** (2.6 mg, 0.0050 mmol) and AgBF_4_ (1.0 mg, 0.0050 mmol) were added aryl bromide **2** (0.10 mmol) and toluene (0.1 mL). The resulting mixture was shaken by hand for 30 s. Then, the solutions in these two vials above were combined. The vial was sealed with a PTFE-lined cap, removed from the dry box, and stirred at 65 ^°^C for 12 h. The reaction solution was condensed and purified by flash column chromatography to afford the pure product **3**.

### General procedure for α-arylations of cyclobutyl esters

In a dry and N_2_-filled glovebox, the small ring ester **4** (0.15 mmol) in toluene (0.3 mL) was added dropwise at room temperature to a 4-mL vial containing solid LiNCy_2_ (30 mg, 0.16 mmol). The resulting mixture was allowed to stir for 15 min at RT. To a second vial containing **[Pd]−5** (0.5 mg, 0.001 mmol) and AgOTf (0.6 mg, 0.001 mmol), were added aryl bromide **2** (0.10 mmol) and toluene (0.1 mL). The resulting mixture was shaken by hand for 30 s. Then, the solutions in these two vials above were combined. The vial was sealed with a PTFE-lined cap, removed from the dry box, and stirred at 50 ^°^C for 12 h. The reaction solution was condensed and purified by flash column chromatography to afford the pure product **6**.

### General procedure for the α-arylations of azetidine esters

In a dry and N_2_-filled glovebox, the small ring ester **7d** (0.15 mmol) in toluene (0.3 mL) was added dropwise at room temperature to a 4 mL vial containing solid LiTMP (23 mg, 0.16 mmol). The resulting mixture was allowed to stir for 15 min at RT. To a second vial containing Pd(dba)_2_ (2.9 mg, 0.0050 mmol) and ^*t*^Bu_3_P (1 M in toluene, 5.0 μL, 0.0050 mmol) were added aryl bromide **2** (0.10 mmol) and toluene (0.1 mL). Then, the solutions in these two vials above were combined. The vial was sealed with a PTFE-lined cap, removed from the dry box, and stirred at 50 ^°^C for 12 h. The reaction solution was condensed and purified by flash column chromatography to afford the pure product **8**.

## Supplementary information

Supplementary Information

## Data Availability

For experimental details and procedures, spectra for all unknown compounds, see supplementary files. The X-ray crystallographic data for **6r** (CCDC 1823271), have been deposited at the Cambridge Crystallographic Data Center (www.ccdc.cam.ac.uk/data_request/cif). All data underlying the findings of this study are available from the corresponding author upon reasonable request.
