# Supplementary material for: Palladium-catalyzed α-arylation for the addition of small rings to aromatic compounds
Source: Nat Commun. 2019 Sep 9;10:4083. doi: 10.1038/s41467-019-12090-z (PMC6733931; doi:10.1038/s41467-019-12090-z)
Supplement: Supplementary file 1 — Supplementary Information [file 41467_2019_12090_MOESM1_ESM.pdf]

Supplementary Information for

Palladium-Catalyzed α-Arylation for the Addition of Small Rings to Aromatic Compounds

He et al

Supplementary Tables

Supplementary Table 1. Development of the α-arylations of cyclopropyl esters

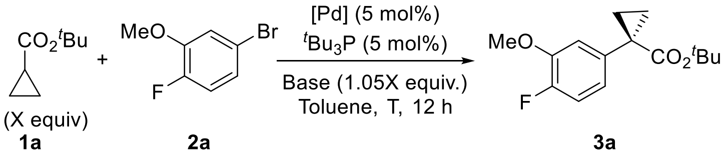

Entry	[Pd]	Base	X	T/°C	Yield[a]
1	$Pd(dba)_2$	$LiNCy_2$	1.3	50	38%
2	$Pd(dba)_2$ and 13 alternative ligands	$LiNCy_2$	1.3	50	< 22%
3	$Pd(dba)_2$	LiTMP	1.3	50	29%
4	$Pd(dba)_2$	LiHMDS	1.3	50	N.D.
5	$Pd(dba)_2$	$Zn(TMP)_2$	1.3	50	N.D.
6	$Pd(dba)_2$	$LiNCy_2$	1.3	65	44%
7	$Pd(dba)_2$	$LiNCy_2$	1.3	80	38%
8	$Pd(dba)_2$	$LiNCy_2$	1.5	50	36%
9	$Pd(dba)_2$	$LiNCy_2$	2.0	50	57%
10	$Pd(dba)_2$	$LiNCy_2$	2.5	50	52%
11	$Pd(dba)_2$	$LiNCy_2$	2.0	65	63%
12	$Pd(dba)_2$ and 25 alternative ligands	$LiNCy_2$	2.0	65	< 52%
13	**[Pd]-1**	$LiNCy_2$	2.0	65	30%
14	**[Pd]-2**	$LiNCy_2$	2.0	65	30%
15[b]	**[Pd]-3**	$LiNCy_2$	2.0	65	10%
16	**[Pd]-4**	$LiNCy_2$	2.0	65	84%
17[b]	**[Pd]-5**	$LiNCy_2$	2.0	65	86%
18[b,c]	**[Pd]-5**	$LiNCy_2$	2.0	65	99%[d]

[a]Yield were determined by crude ^{19}F NMR with fluorobenzene as internal standard. [b]P^tBu_3 was not used. [c]$AgBF_4$ (5 mol%) was used as additives [d]Isolated yield. N.D., not detected. Details of this procedure are described in Supplementary Figure 15.

Supplementary Table 2. Development of the α-arylations of cyclobutyl esters

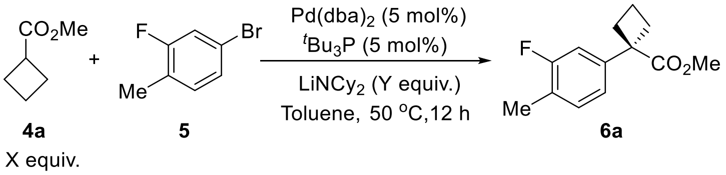

Entry	X	Y	T/°C	Conversion of **1a**[a]	Yield[a]
1[b]	1.2	1.3	RT	14%	N.D.
2[b]	1.2	1.3	50	32%	N.D.
3	1.2	1.3	50	70%	24%
4	1.2	1.3	80	93%	22%
5	1.5	1.6	50	100%	29%

[a]Determined by ^{19}F NMR using fluorobenzene as internal standard. [b]2 mol% Pd(dba)$_2$ and ^{t}Bu$_3$P were used. N.D., not detected.

Supplementary Table 3. Development of the α-arylations of azetidine esters

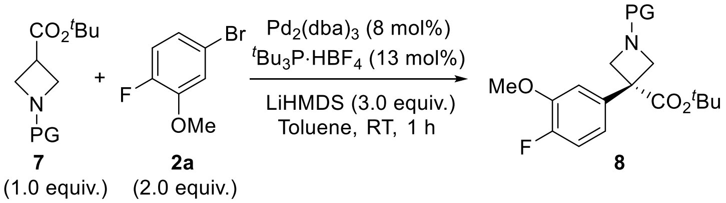

Entry	PG	Yield of **8**	**7** remaining	**2a** remaining
1[b]	Cbz	17%	7%	Not determined
2	Cbz	14%	6%	1.3 equiv.
3	Boc	37%	N.D.	0.92 equiv.
4	CF_3CO	N.D.	N.D.	1.2 equiv.
5[c]	Boc	3%	28%	1.6 equiv.

[a]These experiments were carried out with the same conditions reported in the only case in a patent. All of the yields were determined by crude ^{1}H NMR or ^{19}F NMR. [b]PhBr was used instead of 2a. [c]2.5 mol% Pd$_2$(dba)$_3$ and 5 mol% L were used. N.D. = not detected.

Supplementary Table 4. Further studies of the α-arylations of azetidine esters

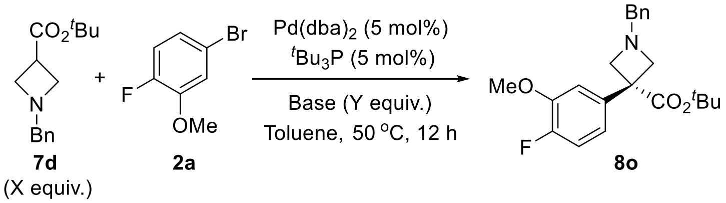

Entry	Base	X	Y	Yield of **8o**[a]
1[b]	LiHMDS	1.1	1.2	N.D.
2	LiNCy$_2$	1.1	1.2	43%
3	LiTMP	1.1	1.2	55%
4	Zn(TMP)$_2$	1.1	1.2	55%
5	LiTMP	1.3	1.4	63%
6	LiTMP	1.5	1.6	88%[c]

[a]Yields were determined by crude ^{19}F NMR spectroscopy. [b]67% yield of the product from C-N coupling between ArBr and LiHMDS, 32% of the azetidine ester was left after reaction. [c]Isolated yield. N.D., not detected. Details of this procedure are described in Supplementary Figure 17.

Supplementary Table 5. Data for X-ray diffraction of 6r

Crystal data and structure refinement for **6r**.

Identification code	**6r**	
Empirical formula	$C_{17} H_{20} O_2 S$	
Formula weight	288.39	
Temperature	100(2) K	
Wavelength	0.71073 Å	
Crystal system	Monoclinic	
Space group	P 21/c	
Unit cell dimensions	a = 11.3021(5) Å	α= 90°.
	b = 17.4804(8) Å	β= 94.899(2)°.
	c = 7.7333(4) Å	γ = 90°.
Volume	1522.25(12) $Å^3$	
Z	4	
Density (calculated)	1.258 Mg/m^3	
Absorption coefficient	0.212 mm^{-1}	
F(000)	616	
Crystal size	0.080 x 0.040 x 0.040 mm^3	
Theta range for data collection	1.808 to 31.617°.	
Index ranges	-14<=h<=16, -25<=k<=24, -10<=l<=10	
Reflections collected	55213	
Independent reflections	4882 [R(int) = 0.0373]	
Completeness to theta = 25.000°	100.0 %	
Absorption correction	Semi-empirical from equivalents	
Max. and min. transmission	0.746 and 0.721	
Refinement method	Full-matrix least-squares on F^2	
Data / restraints / parameters	4882 / 0 / 184	
Goodness-of-fit on F^2	1.041	
Final R indices [I>2sigma(I)]	R1 = 0.0417, wR2 = 0.1065	
R indices (all data)	R1 = 0.0558, wR2 = 0.1138	
Extinction coefficient	n/a	
Largest diff. peak and hole	0.438 and -0.324 e.$Å^{-3}$	

These data (CCDC 1823271) can be obtained free of charge from The Cambridge Crystallographic Data Centre via www.ccdc.cam.ac.uk/data_request/cif.

Supplementary Methods

General information

All air-sensitive procedures were conducted in a nitrogen-filled glovebox or by Schlenk techniques under nitrogen. All dry solvents were obtained by passing them through a solvent column composed of activated A-1 alumina and further degassing them by freeze-pump-thaw methods. Unless otherwise indicated, all commercially available starting materials were purchased and used directly without further purification. ^{1}H, ^{13}C, ^{19}F NMR spectra were acquired on 400 MHz or 600 MHz Bruker instruments at the University of California, Berkeley. Chemical shifts are reported in δ (ppm) with reference to residual solvent peaks ($CHCl_3$ in $CDCl_3$: 7.26 ppm for ^{1}H NMR and 77.10 ppm for ^{13}C NMR, $CFCl_3$: 0 ppm for ^{19}F NMR). Coupling constants (*J*) are reported in Hz.

Synthesis of substrates

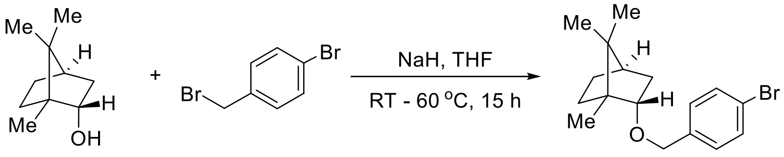

Supplementary Figure 1. Synthesis of substrate **2l**

(1*R*,2*S*)-2-((4-Bromobenzyl)oxy)-1,7,7-trimethylbicyclo[2.2.1]heptane (**2l**): To a well-stirred solution of NaH (60%, dispersion in oil, 0.22 g, 5.5 mmol) in THF (10 mL) under nitrogen at room temperature was added alcohol **30** (0.77 g, 5.0 mmol) in THF (5 mL) dropwise. The solution was stirred at RT for 30 min. Then, **31** (1.3 g, 5.0 mmol) in THF (5 mL) was added to the resulting solution dropwise. The final mixture was stirred at RT for 12 h and then 60 °C for 3 h. After this time, the reaction solution was condensed, and the residue was purified by flash column chromatography (hexane/ethyl acetate = 50/1) to afford the pure **2l** (0.48 g, 30% yield) as a colorless oil. ^{1}H NMR (600 MHz, Chloroform-*d*) δ 7.45 (d, *J* = 8.1 Hz, 2H), 7.22 (d, *J* = 8.2 Hz, 2H), 4.52 (d, *J* = 12.4 Hz, 1H), 4.39 (d, *J* = 12.4 Hz, 1H), 3.67 (d, *J* = 9.2 Hz, 1H), 2.15 – 2.04 (m, 2H), 1.74 – 1.69 (m, 1H), 1.65 (t, *J* = 4.5 Hz, 1H), 1.28 – 1.22 (m, 2H), 1.08 (dd, *J* = 13.0, 3.1 Hz, 1H), 0.89 (s, 3H), 0.85 (s, 3H), 0.83 (s, 3H); ^{13}C NMR (151 MHz, $CDCl_3$) δ 138.66, 131.38, 128.93, 121.00, 84.58, 70.88, 49.39, 47.96, 45.10, 36.17, 28.34, 26.83, 19.86, 18.96, 14.12; HRMS (ESI): $[M+Na]^{\oplus}$ calcd for $C_{17}H_{23}OBrNa^{\oplus}$ 345.0824, found 345.0836.

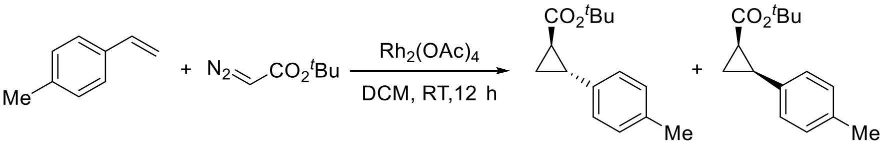

Supplementary Figure 2. Synthesis of substrate **1r**

To a well-stirred solution of **32** (0.98 mL, 7.4 mmol) and $Rh_2(OAc)_4$ (66 mg, 0.15 mmol) in DCM (10 mL) at room temperature was added **33** (15% in toluene, 4.0 mL, 3.7 mmol), followed by additional DCM (10 mL). The resulting mixture was stirred for 12 h. Then, the reaction solution was condensed, and the

residue was purified by flash column chromatography (hexane/ethyl acetate = 50/1) to afford the pure *trans*-**1r** (0.44 g, 54% yield) and *cis*-**1r** (0.24 g, 30% yield) as a colorless oil.

trans-**1r**: ^{1}H NMR (600 MHz, $CDCl_3$) δ 7.08 (d, J = 7.9 Hz, 2H), 6.99 (d, J = 7.9 Hz, 2H), 2.44 – 2.37 (m, 1H), 2.31 (s, 3H), 1.82 – 1.76 (m, 1H), 1.50 (dt, J = 9.4, 4.9 Hz, 1H), 1.46 (s, 9H), 1.20 (ddd, J = 8.3, 6.4, 4.6 Hz, 1H); ^{13}C NMR (151 MHz, $CDCl_3$) δ 172.76, 137.53, 136.00, 129.17, 126.10, 80.54, 28.26, 25.58, 25.25, 21.06, 16.97; HRMS (ESI): [M+Na]$^{\oplus}$ calcd for $C_{15}H_{20}O_2Na^{\oplus}$ 255.1355, found 255.1355.

cis-**1r**: ^{1}H NMR (600 MHz, $CDCl_3$) δ 7.16 (d, J = 7.8 Hz, 2H), 7.06 (d, J = 7.8 Hz, 2H), 2.49 (dd, J = 16.8, 8.5 Hz, 1H), 2.30 (s, 3H), 1.98 – 1.92 (m, 1H), 1.60 (dd, J = 12.4, 5.5 Hz, 1H), 1.21 (td, J = 8.2, 5.0 Hz, 1H), 1.17 (s, 9H); ^{13}C NMR (151 MHz, $CDCl_3$) δ 170.32, 136.02, 133.83, 129.42, 128.56, 80.04, 27.91, 24.85, 22.70, 21.13, 10.70; HRMS (ESI): [M+Na]$^{\oplus}$ calcd for $C_{15}H_{20}O_2Na^{\oplus}$ 255.1355, found255.1355.

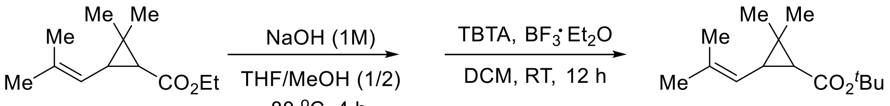

34 **1s**

Supplementary Figure 3. Synthesis of substrate **1s**

***tert*-Butyl 2,2-dimethyl-3-(2-methylprop-1-en-1-yl)cyclopropane-1-carboxylate** (**1s**): To a well-stirred solution of **34** (2.00 g, 10.2 mmol) in THF/MeOH (40 mL/80 mL) at room temperature was added 42 mL of a 1M aqueous solution of NaOH. The resulting mixture was allowed to reflux at 80 °C for 4 h. After this time, the reaction was quenched with water (50 mL) and extracted with Et_2O three times (50 mL × 3). The organic layer was dried with anhydrous $MgSO_4$ and condensed to afford 1.8 g white solid, which was used directly for the second step. The second step was conducted with a synthetic procedure that was the same as that used to prepare **4r**. The product was obtained by flash column chromatography (hexane/ethyl acetate = 60/1) as a colorless oil (1.1 g, 48% yield over the two steps) as a mixture of two isomers (ratio 1:0.14). Major isomer: ^{1}H NMR (400 MHz, $CDCl_3$) δ 4.87 (d, J = 6.6 Hz, 1H), 1.96 (br s, 1H), 1.70 (s, 6H), 1.44 (s, 9H), 1.30 (d, J = 1.6 Hz, 1H), 1.23 (d, J = 1.9 Hz, 3H), 1.11 (d, J = 1.9 Hz, 3H); ^{13}C NMR (151 MHz, $CDCl_3$) δ 171.80, 135.11, 121.60, 79.98, 36.01, 31.85, 28.34, 27.94, 25.61, 22.34, 20.36, 18.49; HRMS (ESI): [M+Na]$^{\oplus}$ calcd for $C_{14}H_{24}O_2Na^{\oplus}$ 247.1668, found 247.1673.

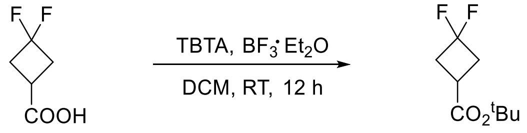

35 **4u**

Supplementary Figure 4. Synthesis of substrate **4u**

***tert*-Butyl 3,3-difluorocyclobutane-1-carboxylate** (**4u**): To a well-stirred solution of **35** (0.68 g, 5.0 mmol) in DCM (20 mL) under N_2 atmosphere at room temperature was added *tert*-butyl 2,2,2-trichloroacetimidate (TBTA) (2.19 g, 10.0 mmol). Then, borontrifluoride etherate (21 mg, 0.15 mmol) was added to the above solution dropwise. The resulting mixture was allowed to stir at room temperature for 12 h. After this time, the reaction solution was condensed and purified by flash column chromatography (pentane/diethyl ether = 80/1) directly to afford the pure substrate **4u** (0.73 g) as a colorless oil in 76% yield. ^{1}H NMR (600 MHz, $CDCl_3$) δ 2.85 – 2.74 (m, 5H), 1.46 (s, 9H); ^{13}C NMR (151 MHz, $CDCl_3$) δ 172.57, 119.00 (dd, J_{C-F} = 285.4 Hz, J_{C-F} = 271.8 Hz), 81.34, 38.74 (dd, J_{C-F} = 24.2,

$J_{C\text{-}F}$ = 24.2 Hz), 28.03, 27.57 (dd, $J_{C\text{-}F}$ = 15.1 Hz, $J_{C\text{-}F}$ = 6.0 Hz); ^{19}F NMR (376 MHz) δ -82.02 – -82.58 (m, 1F), -95.89 – -96.40 (m, 1F); HRMS (ESI): [M+Na]$^{\oplus}$ calcd for $C_9H_{14}F_2O_2Na^{\oplus}$ 215.0854, found 215.0860.

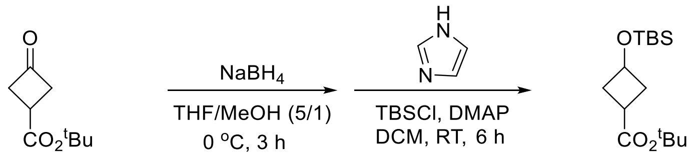

36 4v

Supplementary Figure 5. Synthesis of substrate **4v**

***tert*-Butyl 3-((*tert*-butyldimethylsilyl)oxy)cyclobutane-1-carboxylate** (**4v**): To a well-stirred solution of $NaBH_4$ (89.0 mg, 2.35 mmol) in THF (2 mL) at room temperature was added **36** (400 mg, 2.35 mmol) in THF/MeOH (6 mL/2 mL) dropwise at 0 °C. The resulting mixture was allowed to stir for another 3 h. The reaction was quenched by sat. aqueous Na_2CO_3 (20 mL) and extracted with ethyl acetate (20 mL × 3). The organic layer was dried with anhydrous $MgSO_4$ and condensed for use in the next step. To a 25 mL bottle with above compound in dry DCM (8 mL) was added TBSCl (531 mg, 3.53 mmol), DMAP (58 mg, 0.47 mmol) and 1*H*-imidazole (800 mg, 11.8 mmol) at room temperature. Then the reaction was allowed to stir for 6 h at room temperature. The reaction solution was condensed, and the residue was purified by flash column chromatography (hexane/ethyl acetate = 40/1) to afford the pure substrate **4v** (660 mg) as a colorless oil in 98% yield. ^{1}H NMR (600 MHz, $CDCl_3$) δ 4.12 – 4.08 (m, 1H), 2.44 – 2.39 (m, 3H), 2.24 – 2.10 (m, 2H), 1.43 (s, 9H), 0.87 (s, 9H), 0.03 (s, 6H); ^{13}C NMR (151 MHz, $CDCl_3$) δ 174.00, 80.11, 63.02, 37.25, 30.15, 28.12, 25.88, 18.06, -4.70; HRMS (ESI): [M+Na]$^{\oplus}$ calcd for $C_{15}H_{30}O_3SiNa^{\oplus}$ 309.1856, found 309.1861.

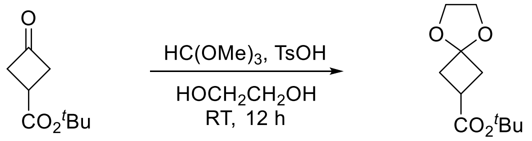

37 4w

Supplementary Figure 6. Synthesis of substrate **4w**

***tert*-Butyl 5,8-dioxaspiro[3.4]octane-2-carboxylate** (**4w**): To a well-stirred solution of **37** (600 mg, 3.53 mmol) in ethylene glycol (4 mL) at room temperature was added *p*-toluenesulfonic acid monohydrate (30 mg, 0.18 mmol) and trimethoxymethane (1.54 mL, 14.1 mmol). The resulting mixture was allowed to stir for 12 h. It was quenched by sat. aqueous $NaHCO_3$ (5 mL), extracted with pentane (10 mL × 3), and finally purified by flash column chromatography (hexane/ethyl acetate = 15/1) to afford the pure substrate **4w** (220 mg) as a colorless oil in 30% yield. ^{1}H NMR (600 MHz, $CDCl_3$) δ 3.88 (br s, 4H), 2.80 – 2.77 (m, 1H), 2.58 (t, *J* = 12.0 Hz, 2H), 2.48 (t, *J* = 12.0 Hz, 2H), 1.44 (s, 9H); ^{13}C NMR (151 MHz, $CDCl_3$) δ 173.86, 105.61, 80.26, 64.29, 63.69, 39.21, 29.43, 27.96; HRMS (ESI): [M+Na]$^{\oplus}$ calcd for $C_{11}H_{18}O_4Na^{\oplus}$ 237.1097, found 237.1106.

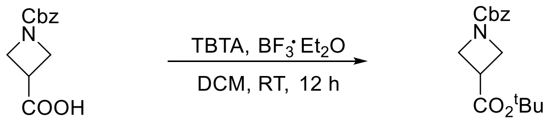

38 **7a**

Supplementary Figure 7. Synthesis of substrate **7a**

1-Benzyl 3-(*tert*-butyl) azetidine-1,3-dicarboxylate (**7a**): The procedure for the synthesis of **7a** is the same as that of **4r**. Purified by flash column chromatography (hexane/ethyl acetate = 7/1), yellow oil, 89% yield. ^{1}H NMR (400 MHz, Chloroform-*d*) δ 7.42 – 7.28 (m, 5H), 5.09 (s, 2H), 4.13 (d, *J* = 7.8 Hz, 4H), 3.32 – 3.24 (m, 1H), 1.46 (s, 9H); ^{13}C NMR (151 MHz, $CDCl_3$) δ 171.52, 156.34, 136.61, 128.55, 128.14, 128.05, 81.70, 66.80, 51.79, 33.54, 28.07; HRMS (ESI): [M+Na]$^{\oplus}$ calcd for $C_{16}H_{21}NO_4Na^{\oplus}$ 314.1362, found 314.1377.

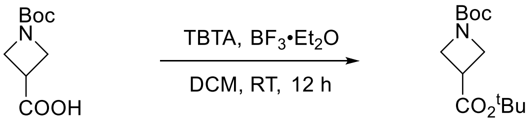

39 **7b**

Supplementary Figure 8. Synthesis of substrate **7b**

Di-*tert*-butyl azetidine-1,3-dicarboxylate (**7b**): The procedure for the synthesis of **7b** is the same as that of **4r**. Purified by flash column chromatography (hexane/ethyl acetate = 12/1), colorless oil, 84% yield. ^{1}H NMR (400 MHz, Chloroform-*d*) δ 4.04 (d, *J* = 7.3 Hz, 4H), 3.32 – 3.14 (m, 1H), 1.46 (s, 9H), 1.43 (s, 9H); ^{13}C NMR (151 MHz, $CDCl_3$) δ 171.80, 156.20, 81.48, 79.71, 51.66, 33.15, 28.43, 28.07; HRMS (ESI): [M+Na]$^{\oplus}$ calcd for $C_{13}H_{23}NO_4Na^{\oplus}$ 280.1519, found 280.1517.

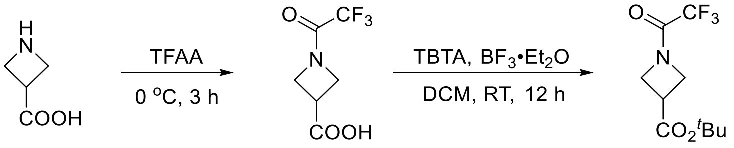

40 **41** **7c**

Supplementary Figure 9. Synthesis of substrate **7c**

***tert*-Butyl 1-(2,2,2-trifluoroacetyl)azetidine-3-carboxylate** (**7c**): To a 20 mL vial, **40** (0.80 g, 8.0 mmol) and TFAA (3.3 mL, 24 mmol) at room temperature were added. The mixture was stirred at 0 °C for 3 h. Then, water (20 mL) was added to quench the reaction and it was extracted with ethyl acetate for 3 times (20 mL × 3). The organic phase was collected and dried by anhydrous $MgSO_4$, filtered through celite and condensed to give crude yellow oil **41**. The second step was the same as described for the preparation of **4r**. Flash column chromatography (hexane/ethyl acetate = 10/1), colorless oil, 1.05 g, 52% yield. ^{1}H NMR (400 MHz, Chloroform-*d*) δ 4.51 (d, *J* = 7.5 Hz, 2H), 4.34 – 4.14 (m, 2H), 3.46 – 3.38 (m, 1H), 1.47 (s, 9H); ^{13}C NMR (151 MHz, $CDCl_3$) δ 170.44, 156.21 (t, *J* = 37.5 Hz), 116.05 (q, *J* = 288.0 Hz), 82.52 (d, *J* = 1.3 Hz), 54.19 (q, *J* = 2.4 Hz), 51.54, 33.85, 28.02 (d, *J* = 1.3 Hz); ^{19}F NMR (376 MHz, $CDCl_3$) δ -72.01. HRMS (ESI): [M+Na]$^{\oplus}$ calcd for $C_{10}H_{14}NF_3O_3Na^{\oplus}$ 276.0808, found 276.0820.

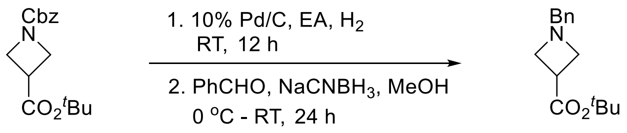

7a **7d**

Supplementary Figure 10. Synthesis of substrate **7d**

***tert*-Butyl 1-benzylazetidine-3-carboxylate** (**7d**): To a 25 mL flask, **7a** (0.50 g, 1.7 mmol), Pd/C (10 wt% on carbon, 0.18 g, 0.17 mmol) and ethyl acetate (6 ml) were added. The mixture was stirred under 1 atm hydrogen at RT for 12 h. The reaction solution was filtered through celite, washed by ethyl acetate and the organic phase was collected. Then acetic acid (0.10 mL, 1.7 mmol) was added to above solution and the resulting solution was condensed carefully to afford yellow oil used for next step. To a 100 mL flask with above crude compound, PhCHO (0.16 mL, 1.5 mmol) and methanol (8 mL) were added and stirred at 0 °C. $NaCNBH_3$ (0.12 g, 1.9 mmol) was added to above reaction in four portions. Then the mixture came back to RT and stirred for 12 h. After this time, the reaction solution was condensed under vacumn to remove most of methanol solvent. Sat. aqueous $NaHCO_3$ (20 mL) was added and the mixture was extracted with ethyl acetate (20 mL × 3), condensed and purified by flash column chromatography (hexane/ethyl acetate = 5/1) to afford the pure substrate **7d** (0.23 g) as a colorless oil in 54% yield. ^{1}H NMR (400 MHz, Chloroform-*d*) δ 7.35 – 7.21 (m, 5H), 3.62 (s, 2H), 3.59 – 3.47 (m, 2H), 3.32 – 3.20 (m, *J* = 4.3 Hz, 3H), 1.46 (s, 9H); ^{13}C NMR (151 MHz, $CDCl_3$) δ 172.57, 137.83, 128.59, 128.44, 127.18, 80.76, 63.53, 56.91, 35.10, 28.14; HRMS (ESI): $[M+H]^{\oplus}$ calcd for $C_{15}H_{22}NO_2^{\oplus}$ 248.1645, found 248.1650.

Reaction information for Supplementary Table 1

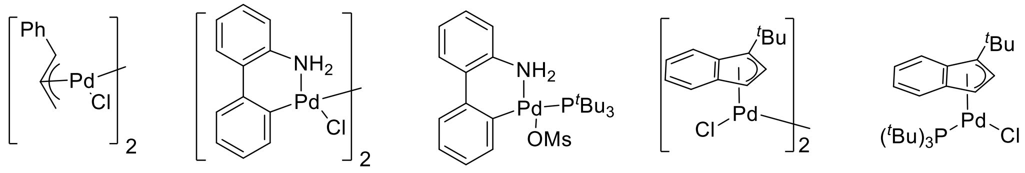

[Pd]-1 **[Pd]-2** **[Pd]-3** **[Pd]-4** **[Pd]-5**

Supplementary Figure 11. Pd complexes used in Supplementary Table 1

^{t}Bu$_3$P	^{t}Bu$_3$P·HBF4	Davephos	Xantphos	Qphos	Johnphos	Xphos	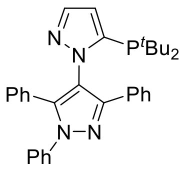
38%	22%	22%	18%	22%	13%	16%	
Sphos	PhDavephos	BINAP	PCy$_3$	Ruphos	PhP^{t}Bu$_2$	^{t}BuXphos	
14%	20%	10%	13%	17%	14%	8%	15%

Supplementary Figure 12. Ligands used for Entry 2 of Supplementary Table 1

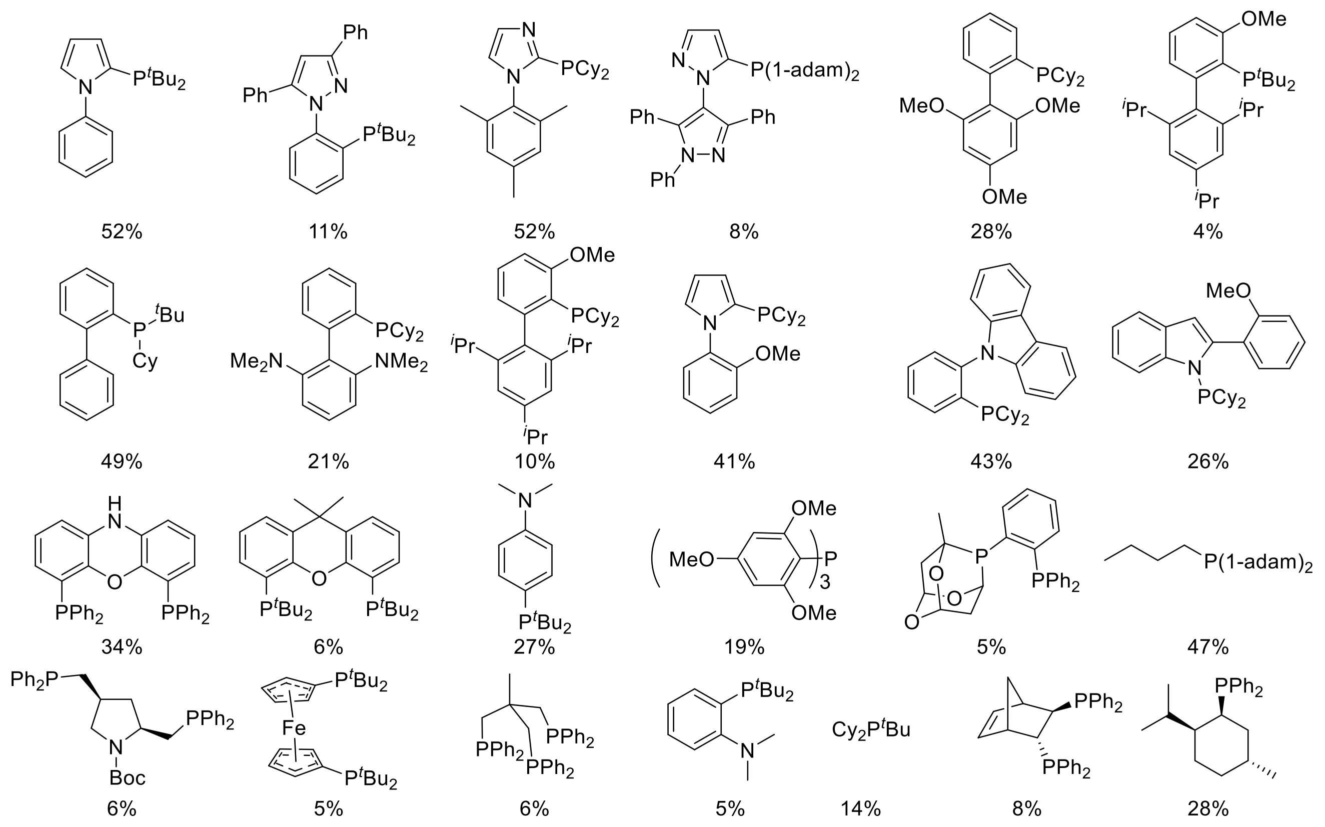

Supplementary Figure 13. Ligands used for Entry 12 of Supplementary Table 1

General procedures for Supplementary Table 2: In a dry and N_2-filled glovebox, to a stirred solution of $LiNCy_2$ in toluene (0.5 mL) in a 4 mL vial was added the small ring ester **2** dropwise at room temperature. The resulting mixture was allowed to stir for another 10 min at RT. Then, $Pd(dba)_2$, $^{t}Bu_3P$ (1M in toluene), and the aryl bromide **1a** (47 mg, 0.25 mmol) in toluene (0.10 mL) were added. This vial then was sealed with a PTFE lined cap, removed from the dry box and stirred outside at the stated temperature for 12 h. Fluorobenzene (23.5 uL, 0.250 mmol) was added to the above reaction solution as the internal standard for determining yields by ^{19}F NMR spectroscopy.

Methyl 1-(3-fluoro-4-methylphenyl)cyclobutane-1-carboxylate (6a)

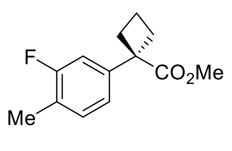

6a

Purified by flash column chromatography (hexane/ethyl acetate = 60/1), colorless oil. ^{1}H NMR (400 MHz, $CDCl_3$) δ 7.08 (m, 2H), 6.94 (t, J = 8.8 Hz, 1H), 3.65 (s, 3H), 2.84 – 2.78 (m, 2H), 2.50 – 2.43 (m, 2H), 2.26 (s, 3H), 2.06 – 1.99 (m, 1H), 1.89 –1.82 (m, 1H); ^{13}C NMR (151 MHz, $CDCl_3$) δ 176.48, 160.19 (d, $J_{C\text{-}F}$ = 243.1 Hz), 139.11 (d, $J_{C\text{-}F}$ = 3.0 Hz), 129.41 (d, $J_{C\text{-}F}$ = 6.0 Hz), 125.20 (d, $J_{C\text{-}F}$ = 7.6 Hz), 124.59 (d, $J_{C\text{-}F}$ = 16.6 Hz), 114.74 (d, $J_{C\text{-}F}$ = 22.7 Hz), 52.44, 51.83, 32.46, 16.58, 14.71; ^{19}F NMR (376 MHz) δ -119.83; HRMS (ESI): $[M+Na]^{\oplus}$ calcd for $C_{13}H_{15}O_2FNa^{\oplus}$ 245.0948, found 245.0947.

tert-Butyl 1-(3-fluoro-4-methylphenyl)cyclobutane-1-carboxylate (6b)

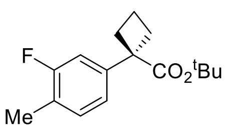

6b

Purified by flash column chromatography (hexane/ethyl acetate = 60/1), colorless oil. ^{1}H NMR (400 MHz, $CDCl_3$) δ 7.08 – 7.03 (m, 2H), 6.97 – 6.87 (m, 1H), 2.82 – 2.68 (m, 2H), 2.47 – 2.35 (m, 2H), 2.26 (d, J = 1.4 Hz, 3H), 2.06 – 1.92 (m, 1H), 1.90 – 1.75 (m, 1H), 1.36 (s, 9H); ^{13}C NMR (151 MHz, $CDCl_3$) δ 175.22, 160.03 (d, $J_{C\text{-}F}$ = 243.3 Hz), 139.79 (d, $J_{C\text{-}F}$ = 3.6 Hz), 129.30 (d, $J_{C\text{-}F}$ = 5.1 Hz,), 125.12 (d, $J_{C\text{-}F}$ = 8.0 Hz), 124.22 (d, $J_{C\text{-}F}$ = 17.4 Hz), 114.50 (d, $J_{C\text{-}F}$ = 22.3 Hz), 80.41, 52.73, 32.36, 27.86, 16.54, 14.68; ^{19}F NMR (376 MHz, $CDCl_3$) δ -120.49; HRMS (ESI): $[M+H]^{\oplus}$ calcd for $C_{16}H_{22}O_2F^{\oplus}$ 265.1598, found 265.1601.

Further evaluation of conditions for cyclobutyl esters

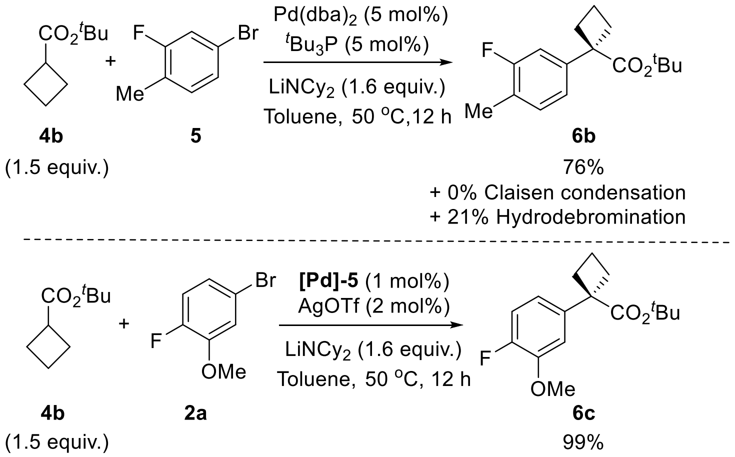

Supplementary Figure 14. Further evaluation of conditions for cyclobutyl esters

General procedures for Supplementary Table 3

In a dry and N_2-filled glovebox, to a 4 mL vial containing $Pd_2(dba)_3$ (7.3 mg, 0.0080 mol%) and ^{t}Bu$_3$P·HBF$_4$ (3.8 mg, 0.013 mmol%) was added LiHMDS (1M in toluene, 0.3 mL). The mixture was stirred at RT for 0.5 h. Then, arylbromide **2** (0.2 mmol) was added, followed by azetidine ester **7** (0.1 mmol) in toluene (0.3 mL) dropwise. The resulting mixture was allowed to stir at RT in the glovebox for 1 h. After this time, the reaction was quenched by addition of a citric acid solution (10% in water, 10 mL), and the resulting mixture was extracted with ethyl acetate (10 mL × 3) and condensed. The according yield was determined by crude ^{1}H NMR or ^{19}F NMR spectroscopy and the pure product was isolated by flash column chromatography.

1-Benzyl 3-(*tert*-butyl) 3-phenylazetidine-1,3-dicarboxylate (8x)

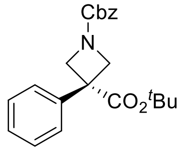

8x

It was purified by flash column chromatography (hexane/ethyl acetate = 12/1), yellow oil. ^{1}H NMR (400 MHz, Chloroform-*d*) δ 7.45 – 7.23 (m, 10H), 5.11 (s, 2H), 4.64 (d, J = 8.5 Hz, 2H), 4.34 (d, J = 8.7 Hz, 2H), 1.39 (s, 9H); ^{13}C NMR (101 MHz, $CDCl_3$) δ 172.15, 156.35, 140.12, 136.52, 128.70, 128.58, 128.19, 128.11, 127.58, 126.21, 82.19, 66.92, 48.50, 32.23, 27.83; HRMS (ESI): $[M+H]^{\oplus}$ calcd for $C_{22}H_{26}NO_4^{\oplus}$ 368.1856, found 368.1863.

1-Benzyl 3-(*tert*-butyl) 3-(4-fluoro-3-methoxyphenyl)azetidine-1,3-dicarboxylate (8y)

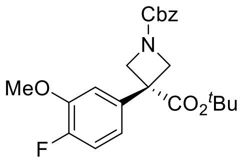

8y

It was purified by flash column chromatography (hexane/ethyl acetate = 5/1), yellow solid. ^{1}H NMR (400 MHz, Chloroform-*d*) δ 7.38 – 7.30 (m, 5H), 7.05 (dd, J = 11.0, 8.4 Hz, 1H), 6.87 – 6.71 (m, 2H), 5.10 (s, 2H), 4.61 (d, J = 8.7 Hz, 2H), 4.29 (d, J = 8.6 Hz, 2H), 3.87 (s, 3H), 1.39 (s, 9H); ^{13}C NMR (151 MHz, $CDCl_3$) δ 171.88, 156.37, 151.87 (d, $J_{C\text{-}F}$ = 247.0 Hz), 147.76 (d, $J_{C\text{-}F}$ = 11.0 Hz), 136.48, 136.39 (d, $J_{C\text{-}F}$ = 3.8 Hz), 128.60, 128.24, 128.15, 118.77 (d, $J_{C\text{-}F}$ = 7.0 Hz), 116.13 (d, $J_{C\text{-}F}$ = 18.7 Hz), 111.78, 82.39, 67.02, 58.54, 56.46, 48.31, 27.85; ^{19}F NMR (376 MHz, $CDCl_3$) δ -135.55; HRMS (ESI): $[M+H]^{\oplus}$ calcd for $C_{23}H_{27}NFO_5^{\oplus}$ 416.1868, found 416.1864.

di-*tert*-Butyl 3-(4-fluoro-3-methoxyphenyl)azetidine-1,3-dicarboxylate (10)

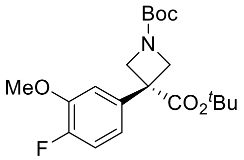

10

It was purified by flash column chromatography (hexane/ethyl acetate = 7/1), yellow oil. ^{1}H NMR (400 MHz, Chloroform-*d*) δ 7.04 (dd, J = 11.0, 8.4 Hz, 1H), 6.89 – 6.71 (m, 2H), 4.51 (d, J = 8.6 Hz, 2H), 4.20 (d, J = 7.3 Hz, 2H), 3.88 (s, 3H), 1.43 (s, 9H), 1.39 (s, 9H); ^{13}C NMR (151 MHz, $CDCl_3$) δ 172.10, 156.22, 151.78 (d, $J_{C\text{-}F}$ = 245.7 Hz), 147.69 (d, $J_{C\text{-}F}$ = 10.9 Hz), 136.68 (d, $J_{C\text{-}F}$ = 3.9 Hz), 118.80 (d, $J_{C\text{-}F}$ = 6.9 Hz), 116.05 (d, $J_{C\text{-}F}$ = 18.6 Hz), 111.81, 82.20, 80.04, 58.02, 56.43, 47.85, 28.42, 27.84; ^{19}F NMR (376 MHz, $CDCl_3$) δ -113.06 – -136.47 (m, 1F); HRMS (ESI): $[M+Na]^{\oplus}$ calcd for $C_{20}H_{28}NFNaO_5^{\oplus}$ 404.1844, found 404.1842.

General procedure for the α-arylation of cyclopropyl esters

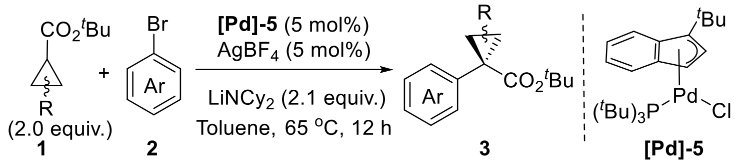

Supplementary Figure 15. General procedure for preparation of **3**

In a dry and N_2-filled glovebox, the small ring ester **1** (0.20 mmol) in toluene (0.3 mL) was added dropwise at room temperature to a 4 mL vial containing solid $LiNCy_2$ (39 mg, 0.21 mmol). The resulting mixture was allowed to stir for 15 min at RT. To a second vial containing **[Pd]-5** (2.6 mg, 0.0050 mmol) and $AgBF_4$ (1.0 mg, 0.0050 mmol) were added arylbromide **2** (0.10 mmol) and toluene (0.1 mL). The resulting mixture was shaken by hand for 30 seconds. Then, the solutions in these two vials above were

mixed together. The vial was sealed with a PTFE-lined cap, removed from the dry box, and stirred at 65 ^{o}C for 12 h. The reaction solution was condensed and purified by flash column chromatography to afford the pure product **3**.

Conditions for related isolation and spectral data

***tert*-Butyl 1-(4-fluoro-3-methoxyphenyl)cyclopropane-1-carboxylate (3a)**

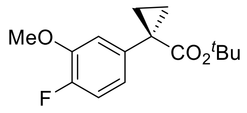

3a

Purified by flash column chromatography (hexane/ethyl acetate = 40/1), yellow oil, 27 mg, 99% yield. ^{1}H NMR (400 MHz, $CDCl_3$) δ 7.01 – 6.92 (m, 2H), 6.84 (ddd, *J* = 8.3, 4.4, 2.1 Hz, 1H), 3.89 (s, 3H), 1.51 (q, *J* = 3.9 Hz, 2H), 1.37 (s, 9H), 1.10 (q, *J* = 3.9 Hz, 2H; ^{13}C NMR (151 MHz, $CDCl_3$) δ 173.35, 151.52 (d, $J_{C\text{-}F}$ = 245.3 Hz), 146.94 (d, $J_{C\text{-}F}$ = 11.0 Hz), 136.54 (d, $J_{C\text{-}F}$ = 3.6 Hz), 122.71 (d, $J_{C\text{-}F}$ = 6.9 Hz), 116.01, 115.36 (d, $J_{C\text{-}F}$ = 18.3 Hz), 80.73, 56.31, 29.83, 28.01, 16.05; ^{19}F NMR (376 MHz, $CDCl_3$) δ -137.10; HRMS (ESI): [M+Na]$^{\oplus}$ calcd for $C_{15}H_{19}FO_3Na^{\oplus}$ 289.1210, found 289.1200.

***tert*-Butyl 1-phenylcyclopropane-1-carboxylate (3b)**

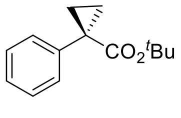

3b

Purified by flash column chromatography (hexane/ethyl acetate = 60/1), yellow oil, 20 mg, 92% yield. ^{1}H NMR (600 MHz, Chloroform-*d*) δ 7.33 (d, *J* = 7.2 Hz, 2H), 7.29 (t, *J* = 7.5 Hz, 2H), 7.23 (t, *J* = 7.2 Hz, 1H), 1.52 (q, *J* = 3.8 Hz, 2H), 1.37 (s, 9H), 1.13 (q, *J* = 3.9 Hz, 2H); ^{13}C NMR (151 MHz, $CDCl_3$) δ 173.68, 140.24, 130.48, 128.01, 126.87, 80.54, 30.07, 28.03, 15.84; HRMS (ESI): [M+Na]$^{\oplus}$ calcd for $C_{14}H_{18}O_2Na^{\oplus}$ 241.1199, found 241.1201.

***tert*-Butyl 1-(4-((*tert*-butyldimethylsilyl)oxy)phenyl)cyclopropane-1-carboxylate (3c)**

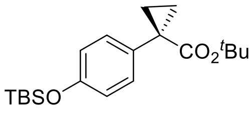

3c

Purified by flash column chromatography (hexane/ethyl acetate = 80/1), yellow oil, 28 mg, 81% yield. ^{1}H NMR (400 MHz, $CDCl_3$) δ 7.16 (d, *J* = 8.6 Hz, 2H), 6.74 (d, *J* = 8.6 Hz, 2H), 1.48 (q, *J* = 3.8 Hz, 2H), 1.35 (s, 9H), 1.07 (q, *J* = 3.8 Hz, 2H), 0.97 (s, 9H), 0.18 (s, 6H); ^{13}C NMR (151 MHz, $CDCl_3$) δ 174.02, 154.44, 133.04, 131.40, 119.50, 80.34, 29.31, 28.02, 25.77, 18.28, 16.01, -4.34; HRMS (ESI): [M+Na]$^{\oplus}$ calcd for $C_{20}H_{33}SiO_3Na^{\oplus}$ 371.2012, found 371.2012.

***tert*-Butyl 1-(4-morpholinophenyl)cyclopropane-1-carboxylate (3d)**

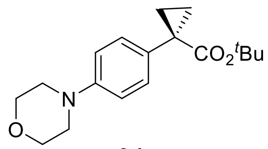

3d

Purified by flash column chromatography (hexane/ethyl acetate = 8/1), yellow oil, 25 mg, 82% yield. ^{1}H NMR (400 MHz, $CDCl_3$) δ 7.24 (d, *J* = 8.7 Hz, 2H), 6.84 (d, *J* = 8.7 Hz, 2H), 3.92 – 3.77 (m, 4H), 3.20 – 3.07 (m, 4H), 1.49 – 1.46 (m, 2H), 1.37 (s, 9H), 1.09 – 1.06 (m, 2H); ^{13}C NMR (151 MHz, $CDCl_3$) δ 174.00, 150.09, 131.68, 131.18, 115.27, 80.37, 67.01, 49.48, 29.20, 28.05, 15.87; HRMS (ESI): [M+Na]$^{\oplus}$ calcd for $C_{18}H_{25}NO_3Na^{\oplus}$ 326.1726, found 326.1722.

tert-Butyl 1-(4-ethoxyphenyl)cyclopropane-1-carboxylate (3e)

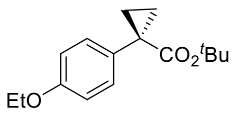

3e

Purified by flash column chromatography (hexane/ethyl acetate = 60/1), colorless oil, 22 mg, 84% yield. ^{1}H NMR (600 MHz, $CDCl_3$) δ 7.23 (d, *J* = 8.5 Hz, 2H), 6.81 (d, *J* = 8.5 Hz, 2H), 4.01 (q, *J* = 6.9 Hz, 2H), 1.48 (dd, *J* = 6.3, 3.6 Hz, 2H), 1.40 (t, *J* = 7.0 Hz, 3H), 1.37 (s, 9H), 1.08 (dd, *J* = 6.3, 3.5 Hz, 2H); ^{13}C NMR (151 MHz, $CDCl_3$) δ 173.98, 157.82, 132.28, 131.47, 113.97, 80.39, 63.45, 29.26, 28.05, 15.96, 14.96; HRMS (ESI): $[M+Na]^{\oplus}$ calcd for $C_{16}H_{22}O_3Na^{\oplus}$ 285.1461, found 285.1457.

tert-Butyl 1-(4-(diethylcarbamoyl)phenyl)cyclopropane-1-carboxylate (3f)

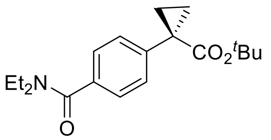

3f

Purified by flash column chromatography (hexane/ethyl acetate = 4/1), colorless oil, 26 mg, 81% yield. ^{1}H NMR (600 MHz, Chloroform-*d*) δ 7.34 (d, *J* = 8.0 Hz, 2H), 7.29 (d, *J* = 8.0 Hz, 2H), 3.39 (d, *J* = 164.5 Hz, 4H), 1.57 – 1.48 (m, 2H), 1.36 (s, 9H), 1.23 (br s, 3H), 1.16 – 0.99 (m, 5H); ^{13}C NMR (151 MHz, $CDCl_3$) δ 173.32, 171.30, 141.27, 135.75, 130.44, 126.07, 80.78, 43.31, 39.29, 29.84, 27.99, 15.95, 14.30, 12.97; HRMS (ESI): $[M+Na]^{\oplus}$ calcd for $C_{19}H_{27}NO_3Na^{\oplus}$ 340.1883, found 340.1879.

tert-Butyl 1-(4-chlorophenyl)cyclopropane-1-carboxylate (3g)

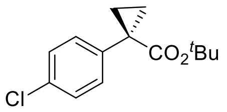

3g

Purified by flash column chromatography (hexane/ethyl acetate =60/1), yellow oil, 23 mg, 90% yield. ^{1}H NMR (400 MHz, Chloroform-*d*) δ 7.26 (br s, 4H), 1.53 (q, *J* = 3.9 Hz, 2H), 1.37 (s, 9H), 1.09 (q, *J* = 3.9 Hz, 2H); ^{13}C NMR (101 MHz, $CDCl_3$) δ 173.21, 138.77, 132.67, 131.85, 128.17, 80.86, 29.46, 28.01, 15.96; HRMS (ESI): $[M+Na]^{\oplus}$ calcd for $C_{14}H_{17}ClO_2Na^{\oplus}$ 275.0809, found 275.0817.

tert-Butyl 1-(3-(methylthio)phenyl)cyclopropane-1-carboxylate (3h)

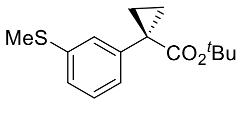

3h

Purified by flash column chromatography (hexane/ethyl acetate = 60/1), colorless oil, 21 mg, 81% yield. ^{1}H NMR (400 MHz, $CDCl_3$) δ 7.24 – 7.18 (m, 2H), 7.12 (t, *J* = 7.1 Hz, 2H), 2.48 (s, 3H), 1.51 (q, *J* = 3.9 Hz, 2H), 1.37 (s, 9H), 1.11 (q, *J* = 3.9 Hz, 2H); ^{13}C NMR (151 MHz, $CDCl_3$) δ 173.32, 140.85, 137.83, 128.83, 128.43, 127.48, 125.20, 80.66, 29.99, 28.01, 16.02, 15.84; HRMS (ESI): $[M+Na]^{\oplus}$ calcd for $C_{15}H_{20}SO_2Na^{\oplus}$ 287.1076, found 287.1082.

tert-Butyl 1-(3-(1,3-dioxolan-2-yl)phenyl)cyclopropane-1-carboxylate (3i)

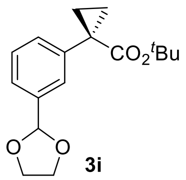

3i

Purified by flash column chromatography (hexane/ethyl acetate = 10/1), colorless oil, 23 mg, 78% yield. ^{1}H NMR (600 MHz, Chloroform-*d*) δ 7.42 (s, 1H), 7.39 – 7.28 (m, 3H), 5.80 (s, 1H), 4.15 – 4.09 (m, 2H), 4.09 – 3.96 (m, 2H), 1.52 – 1.50 (m, 2H), 1.36 (s, 9H), 1.14 – 1.12 (m, 2H); ^{13}C NMR (151 MHz, $CDCl_3$) δ 173.44, 140.29, 137.54, 131.53, 128.34, 128.03, 124.97, 103.77, 80.61, 77.31, 77.10, 76.89, 65.31, 29.94, 28.01, 15.92; HRMS (ESI): $[M+Na]^{\oplus}$ calcd for $C_{17}H_{22}O_4Na^{\oplus}$ 313.1410, found 313.1407.

tert-Butyl 1-(2-fluoro-4-methoxyphenyl)cyclopropane-1-carboxylate (3j)

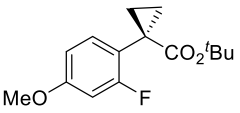

3j

Purified by flash column chromatography (hexane/ethyl acetate = 40/1), yellow oil, 23 mg, 70% yield. ^{1}H NMR (600 MHz, Chloroform-*d*) δ 7.12 (t, *J* = 8.6 Hz, 1H), 6.60 (t, *J* = 8.2 Hz, 2H), 3.78 (s, 3H), 1.56 – 1.49 (m, 2H), 1.36 (s, 9H), 1.11 – 1.04 (m, 2H); ^{13}C NMR (151 MHz, $CDCl_3$) δ 173.09, 163.06 (d, J_{C-F} = 247.7 Hz), 160.07 (d, J_{C-F} = 11.1 Hz), 131.50 (d, J_{C-F} = 5.8 Hz), 119.83 (d, J_{C-F} = 15.0 Hz), 109.04 (d, J_{C-F} = 2.9 Hz), 101.55 (d, J_{C-F} = 25.4 Hz), 80.64, 55.60, 27.98, 24.32, 15.81; HRMS (ESI): [M+Na]$^{\oplus}$ calcd for $C_{15}H_{19}FO_3Na^{\oplus}$ 289.1210, found 289.1216.

tert-Butyl 1-(3-adamantan-1-yl)-4-methoxyphenyl)cyclopropane-1-carboxylate (3k)

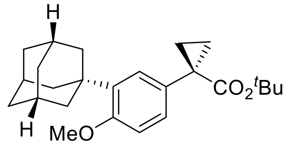

3k

Purified by flash column chromatography (hexane/ethyl acetate = 60/1), yellow oil, 36 mg, 94% yield. ^{1}H NMR (400 MHz, Chloroform-*d*) δ 7.19 (d, *J* = 2.3 Hz, 1H), 7.10 (dd, *J* = 8.3, 2.3 Hz, 1H), 6.78 (d, *J* = 8.4 Hz, 1H), 3.81 (s, 3H), 2.09 – 2.05 (m, 9H), 1.77 (s, 6H), 1.47 (q, *J* = 3.8 Hz, 2H), 1.38 (s, 9H), 1.10 (q, *J* = 3.8 Hz, 2H); ^{13}C NMR (101 MHz, $CDCl_3$) δ 174.09, 157.70, 137.81, 131.80, 129.18, 128.23, 111.02, 80.23, 55.06, 40.70, 37.24, 37.00, 29.67, 29.21, 28.08, 15.93; HRMS (ESI): [M+Na]$^{\oplus}$ calcd for $C_{25}H_{34}O_3Na^{\oplus}$ 405.2400, found 405.2405.

tert-Butyl 1-(4-((((1*R*,2*S*)-1,7,7-trimethylbicyclo[2.2.1]heptan-2-yl)oxy)methyl)phenyl)cyclo-propane-1-carboxylate (3l)

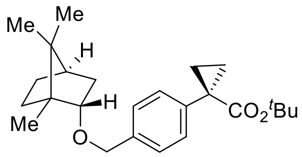

3l

Purified by flash column chromatography (hexane/ethyl acetate = 60/1), yellow oil, 35 mg, 91% yield. ^{1}H NMR (600 MHz, Chloroform-*d*) δ 7.30 -7.26 (m, 4H), 4.56 (d, *J* = 12.2 Hz, 1H), 4.43 (d, *J* = 12.2 Hz, 1H), 3.69 (d, *J* = 8.8 Hz, 1H), 2.14 – 2.07 (m, 2H), 1.76 – 1.67 (m, 1H), 1.67 – 1.62 (m, 1H), 1.56 – 1.49 (m, 2H), 1.38 (s, 9H), 1.26 – 1.23 (m, 2H), 1.11 – 1.08 (m, 3H), 0.90 (s, 3H), 0.86 (s, 3H), 0.83 (s, 3H); ^{13}C NMR (151 MHz, $CDCl_3$) δ 173.70, 139.09, 138.16, 130.30, 126.88, 84.32, 80.49, 71.42, 49.36, 47.92, 45.13, 36.20, 29.77, 28.35, 28.03, 26.85, 19.87, 18.95, 15.88, 14.10; HRMS (ESI): [M+Na]$^{\oplus}$ calcd for $C_{25}H_{36}O_3Na^{\oplus}$ 407.2556, found 407.2556.

tert-Butyl 1-(thiophen-3-yl)cyclopropane-1-carboxylate (3m)

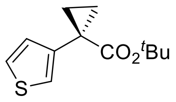

3m

Purified by flash column chromatography (hexane/ethyl acetate = 60/1), yellow oil, 18 mg, 79% yield. ^{1}H NMR (400 MHz, $CDCl_3$) δ 7.22 (dd, *J* = 4.9, 3.0 Hz, 1H), 7.13 – 7.02 (m, 2H), 1.52 – 1.49 (m, 2H), 1.41 (s, 9H), 1.13 – 1.11 (m, 2H); ^{13}C NMR (151 MHz, $CDCl_3$) δ 173.05, 140.95, 129.43, 124.76, 122.39, 80.72, 28.08, 24.96, 16.90; HRMS (ESI): [M+Na]$^{\oplus}$ calcd for $C_{12}H_{16}SO_2Na^{\oplus}$ 247.0763, found 247.0752.

tert-Butyl 1-(1-methyl-1H-indol-5-yl)cyclopropane-1-carboxylate (**3n**)

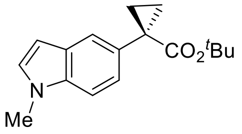

3n

Purified by flash column chromatography (hexane/ethyl acetate = 60/1), yellow oil, 22 mg, 80% yield. ^{1}H NMR (400 MHz, $CDCl_3$) δ 7.61 (s, 1H), 7.30 (s, 2H), 7.07 (d, *J* = 2.9 Hz, 1H), 6.48 (d, *J* = 2.9 Hz, 1H), 3.82 (s, 3H), 1.59 (dd, *J* = 6.4, 3.6 Hz, 2H), 1.42 (s, 9H), 1.23 (dd, *J* = 6.5, 3.7 Hz, 2H); ^{13}C NMR (151 MHz, $CDCl_3$) δ 174.47, 135.84, 131.25, 128.98, 128.18, 124.87, 122.24, 108.69, 100.94, 80.19, 32.92, 30.18, 28.08, 16.16; HRMS (ESI): $[M+Na]^{\oplus}$ calcd for $C_{17}H_{21}O_2Na^{\oplus}$ 294.1464, found 294.1467.

tert-Butyl 1-(2,6-dimethoxypyridin-3-yl)cyclopropane-1-carboxylate (**3o**)

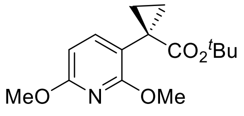

3o

Purified by flash column chromatography (hexane/ethyl acetate = 60/1), yellow oil, 20 mg, 71% yield. ^{1}H NMR (400 MHz, $CDCl_3$) δ 7.34 (d, *J* = 7.9 Hz, 1H), 6.20 (d, *J* = 7.9 Hz, 1H), 3.94 (s, 3H), 3.90 (s, 3H), 1.50 – 1.47 (m, 2H), 1.34 (s, 9H), 0.98 – 0.96 (m, 2H); ^{13}C NMR (151 MHz, $CDCl_3$) δ 173.53, 162.03, 161.89, 141.16, 114.30, 99.44, 80.16, 53.61, 53.30, 28.00, 24.71, 15.90; HRMS (ESI): $[M+H]^{\oplus}$ calcd for $C_{15}H_{22}NO_4^{\oplus}$ 280.1543, found 280.1554.

tert-Butyl 1-(benzo[b]thiophen-5-yl)cyclopropane-1-carboxylate (**3p**)

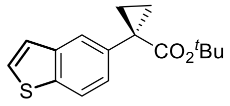

3p

Purified by flash column chromatography (hexane/ethyl acetate = 60/1), yellow oil, 20 mg, 72% yield. ^{1}H NMR (600 MHz, Chloroform-*d*) δ 7.89 – 7.71 (m, 2H), 7.42 (d, *J* = 5.4 Hz, 1H), 7.36 (dd, *J* = 8.3, 1.5 Hz, 1H), 7.29 (d, *J* = 5.4 Hz, 1H), 1.58 (q, *J* = 3.8 Hz, 2H), 1.38 (s, 9H), 1.19 (q, *J* = 3.8 Hz, 2H); ^{13}C NMR (151 MHz, $CDCl_3$) δ 173.79, 139.56, 138.40, 136.51, 127.53, 126.56, 124.99, 123.88, 121.94, 80.65, 30.04, 28.05, 16.08; HRMS (ESI): $[M+Na]^{\oplus}$ calcd for $C_{16}H_{18}O_2SNa^{\oplus}$ 297.0919, found 297.0920.

tert-Butyl 1-(1-(triisopropylsilyl)-1H-pyrrol-3-yl)cyclopropane-1-carboxylate (**3q**)

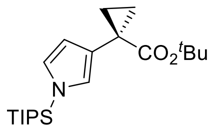

3q

Purified by flash column chromatography (hexane/ethyl acetate = 80/1), yellow oil, 27 mg, 75% yield. ^{1}H NMR (400 MHz, $CDCl_3$) δ 6.79 – 6.72 (m, 1H), 6.64 (t, *J* = 2.4 Hz, 1H), 6.15 (dd, *J* = 2.6, 1.4 Hz, 1H), 1.47 – 1.38 (m, 14H), 1.11 – 1.04 (m, 20H); ^{13}C NMR (151 MHz, $CDCl_3$) δ 174.07, 124.99, 123.42, 122.88, 110.68, 80.01, 28.16, 22.67, 18.20, 17.95, 11.76; HRMS (ESI): $[M+Na]^{\oplus}$ calcd for $C_{21}H_{37}NSiO_2Na^{\oplus}$ 386.2485, found 386.2479.

tert- Butyl 1-(4-fluoro-3-methoxyphenyl)-2-(p-tolyl)cyclopropane-1-carboxylate (**3r**)

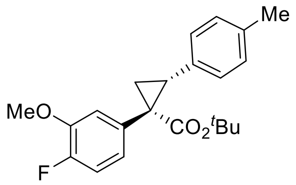

3r

Purified by flash column chromatography (hexane/ethyl acetate = 40/1), light yellow oil, 30 mg, dr 12:1, 85% yield. For major isomer: ^{1}H NMR (400 MHz, $CDCl_3$) δ 7.26 (d, *J* = 8.1 Hz, 2H), 7.18 – 7.09 (m, 3H), 7.04 (d, *J* = 8.2 Hz, 2H), 3.94 (s, 3H), 2.73 (t, *J* = 8.1 Hz, 1H), 2.34 (s, 3H), 2.24 (dd, *J* = 7.1, 5.2 Hz, 1H), 1.53 (dd, *J* = 9.0, 5.0 Hz, 1H), 1.04 (s, 9H); ^{13}C NMR (101 MHz, $CDCl_3$) δ 169.57, 151.65 (d, $J_{C\text{-}F}$ = 245.2 Hz), 147.09 (d, $J_{C\text{-}F}$ = 10.8 Hz), 137.39 (d, $J_{C\text{-}F}$ = 3.6 Hz), 136.40, 133.45, 129.25, 128.73, 122.59 (d, $J_{C\text{-}F}$ = 6.8 Hz), 115.72, 115.54 (d, $J_{C\text{-}F}$ = 18.4 Hz), 80.80, 56.38, 38.38, 32.75, 27.56,

21.13, 17.33; ^{19}F NMR (376 MHz, $CDCl_3$) δ -136.65 – -136.71 (m, 1F).; HRMS (ESI): $[M+Na]^{\oplus}$ calcd for $C_{22}H_{25}FO_3Na^{\oplus}$ 379.1679, found 379.1690.

***tert*-Butyl-1-(4-fluoro-3-methoxyphenyl)-2,2-dimethyl-3-(2-methylprop-1-en-1-yl)cyclopropane-1-carboxylate** (**3s**)

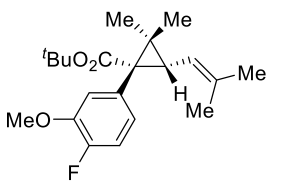

3s (major isomer)

Purified by flash column chromatography (hexane/ethyl acetate = 60/1), yellow oil, 26 mg, 64% yield, dr = 2:1. Major isomer: ^{1}H NMR (400 MHz, $CDCl_3$) δ 7.02 – 6.93 (m, 2H), 6.89 (ddd, *J* = 8.3, 4.5, 2.1 Hz, 1H), 5.37 – 5.27 (m, 1H), 3.88 (s, 3H), 1.90 (d, *J* = 8.2 Hz, 1H), 1.80 (d, *J* = 11.9 Hz, 6H), 1.34 (s, 9H), 1.24 (s, 3H), 0.88 (s, 3H); ^{13}C NMR (151 MHz, $CDCl_3$) δ 169.69, 151.53 (d, $J_{C\text{-}F}$ = 244.2 Hz), 146.92 (d, $J_{C\text{-}F}$ = 10.5 Hz), 136.21 (d, $J_{C\text{-}F}$ = 3.8 Hz), 134.41, 122.86 (d, $J_{C\text{-}F}$ = 6.8 Hz), 119.87, 115.81, 115.32 (d, $J_{C\text{-}F}$ = 18.3 Hz), 80.52, 56.30, 45.23, 34.16, 28.06, 27.76, 26.05, 25.21, 18.69, 18.59; ^{19}F NMR (376 MHz, $CDCl_3$) δ -137.39 (major isomer), -137.75 (minor isomer); HRMS (ESI): $[M+Na]^{\oplus}$ calcd for $C_{21}H_{29}FO_3Na^{\oplus}$ 371.1992, found 371.1993.

General procedure for α-arylation of cyclobutyl esters

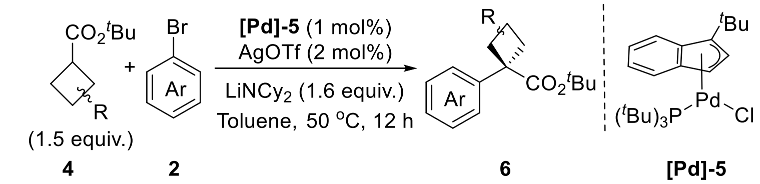

Supplementary Figure 16. General procedure for the preparation of **6**

In a dry and N_2-filled glovebox, the small ring ester **4** (0.15 mmol) in toluene (0.3 mL) was added dropwise at room temperature to a 4 mL vial containing solid $LiNCy_2$ (30 mg, 0.16 mmol). The resulting mixture was allowed to stir for 15 min at RT. To a second vial containing **[Pd]-5** (0.5 mg, 0.001 mmol) and AgOTf (0.6 mg, 0.001 mmol), were added arylbromide **2** (0.10 mmol) and toluene (0.1 mL). The resulting mixture was shaken by hand for 30 seconds. Then, the solutions in these two vials above were mixed together. The vial was sealed with a PTFE-lined cap, removed from the dry box, and stirred at 50 °C for 12 h. The reaction solution was condensed and purified by flash column chromatography to afford the pure product **6**.

Conditions for related isolation and spectral data

***tert*-Butyl 1-(4-fluoro-3-methoxyphenyl)cyclobutane-1-carboxylate** (**6c**)

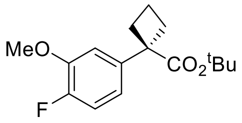

6c

Purified by flash column chromatography (hexane/ethyl acetate = 60/1), colorless oil, 28 mg, quant. yield. ^{1}H NMR (400 MHz, $CDCl_3$) δ 7.00 (dd, *J* = 11.2, 8.4 Hz, 1H), 6.86 (dd, *J* = 8.2, 2.2 Hz, 1H), 6.80 (ddd, *J* = 8.3, 4.3, 2.2 Hz, 1H), 3.88 (s, 3H), 2.81 – 2.70 (m, 2H), 2.47 – 2.34 (m, 2H), 2.06 – 1.95 (m, 1H), 1.91 – 1.76 (m, 1H), 1.36 (s, 9H); ^{13}C NMR (151 MHz, $CDCl_3$) δ 174.94, 151.13 (d, $J_{C\text{-}F}$ = 244.5 Hz), 147.15 (d, $J_{C\text{-}F}$ = 10.5 Hz), 140.69 (d, $J_{C\text{-}F}$ = 3.6 Hz), 118.57 (d, $J_{C\text{-}F}$ = 6.8 Hz), 115.41 (d, $J_{C\text{-}F}$ = 18.3 Hz).111.67, 80.51, 56.26,

53.07, 32.30, 27.83, 16.44; ^{19}F NMR (376 MHz, $CDCl_3$) δ -138.06; HRMS (ESI): $[M+H]^{\oplus}$ calcd for $C_{16}H_{22}O_3F^{\oplus}$ 281.1547, found 281.1543.

***tert*-Butyl 1-(4-(*N*,*N*-dimethylsulfamoyl)phenyl)cyclobutane-1-carboxylate (6d)**

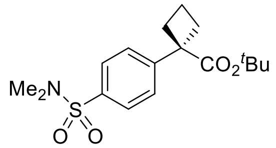

6d

Purified by flash column chromatography (hexane/ethyl acetate = 8/1), light yellow oil, 33 mg, 97% yield. ^{1}H NMR (400 MHz, $CDCl_3$) δ 7.71 (d, J = 8.3 Hz, 2H), 7.42 (d, J = 8.3 Hz, 2H), 2.86 – 2.76 (m, 2H), 2.70 (s, 6H), 2.53 – 2.40 (m, 2H), 2.16 – 2.02 (m, 1H), 1.92 – 1.83 (m, 1H), 1.35 (s, 9H); ^{13}C NMR (151 MHz, $CDCl_3$) δ 173.98, 149.55, 133.26, 127.59, 126.86, 80.95, 53.28, 37.93, 32.22, 27.71, 16.63; HRMS (ESI): $[M+Na]^{\oplus}$ calcd for $C_{17}H_{25}O_4SNNa^{\oplus}$ 362.1396, found 362.1399.

***tert*-Butyl 1-(4-(diethylcarbamoyl)phenyl)cyclobutane-1-carboxylate (6e)**

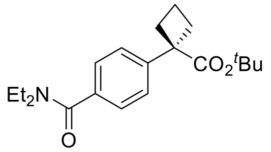

6e

Purified by flash column chromatography (hexane/ethyl acetate = 3/1), yellow oil, 27 mg, 81% yield. ^{1}H NMR (400 MHz, $CDCl_3$) δ 7.39 – 7.24 (m, 4H), 3.53 – 3.26 (m, 4H), 2.88 – 2.69 (m, 2H), 2.47 – 2.40 (m, 2H), 2.13 – 1.95 (m, 1H), 1.91 – 1.81 (m, 1H), 1.35 (s, 9H), 1.24 – 1.11 (m, 6H); ^{13}C NMR (151 MHz, $CDCl_3$) δ 174.83, 171.34, 145.49, 135.22, 126.29, 126.28, 80.58, 53.27, 43.36, 39.34, 32.33, 27.84, 16.66, 14.30, 12.98; HRMS (ESI): $[M+Na]^{\oplus}$ calcd for $C_{20}H_{29}O_3NNa^{\oplus}$ 354.2039, found 354.2034.

***tert*-Butyl 1-(6-methoxynaphthalen-2-yl)cyclobutane-1-carboxylate (6f)**

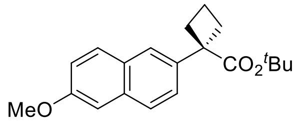

6f

Purified by flash column chromatography (hexane/ethyl acetate = 50/1), white solid, 31 mg, quant. yield. ^{1}H NMR (400 MHz, Chloroform-*d*) δ 7.73 – 7.67 (m, 3H), 7.40 (d, J = 8.4 Hz, 1H), 7.22 – 7.01 (m, 2H), 3.92 (s, 3H), 2.97 – 2.76 (m, 2H), 2.66 – 2.46 (m, 2H), 2.12 – 2.00 (m, 1H), 1.94 – 1.84 (m, 1H), 1.36 (s, 9H); ^{13}C NMR (151 MHz, $CDCl_3$) δ 175.38, 157.58, 139.44, 133.21, 129.43, 128.70, 126.69, 125.50, 124.41, 118.79, 105.62, 80.38, 55.36, 53.26, 32.28, 27.89, 16.66; HRMS (ESI): $[M+Na]^{\oplus}$ calcd for $C_{20}H_{24}O_3Na^{\oplus}$ 335.1617, found 335.1618.

***tert*-Butyl 1-(4-morpholinophenyl)cyclobutane-1-carboxylate (6g)**

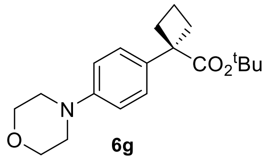

6g

Purified by flash column chromatography (hexane/ethyl acetate = 10/1), yellow oil, 26 mg, 81% yield. ^{1}H NMR (600 MHz, Chloroform-*d*) δ 7.20 (d, J = 8.7 Hz, 2H), 6.87 (d, J = 8.6 Hz, 2H), 3.97 – 3.77 (m, 4H), 3.23 – 3.04 (m, 4H), 2.76 – 2.72 (m, 2H), 2.48 – 2.28 (m, 2H), 2.04 – 1.91 (m, 1H), 1.86 – 1.79 (m, 1H), 1.36 (s, 9H); ^{13}C NMR (151 MHz, $CDCl_3$) δ 175.57, 149.62, 135.77, 127.07, 115.36, 80.17, 67.02, 52.57, 49.48, 32.22, 27.90, 16.52; HRMS (ESI): $[M+H]^{\oplus}$ calcd for $C_{19}H_{28}NO_3^{\oplus}$ 318.2064, found 318.2055.

tert-Butyl 1-(3-chlorophenyl)cyclobutane-1-carboxylate (6h)

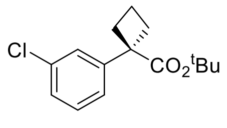

6h

Purified by flash column chromatography (hexane/ethyl acetate = 80/1), yellow oil, 27 mg, quant. yield. ^{1}H NMR (600 MHz, Chloroform-*d*) δ 7.30 – 7.12 (m, 4H), 2.84 – 2.67 (m, 2H), 2.49 – 2.35 (m, 2H), 2.10 – 1.96 (m, 1H), 1.91 – 1.79 (m, 1H), 1.37 (s, 9H); ^{13}C NMR (151 MHz, $CDCl_3$) δ 174.57, 146.46, 134.01, 129.39, 126.63, 126.57, 124.52, 80.76, 53.13, 32.27, 27.86, 16.65; HRMS (ESI): [M+Na]$^{\oplus}$ calcd for $C_{15}H_{19}O_2NaCl^{\oplus}$ 289.0965, found 289.0959.

tert-Butyl 1-(3-(1,3-dioxolan-2-yl)phenyl)cyclobutane-1-carboxylate (6i)

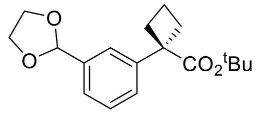

6i

Purified by flash column chromatography (hexane/ethyl acetate = 15/1), yellow oil, 30 mg, quant. yield. ^{1}H NMR (600 MHz, Chloroform-*d*) δ 7.44 – 7.27 (m, 4H), 5.80 (s, 1H), 4.17 – 3.97 (m, 4H), 2.85 – 2.70 (m, 2H), 2.53 – 2.38 (m, 2H), 2.07 – 1.95 (m, 1H), 1.92 – 1.76 (m, 1H), 1.35 (s, 9H); ^{13}C NMR (151 MHz, $CDCl_3$) δ 175.05, 144.51, 137.71, 128.21, 127.21, 124.47, 124.45, 103.90, 80.43, 65.35, 53.31, 32.32, 27.87, 16.65; HRMS (ESI): [M+Na]$^{\oplus}$ calcd for $C_{18}H_{24}O_4Na^{\oplus}$ 327.1566, found 327.1561.

tert-Butyl 1-(3-(methylthio)phenyl)cyclobutane-1-carboxylate (6j)

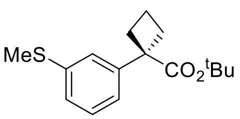

6j

Purified by flash column chromatography (hexane/ethyl acetate = 60/1), yellow oil, 26 mg, 93% yield. ^{1}H NMR (400 MHz, Chloroform-*d*) δ 7.35 – 7.05 (m, 4H), 2.91 – 2.71 (m, 2H), 2.59 – 2.35 (m, 5H), 2.13 – 1.97 (m, 1H), 1.97 – 1.79 (m, 1H), 1.41 (s, 9H); ^{13}C NMR (151 MHz, $CDCl_3$) δ 174.94, 145.07, 138.09, 128.57, 124.66, 124.63, 123.19, 80.49, 53.30, 32.28, 27.88, 16.65, 16.00; HRMS (ESI): [M+Na]$^{\oplus}$ calcd for $C_{18}H_{24}O_4Na^{\oplus}$ 327.1566, found 327.1561.

tert-Butyl 1-(*o*-tolyl)cyclobutane-1-carboxylate (6k)

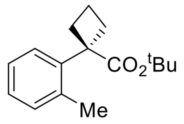

6k

Purified by flash column chromatography (hexane/ethyl acetate = 40/1), colorless oil, 23 mg, 93% yield. ^{1}H NMR (400 MHz, Chloroform-*d*) δ 7.22 – 6.91 (m, 4H), 2.84 – 2.63 (m, 2H), 2.51 (qd, *J* = 9.4, 2.4 Hz, 2H), 2.27 – 2.07 (m, 4H), 1.90 – 1.72 (m, 1H), 1.37 (s, 9H). ^{13}C NMR (151 MHz, $CDCl_3$) δ 175.15, 142.61, 135.98, 130.80, 127.03, 126.50, 125.57, 80.30, 54.02, 32.58, 27.86, 19.88, 17.15. HRMS (ESI): [M+H]$^{\oplus}$ calcd for $C_{16}H_{23}O_2^{\oplus}$ 247.1693, found 247.1690.

tert-Butyl 1-(5-fluoro-2-methoxyphenyl)cyclobutane-1-carboxylate (6l)

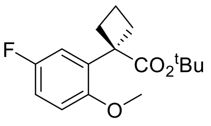

6l

Purified by flash column chromatography (hexane/ethyl acetate = 20/1), colorless oil, 18 mg, 65% yield. ^{1}H NMR (400 MHz, Chloroform-*d*) δ 6.98 (dd, *J* = 9.5, 3.1 Hz, 1H), 6.88 (td, *J* = 8.4, 3.1 Hz, 1H), 6.71 (dd, *J* = 8.8, 4.5 Hz, 1H), 3.72 (s, 3H), 2.77 – 2.59 (m, 2H), 2.41 – 2.22 (m, 2H), 2.20 – 2.03 (m, 1H), 1.92 – 1.76 (m, 1H), 1.36 (s, 9H). ^{13}C NMR (151 MHz, $CDCl_3$) δ 174.85, 156.97 (d, *J* = 236.9 Hz), 153.26, 134.84 (d, *J* = 7.0 Hz), 114.10 (d, *J* = 24.1 Hz), 113.03 (d, *J* = 22.9 Hz), 110.93 (d, *J* = 8.6 Hz), 79.85, 55.64, 50.99, 31.00, 27.87,

16.79. ^{19}F NMR (376 MHz, Chloroform-*d*) δ -123.81. HRMS (ESI): [M+H]$^{\oplus}$ calcd for $C_{16}H_{22}FO_3^{\oplus}$ 281.1547, found 281.1540.

tert-Butyl 1-(2-(trifluoromethyl)phenyl)cyclobutane-1-carboxylate (6m)

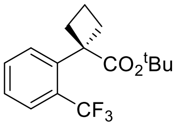

6m

Purified by flash column chromatography (hexane/ethyl acetate = 40/1), yellow oil, 15 mg, 50% yield. ^{1}H NMR (400 MHz, Chloroform-*d*) δ 7.61 (d, *J* = 7.8 Hz, 1H), 7.51 (t, *J* = 7.6 Hz, 1H), 7.35 – 7.31 (m, 2H), 2.76 (t, *J* = 9.4 Hz, 2H), 2.62 (q, *J* = 9.8 Hz, 2H), 2.43 – 2.25 (m, 1H), 1.88 – 1.73 (m, 1H), 1.37 (s, 9H). ^{13}C NMR (151 MHz, $CDCl_3$) δ 174.45, 143.34, 131.33, 129.82, 127.02 (q, *J* = 31.7 Hz), 126.76 (q, *J* = 5.3 Hz), 126.56, 124.56 (q, *J* = 273.3 Hz), 80.67, 52.98, 33.18, 27.77, 16.91. ^{19}F NMR (376 MHz, Chloroform-*d*) δ -57.68. HRMS (ESI): [M+H]$^{\oplus}$ calcd for $C_{16}H_{20}F_3O_2^{\oplus}$ 301.1410, found 301.1405.

tert-Butyl 1-(2-chloropyridin-3-yl)cyclobutane-1-carboxylate (6n)

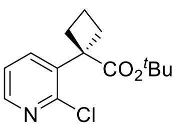

6n

Purified by flash column chromatography (hexane/ethyl acetate = 20/1), yellow oil, 19 mg, 70% yield. ^{1}H NMR (400 MHz, $CDCl_3$) δ 8.27 (dd, *J* = 4.8, 1.9 Hz, 1H), 7.64 (dd, *J* = 7.6, 1.9 Hz, 1H), 7.26 – 7.22 (m, 1H), 2.85 – 2.73 (m, 2H), 2.51 – 2.42 (m, 2H), 2.32 – 2.22 (m, 1H), 1.90 – 1.81 (m, 1H), 1.38 (s, 9H); ^{13}C NMR (151 MHz, $CDCl_3$) δ 173.35, 150.52, 147.39, 138.59, 136.65, 122.15, 81.05, 52.06, 31.10, 27.73, 16.46; HRMS (ESI): [M+Na]$^{\oplus}$ calcd for $C_{14}H_{18}NClO_2Na^{\oplus}$ 290.0918, found 290.0911.

tert-Butyl 1-(1-methyl-1H-indol-5-yl)cyclobutane-1-carboxylate (6o)

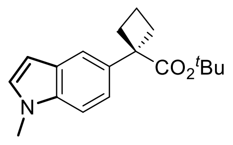

6o

Purified by flash column chromatography (hexane/ethyl acetate = 30/1), yellow oil, 29 mg, quant. yield. ^{1}H NMR (600 MHz, Chloroform-*d*) δ 7.66 – 7.43 (m, 1H), 7.27 (d, *J* = 8.5 Hz, 1H), 7.19 (dd, *J* = 8.5, 1.6 Hz, 1H), 7.04 (d, *J* = 3.0 Hz, 1H), 6.47 (d, *J* = 2.9 Hz, 1H), 3.78 (s, 3H), 2.90 – 2.78 (m, 2H), 2.59 – 2.47 (m, 2H), 2.09 – 1.95 (m, 1H), 1.95 – 1.80 (m, 1H), 1.37 (s, 9H); ^{13}C NMR (151 MHz, $CDCl_3$) δ 176.08, 135.49, 135.32, 129.03, 128.20, 120.38, 118.22, 108.86, 100.94, 79.97, 53.35, 32.90, 32.66, 27.91, 16.68; HRMS (ESI): [M+Na]$^{\oplus}$ calcd for $C_{18}H_{23}NO_2Na^{\oplus}$ 308.1621, found 308.1620.

tert-Butyl 1-(1-methyl-1H-pyrazol-4-yl)cyclobutane-1-carboxylate (6p)

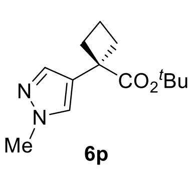

6p

Purified by flash column chromatography (hexane/ethyl acetate = 3/1), yellow oil, 17 mg, 71% yield. ^{1}H NMR (400 MHz, $CDCl_3$) δ 7.45 (s, 1H), 7.28 (s, 1H), 3.87 (s, 3H), 2.72 – 2.64 (m, 2H), 2.25 – 2.18 (m, 2H), 1.96 – 1.84 (m, 2H), 1.43 (s, 9H); ^{13}C NMR (151 MHz, $CDCl_3$) δ 175.11, 137.76, 127.93, 125.26, 80.41, 45.92, 38.97, 33.02, 27.98, 16.29; HRMS (ESI): [M+Na]$^{\oplus}$ calcd for $C_{13}H_{20}N_2O_2Na^{\oplus}$ 259.1417, found 259.1411.

tert-Butyl 1-(thiazol-4-yl)cyclobutane-1-carboxylate (6q)

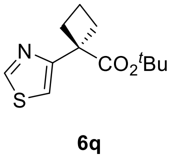

6q

Purified by flash column chromatography (hexane/ethyl acetate = 15/1), yellow oil, 21 mg, 88% yield. 4-Bromo-2-(trimethylsilyl)thiazole was used as substrate. ^{1}H NMR (400 MHz, $CDCl_3$) δ 8.77 (d, *J* = 2.0 Hz, 1H), 7.13 (d, *J* = 2.0 Hz, 1H), 2.82 – 2.67 (m, 2H), 2.59 – 2.44 (m, 2H), 2.10 –

1.89 (m, 2H), 1.43 (s, 9H); ^{13}C NMR (151 MHz, $CDCl_3$) δ 174.02, 159.64, 152.37, 113.08, 80.73, 50.89, 31.82, 27.95, 16.46; HRMS (ESI): [M+Na]$^{\oplus}$ calcd for $C_{12}H_{17}NSO_2Na^{\oplus}$ 262.0872, found 262.0860.

***tert*-Butyl 1-(benzo[b]thiophen-3-yl)cyclobutane-1-carboxylate (6r)**

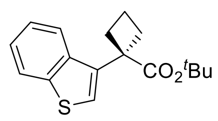

6r

Purified by flash column chromatography (hexane/ethyl acetate = 60/1), white solid, 26 mg, 92% yield. ^{1}H NMR (400 MHz, $CDCl_3$) δ 7.87 – 7.80 (m, 1H), 7.71 – 7.64 (m, 1H), 7.35 – 7.28 (m, 2H), 7.25 (s, 1H), 2.96 – 2.84 (m, 2H), 2.58 – 2.51 (m, 2H), 2.19 – 2.06 (m, 1H), 2.04 – 1.87 (m, 1H), 1.32 (s, 9H); ^{13}C NMR (151 MHz, $CDCl_3$) δ 174.36, 140.67, 138.65, 137.55, 124.10, 123.71, 123.24, 122.85, 121.82, 80.69, 50.48, 31.51, 27.84, 17.19; HRMS (ESI): [M+H]$^{\oplus}$ calcd for $C_{17}H_{21}O_2S^{\oplus}$ 289.1257, found 289.1253.

***tert*-Butyl 1-(thiophen-3-yl)cyclobutane-1-carboxylate (6s)**

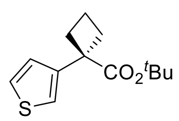

6s

Purified by flash column chromatography (hexane/ethyl acetate = 60/1), colorless oil, 19 mg, 79% yield. ^{1}H NMR (600 MHz, Chloroform-*d*) δ 7.26 – 7.24 (m, 1H), 7.15 – 7.08 (m, 1H), 7.05 (d, *J* = 4.9 Hz, 1H), 2.80 – 2.67 (m, 2H), 2.44 – 2.31 (m, 2H), 2.01 – 1.87 (m, 2H), 1.40 (s, 9H); ^{13}C NMR (151 MHz, $CDCl_3$) δ 174.79, 144.78, 126.84, 125.37, 120.29, 80.47, 50.34, 32.51, 27.95, 16.39; HRMS (ESI): [M+Na]$^{\oplus}$ calcd for $C_{13}H_{18}SO_2Na^{\oplus}$ 261.0919, found 261.0918.

***tert*-Butyl 1-(furan-2-yl)cyclobutane-1-carboxylate (6t)**

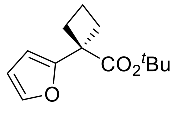

6t

Purified by flash column chromatography (hexane/ethyl acetate = 60/1), light yellow oil, 20 mg, 92% yield. ^{1}H NMR (400 MHz, $CDCl_3$) δ 7.35 (dd, *J* = 1.8, 0.8 Hz, 1H), 6.32 (dd, *J* = 3.2, 1.9 Hz, 1H), 6.17 (dd, *J* = 3.2, 0.8 Hz, 1H), 2.70 – 2.61 (m, 2H), 2.46 – 2.38 (m, 2H), 2.00 – 1.91 (m, 2H), 1.44 (s, 9H); ^{13}C NMR (151 MHz, $CDCl_3$) δ 173.25, 156.36, 141.63, 110.17, 105.27, 80.81, 48.55, 30.79, 27.95, 16.41; HRMS (ESI): [M+H]$^{\oplus}$ calcd for $C_{13}H_{19}O_3^{\oplus}$ 223.1329, found 223.1317.

***tert*-Butyl 3,3-difluoro-1-(6-methoxynaphthalen-2-yl)cyclobutane-1-carboxylate (6u)**

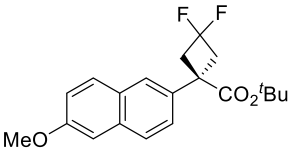

6u

Purified by flash column chromatography (hexane/ethyl acetate = 60/1), yellow solid, 35 mg, quant. yield. ^{1}H NMR (400 MHz, $CDCl_3$) δ 7.73 (d, *J* = 8.7 Hz, 2H), 7.65 (d, *J* = 1.6 Hz, 1H), 7.38 (dd, *J* = 8.5, 1.9 Hz, 1H), 7.17 (dd, *J* = 8.9, 2.5 Hz, 1H), 7.13 (d, *J* = 2.4 Hz, 1H), 3.92 (s, 3H), 3.55 – 3.38 (m, 2H), 3.14 – 3.03 (m, 2H), 1.36 (s, 9H); ^{13}C NMR (151 MHz, $CDCl_3$) δ 172.78, 158.03, 136.34, 133.62, 129.50, 128.58, 127.28, 125.06, 125.04, 119.31, 118.43 (dd, J_{C-F} = 280.9, 274.8 Hz), 105.63, 81.84, 55.37, 45.35 (dd, J_{C-F} = 23.3 Hz, 23.3 Hz), 42.94 (dd, J_{C-F} = 14.8, 7.7 Hz), 27.75; ^{19}F NMR (376 MHz, $CDCl_3$) δ -86.53 – -87.04 (m, 1F), -92.37 – -93.52 (m, 1F); HRMS (ESI): [M+Na]$^{\oplus}$ calcd for $C_{20}H_{22}F_2O_3Na^{\oplus}$ 371.1429, found 371.1423.

***tert*-Butyl 3-((*tert*-butyldimethylsilyl)oxy)-1-(4-fluoro-3-methoxyphenyl)cyclobutane-1-carboxylate (6v)**

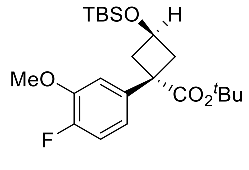

6v (major isomer)

Purified by flash column chromatography (hexane/ethyl acetate = 50/1 – 20/1), colorless oil, 41 mg, dr = 4:1, quant. yield in total. Major isomer: ^{1}H NMR (400 MHz, $CDCl_3$) δ 6.98 (dd, *J* = 11.2, 8.3 Hz, 1H), 6.78 (dd, *J* = 8.1, 2.1 Hz, 1H), 6.71 (ddd, *J* = 8.3, 4.2, 2.2 Hz, 1H), 4.39 – 4.31 (m, 1H), 3.87 (s, 3H), 3.20 – 2.96 (m, 2H), 2.38 – 2.23 (m, 2H), 1.36 (s, 9H), 0.87 (s, 9H), 0.05 (s, 6H); ^{13}C NMR (151 MHz, $CDCl_3$) δ 174.53, 151.20 (d, $J_{C\text{-}F}$ = 243.9 Hz), 147.18 (d, $J_{C\text{-}F}$ = 11.4 Hz), 141.06 (d, $J_{C\text{-}F}$ = 3.7 Hz), 118.68 (d, $J_{C\text{-}F}$ = 6.6 Hz), 115.51 (d, $J_{C\text{-}F}$ = 18.4 Hz), 111.72, 80.91, 62.80, 56.31, 45.57, 44.03, 27.83, 25.86, 18.04, -4.74; ^{19}F NMR (376 MHz, $CDCl_3$) δ -137.98; HRMS (ESI): $[M+Na]^{\oplus}$ calcd for $C_{22}H_{35}FSiO_4Na^{\oplus}$ 433.2180, found 433.2185. Minor isomer: ^{1}H NMR (400 MHz, $CDCl_3$) δ 7.10 – 6.84 (m, 3H), 4.14 – 4.00 (m, 1H), 3.89 (s, 3H), 2.83 – 2.71 (m, 2H), 2.68 – 2.55 (m, 2H), 1.34 (s, 9H), 0.87 (s, 9H), 0.02 (s, 6H); ^{13}C NMR (151 MHz, $CDCl_3$) δ 174.12, 151.36 (d, $J_{C\text{-}F}$ = 245.6 Hz), 147.32 (d, $J_{C\text{-}F}$ = 10.4 Hz), 138.31, 119.22 (d, $J_{C\text{-}F}$ = 6.4 Hz), 115.58 (d, $J_{C\text{-}F}$ = 18.4 Hz), 112.59, 80.67, 62.05, 56.35, 45.18, 43.04, 27.85, 25.86, 18.05, -4.73; ^{19}F NMR (376 MHz, $CDCl_3$) δ -137.47; HRMS (ESI): $[M+Na]^{\oplus}$ calcd for $C_{22}H_{35}FSiO_4Na^{\oplus}$ 433.2180, found 433.2182.

***tert*-Butyl 2-(furan-2-yl)-5,8-dioxaspiro[3.4]octane-2-carboxylate (6w)**

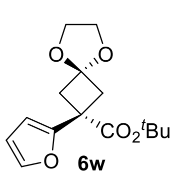

6w

Purified by flash column chromatography (hexane/ethyl acetate = 12/1), yellow oil, 27 mg, 98% yield. ^{1}H NMR (400 MHz, $CDCl_3$) δ 7.35 (dd, *J* = 1.8, 0.8 Hz, 1H), 6.32 (dd, *J* = 3.2, 1.9 Hz, 1H), 6.18 (dd, *J* = 3.3, 0.7 Hz, 1H), 3.89 (dd, *J* = 4.1, 3.0 Hz, 4H), 3.10 – 2.99 (m, 2H), 2.85 – 2.77 (m, 2H), 1.44 (s, 9H); ^{13}C NMR (151 MHz, $CDCl_3$) δ 172.16, 155.12, 141.90, 110.38, 105.96, 104.68, 81.42, 64.34, 64.12, 45.20, 39.70, 27.88; HRMS (ESI): $[M+Na]^{\oplus}$ calcd for $C_{15}H_{20}O_5Na^{\oplus}$ 303.1203, found 303.1198.

***tert*-Butyl 2-(4-fluoro-3-methoxyphenyl)-5,8-dioxaspiro[3.4]octane-2-carboxylate (6x)**

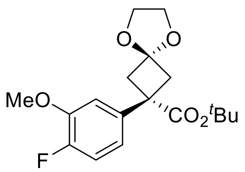

6x

Purified by flash column chromatography (hexane/ethyl acetate = 8/1), colorless oil, 25 mg, 75% yield. ^{1}H NMR (400 MHz, $CDCl_3$) δ 7.00 (dd, *J* = 11.1, 8.4 Hz, 1H), 6.89 (dd, *J* = 8.0, 2.0 Hz, 1H), 6.83 (ddd, *J* = 8.1, 4.0, 2.2 Hz, 1H), 3.99 – 3.80 (m, 7H), 3.15 (d, *J* = 13.6 Hz, 2H), 2.75 (d, *J* = 13.6 Hz, 2H), 1.36 (s, 9H); ^{13}C NMR (151 MHz, $CDCl_3$) δ 173.71, 151.37 (d, $J_{C\text{-}F}$ = 244.5 Hz), 147.22 (d, $J_{C\text{-}F}$ = 10.8 Hz), 139.28 (d, $J_{C\text{-}F}$ = 3.5 Hz), 119.06 (d, $J_{C\text{-}F}$ = 7.0 Hz), 115.49, 112.12, 104.42, 81.04, 64.33, 64.02, 56.28, 46.00, 44.45, 27.77; ^{19}F NMR (376 MHz, $CDCl_3$) δ -137.34;); HRMS (ESI): $[M+Na]^{\oplus}$ calcd for $C_{18}H_{23}FO_5Na^{\oplus}$ 361.1421, found 361.1417.

***tert*-Butyl 1-(4-fluoro-3-methoxyphenyl)-3-oxocyclobutane-1-carboxylate (6y)**

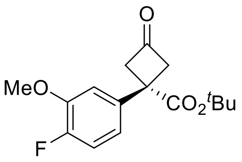

6y

To a 4 mL vial containing compound **6u** (16 mg, 0.047 mmol) in THF/H_2O (0.1 mL/0.2 mL) was added acetic acid (0.8 mL). The vial was sealed with a PTFE lined cap and stirred at 70 ^{o}C for 12 h. The reaction was quenched with saturated, aqueous $NaHCO_3$ (10 mL), and extracted with DCM (10 mL × 3). The organic layer was condensed and purified by flash column chromatography (hexane/ethyl acetate = 10/1) to give **6v** as a colorless oil

(11 mg) in 79% yield. ^{1}H NMR (400 MHz, CDCl$_3$) δ 7.06 (dd, J = 11.0, 8.4 Hz, 1H), 6.95 – 6.81 (m, 2H), 3.90 (s, 3H), 3.87 – 3.74 (m, 2H), 3.56 – 3.43 (m, 2H), 1.39 (s, 9H); ^{13}C NMR (151 MHz, CDCl$_3$) δ 202.94, 172.84, 151.84 (d, J_{C-F} = 246.2 Hz), 147.59 (d, J_{C-F} = 11.0 Hz), 137.56 (d, J_{C-F} = 4.0 Hz), 119.70 (d, J_{C-F} = 7.1 Hz), 115.94 (d, J_{C-F} = 18.6 Hz), 112.88, 82.33, 57.44, 56.51, 44.10, 27.81; ^{19}F NMR (376 MHz, CDCl$_3$) δ -136.07; HRMS (ESI): [M+Na]$^\oplus$ calcd for $C_{16}H_{19}FO_4Na^\oplus$ 317.1159, found 317.1151.

General procedure for α-arylation of azetidine esters

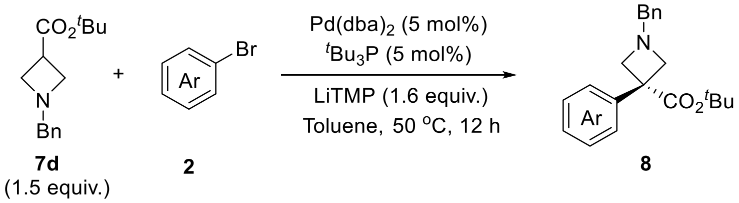

Supplementary Figure 17. General procedure for the preparation of **8**

In a dry and N$_2$-filled glovebox, the small ring ester **7d** (0.15 mmol) in toluene (0.3 mL) was added dropwise at room temperature to a 4 mL vial containing solid LiTMP (23 mg, 0.16 mmol). The resulting mixture was allowed to stir for 15 min at RT. To a second vial containing Pd(dba)$_2$ (2.9 mg, 0.0050 mmol) and ^{t}Bu$_3$P (1 M in toluene, 5.0 µL, 0.0050 mmol) were added arylbromide **2** (0.10 mmol) and toluene (0.1 mL). Then, the solutions in these two vials above were mixed together. The vial was sealed with a PTFE lined cap, removed from the dry box, and stirred at 50 °C for 12 h. The reaction solution was condensed and purified by flash column chromatography to afford the pure product **8**.

Conditions for related isolation and spectral data

tert-Butyl 1-benzyl-3-phenylazetidine-3-carboxylate (8a)

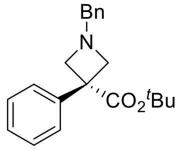

8a

Purified by flash column chromatography (hexane/ethyl acetate = 8/1), yellow oil, 32 mg, 99% yield. ^{1}H NMR (400 MHz, Chloroform-d) δ 7.36 – 7.23 (m, 10H), 3.96 (d, J = 7.7 Hz, 2H), 3.65 (s, 2H), 3.50 (d, J = 7.7 Hz, 2H), 1.39 (s, 9H); ^{13}C NMR (101 MHz, CDCl$_3$) δ 173.43, 141.50, 138.05, 128.47, 128.41, 128.34, 127.04, 126.89, 126.25, 81.07, 63.46, 63.37, 49.66, 27.89; HRMS (ESI): [M+H]$^\oplus$ calcd for $C_{21}H_{26}O_2N^\oplus$ 324.1958, found 324.1955.

tert-Butyl 1-benzyl-3-(4-((*tert*-butyldimethylsilyl)oxy)phenyl)azetidine-3-carboxylate (8b)

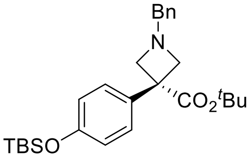

8b

Purified by flash column chromatography (hexane/ethyl acetate = 12/1), yellow oil, 43 mg, 96% yield. ^{1}H NMR (400 MHz, Chloroform-d) δ 7.34 – 7.22 (m, 5H), 7.14 (d, J = 8.6 Hz, 2H), 6.79 (d, J = 8.6 Hz, 2H), 3.92 (d, J = 7.7 Hz, 2H), 3.66 (s, 2H), 3.48 (d, J = 7.7 Hz, 2H), 1.37 (s, 9H), 0.99 (s, 9H), 0.20 (s, 6H); ^{13}C NMR (151 MHz, CDCl$_3$) δ 173.70, 154.56, 138.10, 134.23, 128.49, 128.34, 127.33, 127.04, 119.95, 80.89, 77.31, 77.10, 76.89, 63.49, 63.34, 49.03, 27.88, 25.76, 18.28, -4.34; HRMS (ESI): [M+H]$^\oplus$ calcd for $C_{27}H_{40}O_3NSi^\oplus$ 454.2772, found 454.2764.

tert-Butyl 1-benzyl-3-(4-(trifluoromethoxy)phenyl)azetidine-3-carboxylate (8c)

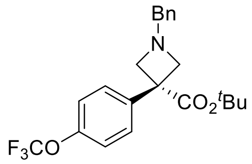

8c

Purified by flash column chromatography (hexane/ethyl acetate = 12/1), yellow oil, 36 mg, 88% yield. ^{1}H NMR (400 MHz, Chloroform-*d*) δ 7.36 – 7.23 (m, 7H), 7.18 (d, *J* = 8.3 Hz, 2H), 3.92 (d, *J* = 7.4 Hz, 2H), 3.65 (s, 2H), 3.50 (d, *J* = 7.5 Hz, 2H), 1.39 (s, 9H); ^{13}C NMR (151 MHz, $CDCl_3$) δ 172.88, 148.20, 140.21, 137.84, 128.51, 128.41, 127.83, 127.18, 120.93, 120.57 (q, *J* = 257.1 Hz), 81.48, 63.38, 63.25, 49.20, 27.90; ^{19}F NMR (376 MHz, $CDCl_3$) δ -57.12; HRMS (ESI): $[M+Na]^{\oplus}$ calcd for $C_{22}H_{24}F_3O_3NNa^{\oplus}$ 430.1600, found 430.1594.

tert-Butyl 1-benzyl-3-(4-(difluoromethyl)phenyl)azetidine-3-carboxylate (8d)

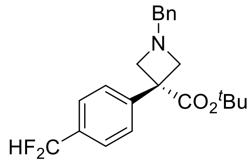

8d

Purified by flash column chromatography (hexane/ethyl acetate = 8/1), yellow oil, 36 mg, 97% yield. ^{1}H NMR (400 MHz, Chloroform-*d*) δ 7.49 (d, *J* = 8.2 Hz, 2H), 7.39 (d, *J* = 8.2 Hz, 2H), 7.36 – 7.23 (m, 5H), 6.65 (t, *J* = 56.5 Hz, 1H), 3.96 (d, *J* = 7.8 Hz, 2H), 3.66 (s, 2H), 3.51 (d, *J* = 7.7 Hz, 2H), 1.40 (s, 9H); ^{13}C NMR (151 MHz, $CDCl_3$) δ 172.81, 144.28, 137.84, 133.06 (t, *J* = 22.4 Hz), 128.49, 128.39, 127.15, 126.69, 125.74 (t, *J* = 6.0 Hz), 114.68 (t, *J* = 238.5 Hz), 81.46, 63.37, 63.23, 49.61, 27.87; ^{19}F NMR (376 MHz, Chloroform-*d*) δ -109.72 (d, *J* = 56.5 Hz); HRMS (ESI): $[M+Na]^{\oplus}$ calcd for $C_{22}H_{25}F_2O_2NNa^{\oplus}$ 396.1745, found 396.1754.

tert-Butyl 1-benzyl-3-(4-(trimethylsilyl)phenyl)azetidine-3-carboxylate (8e)

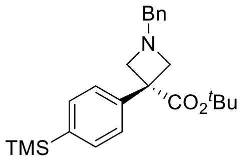

8e

Purified by flash column chromatography (hexane/ethyl acetate = 15/1), light yellow oil, 40 mg, quant. yield. ^{1}H NMR (400 MHz, Chloroform-*d*) δ 7.50 (d, *J* = 7.9 Hz, 2H), 7.40 – 7.13 (m, 7H), 3.97 (d, *J* = 7.5 Hz, 2H), 3.66 (s, 2H), 3.51 (d, *J* = 7.5 Hz, 2H), 1.42 (s, 9H), 0.28 (s, 9H); ^{13}C NMR (151 MHz, $CDCl_3$) δ 173.40, 141.94, 138.83, 138.06, 133.47, 128.47, 128.34, 127.05, 125.56, 81.10, 63.43, 63.41, 49.63, 27.93, -1.04; HRMS (ESI): $[M+H]^{\oplus}$ calcd for $C_{24}H_{34}O_2NSi^{\oplus}$ 396.2353, found 396.2353.

tert-Butyl 1-benzyl-3-(4-(trifluoromethyl)phenyl)azetidine-3-carboxylate (8f)

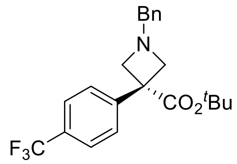

8f

Purified by flash column chromatography (hexane/ethyl acetate = 12/1), yellow oil, 39 mg, quant. yield. ^{1}H NMR (400 MHz, Chloroform-*d*) δ 7.59 (d, *J* = 8.2 Hz, 2H), 7.41 (d, *J* = 8.1 Hz, 2H), 7.34 – 7.23 (m, 5H), 3.93 (d, *J* = 7.7 Hz, 2H), 3.64 (s, 2H), 3.50 (d, *J* = 7.7 Hz, 2H), 1.38 (s, 9H); ^{13}C NMR (101 MHz, $CDCl_3$) δ 172.55, 145.45, 137.75, 129.26 (q, *J* = 32.5 Hz), 128.49, 128.41, 127.19, 126.76, 125.45 (q, *J* = 3.4 Hz), 81.65, 77.42, 77.10, 76.78, 63.35, 63.16, 49.61, 27.86 (The carbon signal of CF_3 group was not observed); ^{19}F NMR (376 MHz, $CDCl_3$) δ -61.85; HRMS (ESI): $[M+H]^{\oplus}$ calcd for $C_{22}H_{25}O_2NF_3^{\oplus}$ 392.1832, found 392.1836.

***tert*-Butyl 1-benzyl-3-(4-(*N*,*N*-dimethylsulfamoyl)phenyl)azetidine-3-carboxylate (8g)**

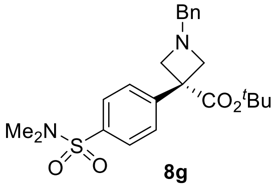

8g

Purified by flash column chromatography (hexane/ethyl acetate = 3/1), yellow oil, 34 mg, 80% yield. ^{1}H NMR (400 MHz, Chloroform-*d*) δ 7.75 (d, *J* = 8.4 Hz, 2H), 7.48 (d, *J* = 8.3 Hz, 2H), 7.36 – 7.24 (m, 5H), 3.93 (d, *J* = 7.5 Hz, 2H), 3.66 (s, 2H), 3.55 (d, *J* = 7.5 Hz, 2H), 2.72 (s, 6H), 1.38 (s, 9H); ^{13}C NMR (151 MHz, $CDCl_3$) δ 172.24, 146.40, 137.58, 134.22, 128.46, 128.42, 127.96, 127.23, 127.06, 81.83, 63.28, 63.02, 49.61, 37.97, 27.83; HRMS (ESI): $[M+Na]^{\oplus}$ calcd for $C_{23}H_{30}O_4N_2SNa^{\oplus}$ 453.1818, found 453.1825.

***tert*-Butyl 1-benzyl-3-(4-(diethylcarbamoyl)phenyl)azetidine-3-carboxylate (8h)**

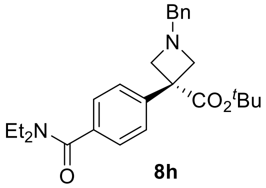

8h

Purified by flash column chromatography (hexane/ethyl acetate = 1/1), light yellow oil, 33 mg, 78% yield. ^{1}H NMR (400 MHz, Chloroform-*d*) δ 7.43 – 7.23 (m, 9H), 3.93 (d, *J* = 7.6 Hz, 2H), 3.65 (s, 2H), 3.57 – 3.50 (m, 4H), 3.26 (s, 2H), 1.38 (s, 9H), 1.24 (s, 3H), 1.11 (s, 3H); ^{13}C NMR (151 MHz, $CDCl_3$) δ ^{13}C NMR (151 MHz, $CDCl_3$) δ 172.95, 171.08, 142.46, 137.81, 135.88, 128.46, 128.36, 127.12, 126.57, 126.36, 81.35, 63.36, 63.20, 49.49, 43.37, 39.35, 27.86, 14.30, 12.93; HRMS (ESI): $[M+Na]^{\oplus}$ calcd for $C_{26}H_{34}O_3N_2Na^{\oplus}$ 445.2461, found 445.2450.

***tert*-Butyl 1-benzyl-3-(4-morpholinophenyl)azetidine-3-carboxylate (8i)**

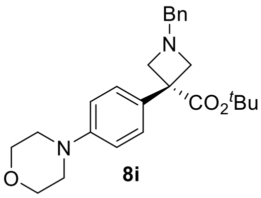

8i

Purified by flash column chromatography (hexane/ethyl acetate = 3/1), yellow oil, 41 mg, quant. yield. ^{1}H NMR (400 MHz, Chloroform-*d*) δ 7.33 – 7.18 (m, 7H), 6.88 (d, *J* = 8.7 Hz, 2H), 3.92 (d, *J* = 7.4 Hz, 2H), 3.89 – 3.83 (m, 4H), 3.65 (s, 2H), 3.47 (d, *J* = 7.4 Hz, 2H), 3.19 – 3.11 (m, 4H), 1.39 (s, 9H); ^{13}C NMR (151 MHz, $CDCl_3$) δ 173.70, 150.06, 138.10, 132.85, 128.47, 128.31, 127.07, 127.01, 115.54, 80.89, 66.97, 63.45, 63.38, 49.35, 48.92, 27.93; HRMS (ESI): $[M+H]^{\oplus}$ calcd for $C_{25}H_{33}O_3N_2^{\oplus}$ 409.2486, found 409.2484.

***tert*-Butyl 1-benzyl-3-(3-chlorophenyl)azetidine-3-carboxylate (8j)**

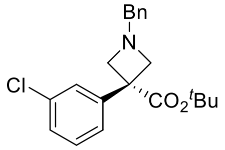

8j

Purified by flash column chromatography (hexane/ethyl acetate = 12/1), colorless oil, 28 mg, 78% yield. ^{1}H NMR (400 MHz, Chloroform-*d*) δ 7.40 – 7.15 (m, 9H), 3.92 (d, *J* = 7.7 Hz, 2H), 3.65 (s, 2H), 3.48 (d, *J* = 7.7 Hz, 2H), 1.40 (s, 9H); ^{13}C NMR (151 MHz, $CDCl_3$) δ 172.73, 143.42, 137.80, 134.33, 129.70, 128.50, 128.39, 127.19, 127.15, 126.71, 124.55, 81.50, 63.38, 63.13, 49.41, 27.88; HRMS (ESI): $[M+H]^{\oplus}$ calcd for $C_{21}H_{25}O_2NCl^{\oplus}$ 358.1568, found 358.1570.

***tert*-Butyl 1-benzyl-3-(3-(*tert*-butoxycarbonyl)phenyl)azetidine-3-carboxylate (8k)**

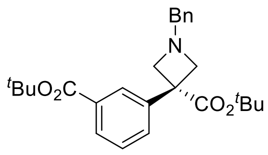

8k

Purified by flash column chromatography (hexane/ethyl acetate = 8/1), yellow oil, 28 mg, 67% yield. ^{1}H NMR (400 MHz, Chloroform-*d*) δ 7.97 – 7.80 (m, 2H), 7.48 (d, *J* = 7.8 Hz, 1H), 7.38 (t, *J* = 7.7 Hz, 1H), 7.33 – 7.23 (m, 5H), 3.93 (d, *J* = 7.6 Hz, 2H), 3.65 (s, 2H), 3.51 (d, *J* = 7.6 Hz, 2H), 1.59 (s, 9H), 1.39 (s, 9H); ^{13}C NMR (101 MHz, $CDCl_3$) δ 172.98, 165.65, 141.68, 137.92, 132.23, 130.45, 128.48, 128.37, 128.09, 127.34,

127.11, 81.37, 81.17, 63.41, 63.30, 49.47, 28.28, 27.91 (one carbon signal of aromatic rings was not observed because of overlap). HRMS (ESI): [M+H]$^{\oplus}$ calcd for $C_{26}H_{34}O_4N^{\oplus}$ 424.2482, found 424.2478.

***tert*-Butyl 1-benzyl-3-(3-(methylthio)phenyl)azetidine-3-carboxylate (8l)**

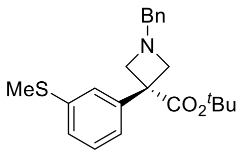

8l

Purified by flash column chromatography (hexane/ethyl acetate = 12/1), yellow oil, 28 mg, 75% yield. ^{1}H NMR (400 MHz, Chloroform-*d*) δ 7.40 – 7.26 (m, 6H), 7.23 – 7.14 (m, 2H), 7.09 (d, *J* = 7.6 Hz, 1H), 3.98 (d, *J* = 7.5 Hz, 2H), 3.69 (s, 2H), 3.52 (d, *J* = 7.5 Hz, 2H), 2.52 (s, 3H), 1.43 (s, 9H); ^{13}C NMR (151 MHz, $CDCl_3$) δ 173.10, 142.14, 138.63, 137.89, 128.85, 128.49, 128.36, 127.10, 125.13, 124.54, 123.10, 81.24, 63.38, 63.22, 49.59, 27.90, 15.89; HRMS (ESI): [M+H]$^{\oplus}$ calcd for $C_{22}H_{28}O_2NS^{\oplus}$ 370.1835, found 370.1834.

***tert*-Butyl 3-(3-(1,3-dioxolan-2-yl)phenyl)-1-benzylazetidine-3-carboxylate (8m)**

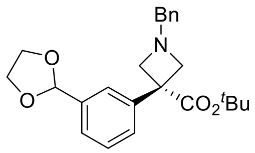

8m

Purified by flash column chromatography (hexane/ethyl acetate = 5/1), yellow oil, 37 mg, 94% yield. ^{1}H NMR (400 MHz, Chloroform-*d*) δ 7.42 – 7.24 (m, 9H), 5.80 (s, 1H), 4.16 – 3.94 (m, 6H), 3.65 (s, 2H), 3.50 (d, *J* = 7.3 Hz, 2H), 1.38 (s, 9H); ^{13}C NMR (151 MHz, $CDCl_3$) δ 173.20, 143.77, 141.62, 138.14, 137.98, 128.49, 128.33, 127.12, 127.06, 125.06, 124.53, 103.70, 81.16, 65.34, 63.40, 63.32, 49.64, 27.87; HRMS (ESI): [M+Na]$^{\oplus}$ calcd for $C_{24}H_{29}O_4NNa^{\oplus}$ 418.1988, found 418.1996.

***tert*-Butyl 1-benzyl-3-(2-isopropylphenyl)azetidine-3-carboxylate (8n)**

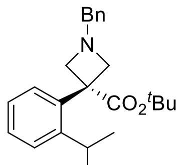

8n

Purified by flash column chromatography (hexane/ethyl acetate = 10/1), yellow oil, 30 mg, 81% yield. ^{1}H NMR (400 MHz, Chloroform-*d*) δ 7.42 – 7.22 (m, 7H), 7.21 – 7.10 (m, 1H), 7.03 (d, *J* = 7.7 Hz, 1H), 4.28 (d, *J* = 7.3 Hz, 2H), 3.78 (s, 2H), 3.59 (br s, 2H), 2.94 – 2.68 (m, 1H), 1.40 (s, 9H), 1.16 (d, *J* = 6.8 Hz, 6H); ^{13}C NMR (151 MHz, $CDCl_3$) δ 171.54, 146.60, 134.50, 129.99, 129.77, 129.25, 129.04, 128.63, 127.02, 126.98, 126.27, 83.29, 60.47, 59.60, 48.84, 29.66, 27.74, 24.40; HRMS (ESI): [M+H]$^{\oplus}$ calcd for $C_{24}H_{32}O_2N^{\oplus}$ 366.2428, found 366.2418.

***tert*-Butyl 1-benzyl-3-(4-fluoro-3-methoxyphenyl)azetidine-3-carboxylate (8o)**

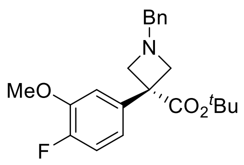

8o

Purified by flash column chromatography (hexane/ethyl acetate = 10/1), yellow oil, 33 mg, 88% yield. ^{1}H NMR (300 MHz, Chloroform-*d*) δ 7.39 – 7.26 (m, 5H), 7.06 (dd, *J* = 11.1, 8.4 Hz, 1H), 6.92 (dd, *J* = 8.1, 2.1 Hz, 1H), 6.85 (ddd, *J* = 8.3, 4.2, 2.2 Hz, 1H), 3.96 (d, *J* = 7.8 Hz, 2H), 3.91 (s, 3H), 3.69 (s, 2H), 3.51 (d, *J* = 7.8 Hz, 2H), 1.43 (s, 9H); ^{13}C NMR (151 MHz, $CDCl_3$) δ 173.08, 151.46 (d, *J* = 245.6 Hz), 147.41 (d, *J* = 10.7 Hz), 137.82, 137.70 (d, *J* = 3.6 Hz), 128.45, 128.33, 127.09, 118.69 (d, *J* = 6.9 Hz), 115.76 (d, *J* = 18.5 Hz), 111.66, 81.23, 77.31, 77.10, 76.89, 63.36, 63.22, 56.25, 49.34, 27.85; ^{19}F NMR (376 MHz, $CDCl_3$) δ -136.98; HRMS (ESI): [M+H]$^{\oplus}$ calcd for $C_{22}H_{27}O_3NF^{\oplus}$ 372.1969, found 372.1971.

***tert*-Butyl 1-benzyl-3-(2,2-difluorobenzo[d][1,3]dioxol-5-yl)azetidine-3-carboxylate (8p)**

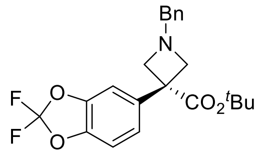

8p

Purified by flash column chromatography (hexane/ethyl acetate = 12/1), yellow oil, 31 mg, 76% yield. ^{1}H NMR (600 MHz, Chloroform-*d*) δ 7.37 – 7.24 (m, 5H), 7.12 – 6.92 (m, 3H), 3.89 (d, *J* = 7.3 Hz, 2H), 3.64 (s, 2H), 3.45 (d, *J* = 7.3 Hz, 2H), 1.40 (s, 9H); ^{13}C NMR (151 MHz, $CDCl_3$) δ 172.72, 143.92, 142.72, 137.78, 137.72, 131.76 (t, *J* = 255.2 Hz), 128.50, 128.42, 127.20, 121.60, 109.23, 108.05, 81.61, 63.34, 63.29, 49.44, 27.91; ^{19}F NMR (376 MHz, $CDCl_3$) δ -49.15; HRMS (ESI): $[M+H]^{\oplus}$ calcd for $C_{22}H_{24}O_4NF_2^{\oplus}$ 404.1668, found 404.1675.

***tert*-Butyl 3-(3-adamantan-1-yl)-4-methoxyphenyl)-1-benzylazetidine-3-carboxylate (8q)**

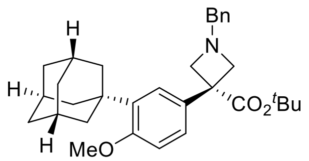

8q

Purified by flash column chromatography (hexane/ethyl acetate = 12/1), light yellow oil, 44 mg, 89% yield. ^{1}H NMR (400 MHz, Chloroform-*d*) δ 7.37 – 7.21 (m, 5H), 7.18 – 7.04 (m, 2H), 6.82 (d, *J* = 8.4 Hz, 1H), 3.96 (d, *J* = 7.2 Hz, 2H), 3.83 (s, 3H), 3.67 (s, 2H), 3.49 (d, *J* = 7.2 Hz, 2H), 2.09 (s, 9H), 1.78 (s, 6H), 1.40 (s, 9H); ^{13}C NMR (151 MHz, $CDCl_3$) δ 173.86, 157.74, 138.33, 138.15, 133.07, 128.50, 128.31, 127.01, 124.80, 124.31, 111.38, 80.78, 63.46, 55.06, 49.30, 40.69, 37.19, 37.14, 29.15, 27.95 (one alkyl carbon signal was not observed); HRMS (ESI): $[M+Na]^{\oplus}$ calcd for $C_{32}H_{41}O_3NNa^{\oplus}$ 510.2978, found 510.2968.

***tert*-Butyl (*E*)-1-benzyl-3-styrylazetidine-3-carboxylate (8r)**

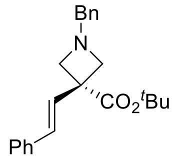

8r

Purified by flash column chromatography (hexane/ethyl acetate = 12/1), yellow oil, 29 mg, 84% yield. ^{1}H NMR (400 MHz, Chloroform-*d*) δ 7.49 – 7.24 (m, 11H), 6.58 (q, *J* = 16.2 Hz, 2H), 3.72 – 3.71 (m, 4H), 3.45 (d, *J* = 7.3 Hz, 2H), 1.54 (s, 9H); ^{13}C NMR (151 MHz, $CDCl_3$) δ 172.99, 137.93, 136.91, 130.41, 129.76, 128.61, 128.50, 128.38, 127.66, 127.10, 126.45, 81.26, 63.34, 62.60, 47.12, 28.10; HRMS (ESI): $[M+H]^{\oplus}$ calcd for $C_{23}H_{28}O_2N^{\oplus}$ 350.2115, found 350.2109.

***tert*-Butyl 1-benzyl-3-(2-methylprop-1-en-1-yl)azetidine-3-carboxylate (8s)**

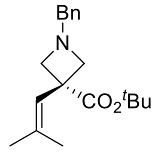

8s

Purified by flash column chromatography (hexane/ethyl acetate = 8/1), light yellow oil, 26 mg, 86% yield. ^{1}H NMR (400 MHz, Chloroform-*d*) δ 7.39 – 7.07 (m, 5H), 5.37 (s, 1H), 3.74 (d, *J* = 7.5 Hz, 2H), 3.62 (s, 2H), 3.20 (d, *J* = 7.4 Hz, 2H), 1.71 (s, 3H), 1.53 (s, 3H), 1.45 (s, 9H). ^{13}C NMR (151 MHz, $CDCl_3$) δ 173.60, 138.03, 135.92, 128.51, 128.32, 127.02, 125.79, 80.59, 63.98, 63.45, 45.27, 28.05, 25.78, 18.76. HRMS (ESI): $[M+H]^{\oplus}$ calcd for $C_{19}H_{28}O_2N^{\oplus}$ 302.2115, found 302.2109.

***tert*-Butyl 1-benzyl-3-(6-(methylthio)pyridin-3-yl)azetidine-3-carboxylate (8t)**

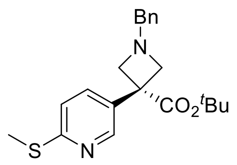

8t

Purified by flash column chromatography (hexane/ethyl acetate = 8/1), yellow oil, 28 mg, 76% yield. ^{1}H NMR (400 MHz, Chloroform-*d*) δ 8.44 (d, *J* = 1.9 Hz, 1H), 7.49 (dd, *J* = 8.4, 2.3 Hz, 1H), 7.38 – 7.27 (m, 5H), 7.19 (d, *J* = 8.3 Hz, 1H), 3.92 (d, *J* = 7.5 Hz, 2H), 3.68 (s, 2H), 3.52 (d, *J* = 7.5 Hz, 2H), 2.60 (s, 3H), 1.41 (s, 9H); ^{13}C NMR (151 MHz, $CDCl_3$) δ

172.54, 158.52, 147.56, 137.68, 134.08, 132.57, 128.47, 128.40, 127.18, 121.09, 81.69, 63.26, 62.95, 47.37, 27.91, 13.38; HRMS (ESI): [M+H]$^{\oplus}$ calcd for $C_{21}H_{27}O_2N_2S^{\oplus}$ 371.1788, found 371.1786.

***tert*-Butyl 1-benzyl-3-(1-methyl-1H-indol-5-yl)azetidine-3-carboxylate (8u)**

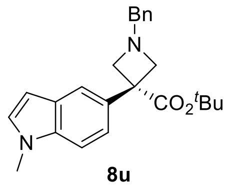

8u

Purified by flash column chromatography (hexane/ethyl acetate = 5/1), light yellow oil, 36 mg, 96% yield. ^{1}H NMR (400 MHz, Chloroform-*d*) δ 7.51 (s, 1H), 7.32 – 7.24 (m, 6H), 7.13 (d, *J* = 8.5 Hz, 1H), 7.05 (d, *J* = 3.0 Hz, 1H), 6.45 (d, *J* = 3.0 Hz, 1H), 4.06 (d, *J* = 7.2 Hz, 2H), 3.79 (s, 3H), 3.66 (s, 2H), 3.54 (d, *J* = 7.3 Hz, 2H), 1.38 (s, 9H); ^{13}C NMR (151 MHz, $CDCl_3$) δ 174.25, 135.78, 132.62, 129.29, 128.53, 128.42, 128.32, 126.99, 120.13, 118.47, 109.15, 101.08, 80.73, 63.87, 63.59, 49.76, 32.94, 27.96; HRMS (ESI): [M+H]$^{\oplus}$ calcd for $C_{24}H_{29}O_2N_2^{\oplus}$ 377.2224, found 377.2226.

***tert*-Butyl 1-benzyl-3-(1-(triisopropylsilyl)-1*H*-pyrrol-3-yl)azetidine-3-carboxylate (8v)**

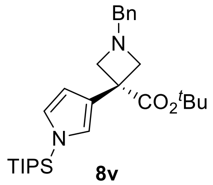

8v

Purified by flash column chromatography (hexane/ethyl acetate = 15/1), light yellow oil, 35 mg, 75% yield. ^{1}H NMR (400 MHz, Chloroform-*d*) δ 7.39 – 7.20 (m, 5H), 6.77 – 6.68 (m, 1H), 6.68 – 6.56 (m, 1H), 6.29 (dd, *J* = 2.6, 1.4 Hz, 1H), 3.85 (d, *J* = 7.5 Hz, 2H), 3.69 (s, 2H), 3.42 (d, *J* = 7.5 Hz, 2H), 1.48 – 1.38 (m, 12H), 1.09 (d, *J* = 7.5 Hz, 18H); ^{13}C NMR (151 MHz, $CDCl_3$) δ 174.34, 138.38, 128.50, 128.26, 126.90, 126.53, 124.06, 121.00, 109.40, 80.36, 64.36, 63.39, 44.60, 28.02, 17.91, 11.74; HRMS (ESI): [M+H]$^{\oplus}$ calcd for $C_{28}H_{45}O_2N_2Si^{\oplus}$ 469.3245, found 469.3241.

***tert*-Butyl 1-benzyl-3-(quinolin-6-yl)azetidine-3-carboxylate (8w)**

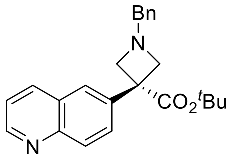

8w

Purified by flash column chromatography (hexane/ethyl acetate = 2/1), yellow oil, 28 mg, 74% yield. ^{1}H NMR (400 MHz, Chloroform-*d*) δ 8.91 (dd, *J* = 4.1, 1.5 Hz, 1H), 8.11 (dd, *J* = 20.0, 8.5 Hz, 2H), 7.74 – 7.62 (m, 2H), 7.45 – 7.39 (m, 1H), 7.36 – 7.22 (m, 5H), 4.03 (d, *J* = 7.5 Hz, 2H), 3.68 (s, 2H), 3.63 (d, *J* = 7.5 Hz, 2H), 1.38 (s, 9H); ^{13}C NMR (151 MHz, $CDCl_3$) δ 172.94, 150.47, 147.42, 139.69, 137.77, 136.11, 129.72, 128.54, 128.40, 128.35, 128.06, 127.17, 124.84, 121.45, 81.54, 63.40, 63.19, 49.65, 27.89; HRMS (ESI): [M+H]$^{\oplus}$ calcd for $C_{24}H_{27}O_2N_2^{\oplus}$ 375.2067, found 375.2063.

Studies on the debenzylation-alkylation process

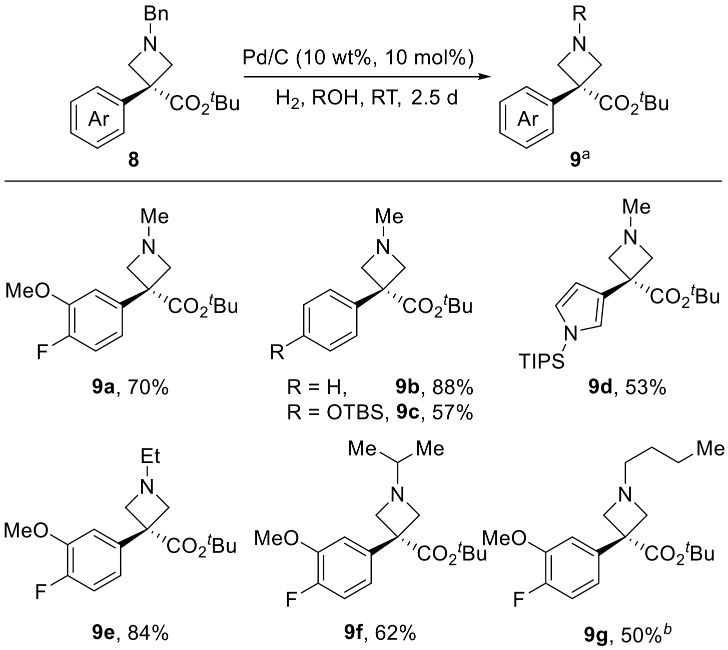

[a]Isolated yields. [b]Cyclopropylmethanol was sued as solvent.

Supplementary Figure 18. Scope of the debenzylation-alkylation process

To a well-stirred solution of arylated azetidine **8** (0.1 mmol) in alcohol solvent (2 mL) at room temperature was added palladium (10 wt% on carbon, 11 mg, 0.010 mmol). The resulting mixture was allowed to stir under hydrogen (1 atm.) for 2.5 days. The reaction solution was filtered through Celite, condensed, and purified by flash column chromatography to afford the pure substrate **9**.

***tert*-Butyl 3-(4-fluoro-3-methoxyphenyl)-1-methylazetidine-3-carboxylate (9a)**

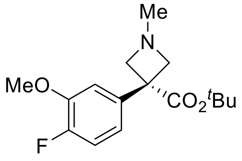

9a

Methanol was used as solvent. The reaction solution was filtered through Celite and condensed to afford pure **9a**. Colorless oil, 21 mg, 70% yield. ^{1}H NMR (400 MHz, Chloroform-*d*) δ 7.16 – 6.95 (m, 1H), 6.96 – 6.68 (m, 2H), 4.05 – 3.70 (m, 5H), 3.45 (d, *J* = 6.7 Hz, 2H), 2.37 (s, 3H), 1.42 (s, 9H); ^{13}C NMR (101 MHz, $CDCl_3$) δ 172.99, 151.51 (d, *J* = 245.1 Hz), 147.47 (d, *J* = 10.9 Hz), 137.55 (d, *J* = 3.7 Hz), 118.70 (d, *J* = 6.9 Hz), 115.84 (d, *J* = 18.6 Hz), 111.63, 81.31, 65.16, 56.33, 49.11, 46.04, 27.87; ^{19}F NMR (376 MHz, $CDCl_3$) δ -136.84; HRMS (ESI): $[M+H]^{\oplus}$ calcd for $C_{16}H_{23}O_3NF^{\oplus}$ 296.1656, found 296.1656.

***tert*-Butyl 1-methyl-3-phenylazetidine-3-carboxylate (9b)**

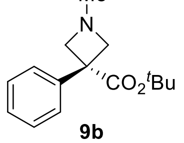

9b

Methanol was used as solvent. The compound was purified by flash column chromatography (hexane/ethyl acetate = 2/1 with 0.5% Et_3N), yellow oil, 22 mg, 88% yield. ^{1}H NMR (400 MHz, Chloroform-*d*) δ 7.37 – 7.22 (m, 5H), 4.01 (d, *J* = 7.6 Hz, 2H), 3.47 (d, *J* = 7.6 Hz, 2H), 2.35 (s, 3H), 1.38 (s, 9H); ^{13}C NMR (101 MHz, $CDCl_3$) δ 173.26, 141.29, 128.44, 126.90, 126.16, 81.13, 65.22, 49.38, 46.05, 27.87; HRMS (ESI): $[M+H]^{\oplus}$ calcd for $C_{15}H_{22}O_2N^{\oplus}$ 248.1645, found 248.1643.

tert-Butyl 3-(4-((tert-butyldimethylsilyl)oxy)phenyl)-1-methylazetidine-3-carboxylate (9c)

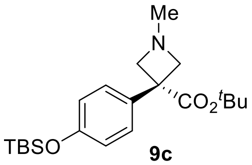

9c

Methanol was used as solvent. The compound was purified by flash column chromatography (hexane/ethyl acetate = 1/1 with 0.5% Et_3N), colorless oil, 21 mg, 57% yield. ^{1}H NMR (400 MHz, Chloroform-*d*) δ 7.10 (d, *J* = 8.5 Hz, 2H), 6.77 (d, *J* = 8.5 Hz, 2H), 3.91 (d, *J* = 7.5 Hz, 2H), 3.43 (d, *J* = 7.4 Hz, 2H), 2.33 (s, 3H), 1.35 (s, 9H), 0.97 (s, 9H), 0.18 (s, 6H); ^{13}C NMR (151 MHz, $CDCl_3$) δ 173.48, 154.60, 133.97, 127.26, 120.01, 81.00, 65.14, 48.74, 45.99, 27.88, 25.76, 18.28, -4.34; HRMS (ESI): [M+H]$^\oplus$ calcd for $C_{21}H_{36}O_3NSi^\oplus$ 378.2459, found 378.2457.

tert-Butyl 1-methyl-3-(1-(triisopropylsilyl)-1*H*-pyrrol-3-yl)azetidine-3-carboxylate (9d)

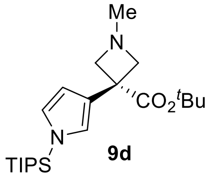

9d

Methanol was used as solvent. The compound was purified by flash column chromatography (hexane/ethyl acetate = 1/3 with 0.5% Et_3N), colorless oil, 21 mg, 53% yield. ^{1}H NMR (400 MHz, Chloroform-*d*) δ 6.68 (s, 1H), 6.61 (s, 1H), 6.27 (s, 1H), 3.84 (d, *J* = 7.4 Hz, 2H), 3.38 (d, *J* = 7.4 Hz, 2H), 2.35 (s, 3H), 1.46 – 1.36 (m, 12H), 1.07 (d, *J* = 7.5 Hz, 18H); ^{13}C NMR (151 MHz, $CDCl_3$) δ 174.12, 126.12, 124.16, 121.01, 109.32, 80.51, 66.16, 45.96, 44.27, 28.01, 17.90, 11.75; HRMS (ESI): [M+H]$^\oplus$ calcd for $C_{22}H_{41}O_2N_2Si^\oplus$ 393.2932, found 393.2916.

tert-Butyl 1-ethyl-3-(4-fluoro-3-methoxyphenyl)azetidine-3-carboxylate (9e)

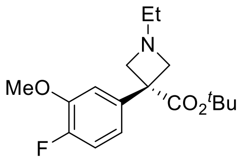

9e

Ethanol was used as solvent. The compound was purified by flash column chromatography (hexane/ethyl acetate = 2/1 with 0.5% Et_3N), colorless oil, 26 mg, 84% yield. ^{1}H NMR (400 MHz, Chloroform-*d*) δ 7.01 (dd, *J* = 11.1, 8.4 Hz, 1H), 6.87 (dd, *J* = 8.0, 2.1 Hz, 1H), 6.81 (ddd, *J* = 8.3, 4.2, 2.2 Hz, 1H), 3.86 (d, *J* = 12.9 Hz, 5H), 3.41 (d, *J* = 7.6 Hz, 2H), 2.46 (q, *J* = 7.2 Hz, 2H), 1.37 (s, 9H), 0.96 (t, *J* = 7.2 Hz, 3H); ^{13}C NMR (151 MHz, $CDCl_3$) δ 173.08, 151.55 (d, *J* = 245.1 Hz), 147.48 (d, *J* = 11.0 Hz), 137.76 (d, *J* = 3.7 Hz), 118.79 (d, *J* = 6.9 Hz), 115.84 (d, *J* = 18.4 Hz), 111.77, 81.28, 62.98, 56.36, 53.71, 49.08, 27.89, 12.28; ^{19}F NMR (376 MHz, $CDCl_3$) δ -136.95; HRMS (ESI): [M+H]$^\oplus$ calcd for $C_{17}H_{25}O_3NF^\oplus$ 310.1813, found 310.1814.

tert-Butyl 3-(4-fluoro-3-methoxyphenyl)-1-isopropylazetidine-3-carboxylate (9f)

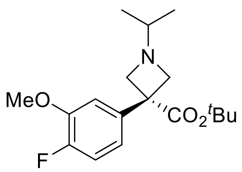

9f

iso-Propanol was used as solvent. The compound was purified by flash column chromatography (hexane/ethyl acetate = 2/1 with 0.5% Et_3N), colorless oil, 20 mg, 62% yield. ^{1}H NMR (400 MHz, Chloroform-*d*) δ 7.01 (dd, *J* = 11.1, 8.4 Hz, 1H), 6.89 (dd, *J* = 8.1, 1.9 Hz, 1H), 6.83 (ddd, *J* = 8.0, 4.1, 2.1 Hz, 1H), 3.88 (s, 3H), 3.82 (d, *J* = 7.5 Hz, 2H), 3.47 (d, *J* = 7.5 Hz, 2H), 2.38 – 2.19 (m, 1H), 1.36 (s, 9H), 0.93 (d, *J* = 6.2 Hz, 6H); ^{13}C NMR (151 MHz, $CDCl_3$) δ 173.11, 151.59 (d, *J* = 244.3 Hz), 147.48 (d, *J* = 11.0 Hz), 137.91 (d, *J* = 3.7 Hz), 1118.93 (d, *J* = 6.9 Hz), 115.84 (d, *J* = 18.5 Hz), 111.95, 81.22, 61.93, 58.89, 56.39, 47.66, 27.91, 19.48; ^{19}F NMR (376 MHz, $CDCl_3$) δ -137.08; HRMS (ESI): [M+H]$^\oplus$ calcd for $C_{18}H_{27}O_3NF^\oplus$ 324.1969 found 324.1969.

***tert*-Butyl 1-butyl-3-(4-fluoro-3-methoxyphenyl)azetidine-3-carboxylate (9g)**

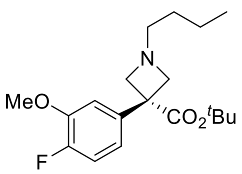

9g

Cyclopropylmethanol was used as solvent. The compound was purified by flash column chromatography (hexane/ethyl acetate = 3/1 with 0.5% Et_3N), colorless oil, 17 mg, 50% yield. ^1H NMR (400 MHz, Chloroform-*d*) δ 7.01 (dd, *J* = 11.1, 8.5 Hz, 1H), 6.92 – 6.84 (m, 1H), 6.81 (ddd, *J* = 8.2, 3.8, 2.0 Hz, 1H), 4.04 – 3.72 (m, 5H), 3.42 (d, *J* = 7.3 Hz, 2H), 2.42 (t, *J* = 7.0 Hz, 2H), 1.42 – 1.26 (m, 13H), 0.89 (t, *J* = 6.8 Hz, 3H); ^{13}C NMR (101 MHz, $CDCl_3$) δ 173.15, 155.39 (d, *J* = 531.8 Hz), 147.46 (d, *J* = 10.6 Hz), 137.83 (d, *J* = 3.9 Hz), 118.77 (d, *J* = 6.8 Hz), 115.82 (d, *J* = 18.5 Hz), 111.79 – 111.69 (m), 81.25, 63.43, 59.71, 56.35, 49.28, 29.71, 27.91, 20.60, 14.15; ^{19}F NMR (376 MHz, $CDCl_3$) δ -137.06; HRMS (ESI): $[M+H]^{\oplus}$ calcd for $C_{19}H_{29}O_3NF^{\oplus}$ 338.2126 found 338.2114.

Mechanistic studies on the debenzylation-alkylation process

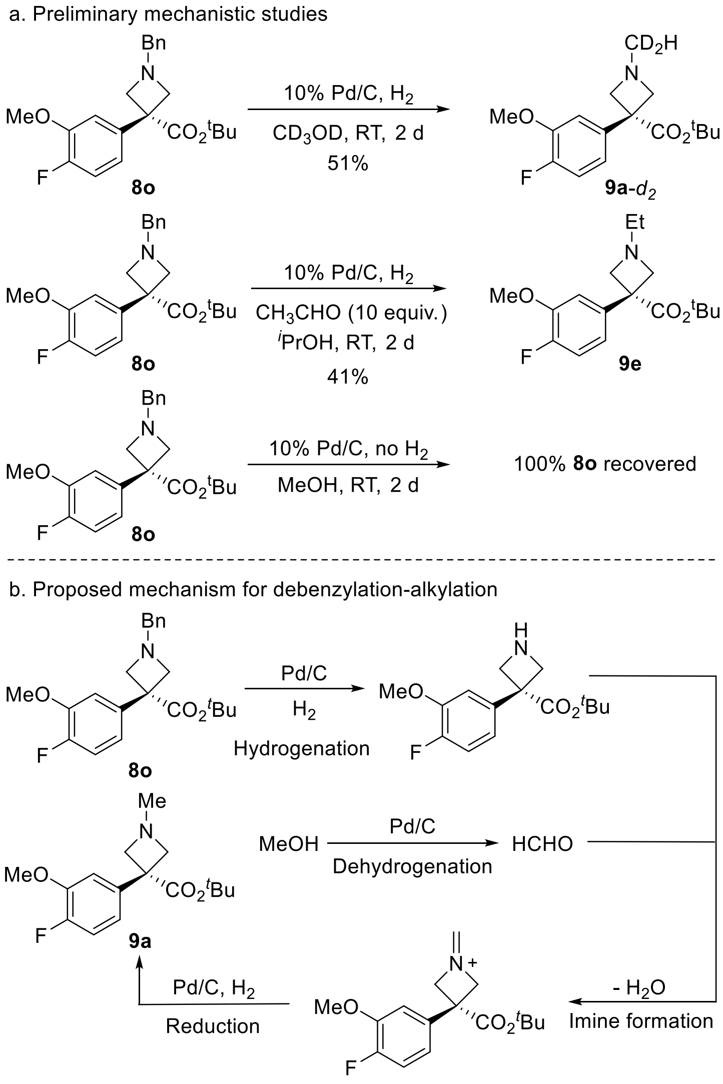

Supplementary Figure 19. Mechanistic studies. **a** Preliminary mechanistic studies. **b** Proposed mechanism. The series of experiments summarized in Supplementary Figure 19 showed that the reaction occurs by formation of an aldehyde from the alcohol solvent and reductive amination with this aldehyde.

The reaction in d_4-MeOH gave 9a-d_2 containing only two deuterium atoms, indicating that the alkyl group on the final product originated from the aldehyde generated from the alcohol. This hypothesis was corroborated by the reaction with acetaldehyde in *i*-propanol, which formed 41% ethylated product 9e rather than the isopropyl products that could be derived from a more direct reaction with the alcohol. Finally, the lack of conversion without added hydrogen showed that the hydrogenolysis did not occur with hydrogen generated from the alcohol.

***tert*-Butyl 3-(4-fluoro-3-methoxyphenyl)-1-(methyl-*d*2)azetidine-3-carboxylate (9a-*d*2)**

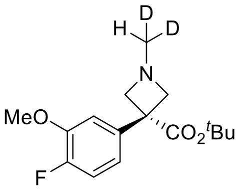

9a-*d*2

Methanol-d_4 as solvent. The reaction solution was filtered through Celite and condensed to afford pure product. Colorless oil, 15 mg, 51% yield. ^{1}H NMR (600 MHz, Chloroform-*d*) δ 7.01 (dd, *J* = 10.9, 8.6 Hz, 1H), 6.85 (d, *J* = 8.0 Hz, 1H), 6.82 – 6.75 (m, 1H), 3.90 (d, *J* = 7.0 Hz, 2H), 3.87 (s, 3H), 3.41 (d, *J* = 7.0 Hz, 2H), 2.28 (s, 1H), 1.38 (s, 9H); ^{13}C NMR (151 MHz, $CDCl_3$) δ 173.02, 151.58 (d, *J* = 245.5 Hz), 147.52 (d, *J* = 10.9 Hz), 137.61 (d, *J* = 3.8 Hz), 118.75 (d, *J* = 6.9 Hz), 115.87 (d, *J* = 18.5 Hz), 111.76, 81.31, 65.15, 56.40, 49.17, 45.72 – 45.19 (m, 1C), 27.91; ^{19}F NMR (376 MHz, $CDCl_3$) δ -136.90; HRMS (ESI): $[M+H]^{\oplus}$ calcd for $C_{16}H_{21}D_2O_3NF^{\oplus}$ 298.1782 found 298.1775.

Derivatizations of arylated *N*-benzyl azetidines

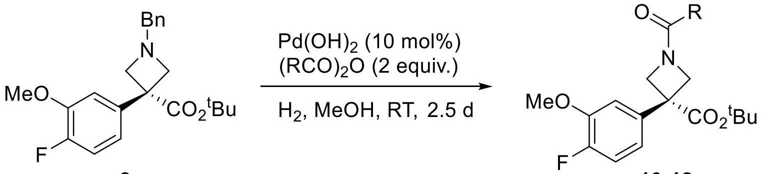

Supplementary Figure 20. Procedure for preparation of **10-12**

To a well-stirred solution of arylated azetidine **8o** (0.1 mmol) in methanol (2 mL) at room temperature was added $Pd(OH)_2$ (20 wt% on carbon, 7 mg, 0.010 mmol) and the anhydride (0.2 mmol). The resulting mixture was allowed to stir under hydrogen (1 atm) for 2.5 days. The reaction solution was filtered through Celite, condensed and purified by flash column chromatography to afford the pure substrate **10-12**.

***tert*-Butyl 1-acetyl-3-(4-fluoro-3-methoxyphenyl)azetidine-3-carboxylate (11)**

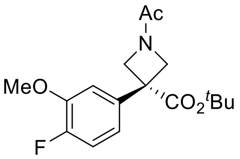

11

Ac_2O (5 equiv) was used. The reaction solution was filtered through Celite and condensed to afford pure product as yellow oil, 26 mg, 81% yield. ^{1}H NMR (400 MHz, Chloroform-*d*) δ 7.07 (dd, *J* = 10.9, 8.5 Hz, 1H), 6.89 – 6.70 (m, 2H), 4.78 (d, *J* = 8.4 Hz, 1H), 4.54 (d, *J* = 10.0 Hz, 1H), 4.43 – 4.26 (m, 2H), 3.88 (s, 3H), 1.90 (s, 3H), 1.39 (s, 9H); ^{13}C NMR (151 MHz, $CDCl_3$) δ 171.76, 170.74, 151.92 (d, *J* = 247.0 Hz), 147.82 (d, *J* = 11.1 Hz), 136.08 (d, *J* = 3.8 Hz), 118.75 (d, *J* = 7.0 Hz), 116.22 (d, *J* = 18.7 Hz), 111.76, 82.63, 59.47, 56.89, 56.48, 47.47, 27.83, 18.89; ^{19}F NMR (376 MHz, $CDCl_3$) δ -135.19; HRMS (ESI): $[M+Na]^{\oplus}$ calcd for $C_{17}H_{22}FNO_4Na^{\oplus}$ 346.1425, found 346.1416.

***tert*-Butyl 3-(4-fluoro-3-methoxyphenyl)-1-hexanoylazetidine-3-carboxylate (12)**

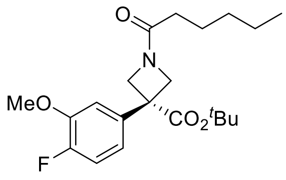

12

Purified by flash column chromatography (hexane/ethyl acetate = 3/1), yellow oil, 31 mg, 85% yield. ^{1}H NMR (400 MHz, Chloroform-*d*) δ 7.07 (dd, *J* = 10.8, 8.4 Hz, 1H), 6.89 – 6.73 (m, 2H), 4.77 (d, *J* = 8.4 Hz, 1H), 4.54 (d, *J* = 9.9 Hz, 1H), 4.33 (dd, *J* = 19.5, 9.1 Hz, 2H), 3.89 (s, 3H), 2.18 – 2.02 (m, 2H), 1.70 – 1.53 (m, 2H), 1.35 – 1.25 (m, 4H), 0.89 (t, *J* = 6.7 Hz, 3H), ^{13}C NMR (151 MHz, $CDCl_3$) δ 173.60, 171.85, 151.91 (d, *J* = 247.1 Hz), 147.81 (d, *J* = 11.0 Hz), 136.23 (d, *J* = 3.8 Hz), 118.76 (d, *J* = 7.0 Hz), 116.19 (d, *J* = 18.7 Hz), 111.79, 82.54, 59.19, 56.82, 56.46, 47.70, 31.65, 31.59, 27.83, 24.52, 22.48, 13.99; ^{19}F NMR (376 MHz, $CDCl_3$) δ -135.31; HRMS (ESI): $[M+Na]^{\oplus}$ calcd for $C_{21}H_{30}FNO_4Na^{\oplus}$ 402.2051, found 402.2051.

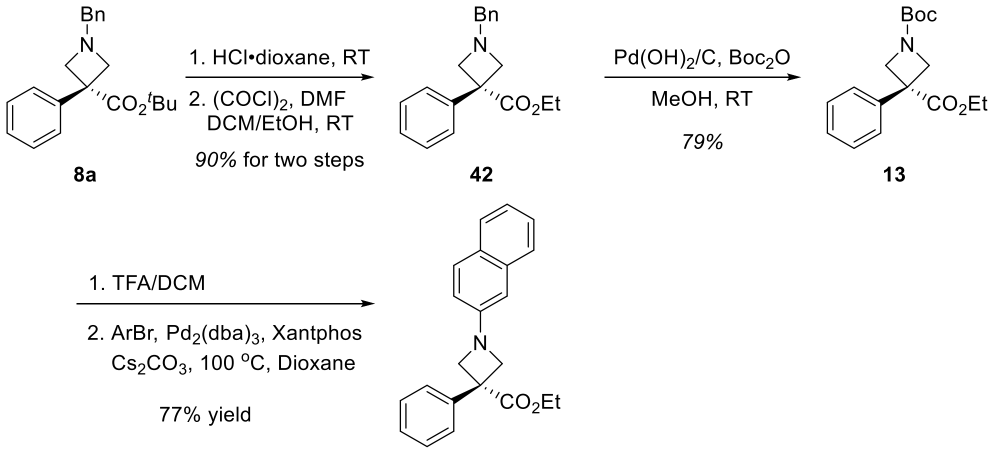

Supplementary Figure 21. Procedure for preparation of **14**

Ethyl 1-benzyl-3-phenylazetidine-3-carboxylate (42): To a 4 ml vial containing arylated azetidine **8a** (22 mg, 0.075 mmol) at room temperature was added HCl (4M in dioxane, 0.3 mL). The resulting mixture was allowed to stir at room temperature for 12 h. Then, the solution was condensed, and the residue was dissolved in DCM (0.3 mL). DMF (5 drops) and oxalyl chloride (12 μL, 2.0 equiv) were added. The reaction was stirred at RT for 1 h, at which time ethanol (0.3 mL) was added, and the resulting mixture was stirred at RT for 12 h. After this time, the reaction was quenched with saturated aqueous $NaHCO_3$ (10 mL). The aqueous layer was extracted with ethyl acetate (10 mL × 3), and the combined organic layers were condensed. The product was purified by flash column chromatography (hexane/ethyl acetate = 4/1) to afford the pure **42** as a colorless oil (18 mg) in 90% yield. ^{1}H NMR (400 MHz, Chloroform-*d*) δ 7.40 – 7.16 (m, 10H), 4.16 (q, *J* = 7.1 Hz, 2H), 4.05 (d, *J* = 7.6 Hz, 2H), 3.66 (s, 2H), 3.52 (d, *J* = 7.6 Hz, 2H), 1.20 (t, *J* = 7.1 Hz, 3H); ^{13}C NMR (101 MHz, $CDCl_3$) δ 174.24, 141.02, 137.82, 128.57, 128.52, 128.38, 127.13, 126.31, (one aromatic carbon signal was not observed because of the overlap of two phenyl groups), 63.45, 61.34, 49.00, 14.05, (the tertiary alkyl carbon signal was not observed); HRMS (ESI): $[M+H]^{\oplus}$ calcd for $C_{19}H_{22}O_2N^{\oplus}$ 296.1645 found 296.1637.

1-(*tert*-Butyl) 3-ethyl 3-phenylazetidine-1,3-dicarboxylate (13): The procedure for the synthesis of **13** from **42** was the same as that described in "Derivatizations of arylated *N*-benzyl azetidine" above. ^{1}H NMR (600 MHz, Chloroform-*d*) δ 7.41 – 7.34 (m, 2H), 7.34 – 7.26 (m, 3H), 4.59 (d, *J* = 8.7 Hz, 2H), 4.29 (d, *J* = 8.6 Hz, 2H), 4.16 (q, *J* = 7.1 Hz, 2H), 1.43 (s, 9H), 1.21 (t, *J* = 7.1 Hz, 3H); ^{13}C NMR (151 MHz,

CDCl3) δ 173.29, 156.16, 139.96, 128.77, 127.66, 126.36, 79.98, 61.79, 58.46, 47.35, 28.41, 14.04; HRMS (ESI): [M+Na]$^{\oplus}$ calcd for $C_{17}H_{23}NO_4Na^{\oplus}$ 328.1519, found 328.1524.

Ethyl 1-(naphthalen-2-yl)-3-phenylazetidine-3-carboxylate (**14**): To a 4 ml vial containing **13** (20 mg, 0.066 mmol) at room temperature was added TFA (0.2 mL) and DCM (0.2 mL). The resulting mixture was allowed to stir at room temperature for 12 h. Then the solution was condensed and dissolved in dioxane (1 mL), followed by the addition of 2-bromonaphthalene (27 mg, 2.0 equiv), cesium carbonate (65 mg, 3.0 equiv), $Pd_2(dba)_3$ (3 mg, 5 mol%) and Xantphos (4.6 mg, 12 mol%). The reaction was stirred at 100 °C for 12 h. After this time, the reaction solution was condensed and purified by flash column chromatography (hexane/ethyl acetate = 15/1) to afford the pure substrate **14** as yellow solid (17 mg) in 77% yield. ^{1}H NMR (600 MHz, Chloroform-*d*) δ 7.71 (dd, *J* = 8.3, 4.8 Hz, 2H), 7.66 (d, *J* = 8.2 Hz, 1H), 7.45 – 7.35 (m, 5H), 7.30 (d, *J* = 6.7 Hz, 1H), 7.24 (t, *J* = 7.4 Hz, 1H), 6.87 (dd, *J* = 8.8, 2.2 Hz, 1H), 6.78 (s, 1H), 4.73 (d, *J* = 7.1 Hz, 2H), 4.31 (d, *J* = 7.1 Hz, 2H), 4.22 (q, *J* = 7.1 Hz, 2H), 1.26 (t, *J* = 7.1 Hz, 3H); ^{13}C NMR (151 MHz, CDCl3) δ 173.71, 149.17, 140.32, 134.58, 129.05, 128.76, 127.84, 127.76, 127.53, 126.50, 126.29, 126.13, 122.62, 114.78, 106.02, 61.73, 61.63, 48.83, 14.13; HRMS (ESI): [M+H]$^{\oplus}$ calcd for $C_{22}H_{22}O_2N^{\oplus}$ 332.1645 found 332.1656.

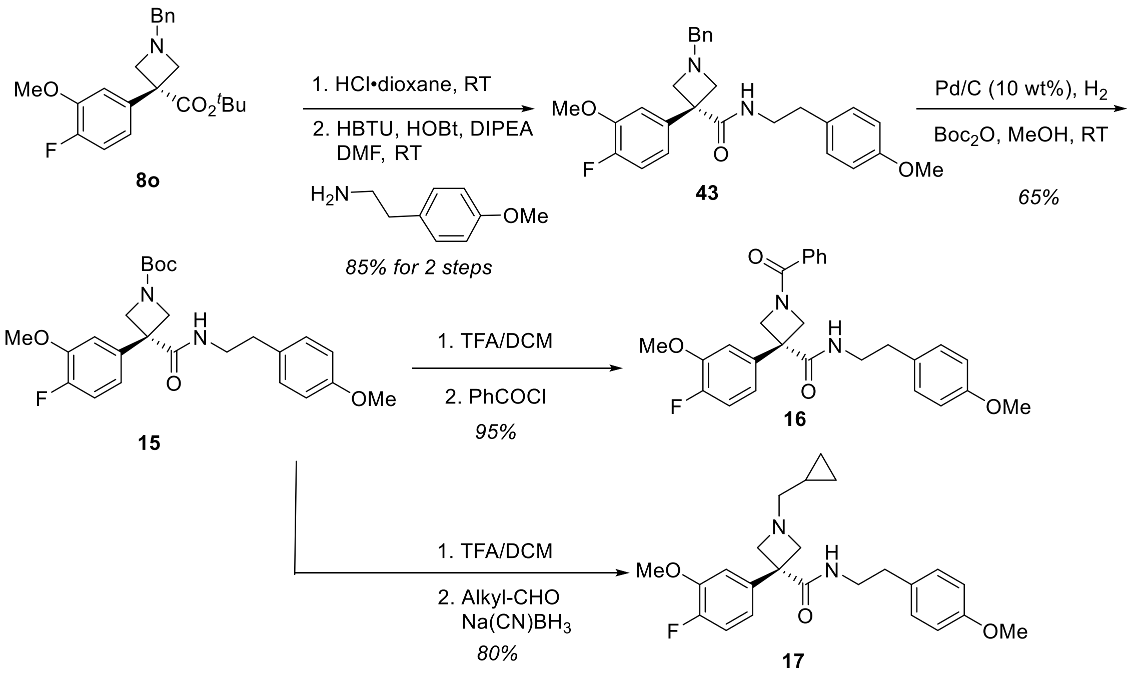

Supplementary Figure 22. Procedure for preparation of **16-17**

1-Benzyl-3-(4-fluoro-3-methoxyphenyl)-*N*-(4-methoxyphenethyl)azetidine-3-carboxamide (**43**): To a 4 ml vial containing arylated azetidine **8o** (36 mg, 0.097 mmol) at room temperature was added HCl (4M in dioxane, 0.4 mL). The resulting mixture was allowed to stir at room temperature for 12 h. Then the solution was condensed and dissolved in DMF (0.5 mL), followed by the addition of 2-(4-methoxyphenyl)ethan-1-amine (23 μL, 1.5 equiv), HBTU (56 mg, 1.5 equiv), HOBt (20 mg, 1.5 equiv) and DIPEA (50 μL, 3.0 equiv), and continued to stir at RT for 12 h. After this time, it was quenched by saturated $NaHCO_3$ aqueous solution (10 mL), extracted with ethyl acetate (10 mL × 3), condensed and purified by flash column chromatography (hexane/ethyl acetate = 2/3) to afford compound **43** as yellow oil (37 mg) in 85% yield. ^{1}H NMR (600 MHz, Chloroform-*d*) δ 7.30 (t, *J* = 7.3 Hz, 2H), 7.28 – 7.21 (m,

3H), 7.01 (dd, J = 10.9, 8.4 Hz, 1H), 6.92 (d, J = 8.4 Hz, 2H), 6.75 (d, J = 8.4 Hz, 2H), 6.72 – 6.62 (m, 2H), 6.06 (s, 1H), 3.83 (d, J = 7.2 Hz, 2H), 3.82 (s, 3H), 3.77 (s, 3H), 3.66 (s, 2H), 3.51 (d, J = 7.1 Hz, 2H), 3.46 (q, J = 6.3 Hz, 2H), 2.68 (t, J = 6.6 Hz, 2H); ^{13}C NMR (151 MHz, $CDCl_3$) δ 173.69, 158.33, 151.60 (d, J = 245.9 Hz), 147.85 (d, J = 10.8 Hz), 138.19 (d, J = 3.7 Hz), 137.59, 130.63, 129.67, 128.61, 128.44, 127.26, 118.91 (d, J = 6.9 Hz), 116.08 (d, J = 18.4 Hz), 114.03, 112.10, 63.15, 63.11, 56.31, 55.27, 49.08, 41.00, 34.51; ^{19}F NMR (376 MHz, $CDCl_3$) δ -136.52; HRMS (ESI): $[M+H]^{\oplus}$ calcd for $C_{27}H_{30}O_3NF^{\oplus}$ 449.2235 found 449.2218.

***tert*-Butyl 3-(4-fluoro-3-methoxyphenyl)-3-((4-methoxyphenethyl)carbamoyl)azetidine-1-carboxylate** (**15**): The procedure for the synthesis of **15** from **43** was the same as that described in "Derivatizations of arylated *N*-benzyl azetidine" above. ^{1}H NMR (600 MHz, Chloroform-*d*) δ 7.09 – 6.99 (m, 1H), 6.82 (d, J = 8.2 Hz, 2H), 6.74 (d, J = 8.3 Hz, 2H), 6.70 – 6.58 (m, 2H), 5.09 (s, 1H), 4.53 (d, J = 60.0 Hz, 2H), 4.14 (s, 2H), 3.81 (s, 3H), 3.79 (s, 3H), 3.47 – 3.36 (m, 2H), 2.62 (t, J = 6.3 Hz, 2H), 1.43 (s, 9H); ^{13}C NMR (151 MHz, $CDCl_3$) δ 172.55, 158.42, 156.15, 151.90 (d, J = 247.7 Hz), 148.32 (d, J = 10.8 Hz), 136.89 (d, J = 3.7 Hz), 130.09, 129.54, 118.90 (d, J = 6.9 Hz), 116.54 (d, J = 18.6 Hz), 114.08, 111.84, 80.00, 56.36, 55.27, 47.34, 41.07, 34.25, 28.43; ^{19}F NMR (376 MHz, $CDCl_3$) δ -134.66; HRMS (ESI): $[M+H]^{\oplus}$ calcd for $C_{25}H_{32}O_5N_2F^{\oplus}$ 459.2290 found 459.2291.

1-Benzoyl-3-(4-fluoro-3-methoxyphenyl)-*N*-(4-methoxyphenethyl)azetidine-3-carboxamide (**16**): To a 4 ml vial containing **15** (30 mg, 0.065 mmol) at room temperature was added TFA (0.3 mL) and DCM (0.3 mL). The resulting mixture was allowed to stir at room temperature for 12 h. Then the solution was condensed and dissolved in DCM (0.5 mL), followed by the addition of triethylamine (108 μL, 10 equiv) and benzoyl chloride (22 μL, 3.0 equiv). The reaction was stirred at RT for 12 h. After this time, the reaction solution was condensed and purified by flash column chromatography (hexane/ethyl acetate = 2/3) to afford product **16** as a colorless oil (29 mg) in 95% yield. ^{1}H NMR (400 MHz, Chloroform-*d*) δ 7.62 (d, J = 7.0 Hz, 2H), 7.52 – 7.32 (m, 3H), 7.04 (dd, J = 10.8, 8.3 Hz, 1H), 6.81 (d, J = 8.5 Hz, 2H), 6.78 – 6.59 (m, 4H), 5.29 (s, 1H), 5.03 (d, J = 7.8 Hz, 1H), 4.66 (d, J = 9.3 Hz, 1H), 4.54 (d, J = 9.5 Hz, 1H), 4.36 (d, J = 8.0 Hz, 1H), 3.79 (s, 3H), 3.77 (s, 3H), 3.51 – 3.33 (m, 2H), 2.62 (t, J = 6.4 Hz, 2H); ^{13}C NMR (101 MHz, $CDCl_3$) δ 172.34, 170.47, 158.36, 151.93 (d, J = 248.7 Hz), 148.38 (d, J = 11.1 Hz), 136.38 (d, J = 3.0 Hz), 132.81, 131.27, 129.99, 129.51, 128.47, 127.97, 118.80 (d, J = 6.9 Hz), 116.61 (d, J = 18.6 Hz), 114.02, 111.71, 62.22, 57.61, 56.32, 55.22, 47.90, 41.09, 34.16; ^{19}F NMR (376 MHz, $CDCl_3$) δ -134.30; HRMS (ESI): $[M+Na]^{\oplus}$ calcd for $C_{27}H_{27}O_4N_2FNa^{\oplus}$ 485.1847 found 485.1843.

1-(Cyclopropylmethyl)-3-(4-fluoro-3-methoxyphenyl)-N-(4-methoxyphenethyl)azetidine-3-carboxamide (**17**): To a 4 ml vial containing **15** (20 mg, 0.044 mmol) was added TFA (0.2 mL) and DCM (0.2 mL) at room temperature. The resulting mixture was allowed to stir at room temperature for 12 h. Then, the solution was condensed and dissolved in methanol (0.4 mL), followed by the addition of cyclopropanecarbaldehyde (9.0 μL, 2.5 equiv), and continued to stir at RT for 0.5 h. Then $Na(CN)BH_3$ (11 mg, 4.0 equiv) was added into above reaction in 3 portions in 0.5 h. The reaction was stirred at RT for two days. After this time, the reaction solution was condensed and purified by flash column chromatography (DCM/methanol = 40/1) to afford product **17** as yellow oil (29 mg) in 80% yield. ^{1}H NMR (400 MHz, Chloroform-*d*) δ 7.02 (dd, J = 11.0, 8.2 Hz, 1H), 6.89 (d, J = 8.5 Hz, 2H), 6.82 – 6.59 (m, 4H), 5.91 (s, 1H), 3.90 (d, J = 7.7 Hz, 2H), 3.82 (s, 3H), 3.78 (s, 3H), 3.70 (d, J = 7.6 Hz, 2H), 3.42 (q, J = 6.4 Hz, 2H), 2.65 (t, J = 6.7 Hz, 2H), 2.44 (d, J = 6.8 Hz, 2H), 0.96 – 0.71 (m, 1H), 0.57 – 0.33 (m, 2H), 0.12 (q, J = 5.0 Hz, 2H); ^{13}C NMR (151 MHz, $CDCl_3$) δ 173.22, 158.30, 151.67 (d, J = 246.6 Hz), 147.93 (d, J = 10.8 Hz), 137.32, 130.43, 129.61, 118.94 (d, J = 7.1 Hz), 116.20 (d, J = 18.6 Hz), 113.97, 112.03, 62.68, 62.53, 56.29, 55.23, 49.26, 41.02, 34.36, 8.31, 2.90; ^{19}F NMR (376 MHz, $CDCl_3$) δ -135.91; HRMS (ESI): $[M+Na]^{\oplus}$ calcd for $C_{24}H_{29}O_3N_2FNa^{\oplus}$ 435.2054 found 435.2044.

Synthesis of biologically relevant molecules

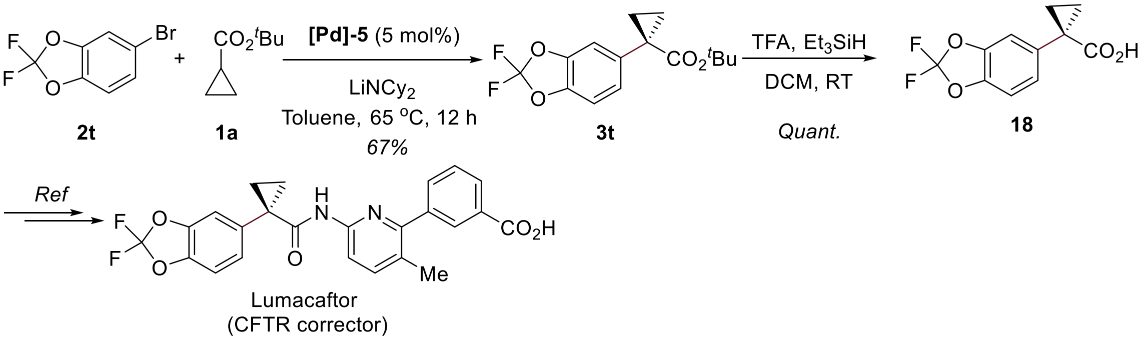

Supplementary Figure 23. Procedure for preparation of Lumacaftor

***tert*-Butyl 1-(2,2-difluorobenzo[*d*][1,3]dioxol-5-yl)cyclopropane-1-carboxylate** (**3t**): This compound was prepared by a synthetic procedure that was the same as that used to prepare **3a** but without $AgBF_4$. It was purified by flash column chromatography (hexane/ethyl acetate = 60/1) to afford **3t** as a colorless oil in 67% yield. ^{1}H NMR (400 MHz, $CDCl_3$) δ 7.06 – 7.03 (m, 2H), 6.95 (d, *J* = 8.1 Hz, 1H), 1.55 – 1.53 (m, 2H), 1.37 (s, 9H), 1.11 – 1.08 (m, 2H); ^{13}C NMR (100 MHz, $CDCl_3$) δ 173.11, 143.40, 142.67, 136.47, 131.76 (t, $J_{C\text{-}F}$ = 254.4 Hz), 125.65, 112.06, 108.79, 81.05, 29.94, 28.00, 16.21; ^{19}F NMR (376 MHz, $CDCl_3$) δ -49.02; HRMS (ESI): [M+Na]$^{\oplus}$ calcd for $C_{15}H_{16}F_2O_4Na^{\oplus}$ 321.0908, found 321.0903.

1-(2,2-Difluorobenzo[d][1,3]dioxol-5-yl)cyclopropane-1-carboxylic acid (**18**): To a 4 mL vial containing **3t** (27 mg, 0.090 mmol) and TFA (0.09 mL) in DCM (0.2 mL) was added Et_3SiH (36 uL, 0.23 mmol) under N_2. The resulting mixture was allowed to stir at RT for 12 h. The reaction solution was condensed directly to afford **18** as a white solid (22 mg) in quantitative yield. ^{1}H NMR (400 MHz, CD_3OD) δ 7.22 (d, *J* = 1.4 Hz, 1H), 7.15 (dd, *J* = 8.3, 1.7 Hz, 1H), 7.09 (d, *J* = 8.3 Hz, 1H), 1.59 (q, *J* = 4.0 Hz, 2H), 1.21 (q, *J* = 4.0 Hz, 2H); ^{13}C NMR (151 MHz, CD_3OD) δ 177.76, 133.08 (t, $J_{C\text{-}F}$ = 252.4 Hz), 144.56, 143.88, 138.16, 127.23, 113.26, 109.94, 29.72, 17.32; ^{19}F NMR (376 MHz, CD_3OD) δ -51.60; HRMS (ESI): [M+Na]$^{\oplus}$ calcd for $C_{11}H_8F_2O_4Na^{\oplus}$ 265.0282, found 265.0281.

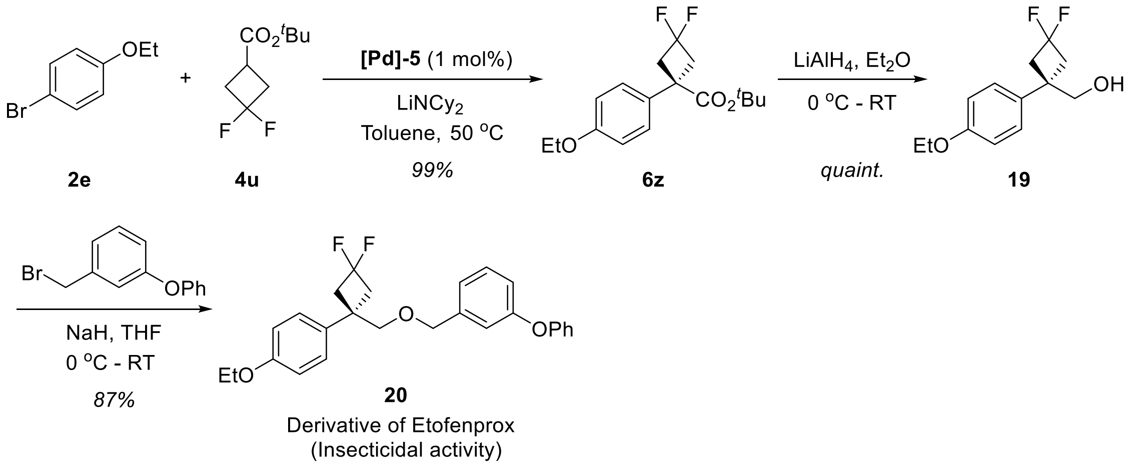

Supplementary Figure 24. Procedure for preparation of **20**

***tert*-Butyl 1-(4-ethoxyphenyl)-3,3-difluorocyclobutane-1-carboxylate** (**6z**): This compound was prepared by a synthetic procedure that was the same as that used to prepare **6c** but without AgOTf. It was purified by flash column chromatography (hexane/ethyl acetate = 60/1) to afford **6z** as a yellow oil in 99% yield. ^{1}H NMR (300 MHz, $CDCl_3$) δ 7.19 (d, *J* = 8.8 Hz, 2H), 6.86 (d, *J* = 8.7 Hz, 2H), 4.02 (q, *J* = 7.0 Hz, 2H), 3.45 – 3.28 (m, 2H), 3.02 – 2.85 (m, 2H), 1.41 (t, *J* = 7.0 Hz, 3H), 1.36 (s, 9H); ^{13}C NMR (100 MHz, $CDCl_3$) δ 172.91, 158.15, 133.26, 127.61, 118.40 (dd, $J_{C\text{-}F}$ = 279.6, 273.8 Hz),114.43, 81.65, 63.52, 45.33 (t, $J_{C\text{-}F}$ = 23.3 Hz), 42.30 (dd, $J_{C\text{-}F}$ = 14.6, 7.8 Hz), 27.75, 14.89; ^{19}F NMR (376 MHz, $CDCl_3$) δ -86.62 – -87.24 (m, 1F), -92.61 – -93.28 (m, 1F); HRMS (ESI): [M+Na]$^{\oplus}$ calcd for $C_{17}H_{22}F_2O_3Na^{\oplus}$ 335.1429, found 335.1415.

(1-(4-Ethoxyphenyl)-3,3-difluorocyclobutyl)methanol (**19**): To a Schlenk tube containing **6z** (31.8 mg, 0.100 mmol) and Et_2O (1 mL) at 0 °C under N_2 was added $LiAlH_4$ (11 mg, 0.30 mmol) in Et_2O (1 mL) dropwise. The resulting solution was stirred at RT for 2 h. After this time, the reaction was quenched by adding ethyl acetate dropwise at 0 °C, followed by saturated, aqueous NH_4Cl (10 mL). The resulting mixture was extracted with ethyl acetate (10 mL × 3) and purified by flash column chromatography (hexane/ethyl acetate = 5/1) to afford **19** (25 mg) as a colorless oil in quantitative yield. ^{1}H NMR (400 MHz, $CDCl_3$) δ 7.08 (d, *J* = 8.6 Hz, 2H), 6.90 (d, *J* = 8.6 Hz, 2H), 4.03 (q, *J* = 7.0 Hz, 2H), 3.70 (s, 2H), 2.91 – 2.73 (m, 4H), 1.42 (t, *J* = 7.0 Hz, 3H); ^{13}C NMR (100 MHz, $CDCl_3$) δ 157.96, 135.68, 127.64, 119.18 (dd, $J_{C\text{-}F}$ = 281.6, 275.3 Hz), 114.73, 70.37, 63.61, 43.36 (t, $J_{C\text{-}F}$ = 22.3 Hz), 37.05 (dd, $J_{C\text{-}F}$ = 13.7, 7.0 Hz), 14.90; ^{19}F NMR (376 MHz, $CDCl_3$) δ -82.56 – -83.19 (m, 1F), -89.40 – -90.07 (m, 1F); HRMS (ESI): [M+H]$^{\oplus}$ calcd for $C_{13}H_{17}F_2O_2^{\oplus}$ 243.1180, found 243.1180.

1-(((1-(4-Ethoxyphenyl)-3,3-difluorocyclobutyl)methoxy)methyl)-3-phenoxybenzene (**20**): To a Schlenk tube containing NaH (60% dispersion in mineral oil, 10 mg, 0.25 mmol) and THF (0.2 mL) at 0 °C under N_2 was added **19** (24 mg, 0.10 mmol) in THF (0.3 mL) dropwise. The resulting solution was stirred at 0 °C for 20 min. Then, 1-(bromomethyl)-3-phenoxybenzene (52 mg, 0.20 mmol) was added to the above solution dropwise. The resulting mixture was allowed to stir at RT for 24 h. After this time, the reaction was quenched with water (10 mL), and the resulting mixture was extracted with ethyl acetate (10 mL × 3). The organic layer was concentrated under vacuum, and the residue purified by flash column chromatography (hexane/ethyl acetate = 40/1) to afford **20** (36 mg) as a colorless oil in 87% yield. ^{1}H NMR (400 MHz, $CDCl_3$) δ 7.38 – 7.31 (m, 2H), 7.27 - 7.24 (m, 1H), 7.15 – 7.05 (m, 3H), 7.03 – 6.97 (m, 2H), 6.93 – 6.89 (m, 2H), 6.87 – 6.80 (m, 3H), 4.42 (s, 2H), 4.01 (q, *J* = 7.0 Hz, 2H), 3.52 (d, *J* = 1.3 Hz, 2H), 3.01 – 2.72 (m, 4H), 1.41 (t, *J* = 7.0 Hz, 3H); ^{13}C NMR (151 MHz, $CDCl_3$) δ 157.65, 157.48, 157.21, 140.44, 136.61, 129.82, 129.68, 127.71, 123.37, 121.96, 119.23 (t, $J_{C\text{-}F}$ = 279.4 Hz), 119.06, 117.92, 117.53, 114.27, 77.23, 72.70, 63.50, 43.57 (dd, $J_{C\text{-}F}$ = 22.3, 22.3 Hz), 35.94 (dd, $J_{C\text{-}F}$ = 12.6, 8.8 Hz), 14.93; ^{19}F NMR (376 MHz, $CDCl_3$) δ -83.99 – -84.63 (m, 1F) , -88.76 – -89.42 (m, 1F); HRMS (ESI): [M+Na]$^{\oplus}$ calcd for $C_{26}H_{26}F_2O_3Na^{\oplus}$ 447.1742, found 447.1745.

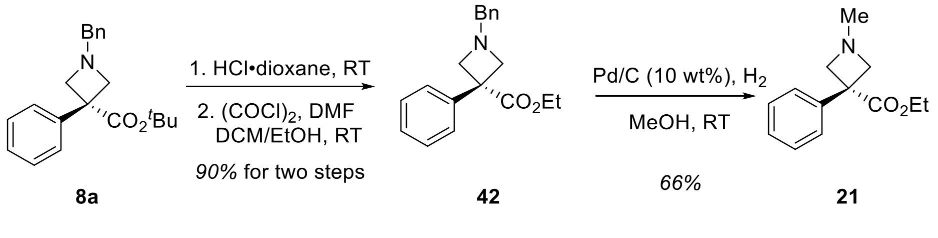

Supplementary Figure 25. Procedure for preparation of **21**

Ethyl 1-methyl-3-phenylazetidine-3-carboxylate (**21**): The procedure used for the synthesis of **21** from **42** was the same as that described for the synthesis of **9a**. ^{1}H NMR (600 MHz, Chloroform-*d*) δ 7.33 (t, *J* = 6.5 Hz, 2H), 7.30 – 7.20 (m, 3H), 4.18 – 3.68 (m, 4H), 3.45 (d, *J* = 6.1 Hz, 2H), 2.34 (s, 3H), 1.37 – 0.98 (m, 3H); ^{13}C NMR (101 MHz, $CDCl_3$) δ 174.14, 140.73, 128.61, 127.17, 126.26, 65.34, 61.42, 48.83, 45.91, 14.07; HRMS (ESI): [M+H]$^⊕$ calcd for $C_{13}H_{18}NO_2^{⊕}$ 220.1332, found 220.1328.

Competition experiments and H/D exchange studies

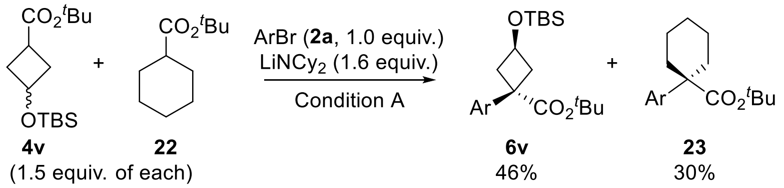

Supplementary Figure 26. Competition reaction between **4v** and **22**

Competition reaction between **4v** and **22** as a representative example for condition A: In a dry and N_2-filled glovebox, small ring esters **4v** (43 mg, 0.15 mmol) and **22** (28 mg, 0.15 mmol) in toluene (0.3 mL) were added dropwise at room temperature to a 4 mL vial containing solid $LiNCy_2$ (30 mg, 0.16 mmol) while stirring. The resulting mixture was allowed to stir for another 15 min at RT. Then, the above solution was transferred to a second vial containing **[Pd]-5** (2.6 mg, 0.0050 mmol), $AgBF_4$ (1.0 mg, 0.0050 mmol), 4-bromo-1-fluoro-2-methoxybenzene **2a** (20 mg, 0.10 mmol) and toluene (0.1 mL). The vial was sealed with a PTFE lined cap, removed from the dry box and stirred at 50 °C for 12 h. An ^{19}F NMR spectrum of the crude reaction was obtained with fluorobenzene as internal standard to determine the yield of each product.

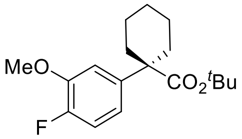

23

Purified by flash column chromatography (hexane/ethyl acetate = 60/1), white solid. ^{1}H NMR (500 MHz, $CDCl_3$) δ 7.02 – 7.01 (m, 2H), 6.92 – 6.89 (m, 1H), 3.86 (s, 3H), 2.40 (d, *J* = 12.6 Hz, 2H), 1.72 – 1.55 (m, 5H), 1.53 – 1.44 (m, 2H), 1.37 (s, 9H), 1.28 – 1.20 (m, 1H); ^{13}C NMR (151 MHz, $CDCl_3$) δ 174.00, 151.18 (d, J_{C-F} = 244.1 Hz), 147.15 (d, J_{C-F} = 10.7 Hz), 141.02 (d, J_{C-F} = 3.1 Hz), 118.18 (d, J_{C-F} = 6.6 Hz), 115.54 (d, J_{C-F} = 18.0 Hz), 111.56 (d, J_{C-F} = 1.3 Hz), 80.42, 56.24, 51.07, 34.95, 27.86, 25.62, 23.77; ^{19}F NMR (376 MHz, $CDCl_3$) δ -137.91; HRMS (ESI): [M+Na]$^⊕$ calcd for $C_{18}H_{25}FO_3Na^{⊕}$ 331.1679, found 331.1683.

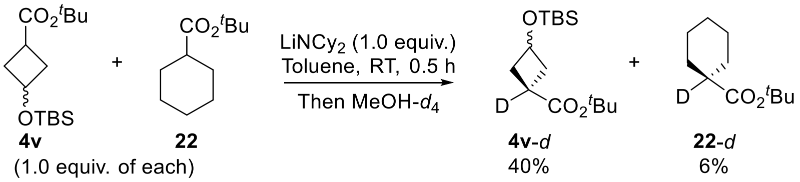

Supplementary Figure 27. H/D exchange studies between **4v** and **22**

Representative procedure for H/D exchange studies: In a dry and N_2-filled glovebox, small ring esters **4v** (29 mg, 0.10 mmol) and **22** (18 mg, 0.10 mmol) in toluene (0.3 mL) were added dropwise at room temperature to a 4 mL vial containing solid $LiNCy_2$ (19 mg, 0.10 mmol) while stirring. The resulting mixture was allowed to stir for another 30 min at RT. Then, MeOH- d_4 (0.2 mL) was added to quench the reaction. The reaction solution was concentrated and the residue was purified by flash column

chromatography to recover the two esters. A ^{1}H NMR spectrum of the esters was obtained to determine the amount of remaining protons remaining in the α position.

Studies on the relative rates for transmetallation

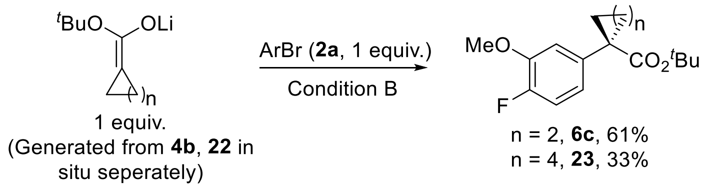

Supplementary Figure 28. Condition B for the competition reaction

Competition experiments between **4b** and **22** in as a representative example for condition B: In a dry and N_2-filled glovebox, cyclobutyl ester **4b** (16 mg, 0.10 mmol) in toluene (0.3 mL) was added dropwise at room temperature to a 4 mL vial containing solid $LiNCy_2$ (19 mg, 0.10 mmol) while stirring. Meanwhile, cyclohexyl ester **22** (18 mg, 0.10 mmol) in toluene (0.3 mL) was added dropwise at room temperature to another 4 mL vial containing solid $LiNCy_2$ (19 mg, 0.10 mmol) while stirring. Both of these two reactions were stirred at RT for 15 min. Then, these two enolates formed in situ were added simultaneously to the third 4 mL vial containing **[Pd]-5** (2.6 mg, 0.0050 mmol), $AgBF_4$ (1.0 mg, 0.0050 mmol), 4-bromo-1-fluoro-2-methoxybenzene **2a** (20 mg, 0.10 mmol) and toluene (0.1 mL). The vial was sealed with a PTFE lined cap, removed from the dry box and stirred at 65 ^{o}C for 6 h. An ^{19}F NMR spectrum of the crude reaction was obtained with fluorobenzene as internal standard to determine the yield of each product.

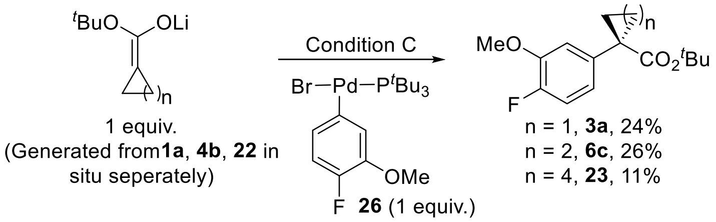

Supplementary Figure 29. Condition C for the competition reaction

In a dry and N_2-filled glovebox, cyclopropyl ester **1a** (14 mg, 0.10 mmol) in toluene (0.3 mL) was added dropwise at room temperature to a 4 mL vial containing solid $LiNCy_2$ (19 mg, 0.10 mmol) while stirring. Meanwhile, the enolates from cyclobutyl ester **4b** and cyclohexyl ester **22** were prepared separately in situ by the same procedure for the generation of enolate from cyclopropyl etser **1a**. All of these three reactions were stirred at RT for 15 min. Then, these three enolates formed in situ were added simultaneously to the fourth 4 mL vial containing palladium complex **26** (51 mg, 0.10 mmol) and toluene (0.1 mL). The vial was sealed with a PTFE lined cap, removed from the dry box and stirred at 65 ^{o}C for 6 h. An ^{19}F NMR spectrum of the crude reaction was obtained with fluorobenzene as internal standard to determine the yield of each product.

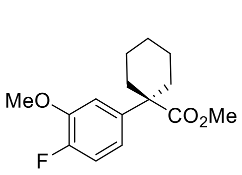

25

Colorless oil. ^{1}H NMR (400 MHz, Chloroform-*d*) δ 7.06 – 6.95 (m, 2H), 6.91 (ddd, *J* = 8.5, 4.3, 2.4 Hz, 1H), 3.88 (s, 3H), 3.64 (s, 3H), 2.55 – 2.39 (m, 2H), 1.77 – 1.58 (m, 5H), 1.52 – 1.38 (m, 2H), 1.26 (qt, *J* = 12.9, 3.4 Hz, 1H); ^{13}C NMR (151 MHz, $CDCl_3$) δ 175.52, 151.35 (d, *J* = 245.1 Hz), 147.33 (d, *J* = 10.6 Hz), 140.25 (d, *J* = 2.1 Hz), 118.33 (d, *J* = 6.6 Hz), 115.72 (d, *J* = 18.1 Hz), 111.65, 56.31, 52.14, 50.60, 34.88, 25.49, 23.70; ^{19}F NMR (376 MHz, $CDCl_3$) δ -137.28; HRMS (ESI): [M+Na]$^{\oplus}$ calcd for $C_{15}H_{19}FO_3Na^{\oplus}$ 289.1210, found 289.1213.

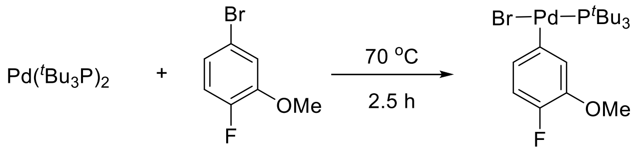

Supplementary Figure 30. Procedure for preparation of **26**

In a dry and N_2-filled glovebox, to a 4 mL vial containing Pd(^{t}Bu$_3$P)$_2$ (200 mg, 0.392 mmol) was added arylbromide **2a** (1.5 mL). The mixture was stirred at 70 °C for 2.5 h. After this time, anhydrous pentane (40 mL) was added to above reaction solution under N_2 and the resulting mixture was stirred at RT for 2 h and then filtered to collect the complex **26** as yellow solid (80 mg) in 40% yield. ^{1}H NMR (600 MHz, Chloroform-*d*) δ 6.91 (d, *J* = 8.3 Hz, 1H), 6.85 – 6.75 (m, 1H), 6.70 (dd, *J* = 11.2, 9.0 Hz, 1H), 3.83 (s, 3H), 1.47 (d, *J* = 12.7 Hz, 27H); ^{13}C NMR (151 MHz, Chloroform-*d*) δ 150.41 (d, *J* = 241.8 Hz), 145.23 (dd, *J* = 10.5, 3.1 Hz), 127.30 (t, *J* = 4.9 Hz), 123.85, 120.28 (d, *J* = 3.3 Hz), 113.83 (dd, *J* = 17.8, 3.2 Hz), 56.52, 40.82 (d, $J_{P\text{-}C}$ = 10.1 Hz), 32.01 (d, $J_{P\text{-}C}$ = 2.7 Hz); ^{19}F NMR (376 MHz, $CDCl_3$) δ -141.80; ^{31}P NMR (162 MHz, $CDCl_3$) δ 61.60; HRMS (ESI): [M-Br]$^{\oplus}$ calcd for $C_{19}H_{33}FPOPd^{\oplus}$ 433.1282, found 433.1283.

Studies on Claisen condensation of a series of esters

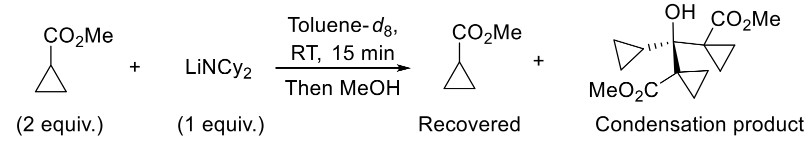

Supplementary Figure 31. Studies on the Claisen condensation for subtrate **1t**

Studies on the Claisen condensation for substrate **1t** as a representative example: In a dry and N_2-filled glovebox, cyclopropyl methyl ester **1t** (20 mg, 0.20 mmol) in toluene-d_8 (0.3 mL) was added dropwise at room temperature to a 4 mL vial containing solid $LiNCy_2$ (19 mg, 0.10 mmol) while stirring. The resulting mixture was allowed to stir for another 15 min at RT. Then, MeOH (9 μL, 0.3 mmol) was added to quench the reaction. A ^{1}H NMR spectrum of the crude reaction was obtained to determine the amount of remaining cyclopropyl methyl ester **1t**. Then, the crude reaction solution was condensed and purified by flash column chromatography (hexane/ethyl acetate = 20/1) to afford condensation product **27** as a colorless oil (5.9 mg) in 33% yield based on the cyclopropyl methyl ester used.

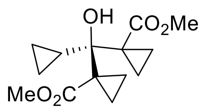

27

It was purified by flash column chromatography (hexane/ethyl acetate = 20/1), colorless oil. ^{1}H NMR (600 MHz, Chloroform-*d*) δ 4.06 (s, 1H), 3.64 (s, 6H), 1.38 – 1.24 (m, 7H), 1.18 (ddd, *J* = 11.0, 6.6, 4.3 Hz, 2H), 0.92 (ddd, *J* = 10.3, 6.8, 4.0 Hz, 2H), 0.52 – 0.43 (m, 2H), 0.30 – 0.22 (m, 2H); ^{13}C NMR (151 MHz, $CDCl_3$) δ 174.88, 69.88, 51.86, 32.63, 14.19, 11.48 (Me-), 11.44 (Me-), -0.57; HRMS (ESI): $[M+H]^{\oplus}$ calcd for $C_{14}H_{21}O_5^{\oplus}$ 269.1384, found 269.1379.

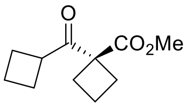

28

It was purified by flash column chromatography (hexane/ethyl acetate = 40/1), colorless oil, 9 mg, 46% yield. ^{1}H NMR (600 MHz, Chloroform-*d*) δ 3.70 (s, 3H), 3.41 – 3.32 (m, 1H), 2.50 – 2.41 (m, 4H), 2.33 – 2.22 (m, 2H), 2.06 – 1.90 (m, 4H), 1.87 – 1.78 (m, 2H); ^{13}C NMR (151 MHz, $CDCl_3$) δ 206.50, 173.06, 58.17, 52.40, 42.11, 27.79, 25.72, 18.21, 15.78; HRMS (ESI): $[M+H]^{\oplus}$ calcd for $C_{11}H_{17}O_3^{\oplus}$ 197.1172, found 197.1173.

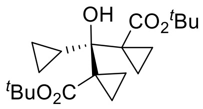

29

It was purified by flash column chromatography (hexane/ethyl acetate = 60/1), colorless oil, 12 mg, 50% yield. ^{1}H NMR (600 MHz, Chloroform-*d*) δ 4.35 (s, 1H), 1.44 (s, 18H), 1.31 – 1.16 (m, 5H), 1.10 – 0.99 (m, 2H), 0.88 – 0.78 (m, 2H), 0.48 – 0.39 (m, 2H), 0.27 – 0.16 (m, 2H); ^{13}C NMR (151 MHz, $CDCl_3$) δ 173.61, 80.76, 69.87, 33.62, 28.03, 14.33, 11.26, 11.09, -0.71; HRMS (ESI): $[M+H]^{\oplus}$ calcd for $C_{20}H_{33}O_5^{\oplus}$ 353.2323, found 353.2319.

Studies on the stability of the enolates of azetidine esters

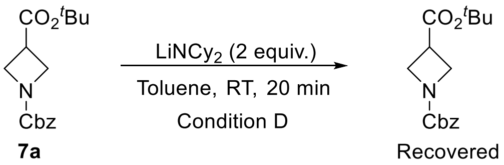

Supplementary Figure 32. Condition D for the studies on the stability of **7a**

Condition D (the studies on the stability of **7a** with $LiNCy_2$ as the base as a representative example): In a dry and N_2-filled glovebox, Cbz-protected azetidine ester **7a** (29 mg, 0.10 mmol) in toluene (0.3 mL) was added droepwise at room temperature to a 4 mL vial containing solid $LiNCy_2$ (37 mg, 0.20 mmol) while stirring. The resulting mixture was allowed to stir for another 20 min at RT. Then, MeOH (0.1 mL) was added to quench the reaction. The crude reaction solution was condensed and a ^{1}H NMR spectrum of the crude reaction with dibromomethane as internal standard was obtained to determine the amount of remaining **7a**.

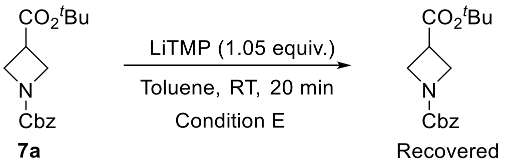

Supplementary Figure 33. Condition E for the studies on the stability of **7a**

Condition E (the studies on the stability of **7a** with LiTMP as the base as a representative example): In a dry and N_2-filled glovebox, Cbz-protected azetidine ester **7a** (29 mg, 0.10 mmol) in toluene (0.3 mL) was added dropwise at room temperature to a 4 mL vial containing solid LiTMP (15.5 mg, 0.0105 mmol) while stirring. The resulting mixture was allowed to stir for another 20 min at RT. Then, MeOH (0.1 mL) was added to quench the reaction. The crude reaction solution was condensed and a ^{1}H NMR spectrum of the crude reaction with dibromomethane as internal standard was obtained to determine the amount of remaining **7a**.

Copies of NMR spectra of all new compounds

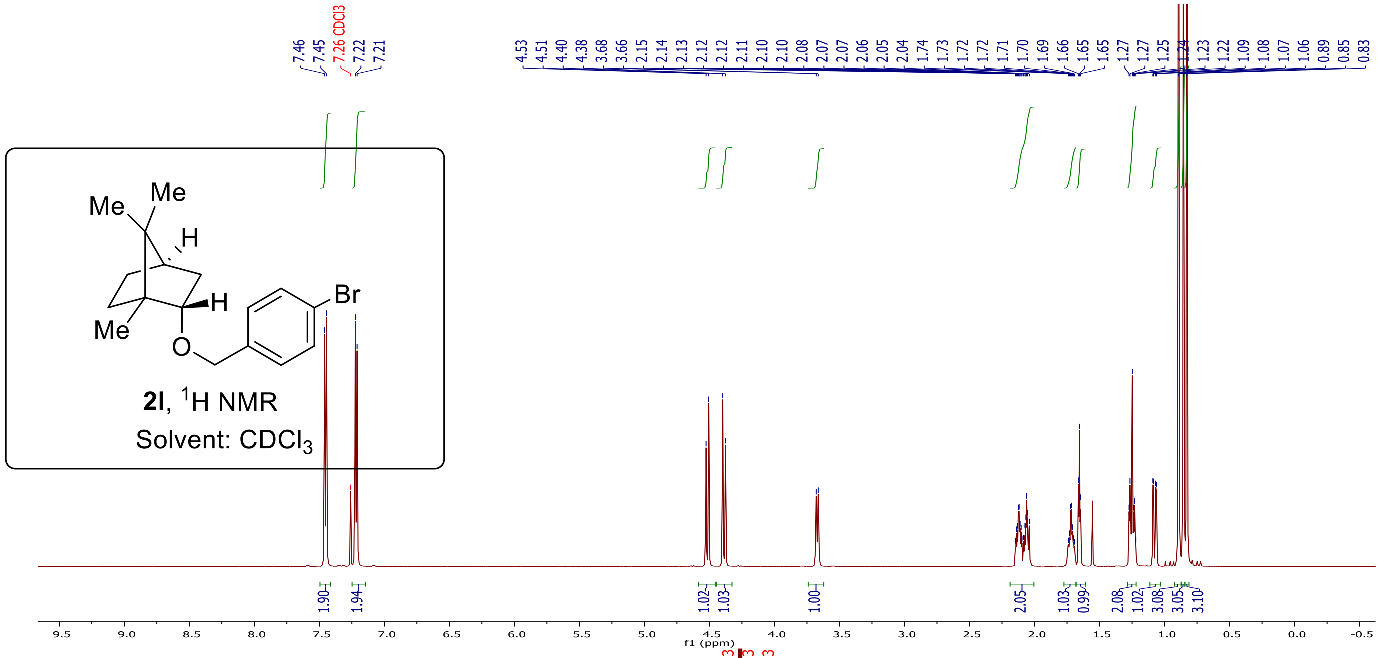

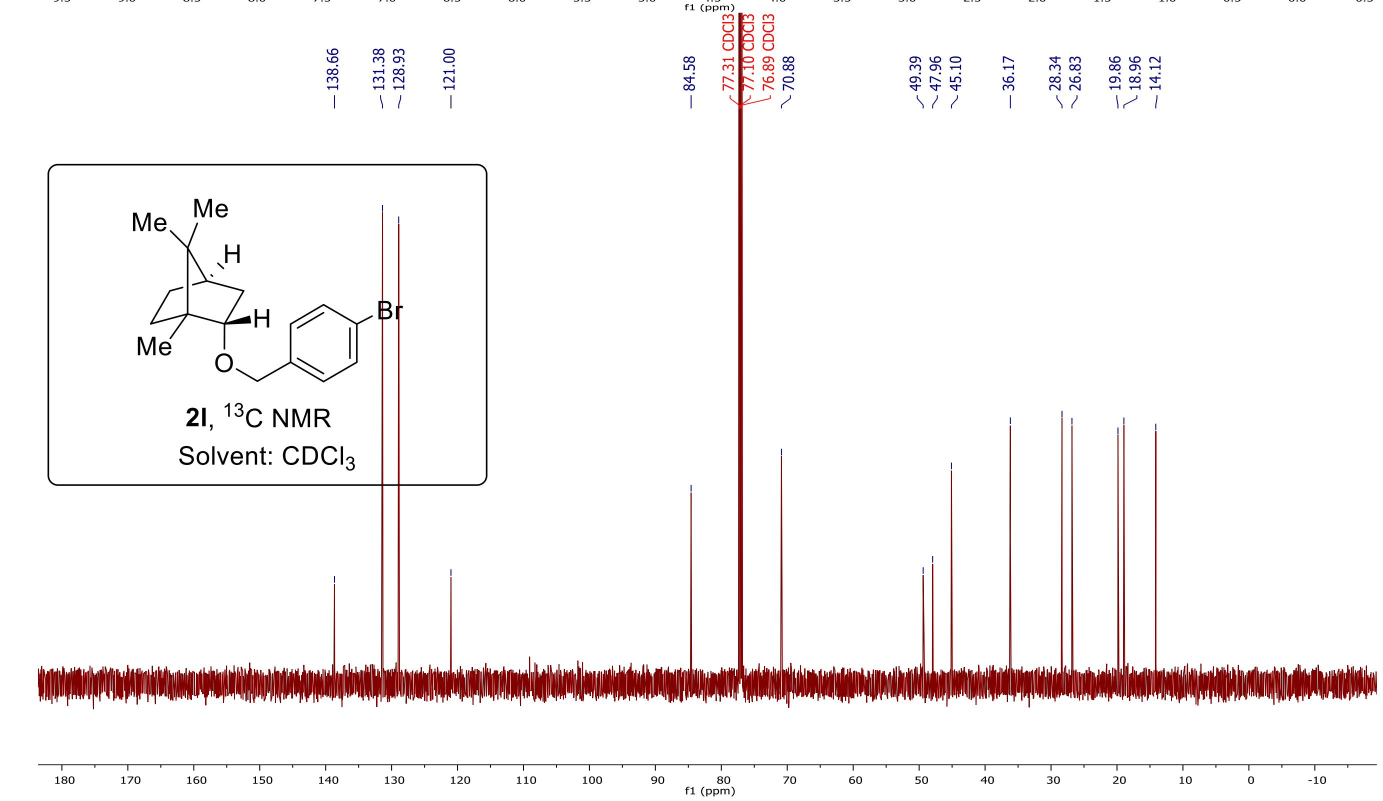

Supplementary Figure 34. NMR spectra of **2l**

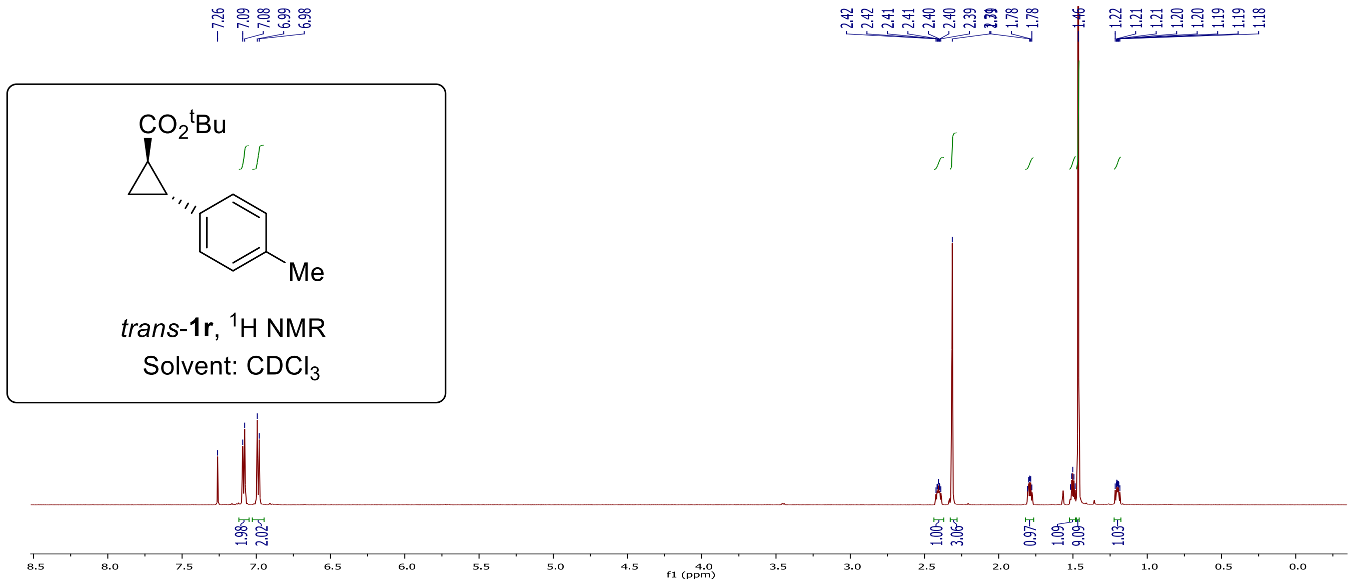

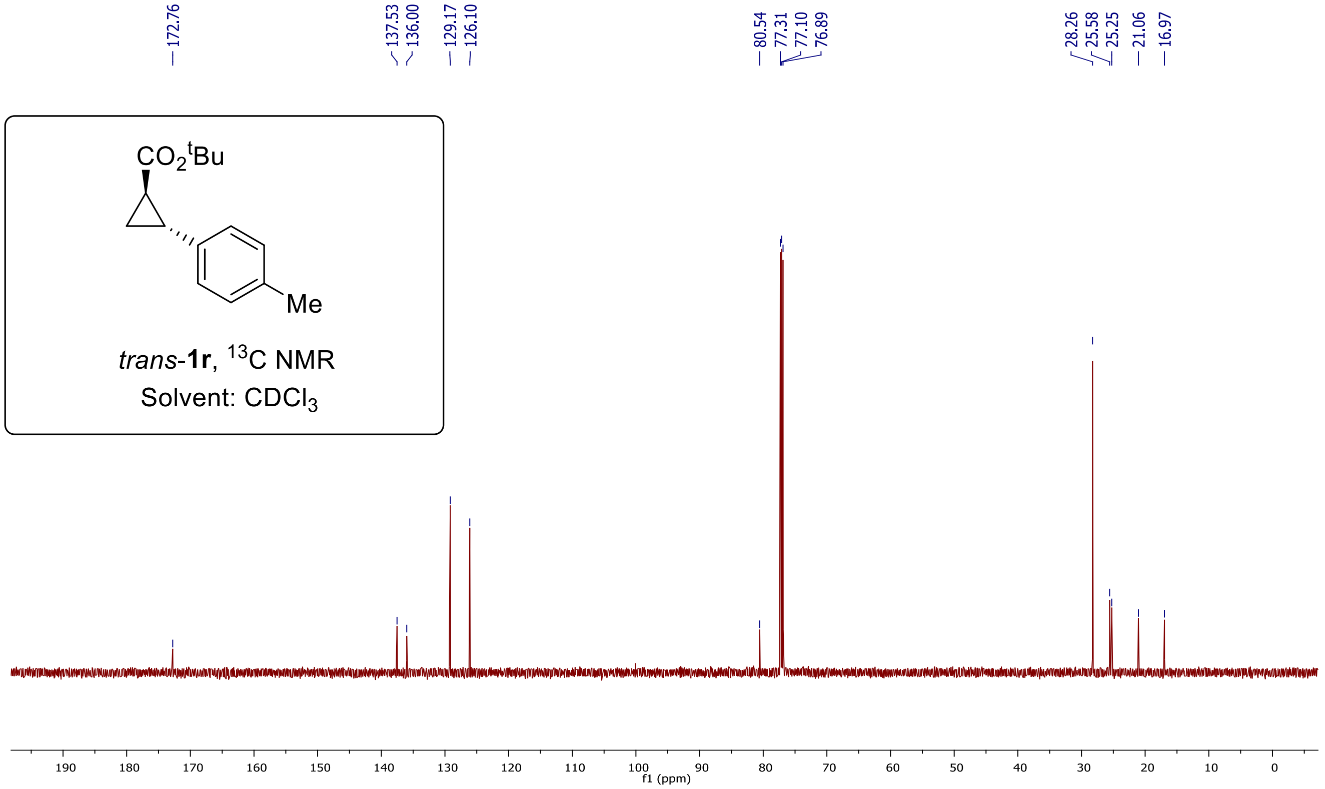

Supplementary Figure 35. NMR spectra of *trans*-**1r**

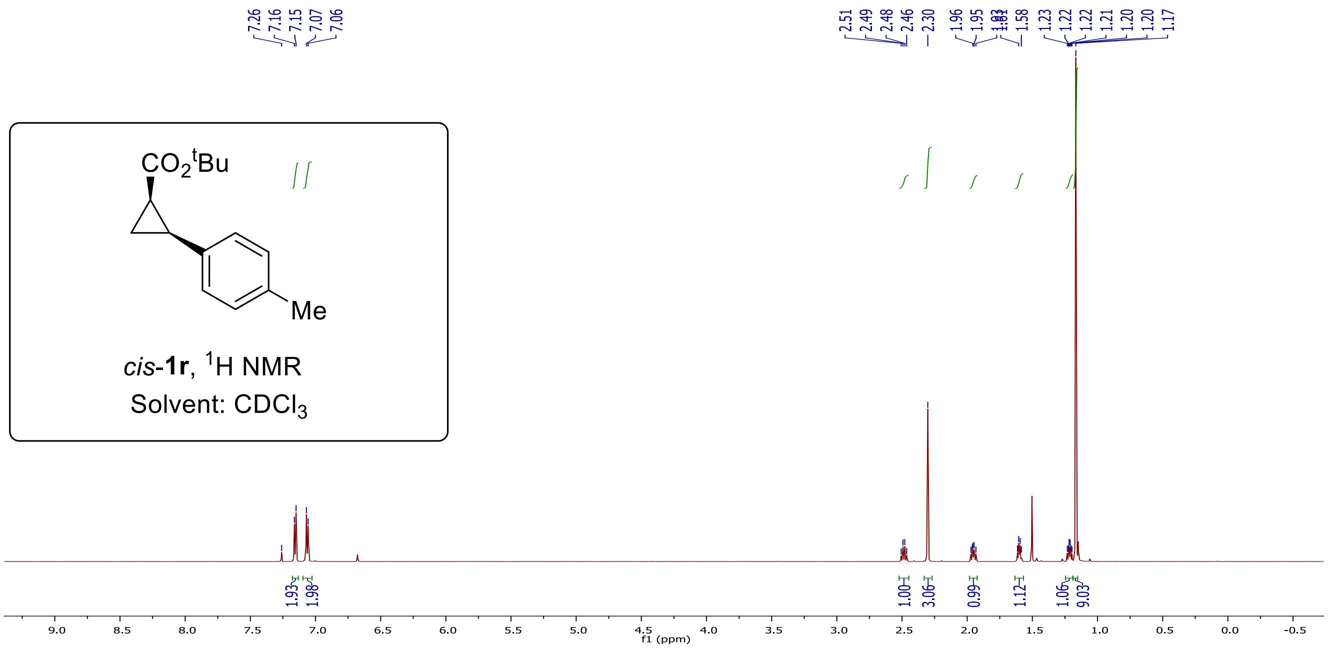

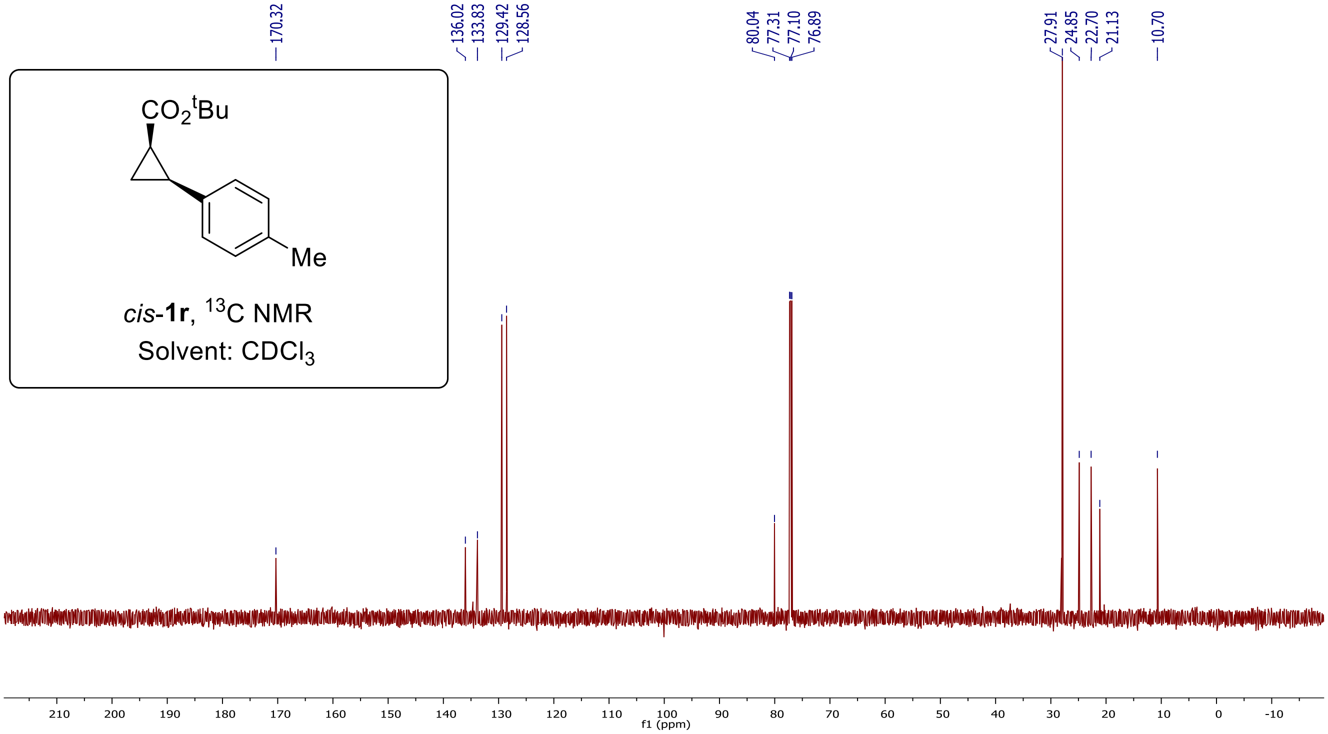

Supplementary Figure 36. NMR spectra of *cis*-**1r**

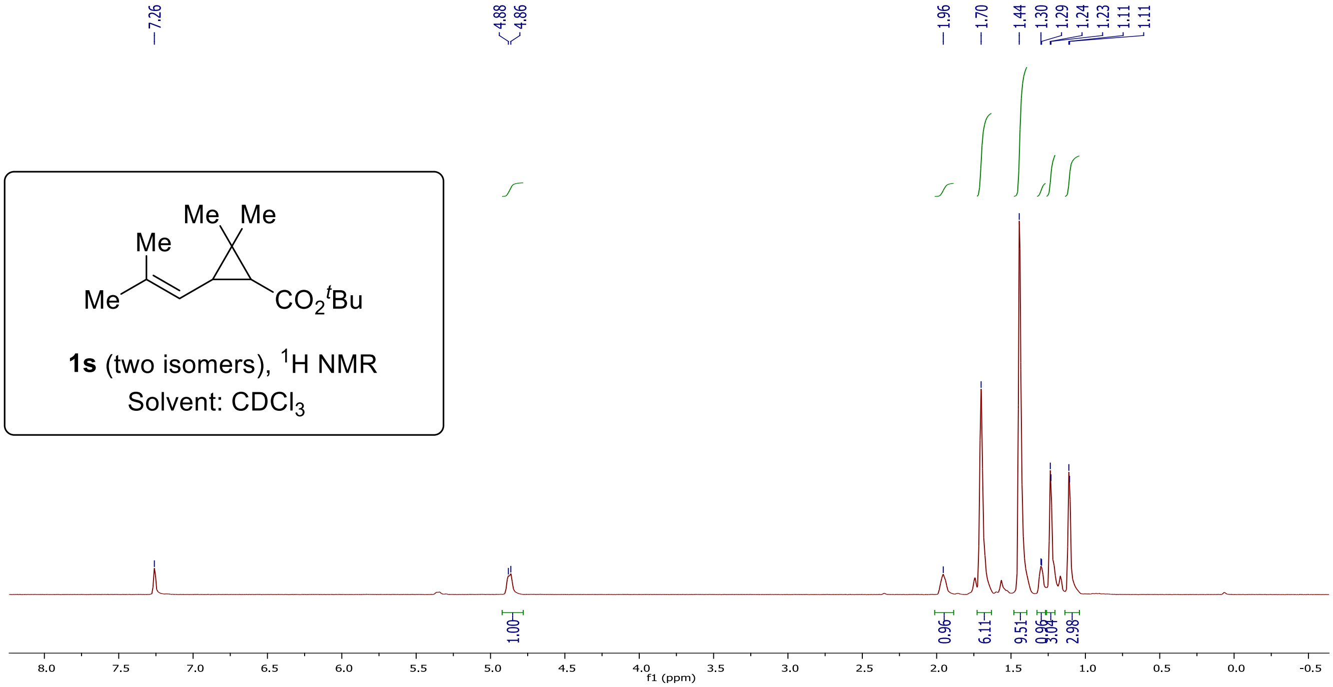

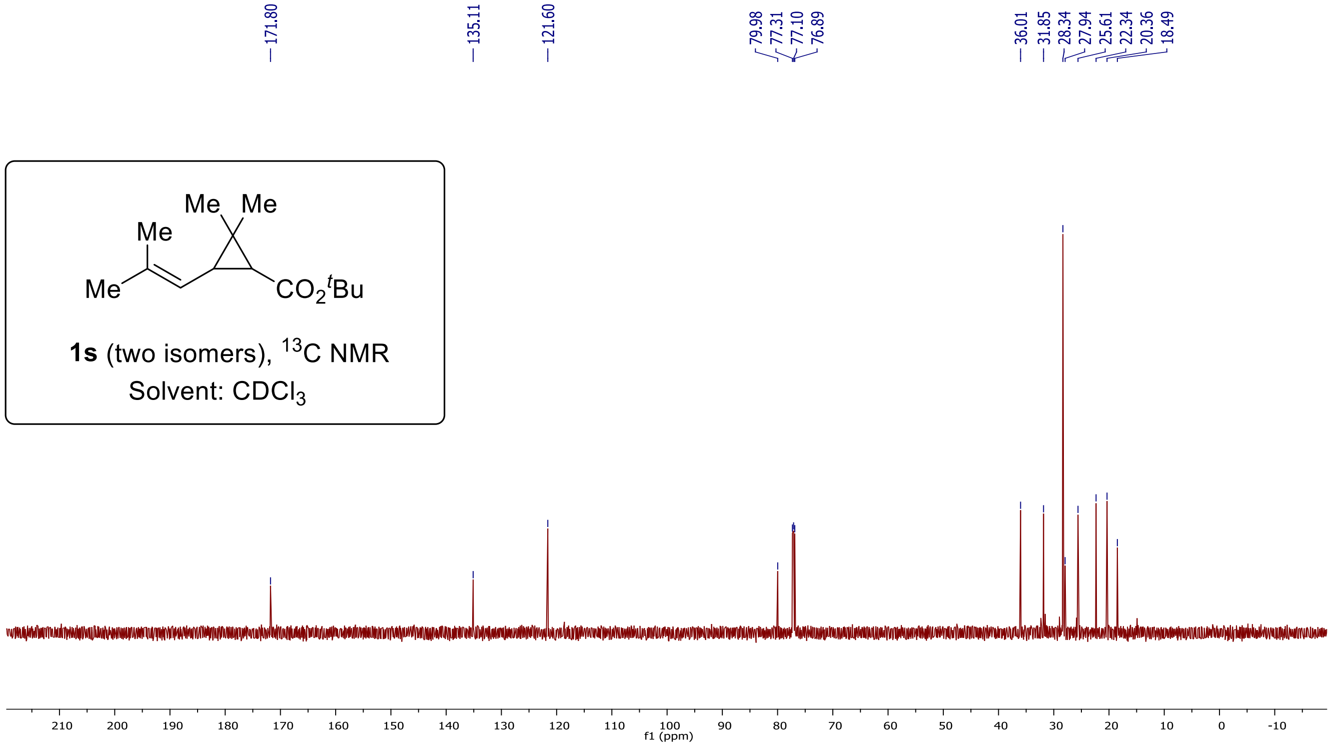

Supplementary Figure 37. NMR spectra of **1s**

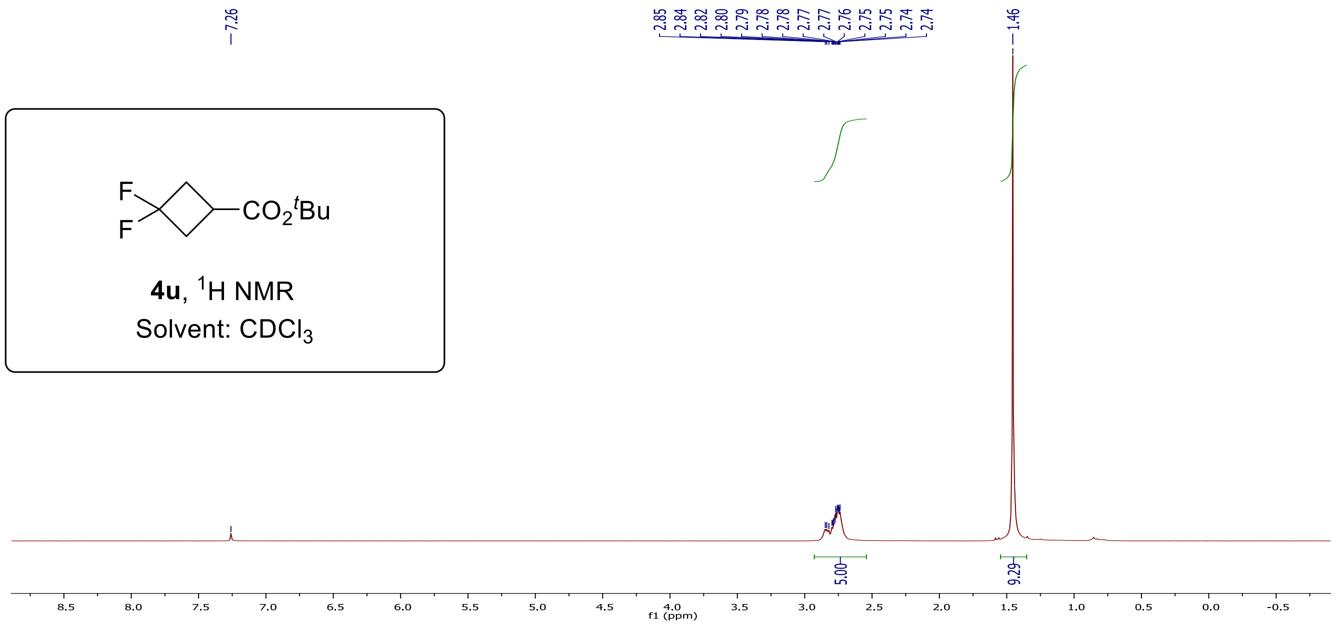

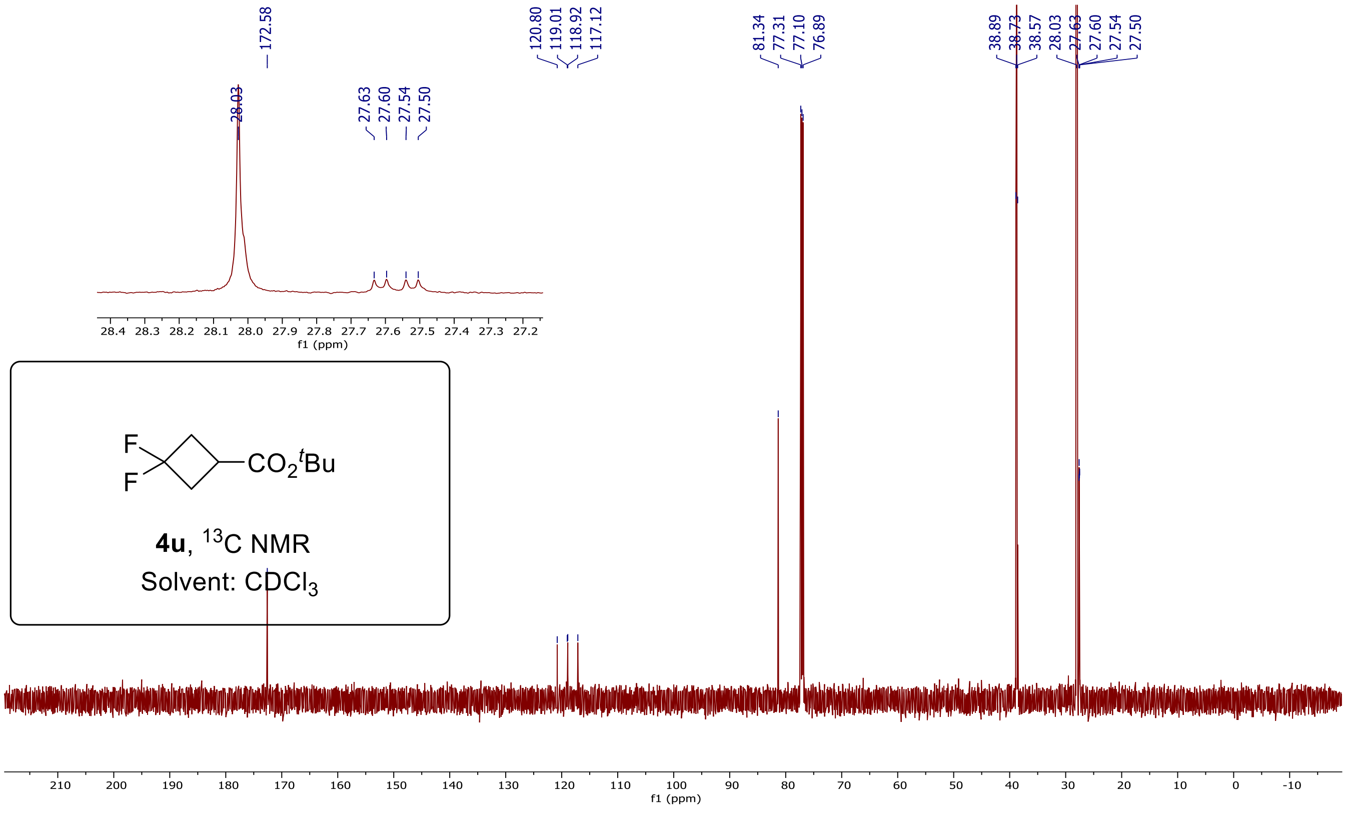

Supplementary Figure 38. NMR spectra of **4u**

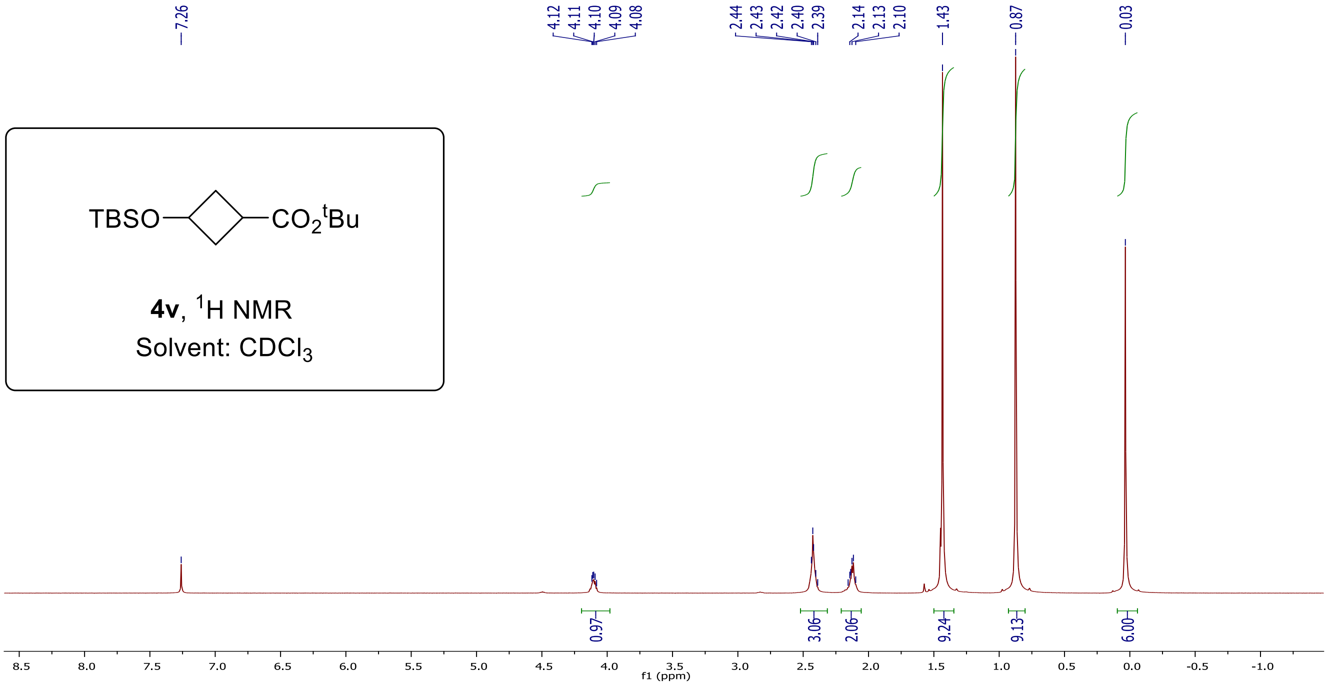

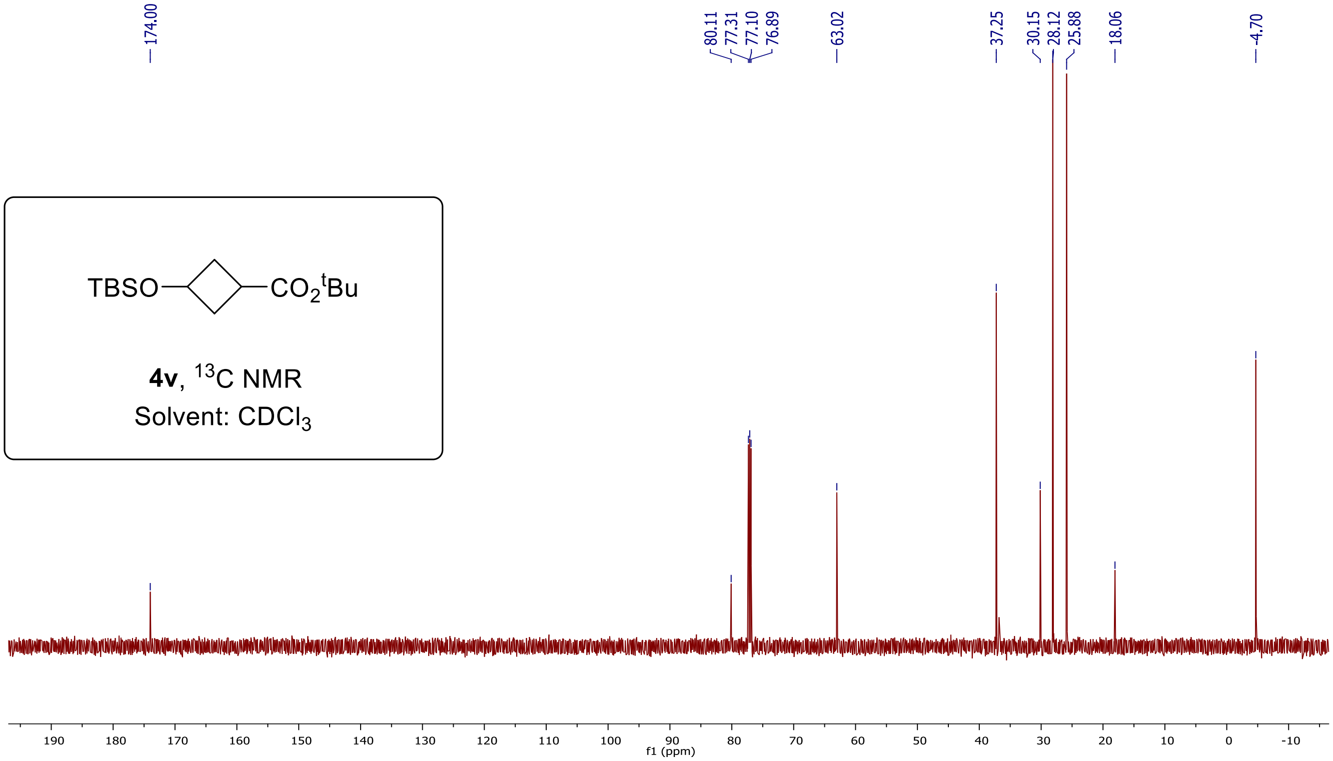

Supplementary Figure 39. NMR spectra of **4v**

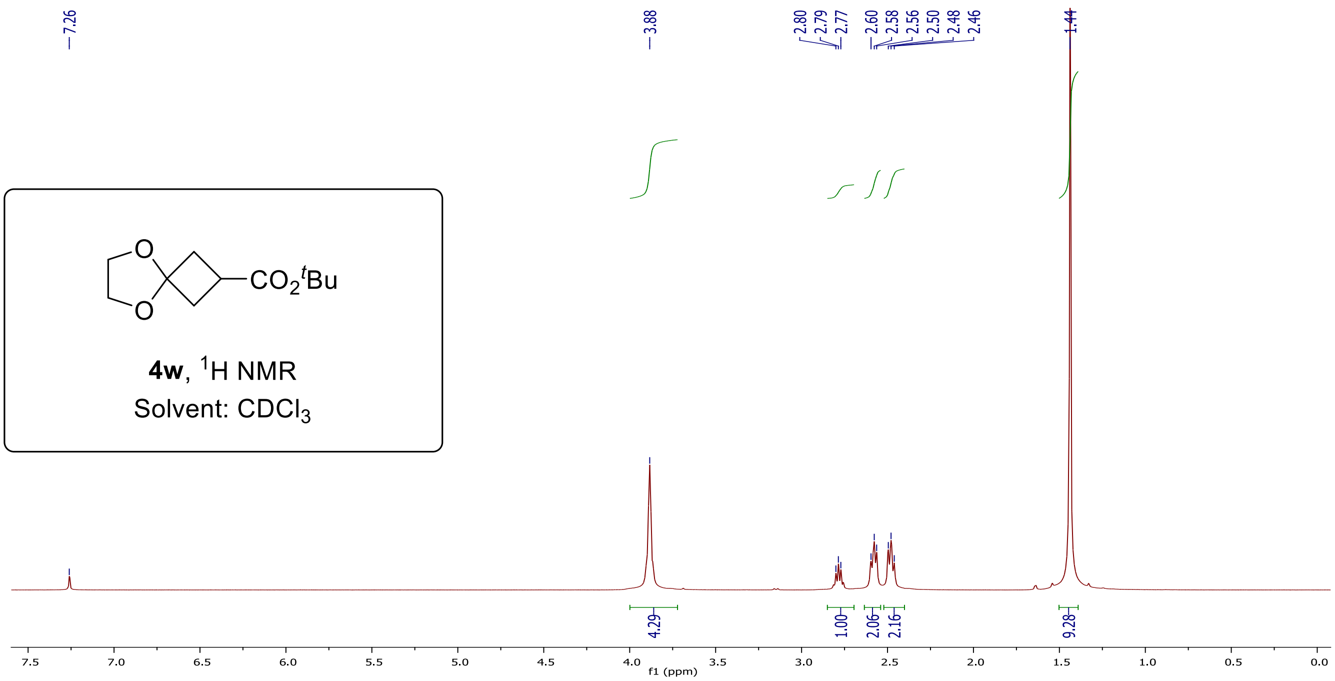

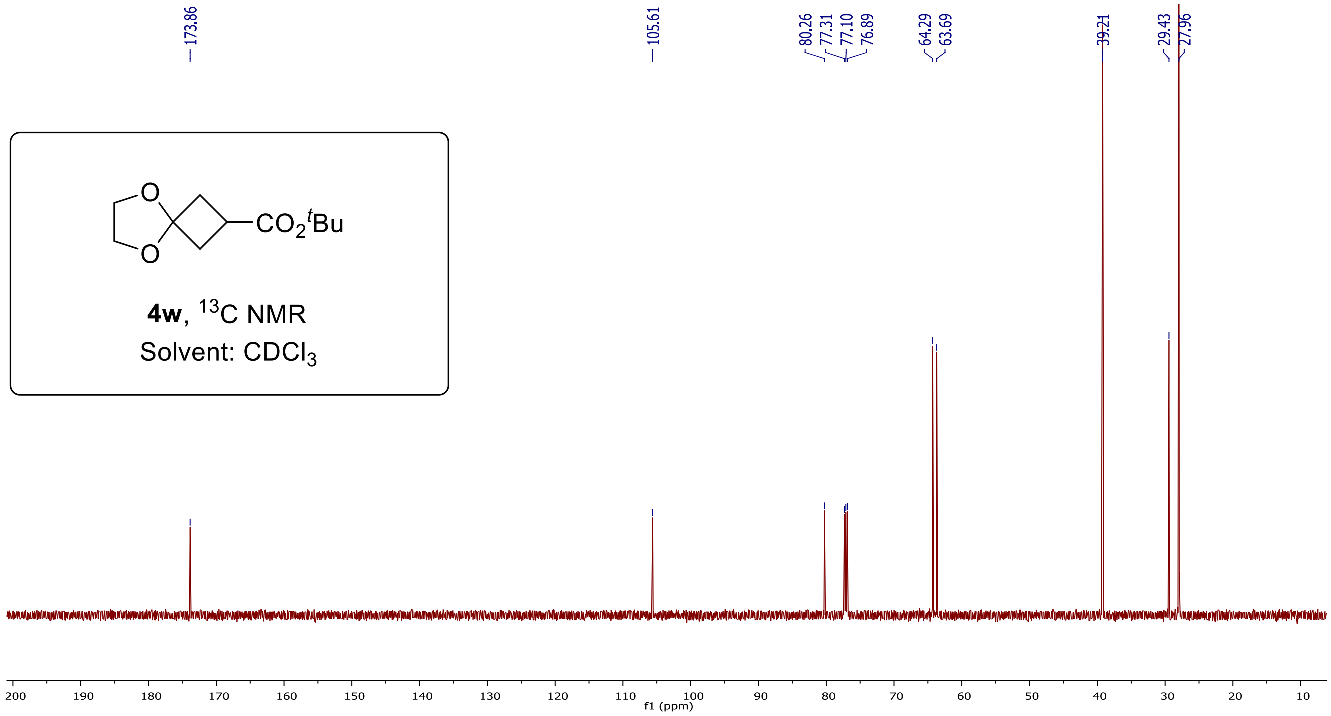

Supplementary Figure 40. NMR spectra of **4w**

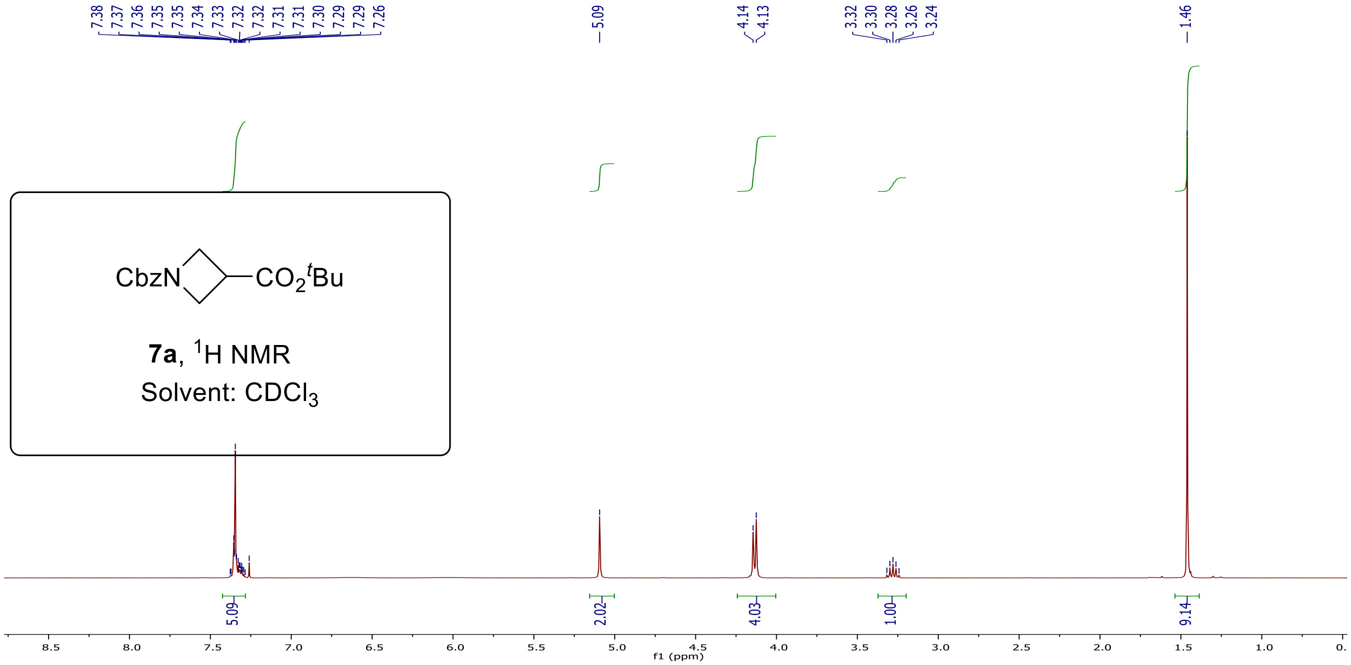

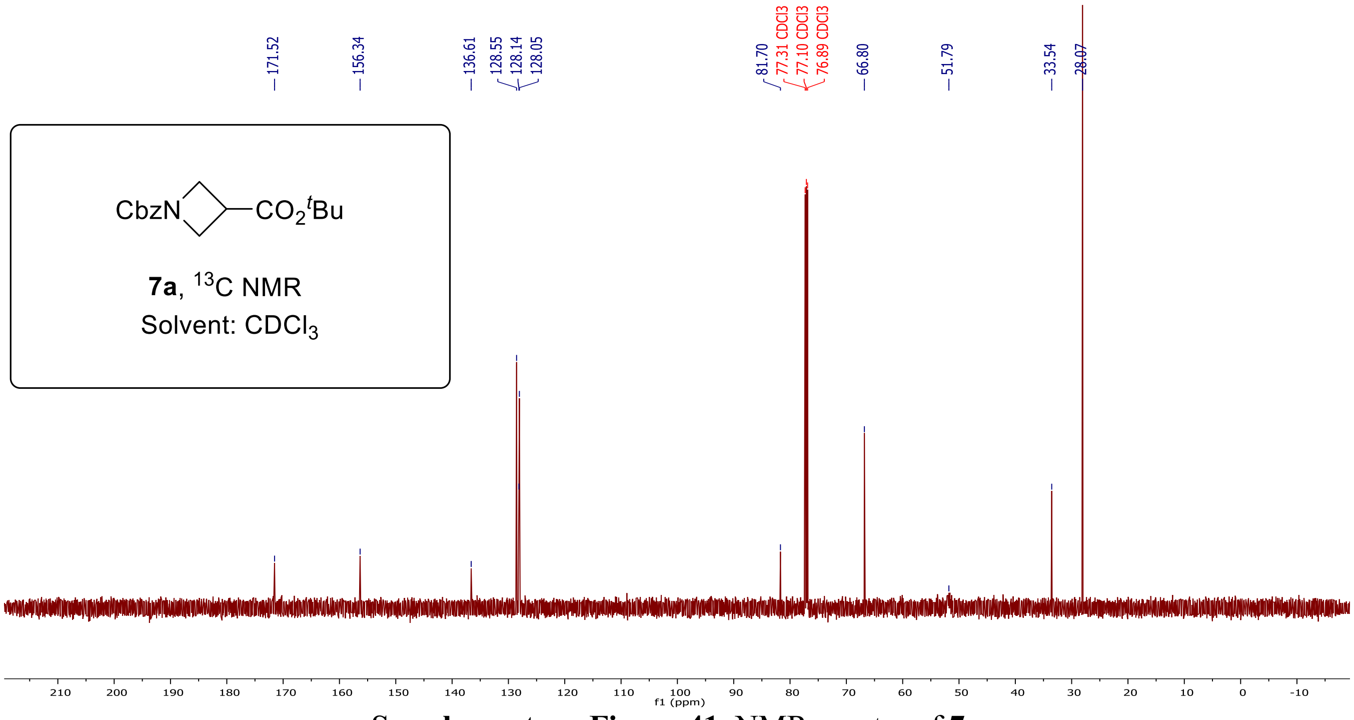

Supplementary Figure 41. NMR spectra of **7a**

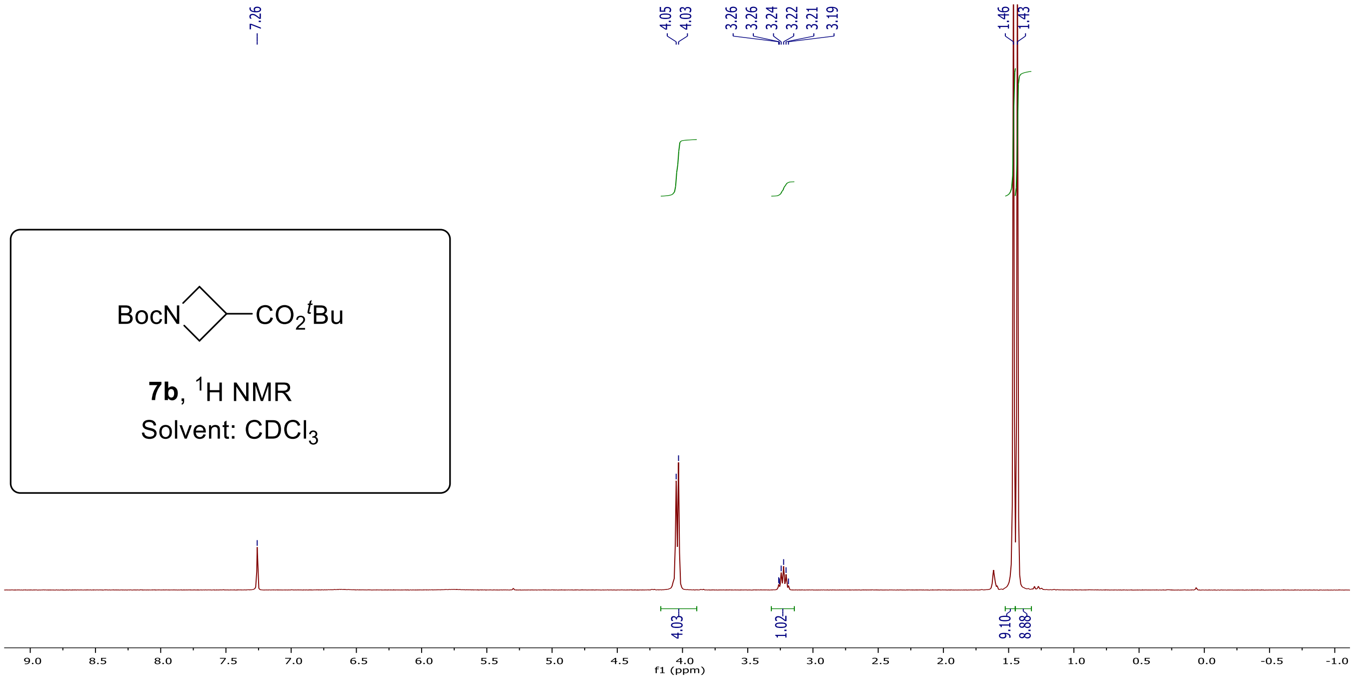

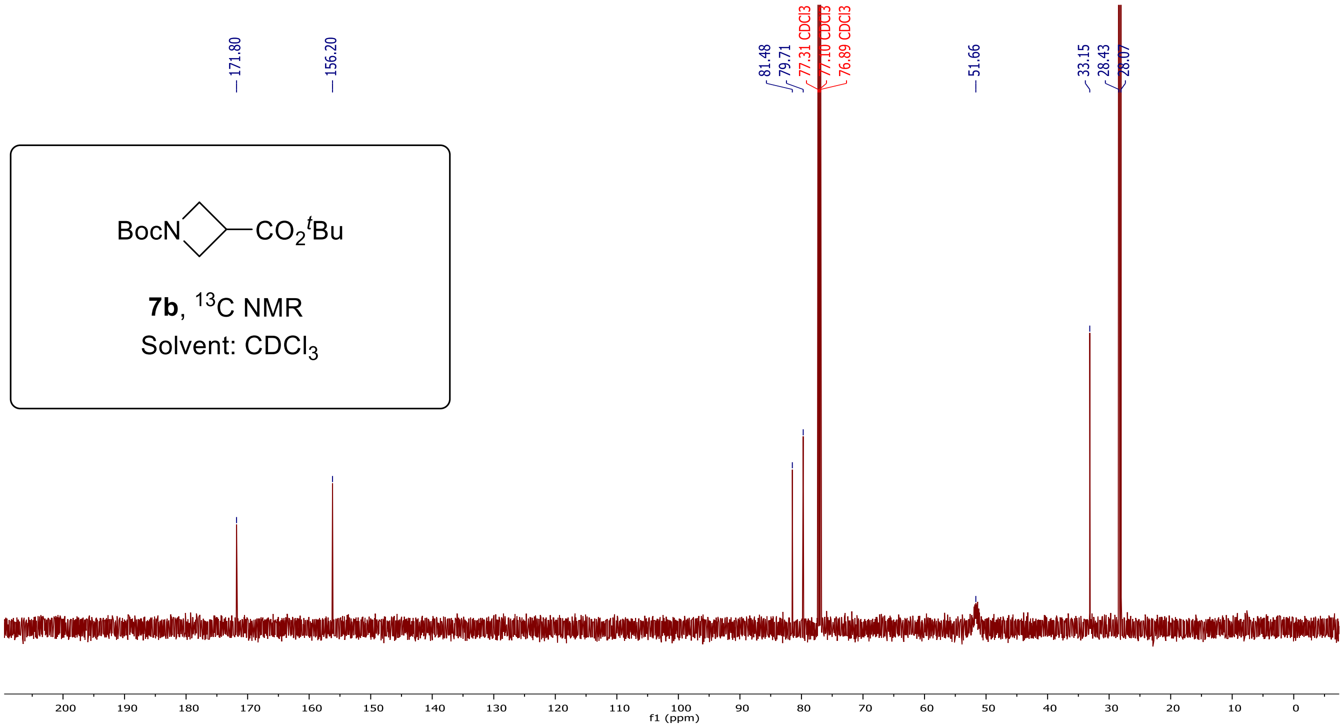

Supplementary Figure 42. NMR spectra of **7b**

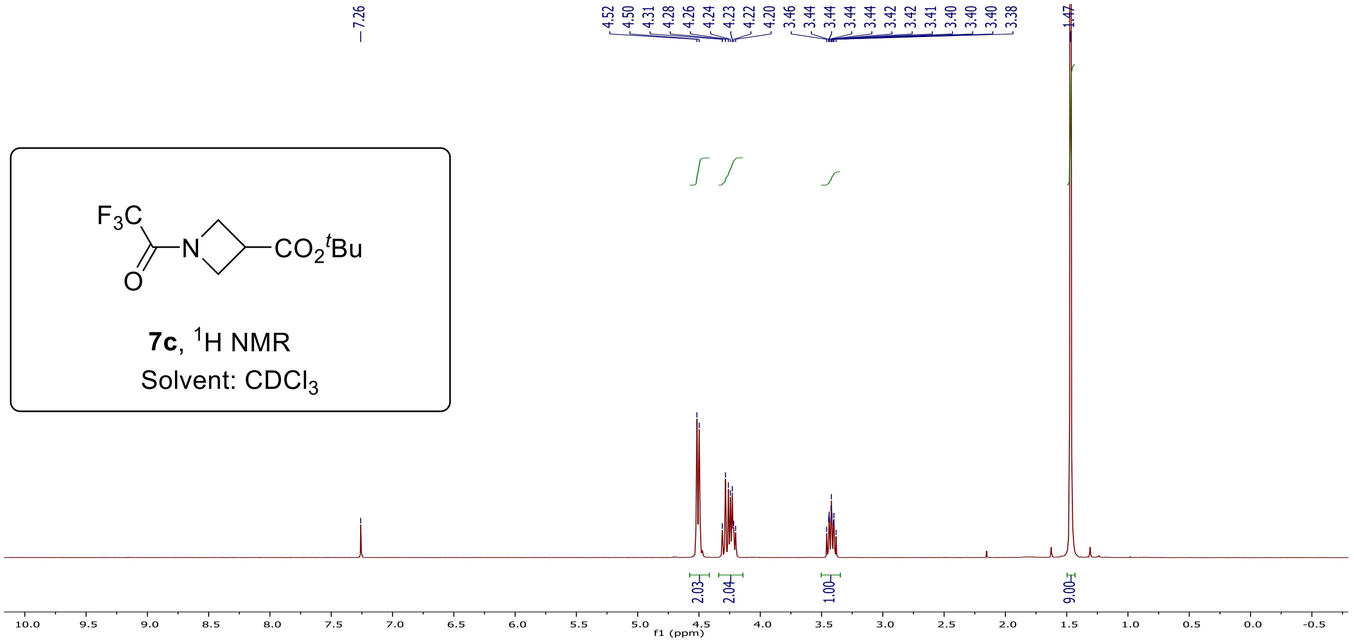

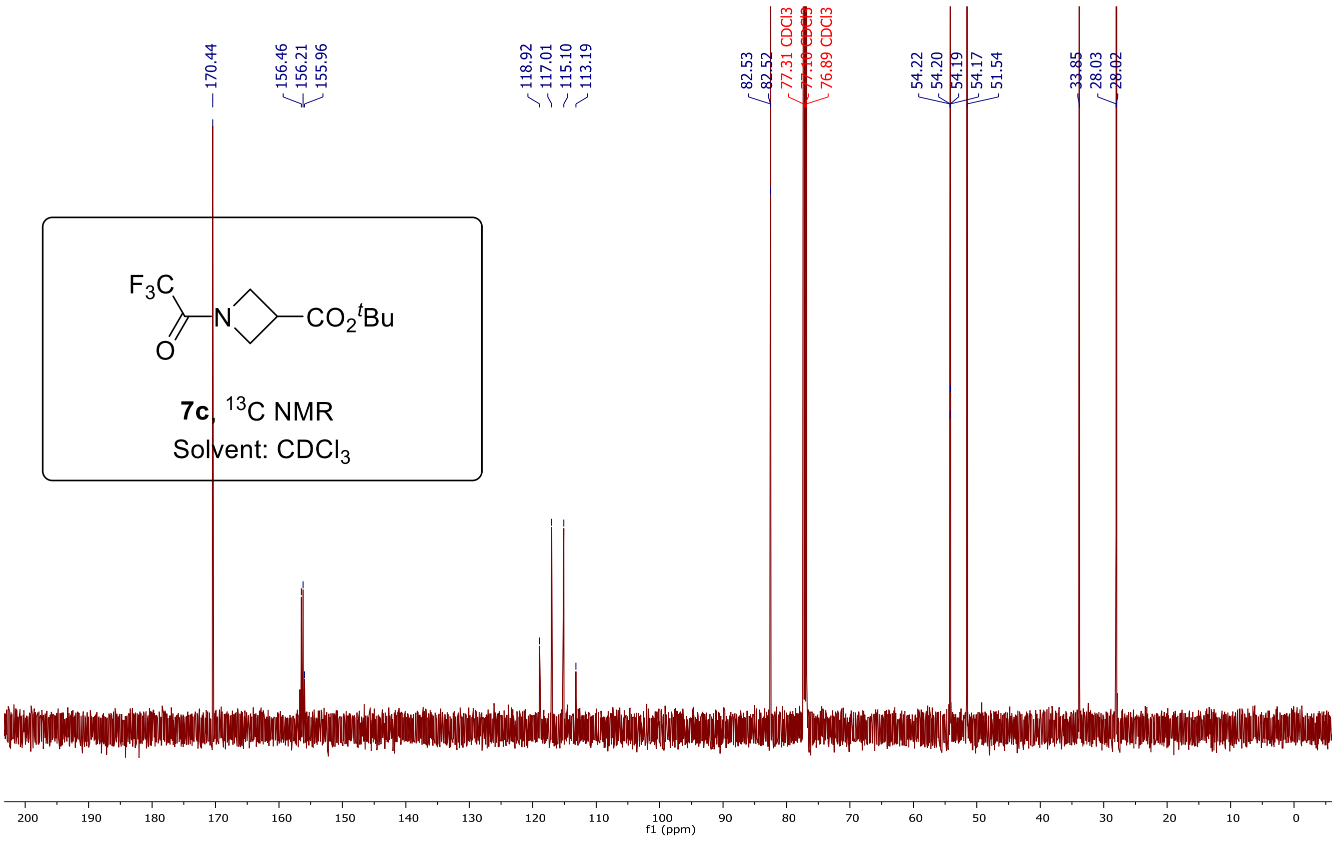

Supplementary Figure 43. NMR spectra of **7c**

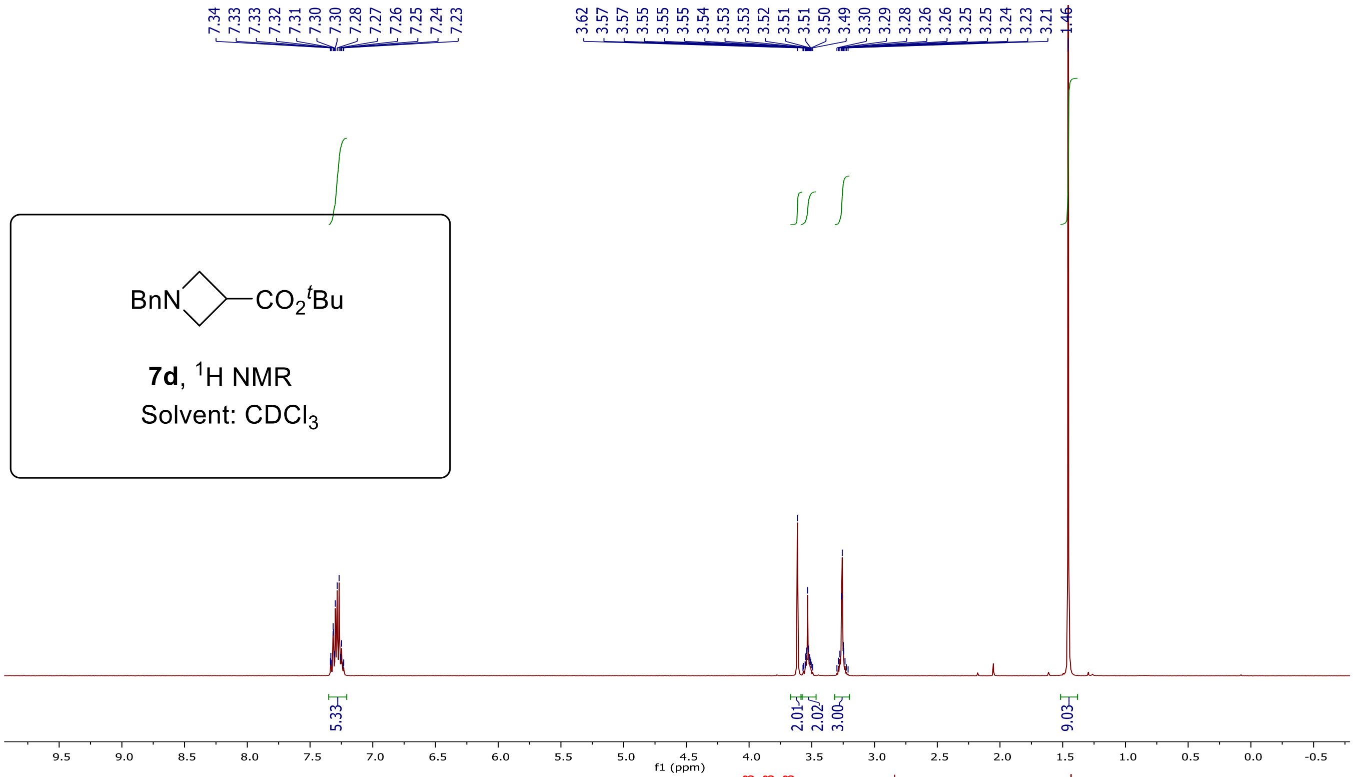

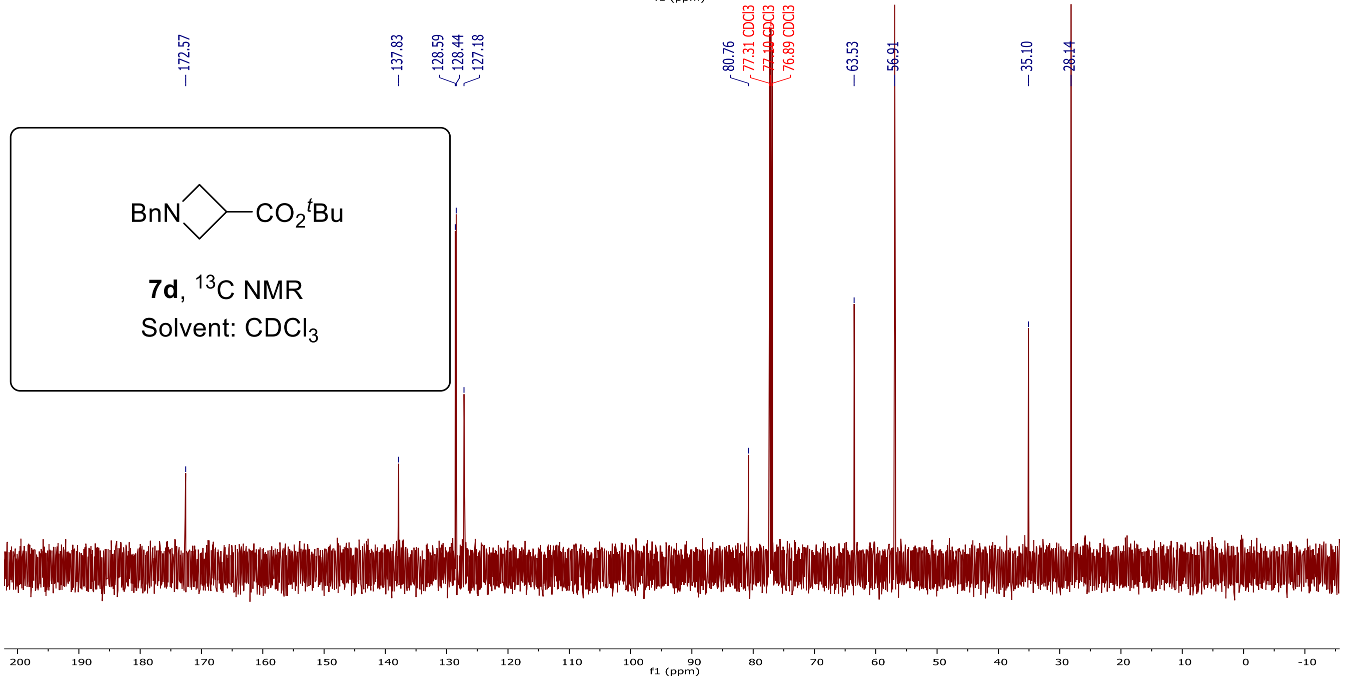

Supplementary Figure 44. NMR spectra of **7d**

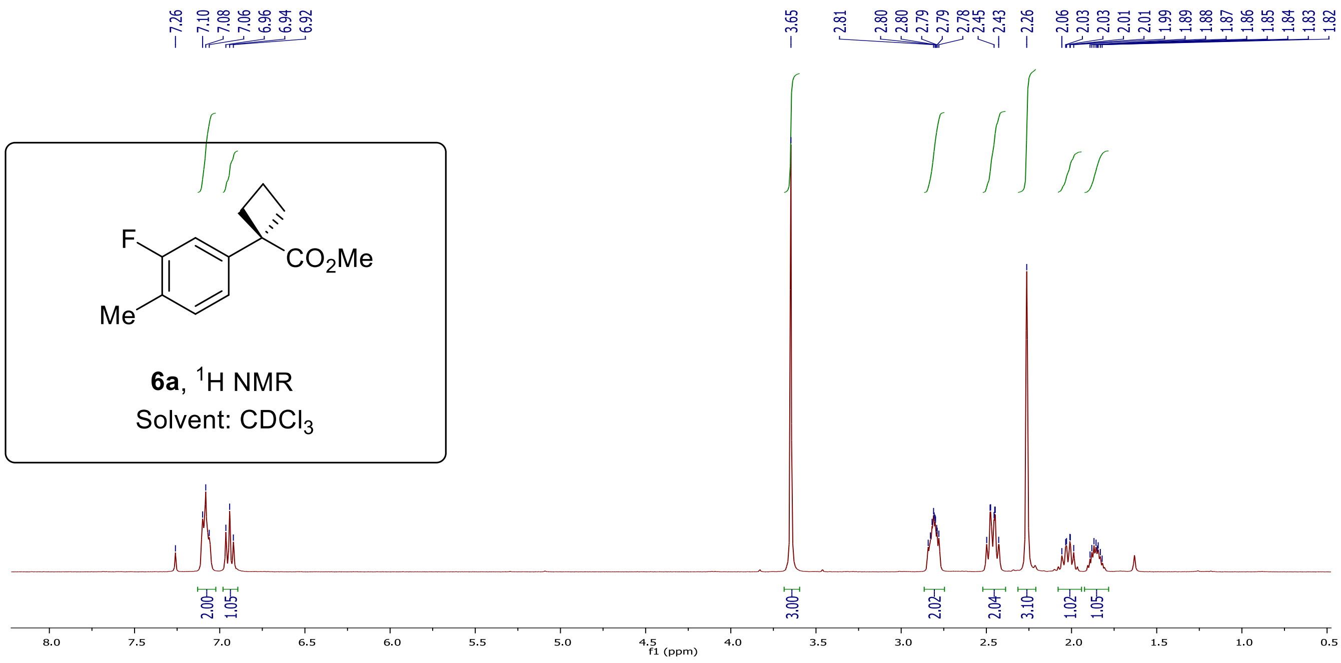

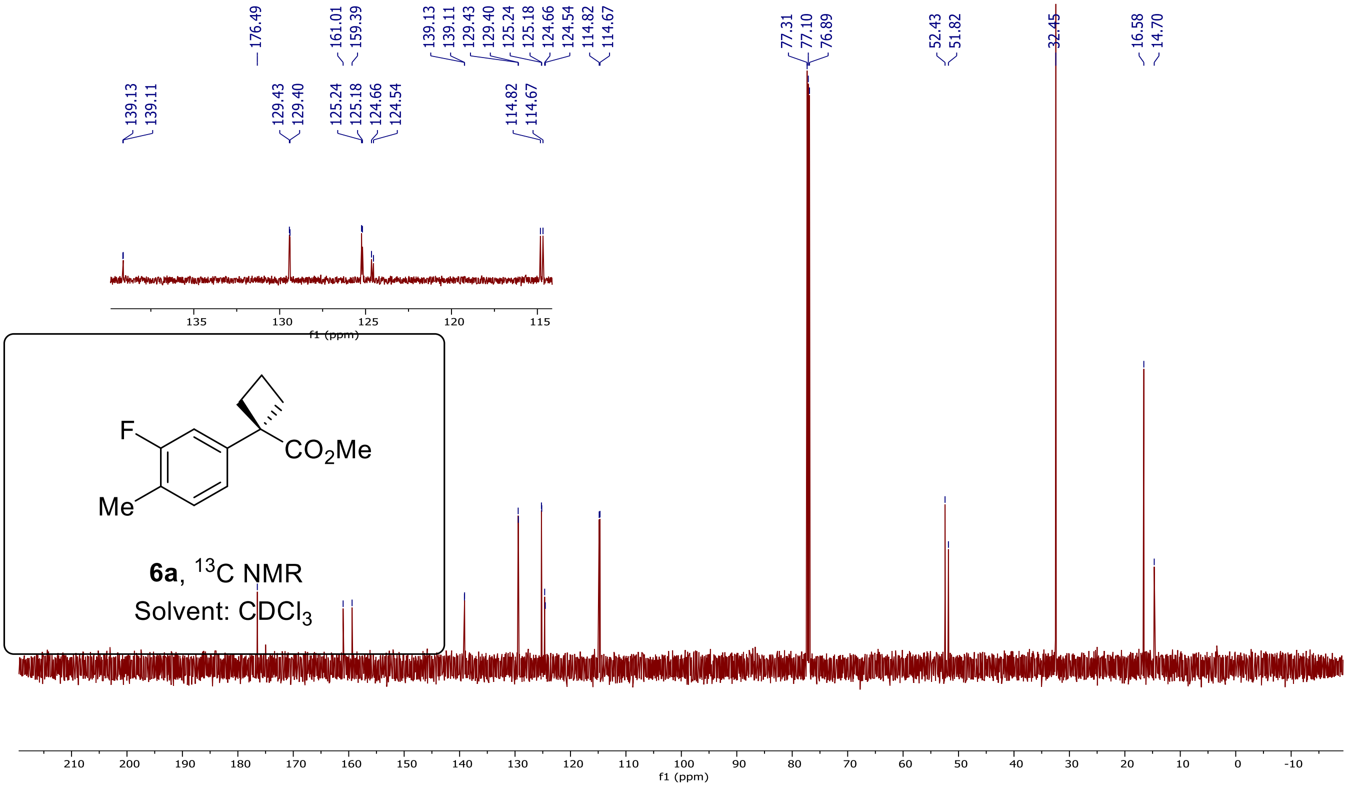

Supplementary Figure 45. NMR spectra of **6a**

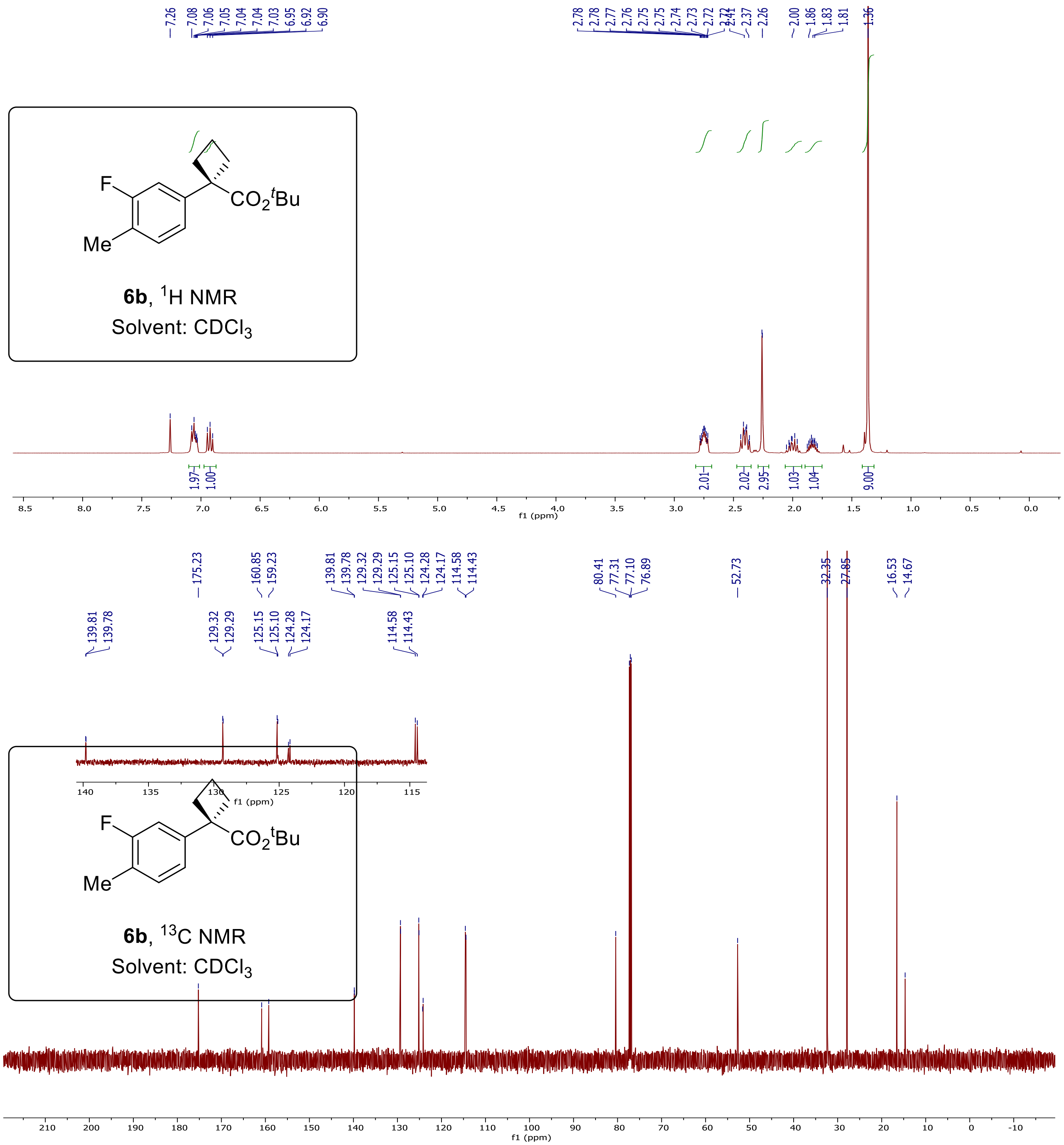

Supplementary Figure 46. NMR spectra of **6b**

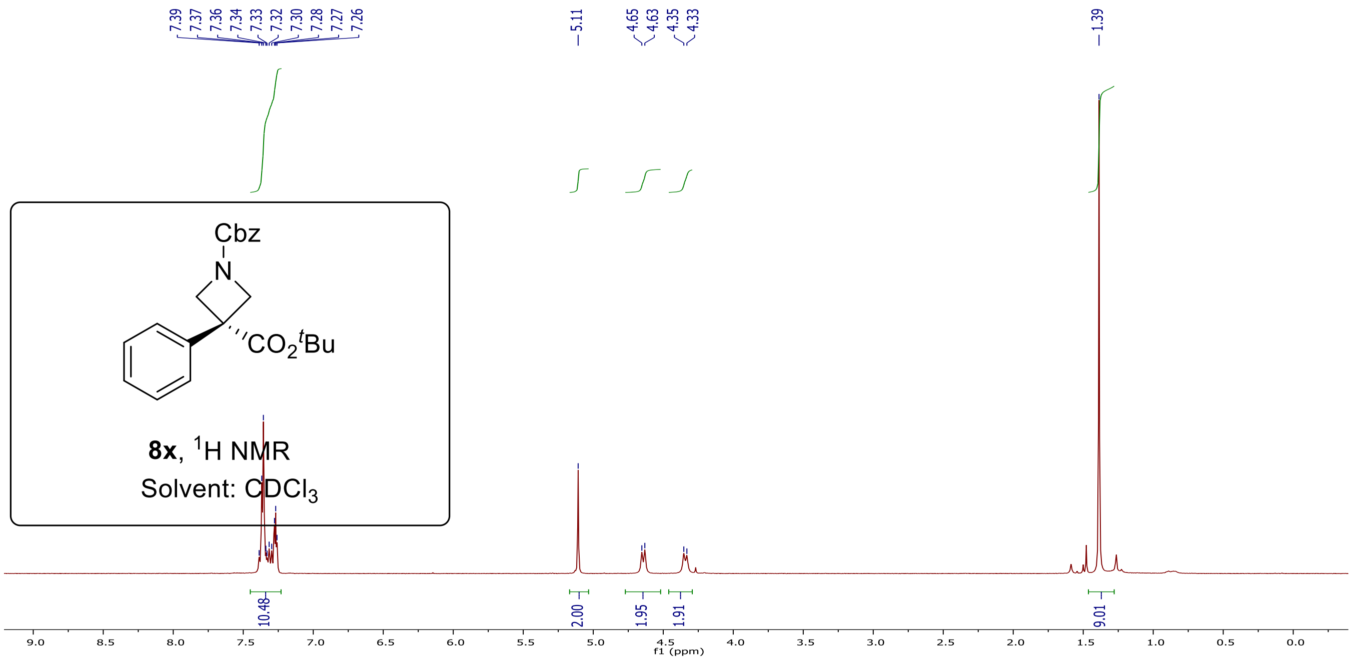

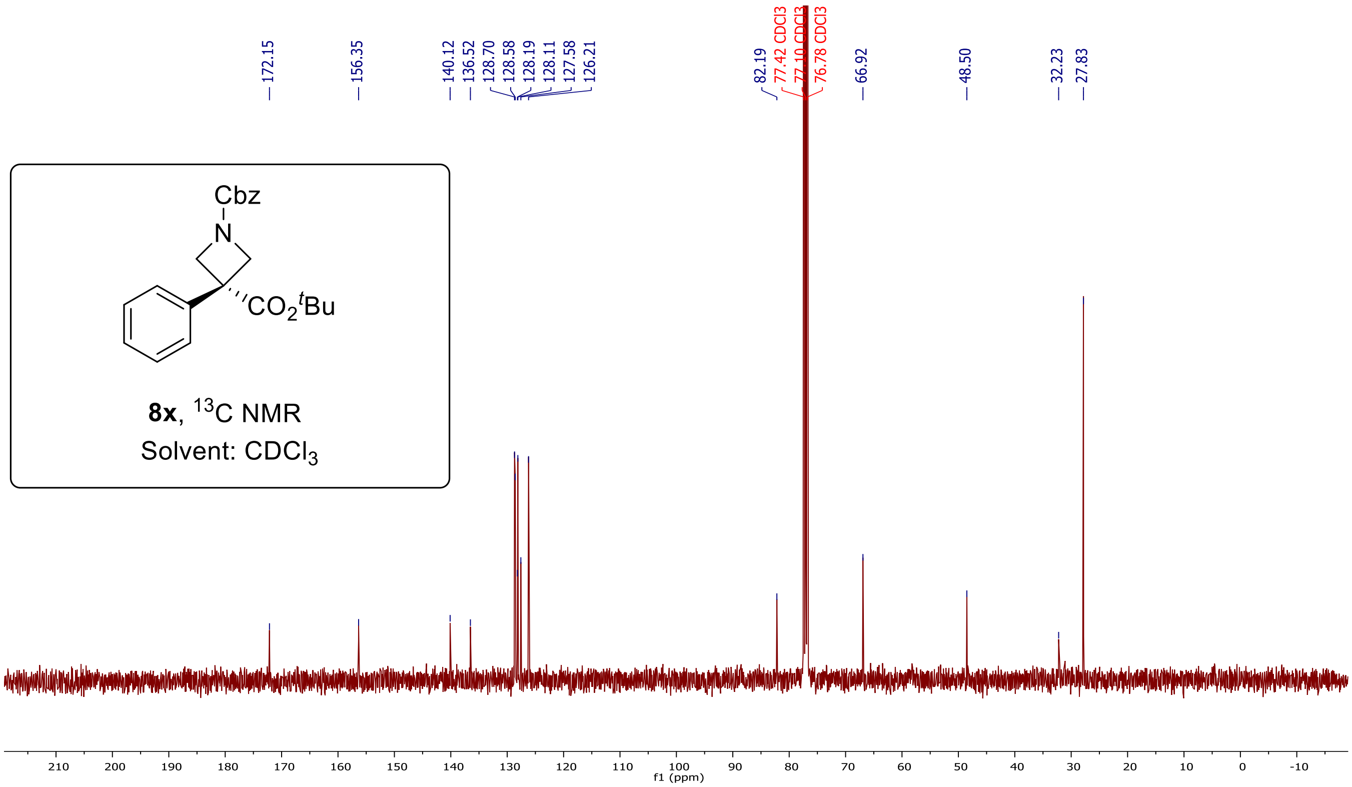

Supplementary Figure 47. NMR spectra of **8x**

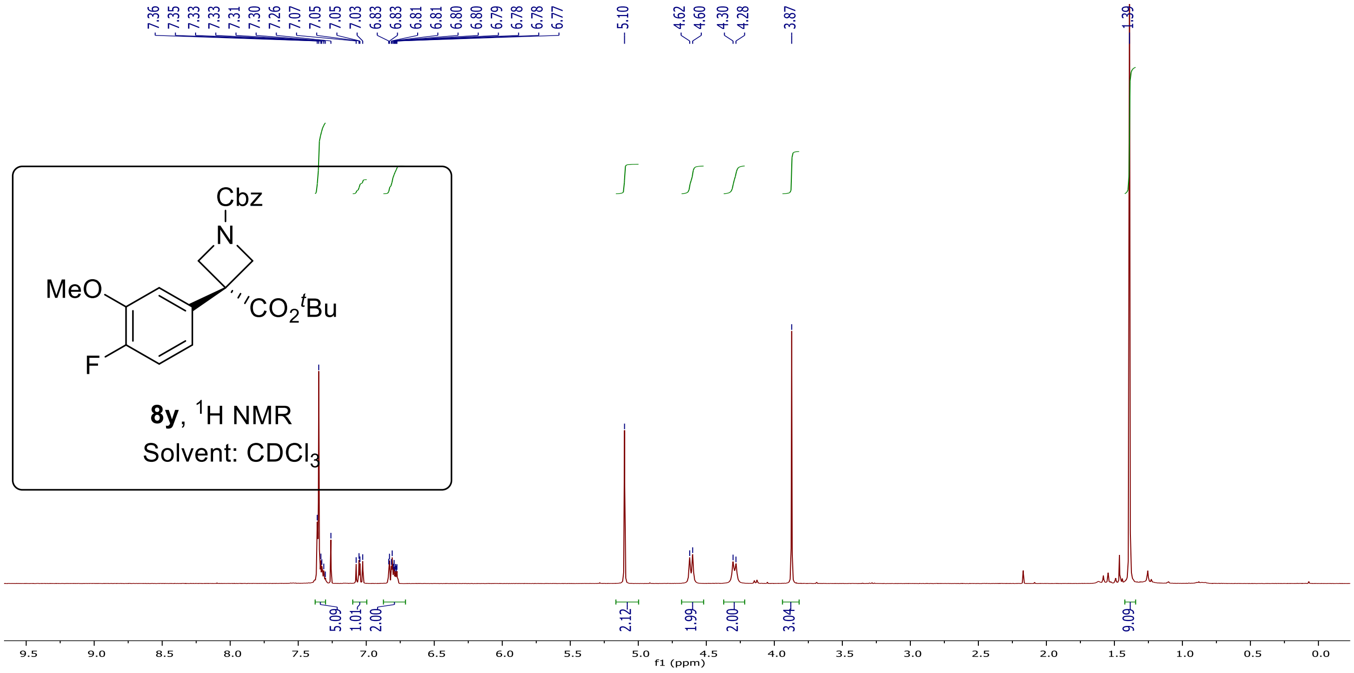

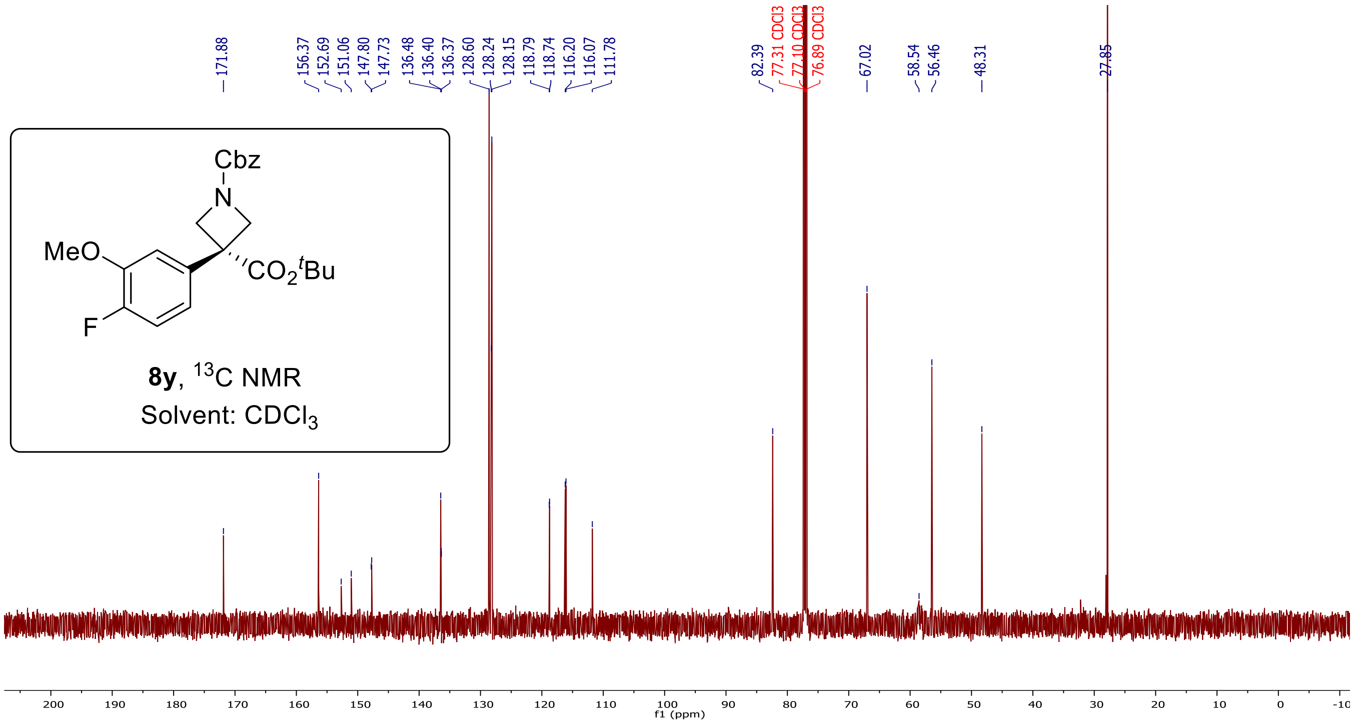

Supplementary Figure 48. NMR spectra of **8y**

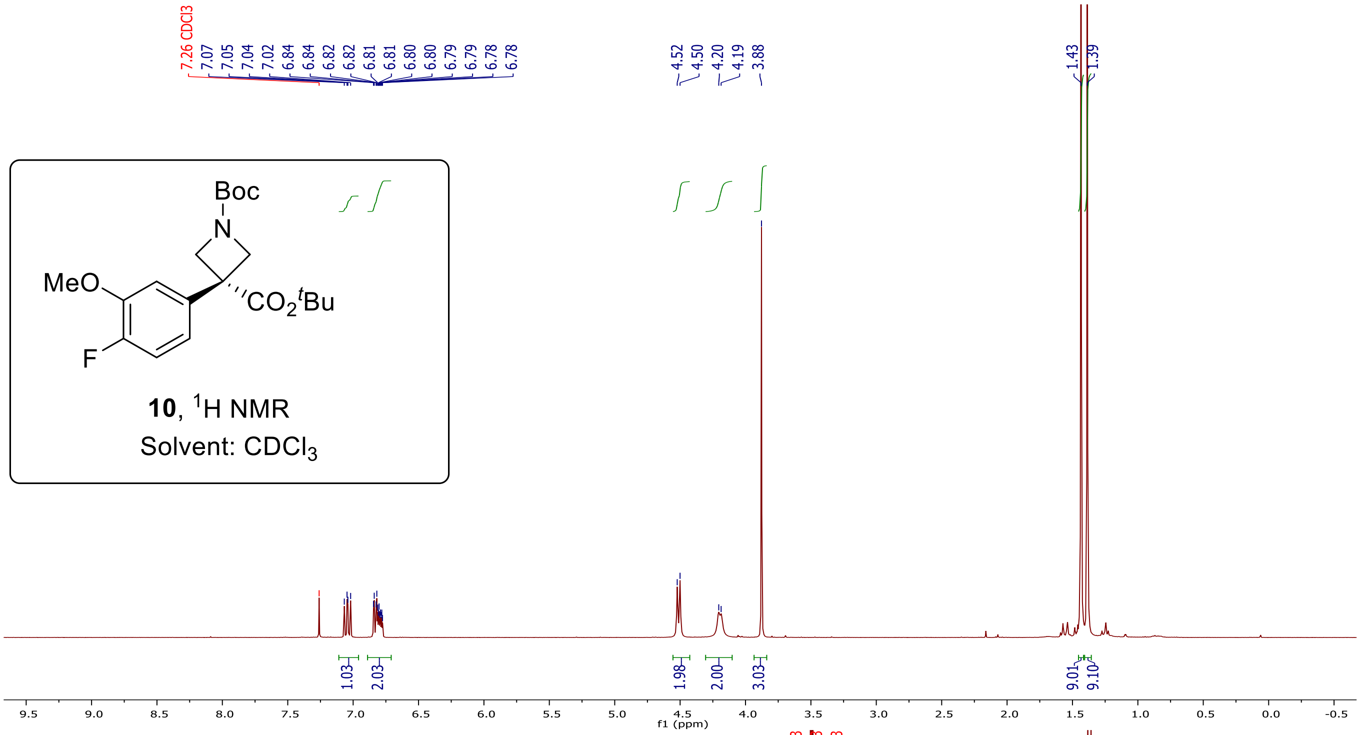

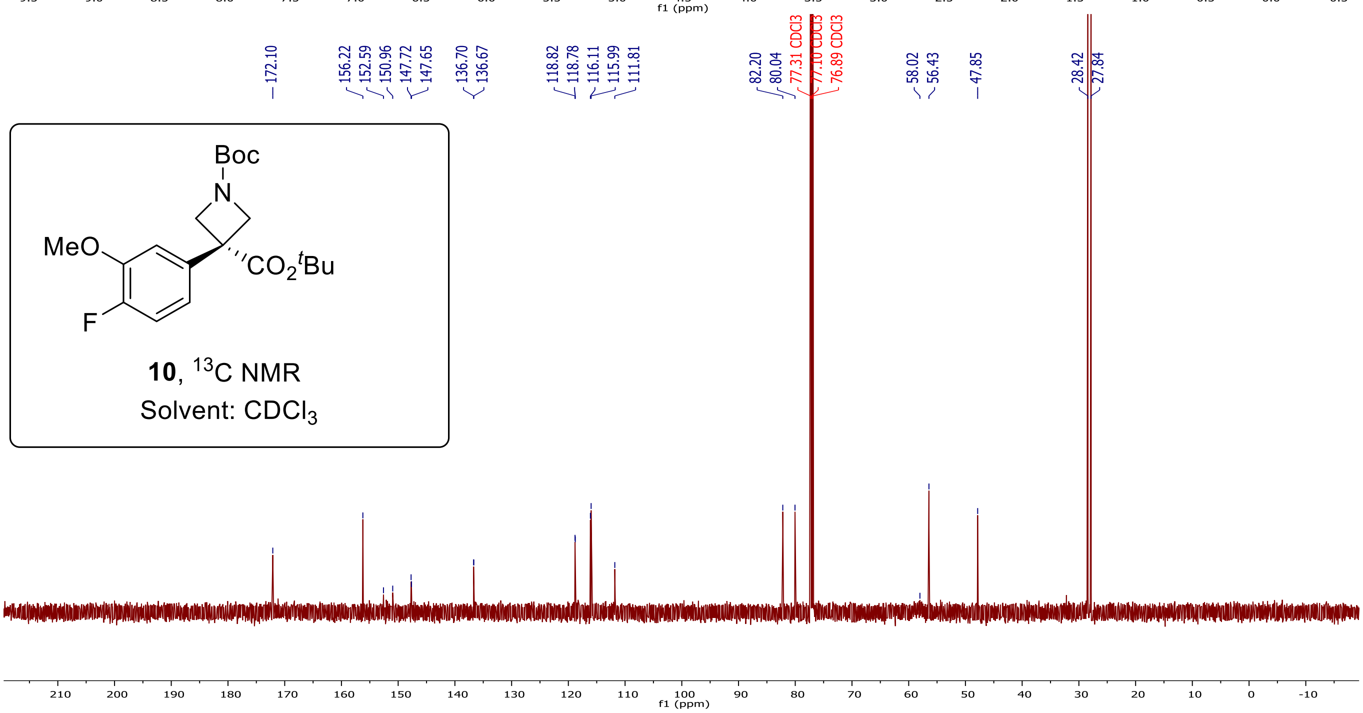

Supplementary Figure 49. NMR spectra of **10**

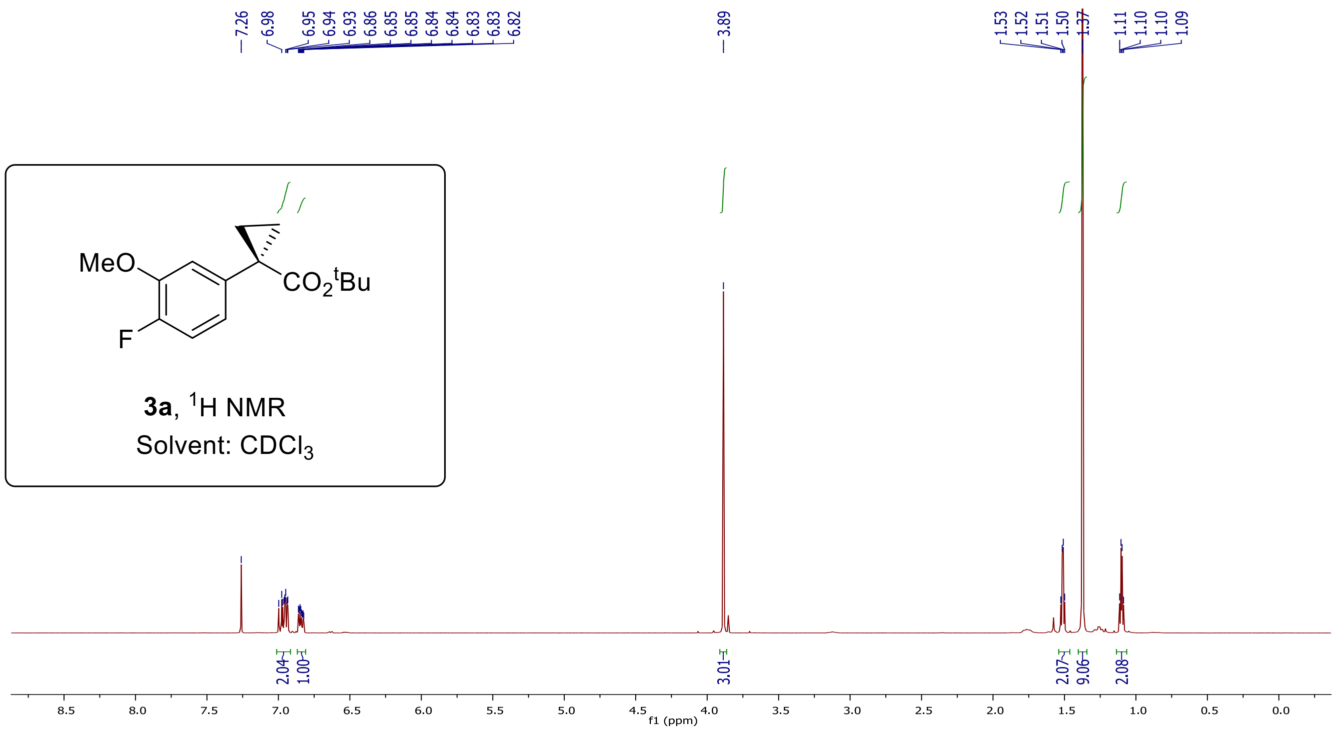

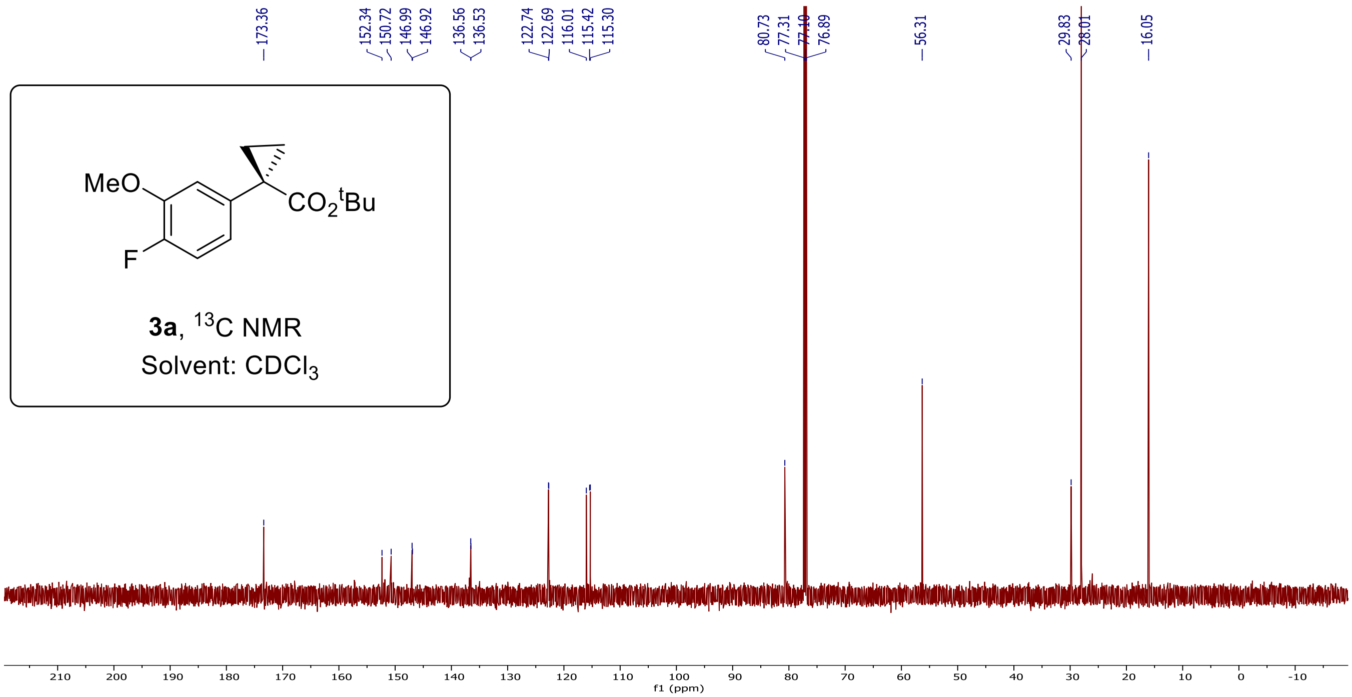

Supplementary Figure 50. NMR spectra of **3a**

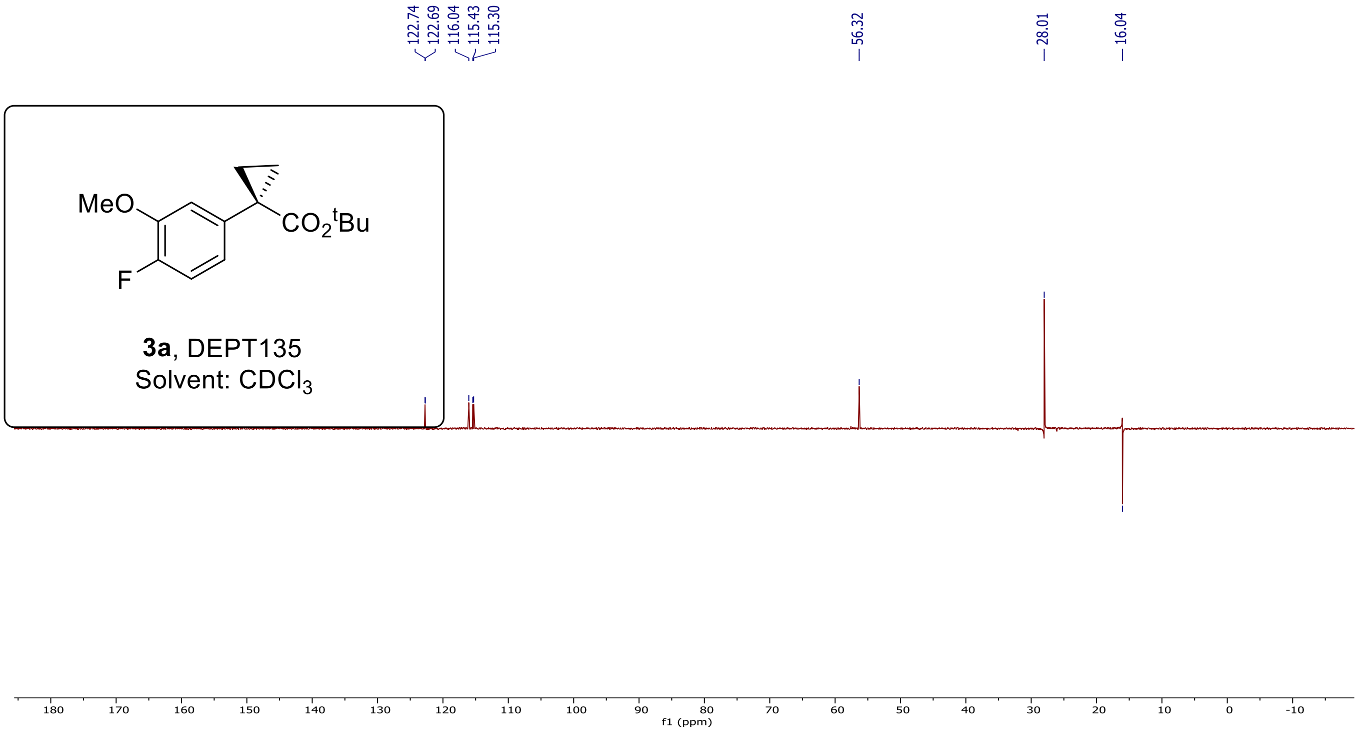

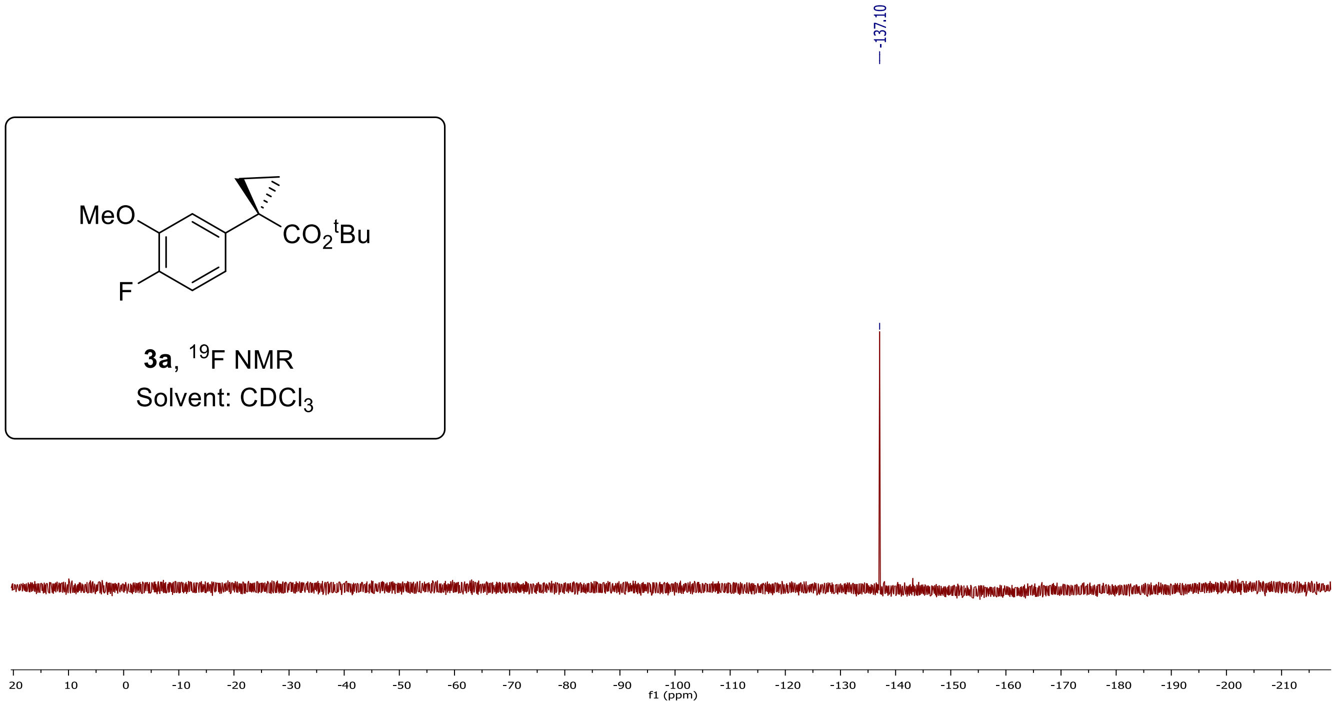

Supplementary Figure 51. NMR spectra of **3a**

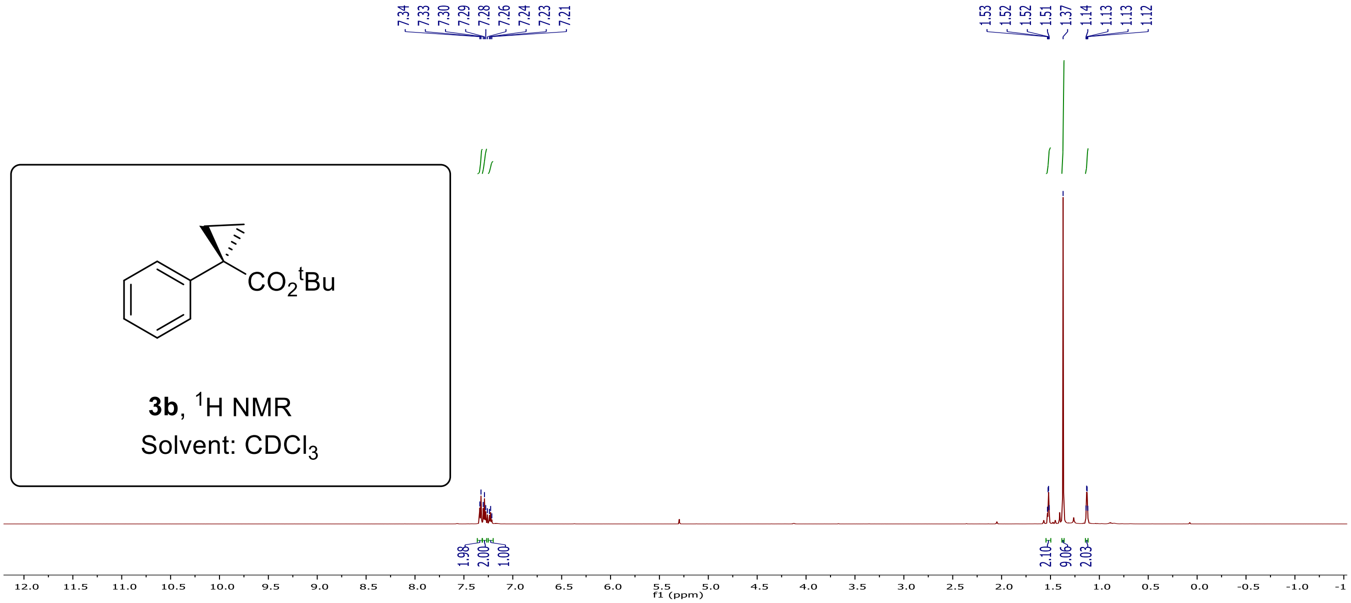

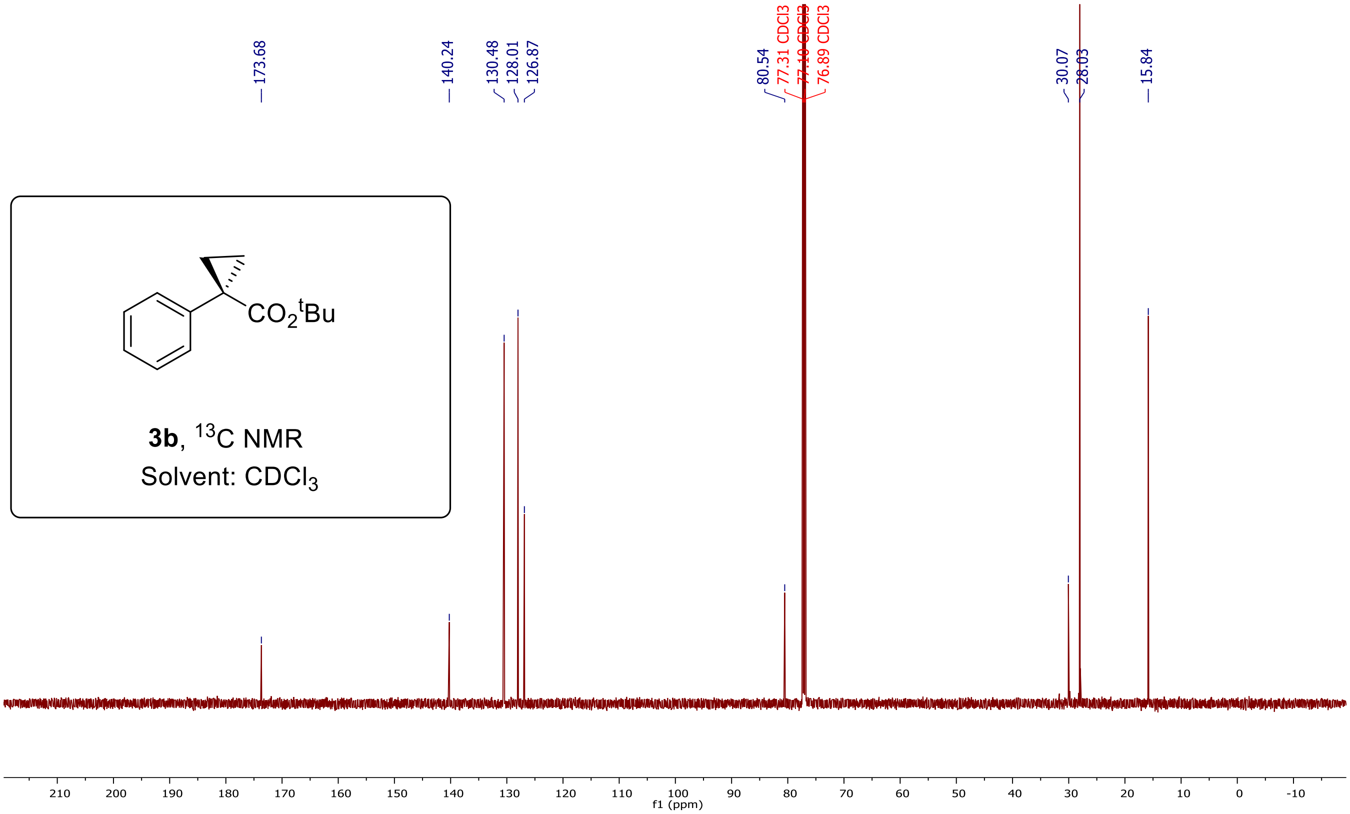

Supplementary Figure 52. NMR spectra of **3b**

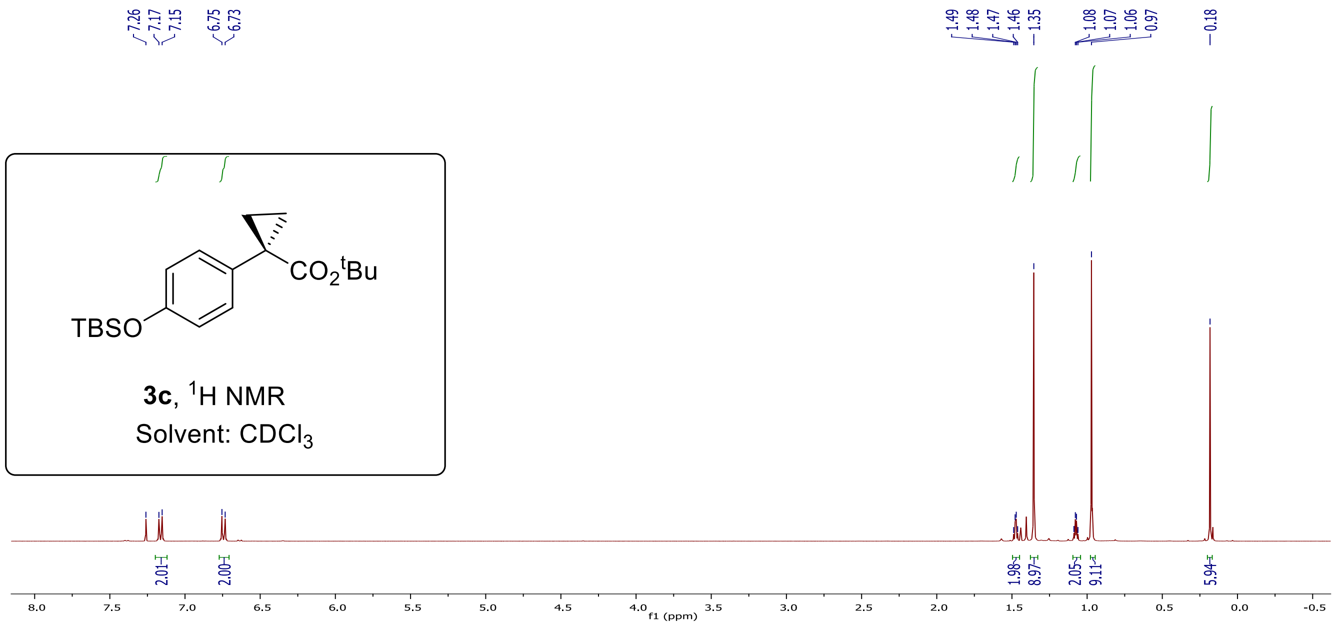

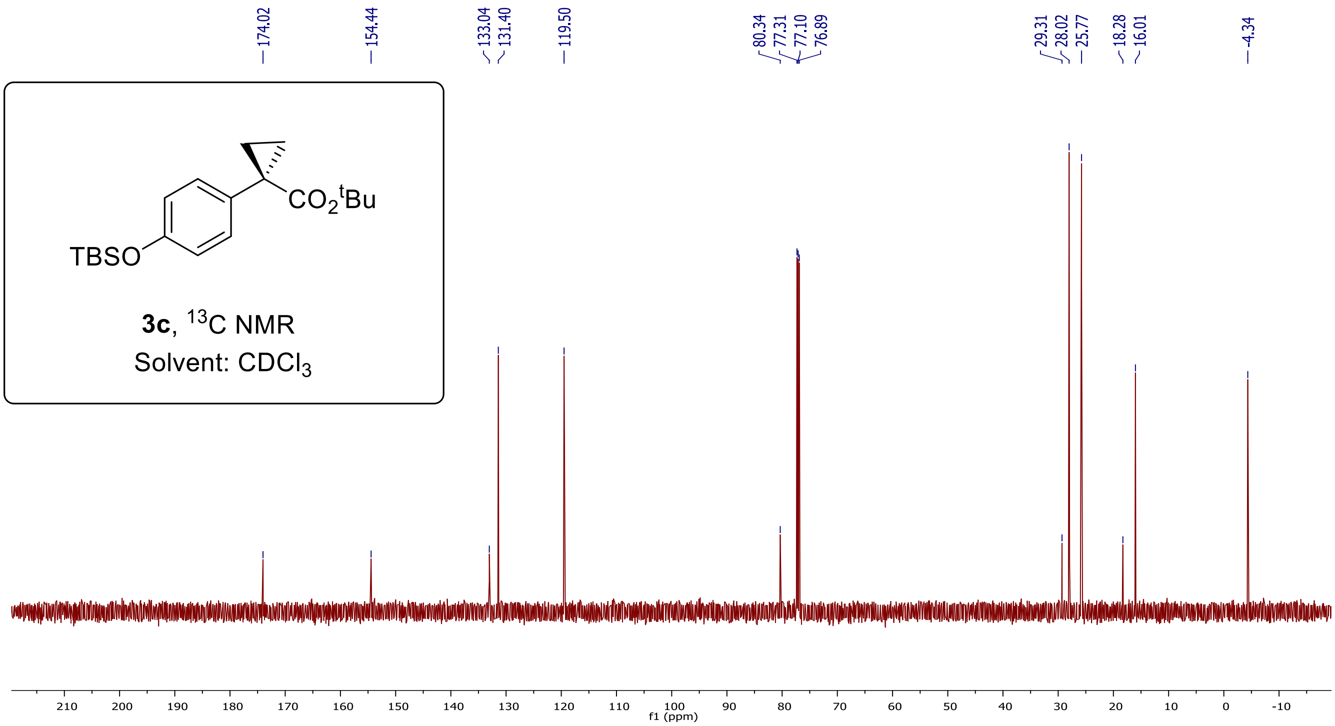

Supplementary Figure 53. NMR spectra of **3c**

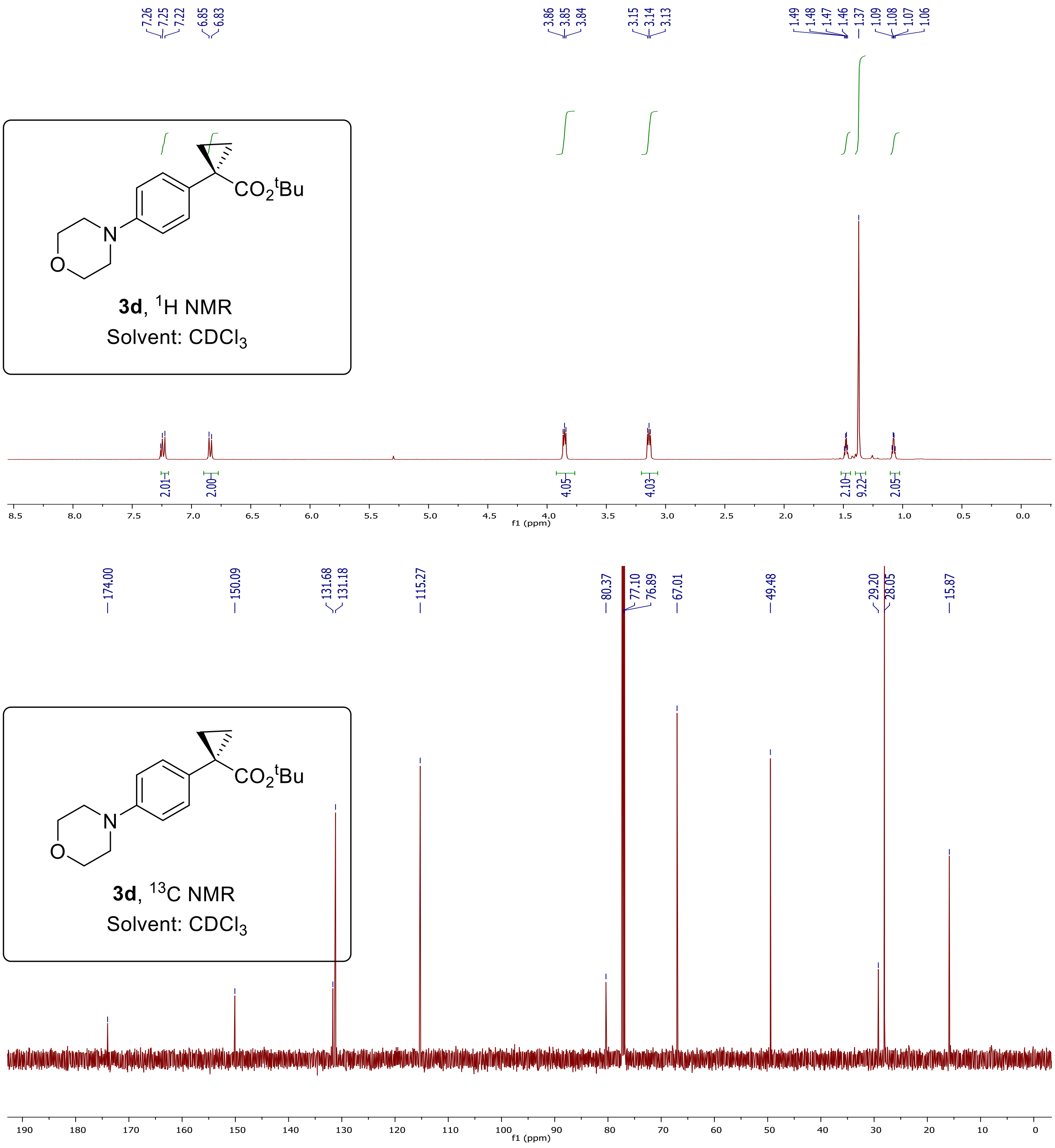

Supplementary Figure 54. NMR spectra of **3d**

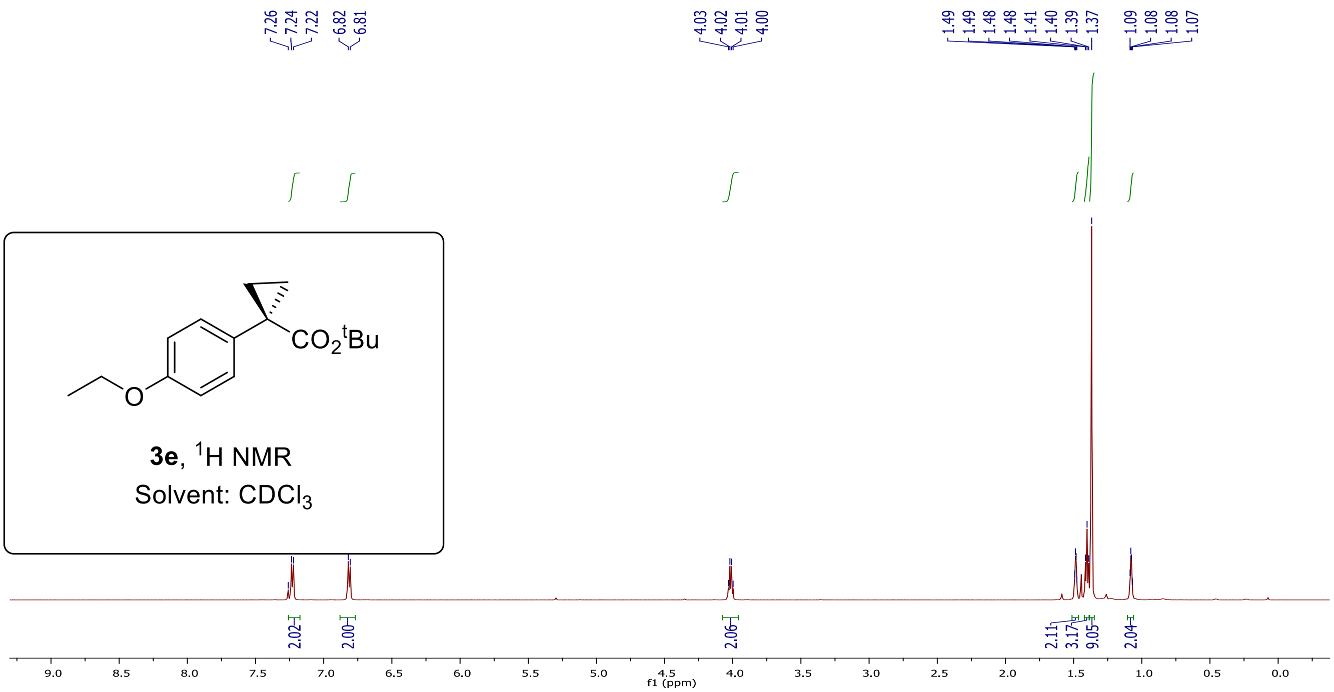

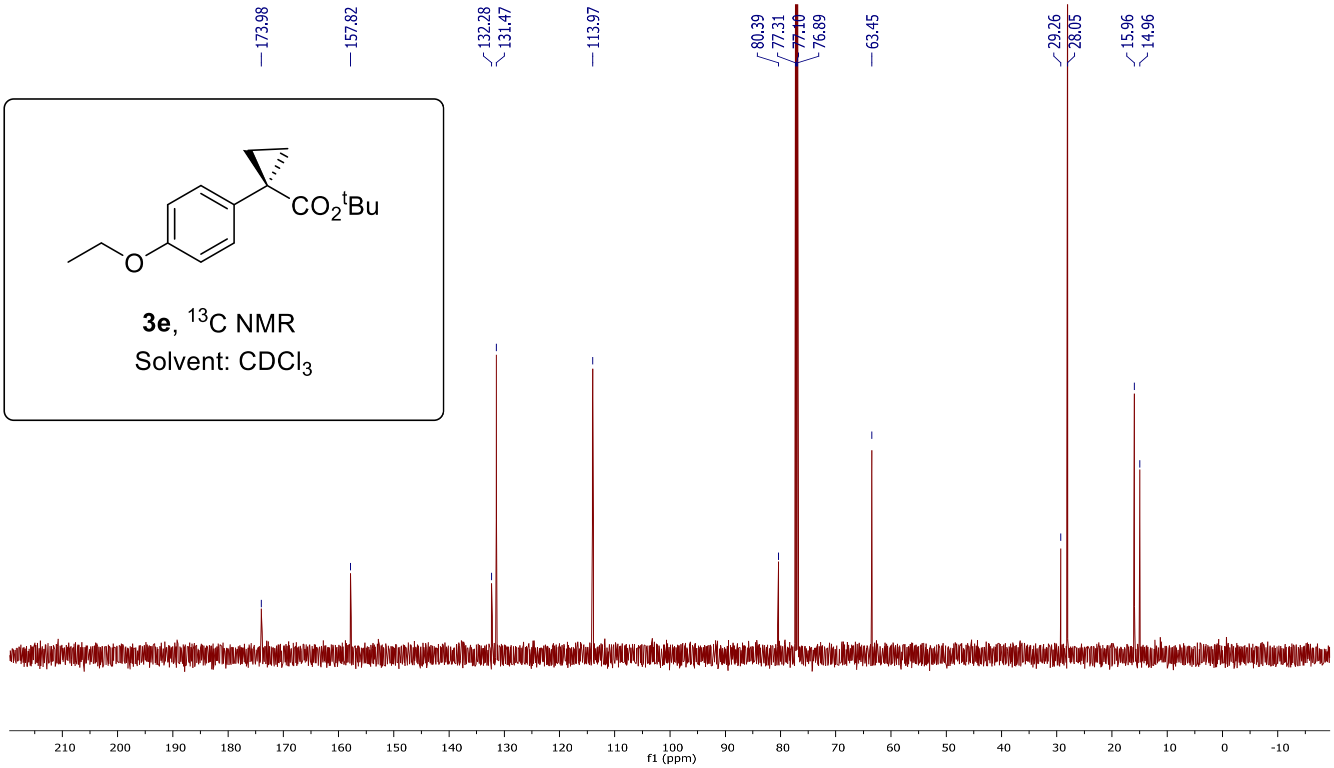

Supplementary Figure 55. NMR spectra of **3e**

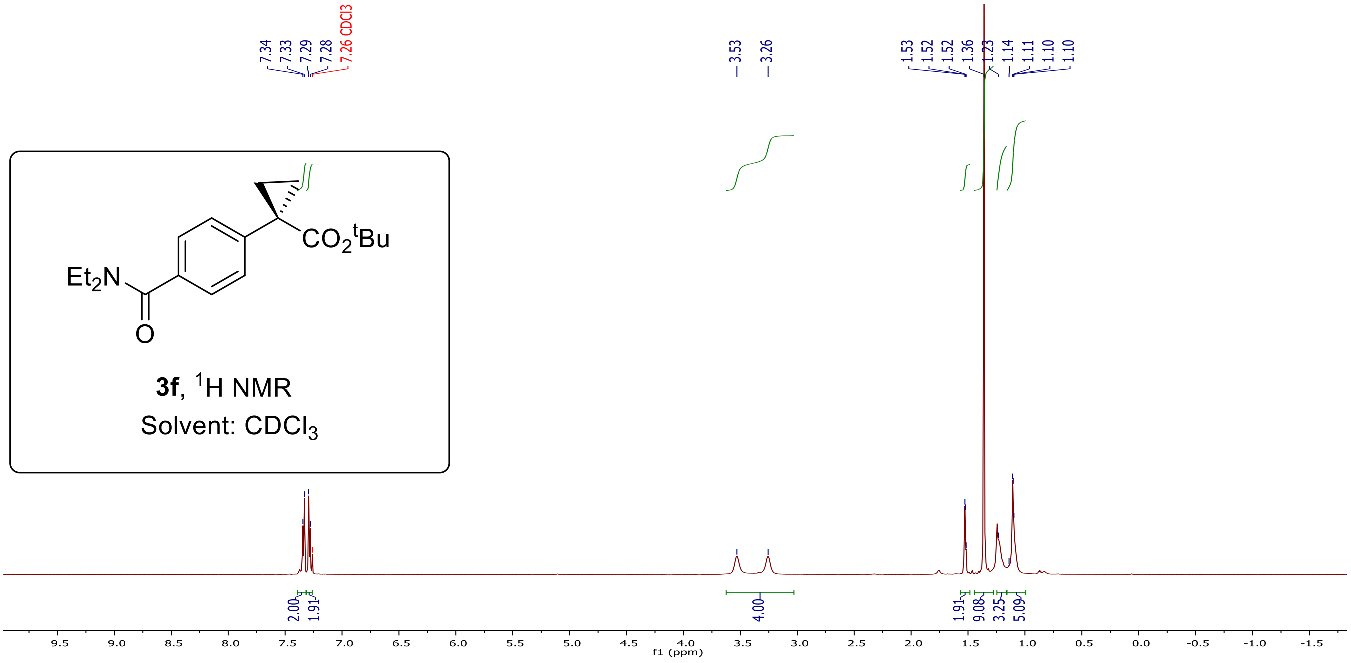

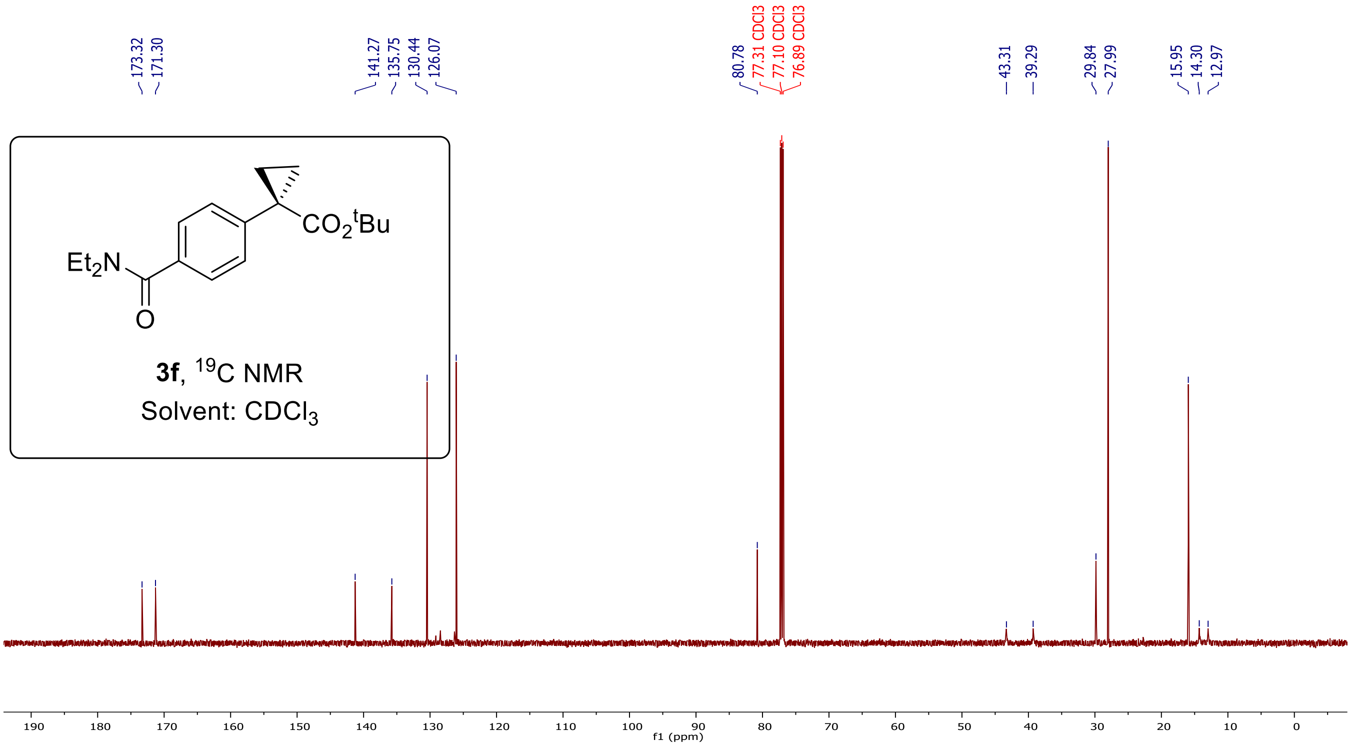

Supplementary Figure 56. NMR spectra of **3f**

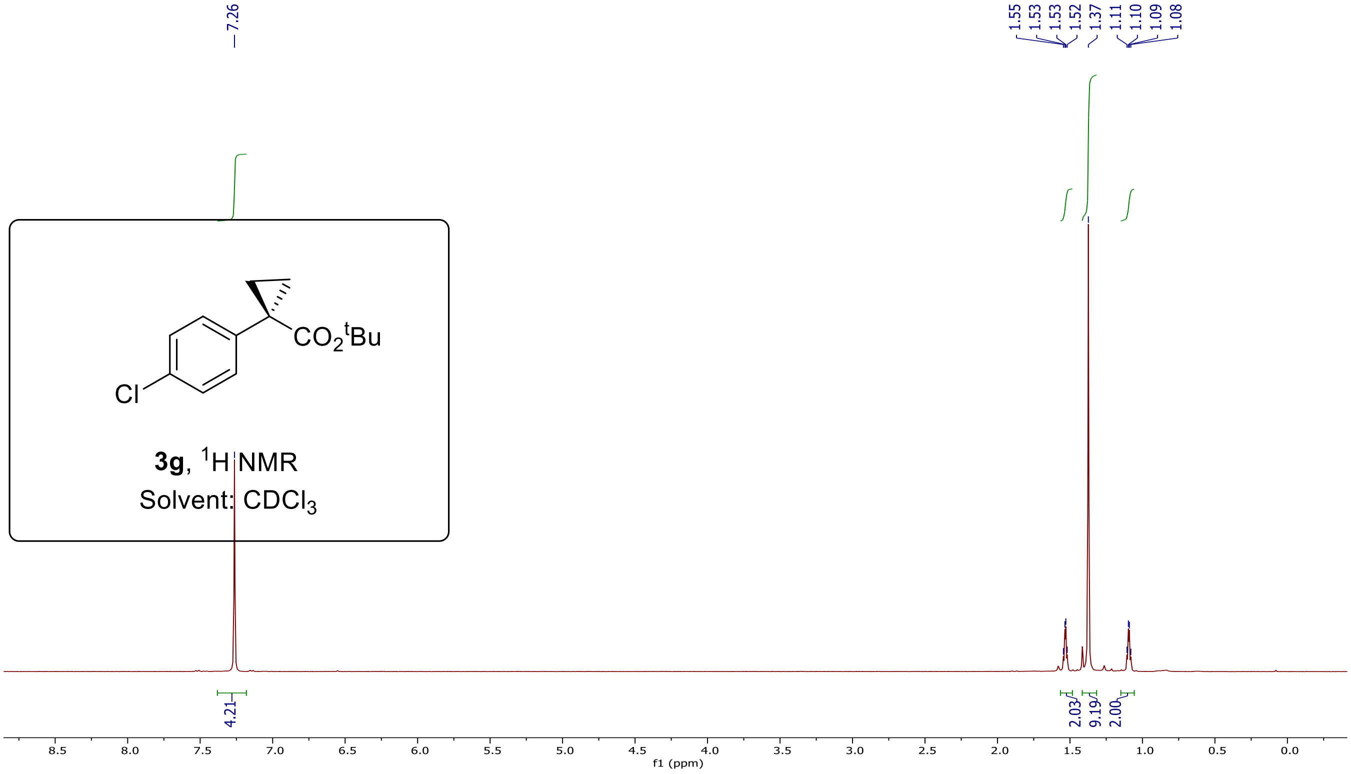

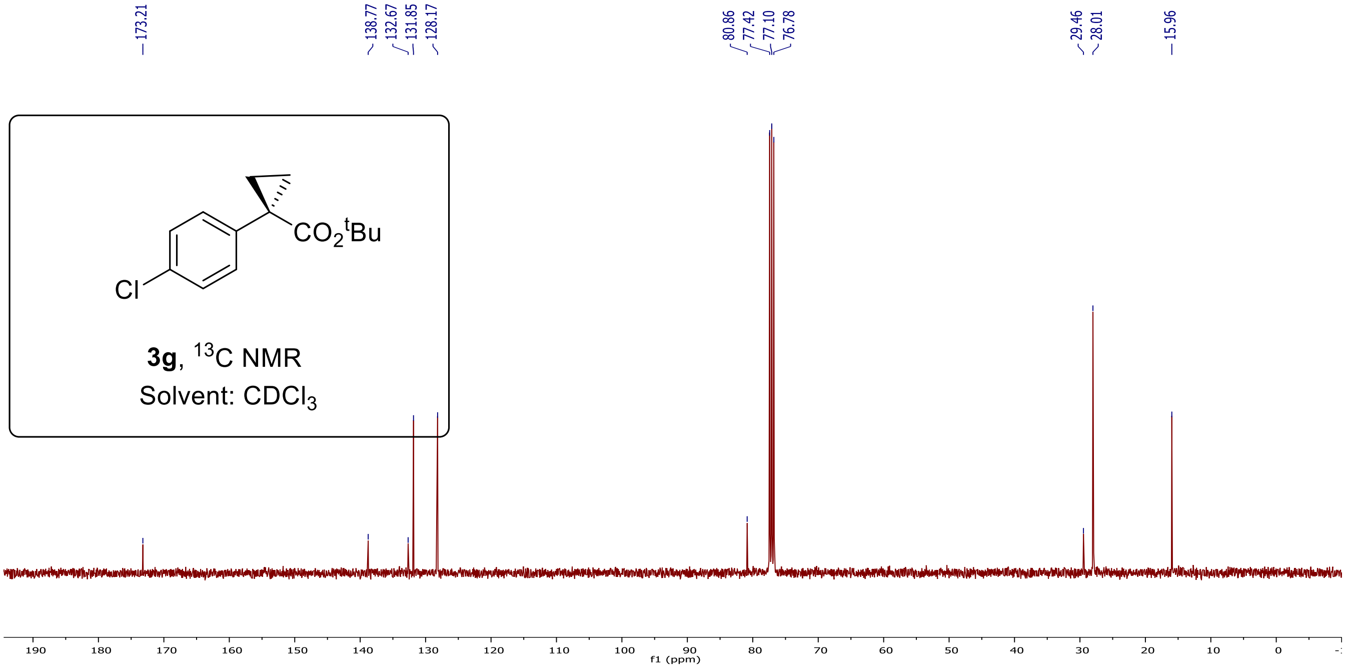

Supplementary Figure 57. NMR spectra of **3g**

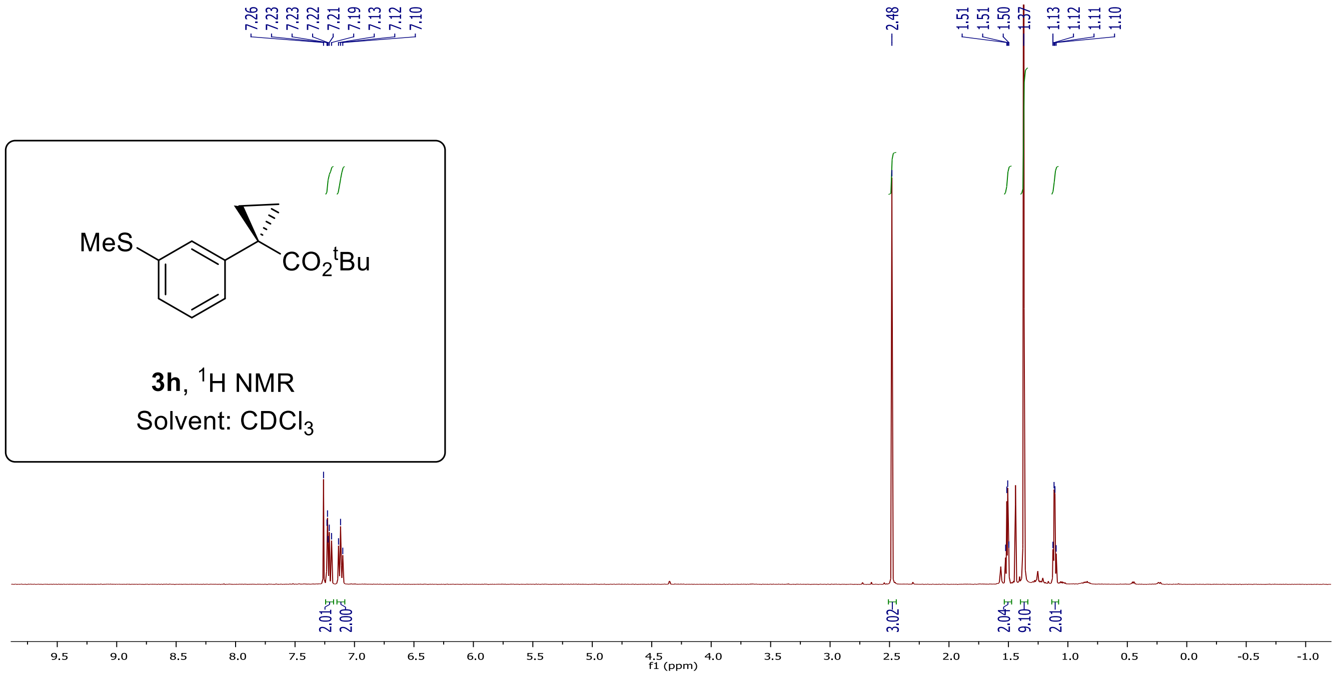

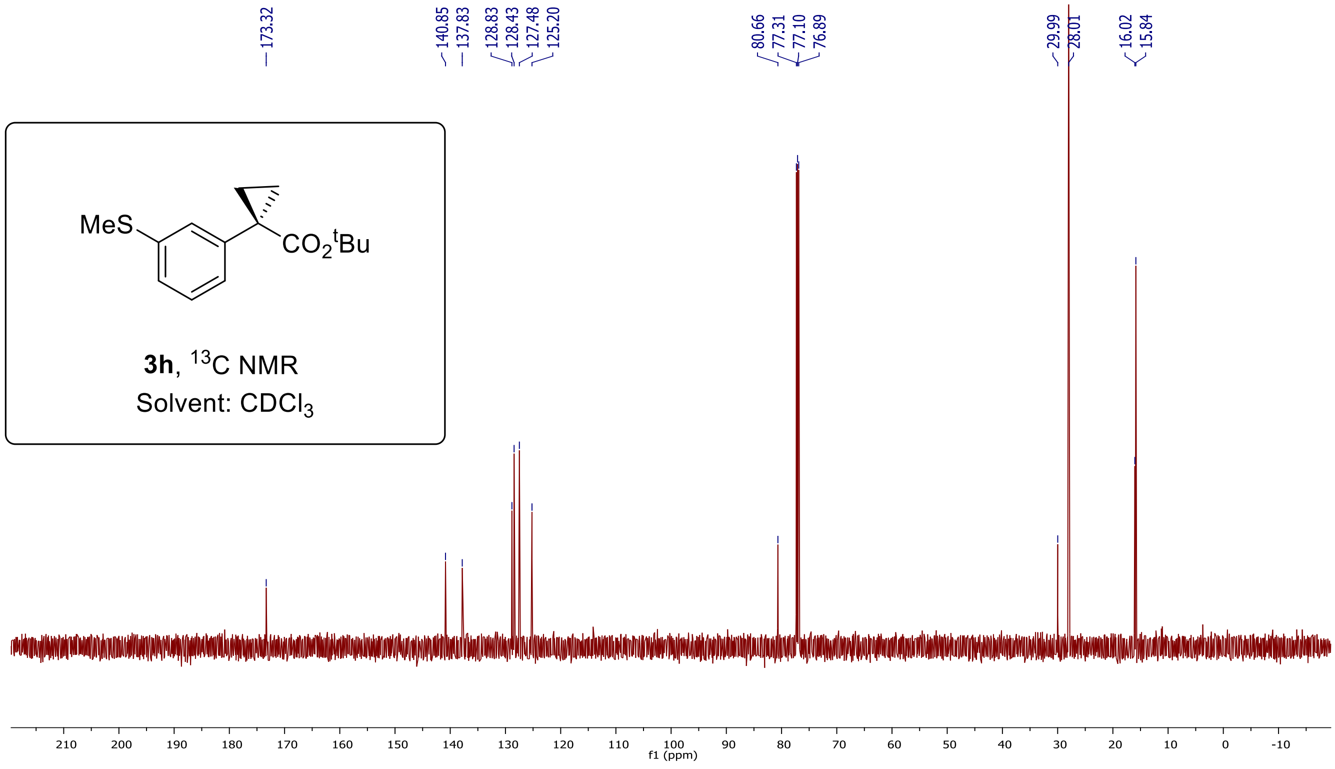

Supplementary Figure 58. NMR spectra of **3h**

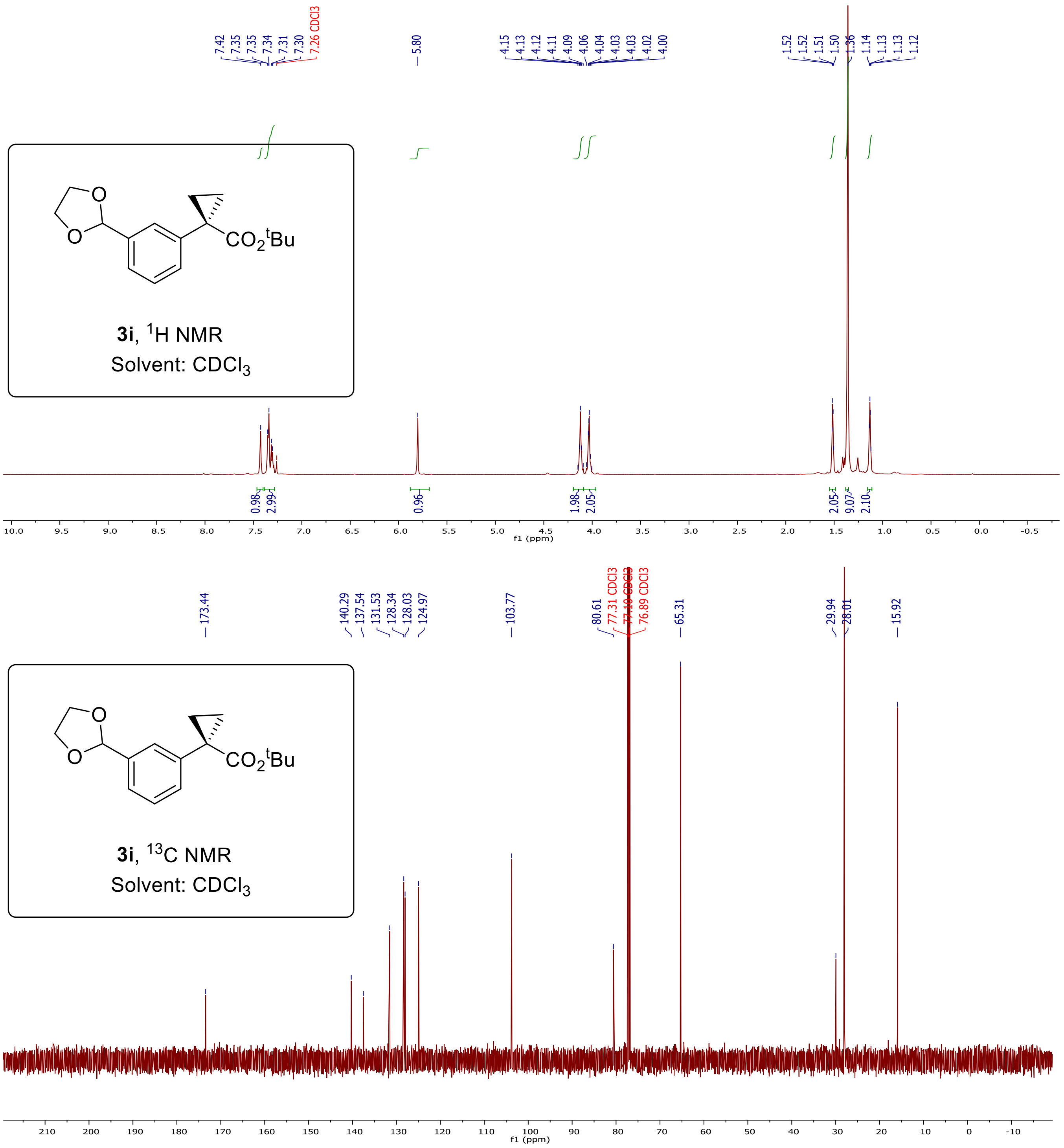

Supplementary Figure 59. NMR spectra of **3i**

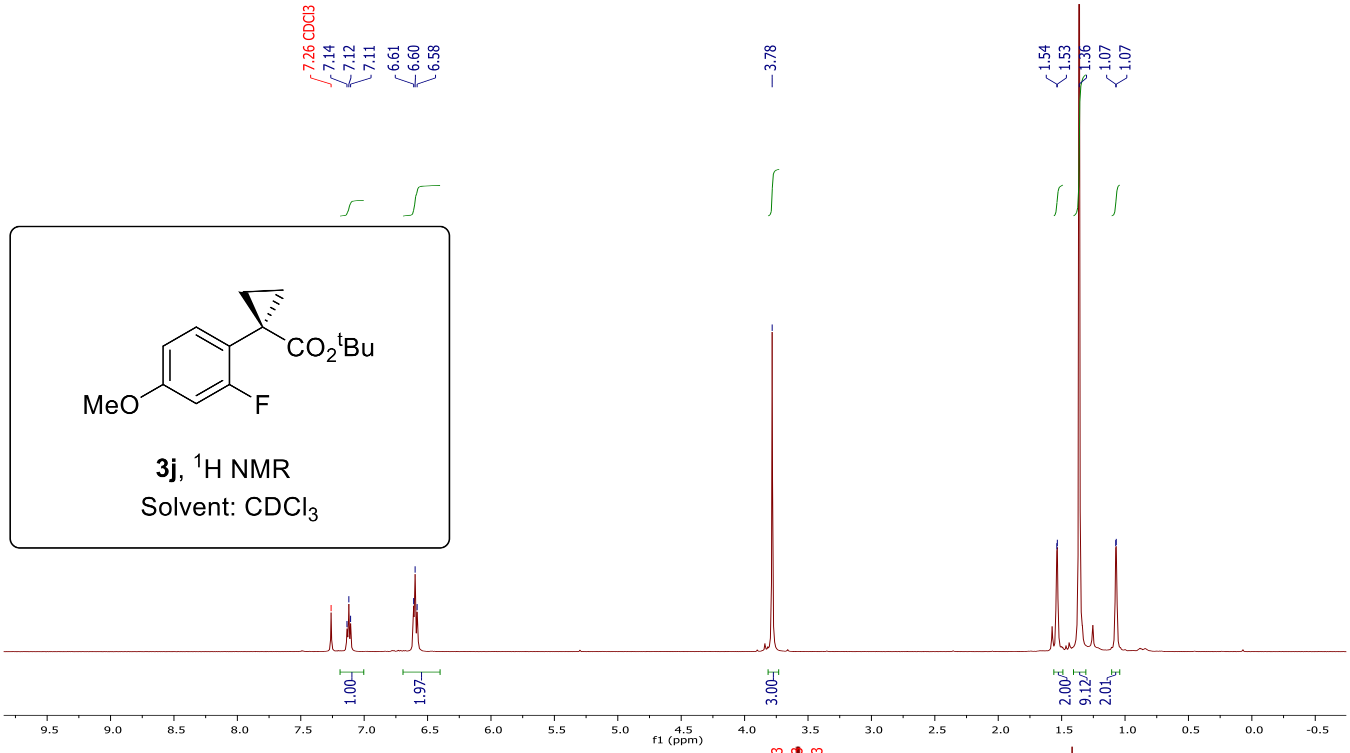

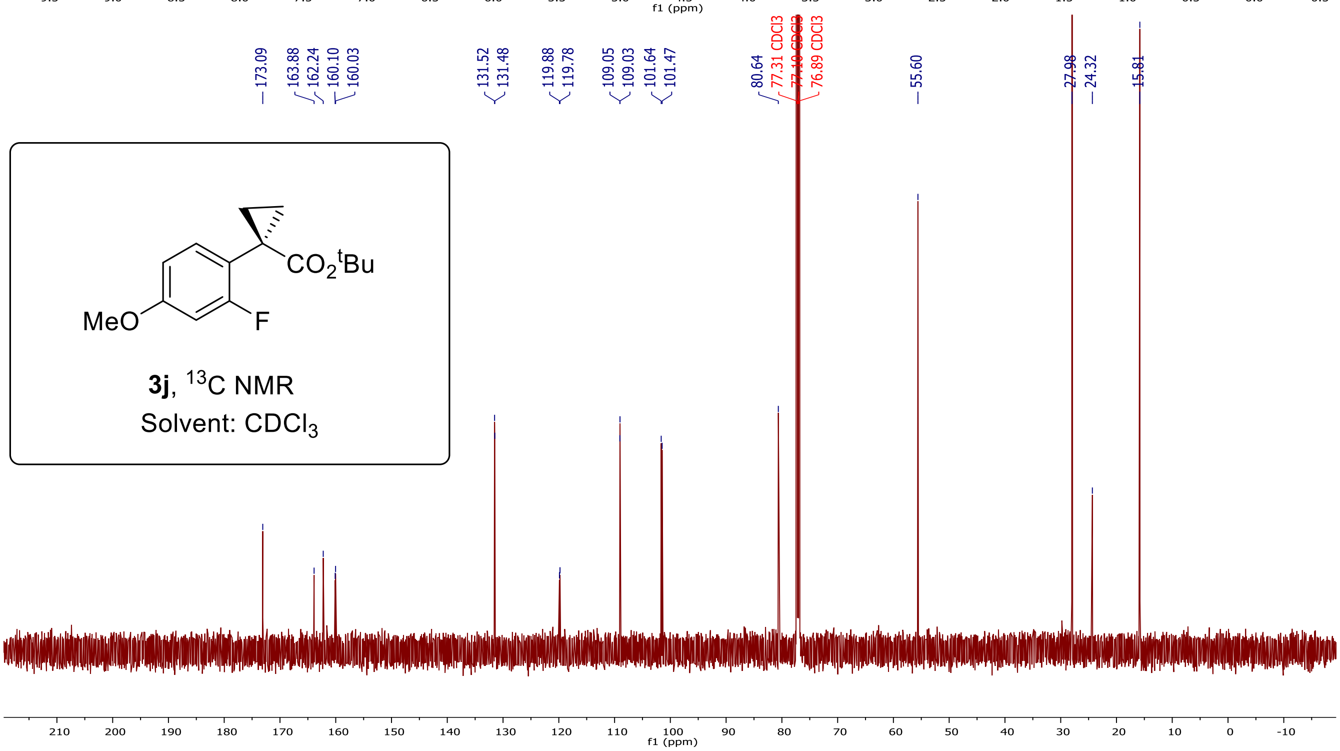

Supplementary Figure 60. NMR spectra of **3j**

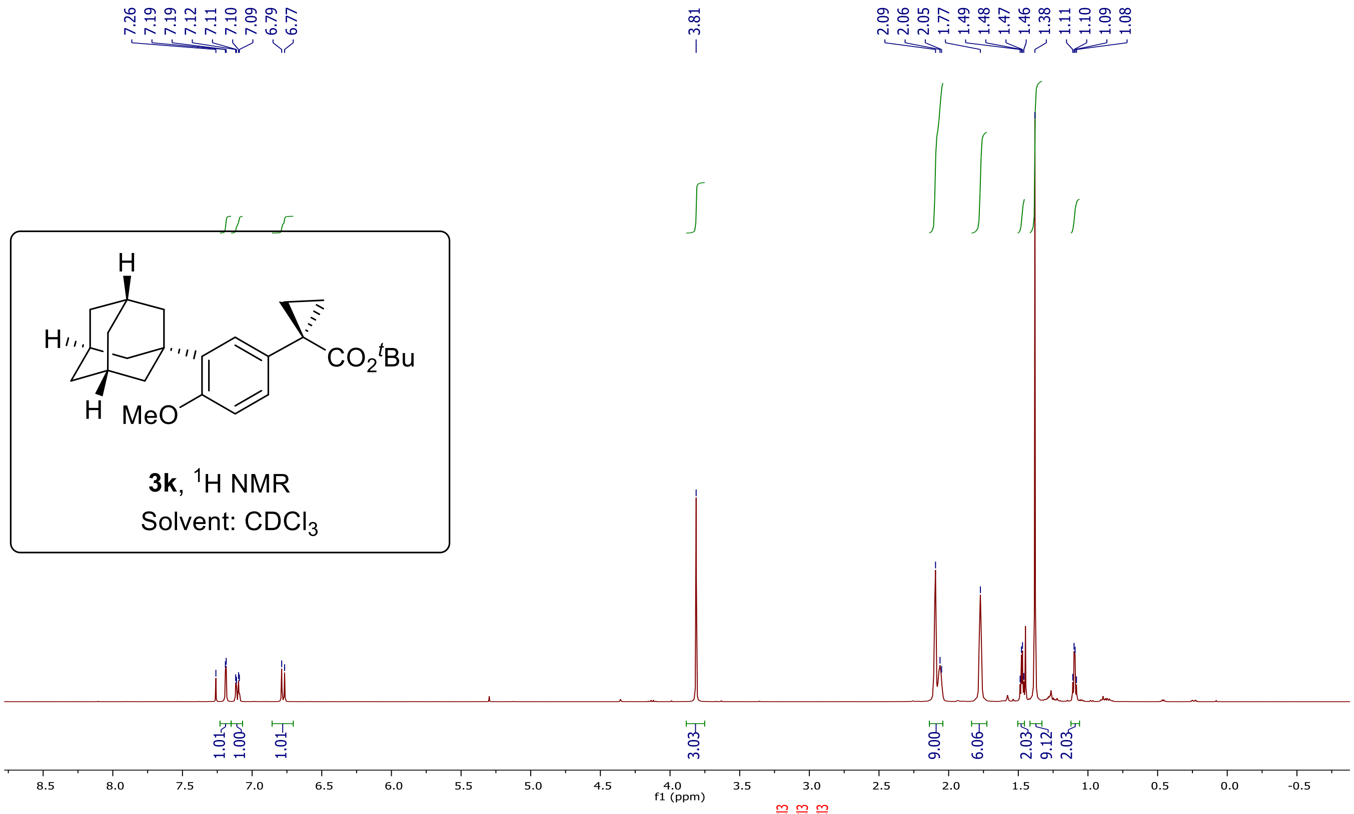

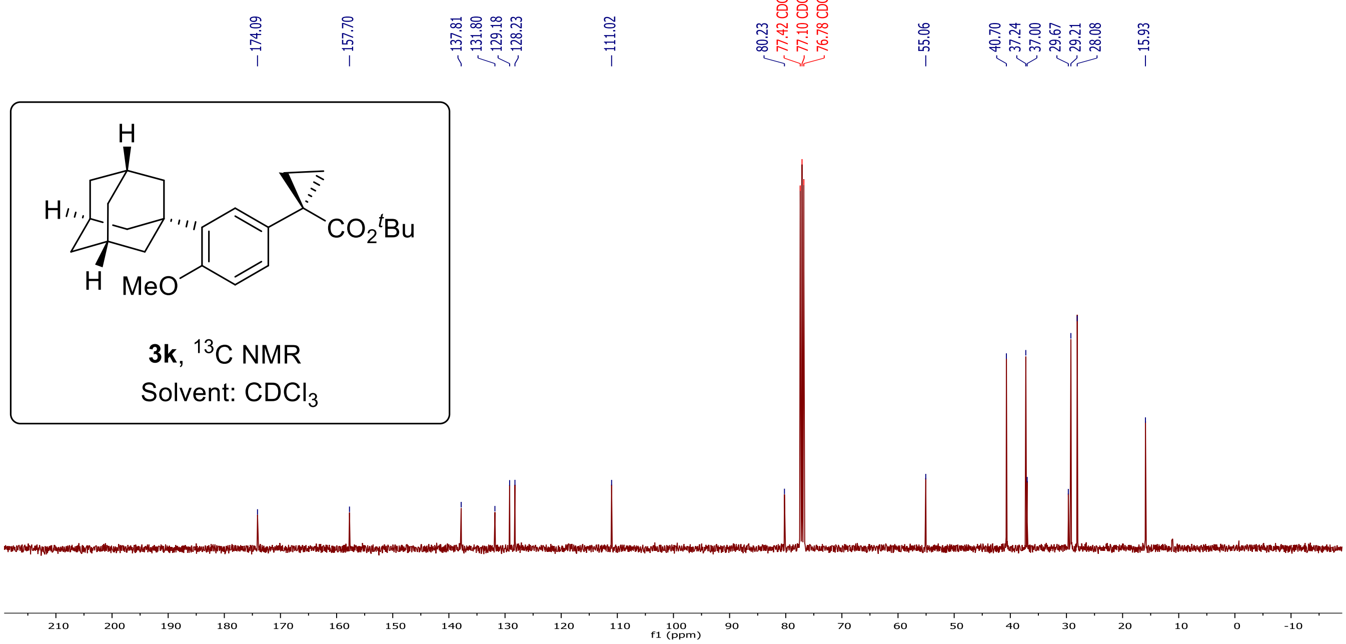

Supplementary Figure 61. NMR spectra of **3k**

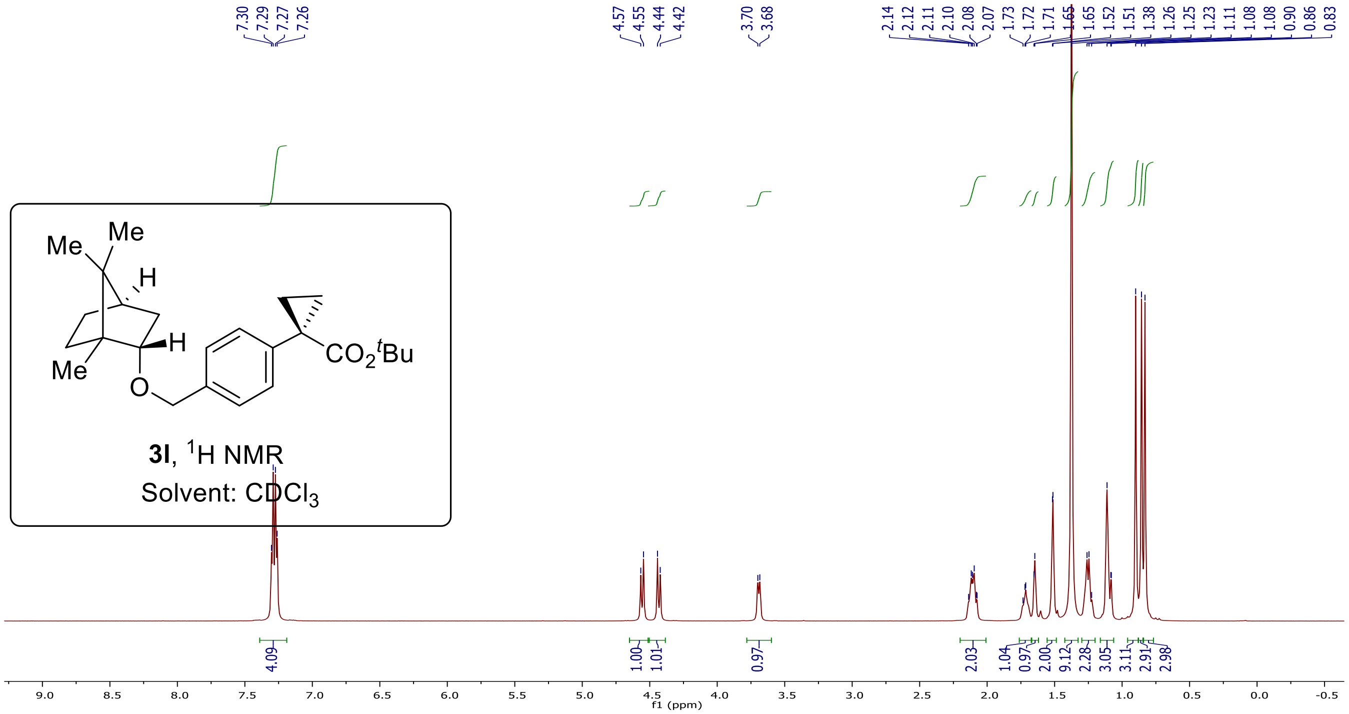

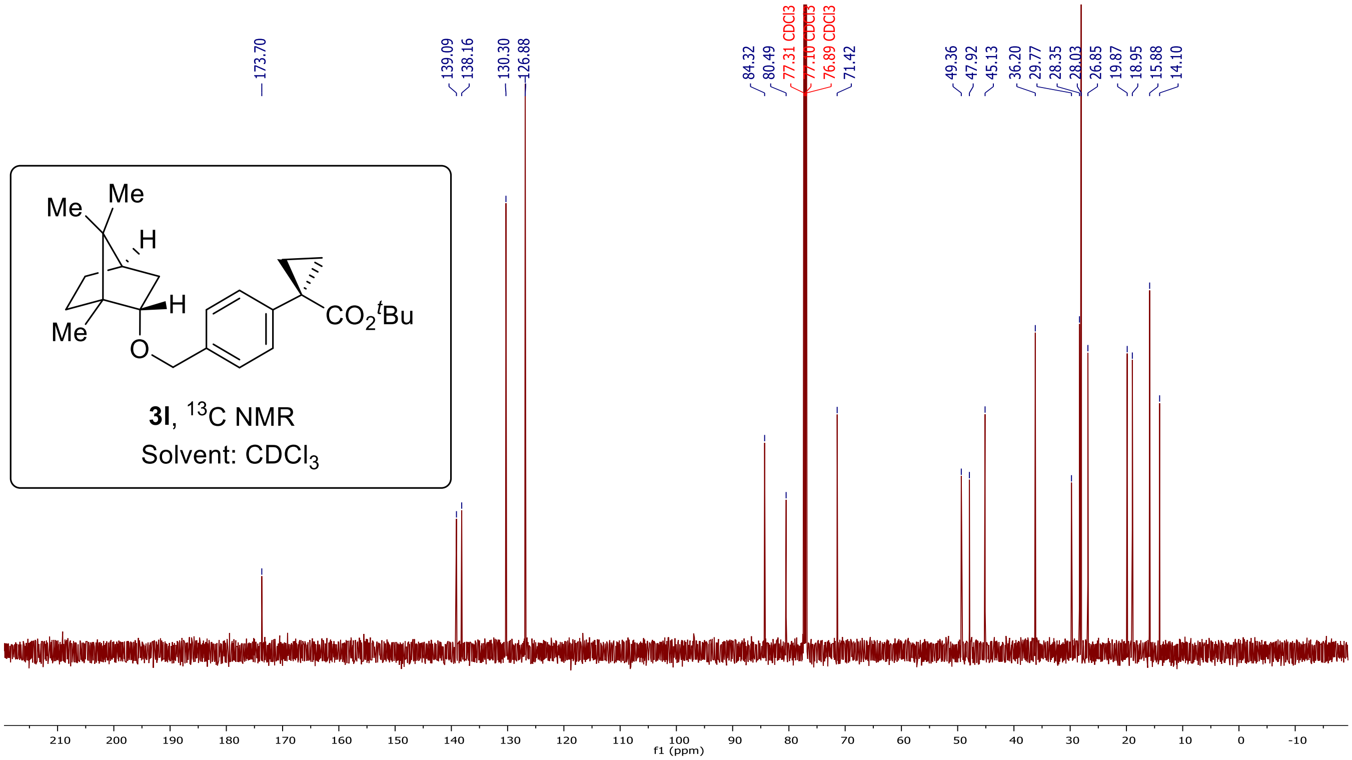

Supplementary Figure 62. NMR spectra of **3l**

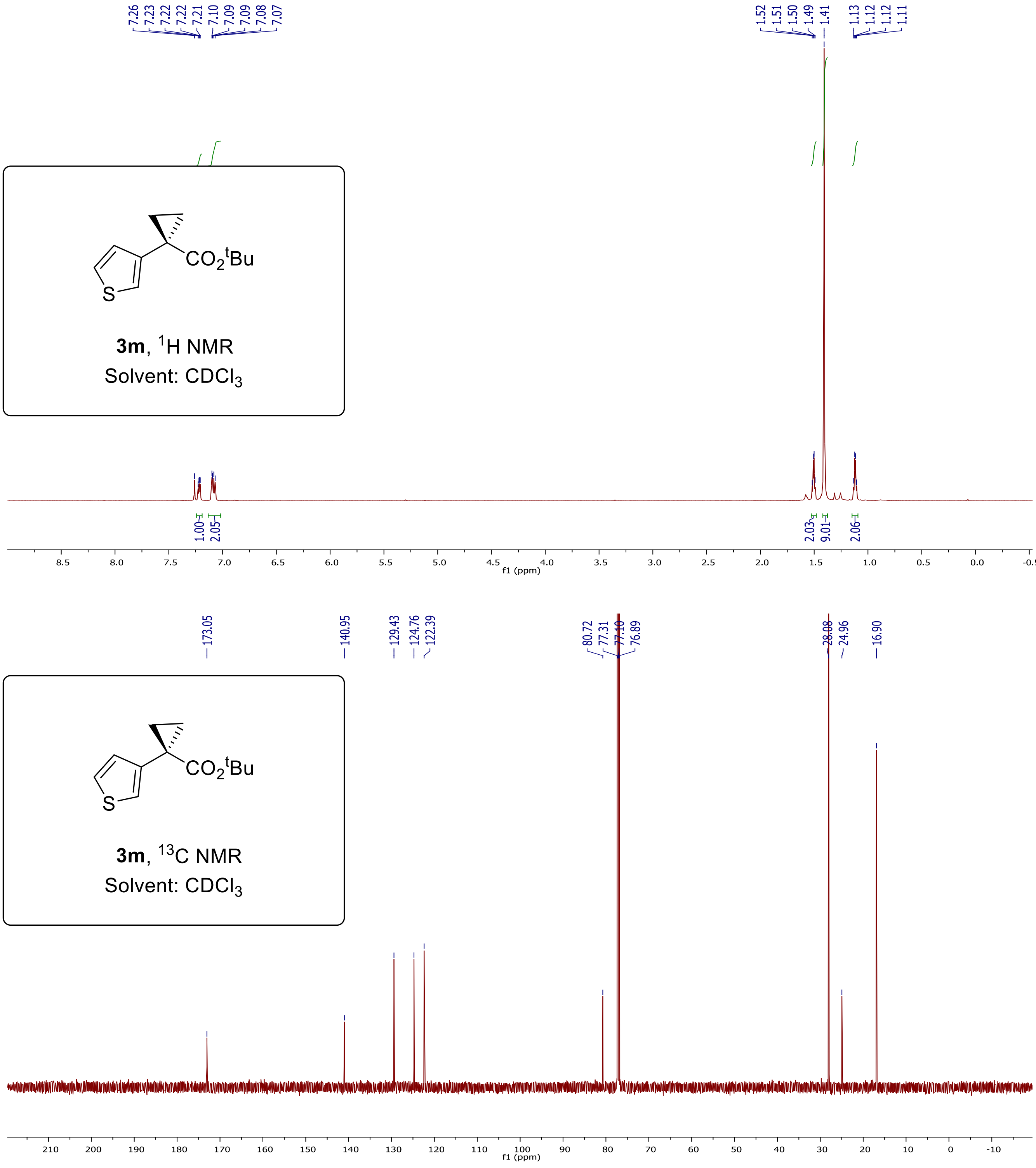

Supplementary Figure 63. NMR spectra of **3m**

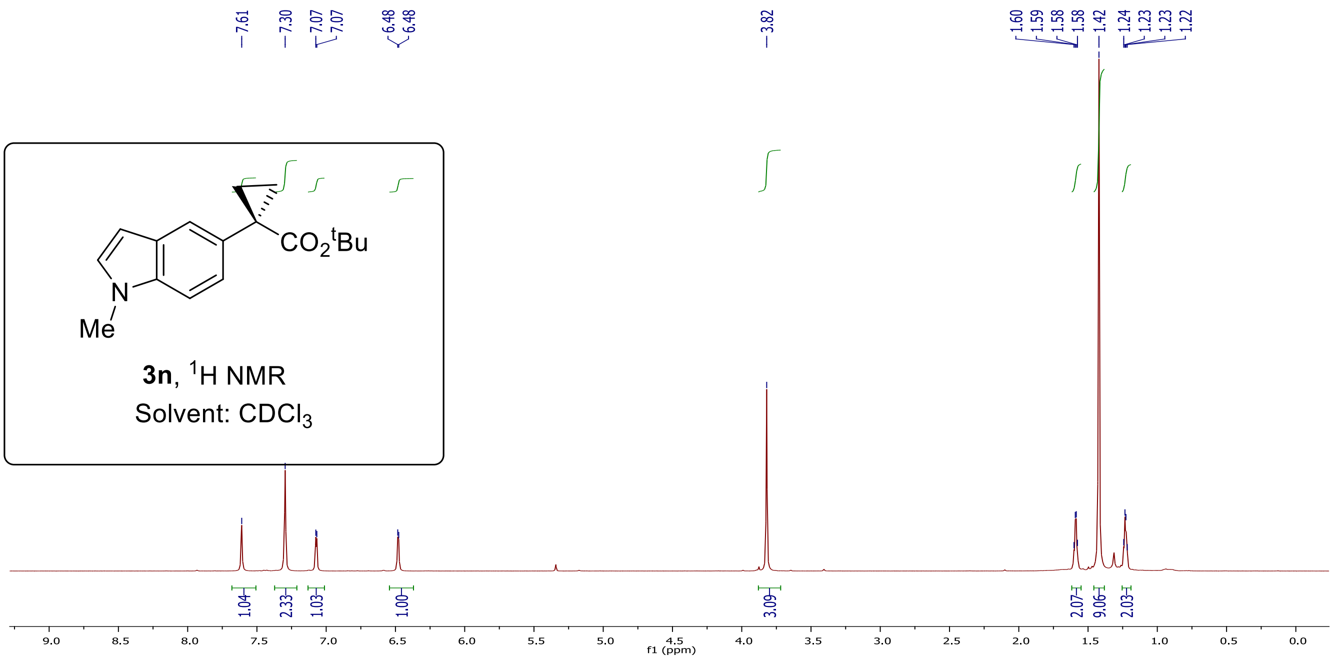

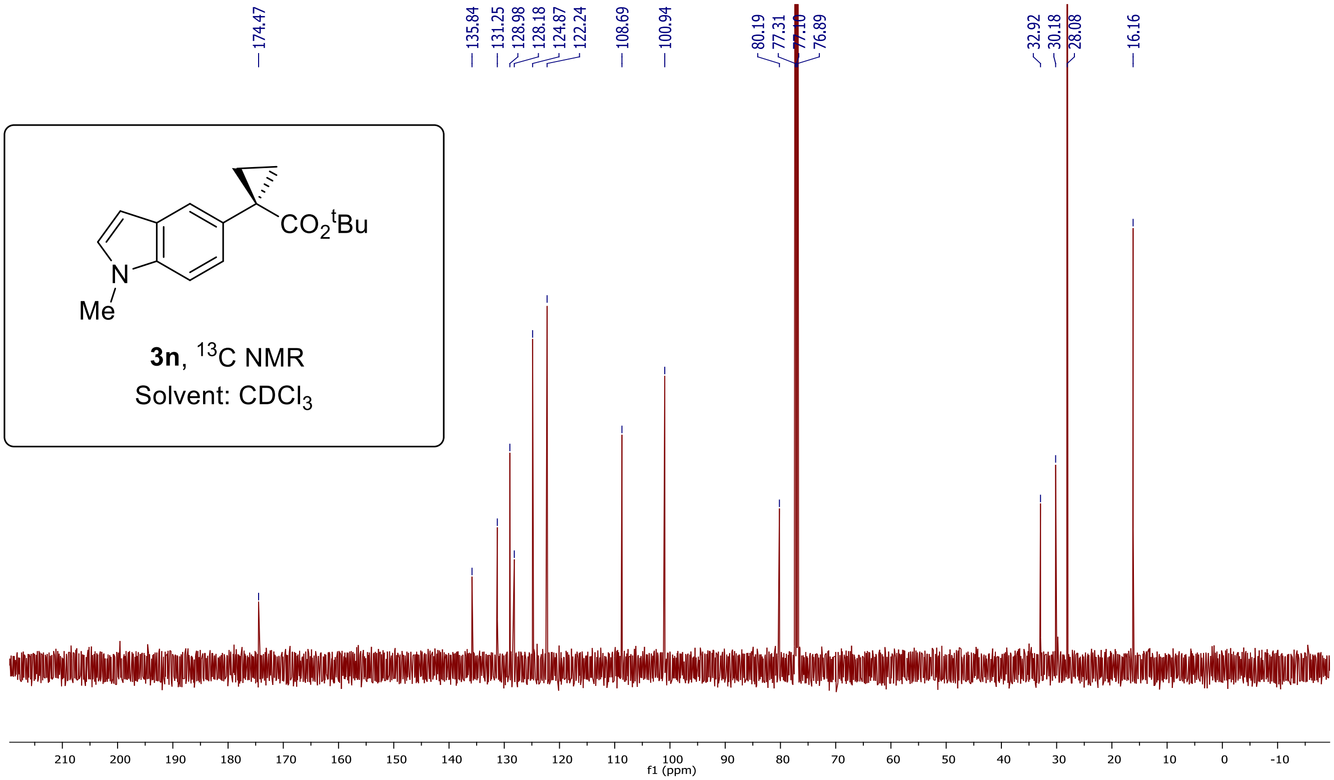

Supplementary Figure 64. NMR spectra of **3n**

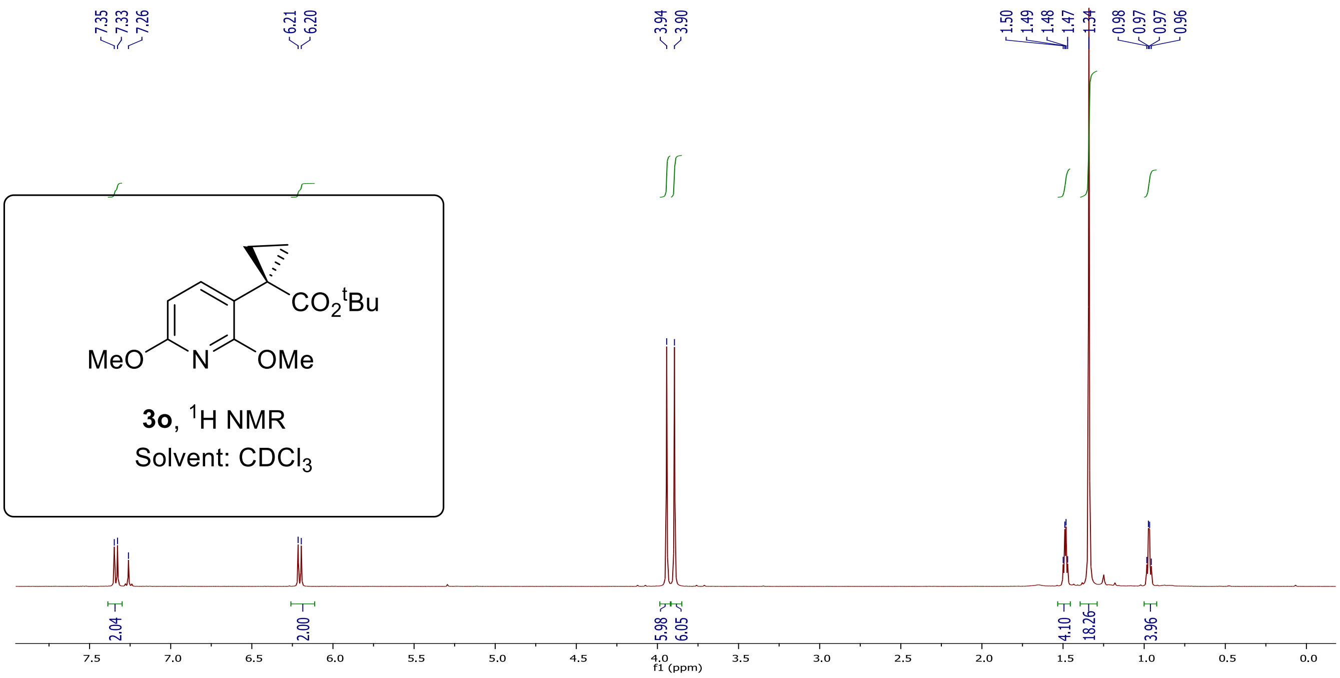

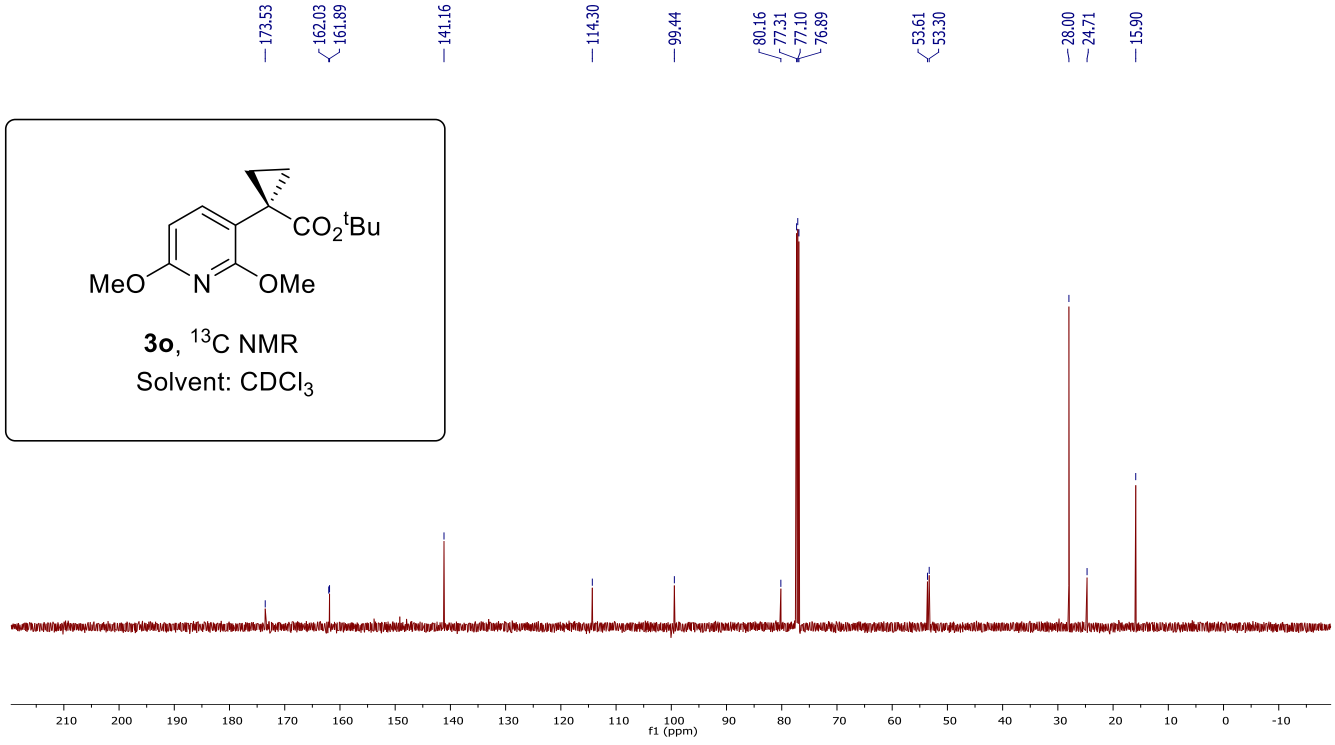

Supplementary Figure 65. NMR spectra of **3o**

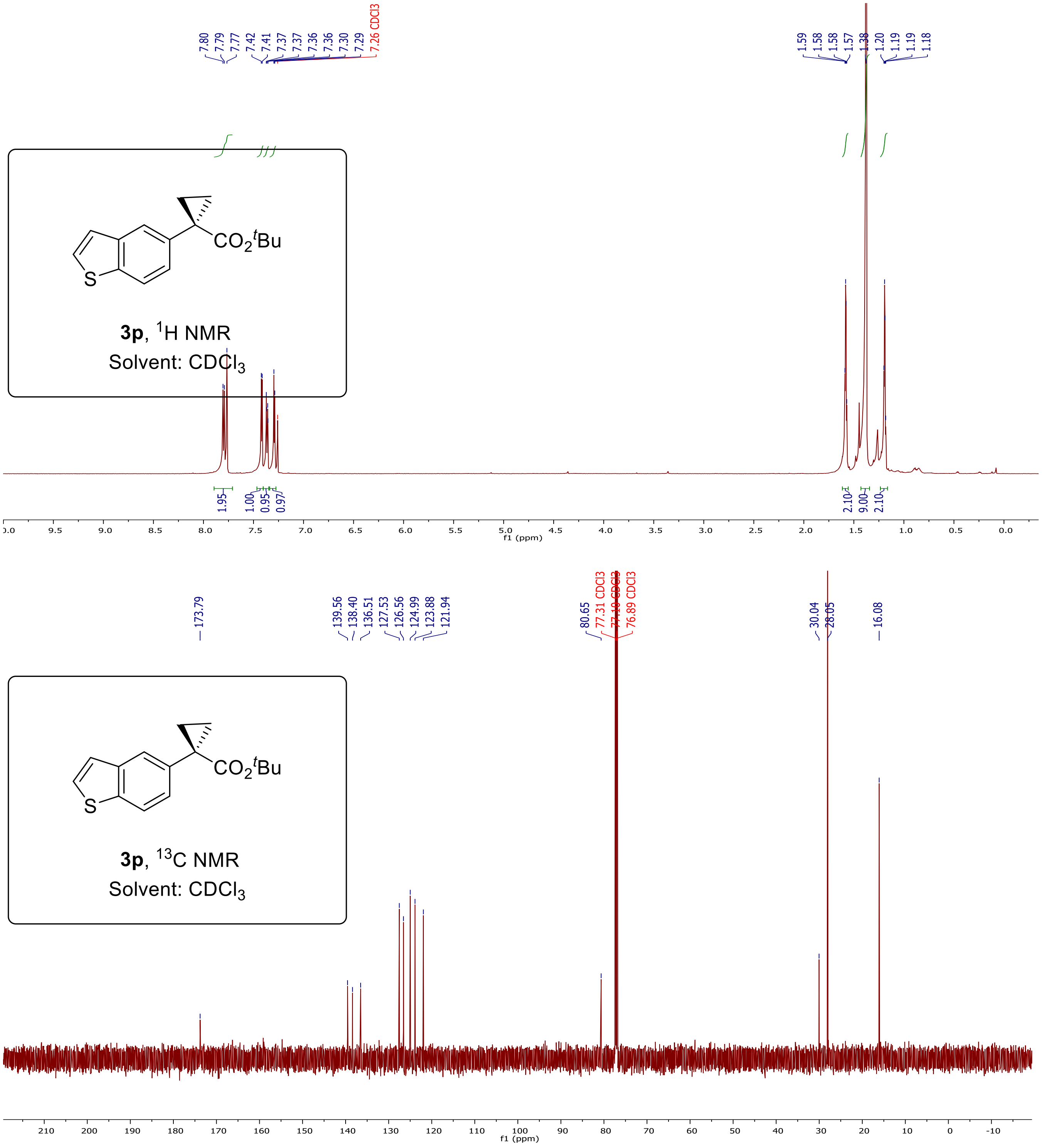

Supplementary Figure 66. NMR spectra of **3p**

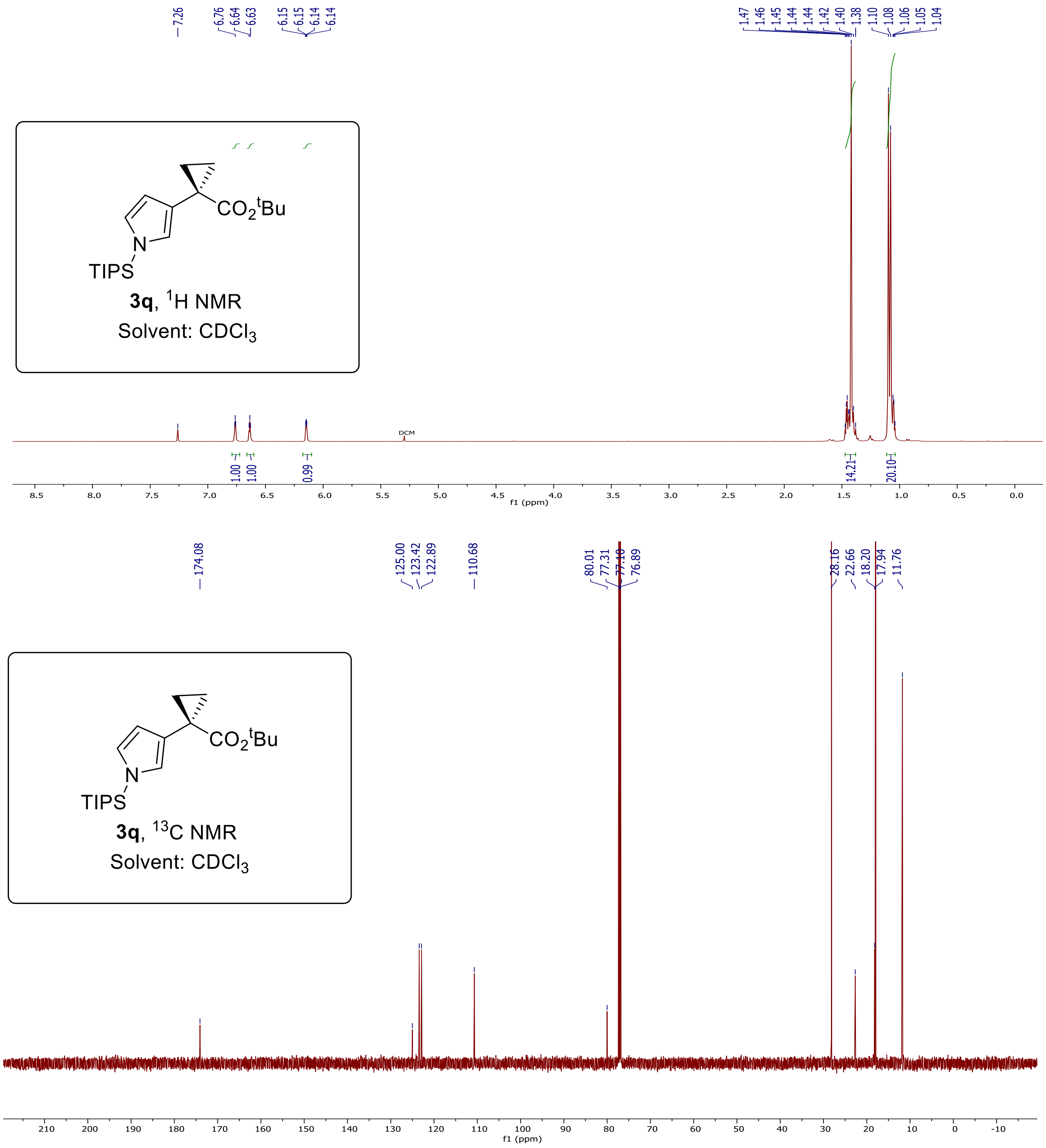

Supplementary Figure 67. NMR spectra of **3q**

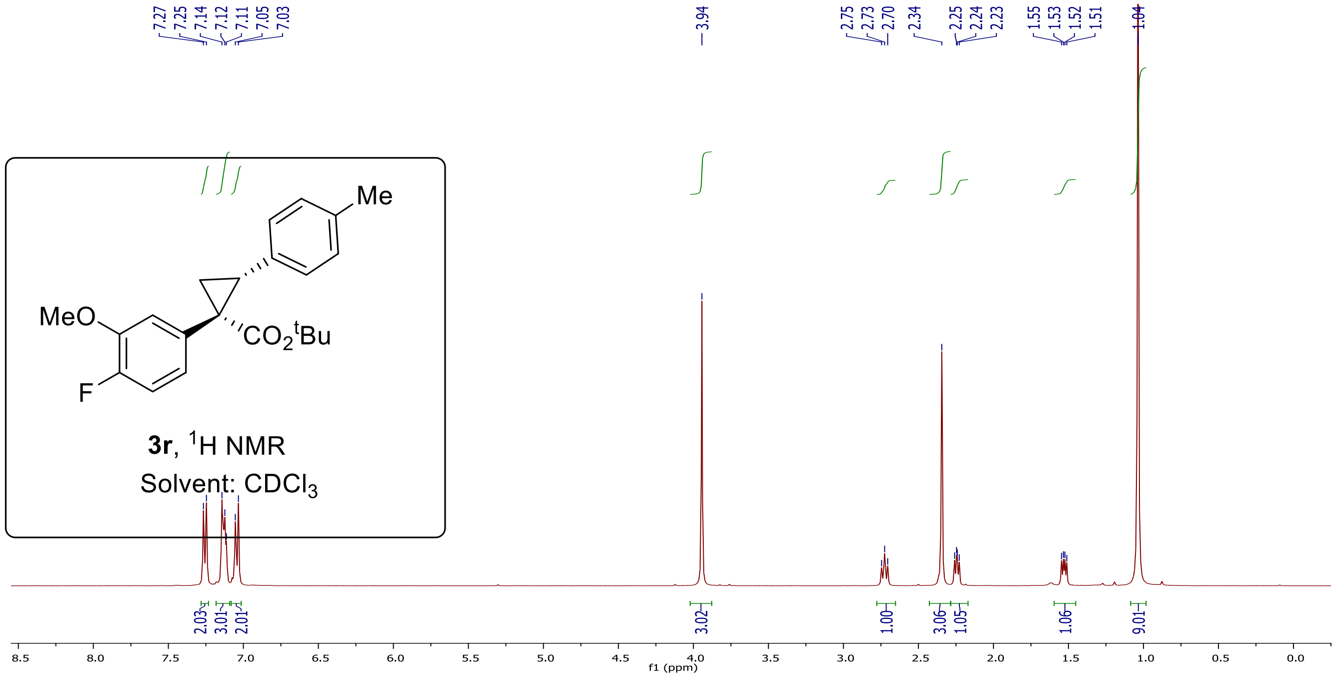

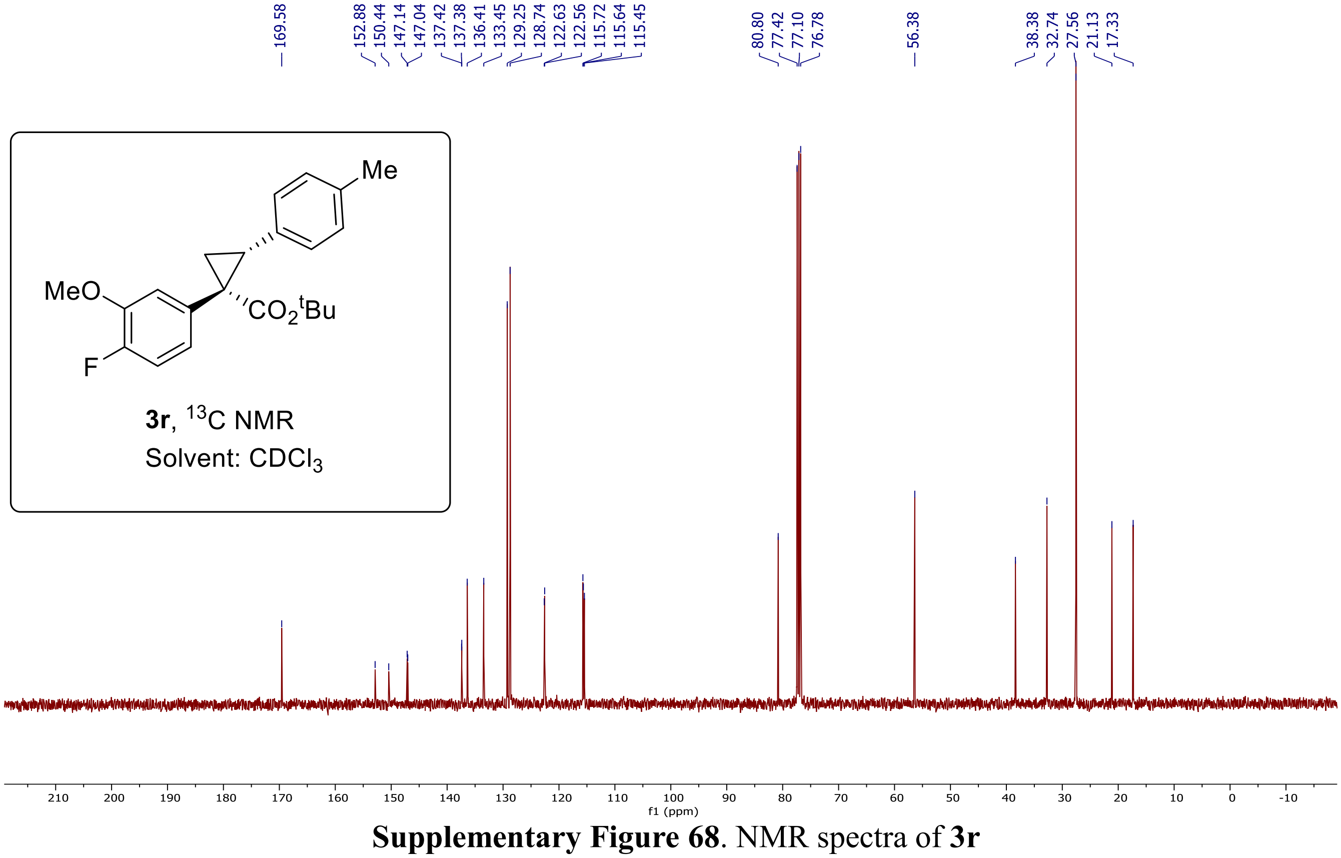

Supplementary Figure 68. NMR spectra of **3r**

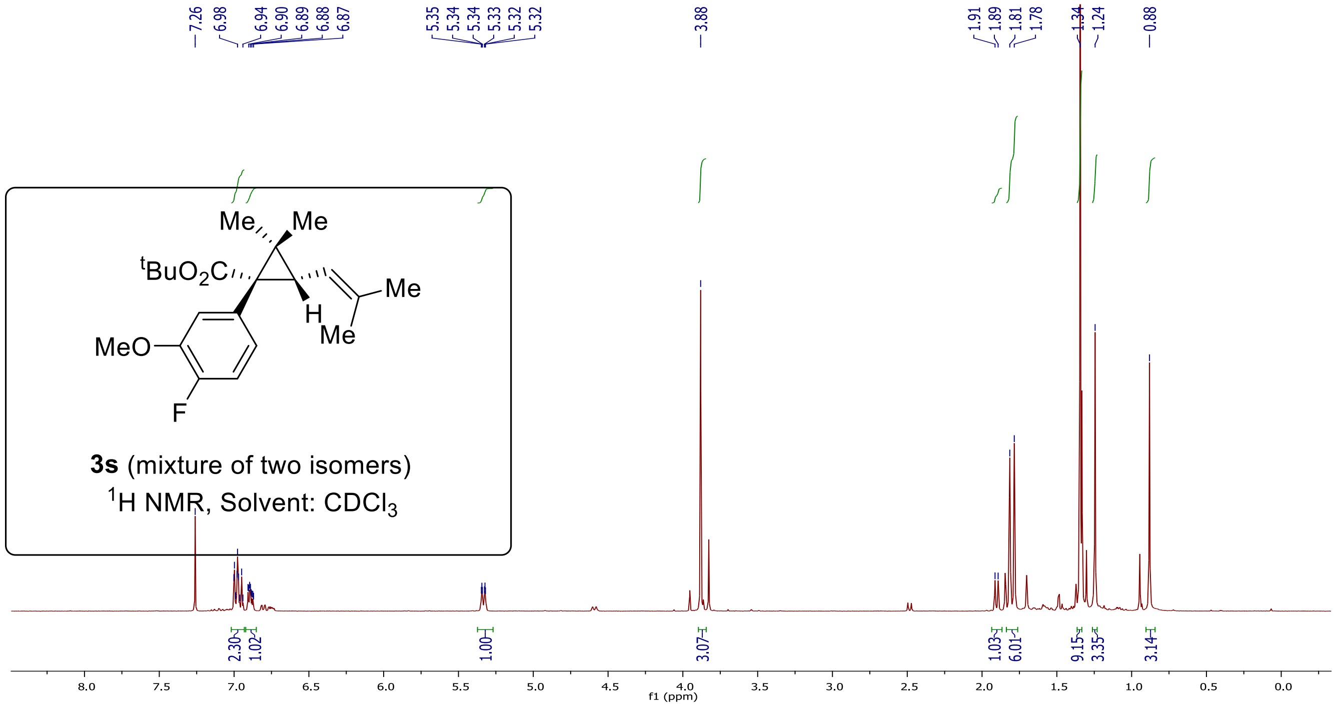

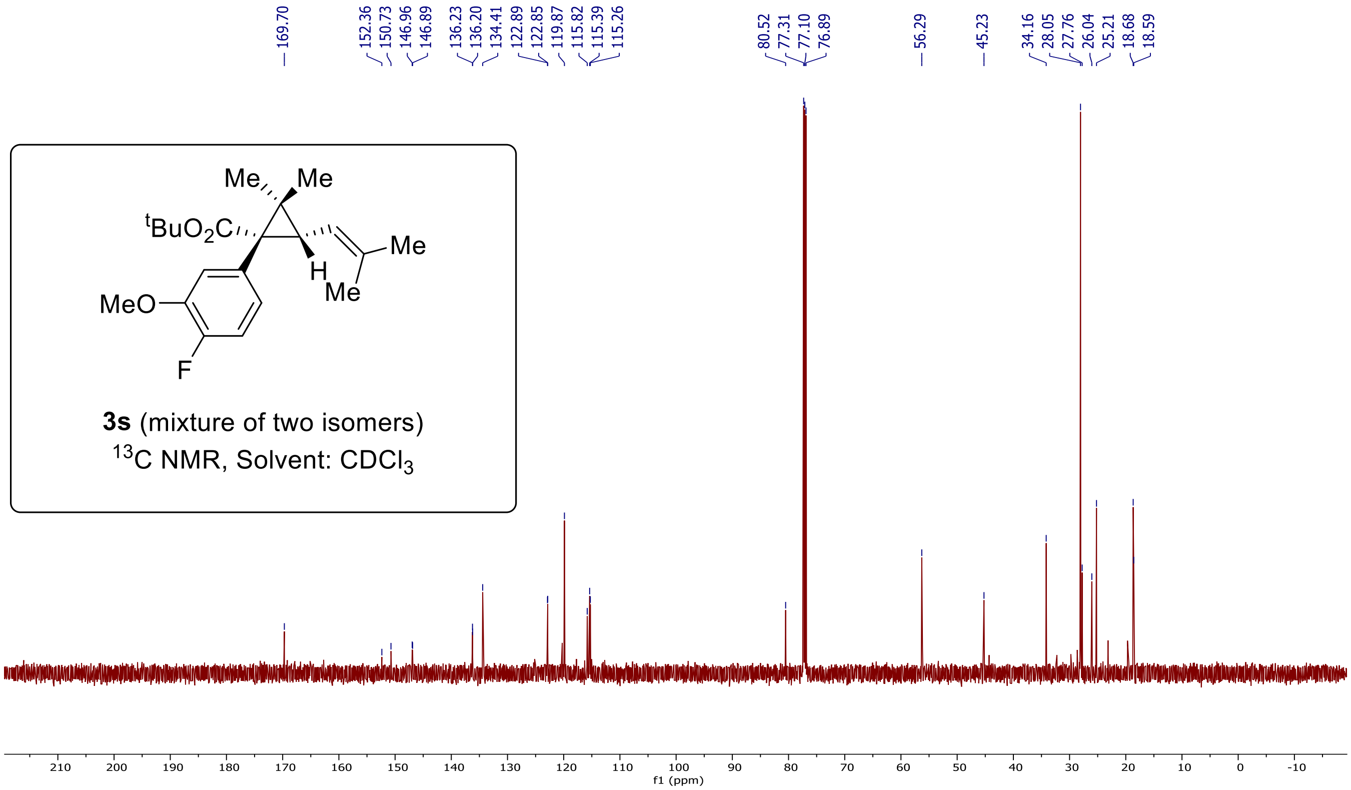

Supplementary Figure 69. NMR spectra of **3s**

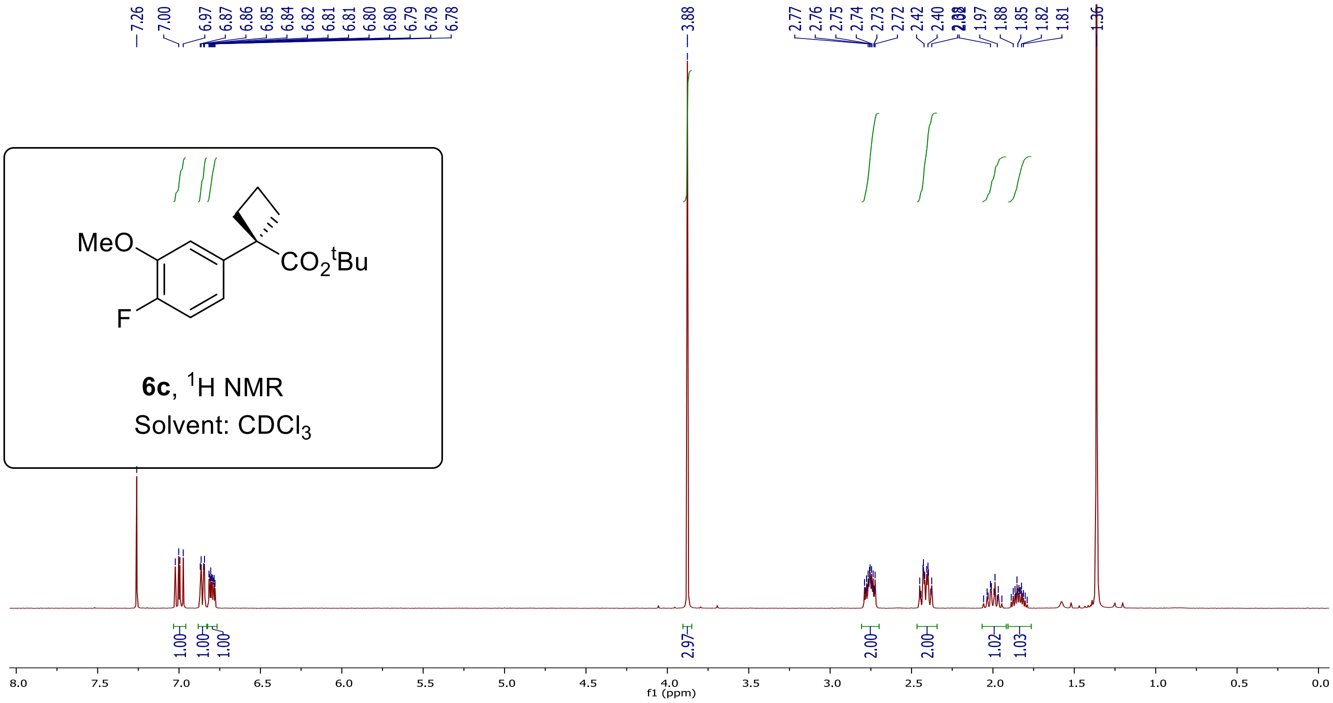

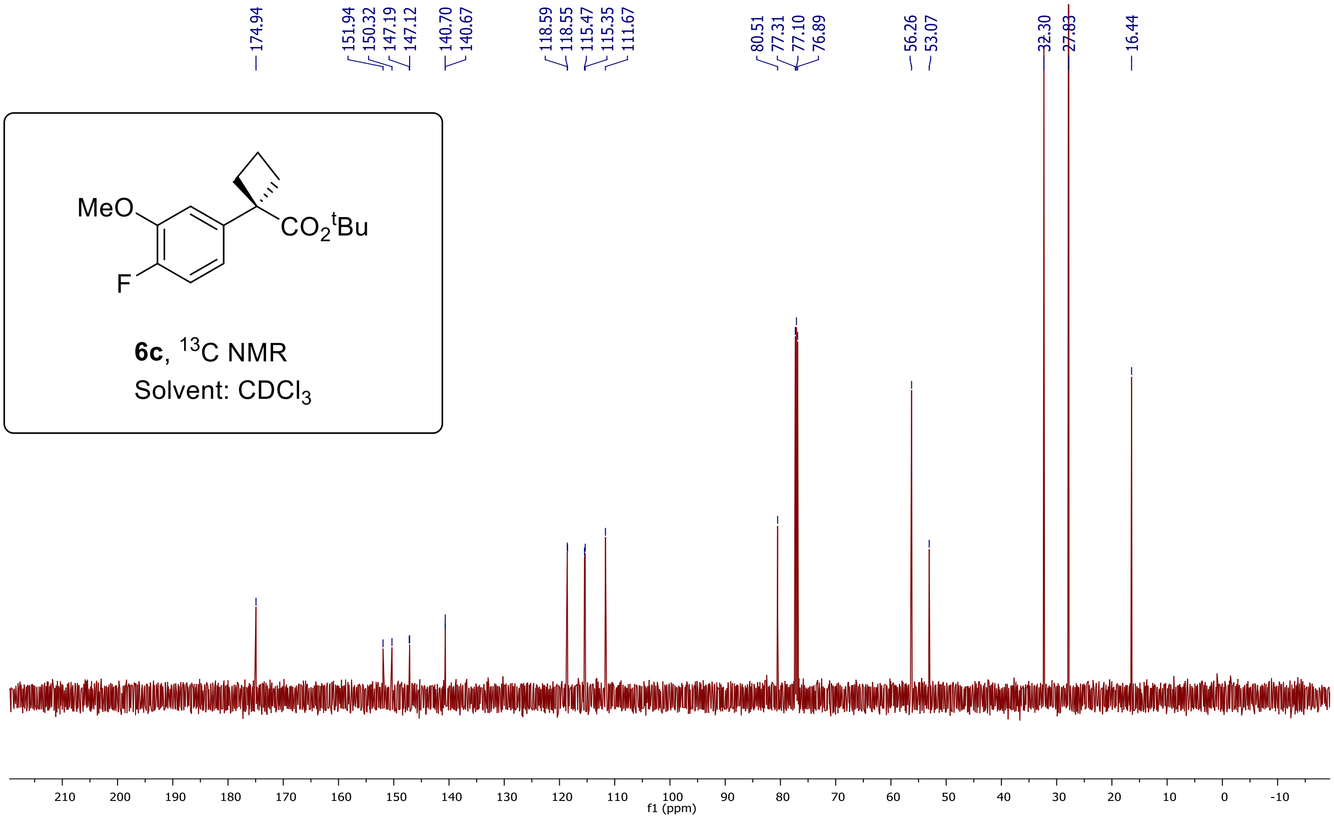

Supplementary Figure 70. NMR spectra of **6c**

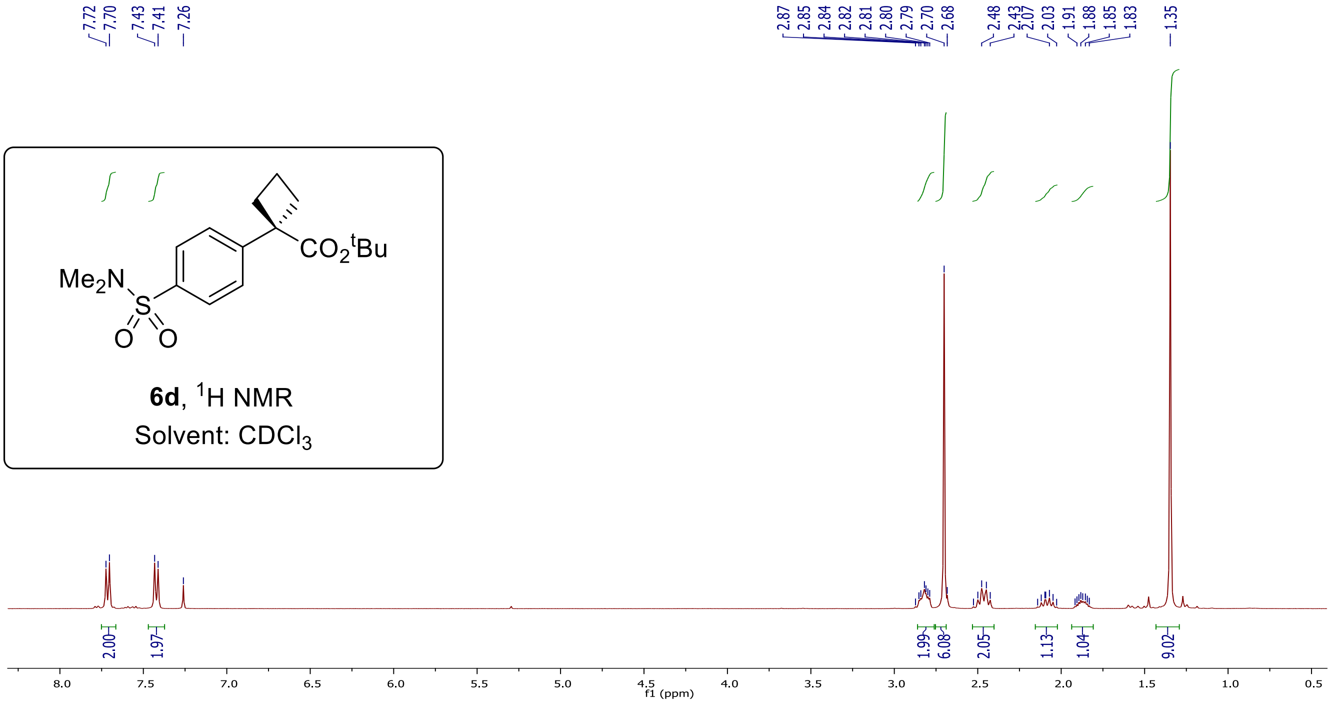

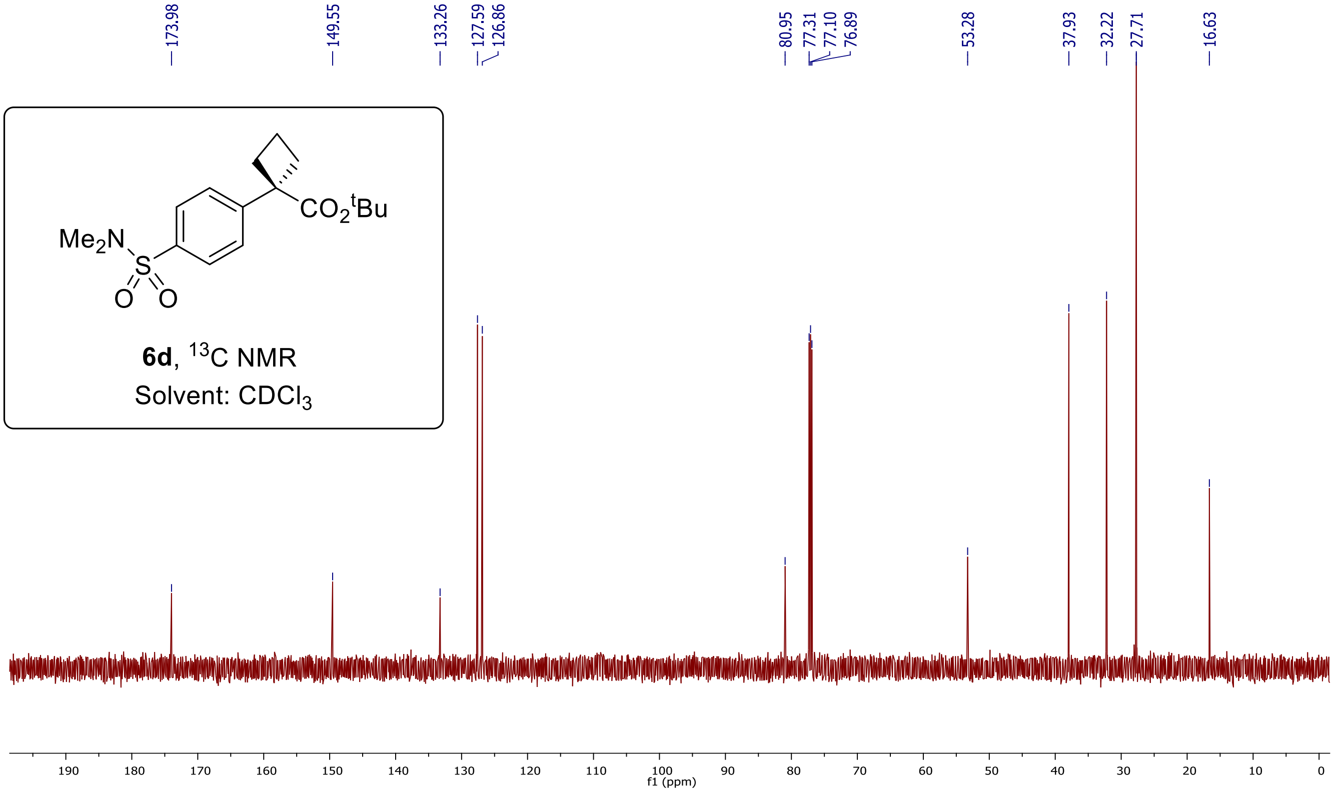

Supplementary Figure 71. NMR spectra of **6d**

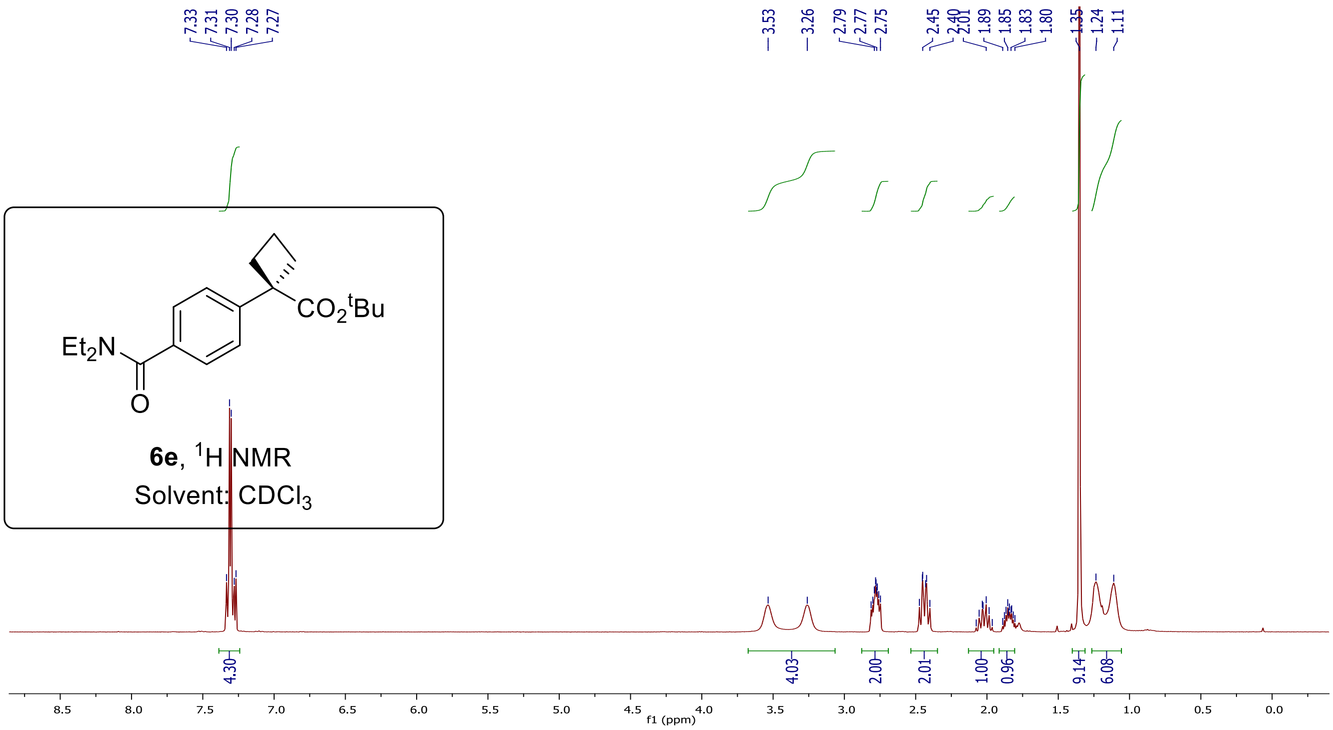

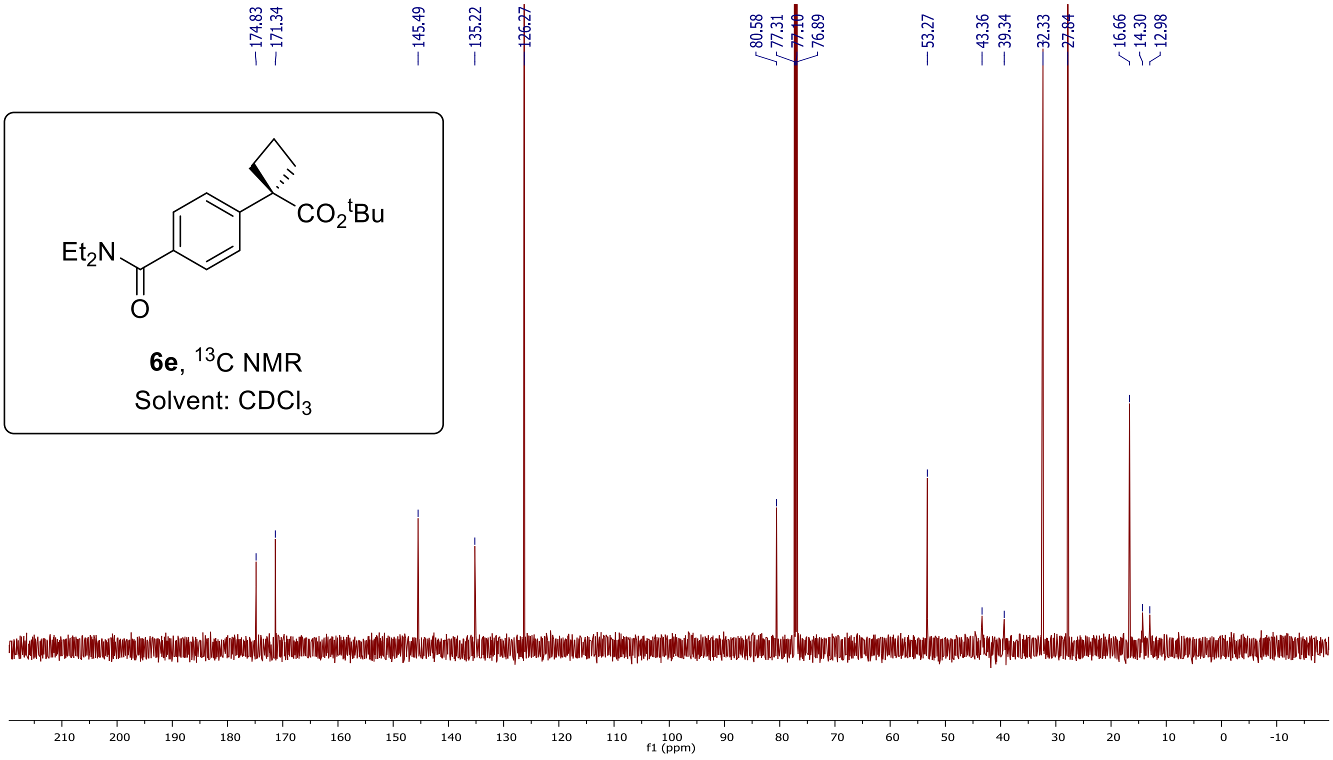

Supplementary Figure 72. NMR spectra of **6e**

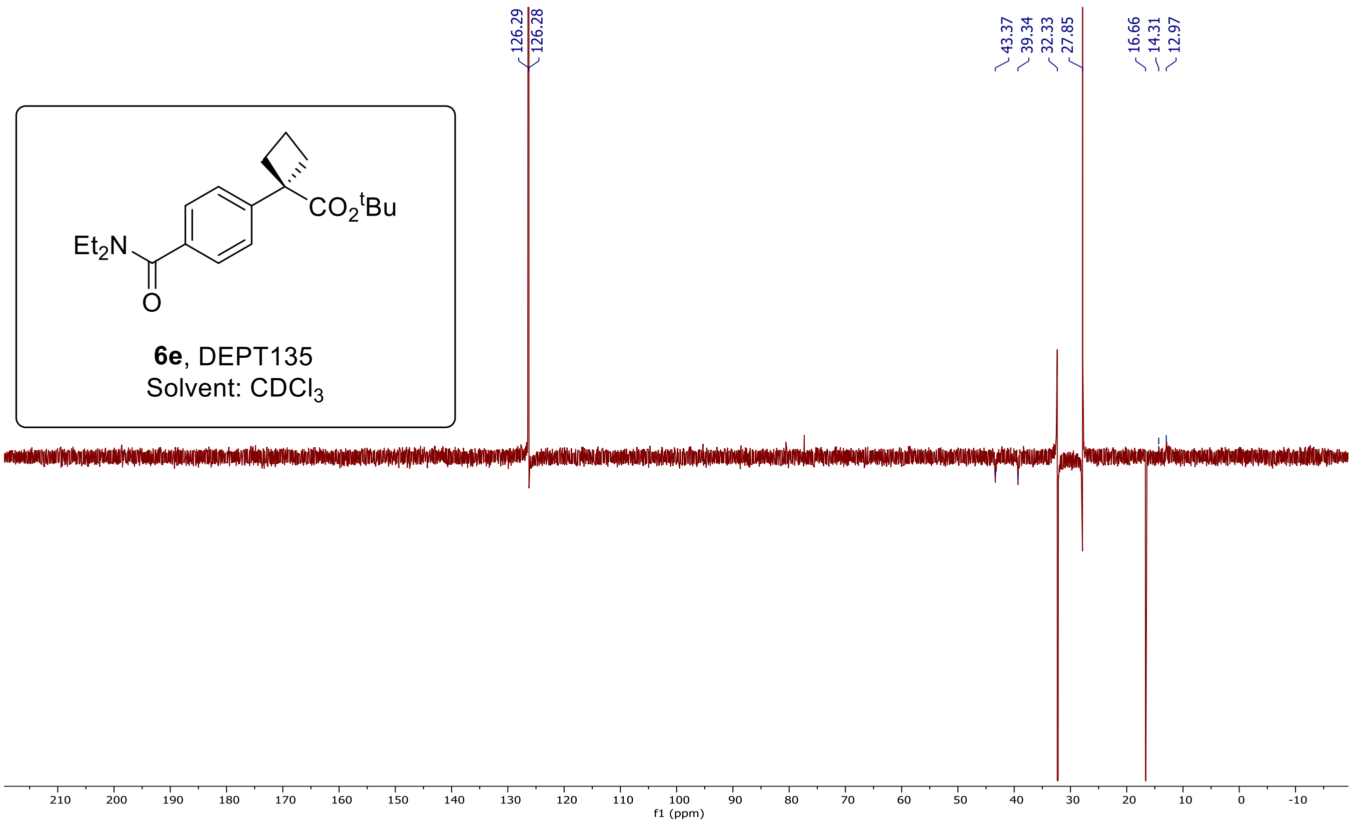

Supplementary Figure 73. DEPT135 spectrum of **6e**

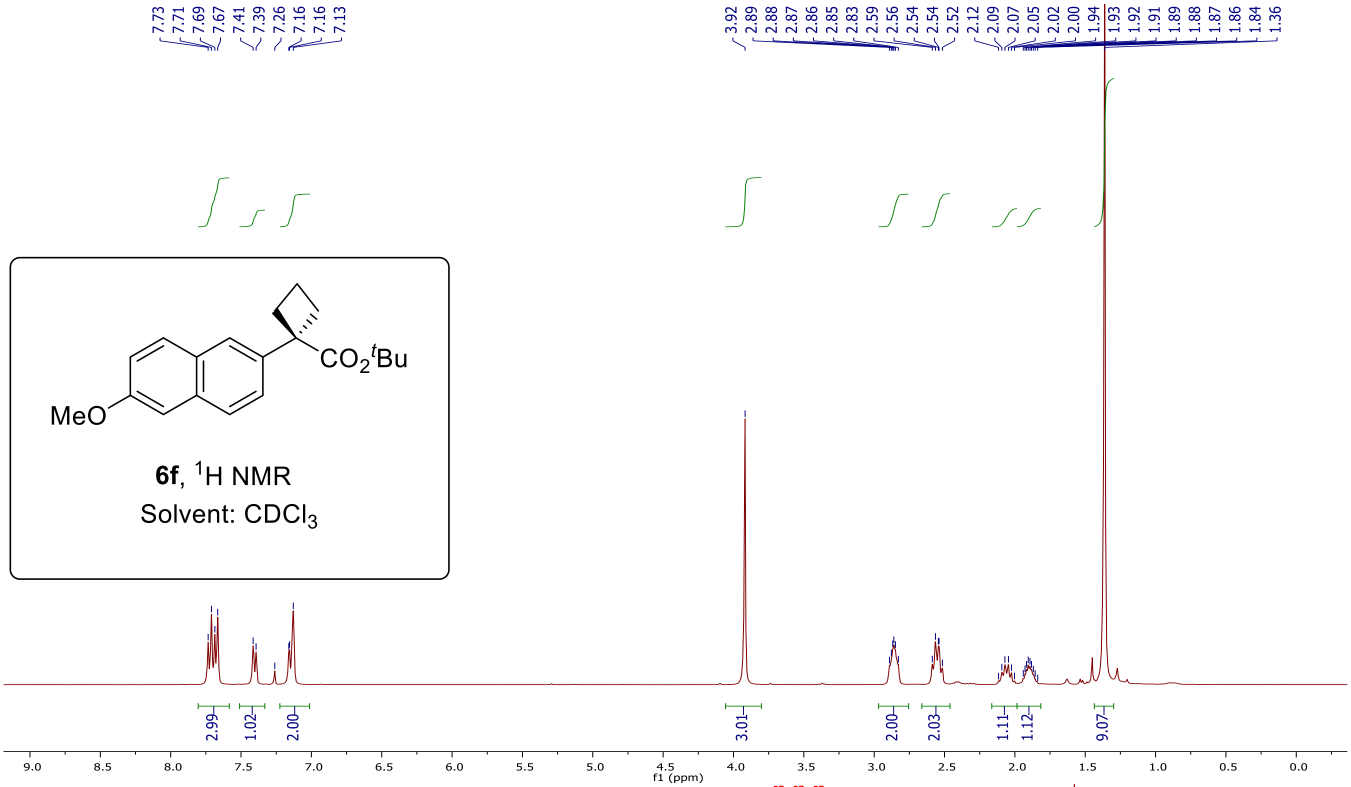

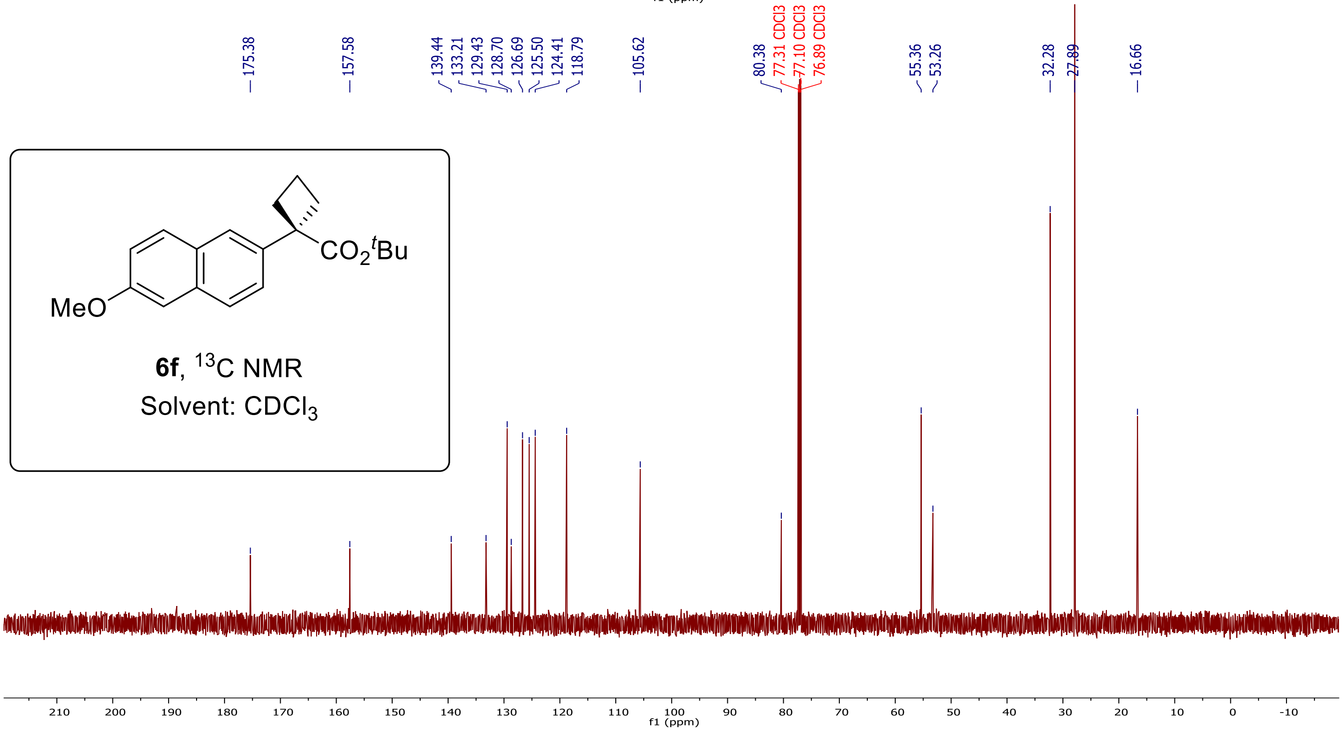

Supplementary Figure 74. NMR spectra of **6f**

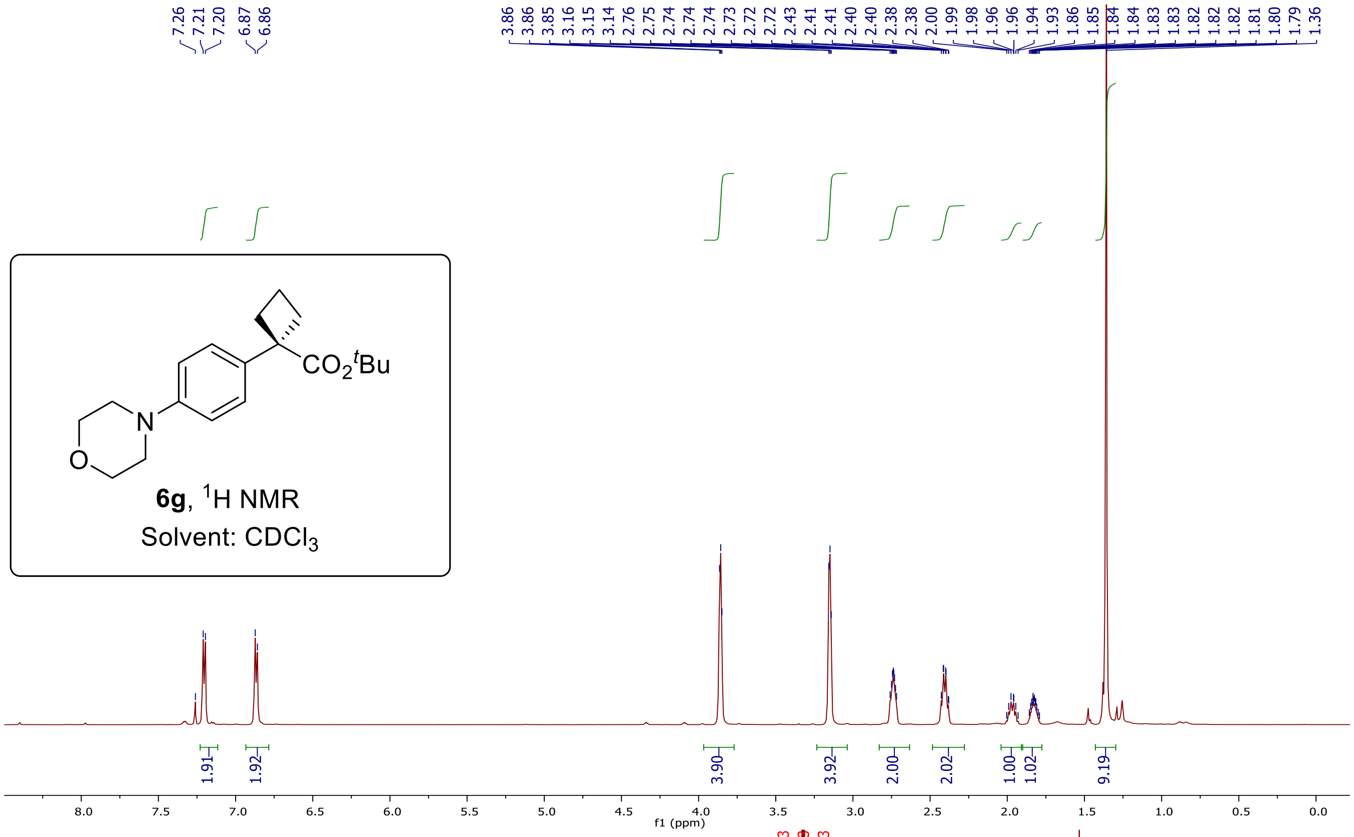

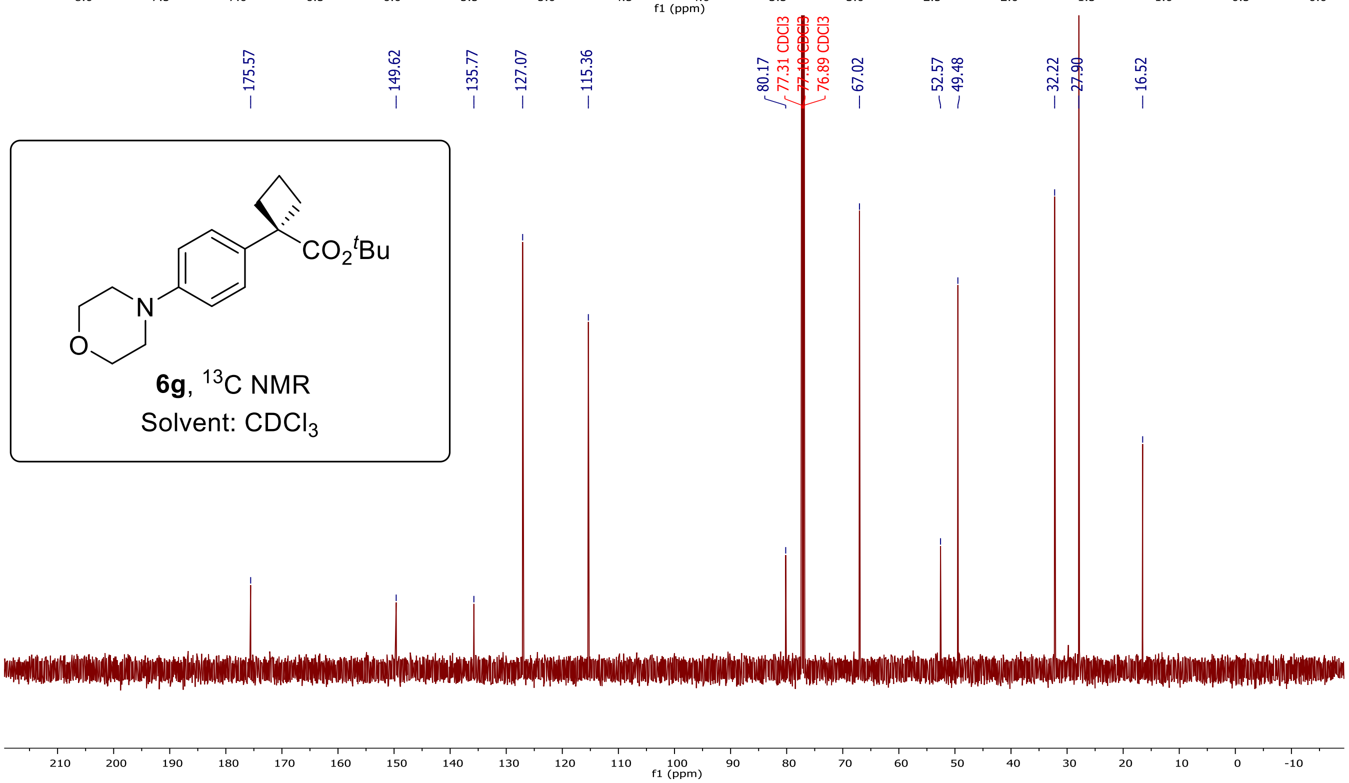

Supplementary Figure 75. NMR spectra of **6g**

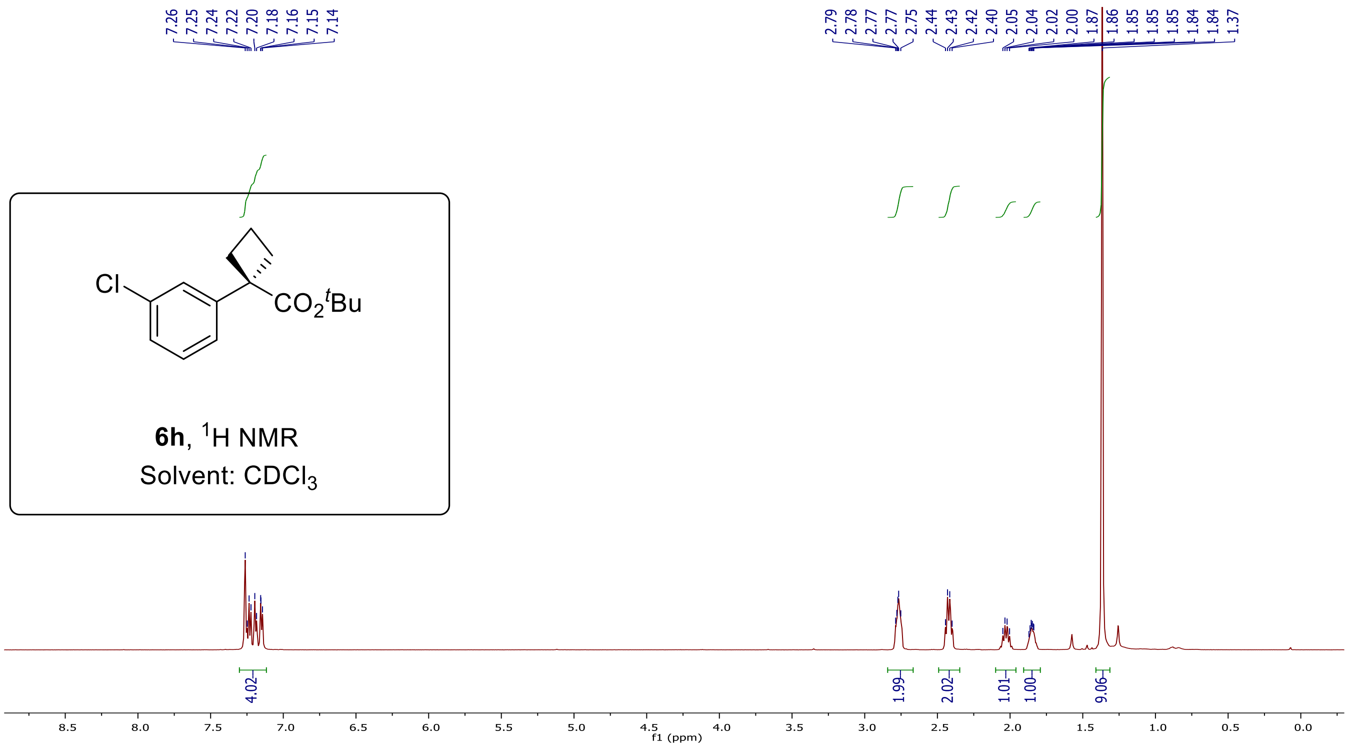

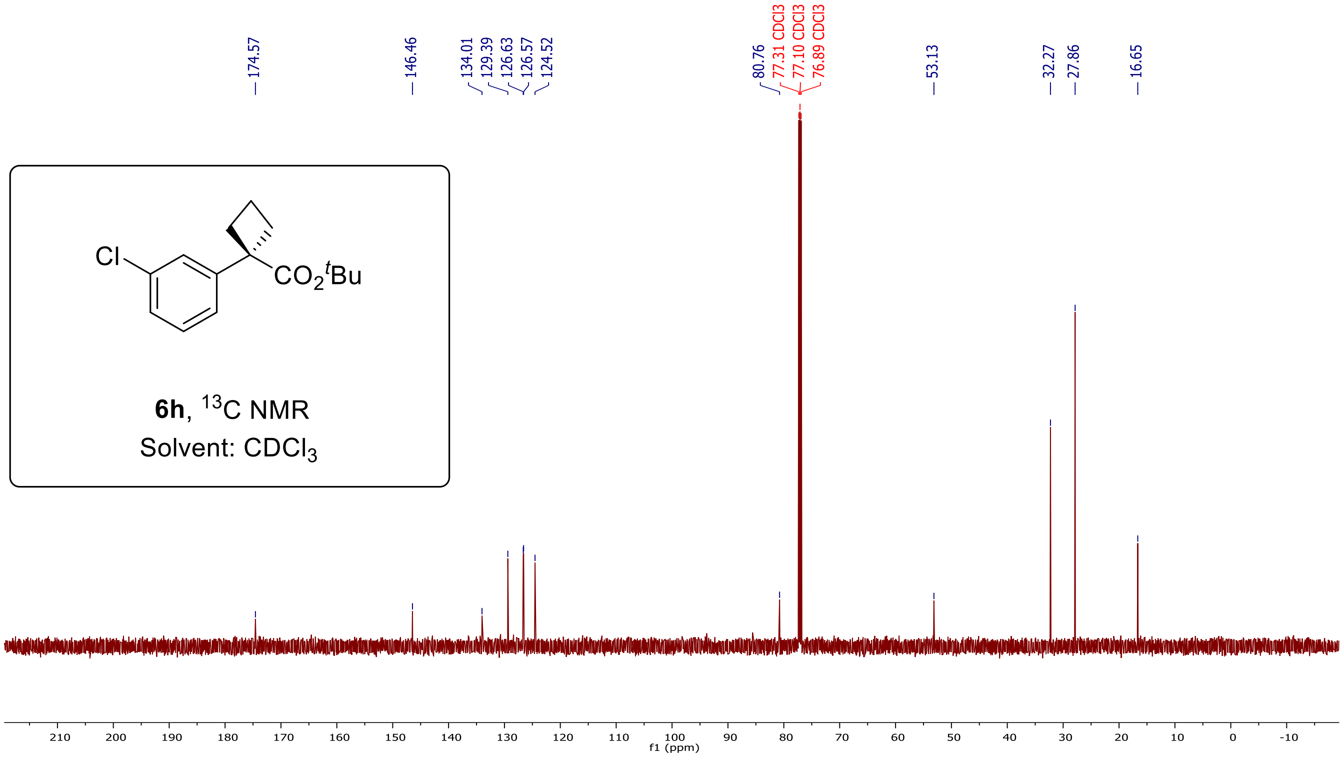

Supplementary Figure 76. NMR spectra of **6h**

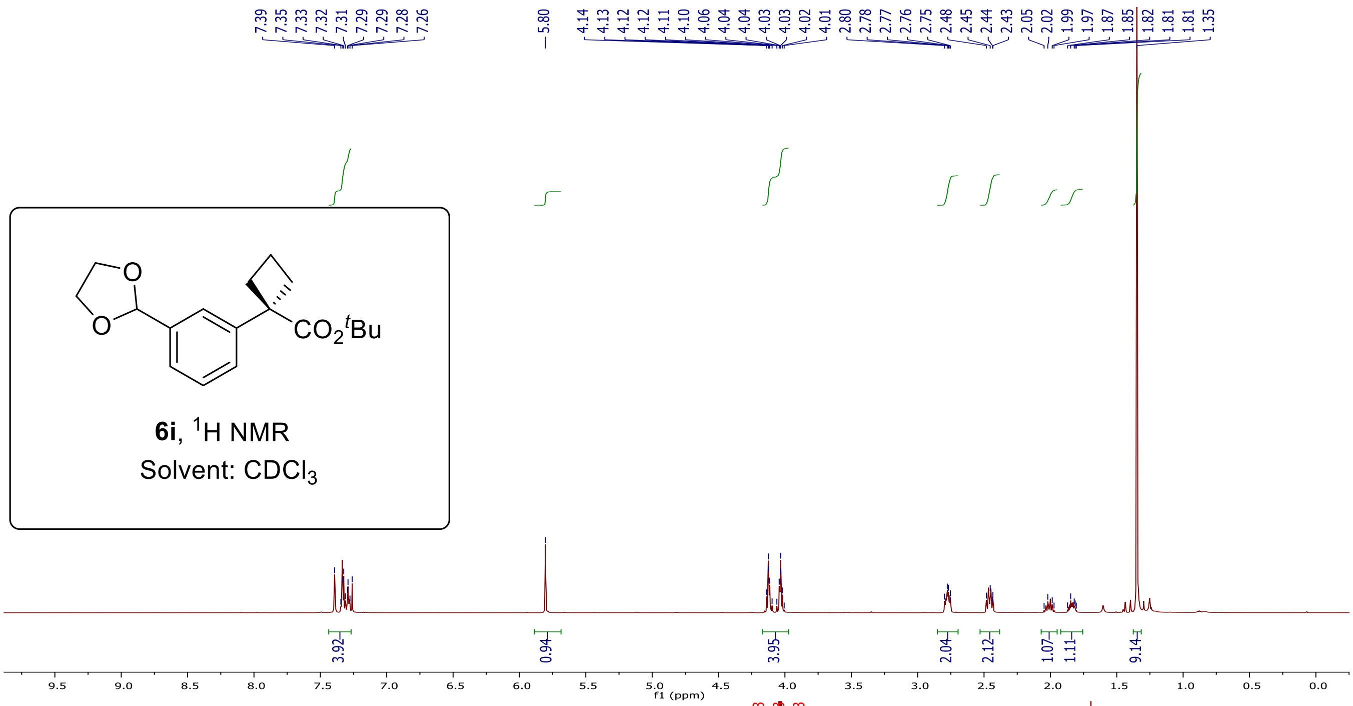

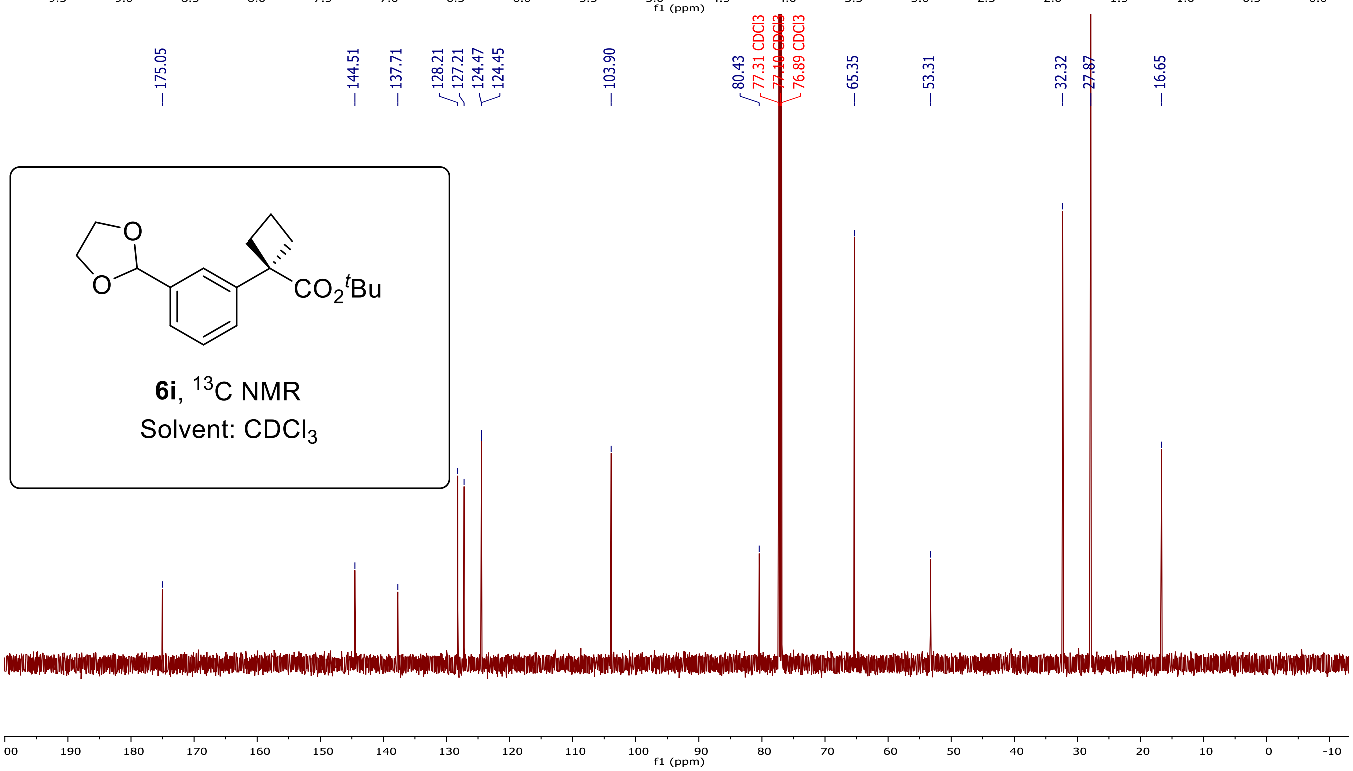

Supplementary Figure 77. NMR spectra of **6i**

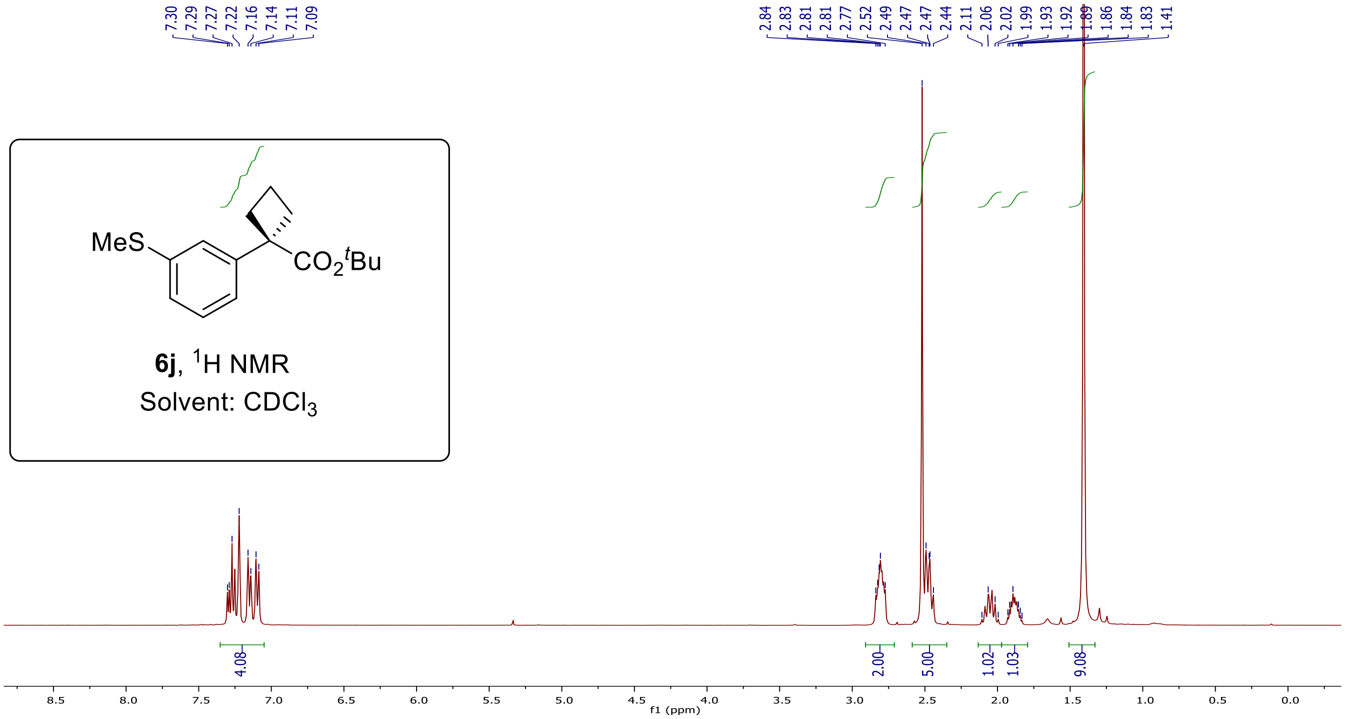

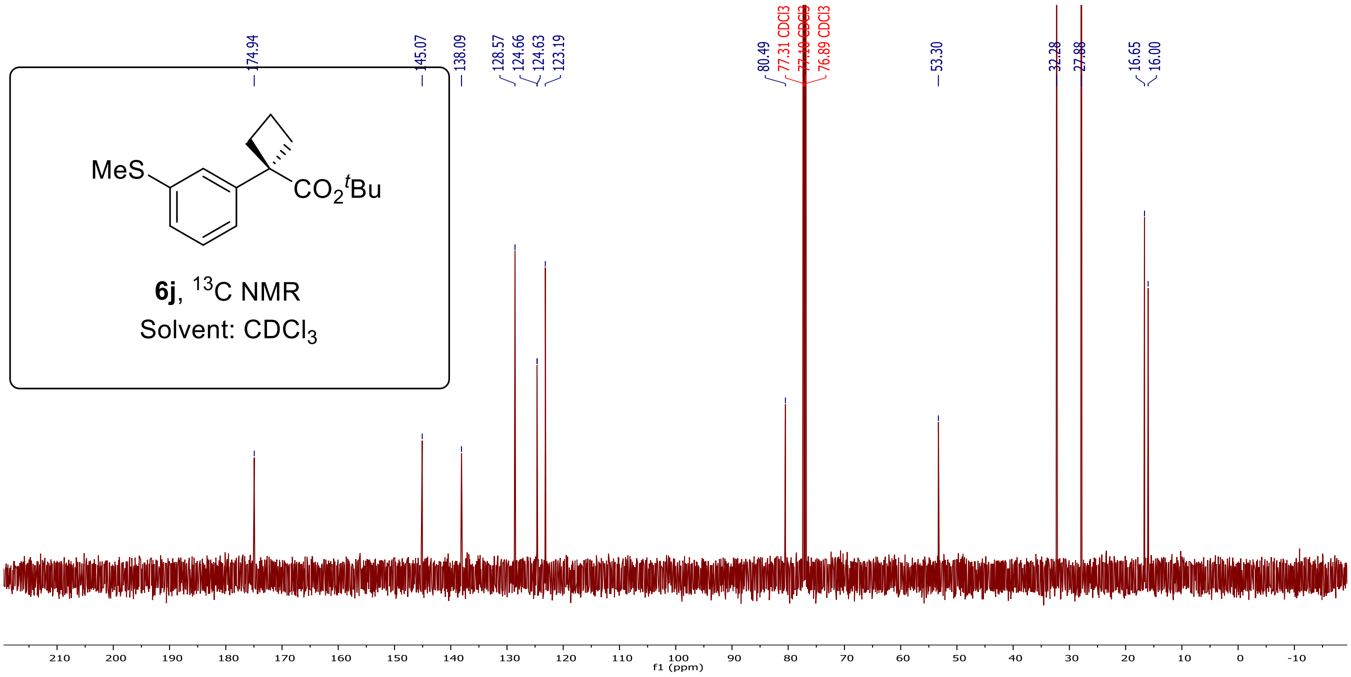

Supplementary Figure 78. NMR spectra of **6j**

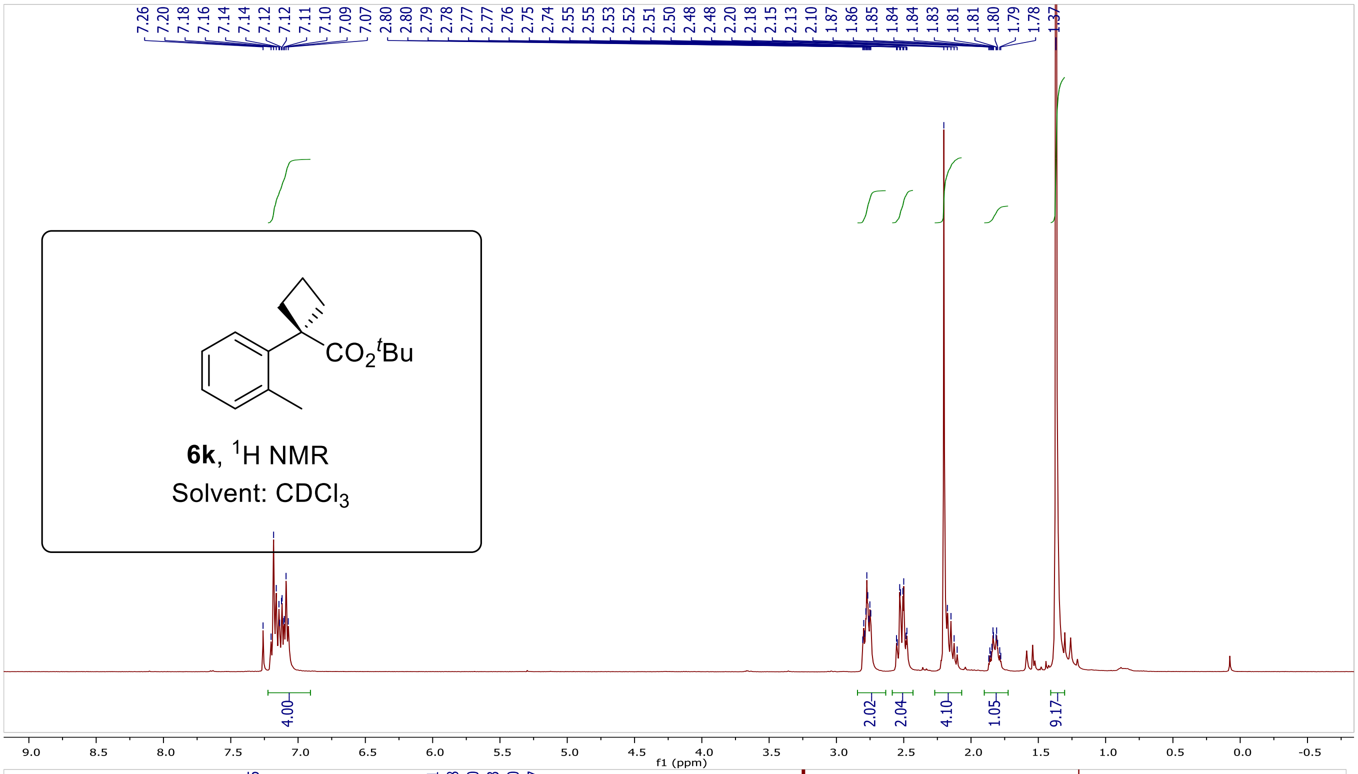

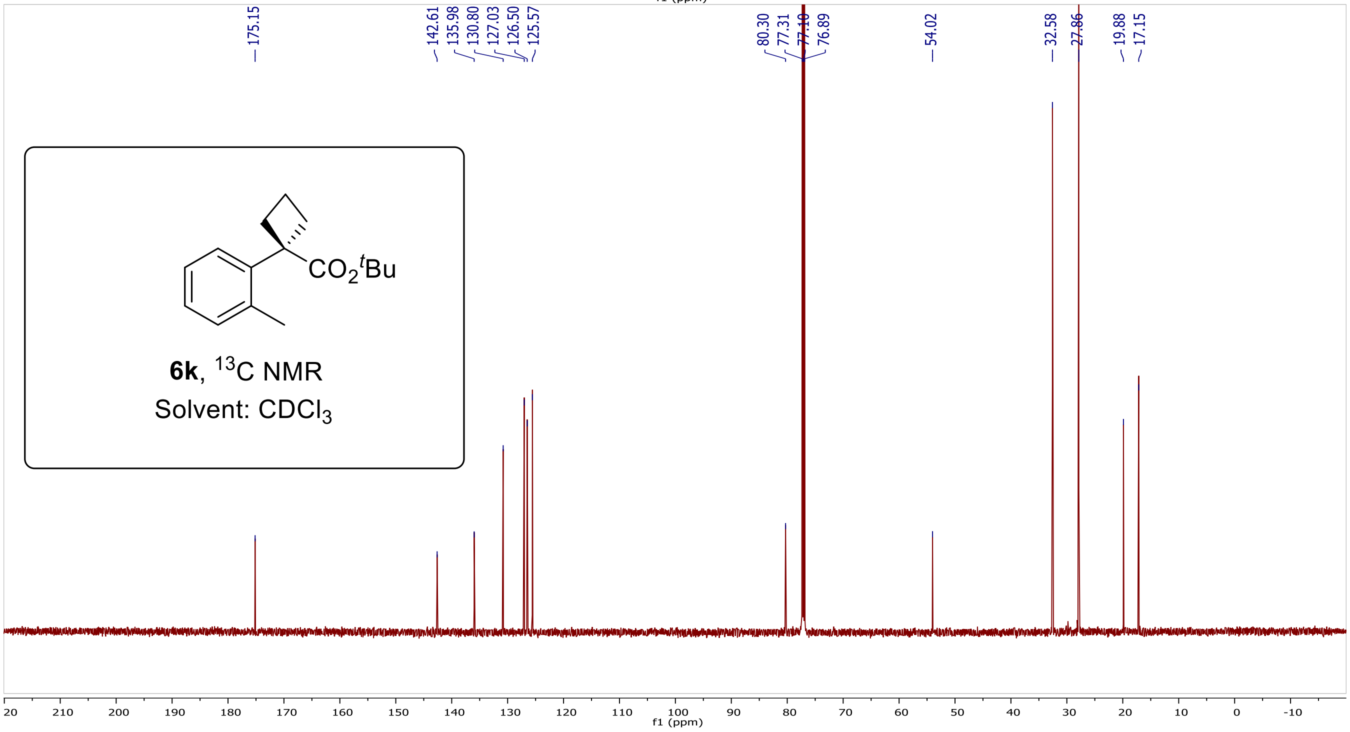

Supplementary Figure 79. NMR spectra of **6k**

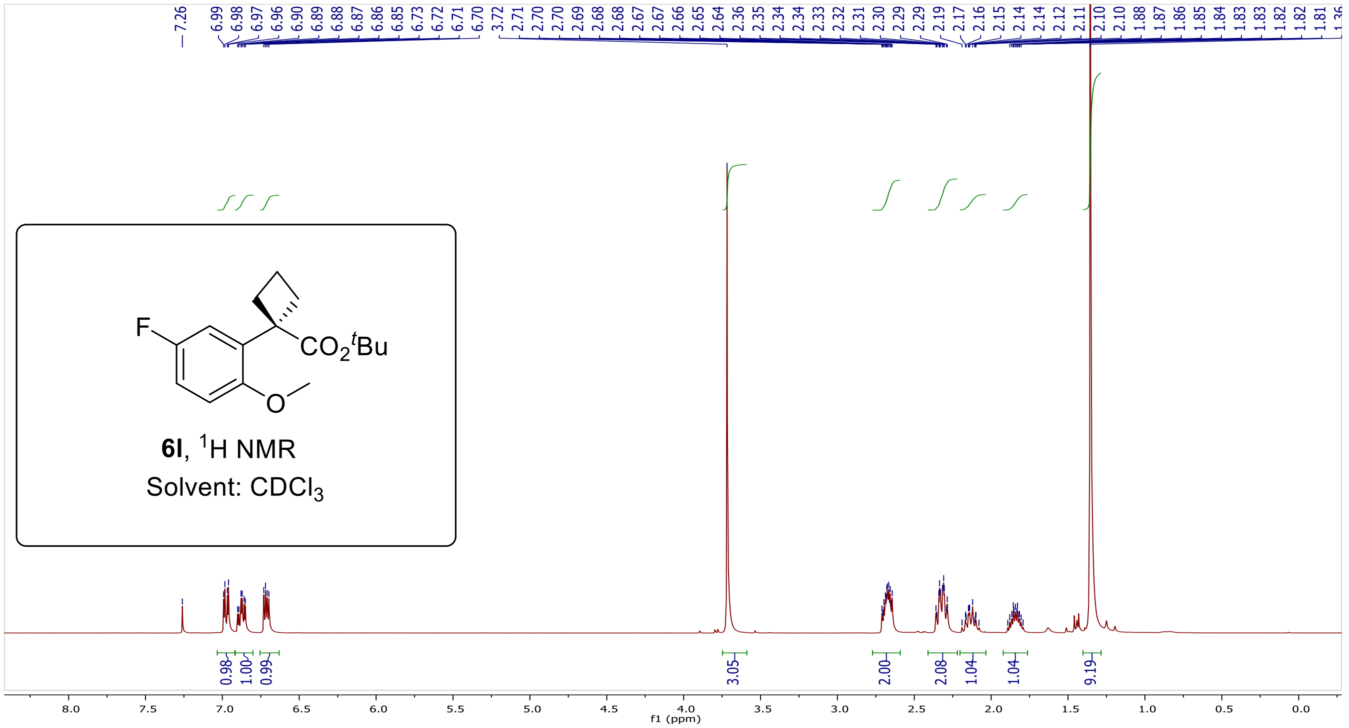

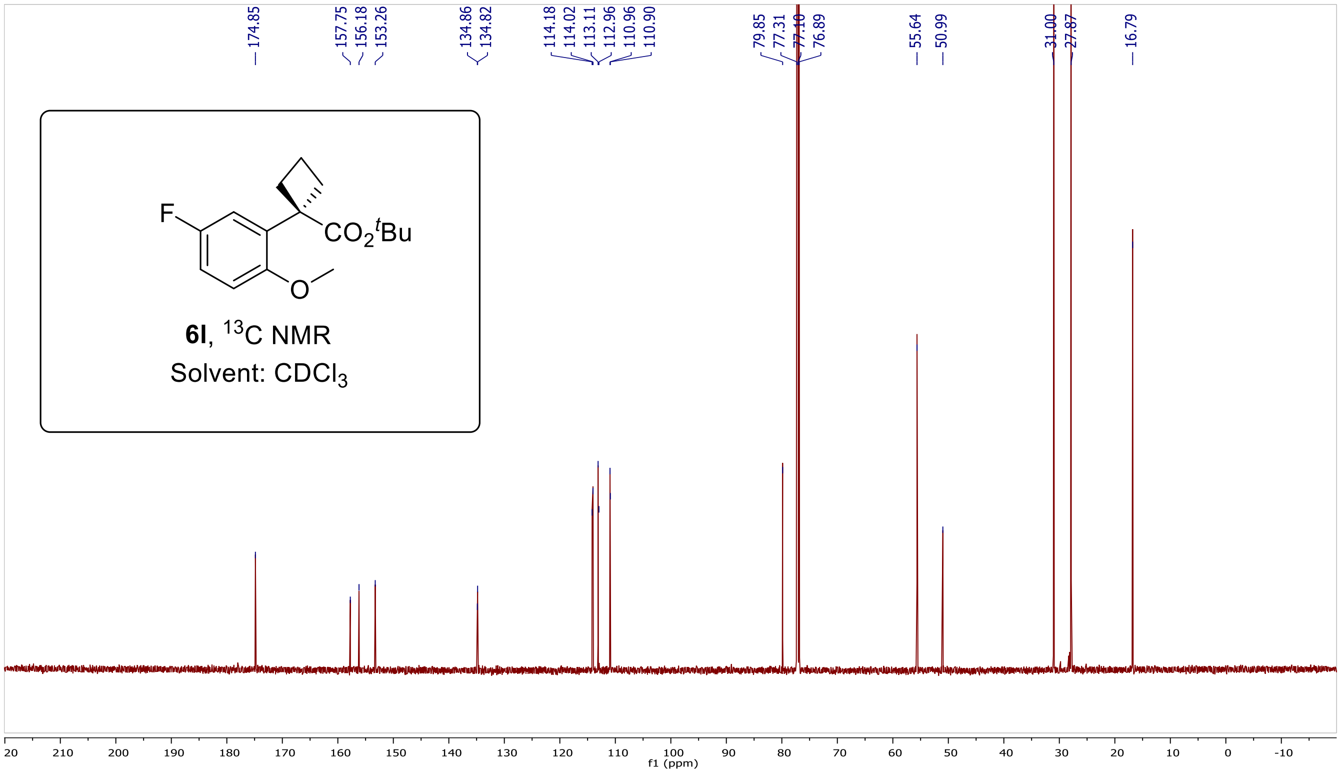

Supplementary Figure 80. NMR spectra of **6l**

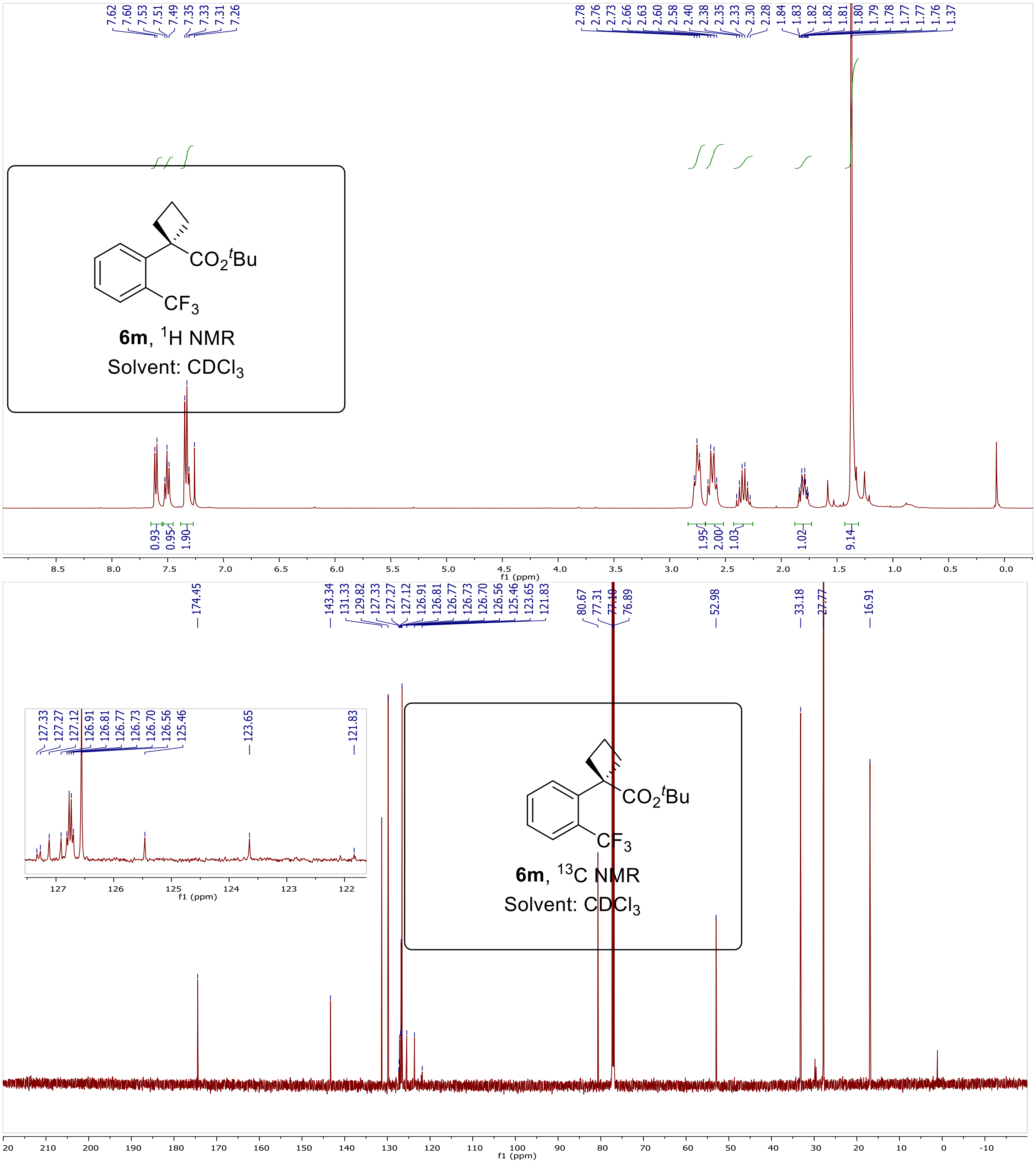

Supplementary Figure 81. NMR spectra of **6m**

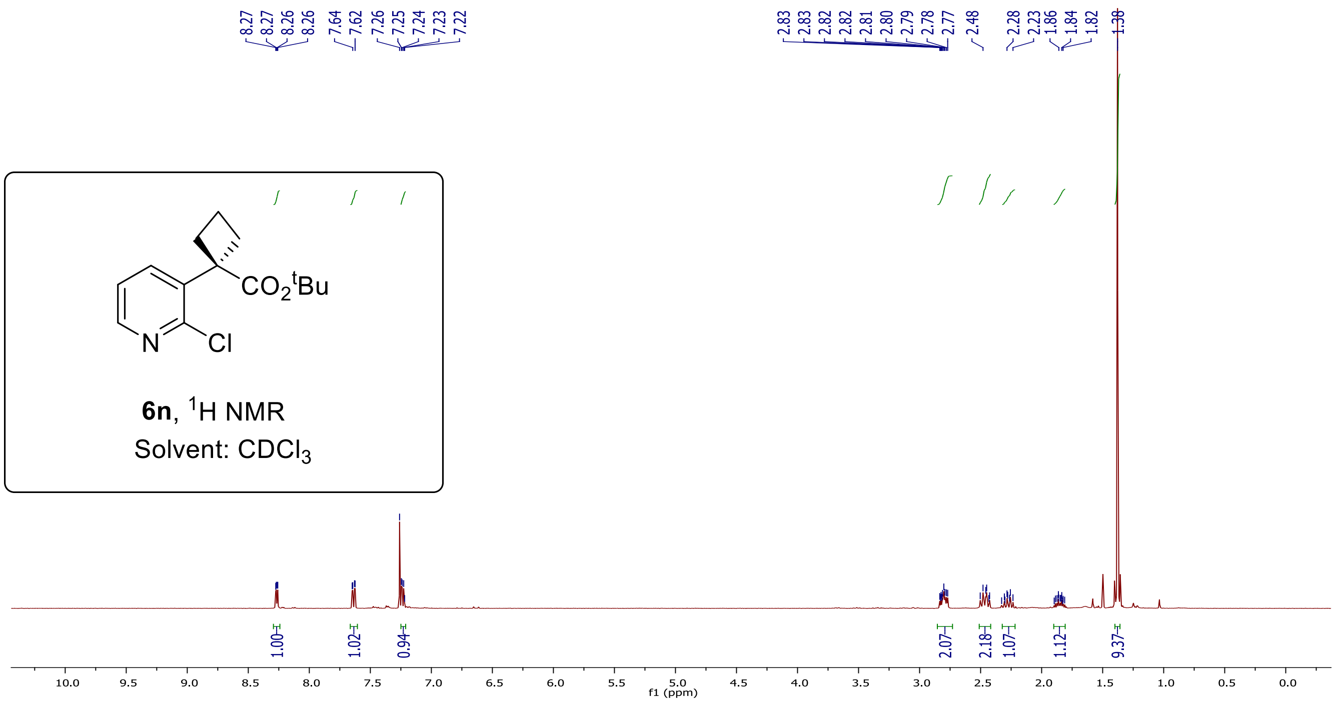

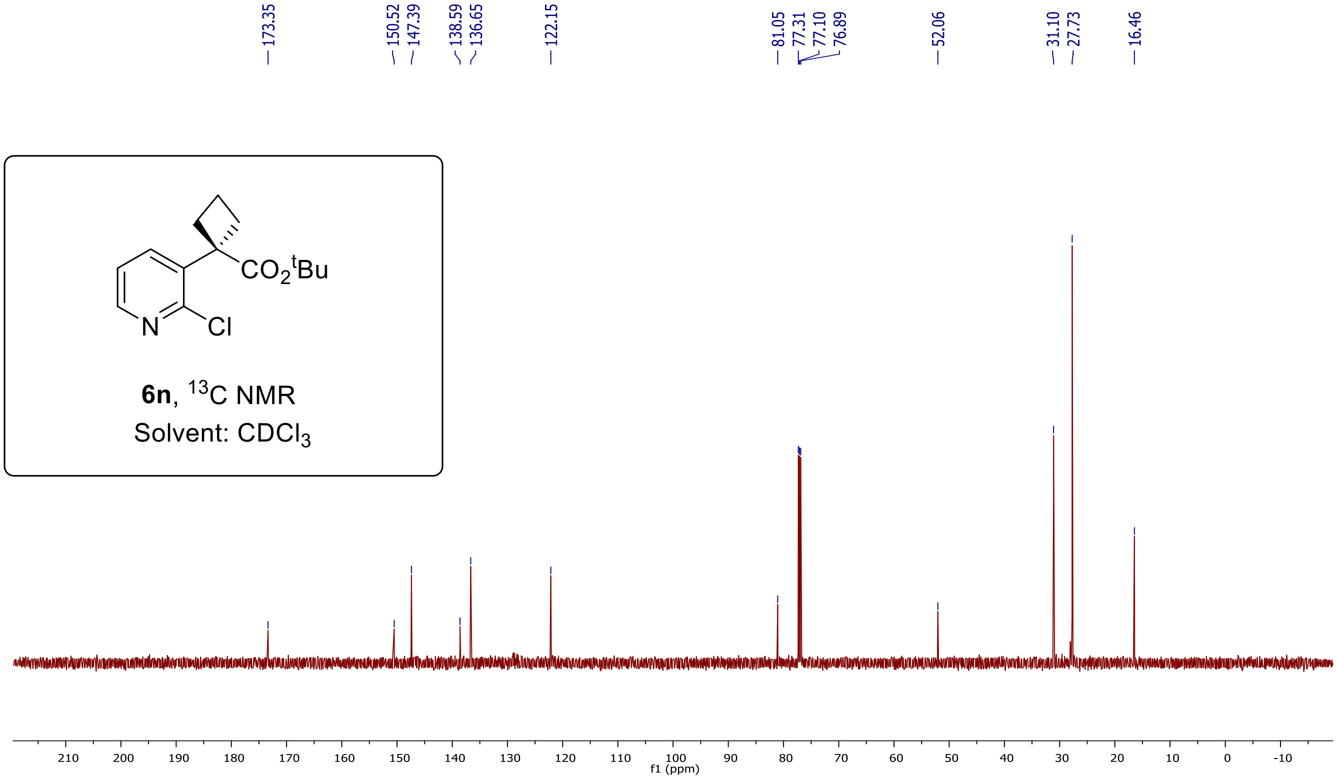

Supplementary Figure 82. NMR spectra of **6n**

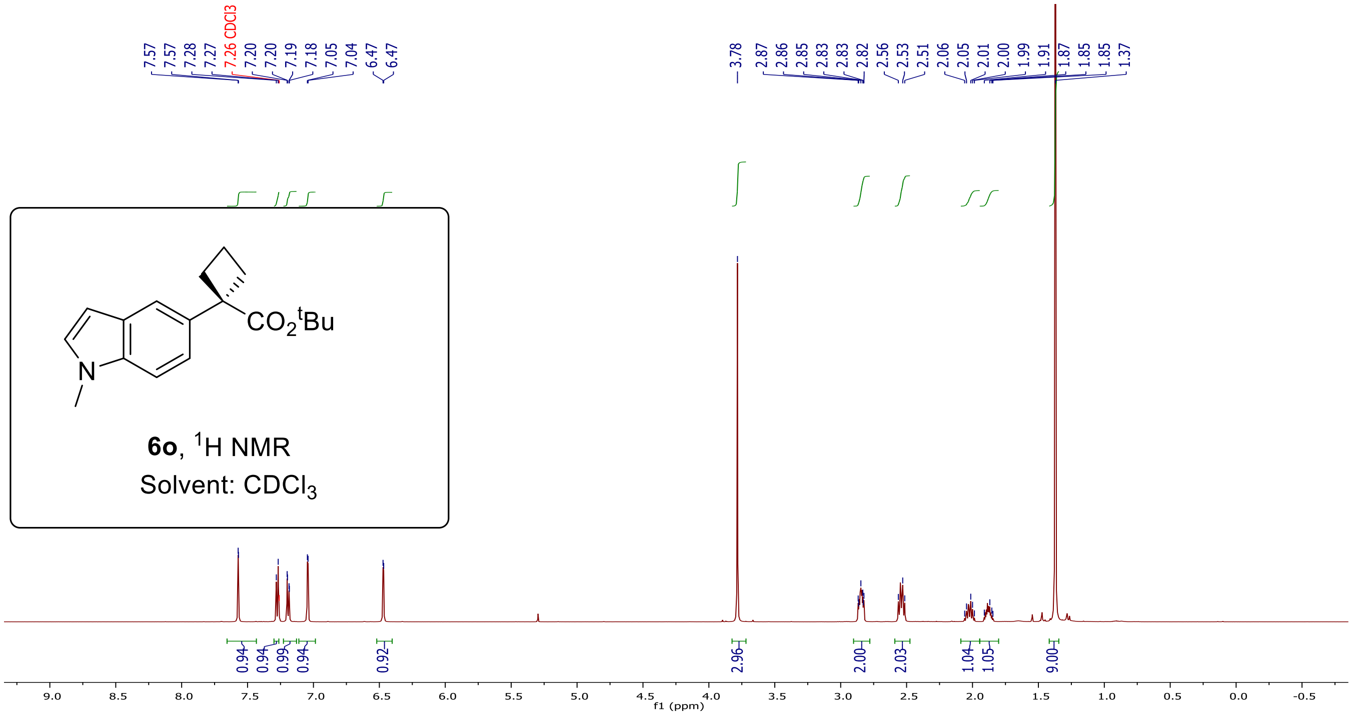

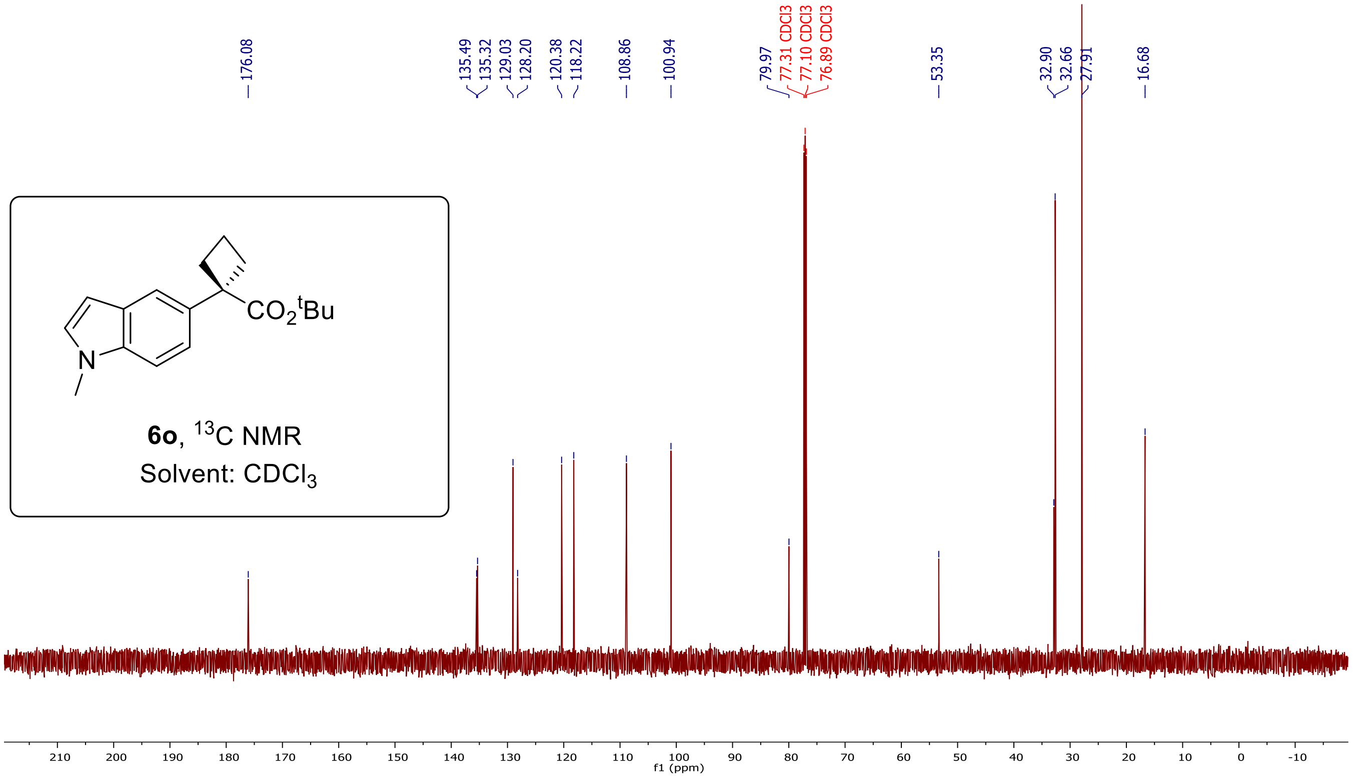

Supplementary Figure 83. NMR spectra of **6o**

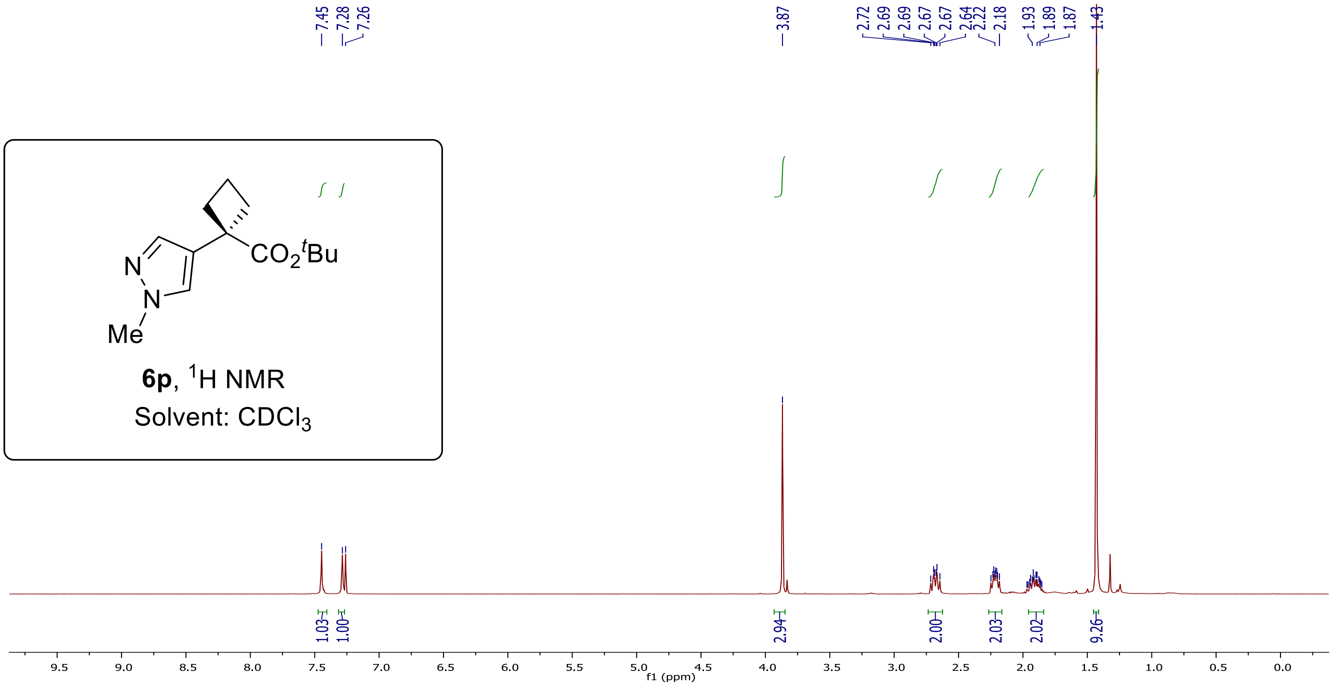

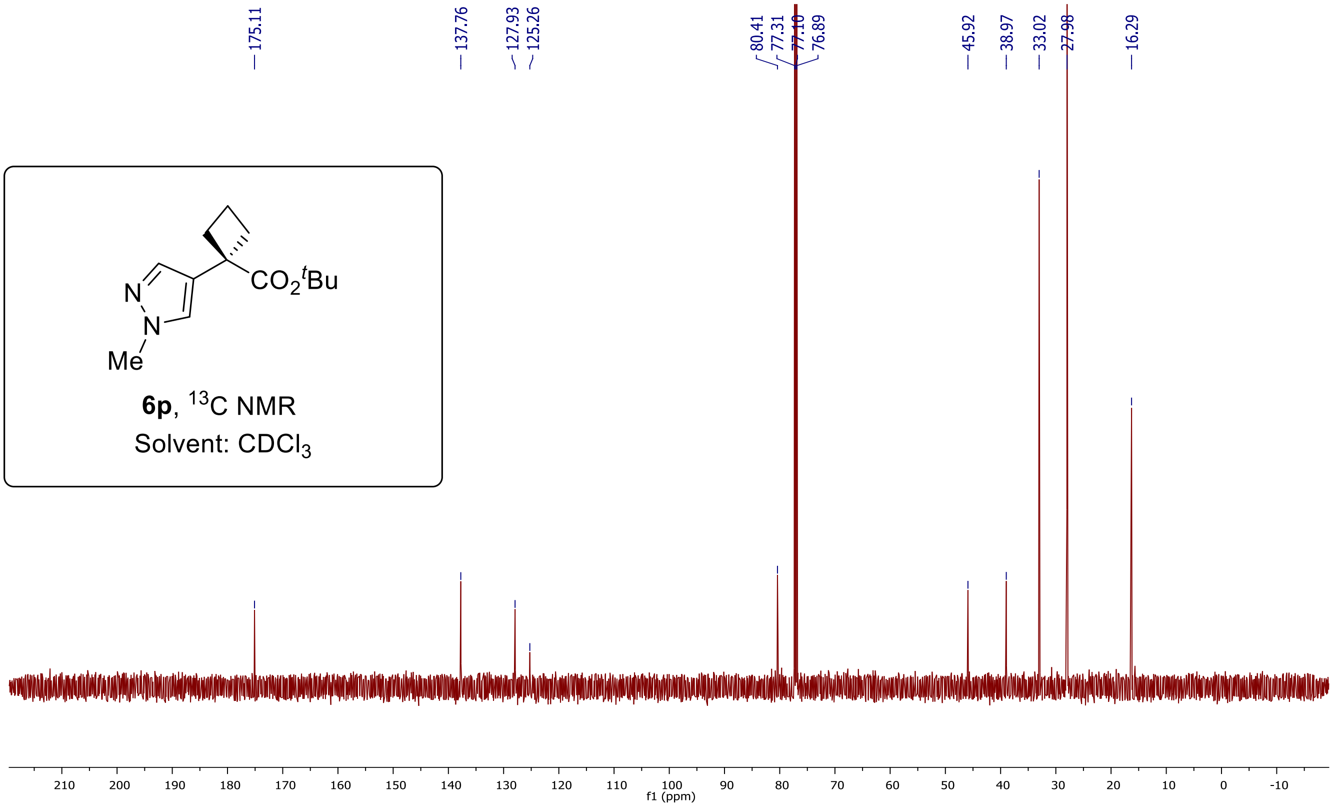

Supplementary Figure 84. NMR spectra of **6p**

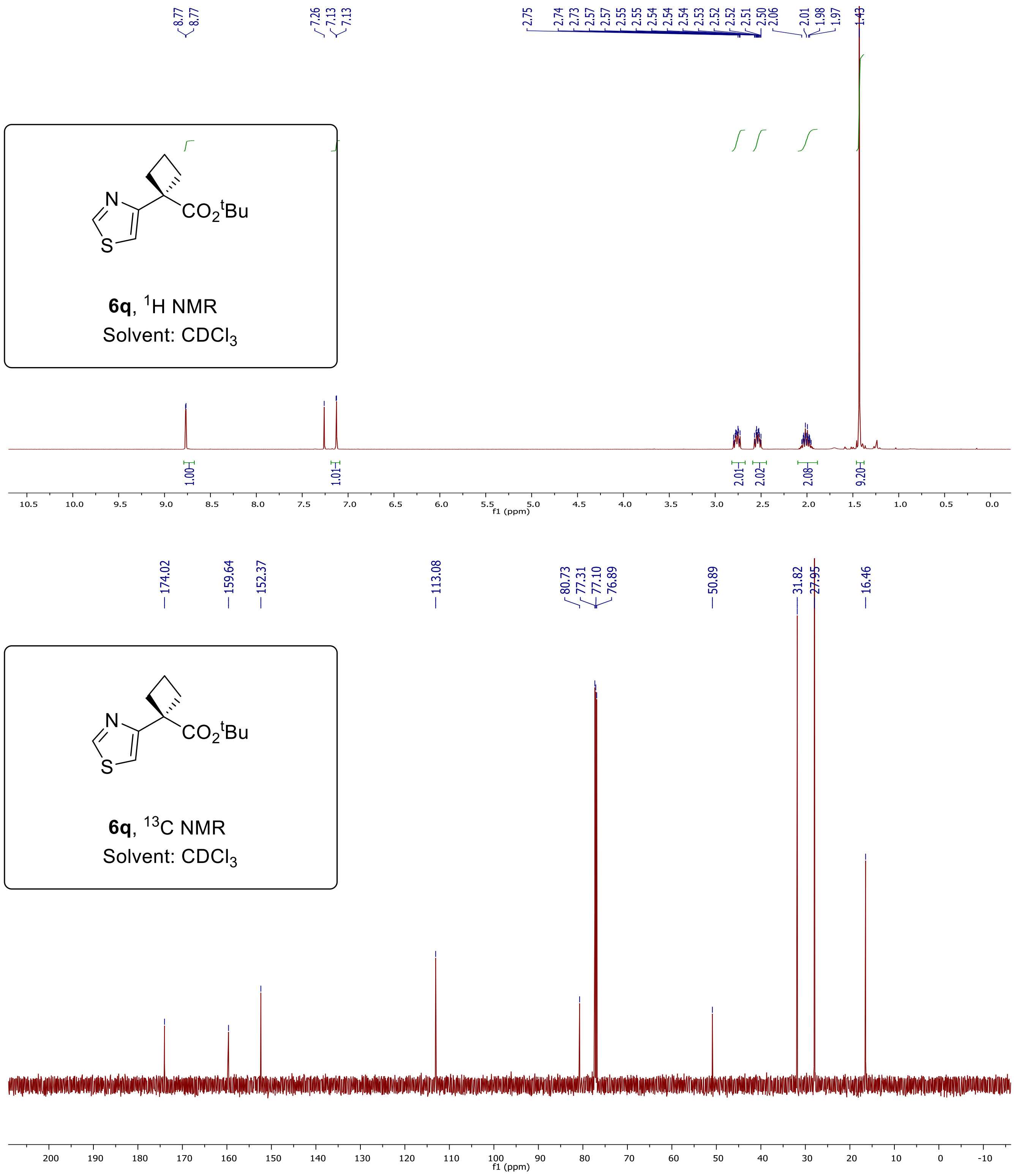

Supplementary Figure 85. NMR spectra of **6q**

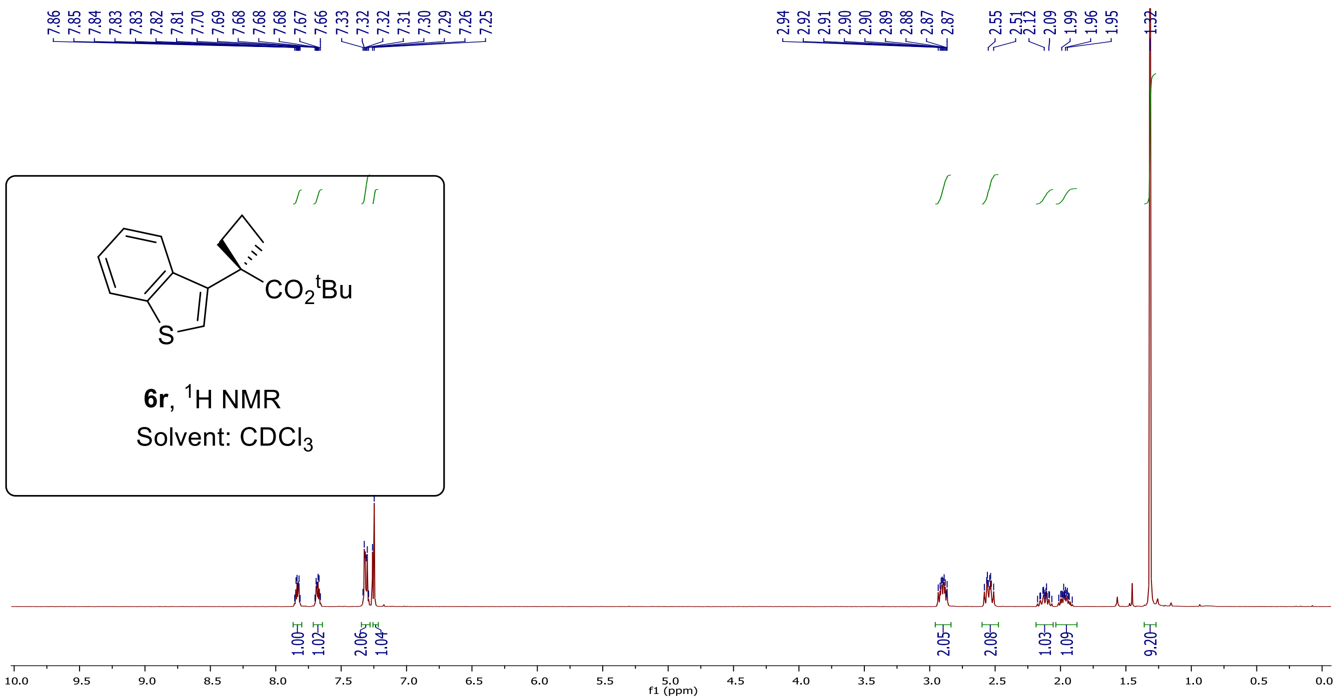

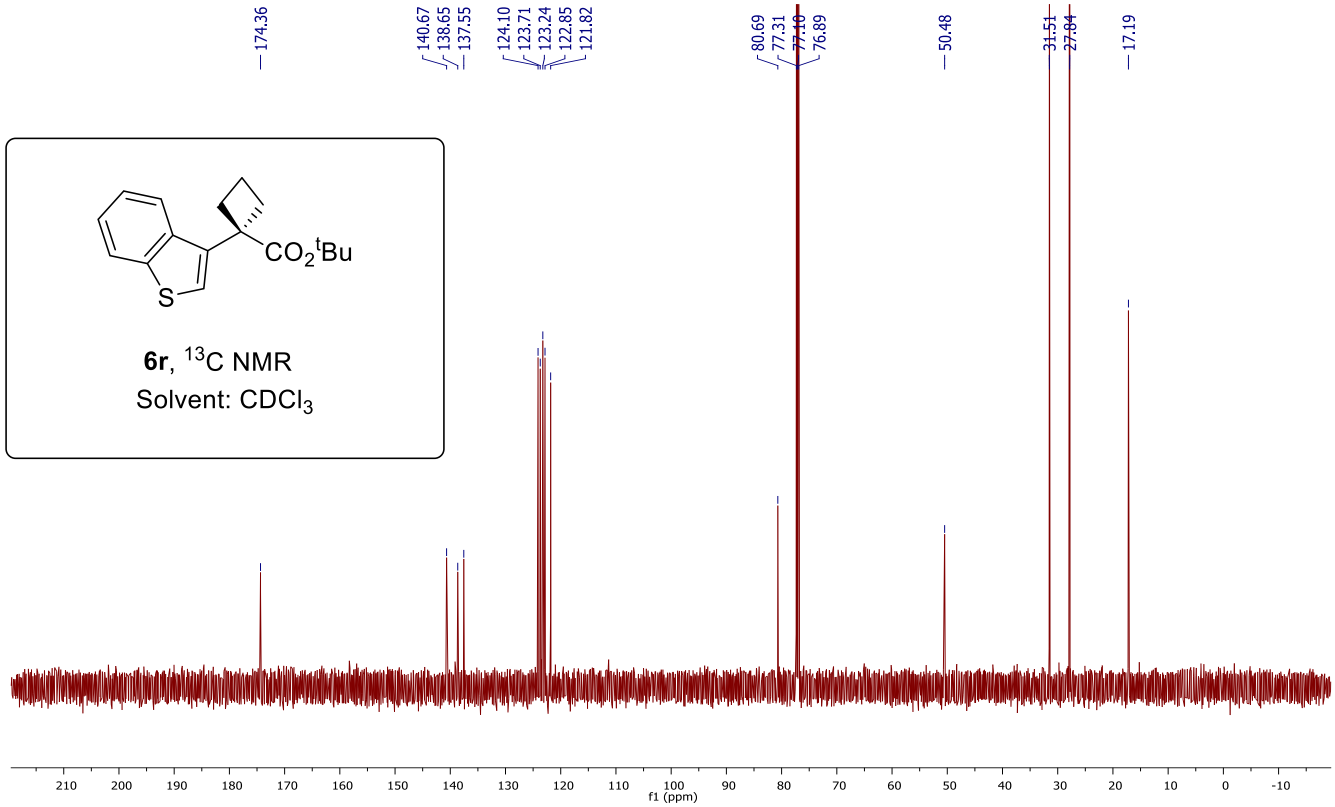

Supplementary Figure 86. NMR spectra of **6r**

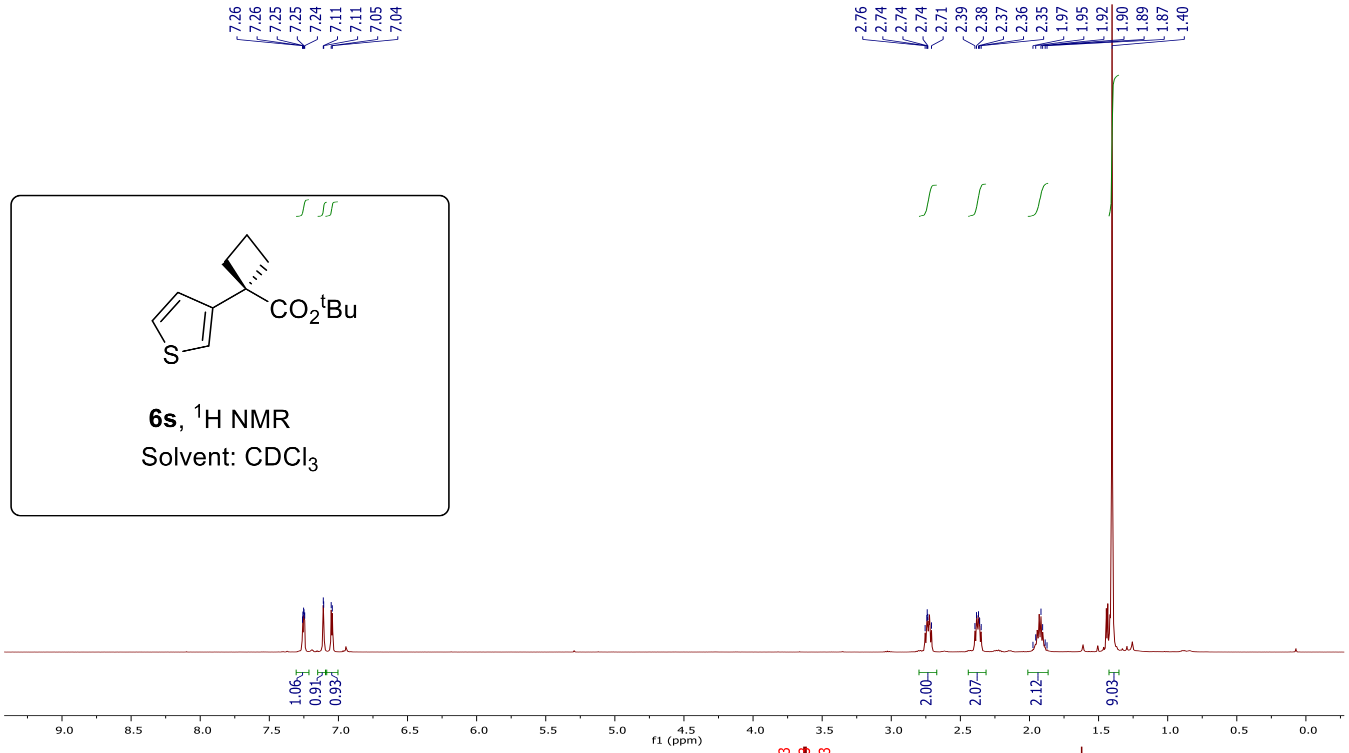

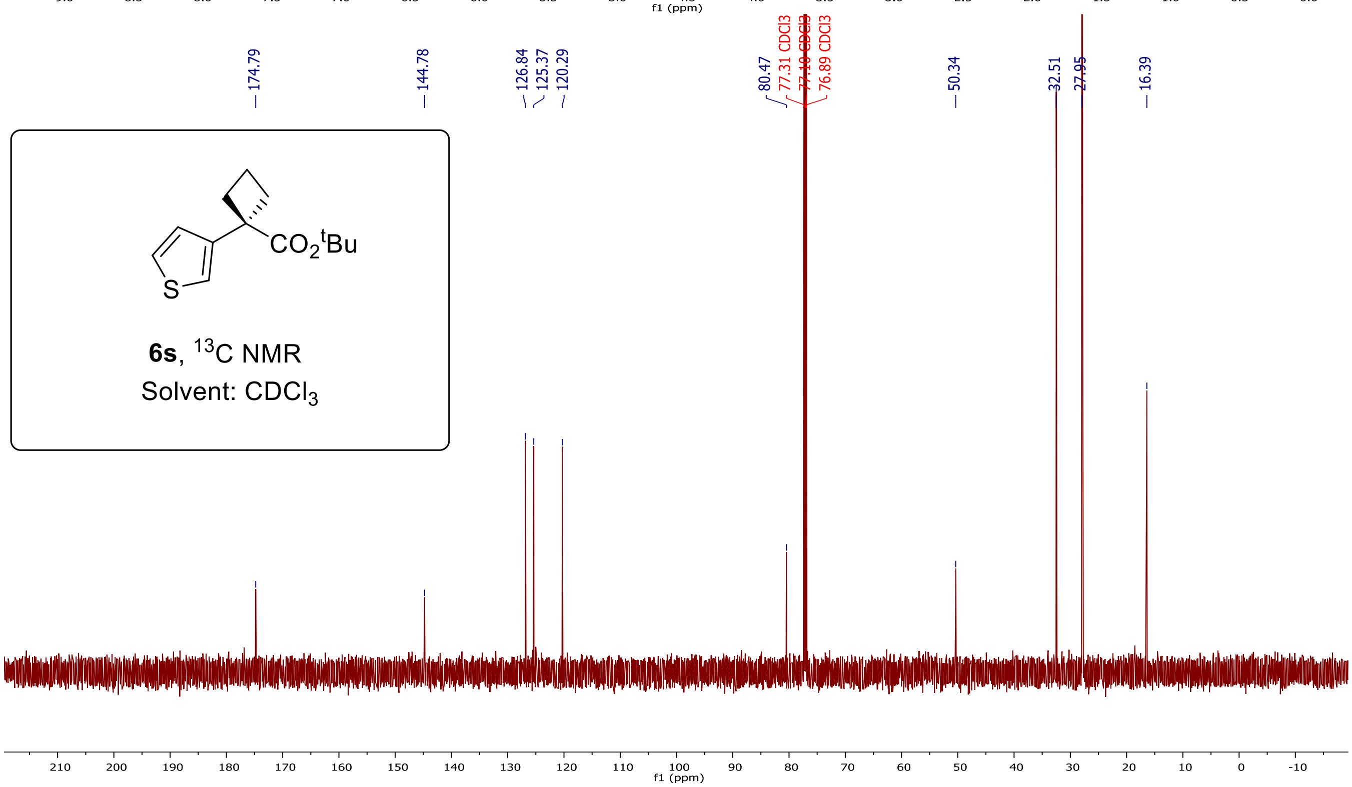

Supplementary Figure 87. NMR spectra of **6s**

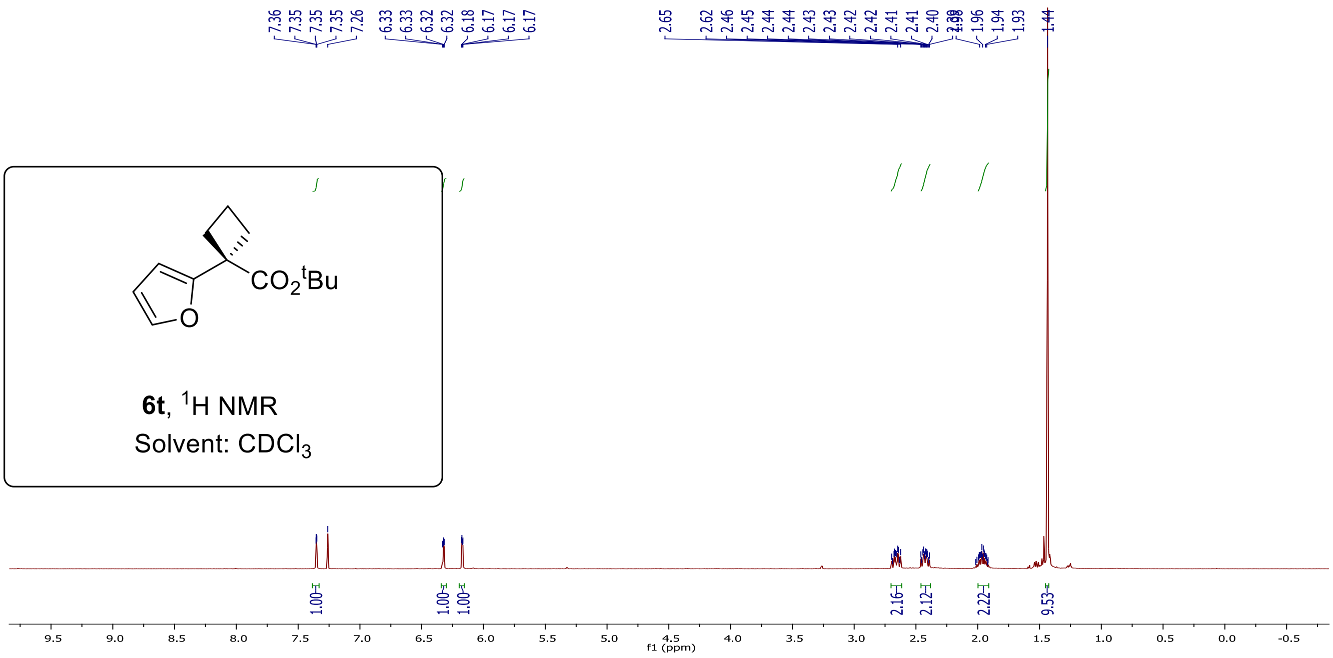

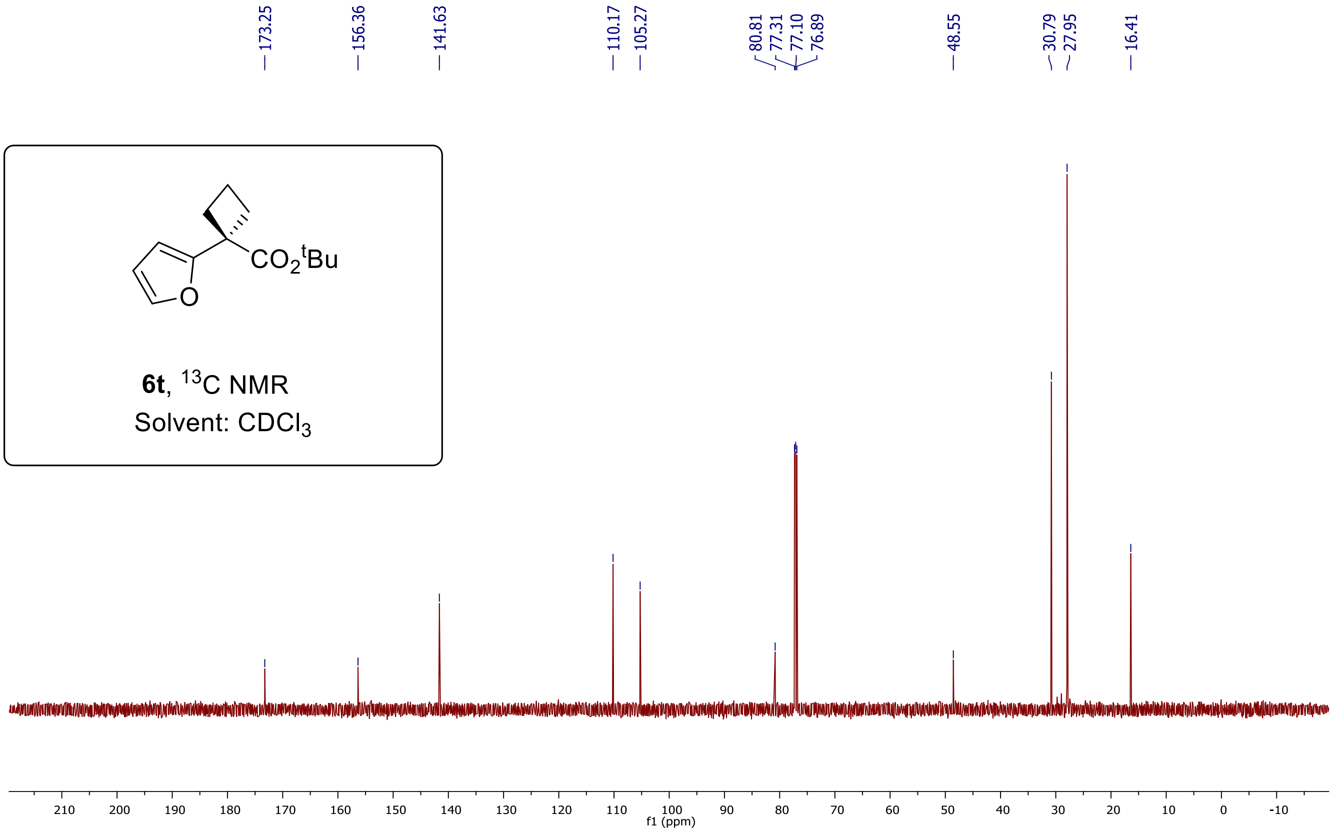

Supplementary Figure 88. NMR spectra of **6t**

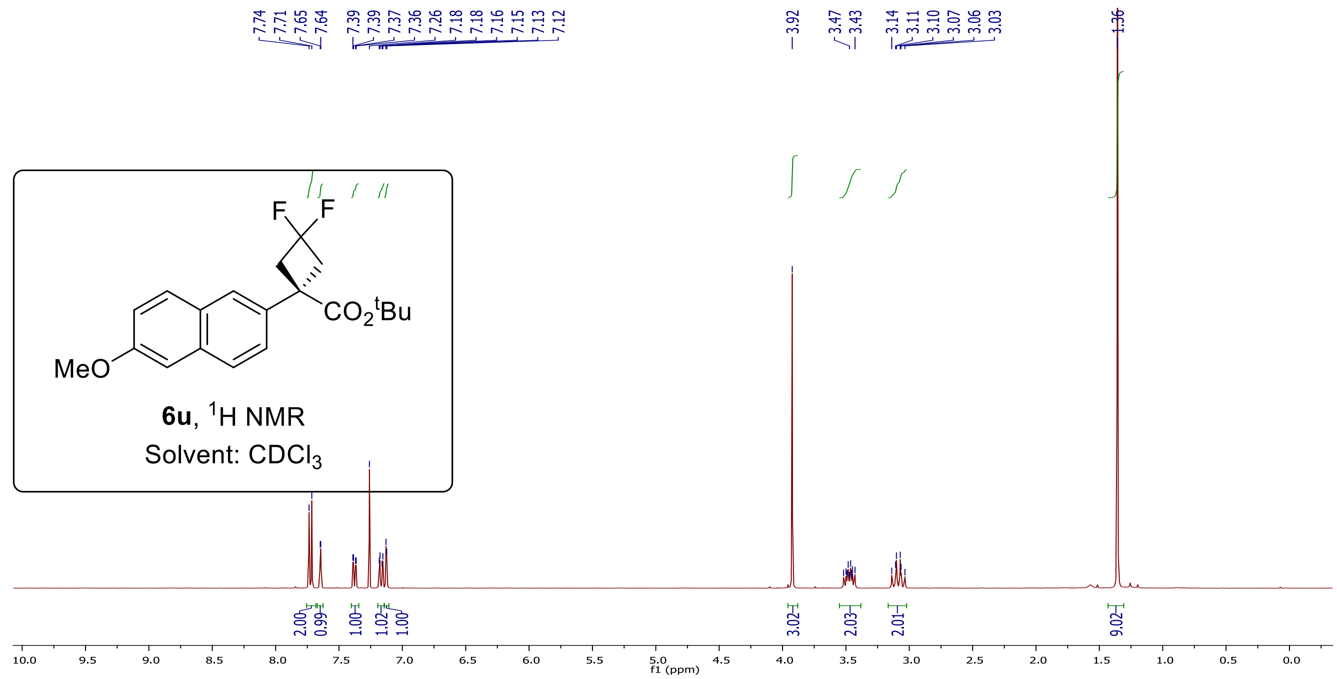

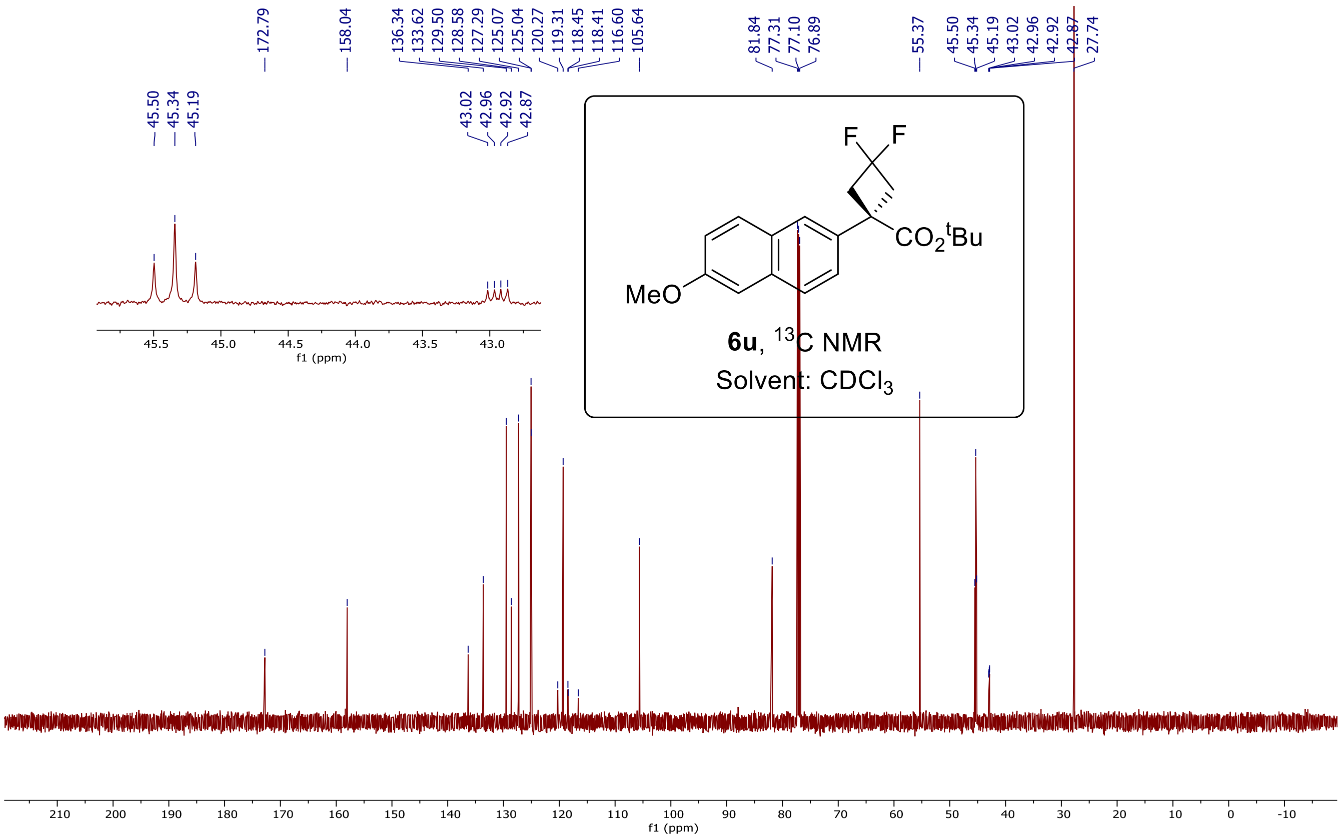

Supplementary Figure 89. NMR spectra of **6u**

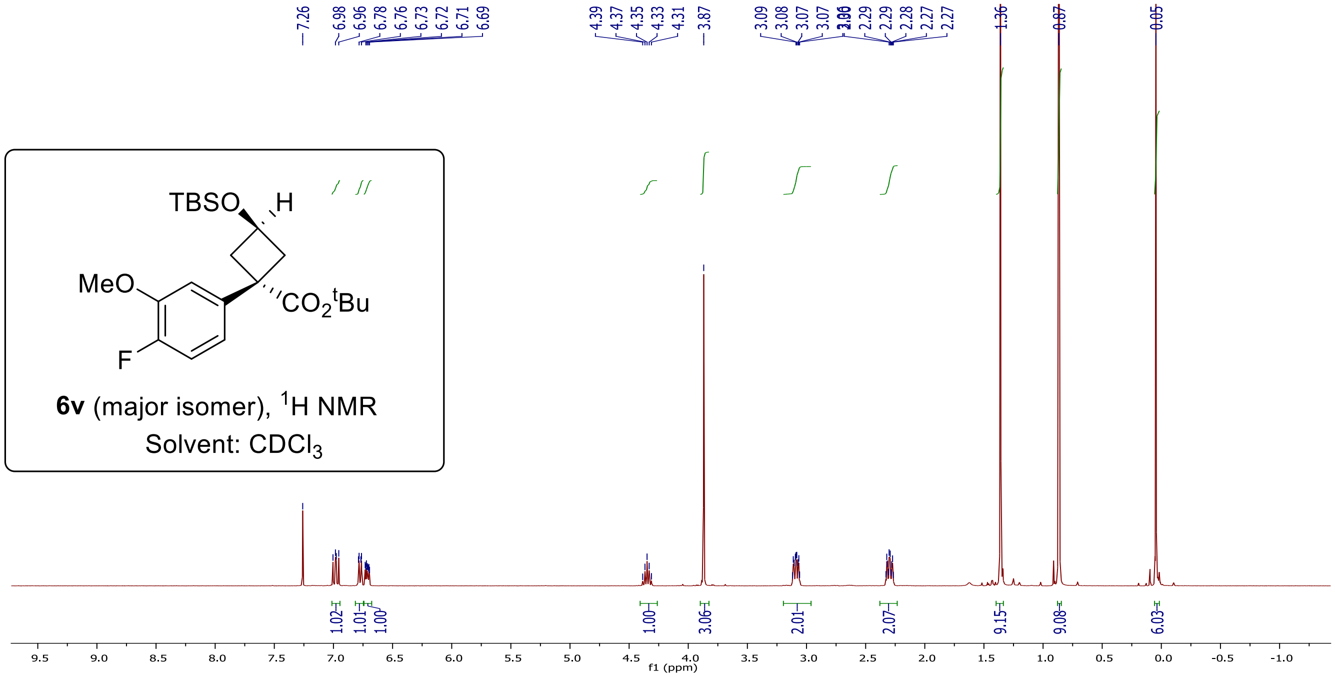

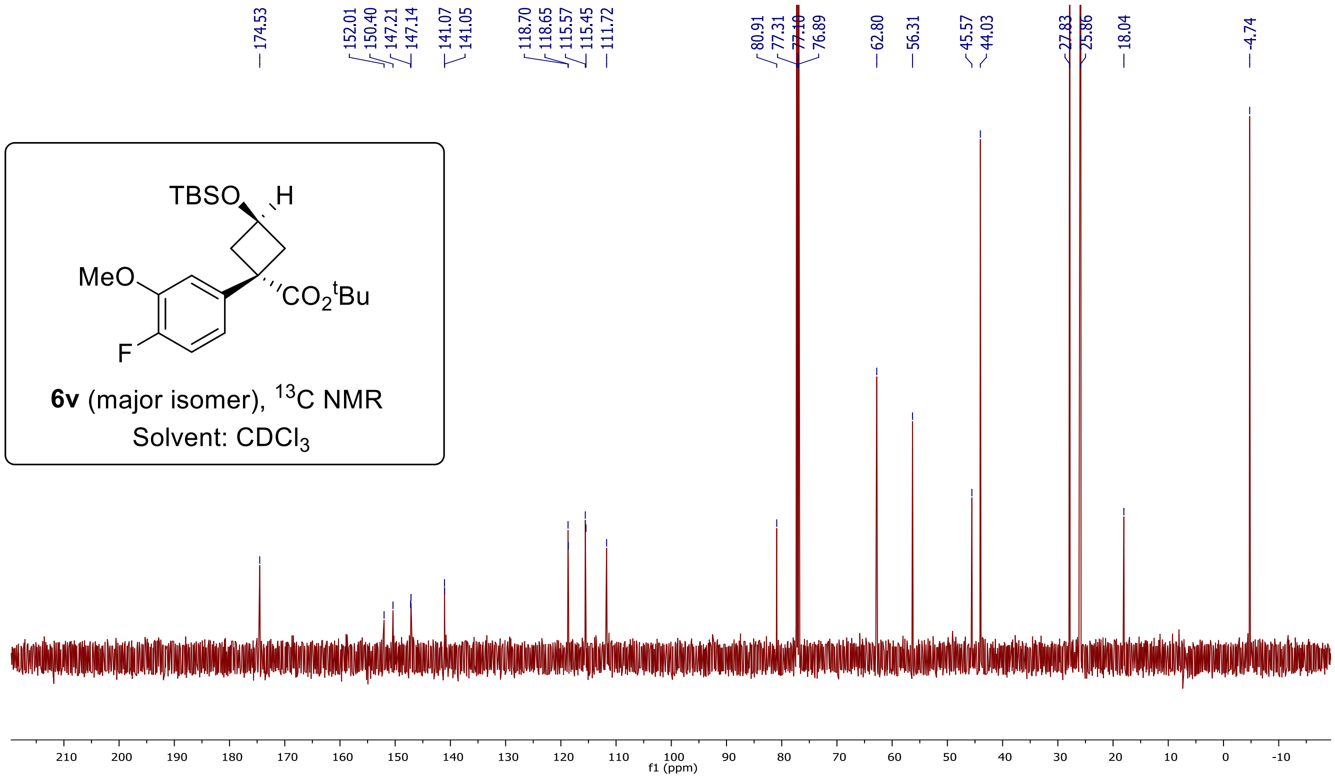

Supplementary Figure 90. NMR spectra of **6v** (major isomer)

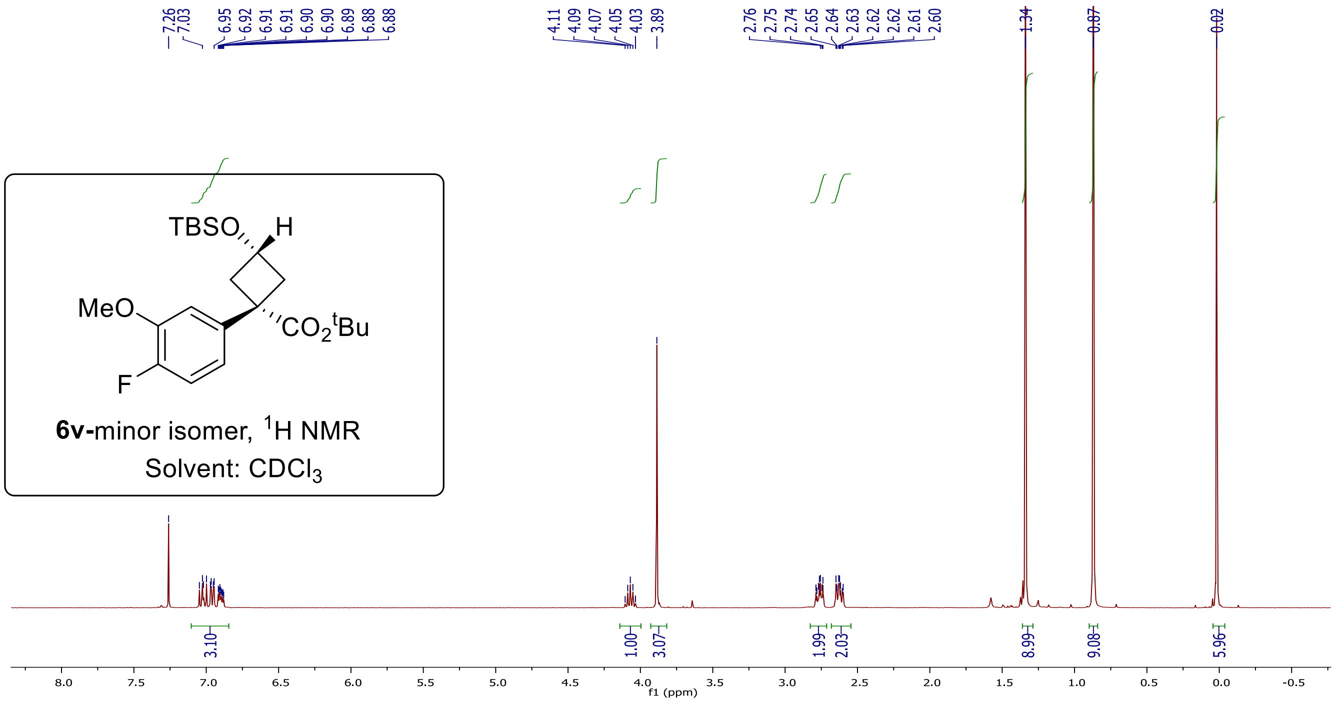

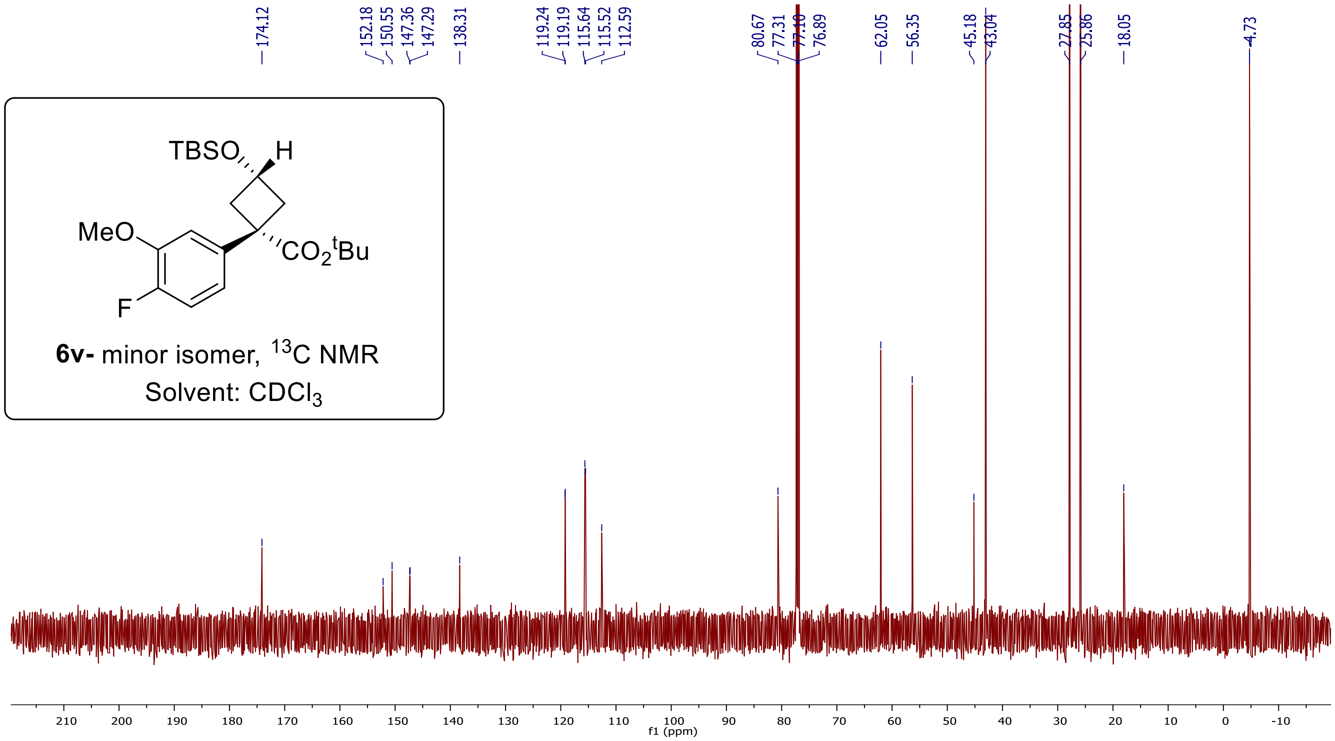

Supplementary Figure 91. NMR spectra of **6v** (minor isomer)

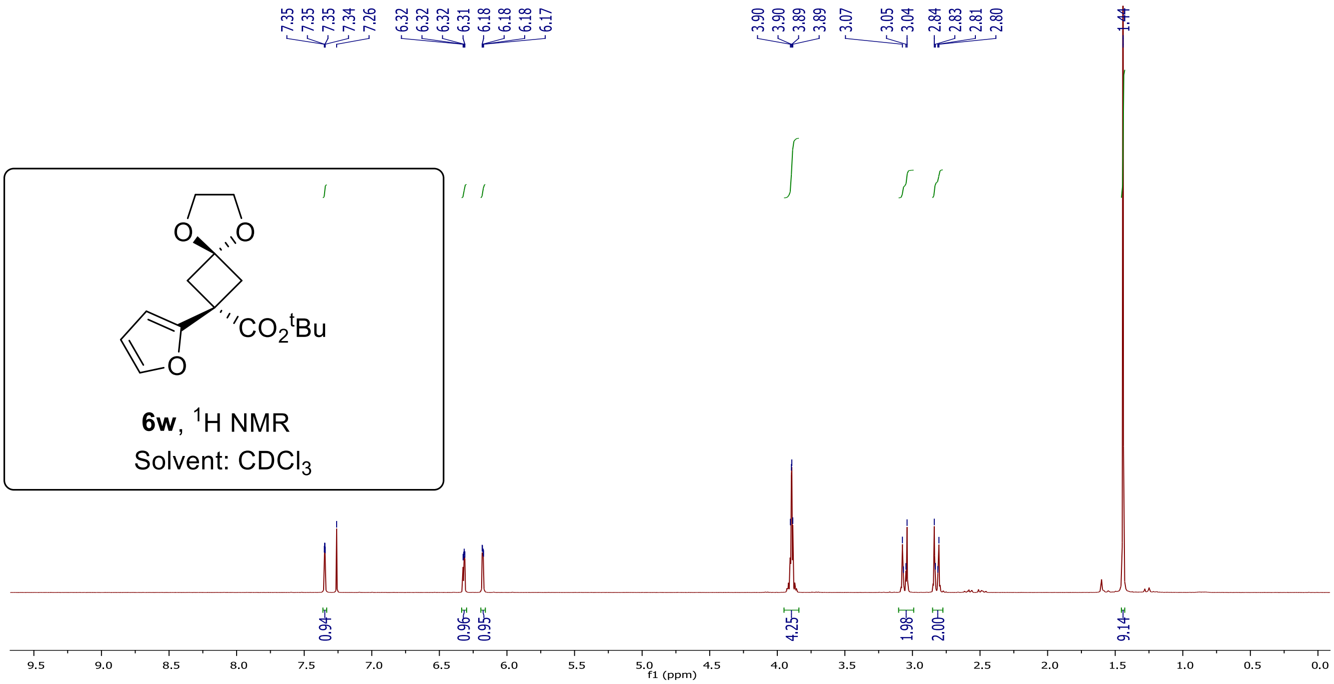

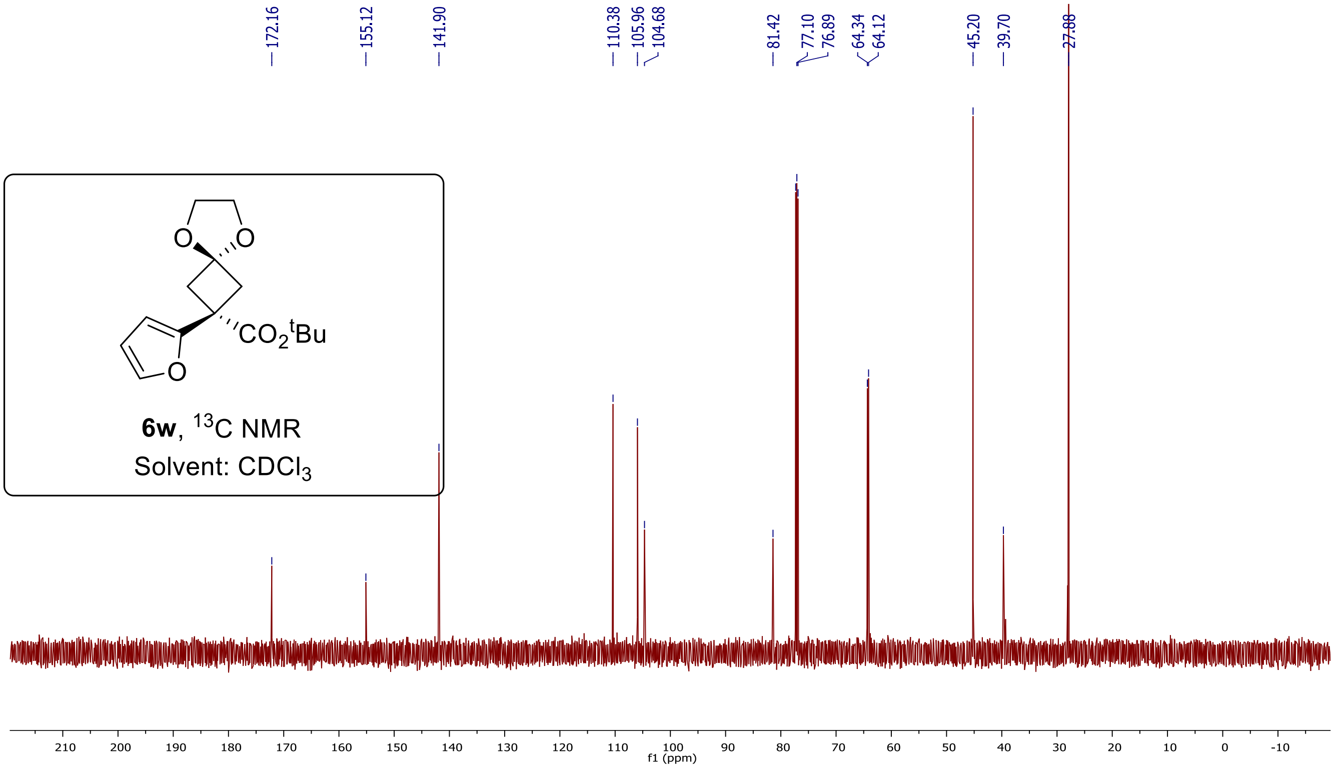

Supplementary Figure 92. NMR spectra of **6w**

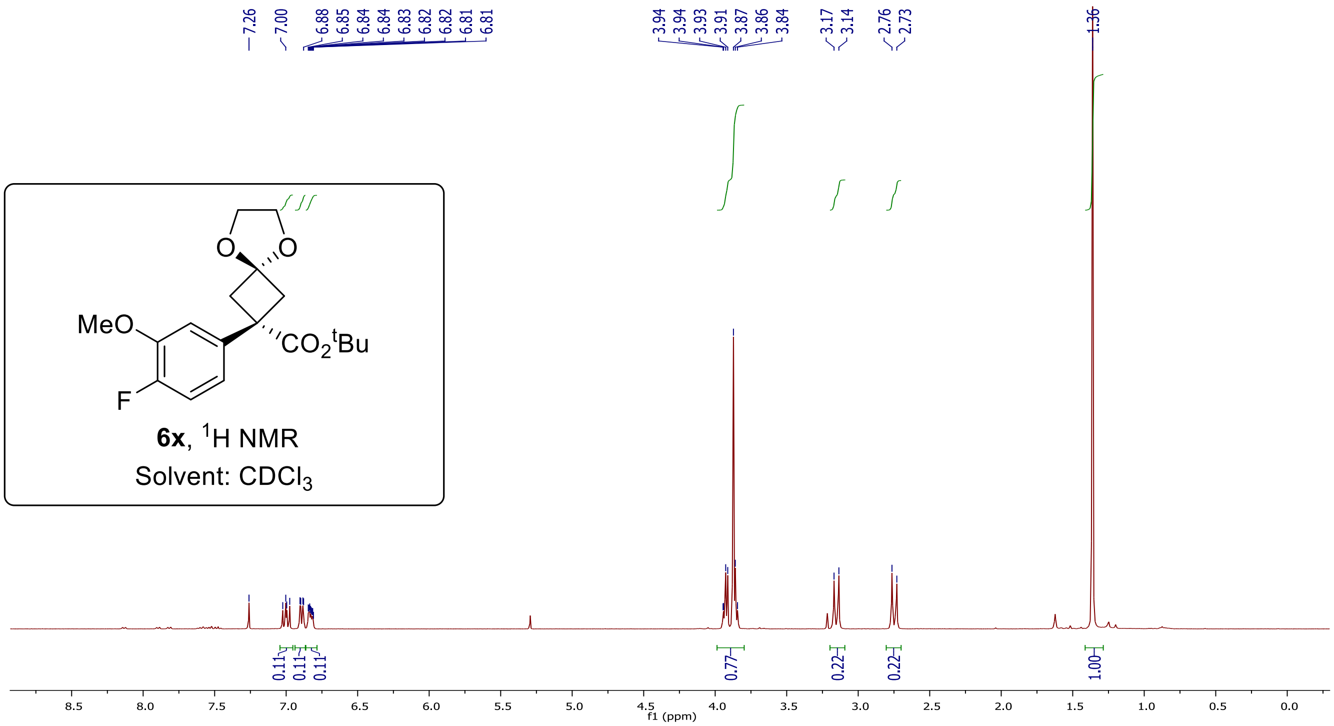

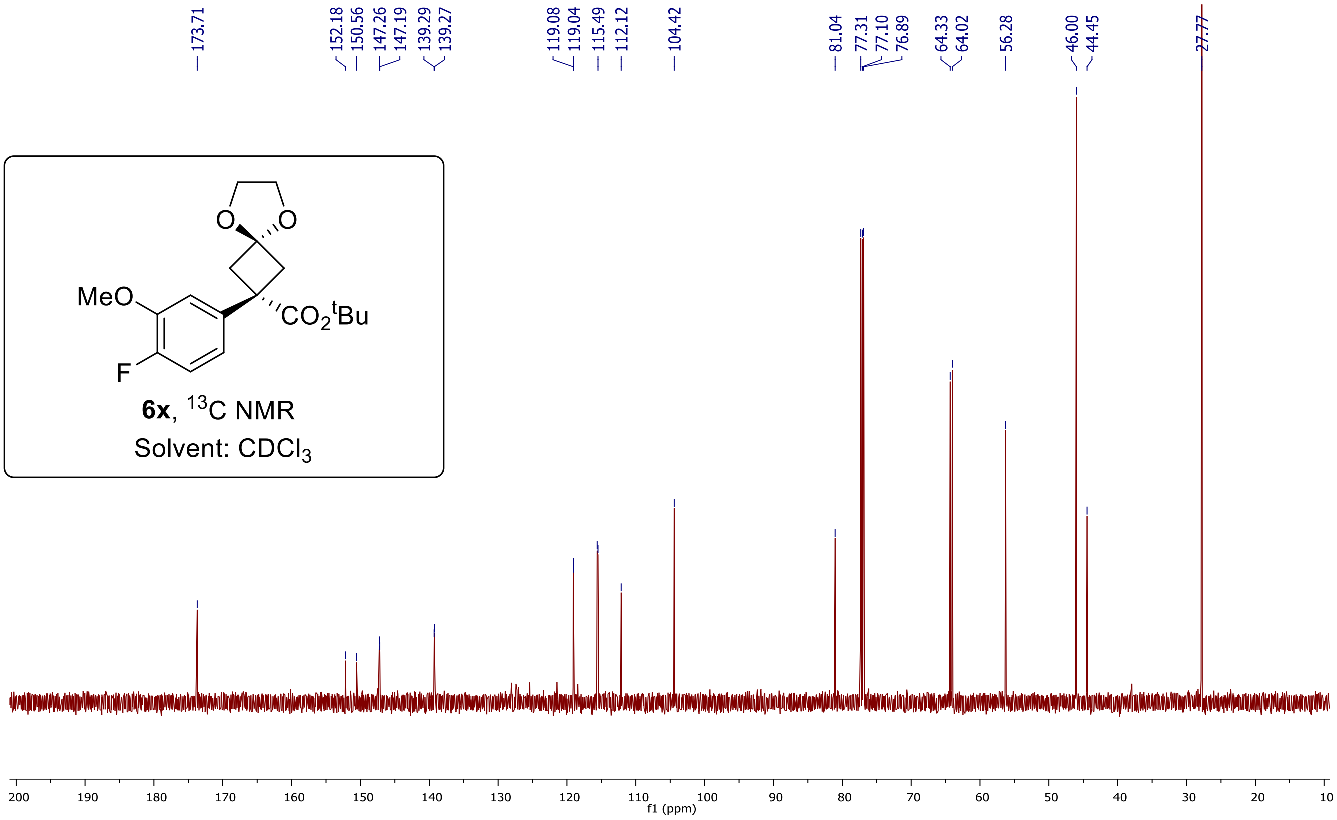

Supplementary Figure 93. NMR spectra of **6x**

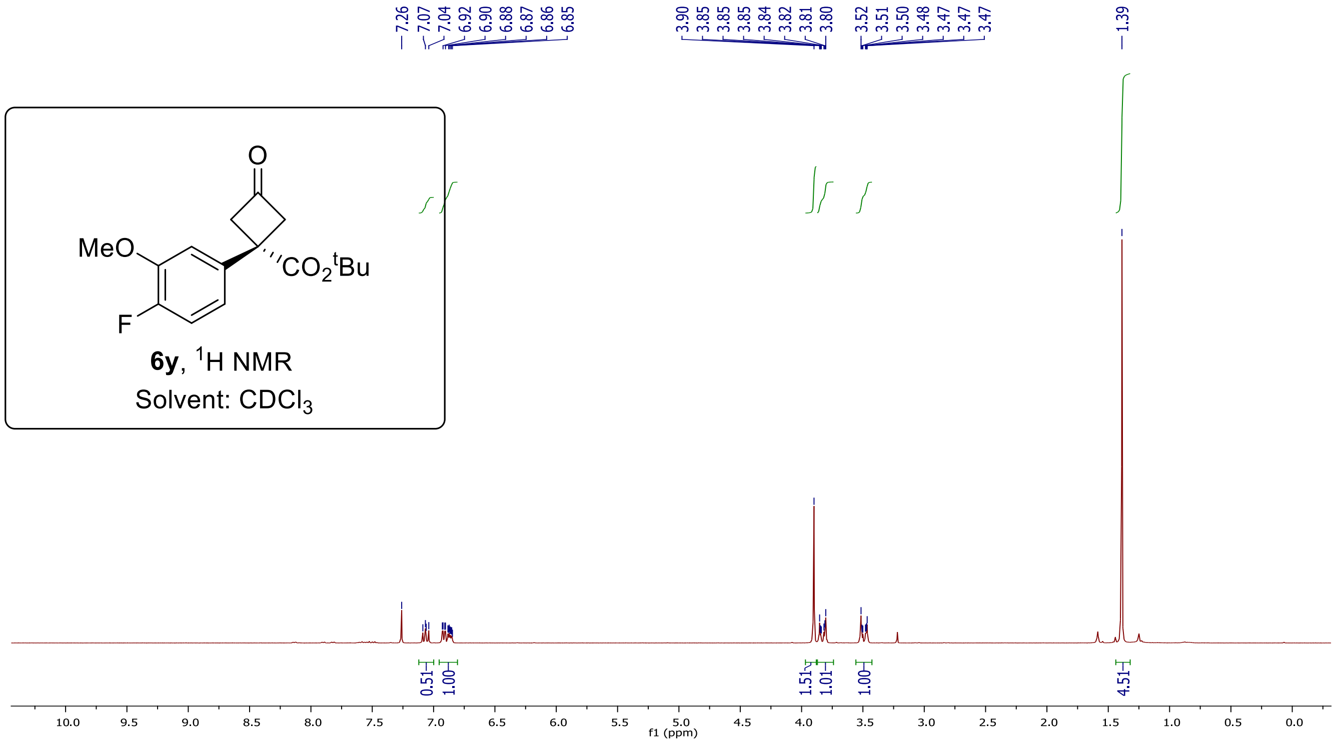

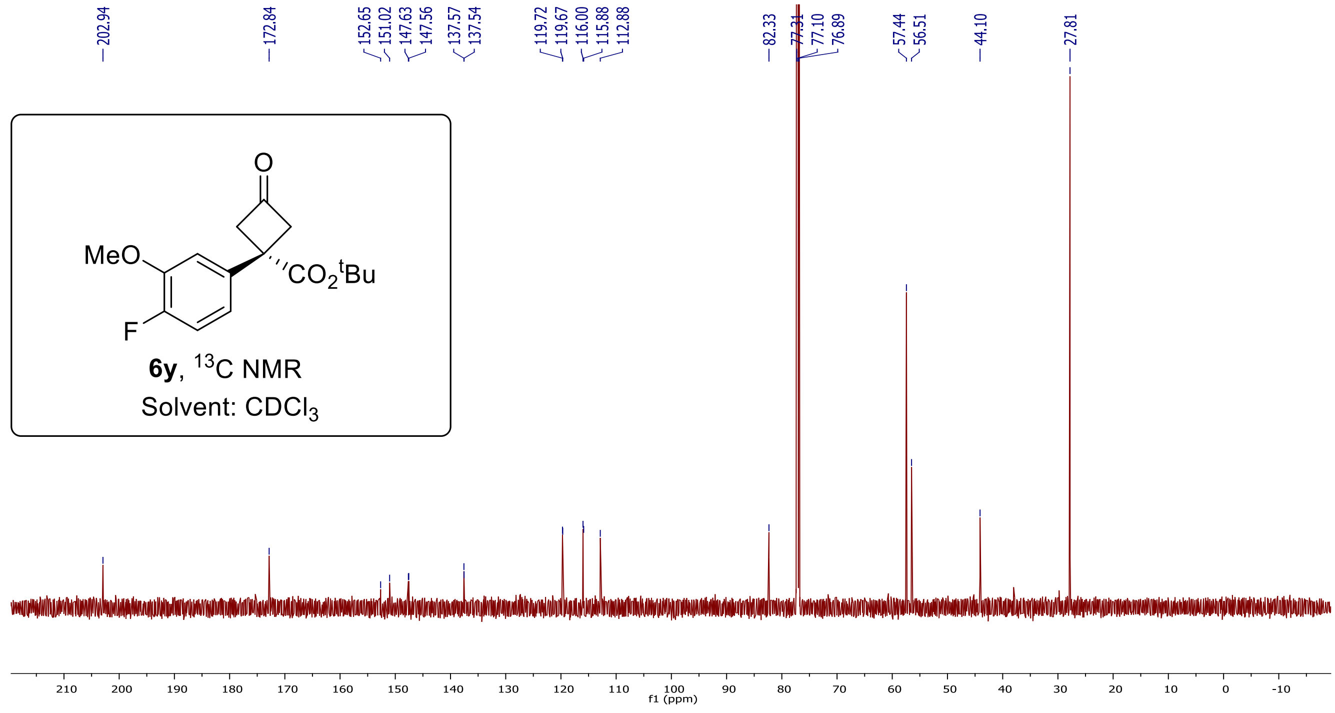

Supplementary Figure 94. NMR spectra of **6y**

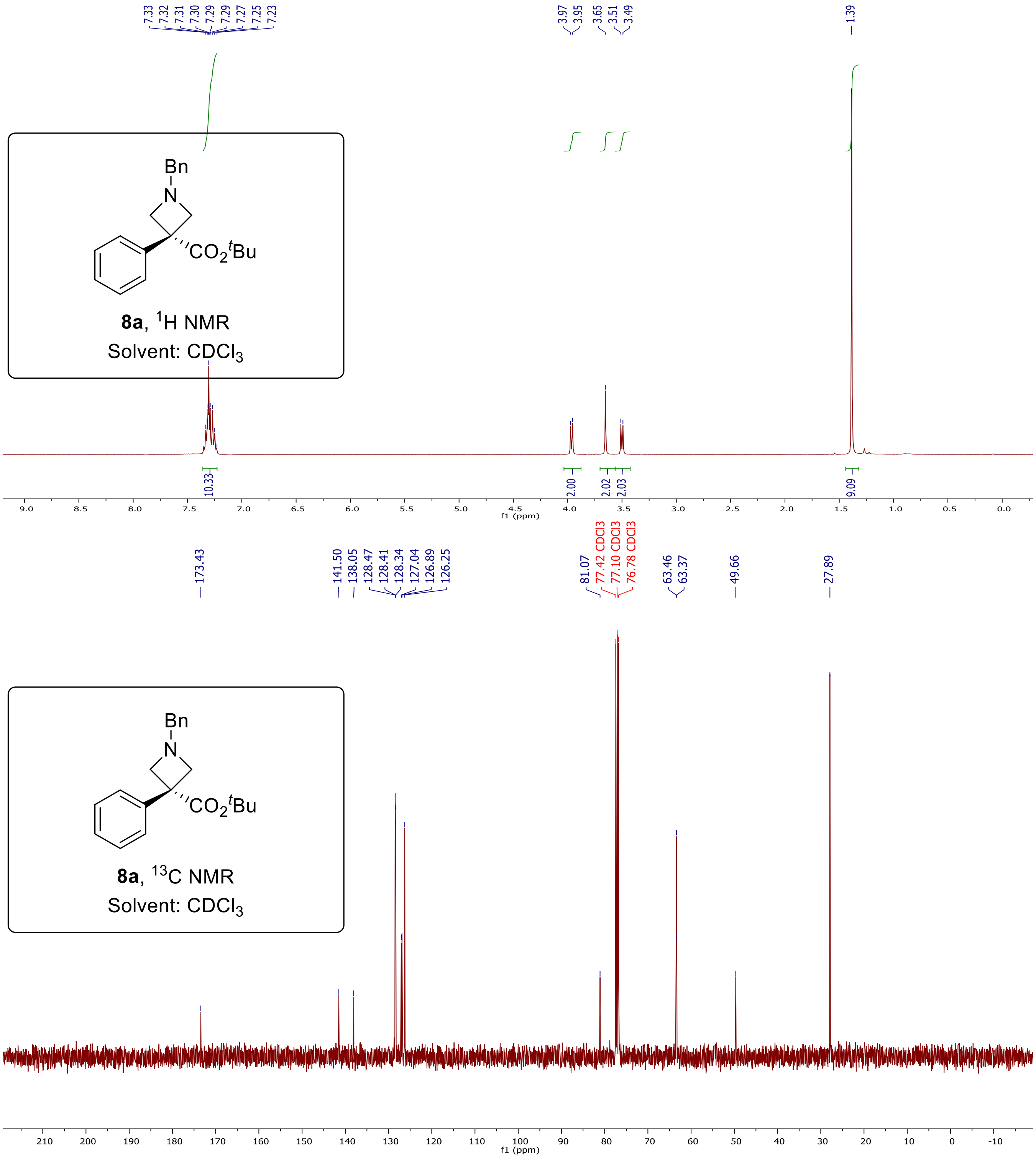

Supplementary Figure 95. NMR spectra of **8a**

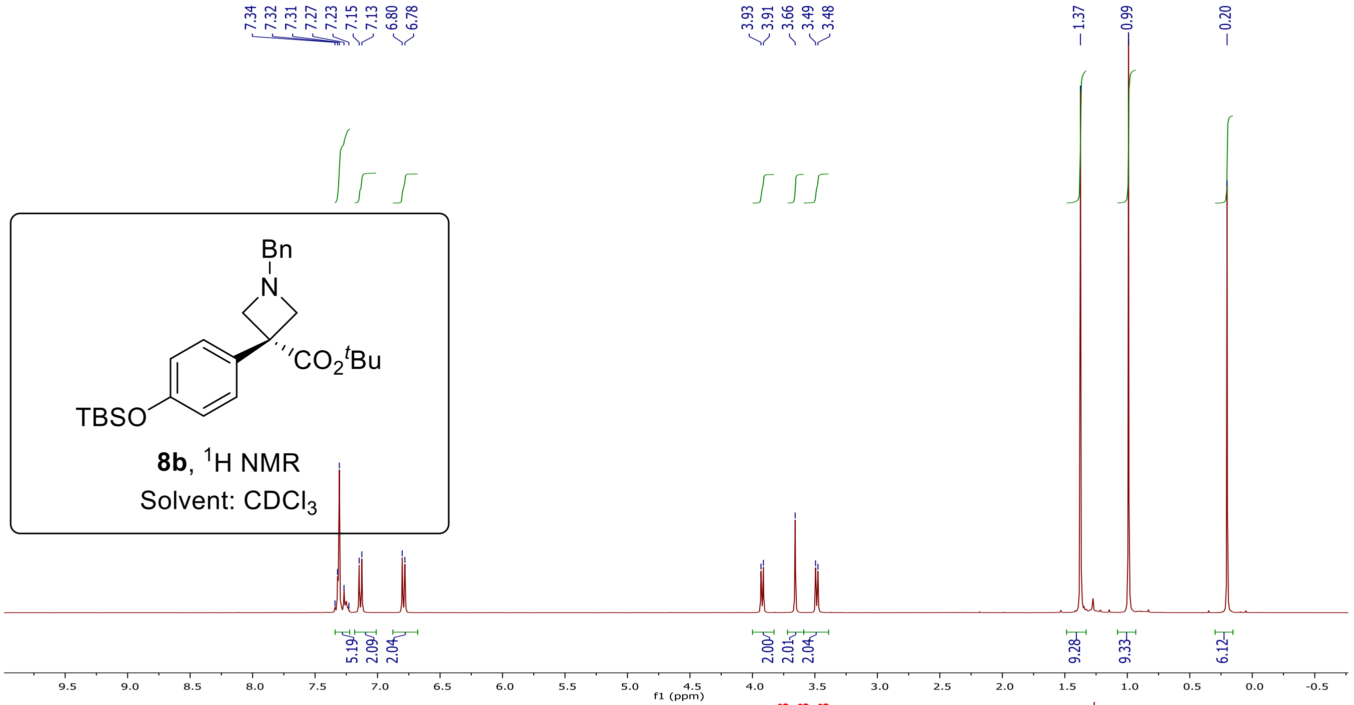

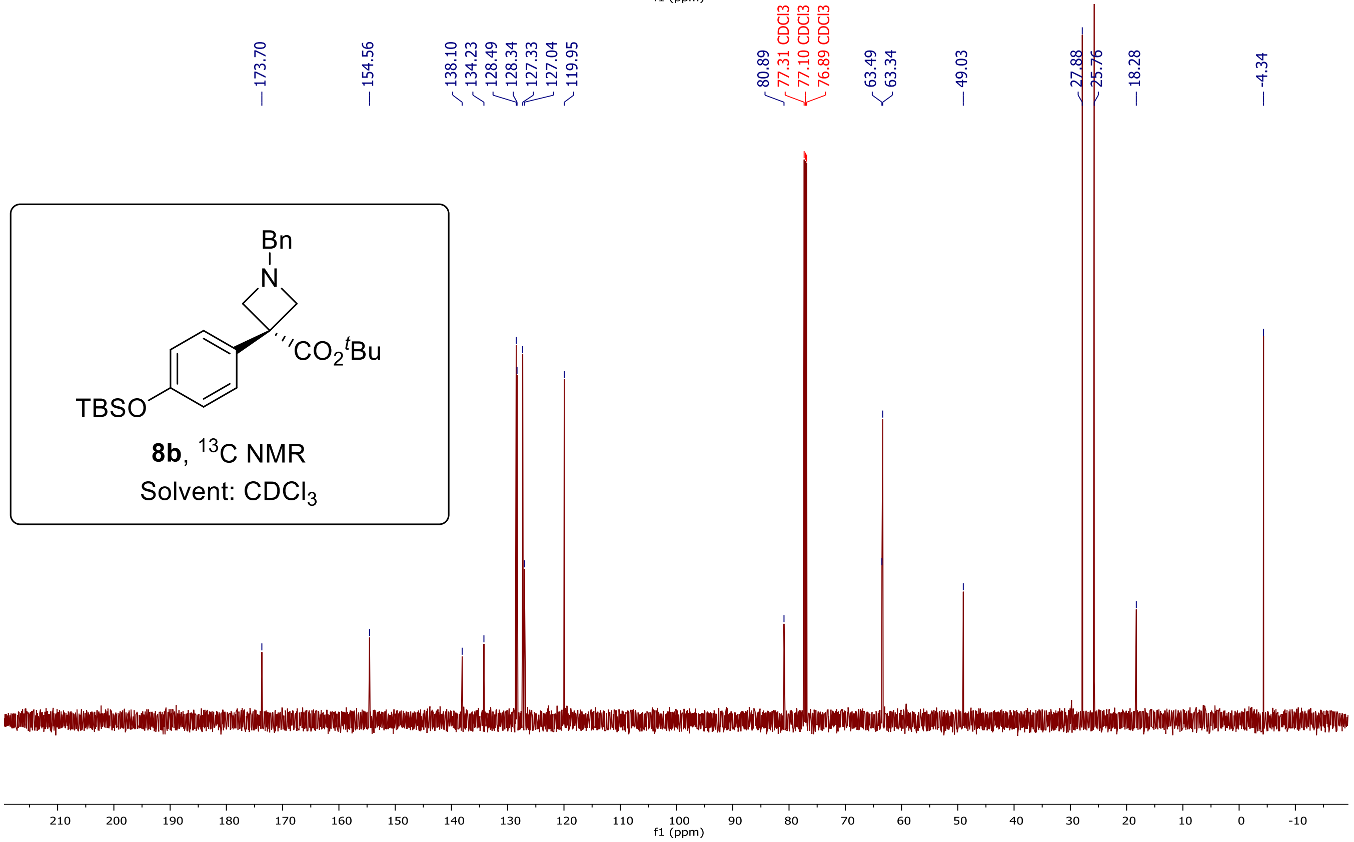

Supplementary Figure 96. NMR spectra of **8b**

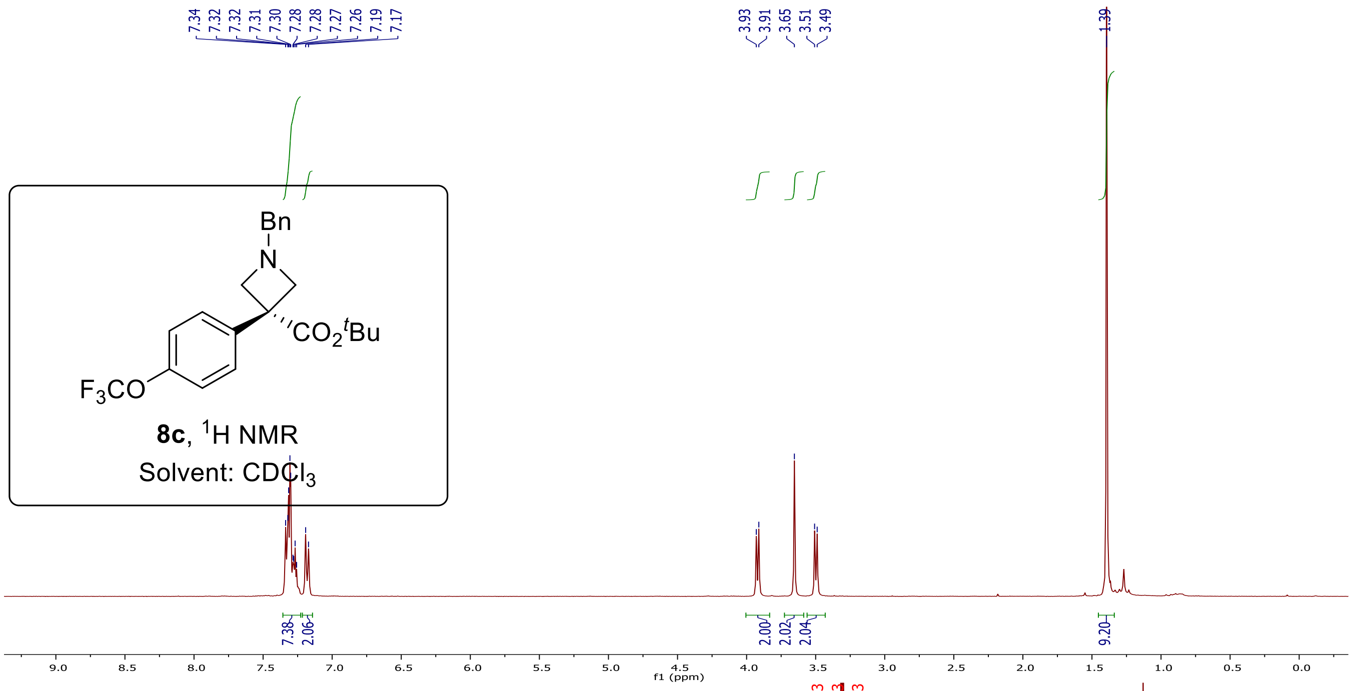

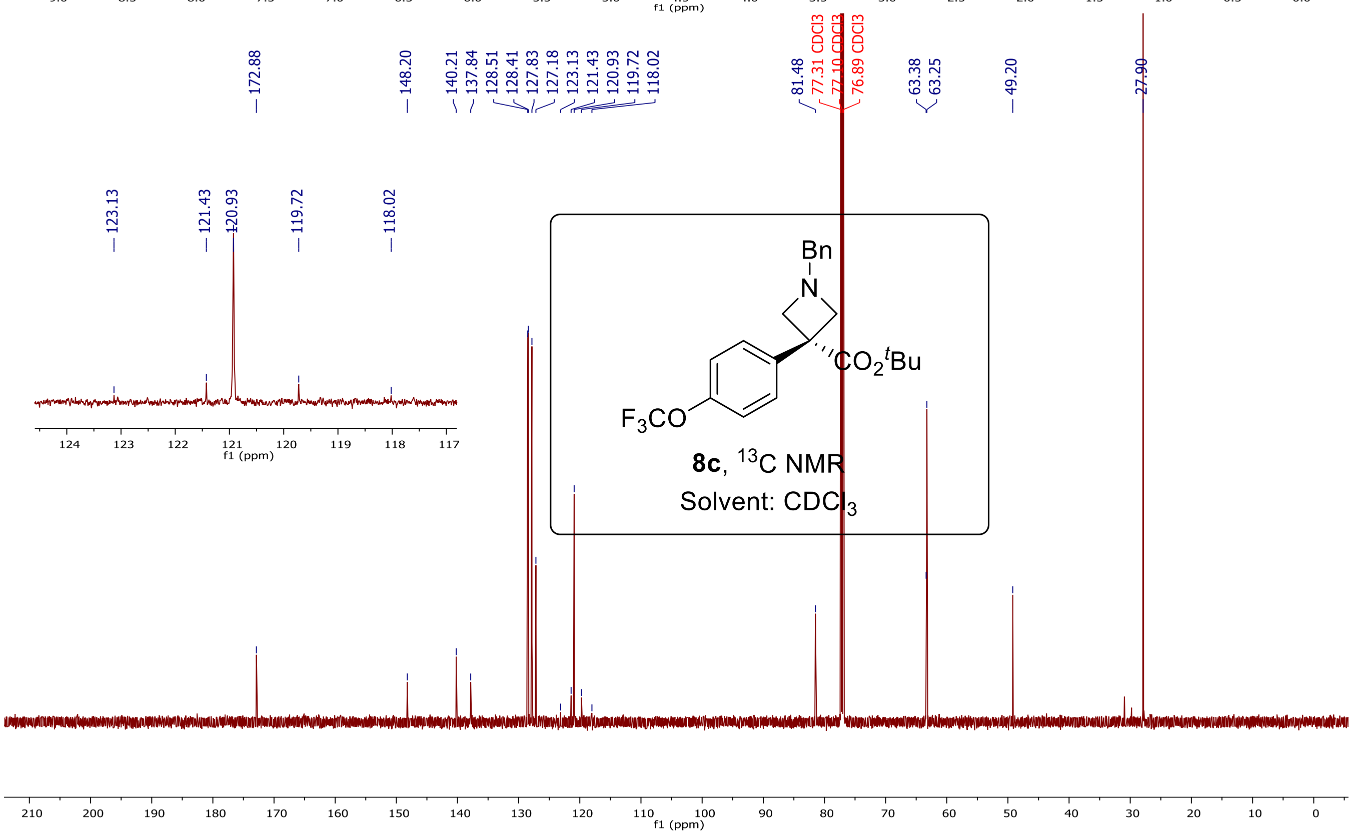

Supplementary Figure 97. NMR spectra of **8c**

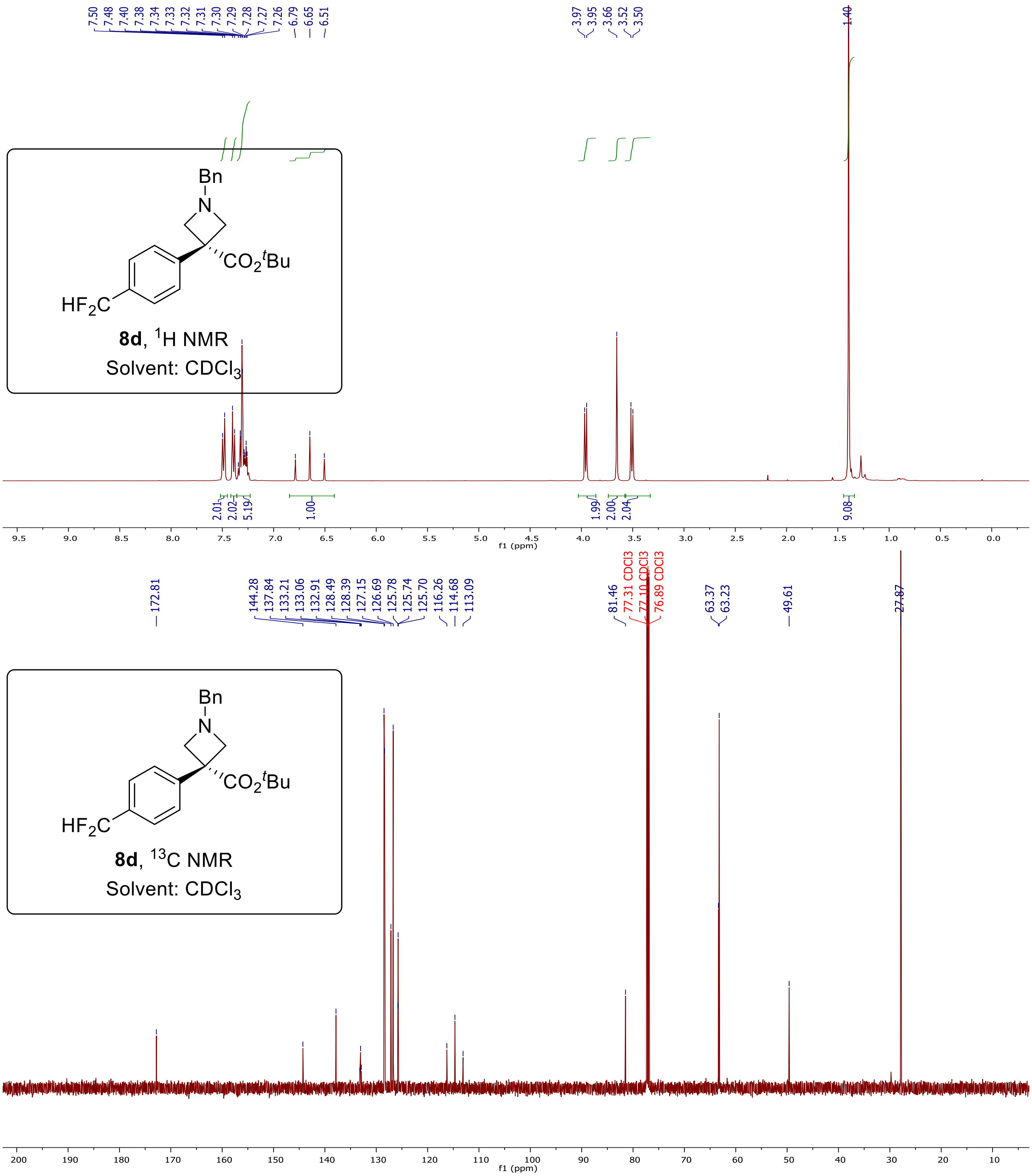

Supplementary Figure 98. NMR spectra of **8d**

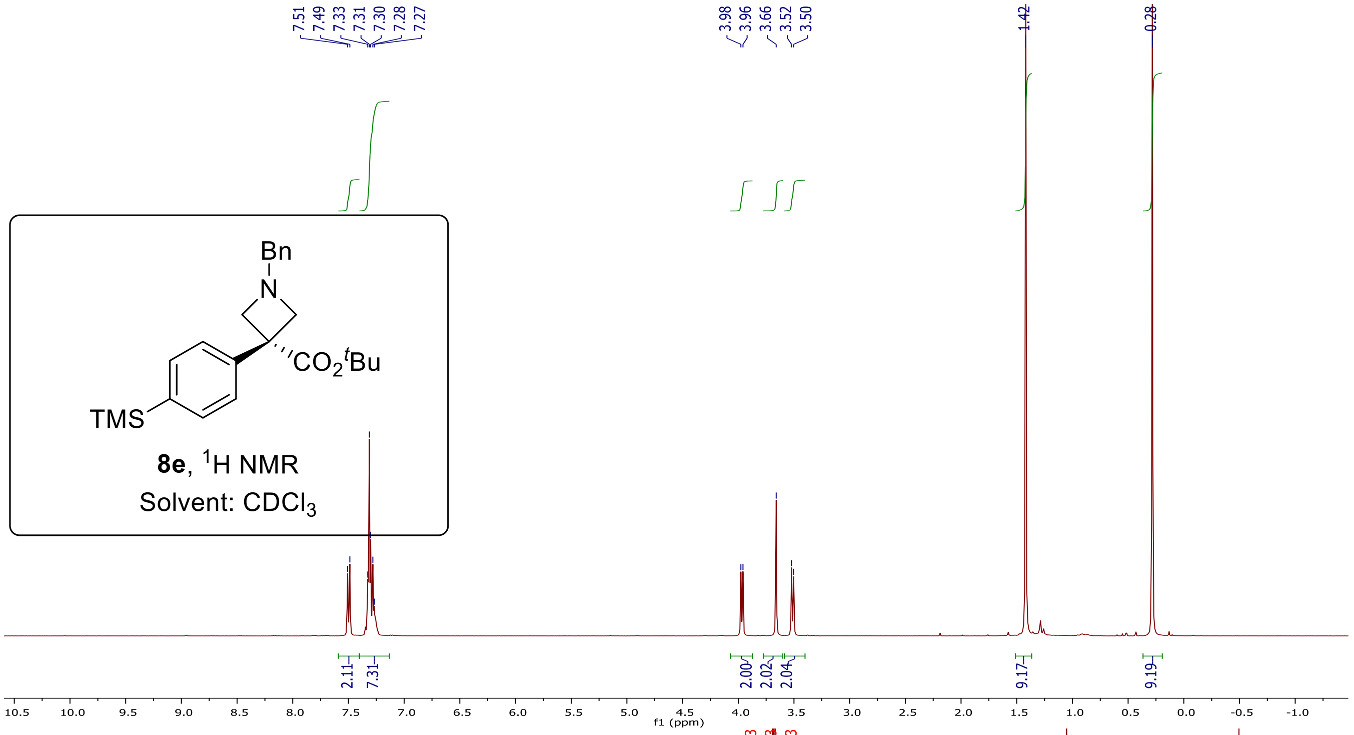

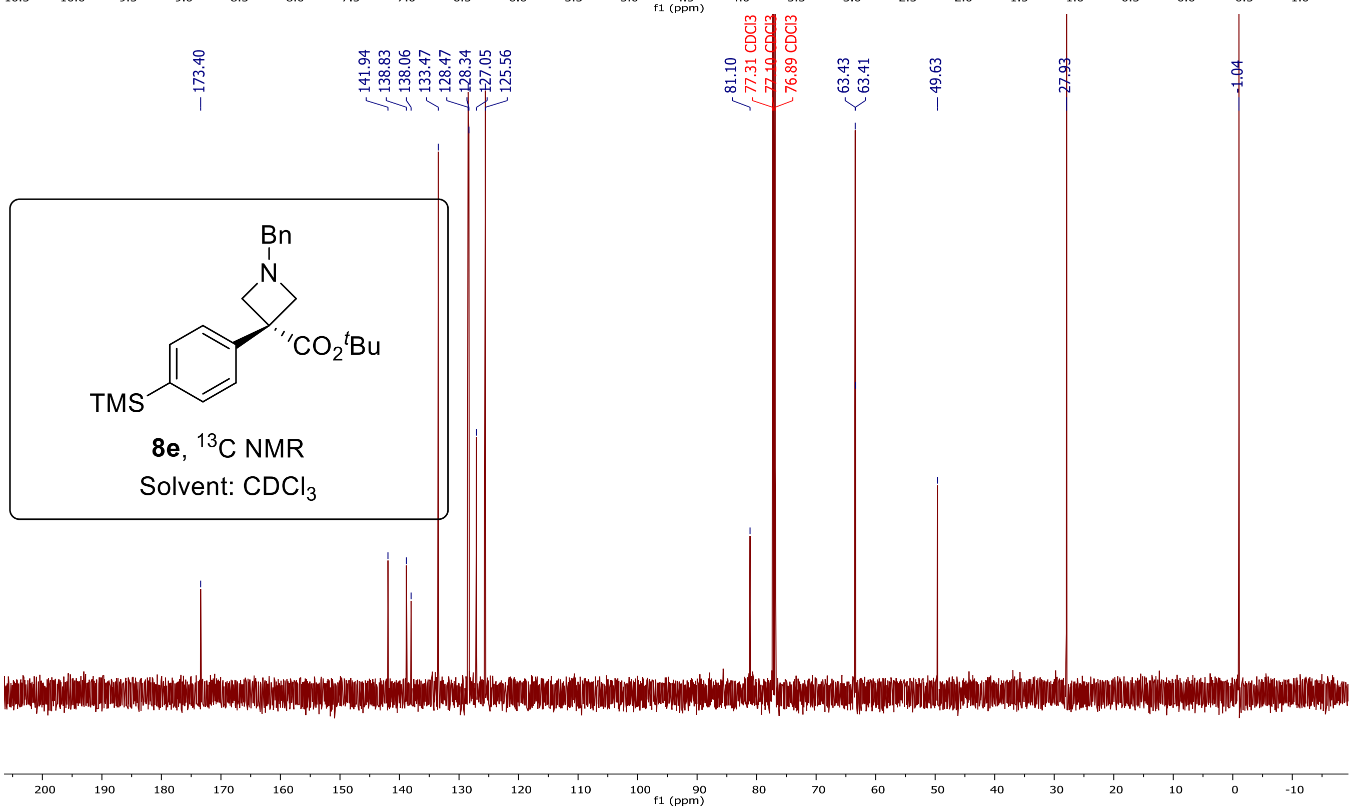

Supplementary Figure 99. NMR spectra of **8e**

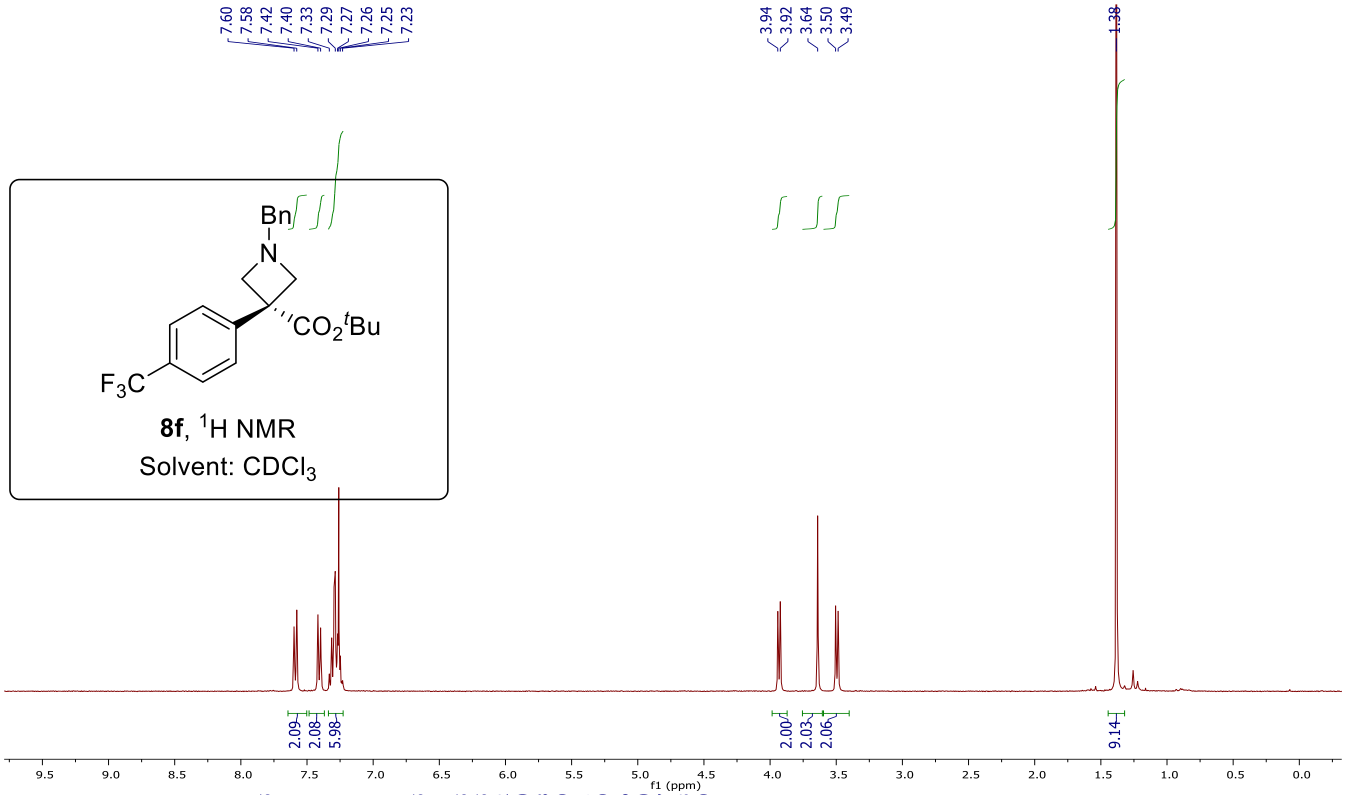

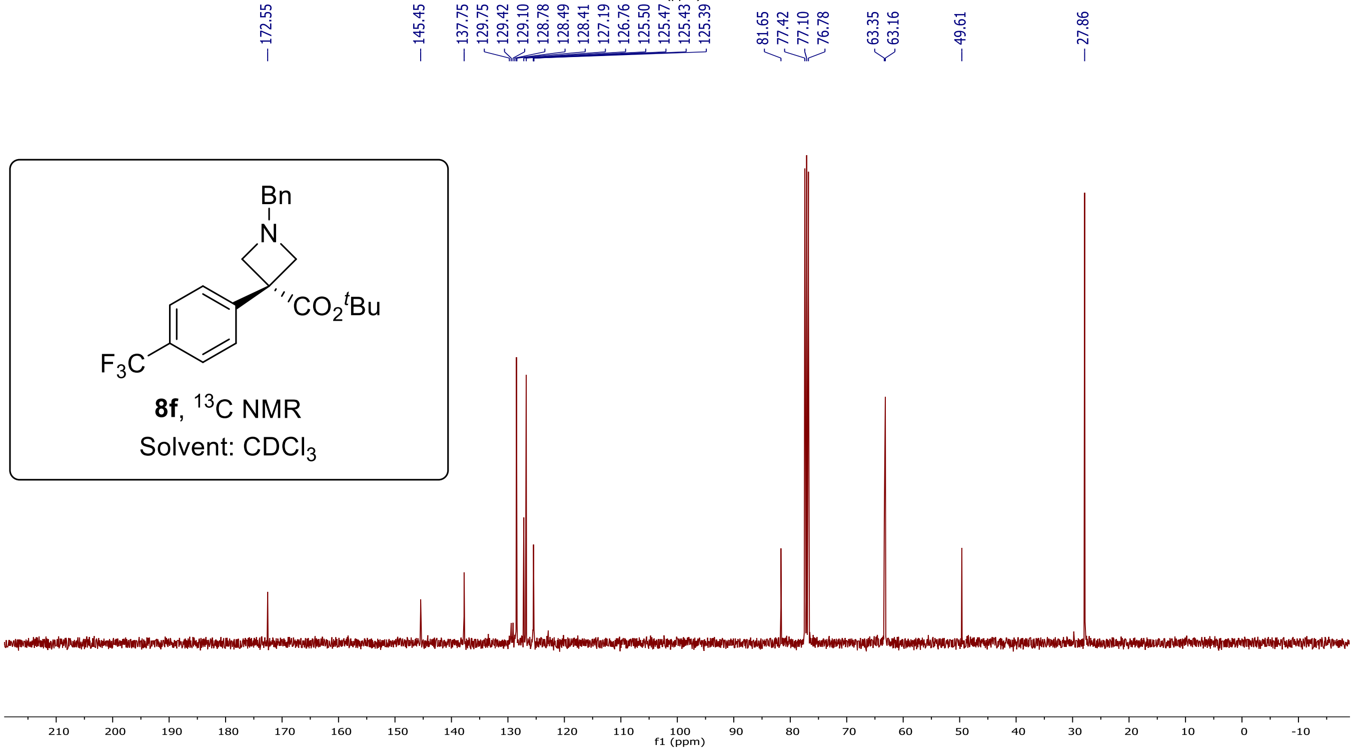

Supplementary Figure 100. NMR spectra of **8f**

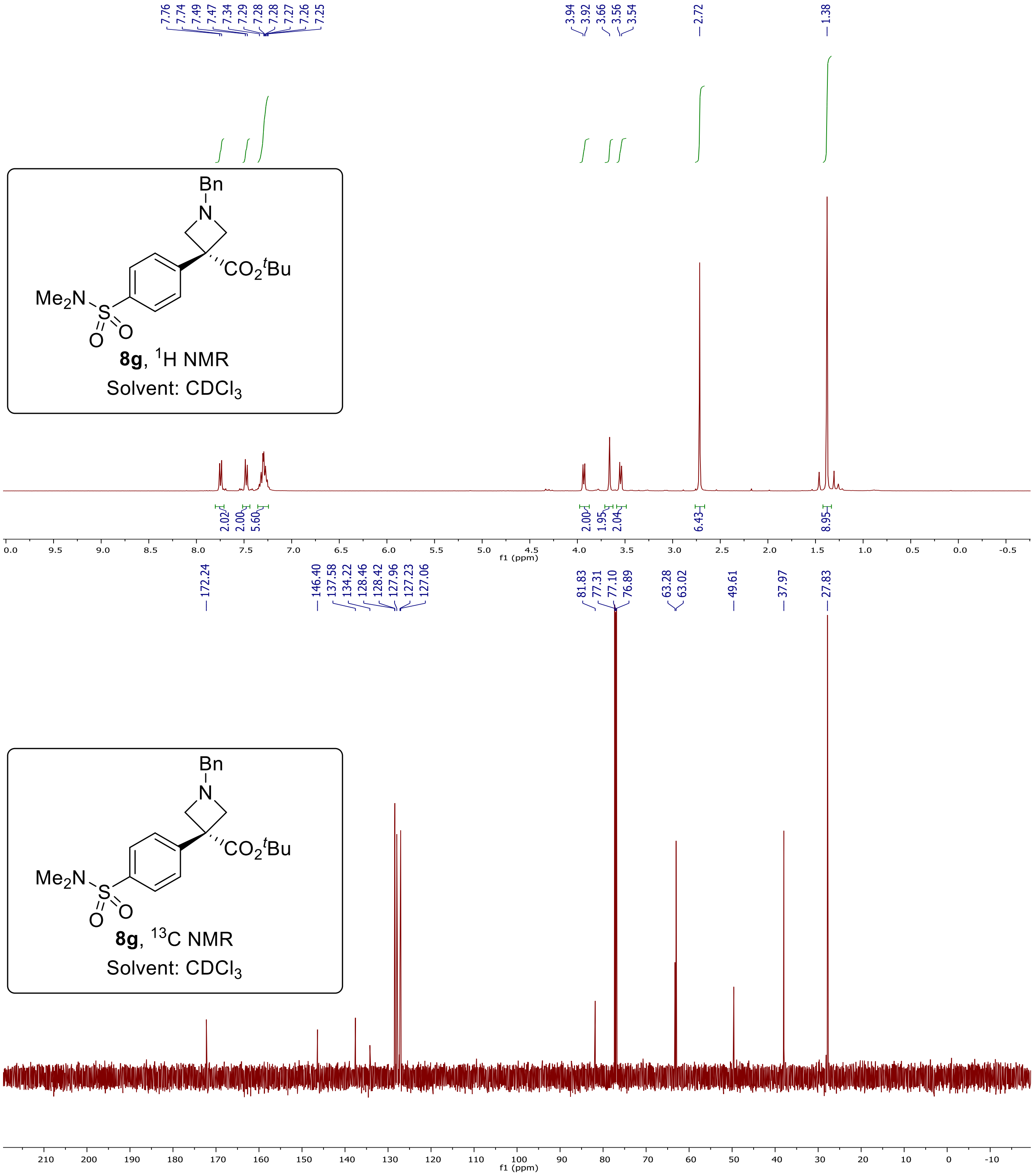

Supplementary Figure 101. NMR spectra of **8g**

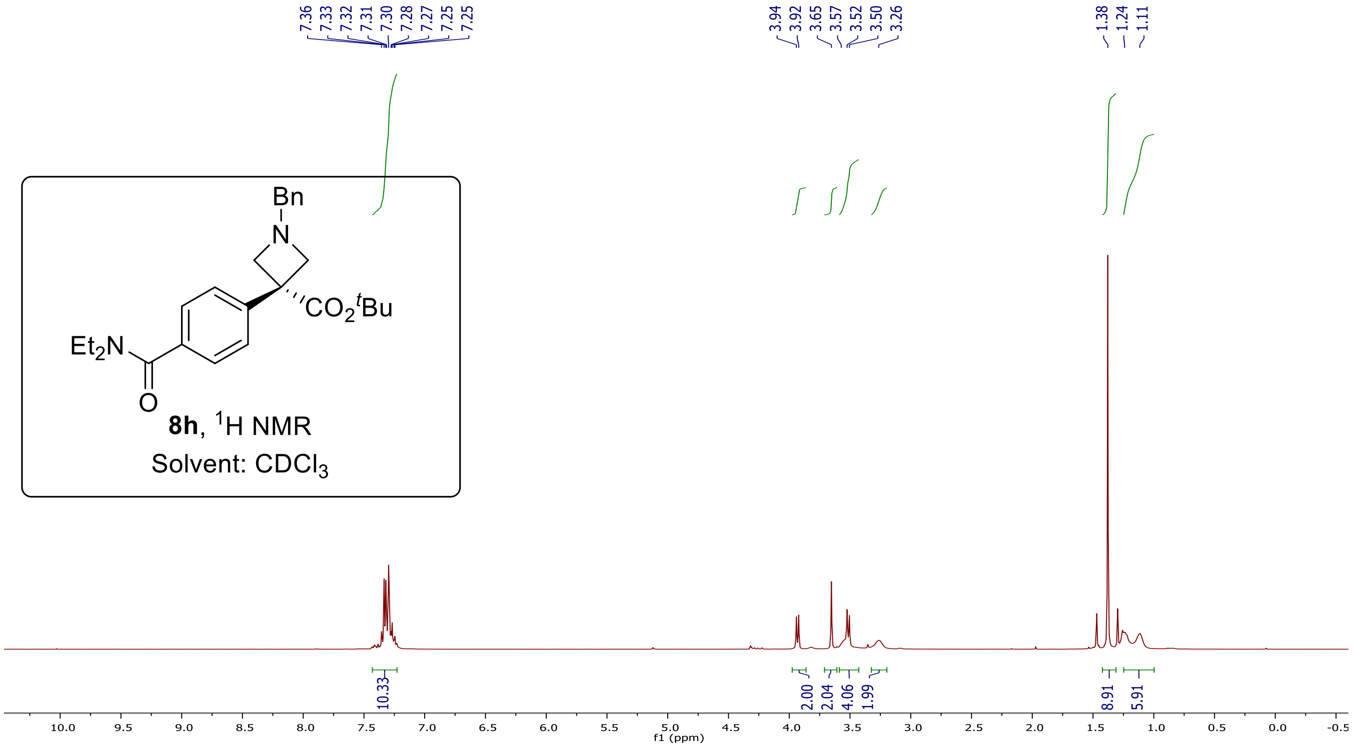

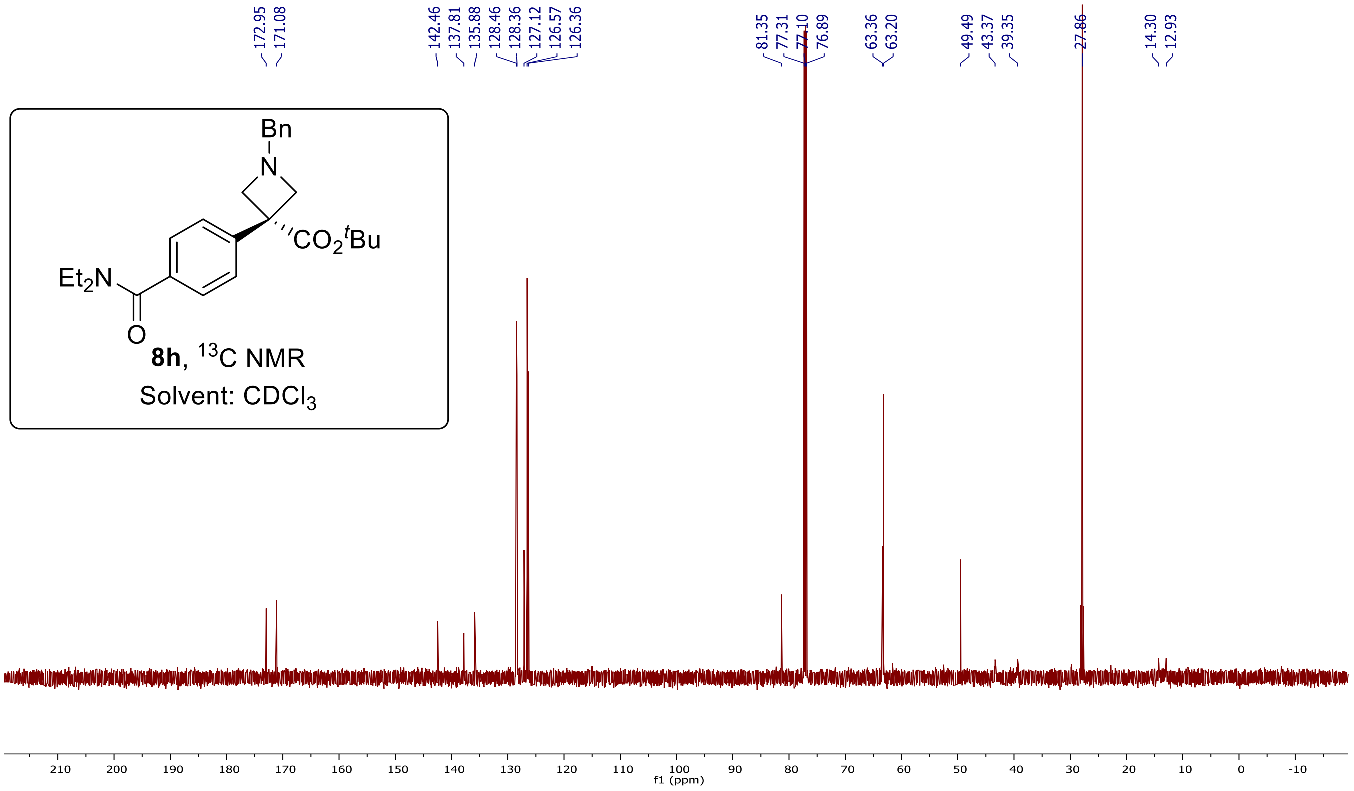

Supplementary Figure 102. NMR spectra of **8h**

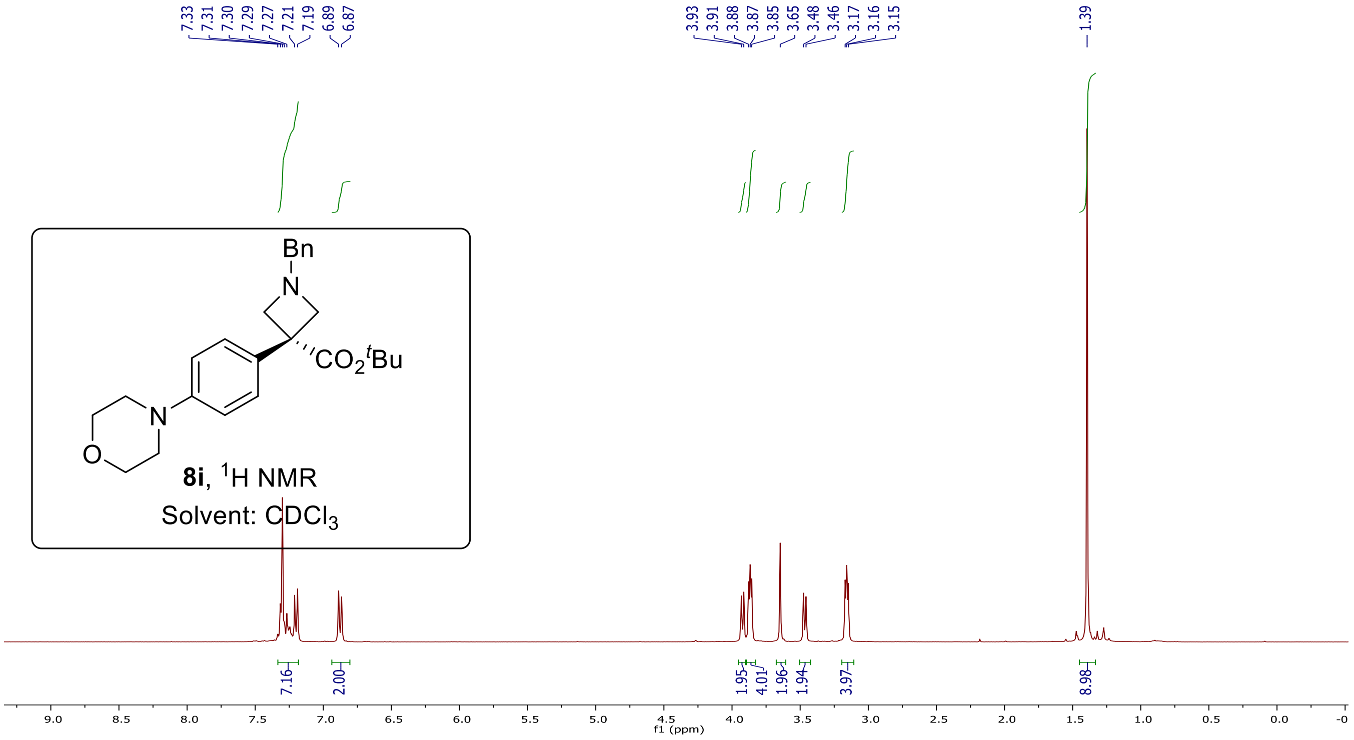

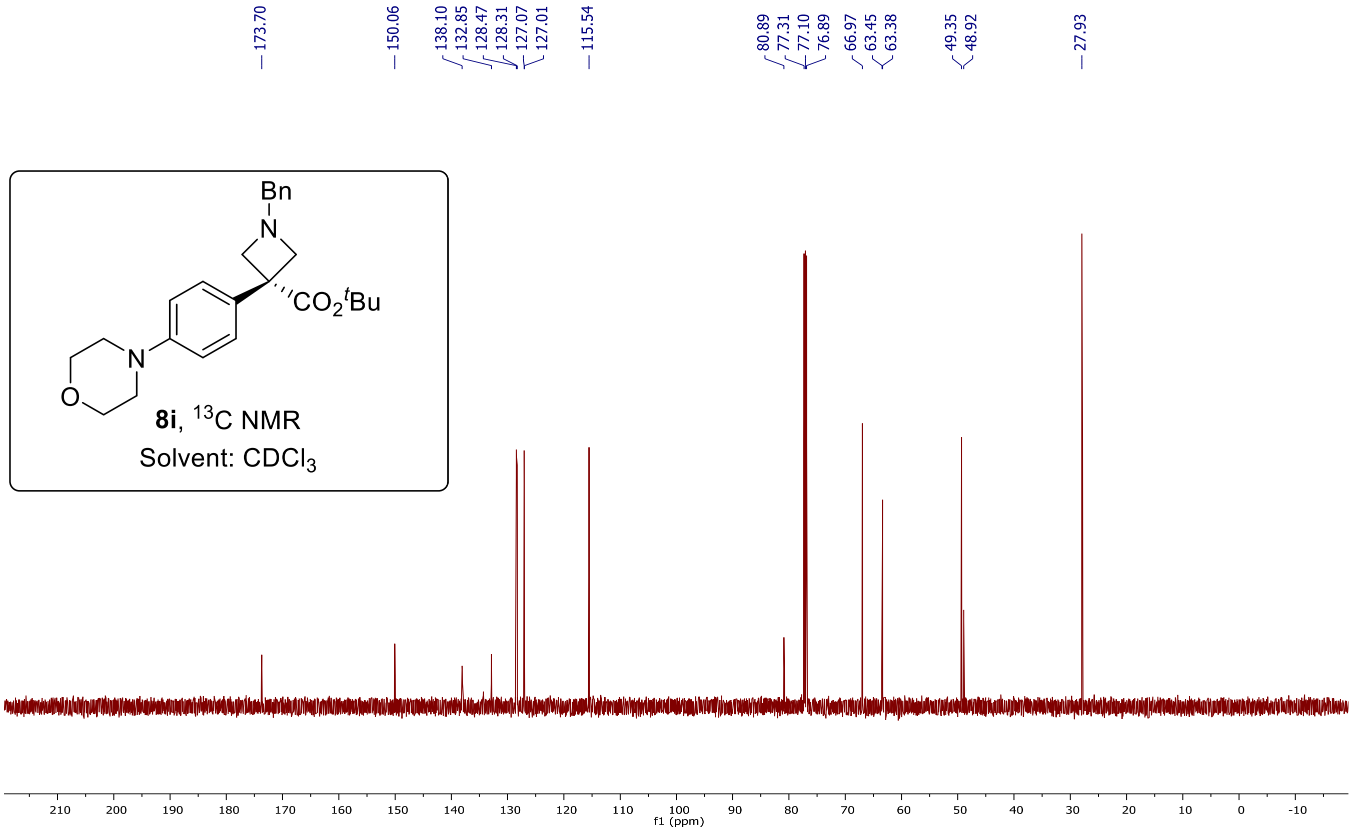

Supplementary Figure 103. NMR spectra of **8i**

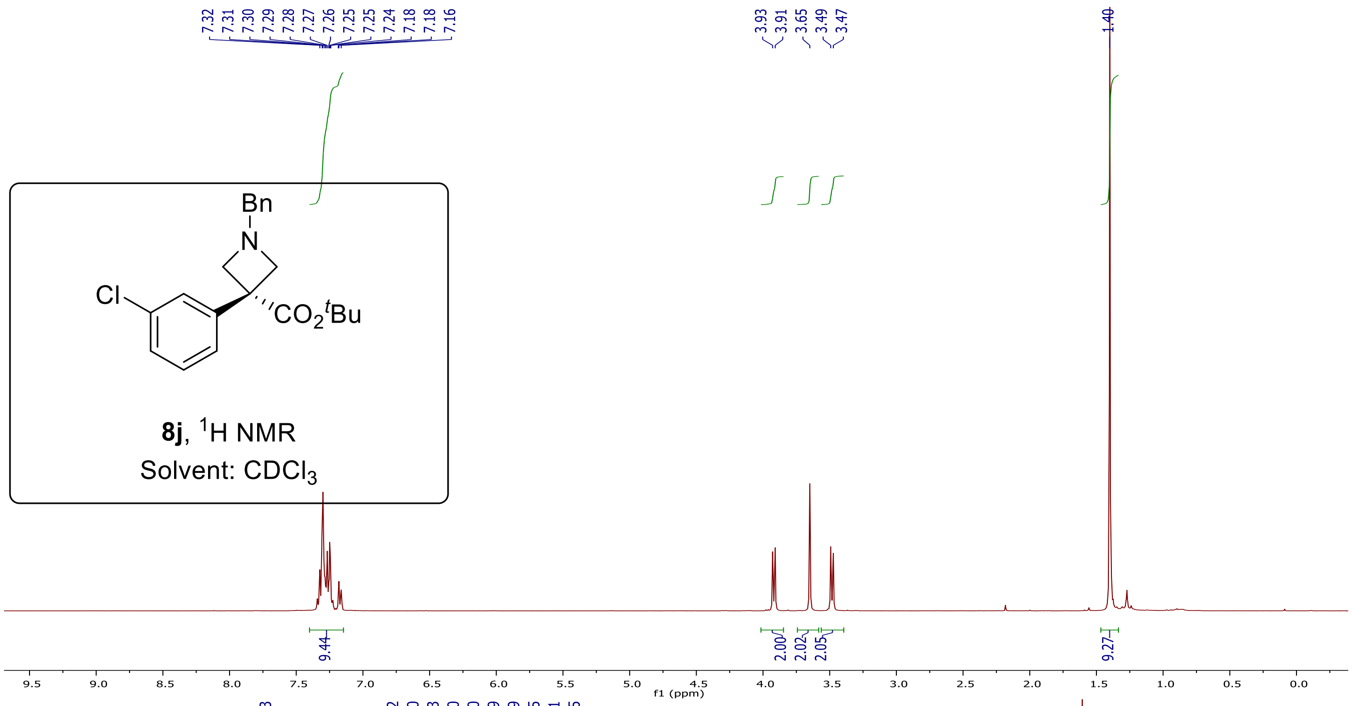

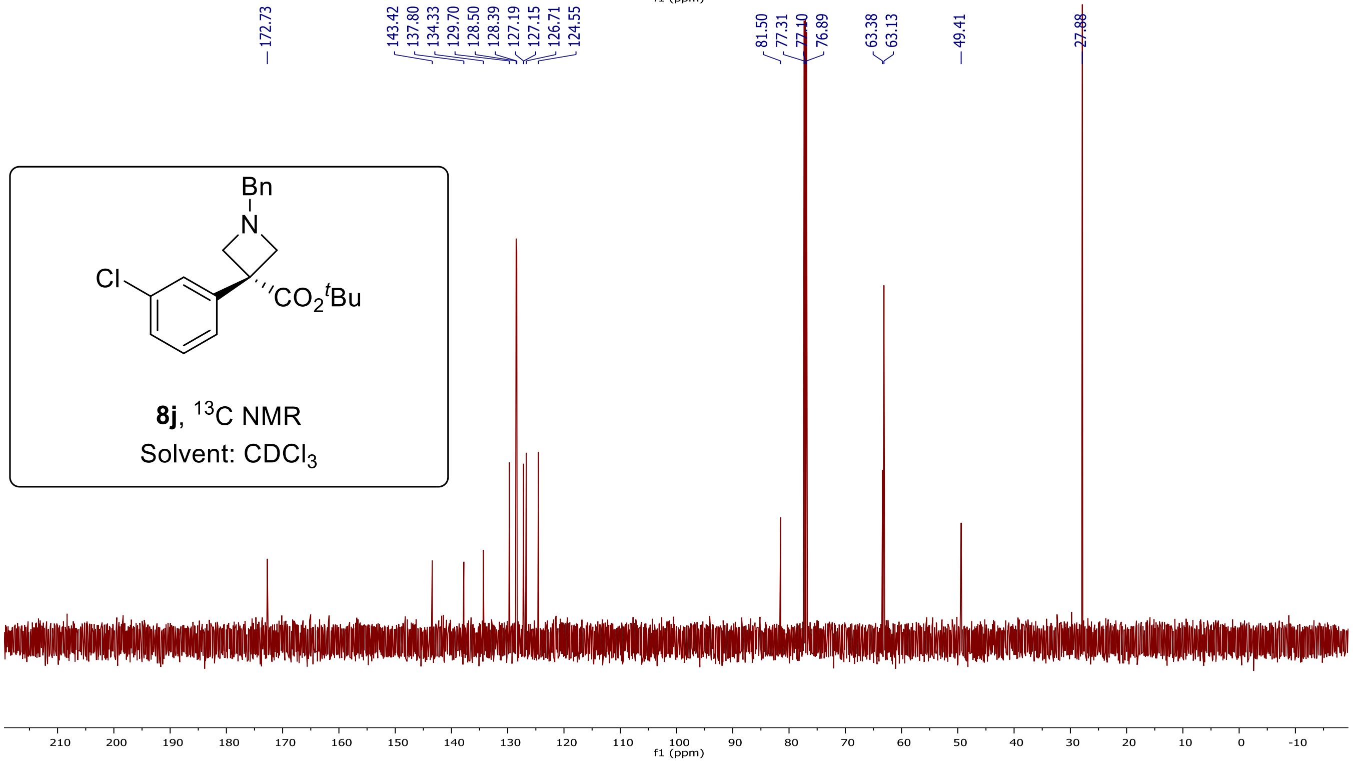

Supplementary Figure 104. NMR spectra of **8j**

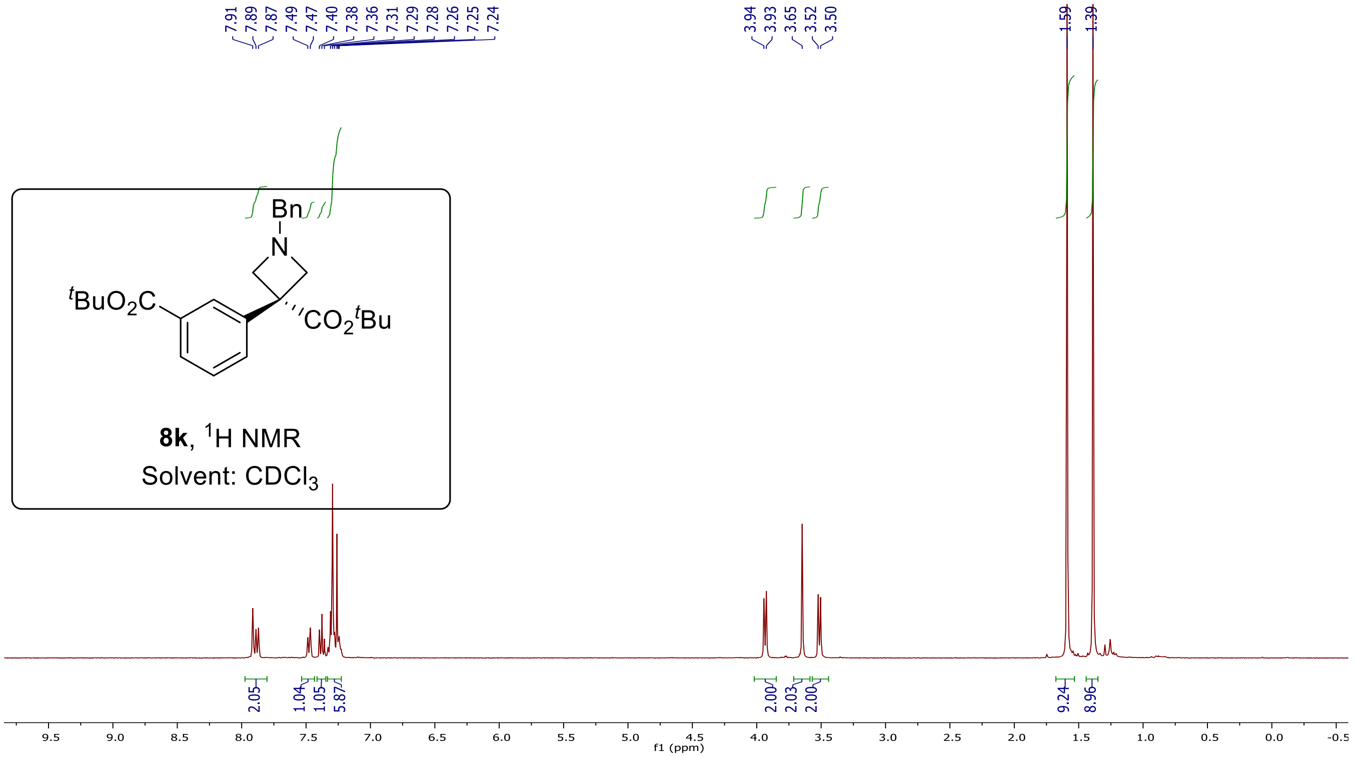

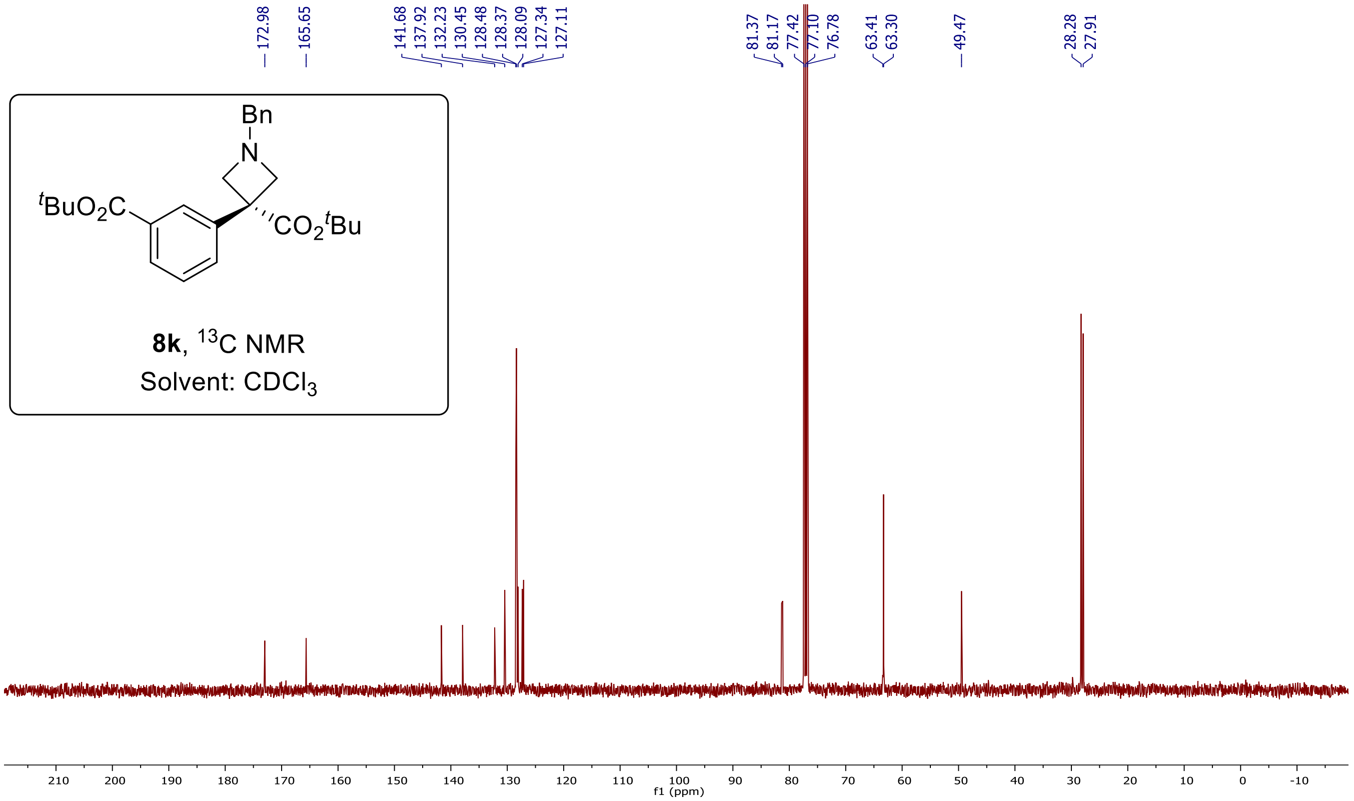

Supplementary Figure 105. NMR spectra of **8k**

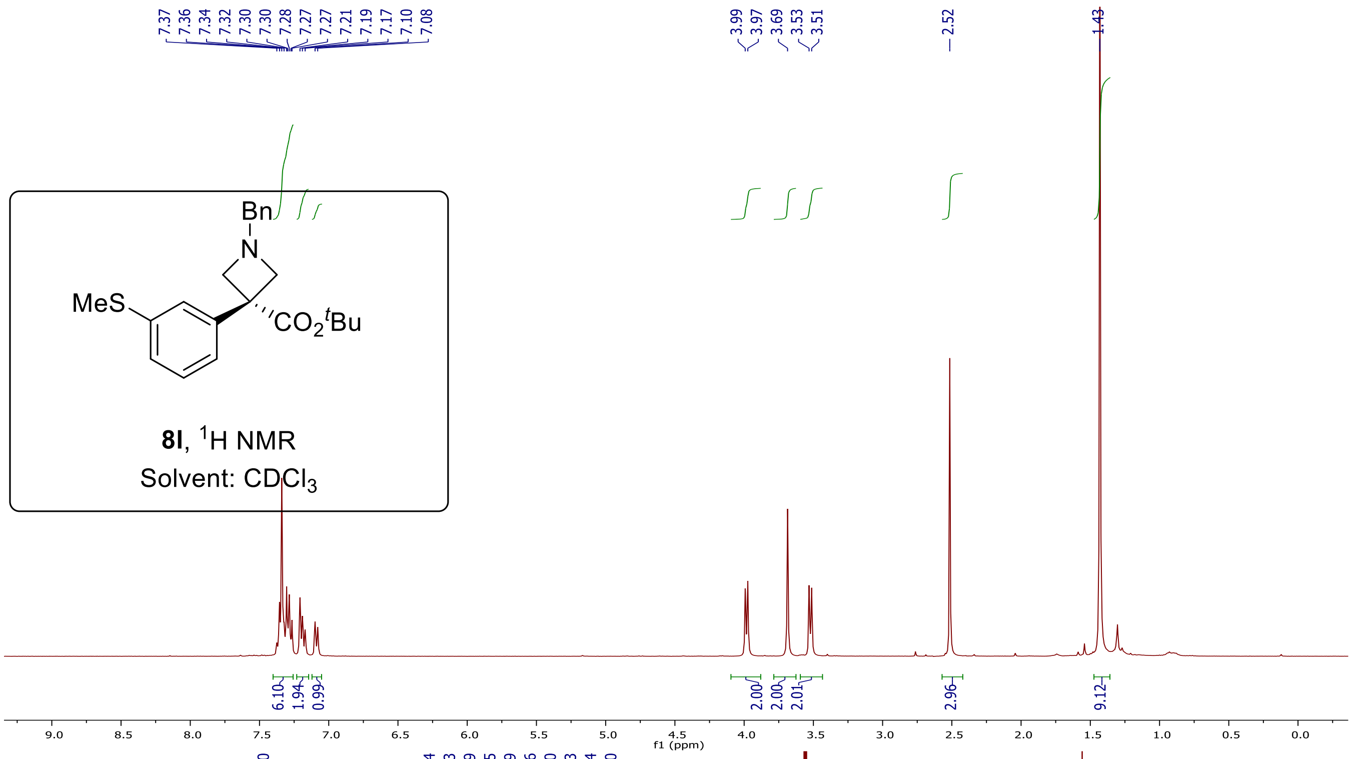

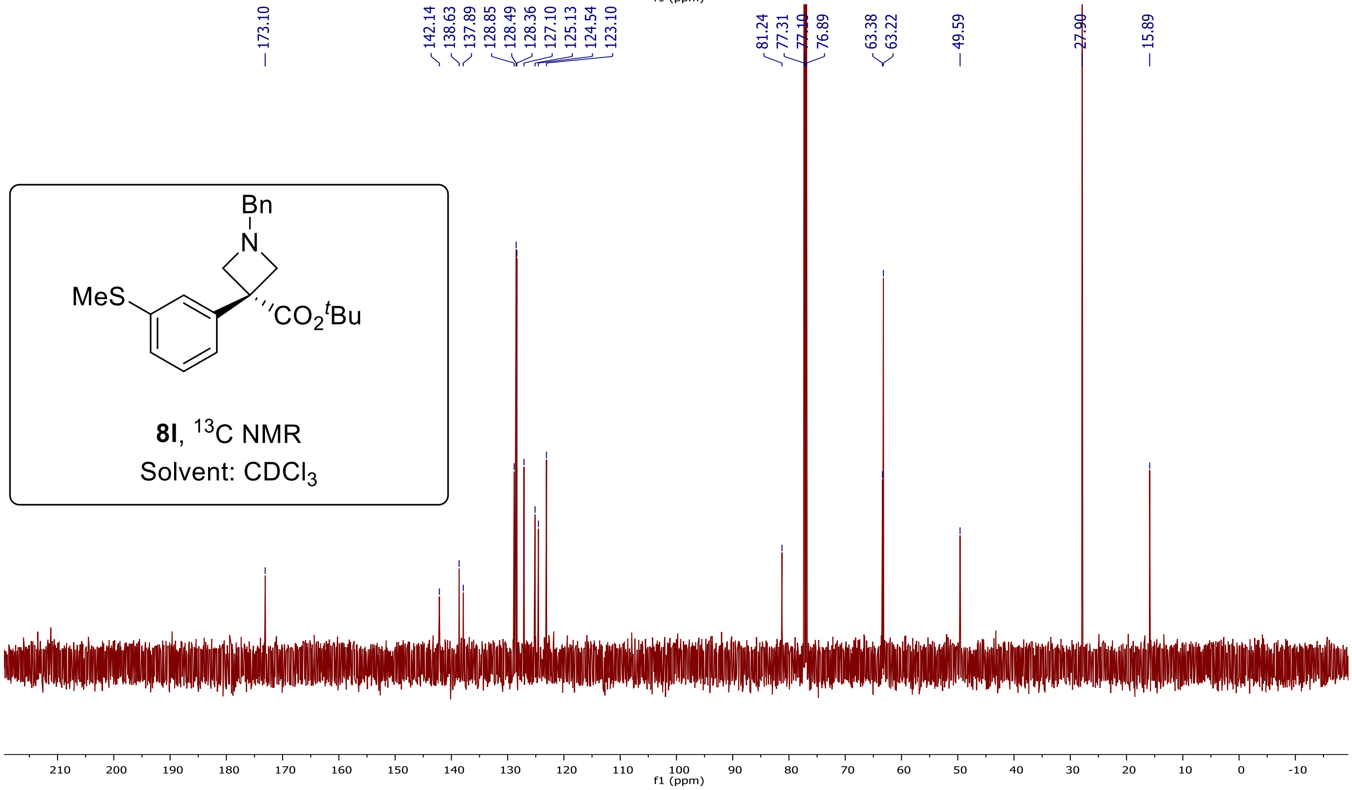

Supplementary Figure 106. NMR spectra of **8l**

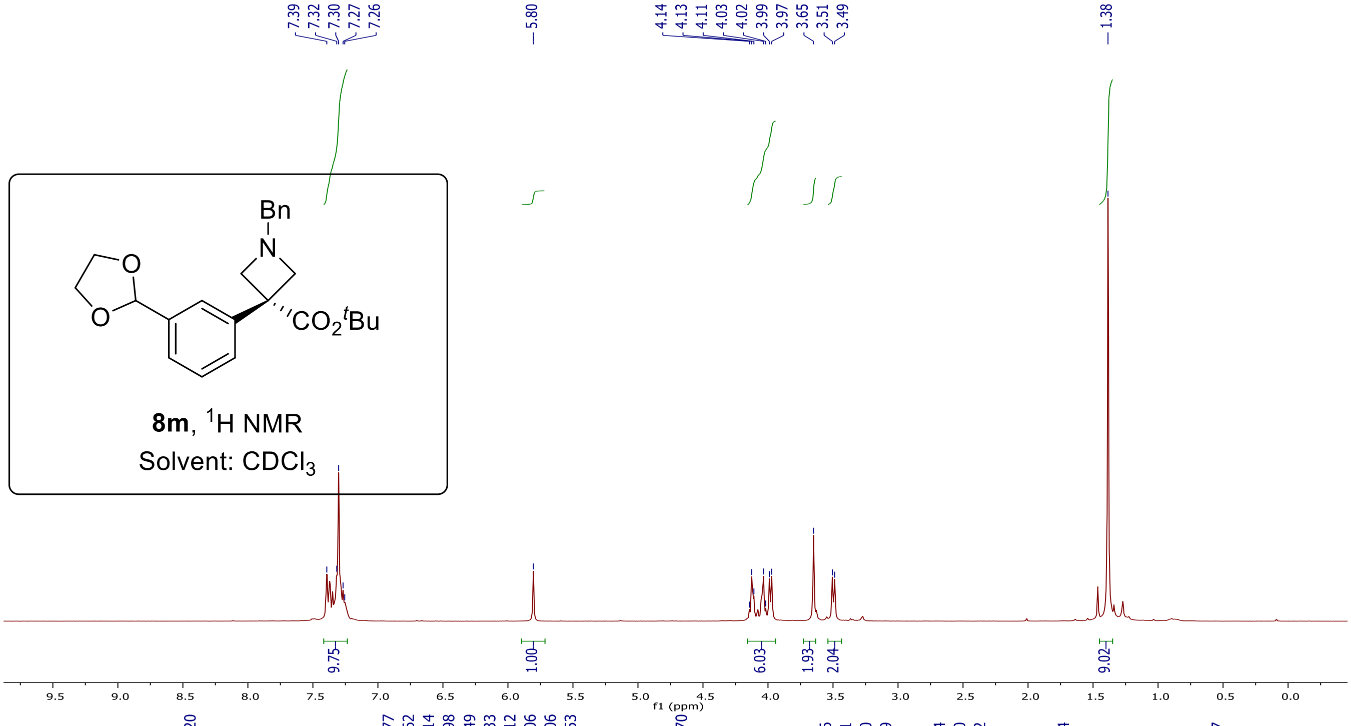

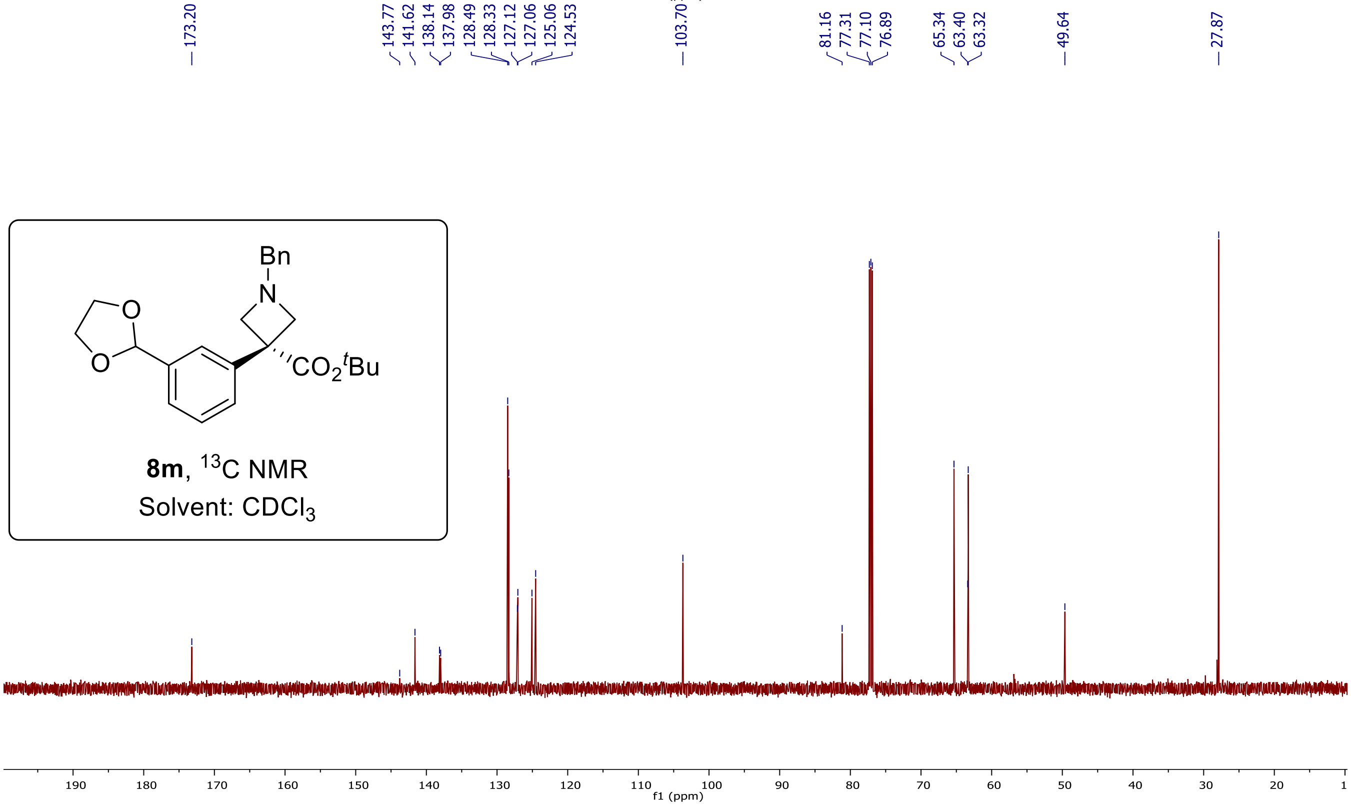

Supplementary Figure 107. NMR spectra of **8m**

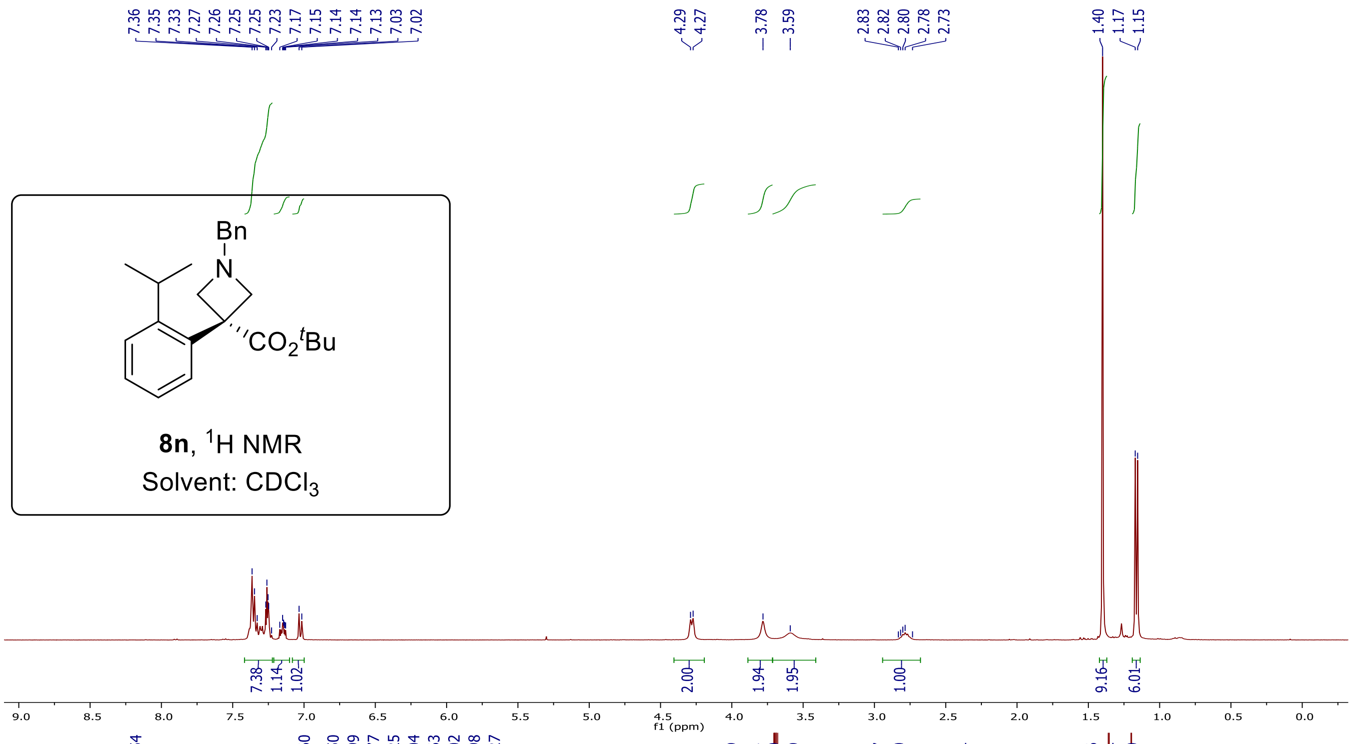

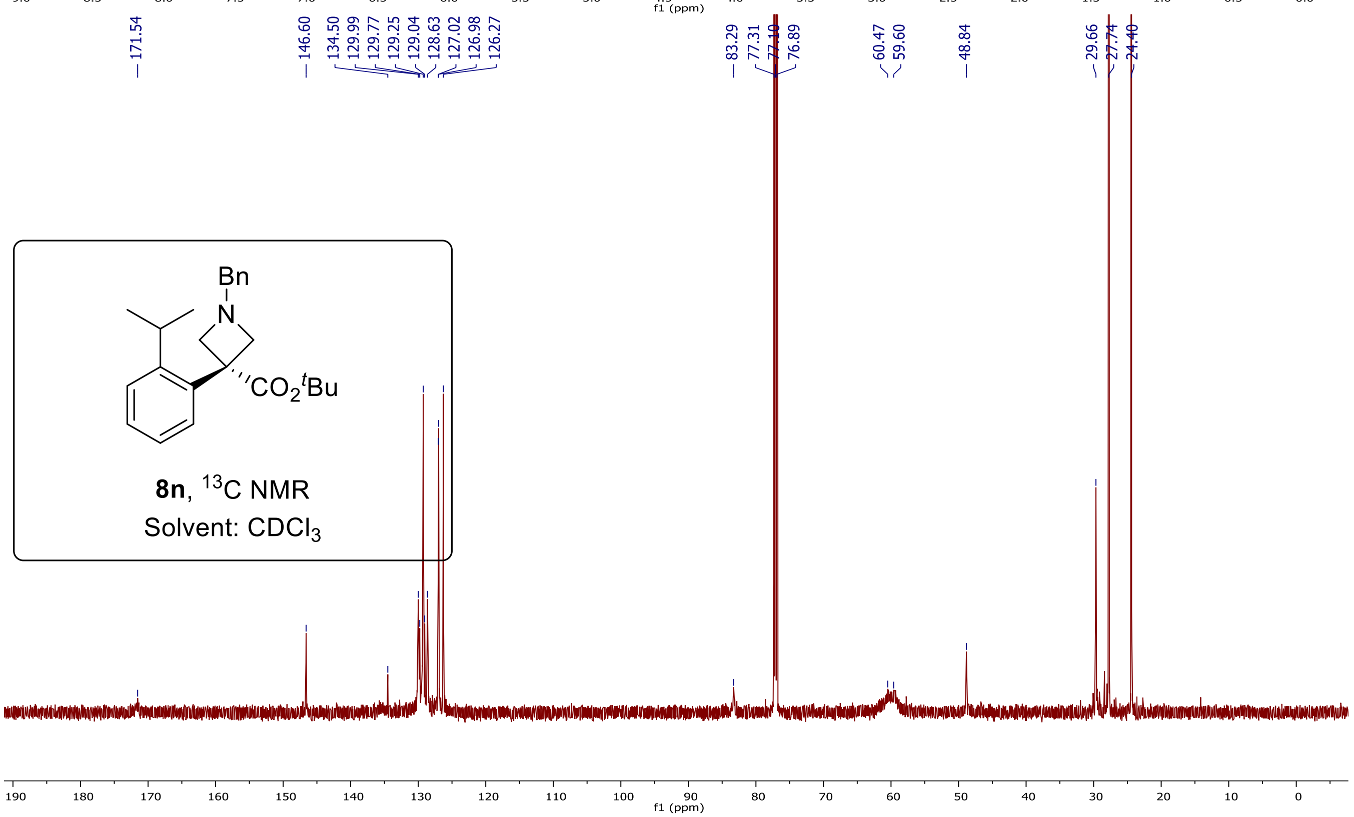

Supplementary Figure 108. NMR spectra of **8n**

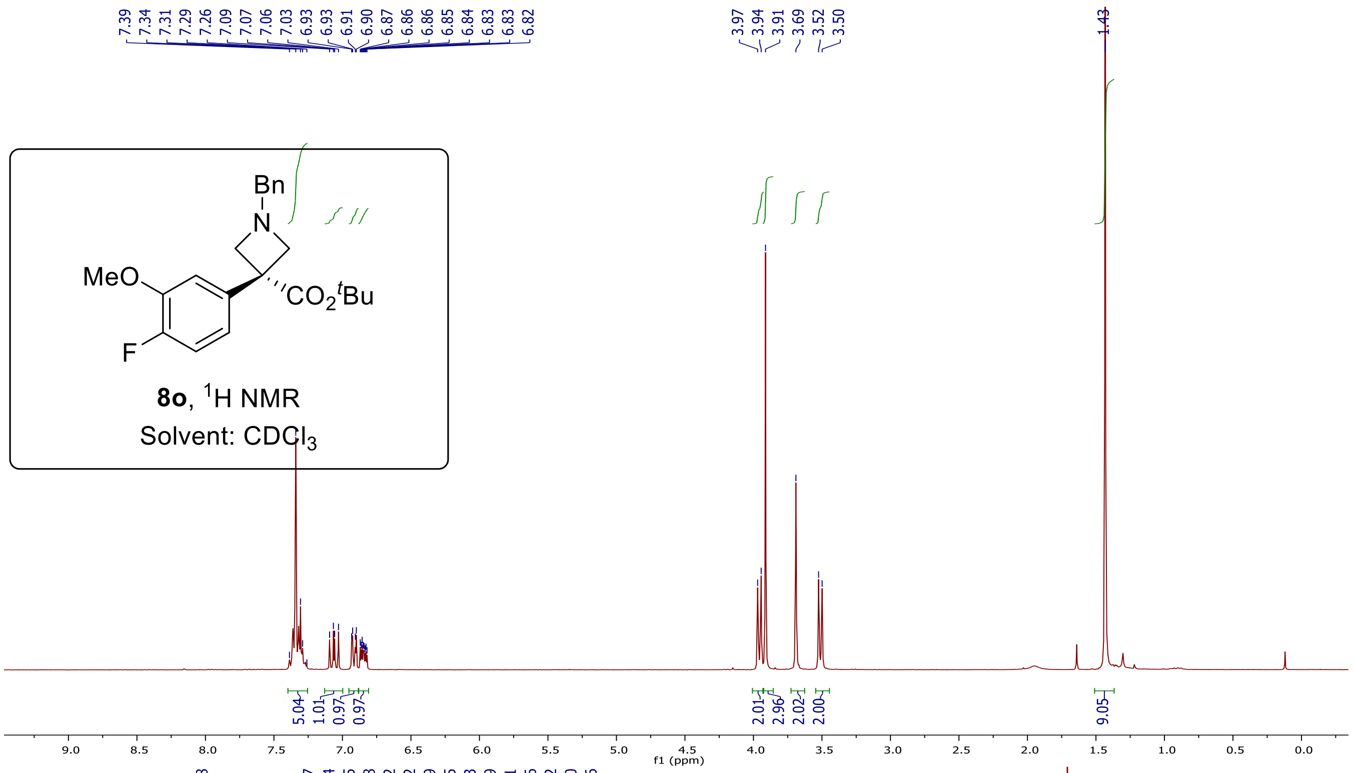

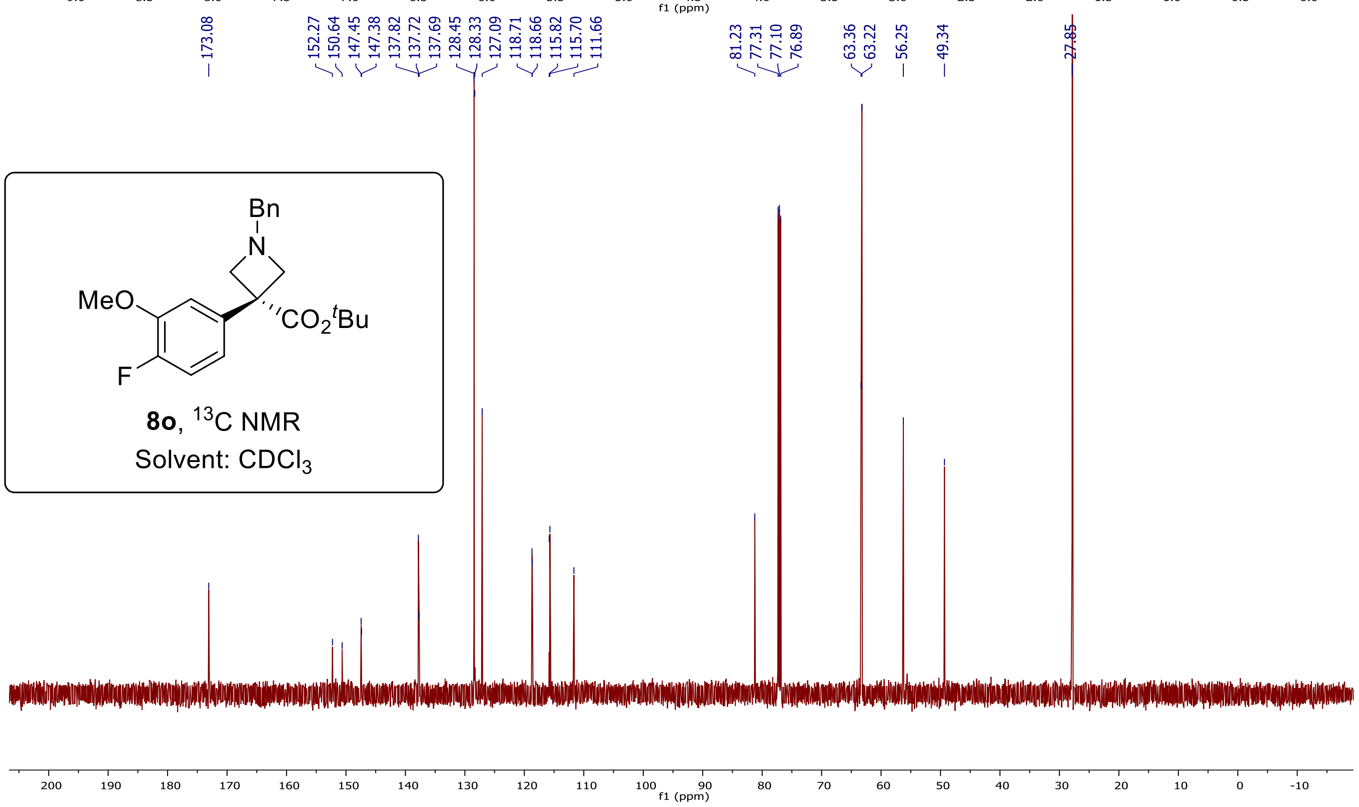

Supplementary Figure 109. NMR spectra of **8o**

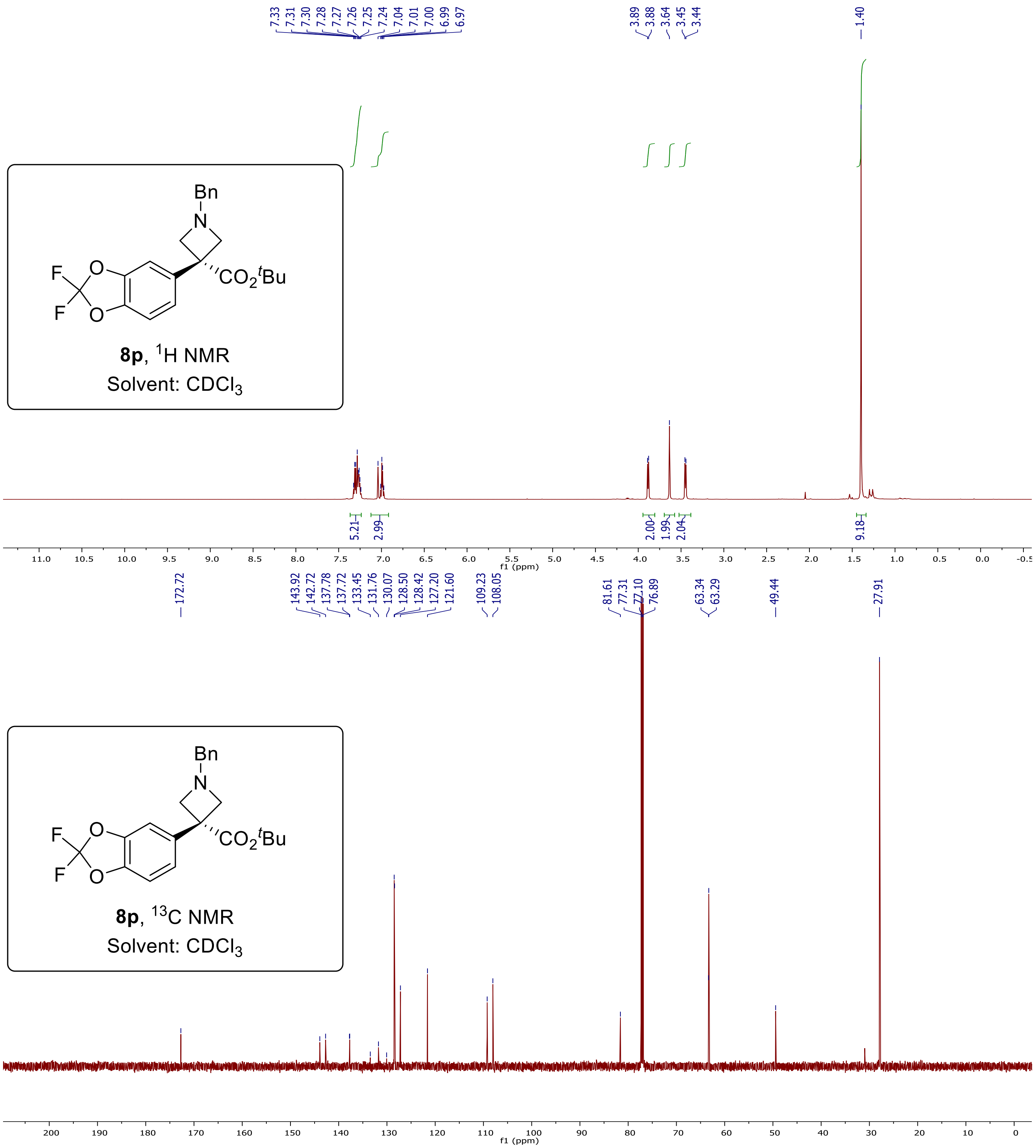

Supplementary Figure 110. NMR spectra of **8p**

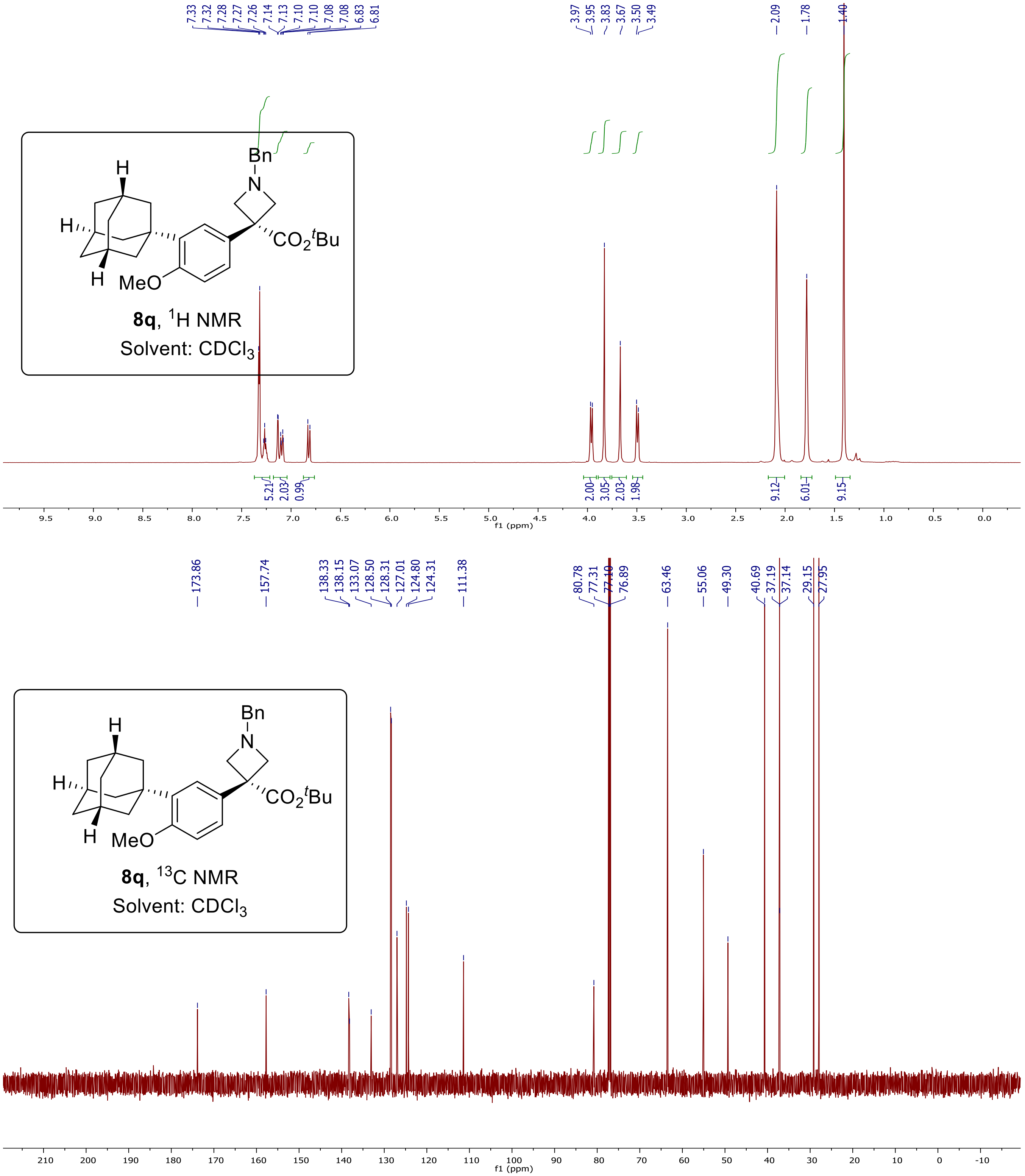

Supplementary Figure 111. NMR spectra of **8q**

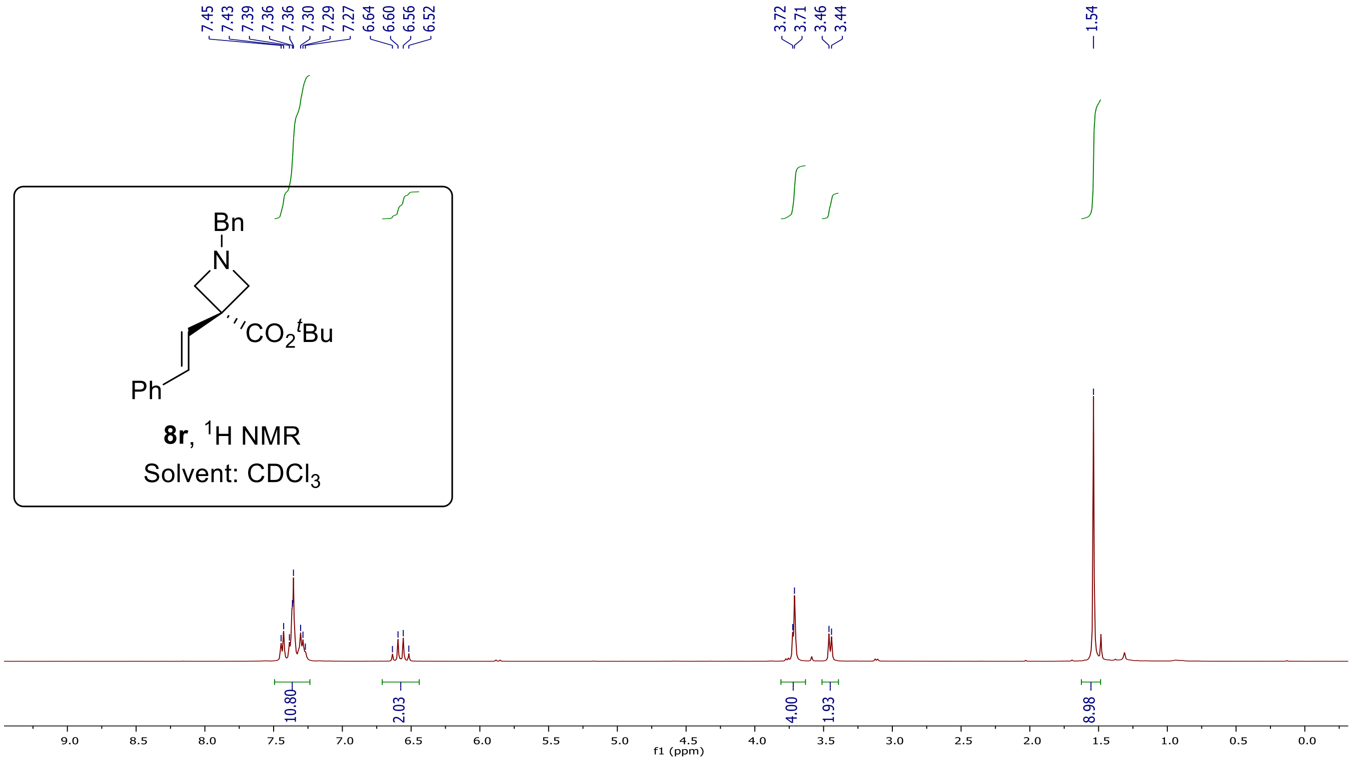

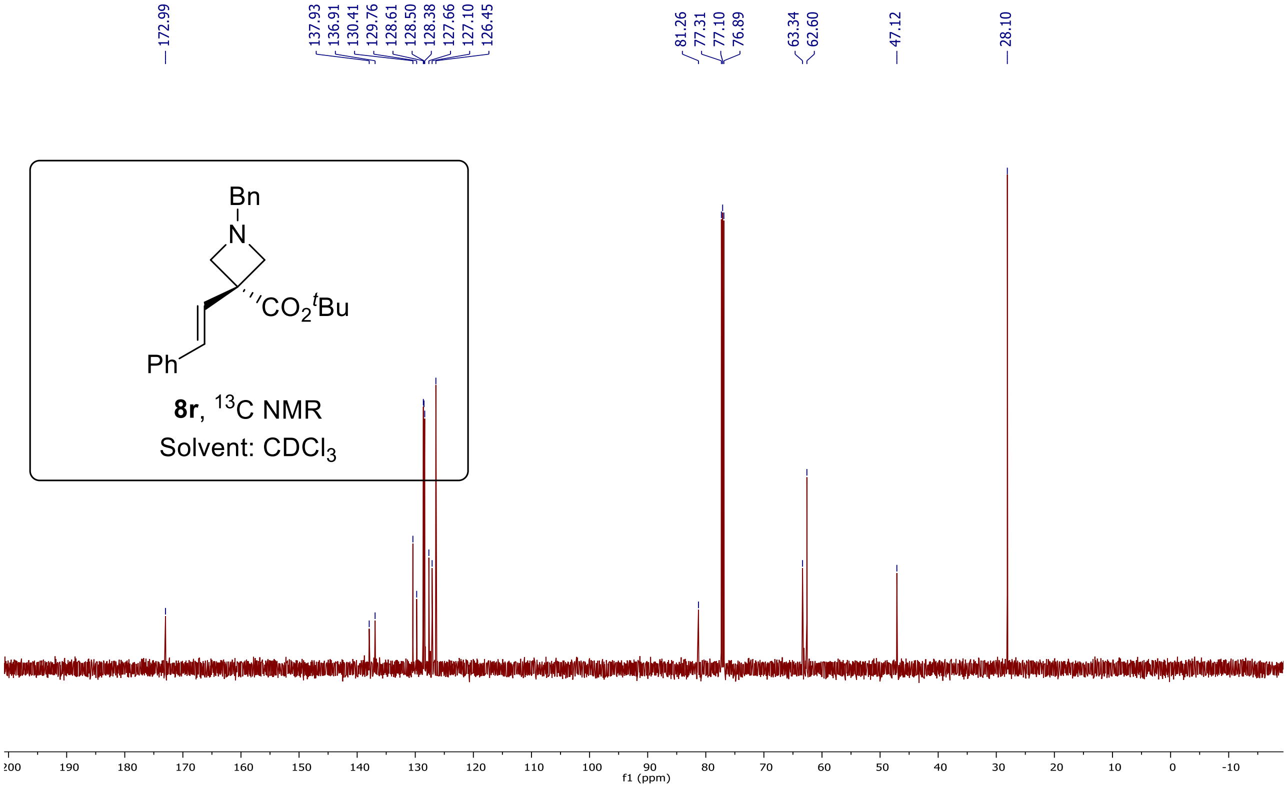

Supplementary Figure 112. NMR spectra of **8r**

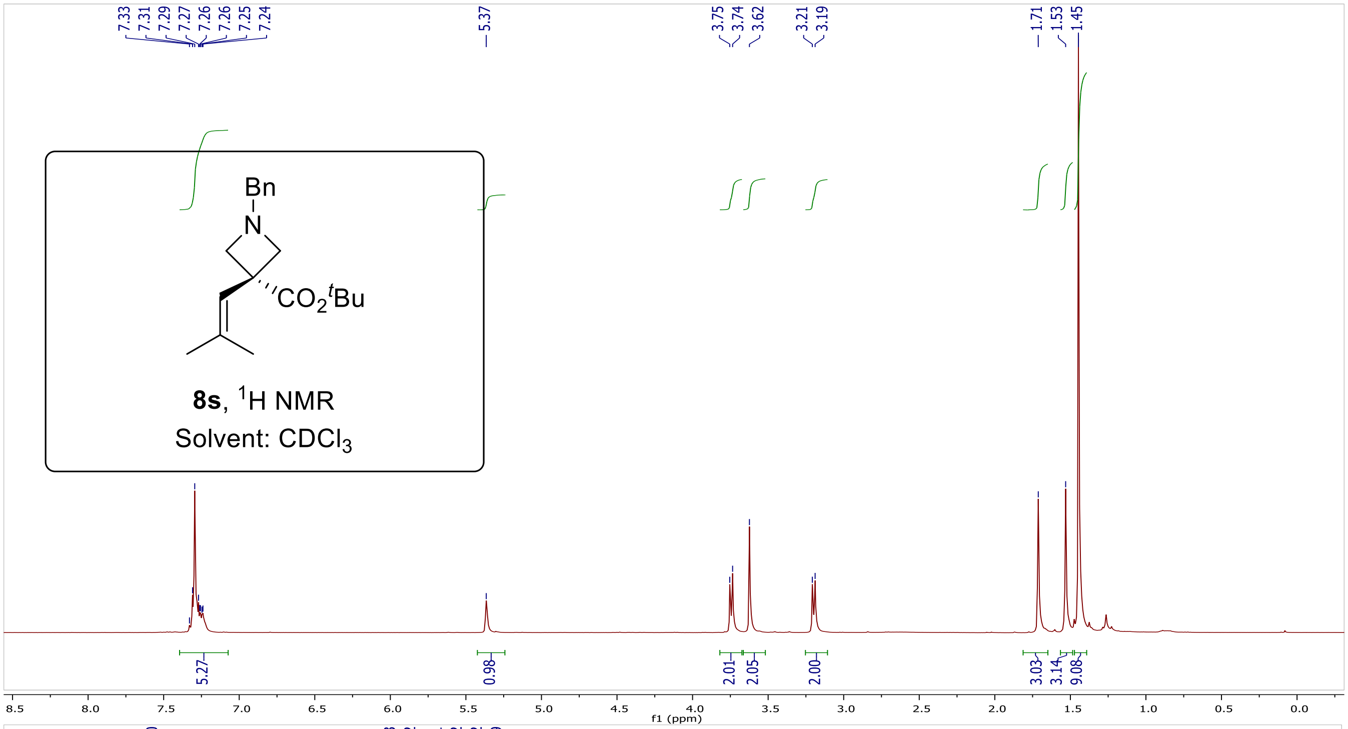

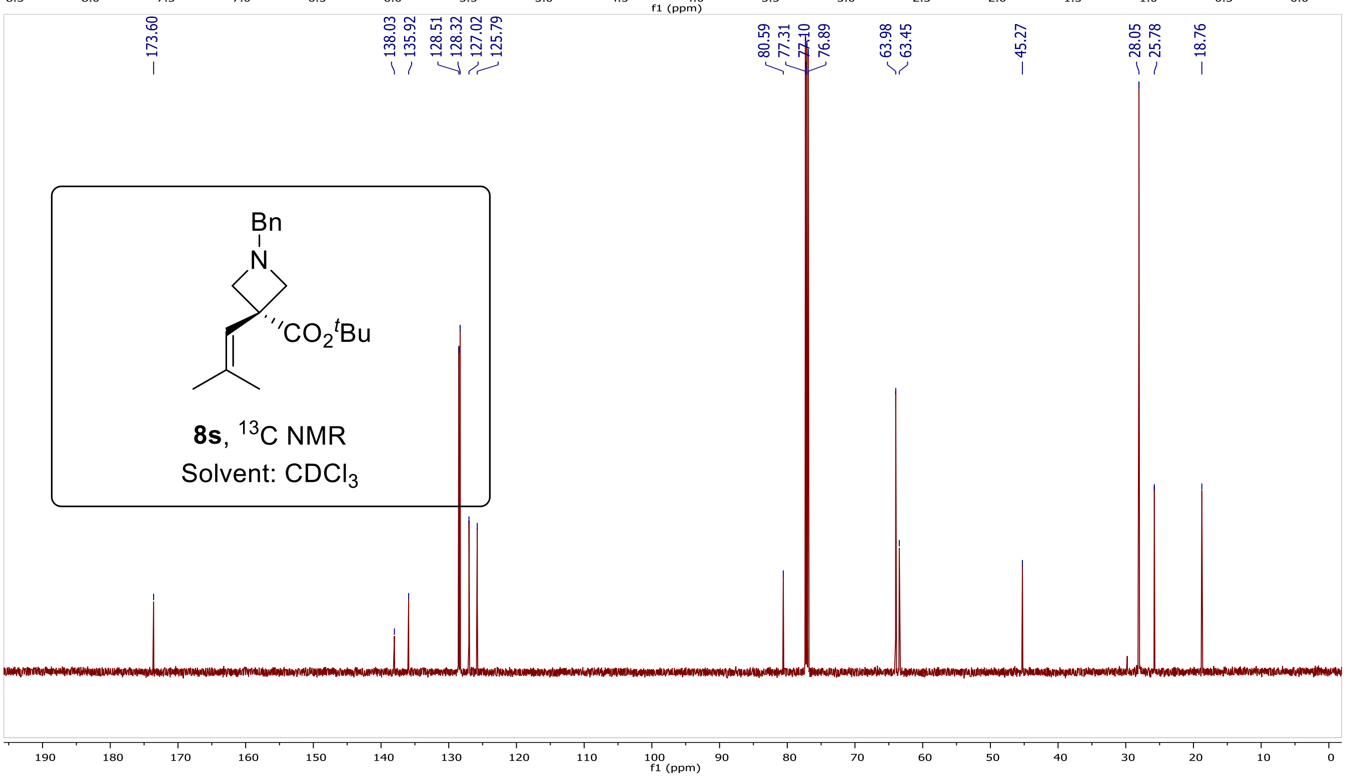

Supplementary Figure 113. NMR spectra of **8s**

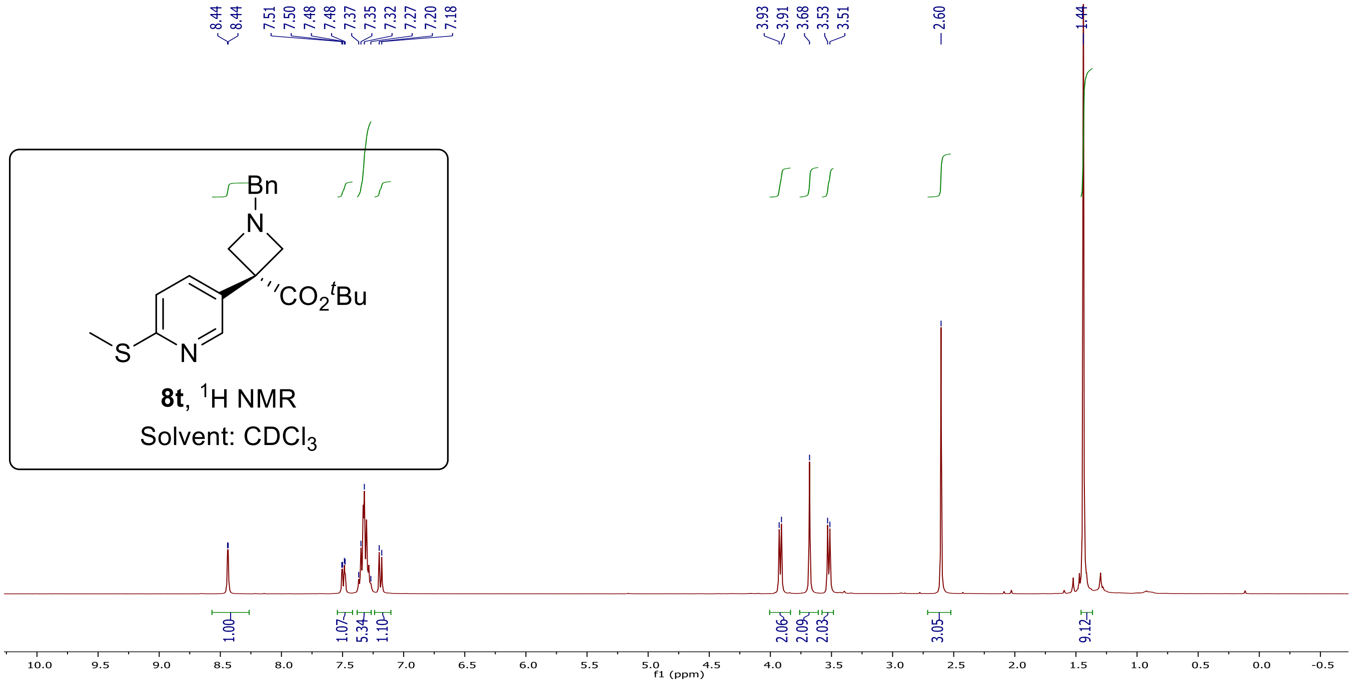

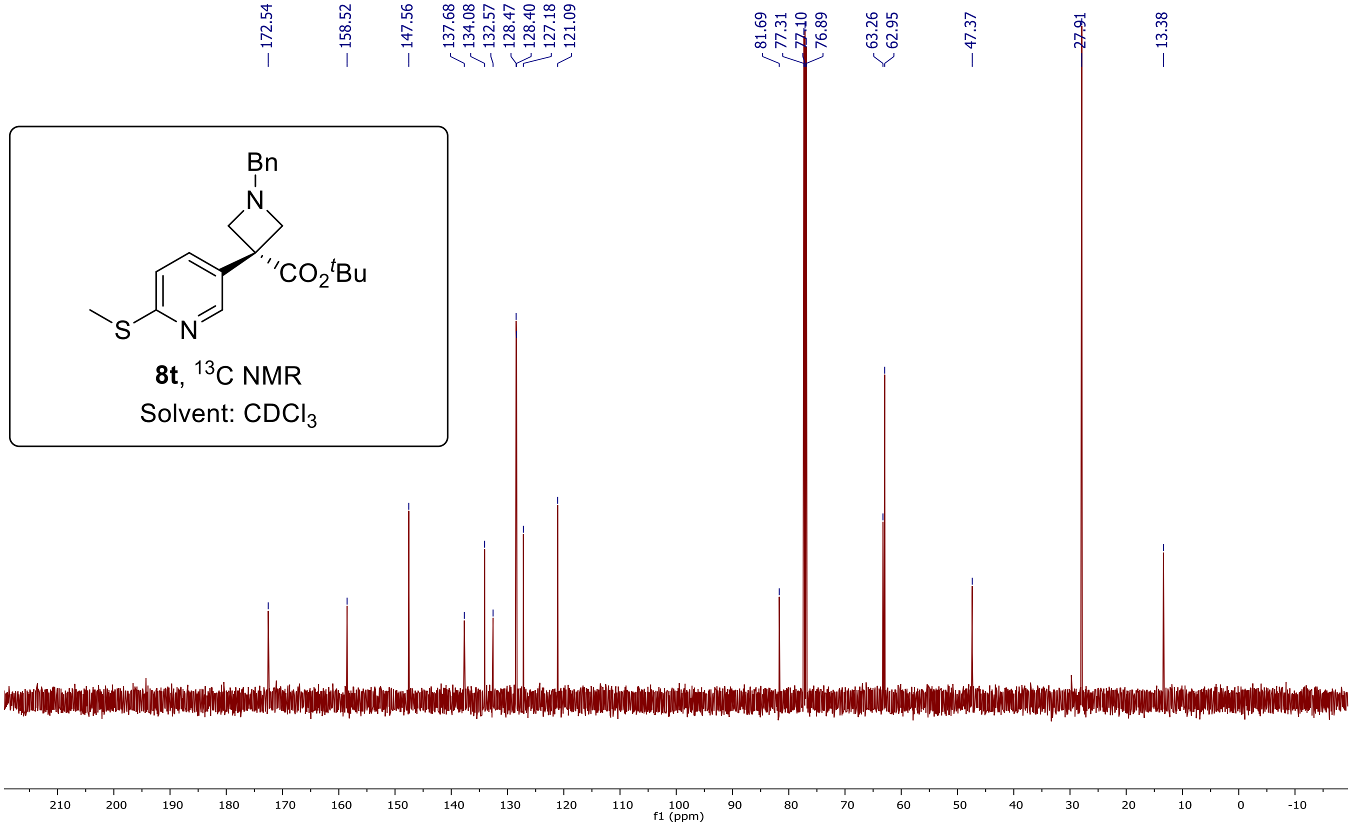

Supplementary Figure 114. NMR spectra of **8t**

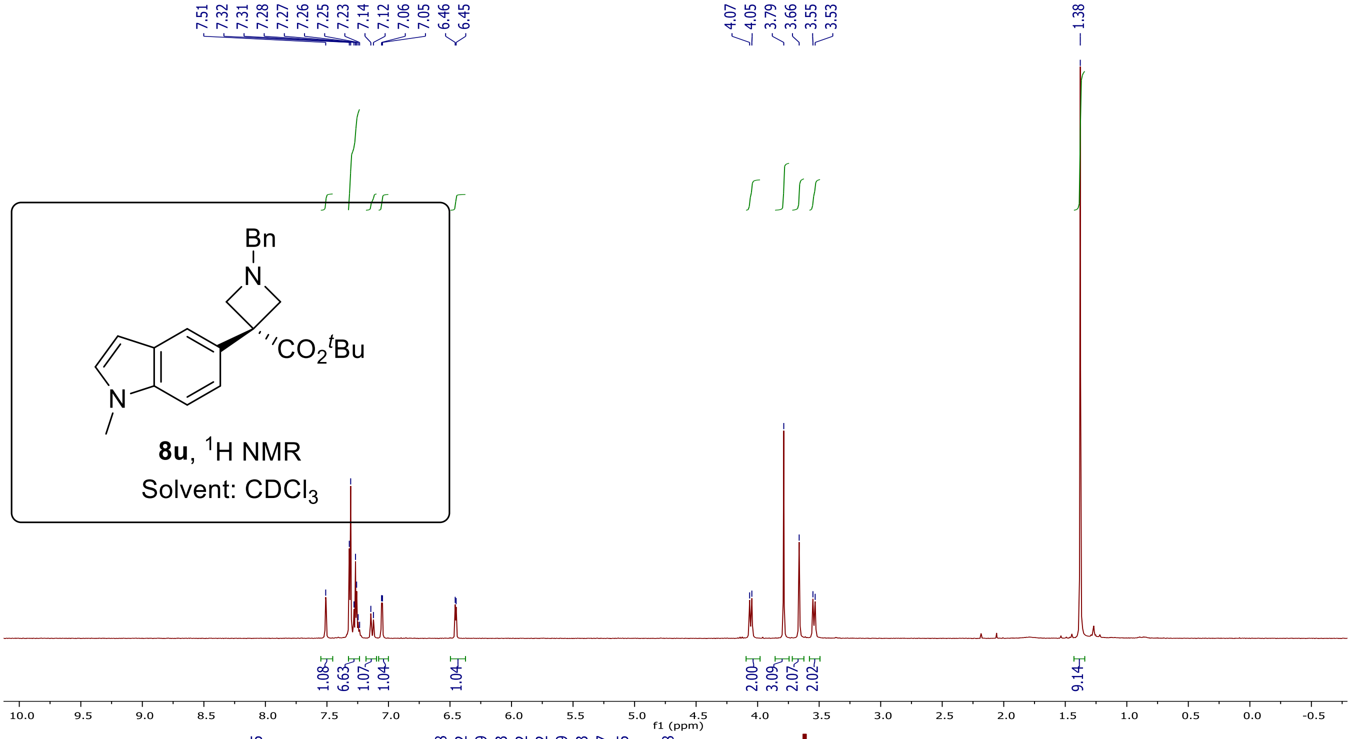

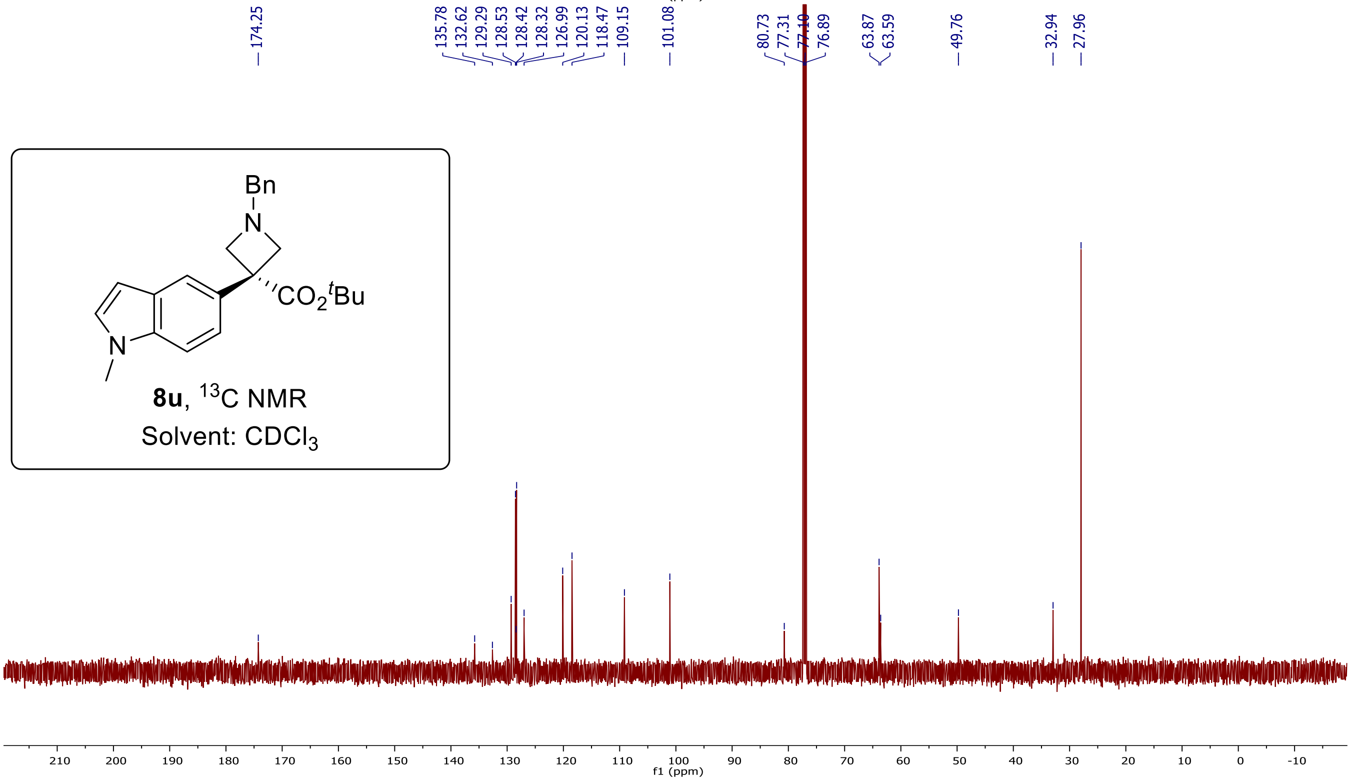

Supplementary Figure 115. NMR spectra of **8u**

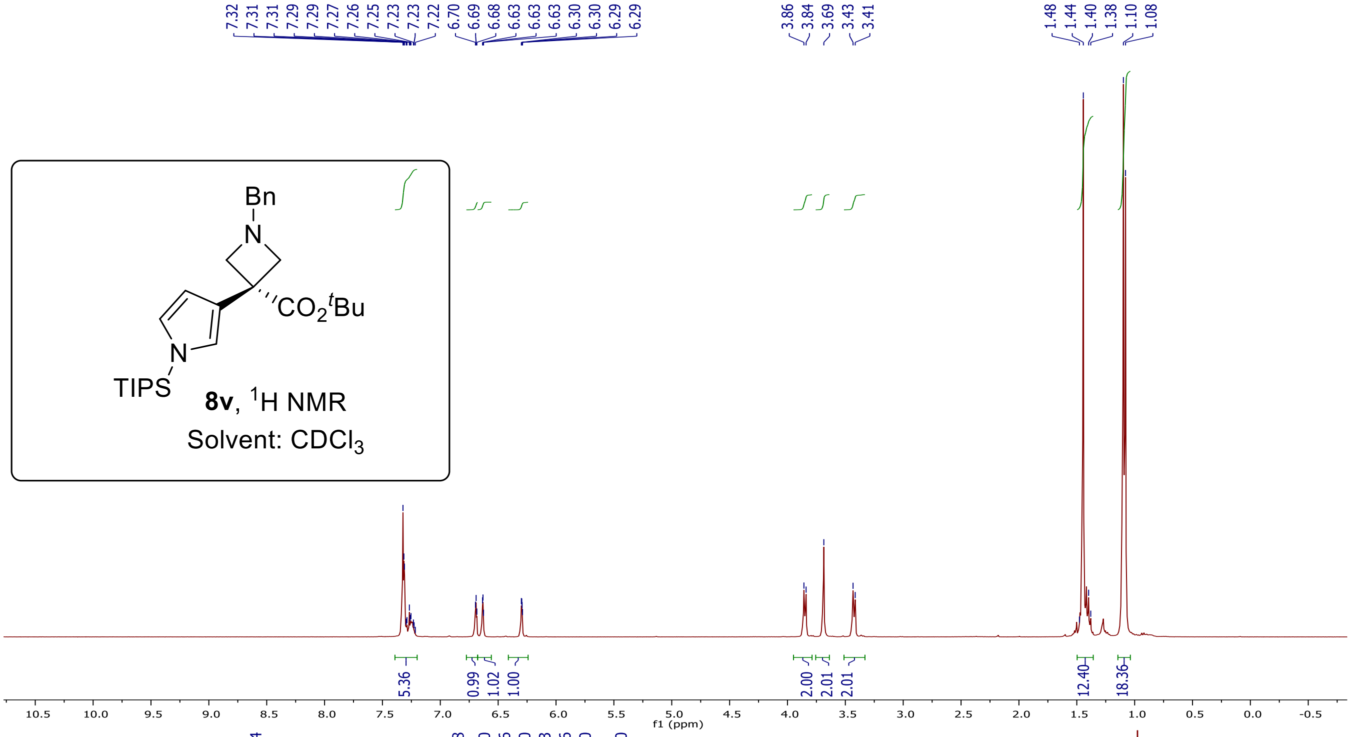

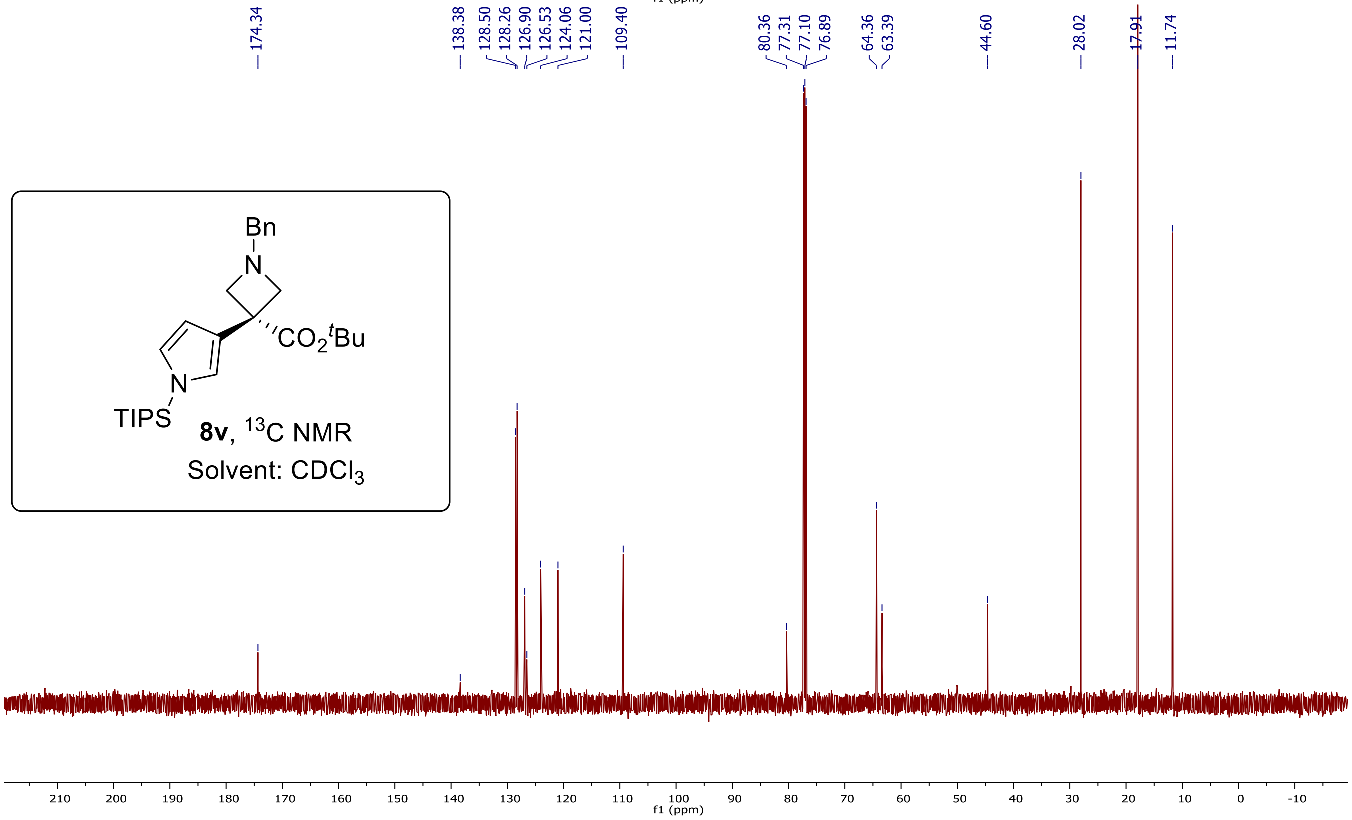

Supplementary Figure 116. NMR spectra of **8v**

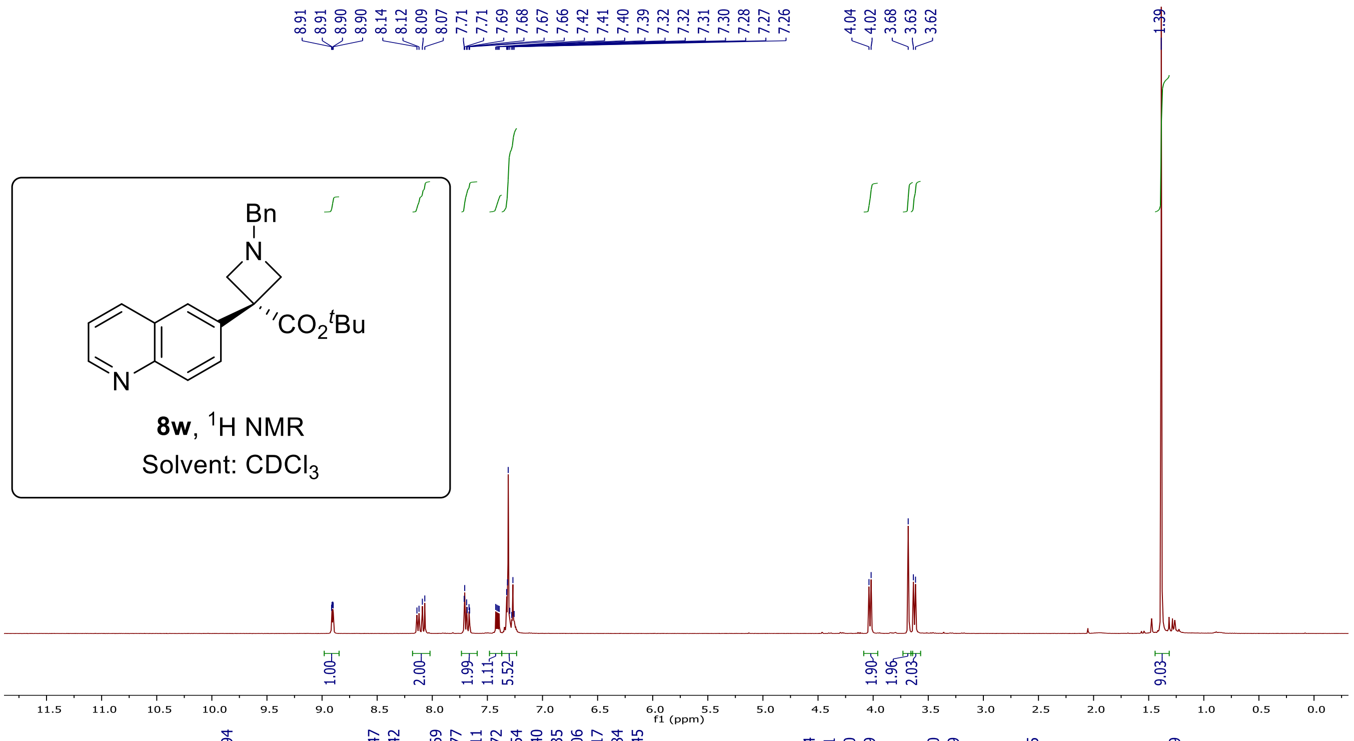

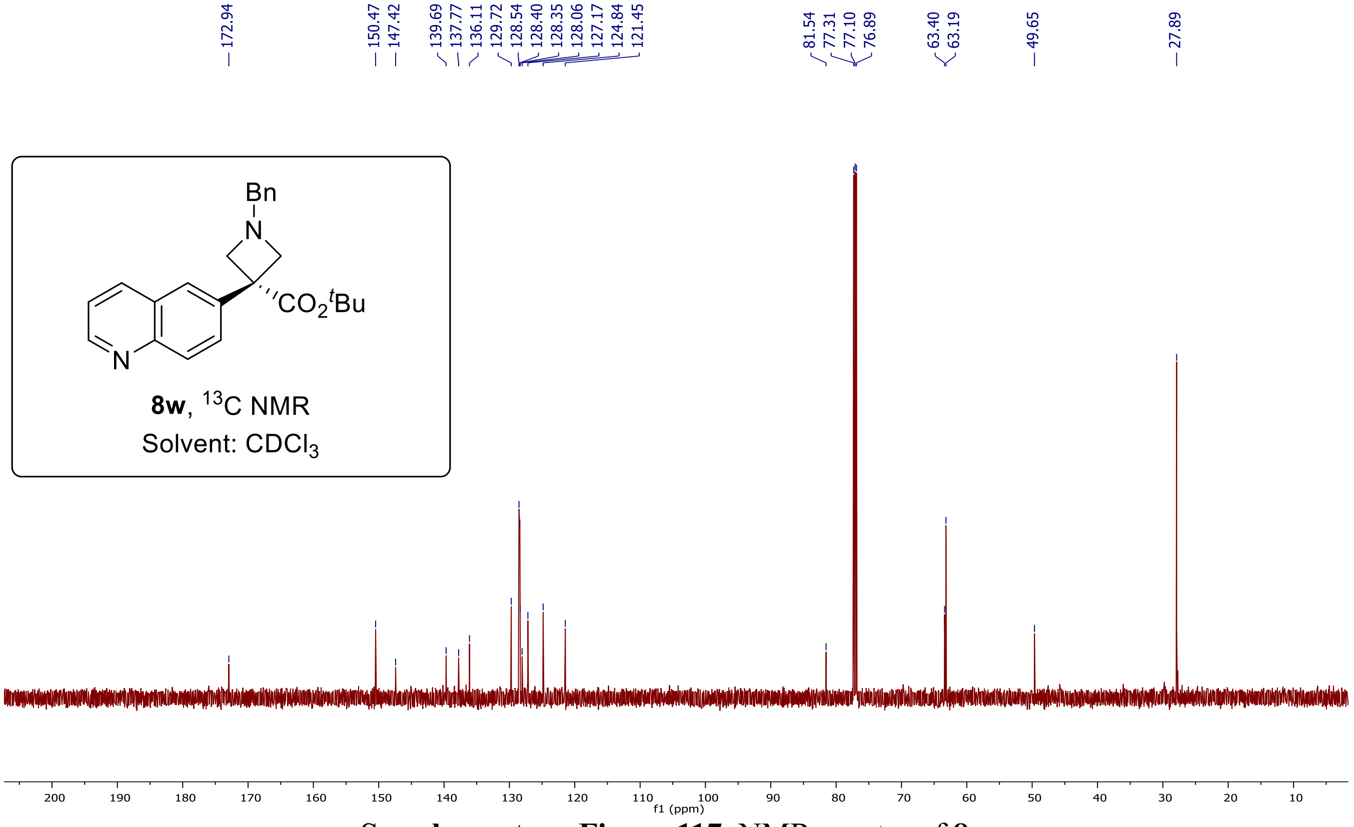

Supplementary Figure 117. NMR spectra of **8w**

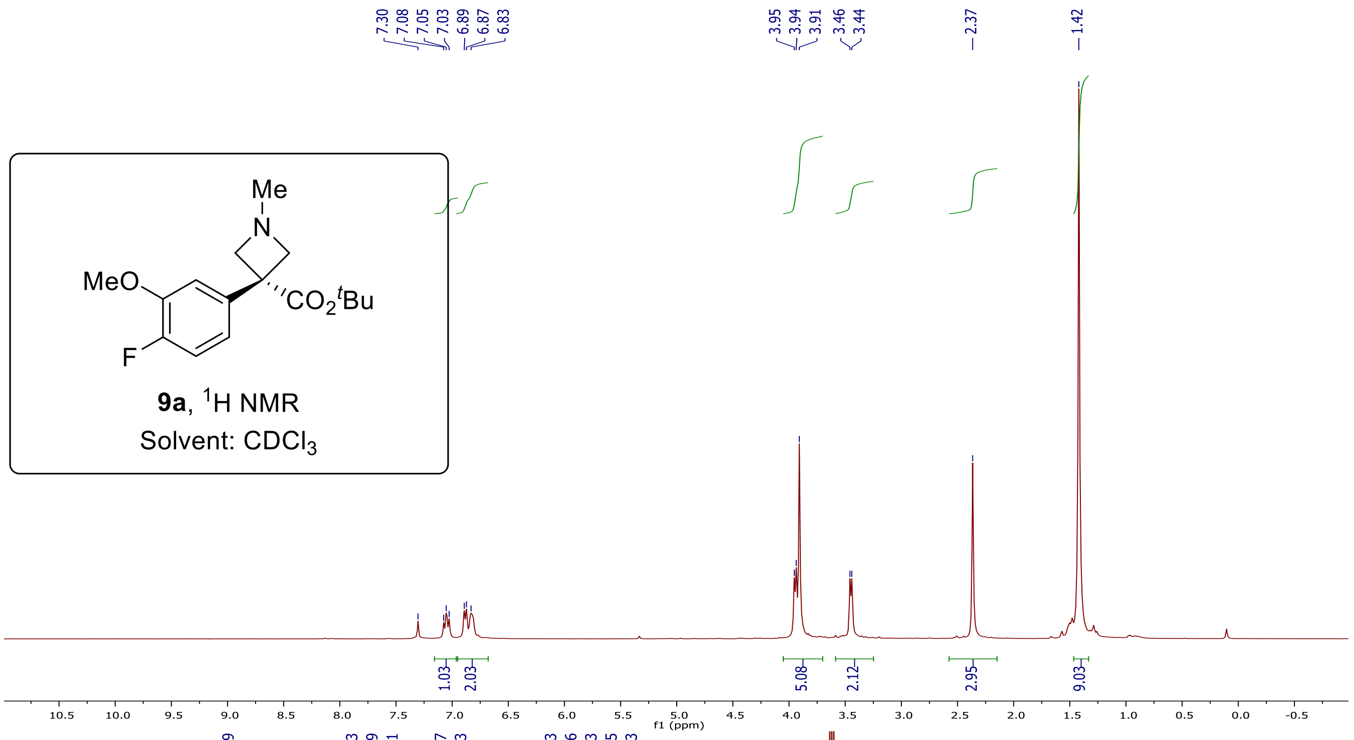

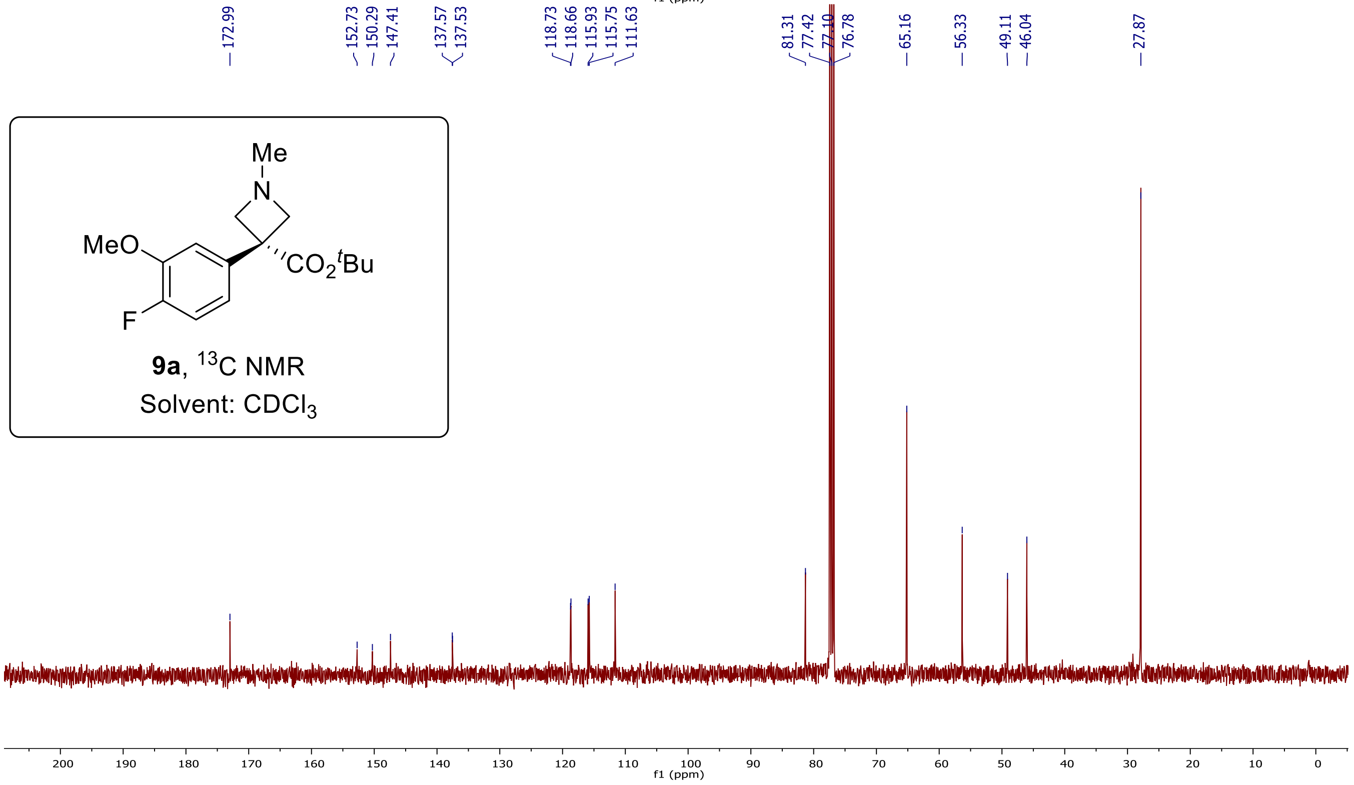

Supplementary Figure 118. NMR spectra of **9a**

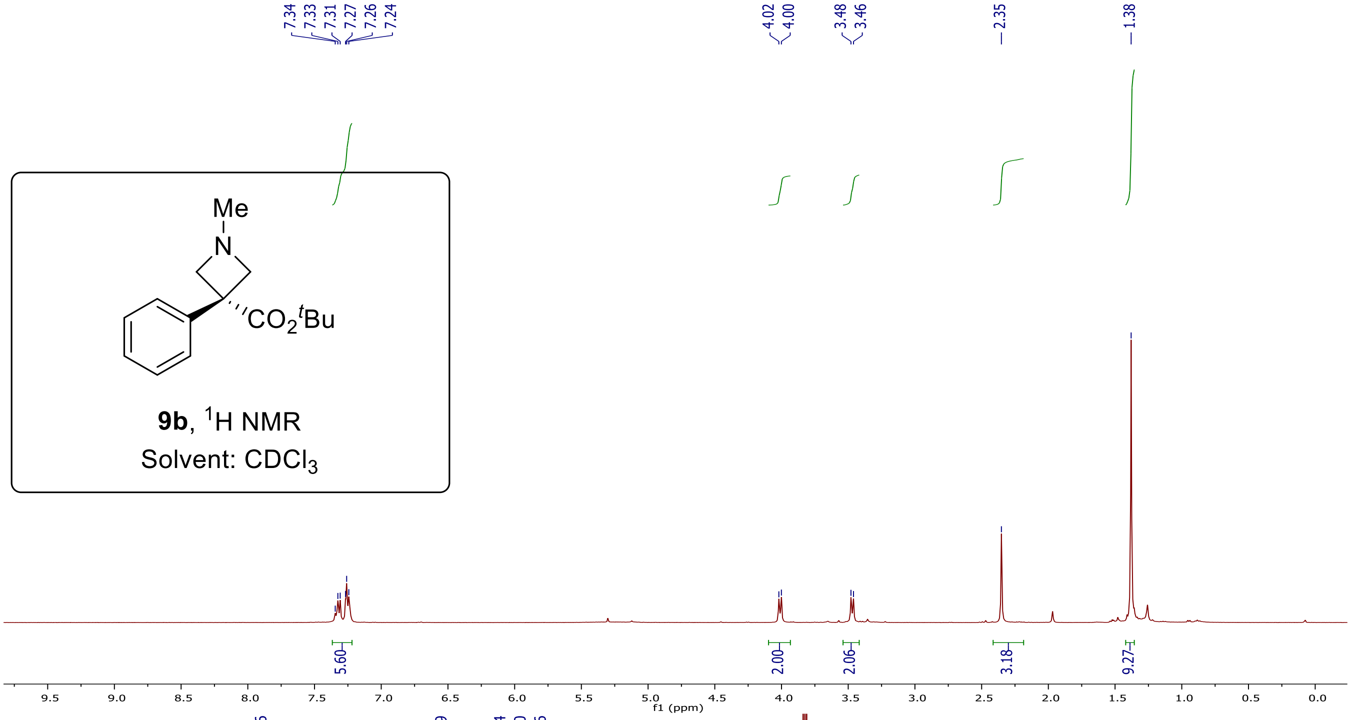

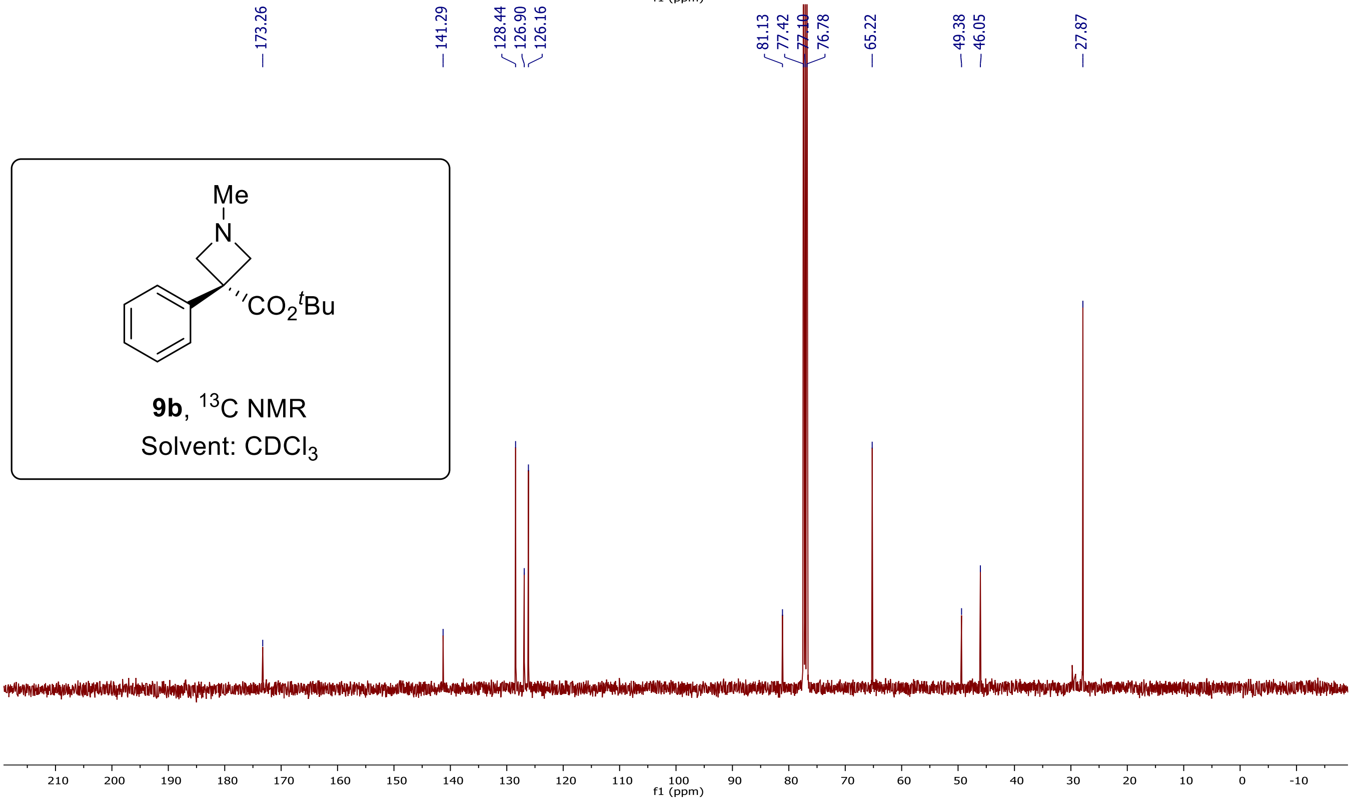

Supplementary Figure 119. NMR spectra of **9b**

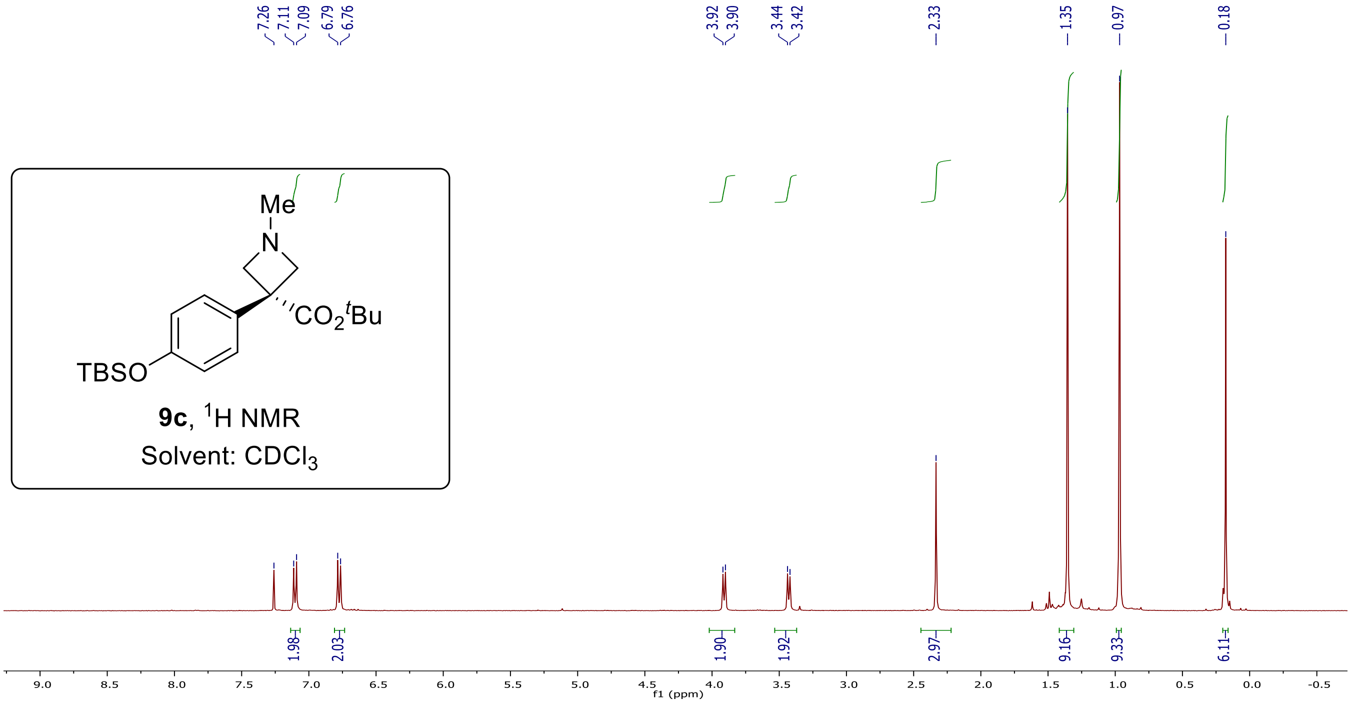

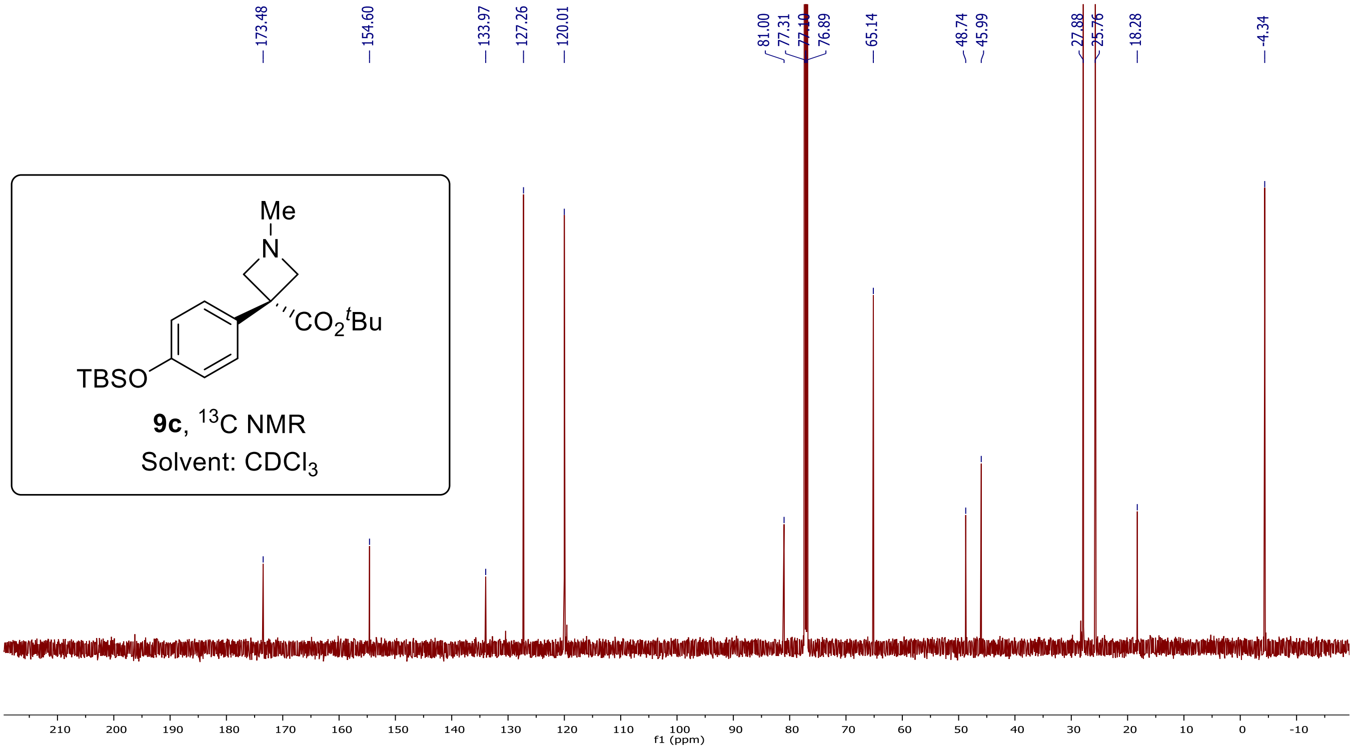

Supplementary Figure 120. NMR spectra of **9c**

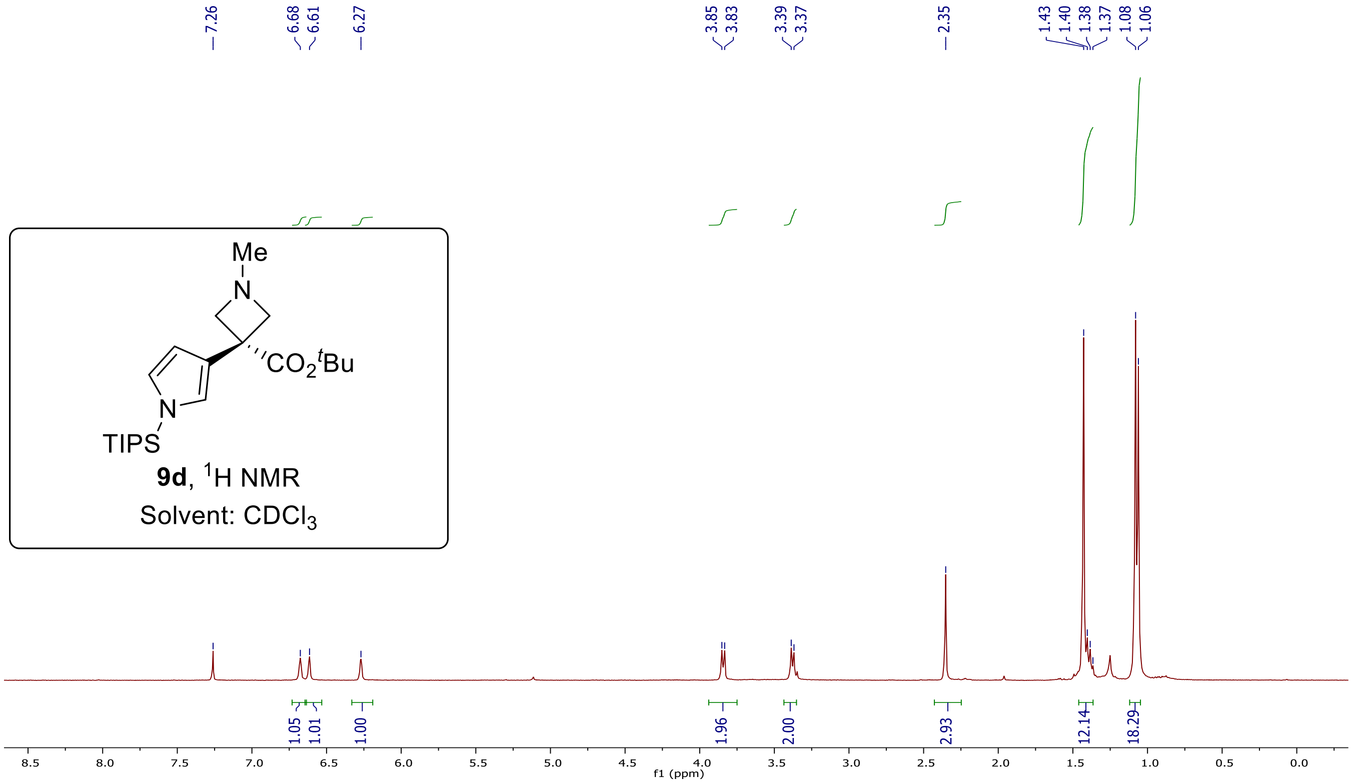

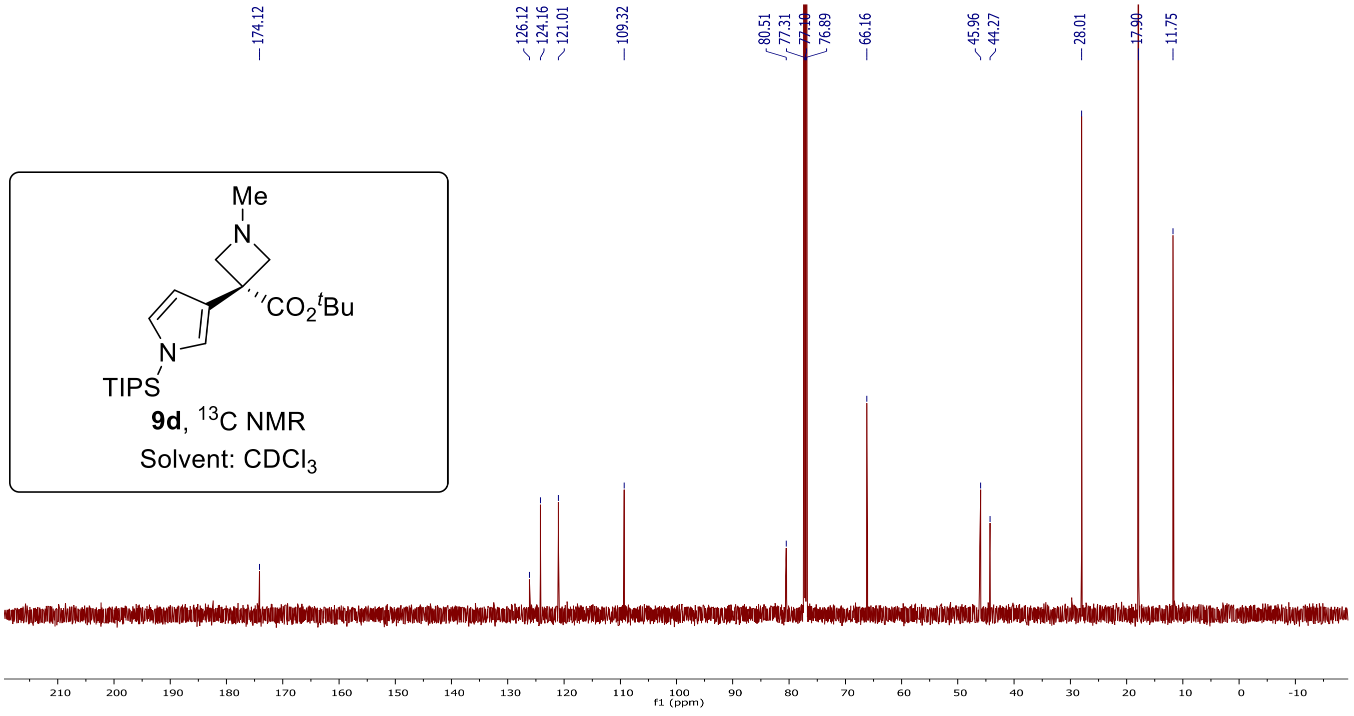

Supplementary Figure 121. NMR spectra of **9d**

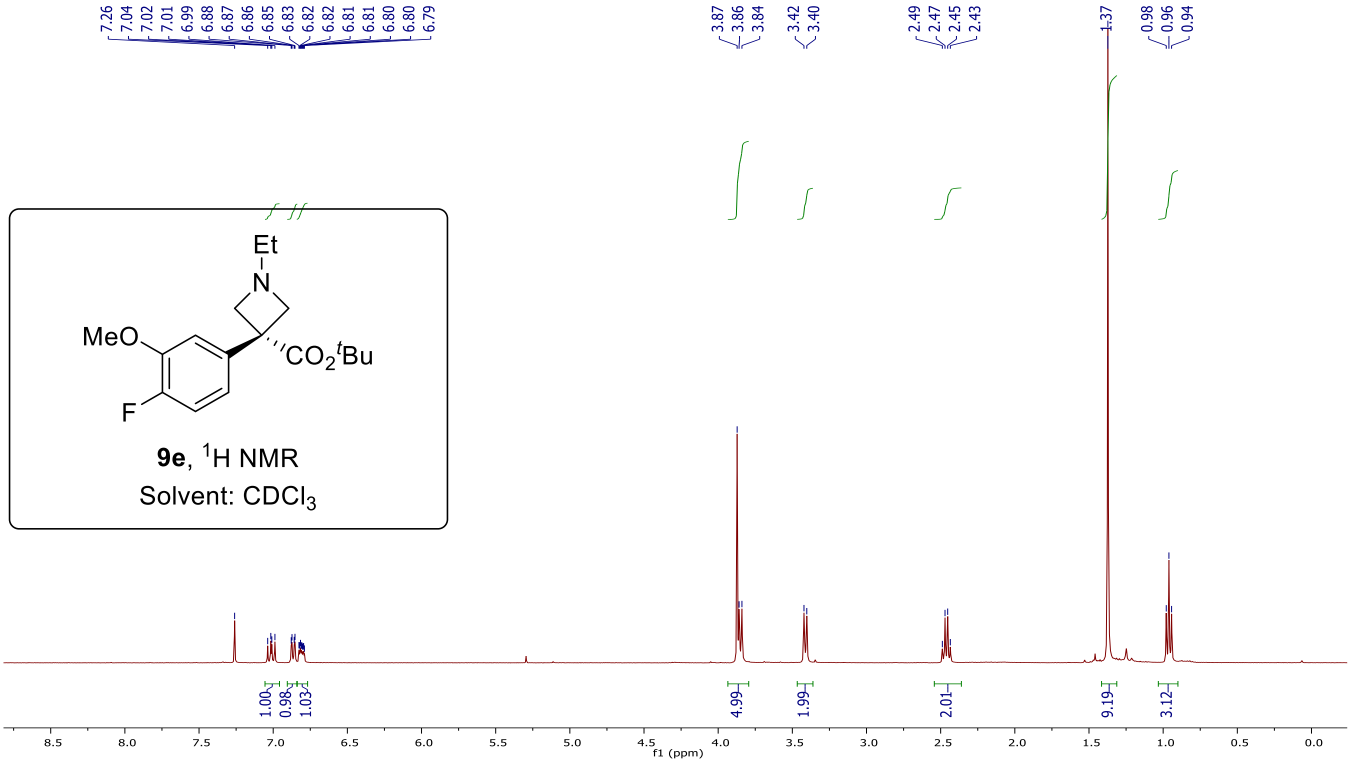

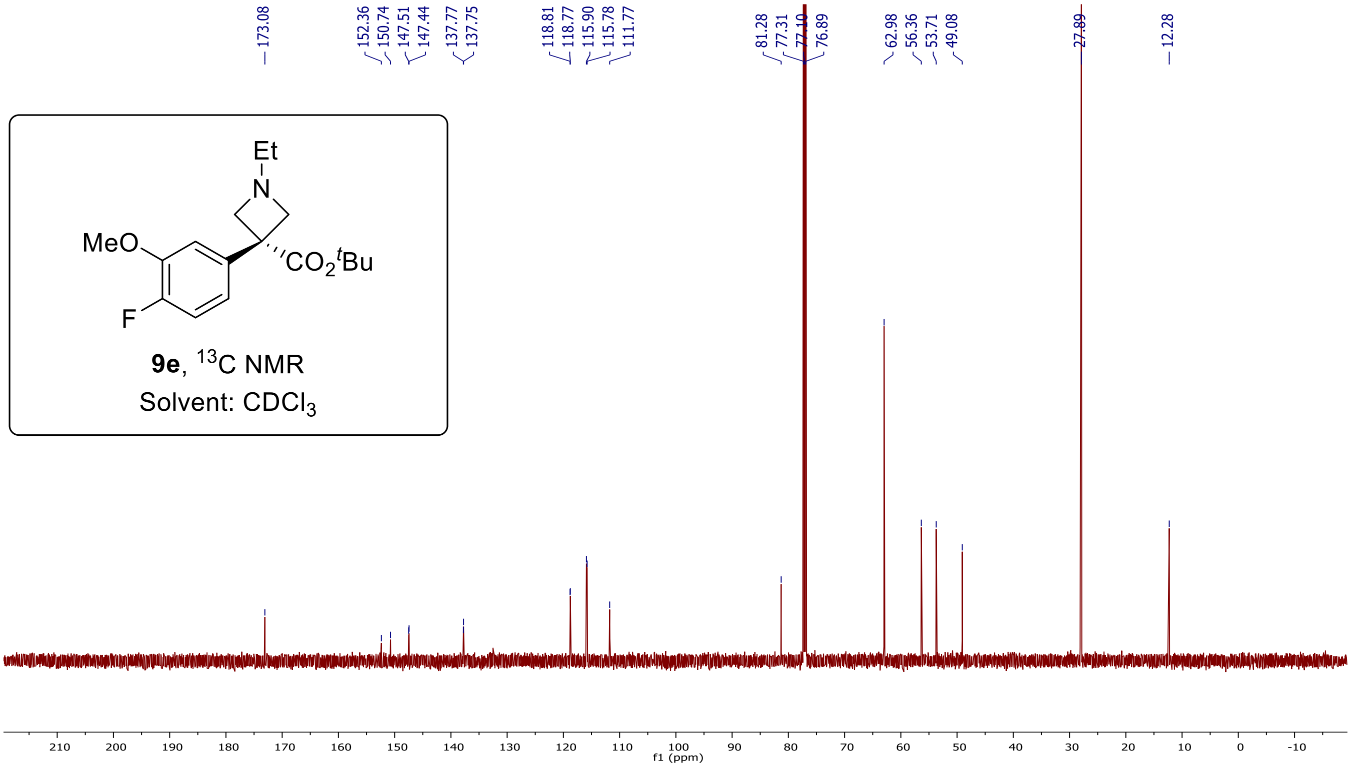

Supplementary Figure 122. NMR spectra of **9e**

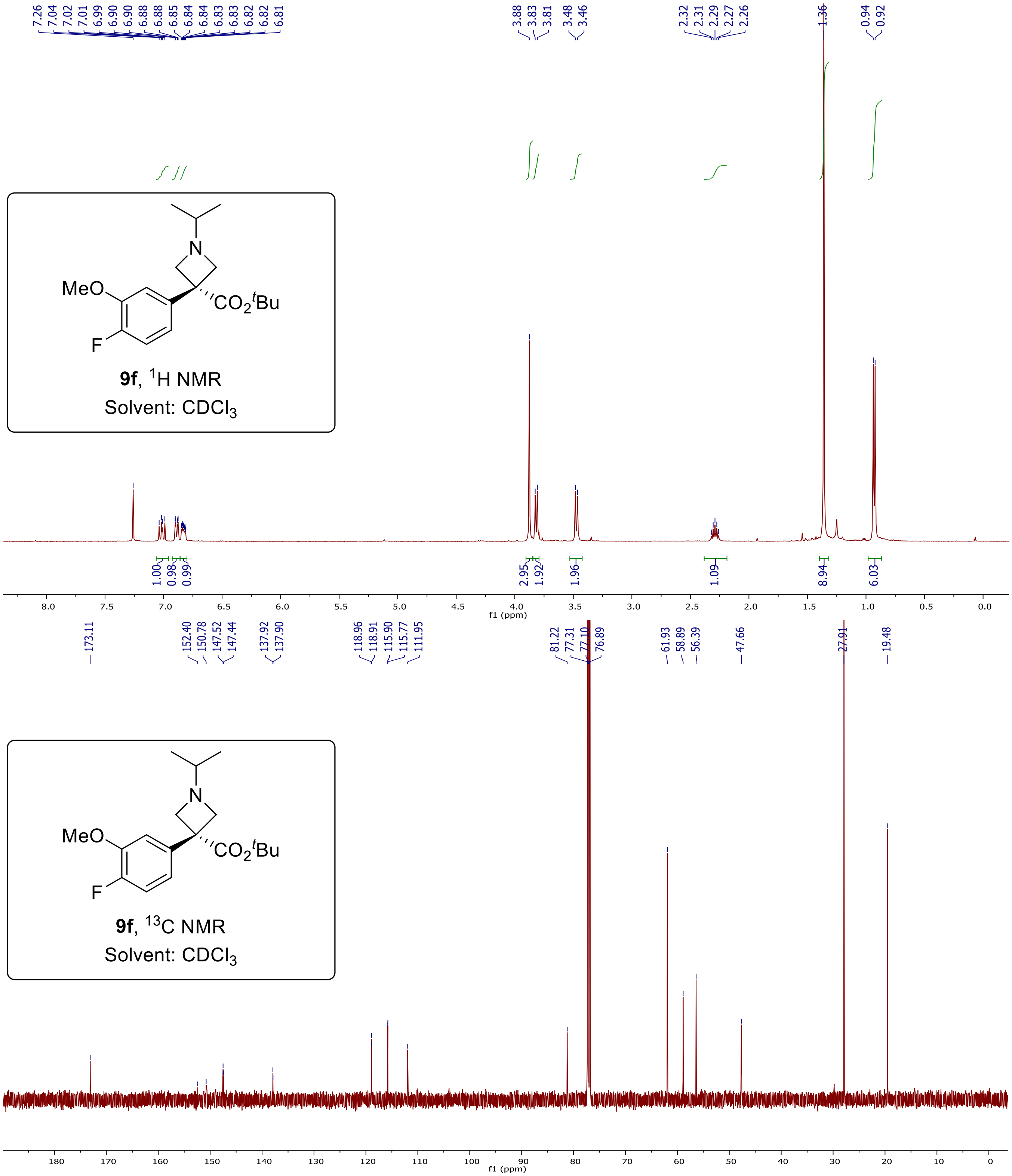

Supplementary Figure 123. NMR spectra of **9f**

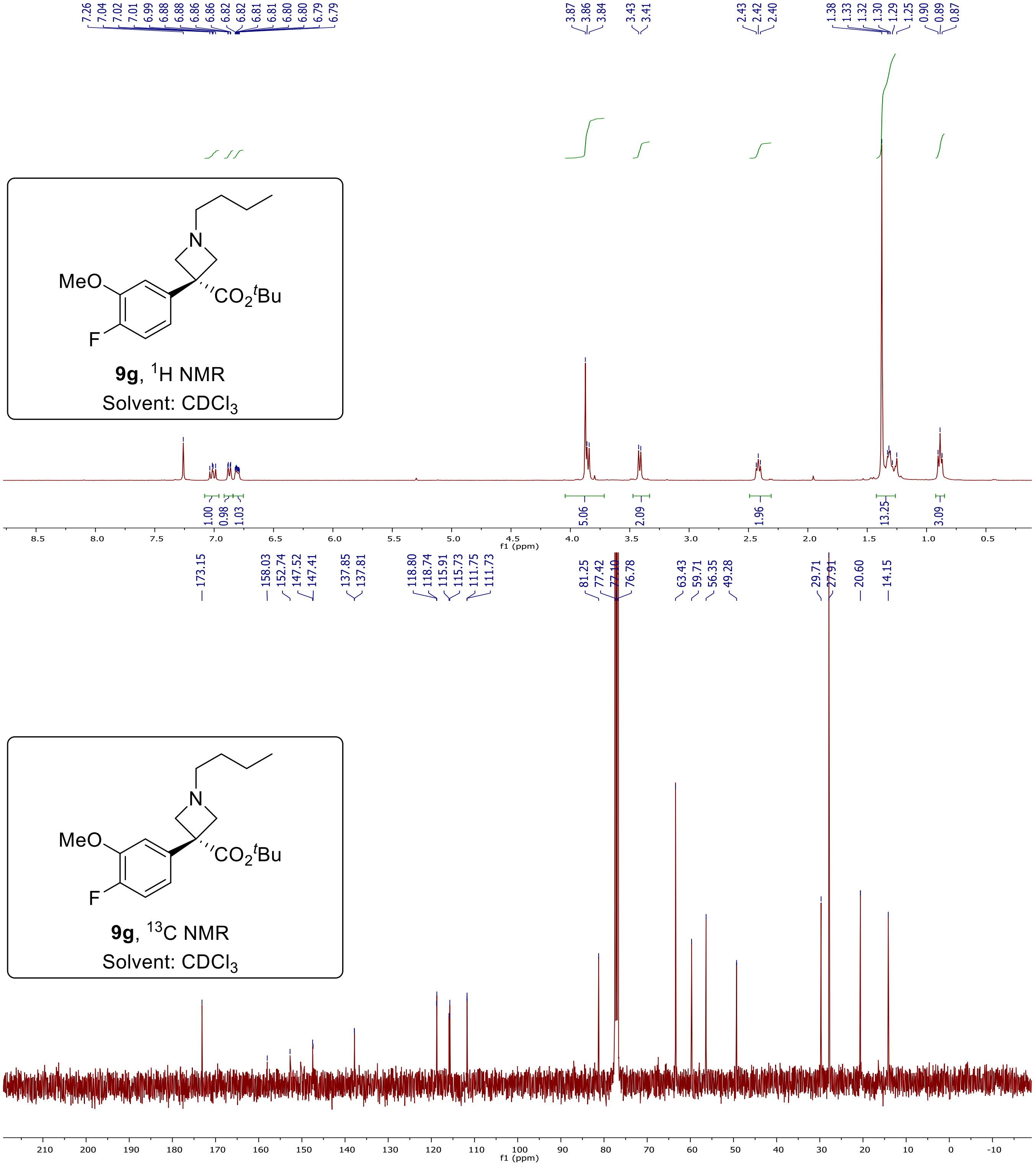

Supplementary Figure 124. NMR spectra of **9g**

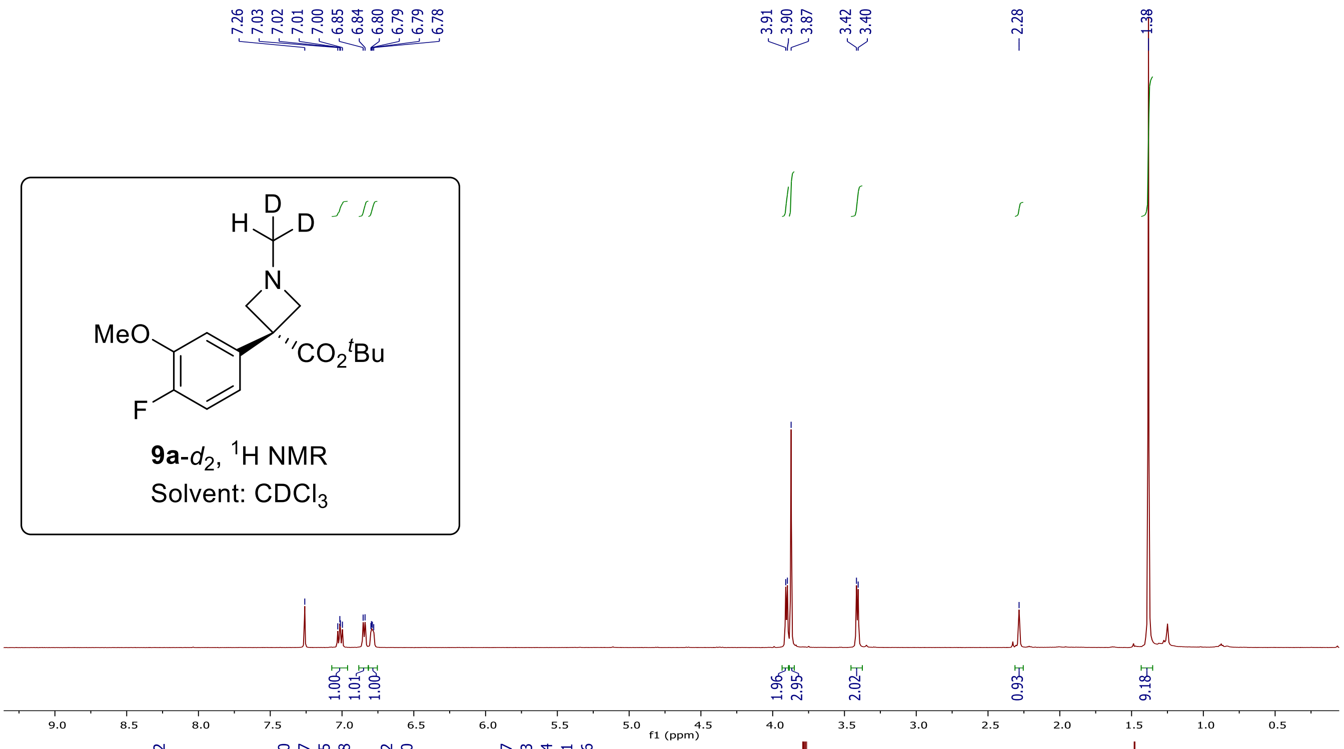

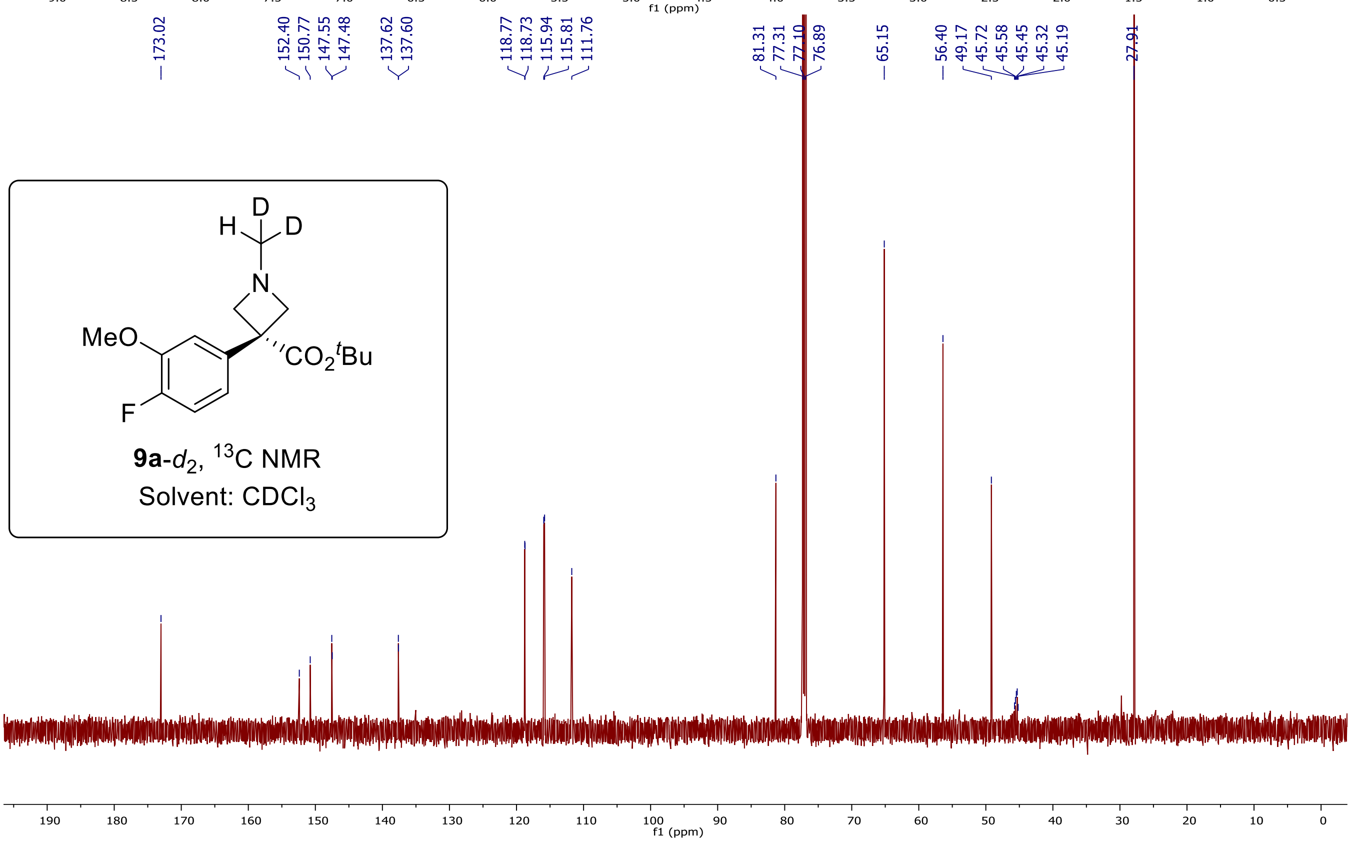

Supplementary Figure 125. NMR spectra of **9a-***d*$_2$

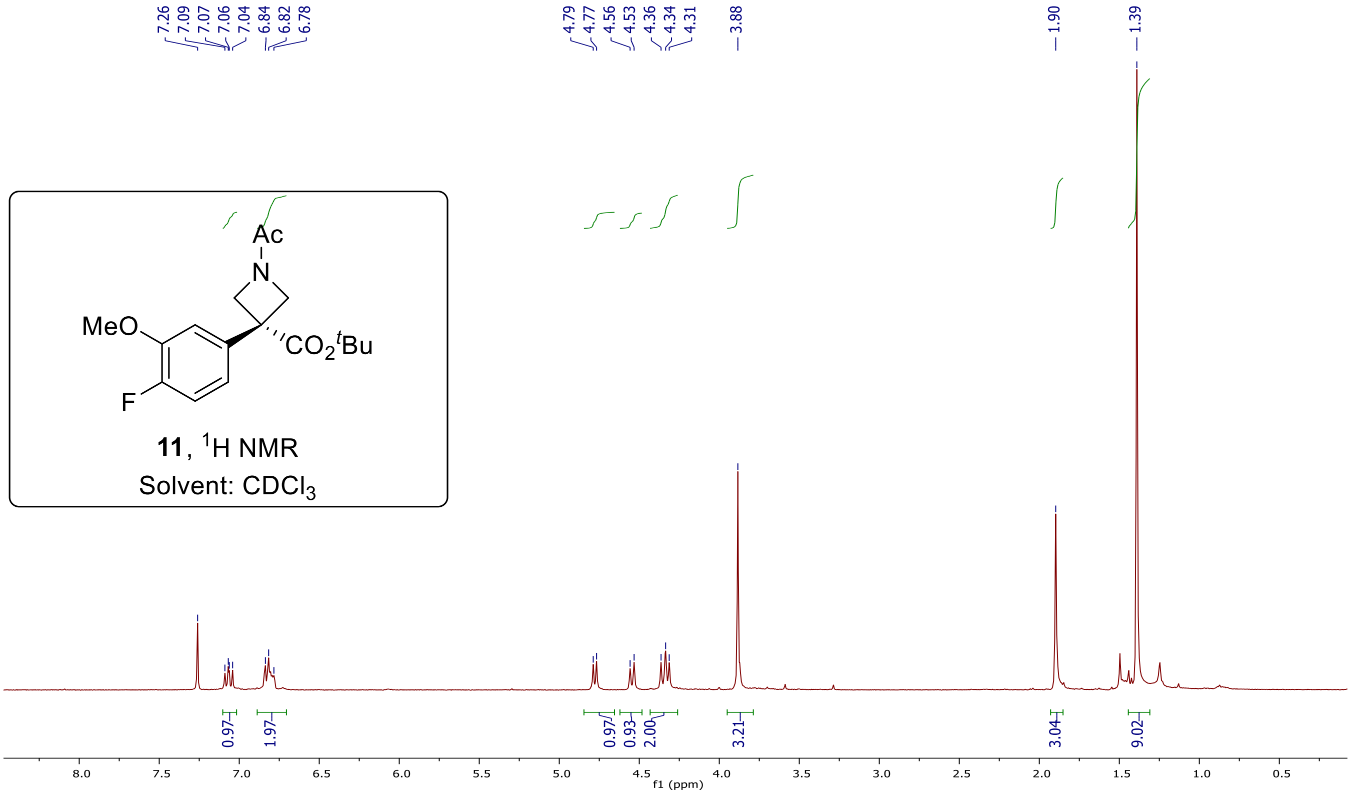

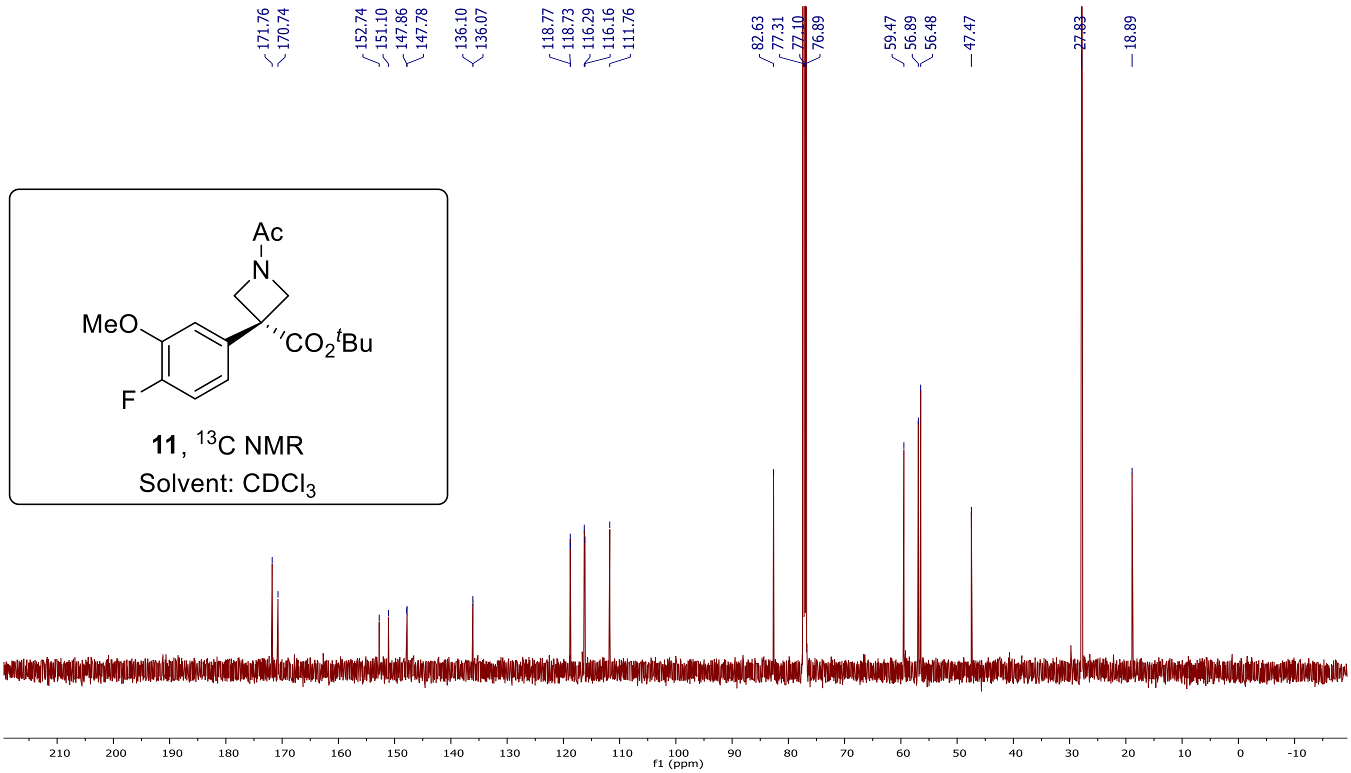

Supplementary Figure 126. NMR spectra of **11**

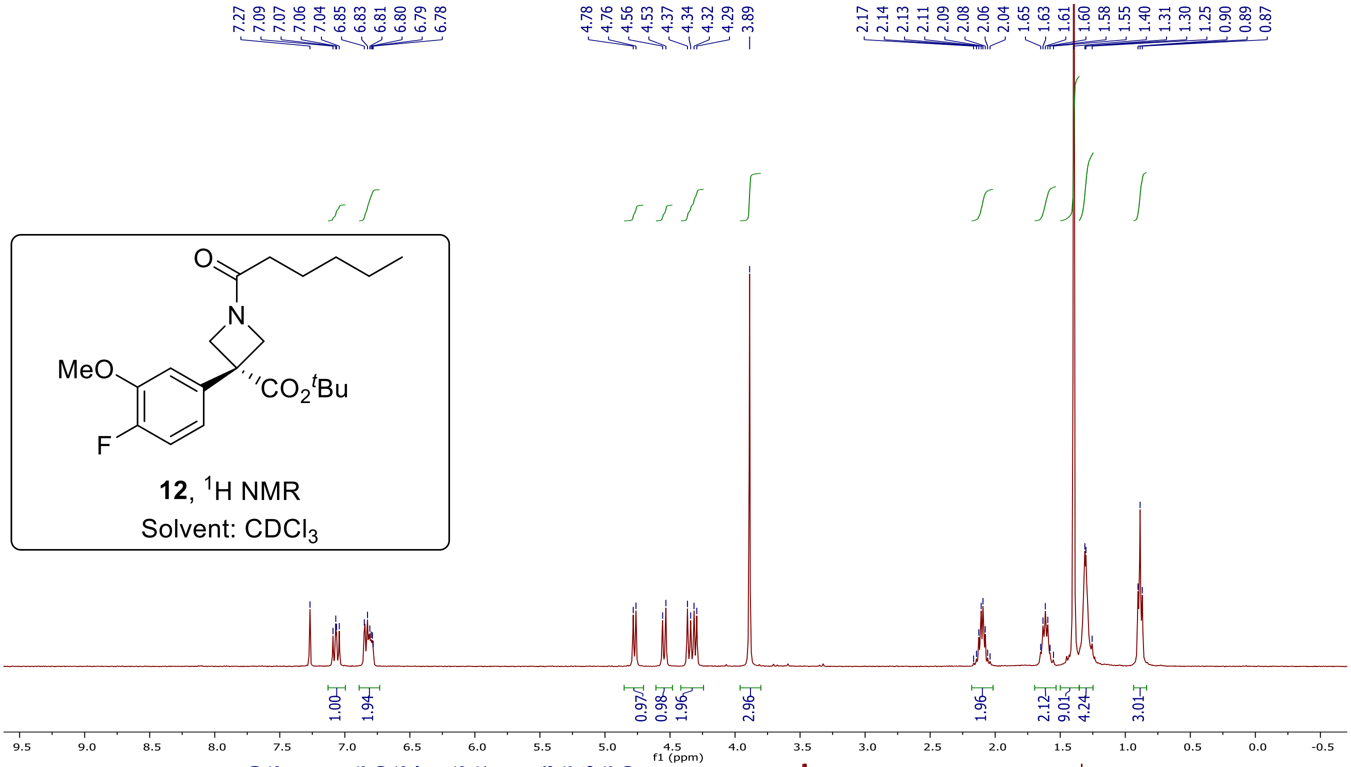

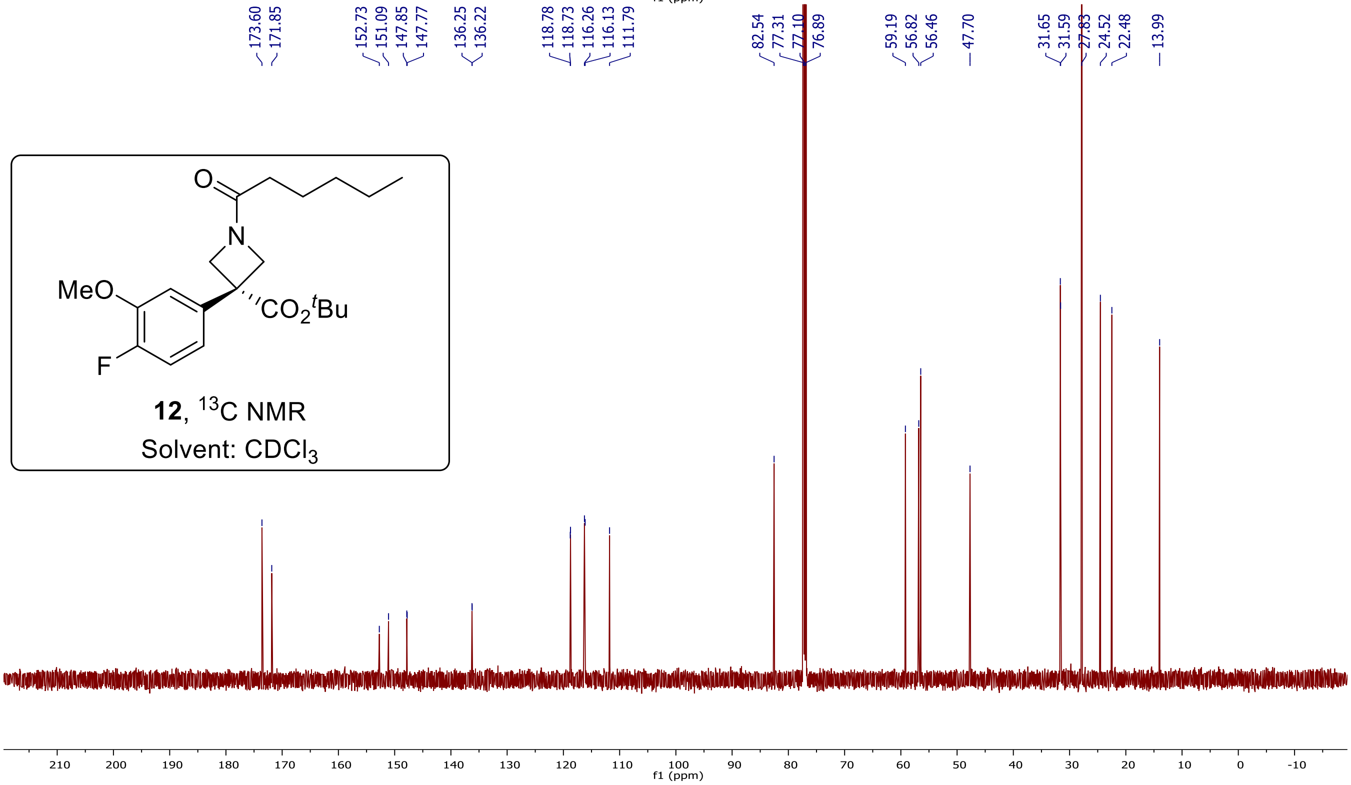

Supplementary Figure 127. NMR spectra of **12**

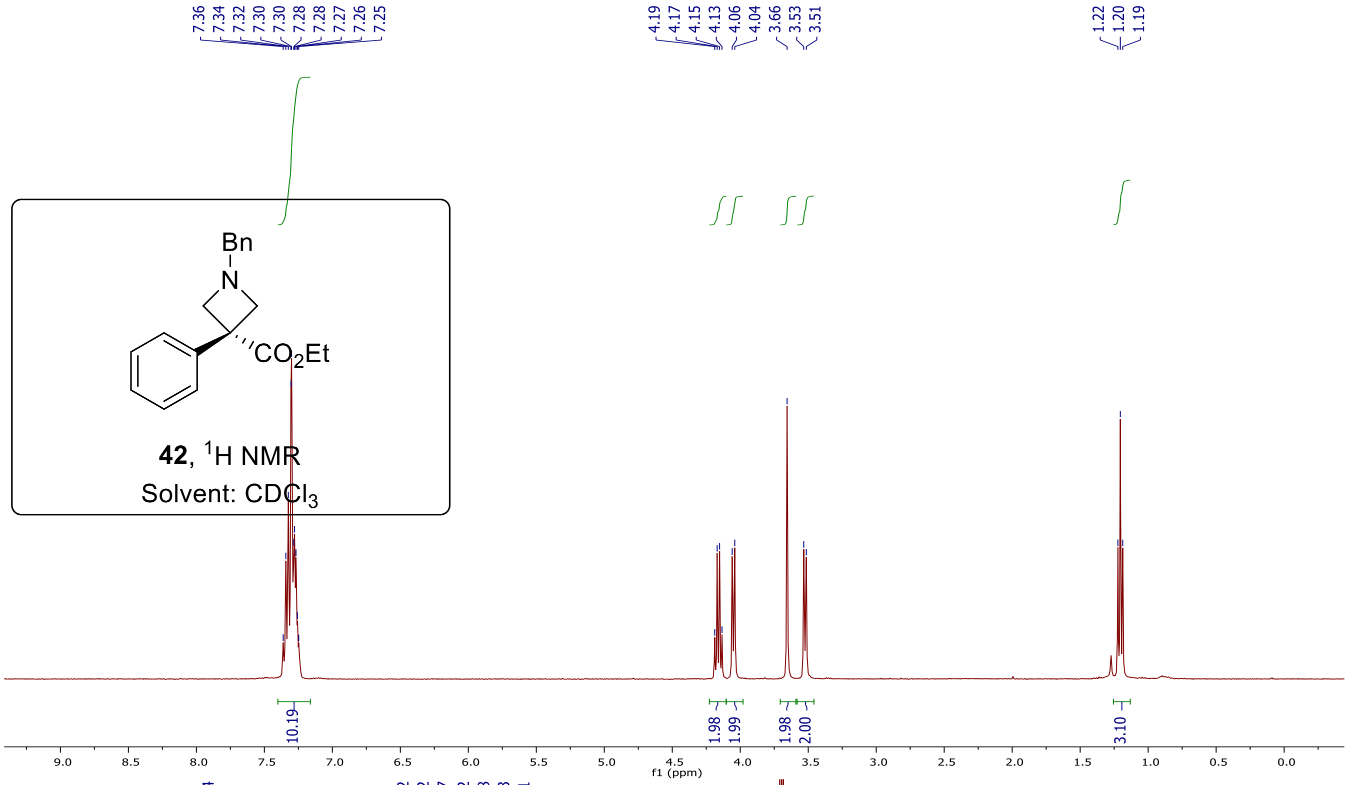

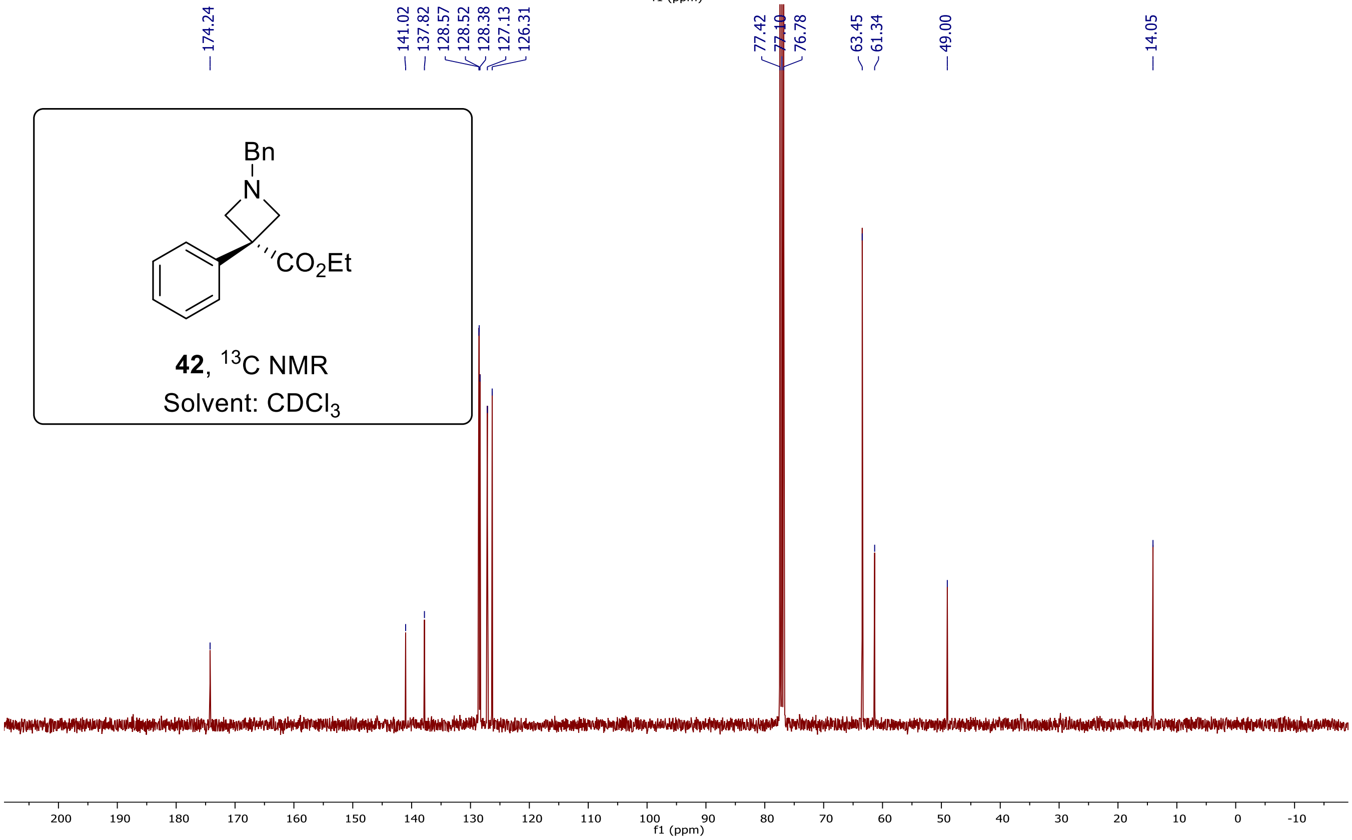

Supplementary Figure 128. NMR spectra of **42**

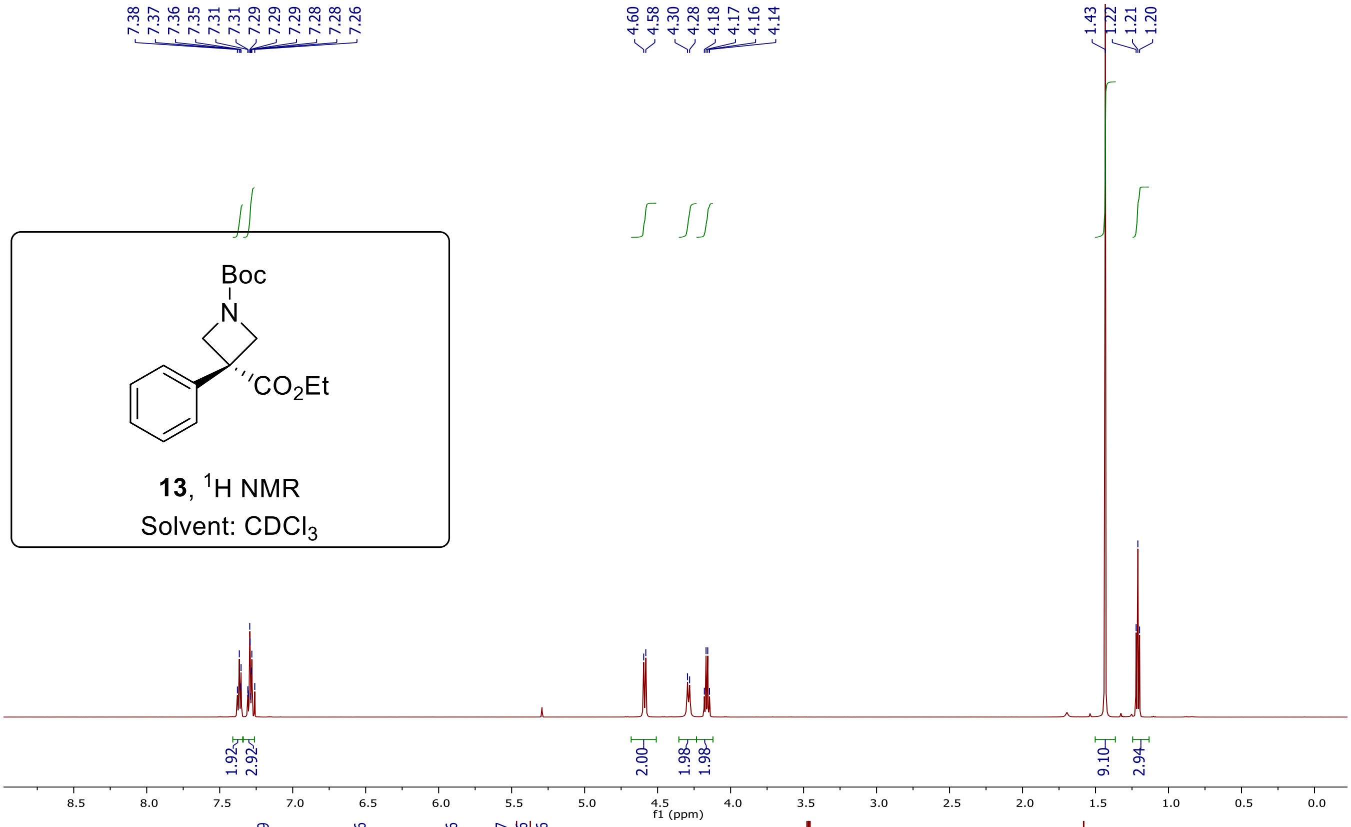

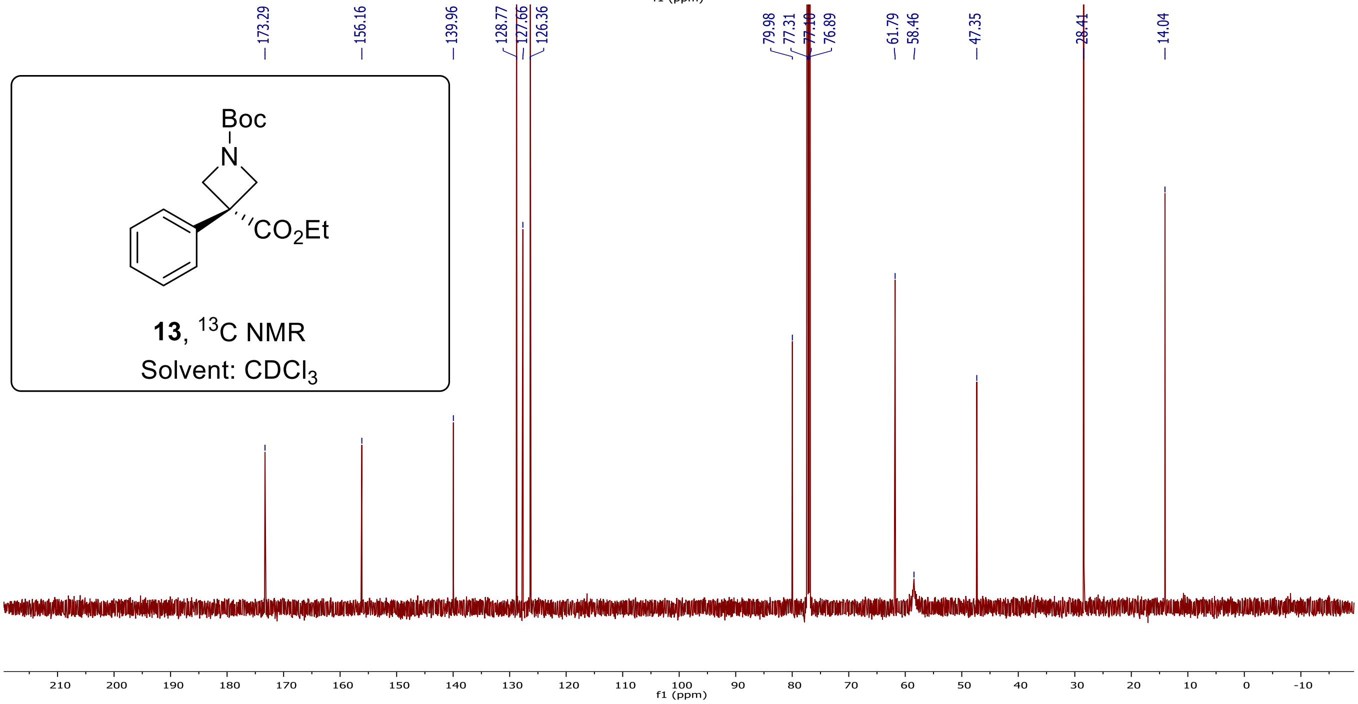

Supplementary Figure 129. NMR spectra of **13**

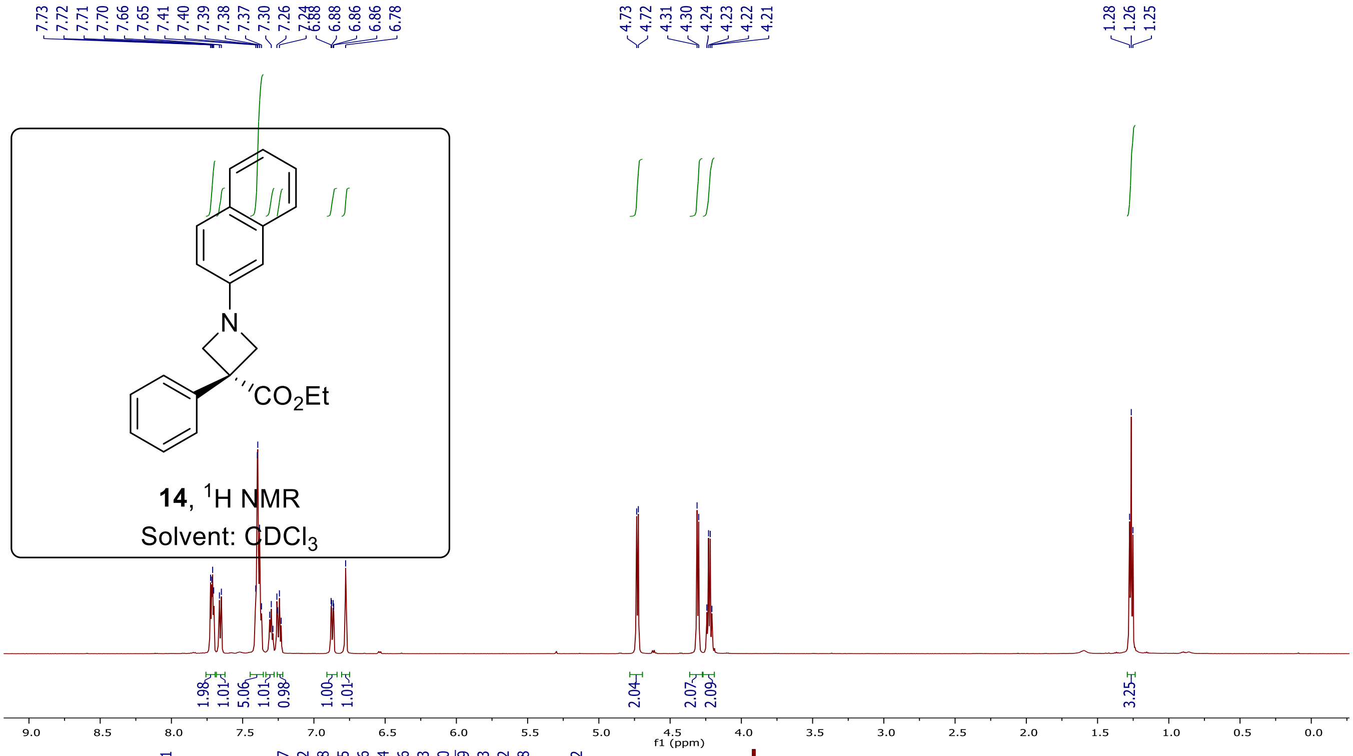

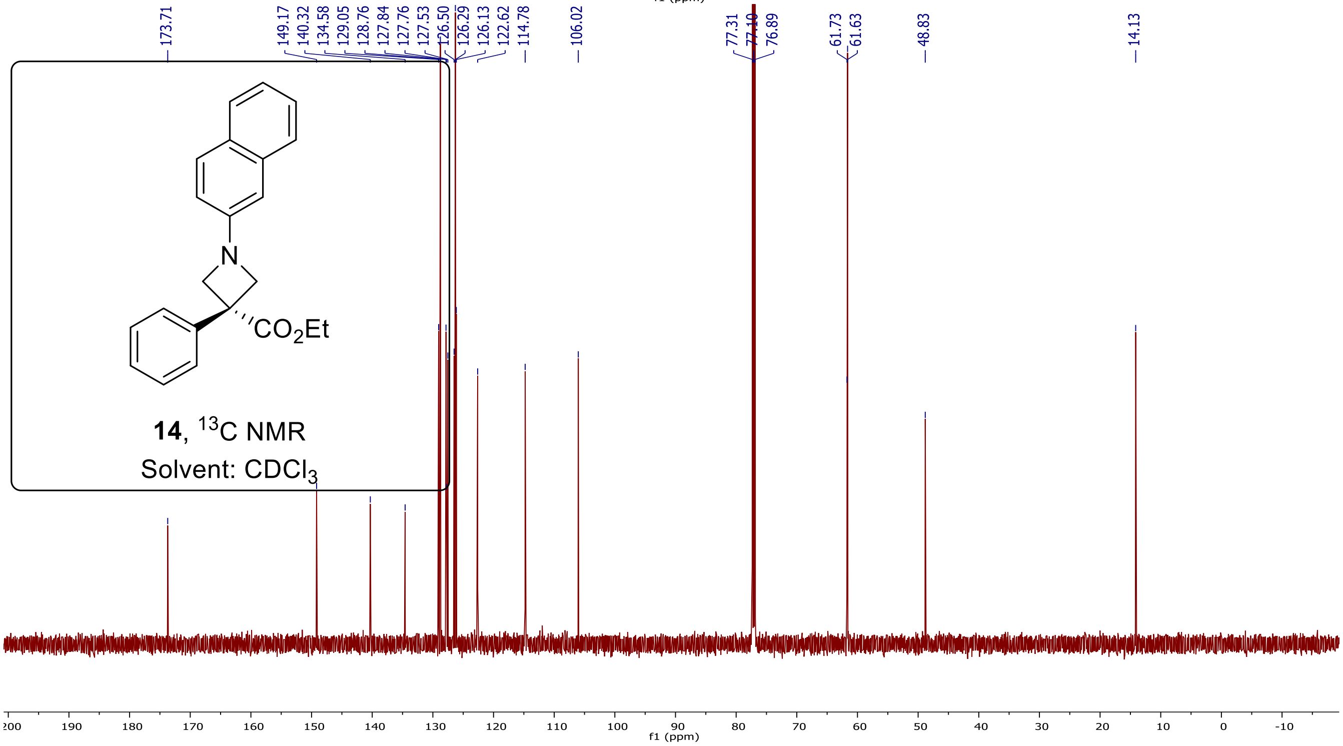

Supplementary Figure 130. NMR spectra of **14**

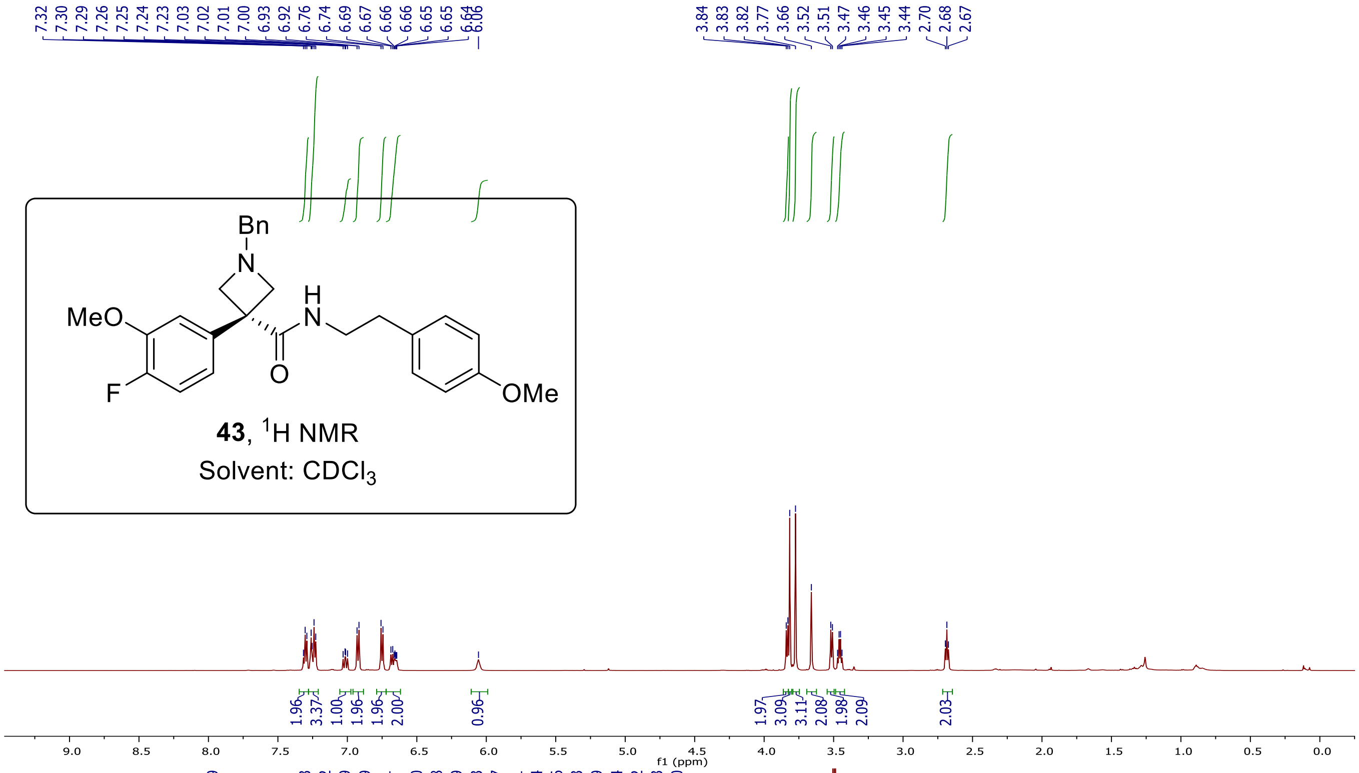

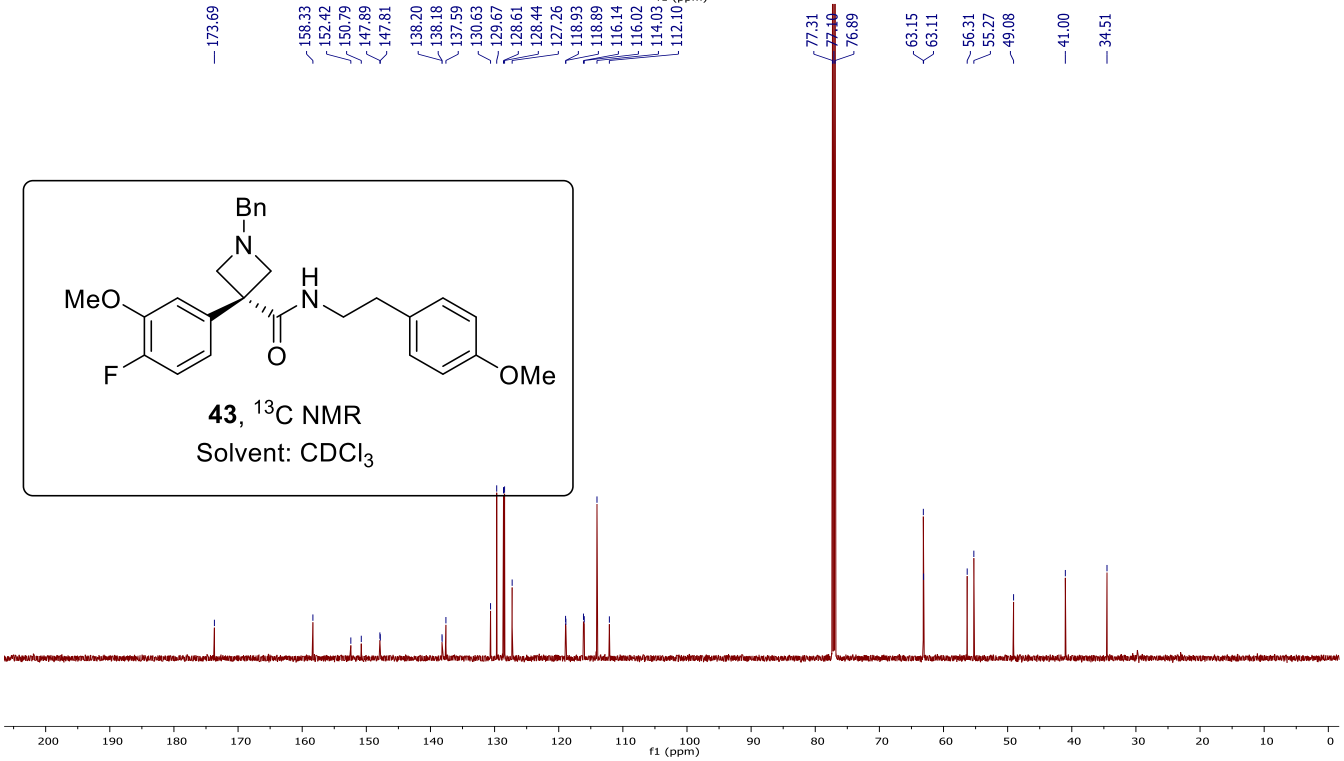

Supplementary Figure 131. NMR spectra of **43**

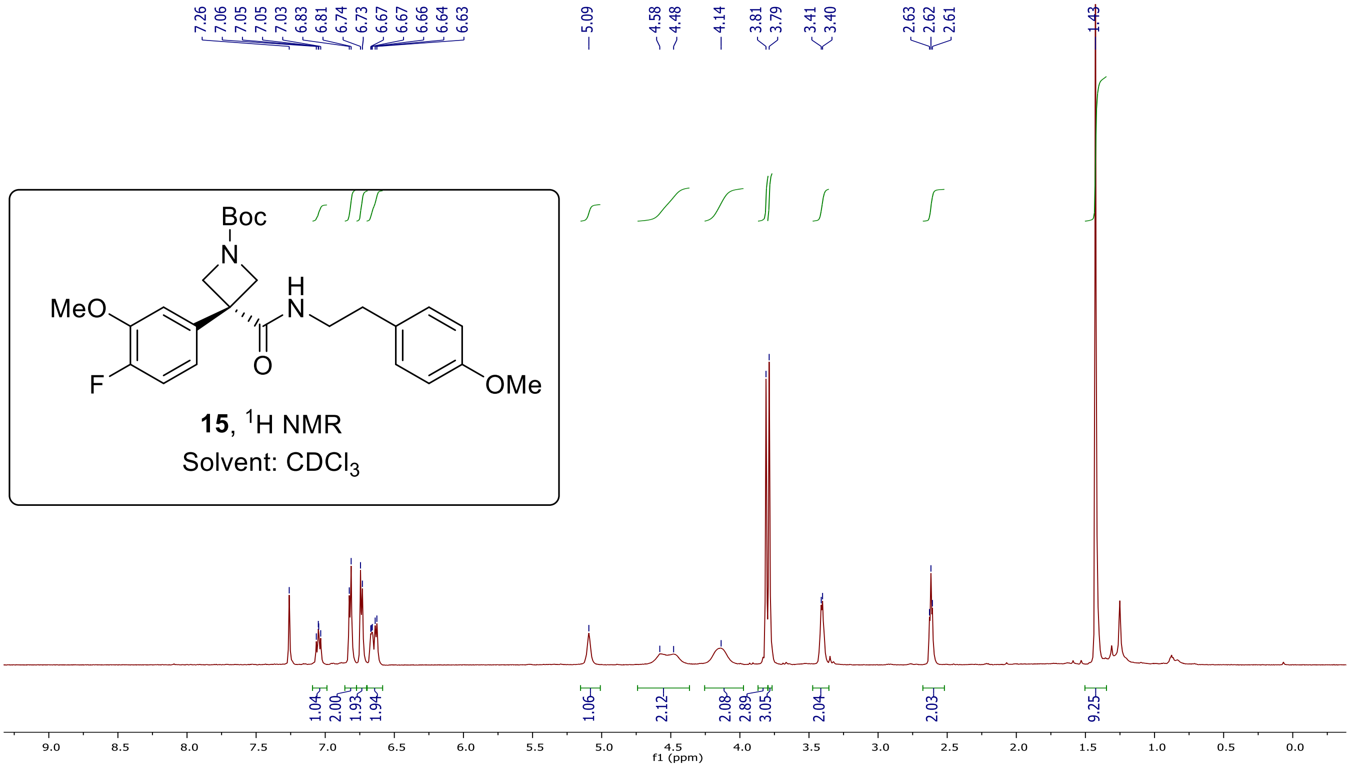

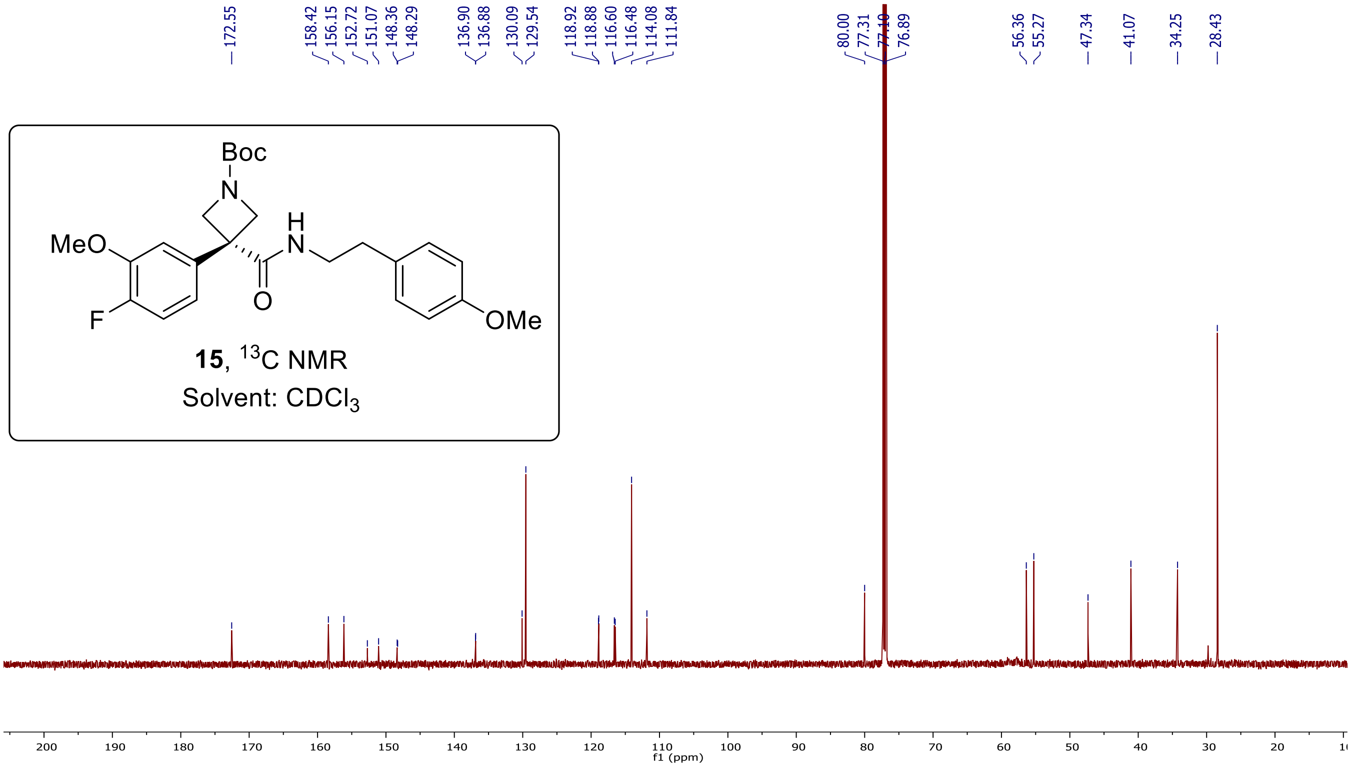

Supplementary Figure 132. NMR spectra of **15**

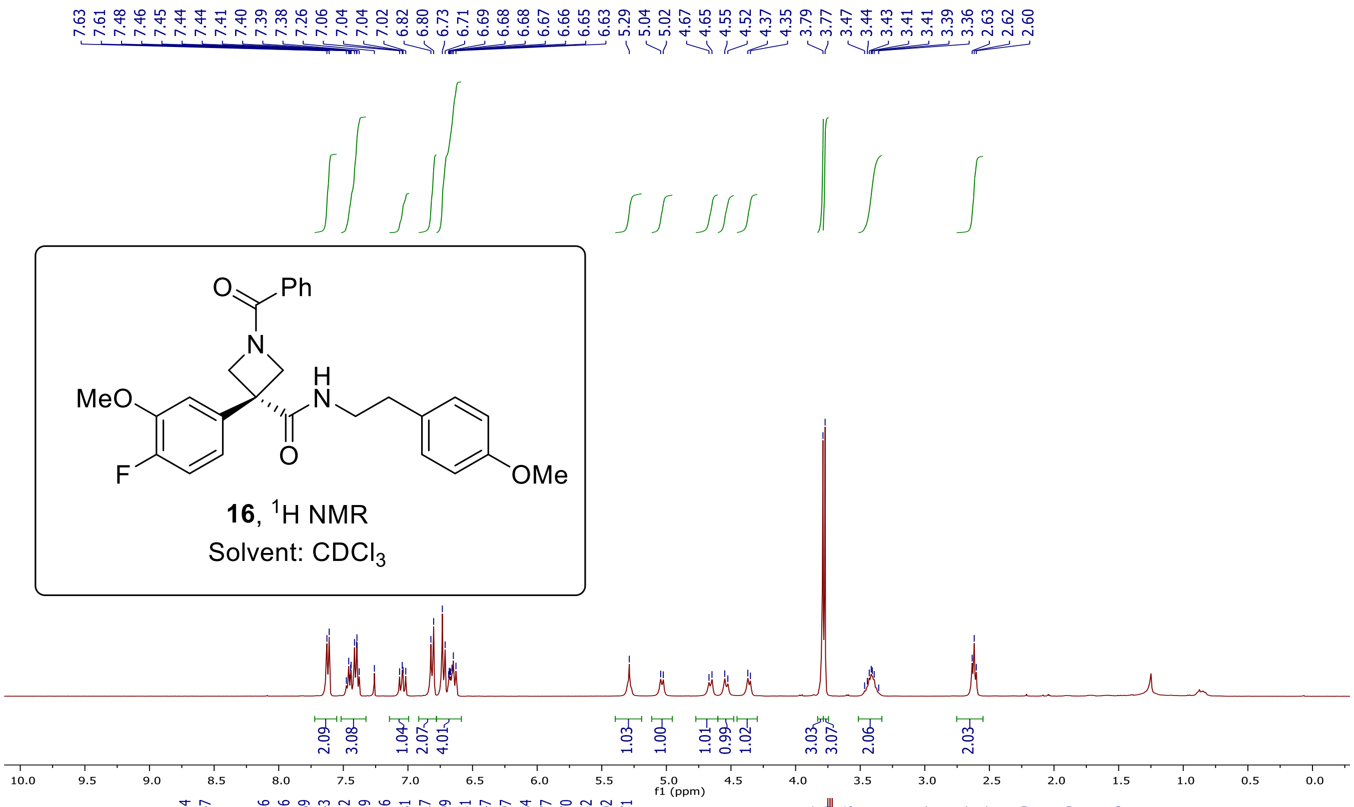

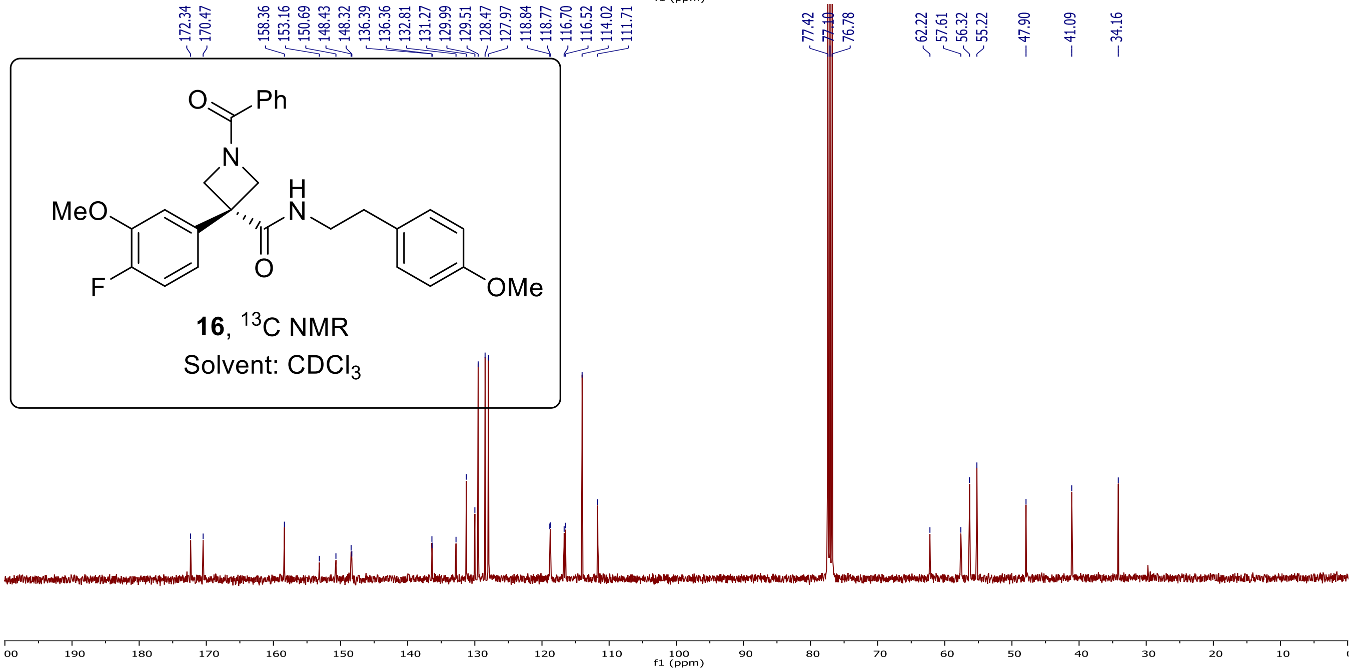

Supplementary Figure 133. NMR spectra of **16**

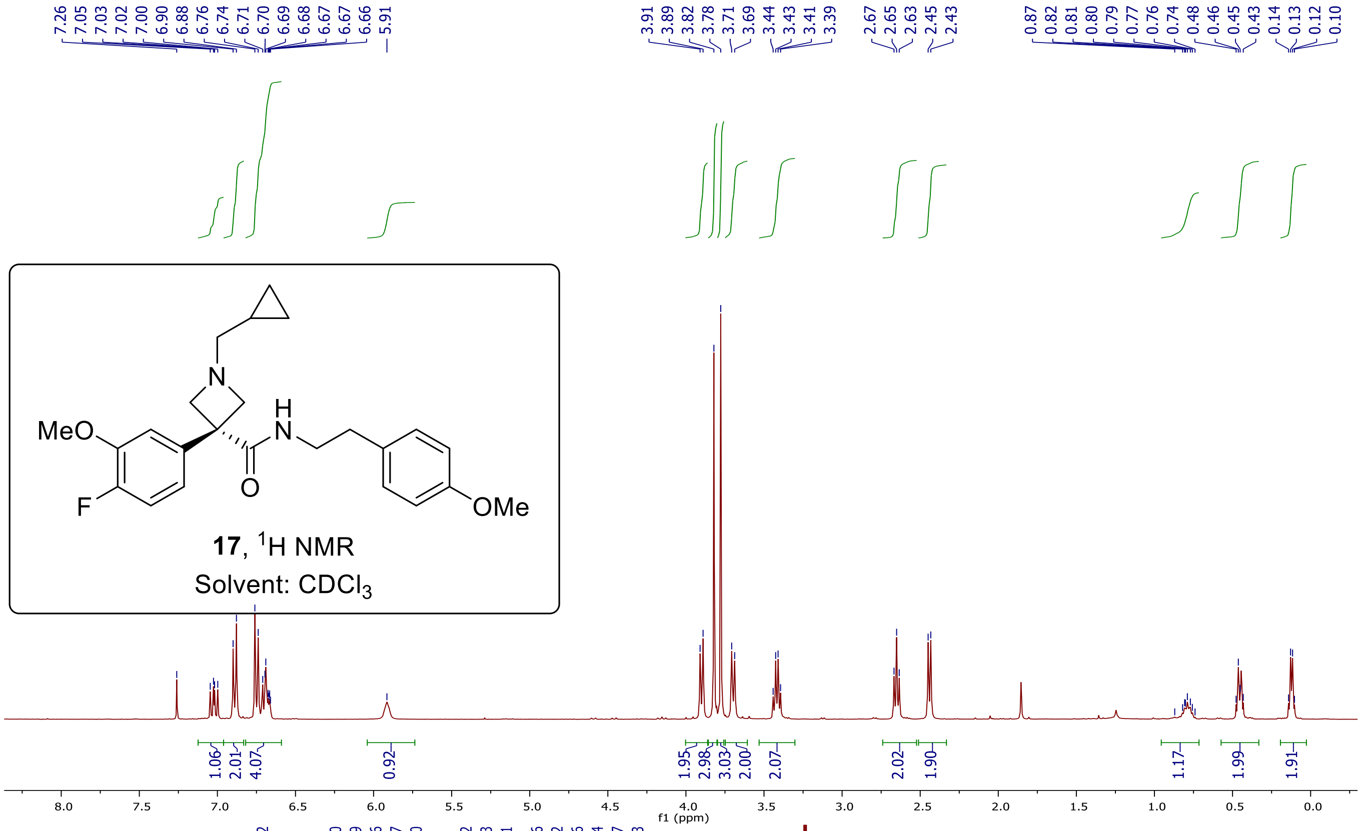

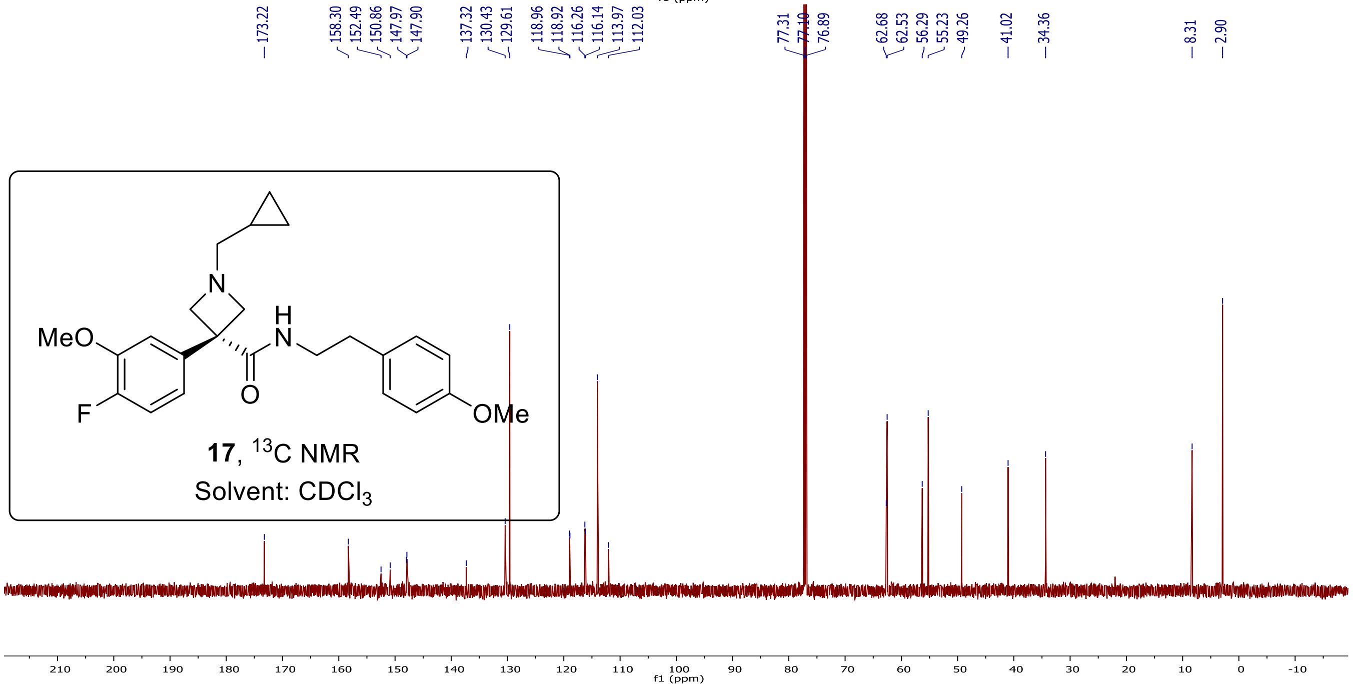

Supplementary Figure 134. NMR spectra of **17**

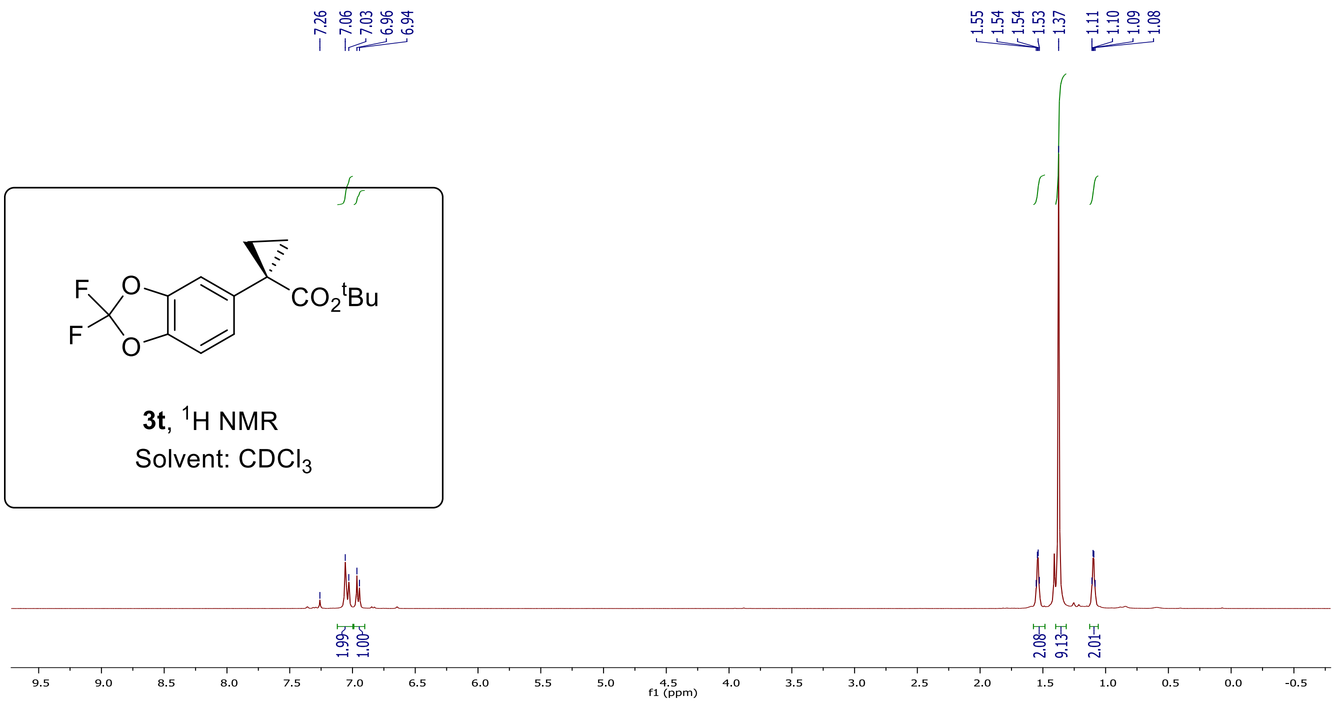

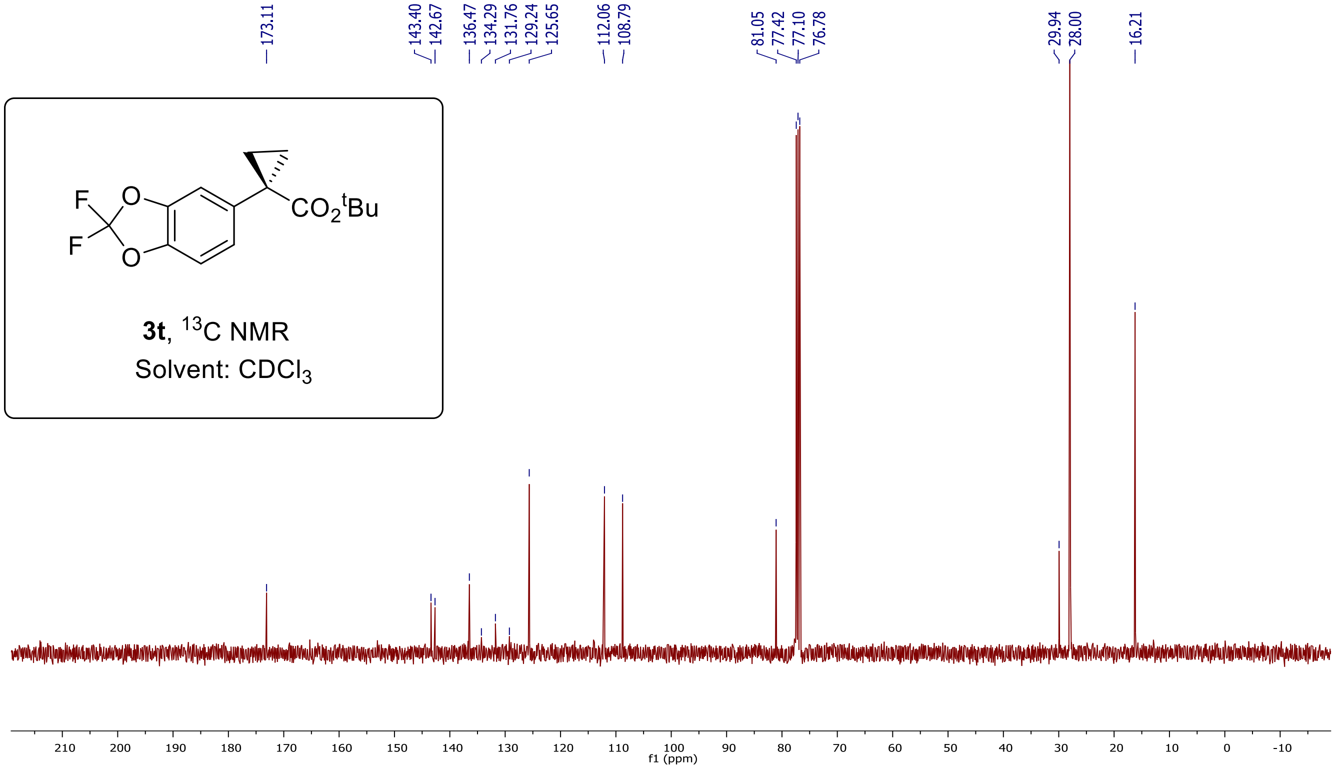

Supplementary Figure 135. NMR spectra of **3t**

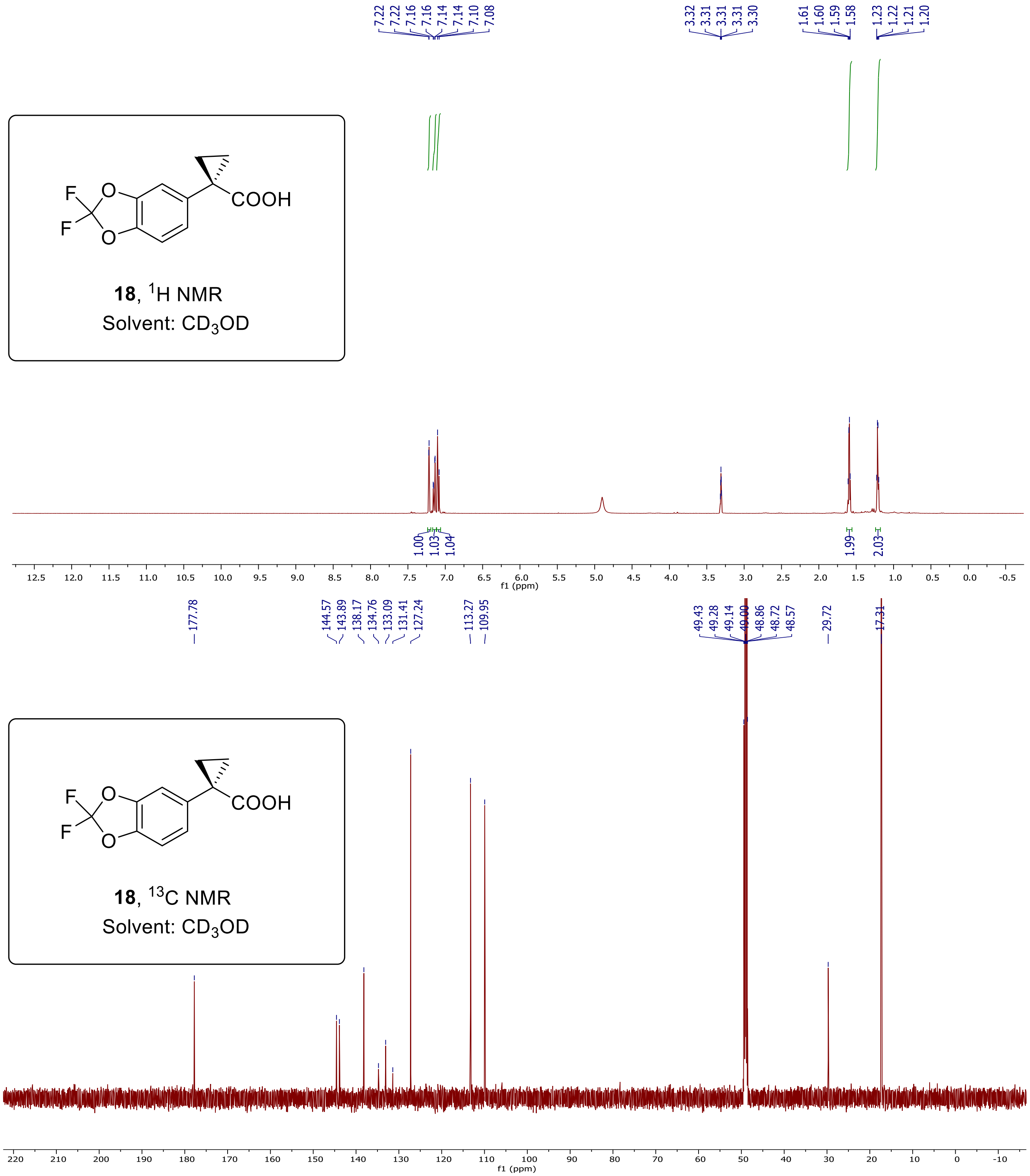

Supplementary Figure 136. NMR spectra of **18**

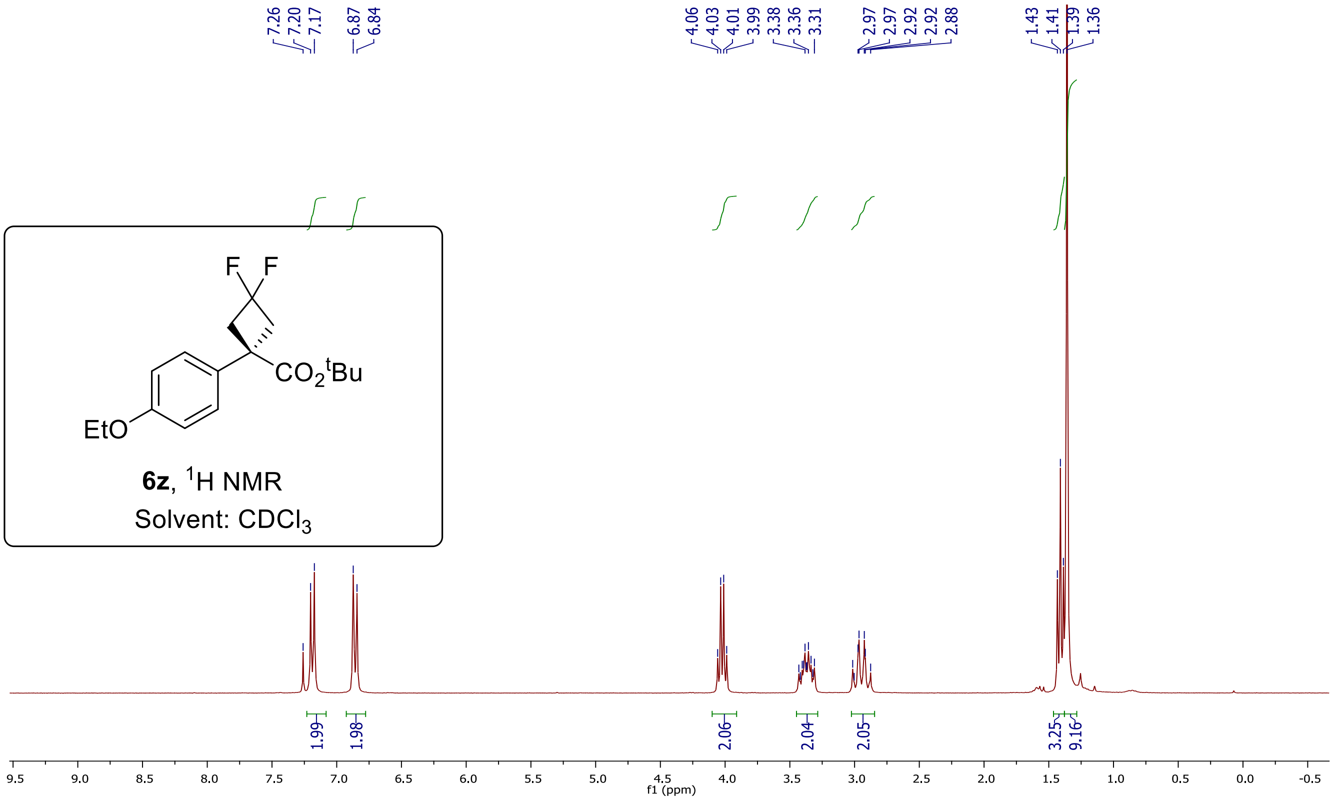

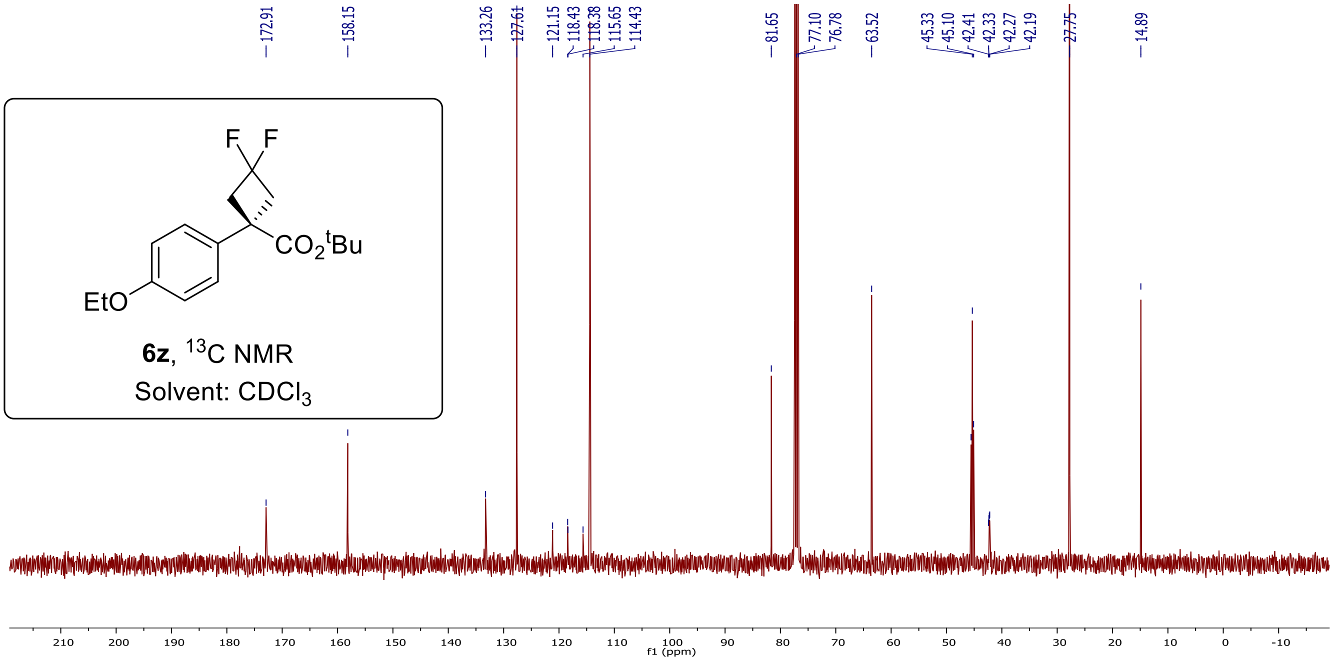

Supplementary Figure 137. NMR spectra of **6z**

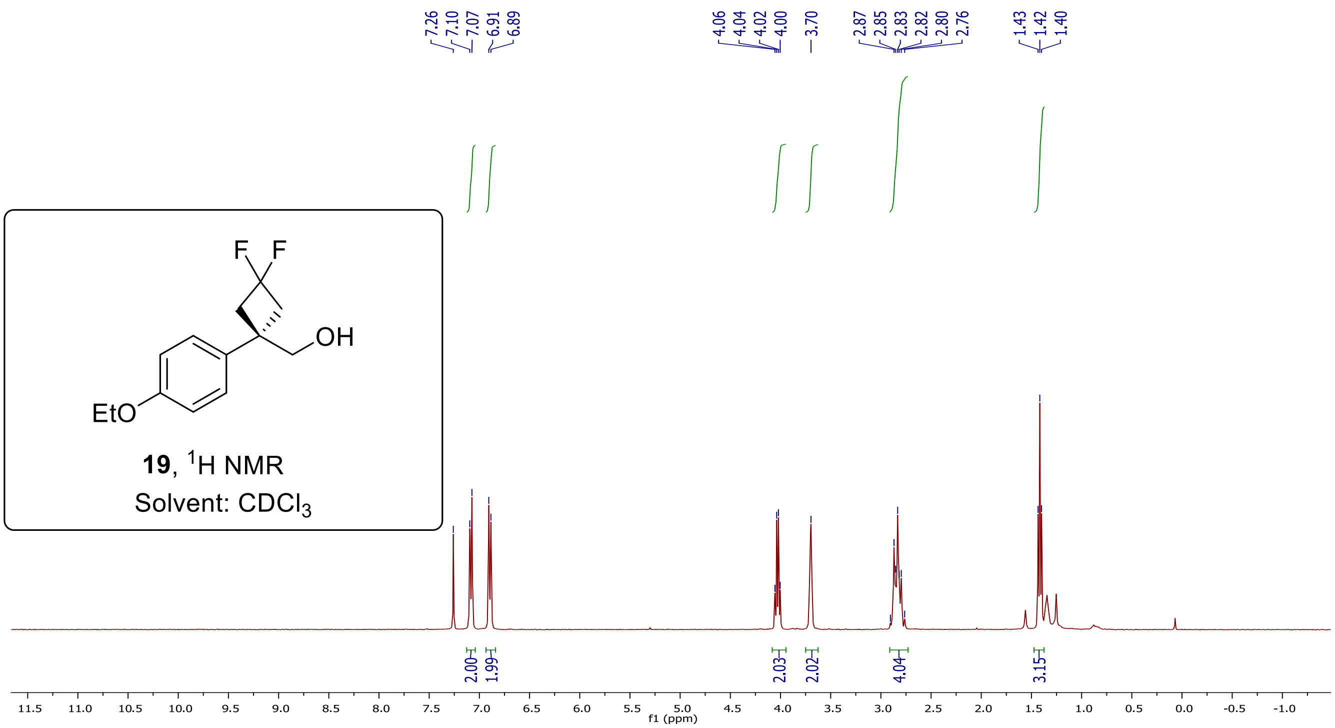

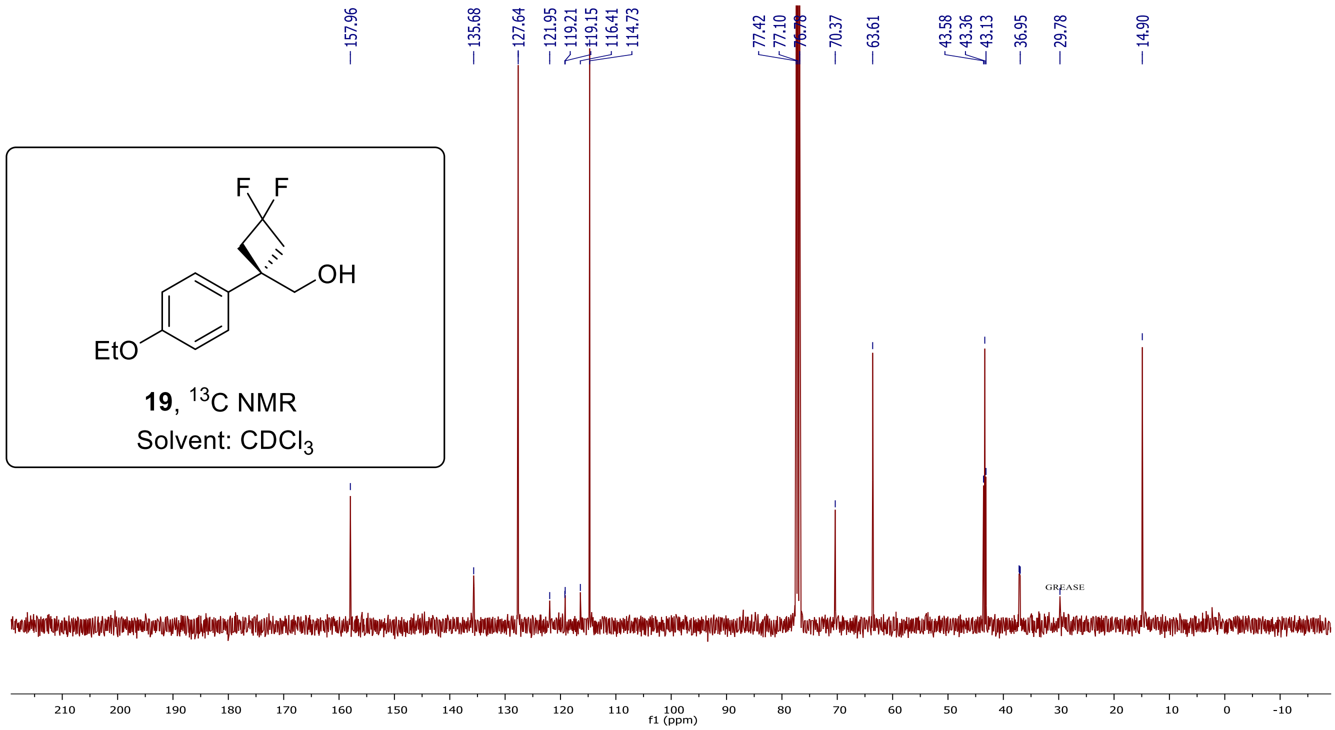

Supplementary Figure 138. NMR spectra of **19**

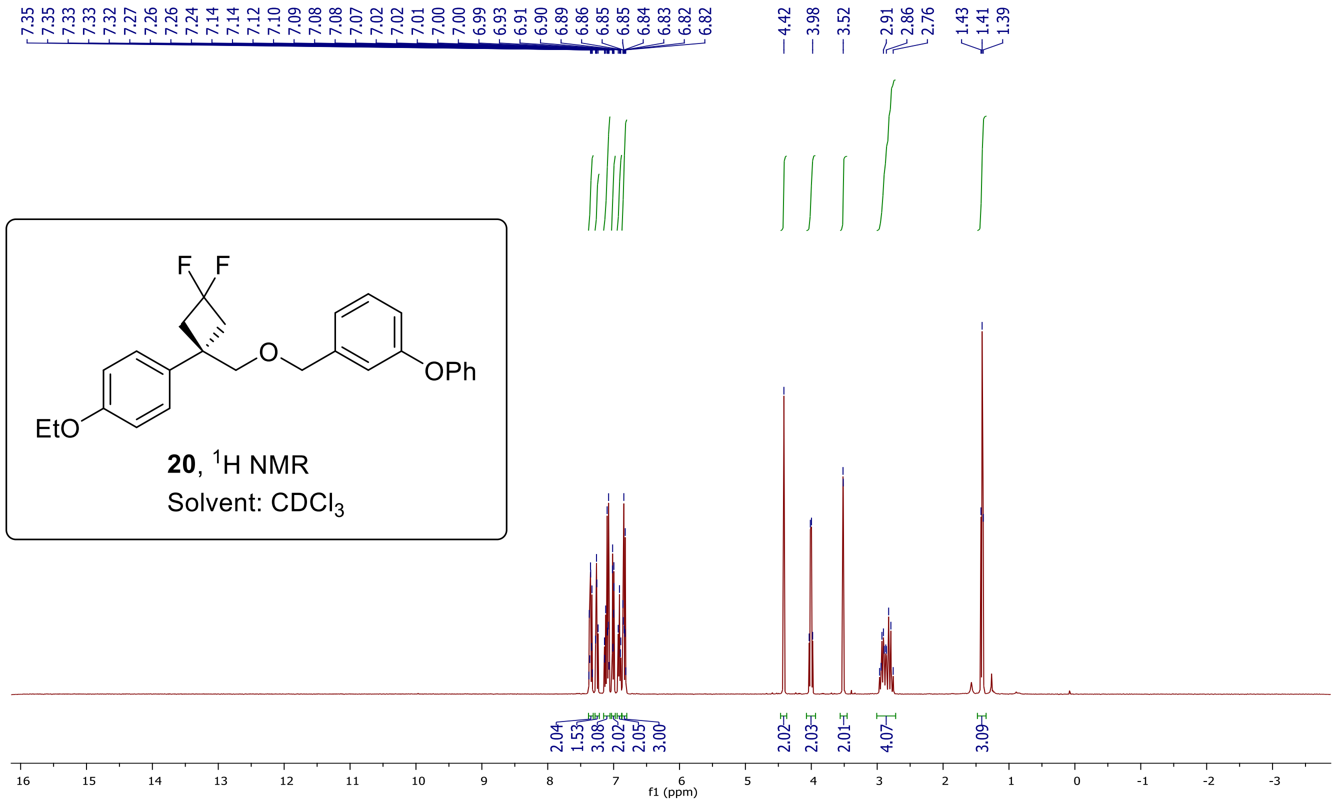

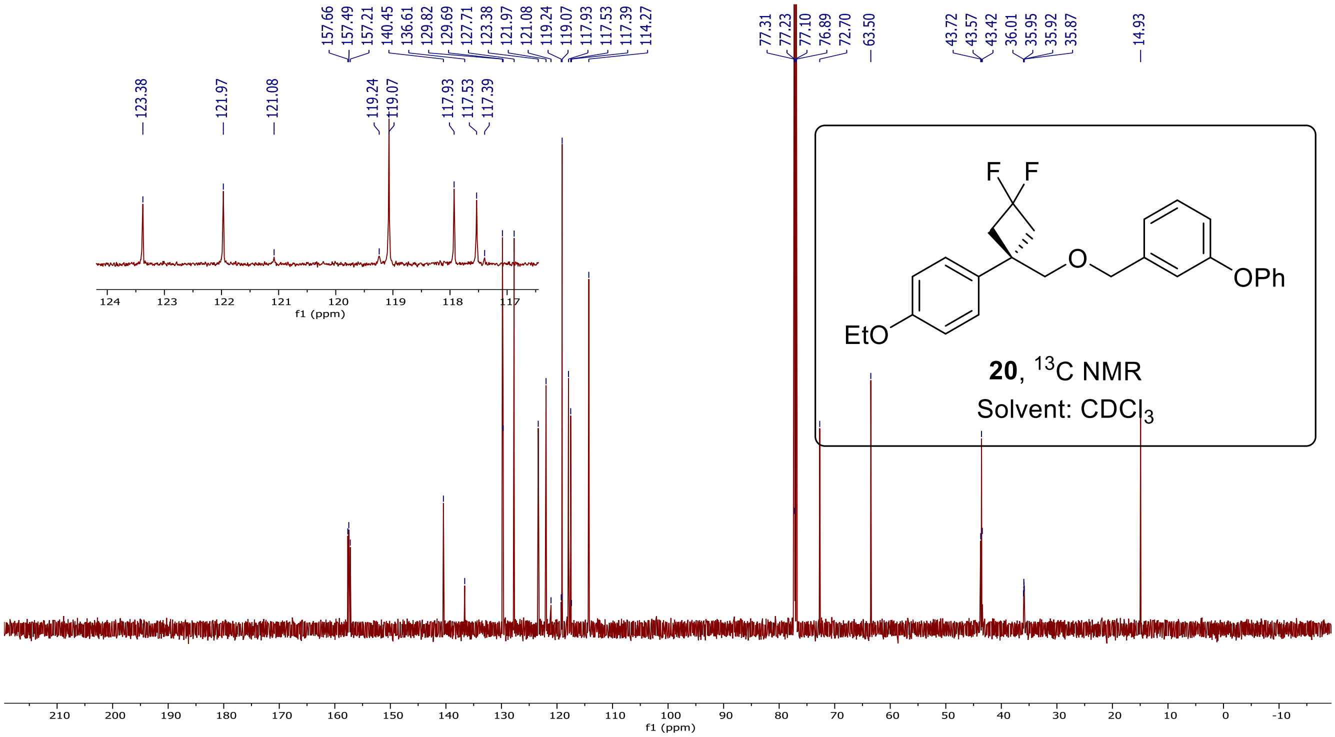

Supplementary Figure 139. NMR spectra of **20**

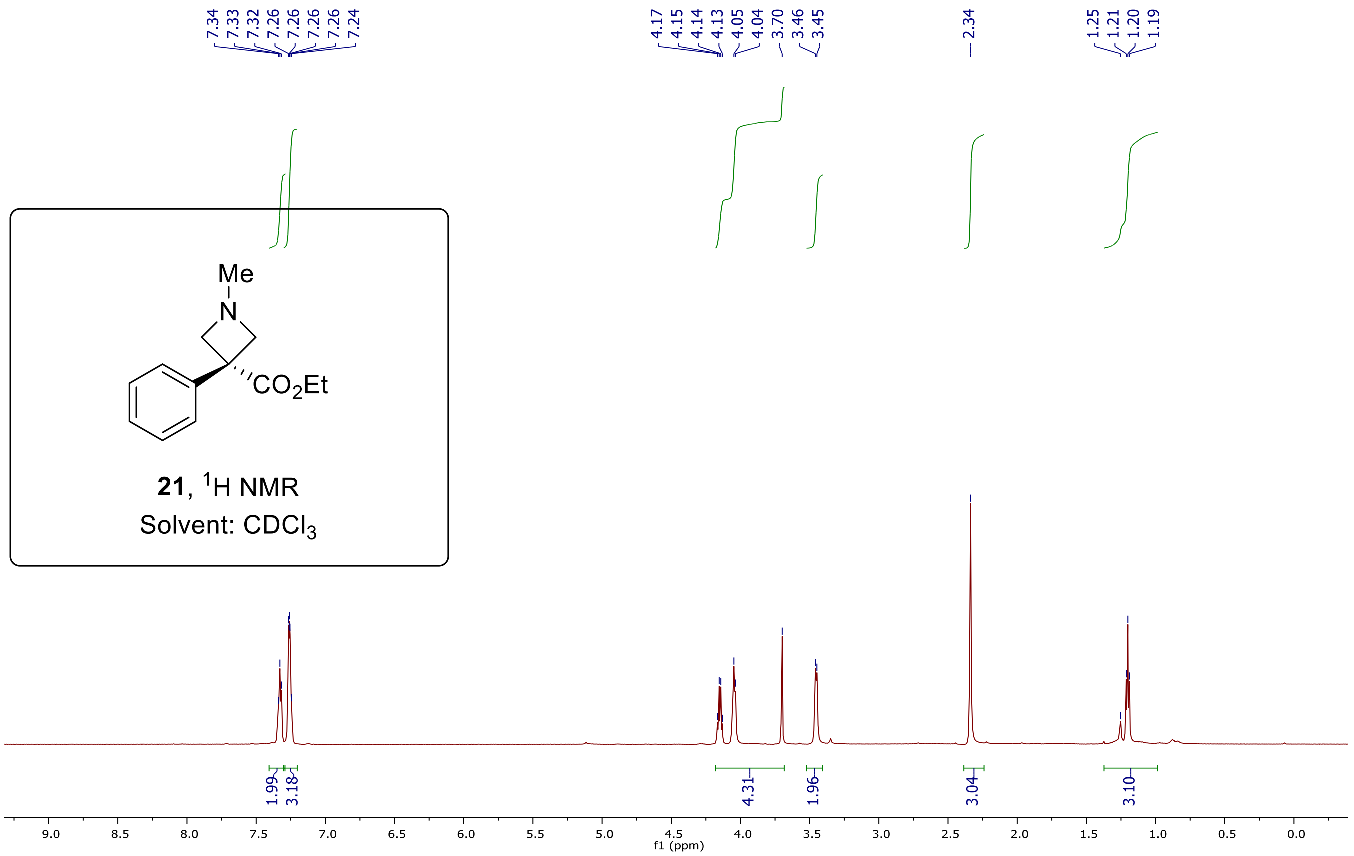

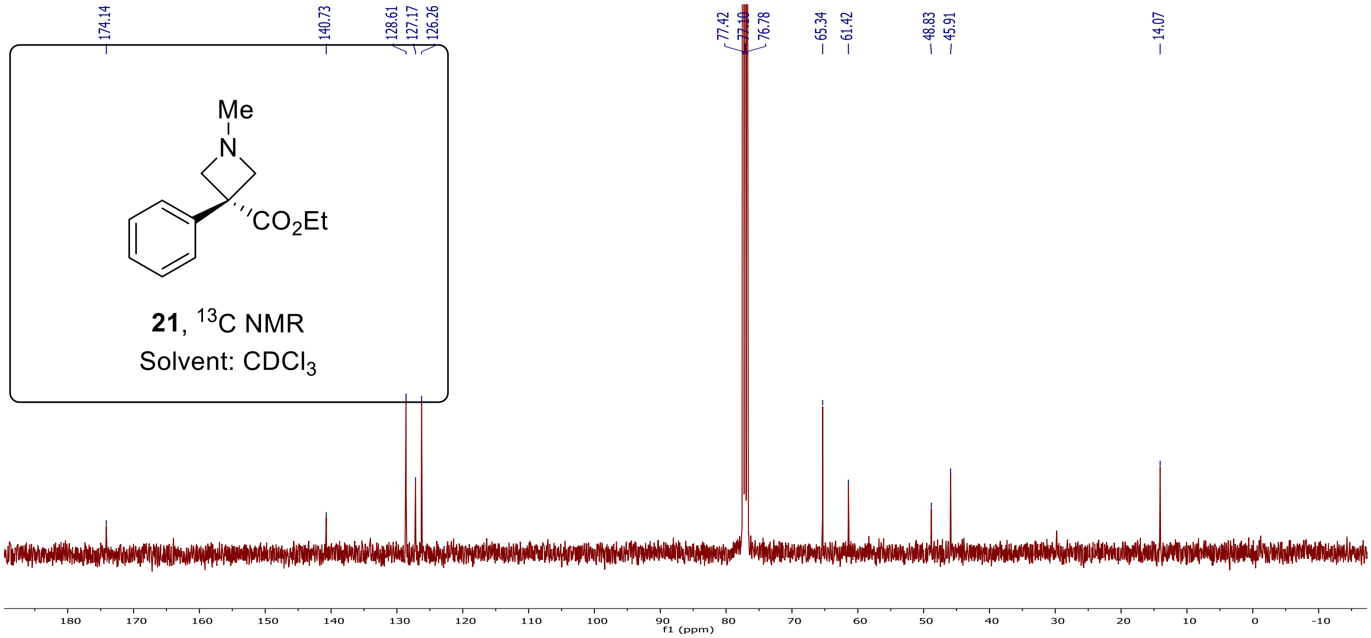

Supplementary Figure 140. NMR spectra of **21**

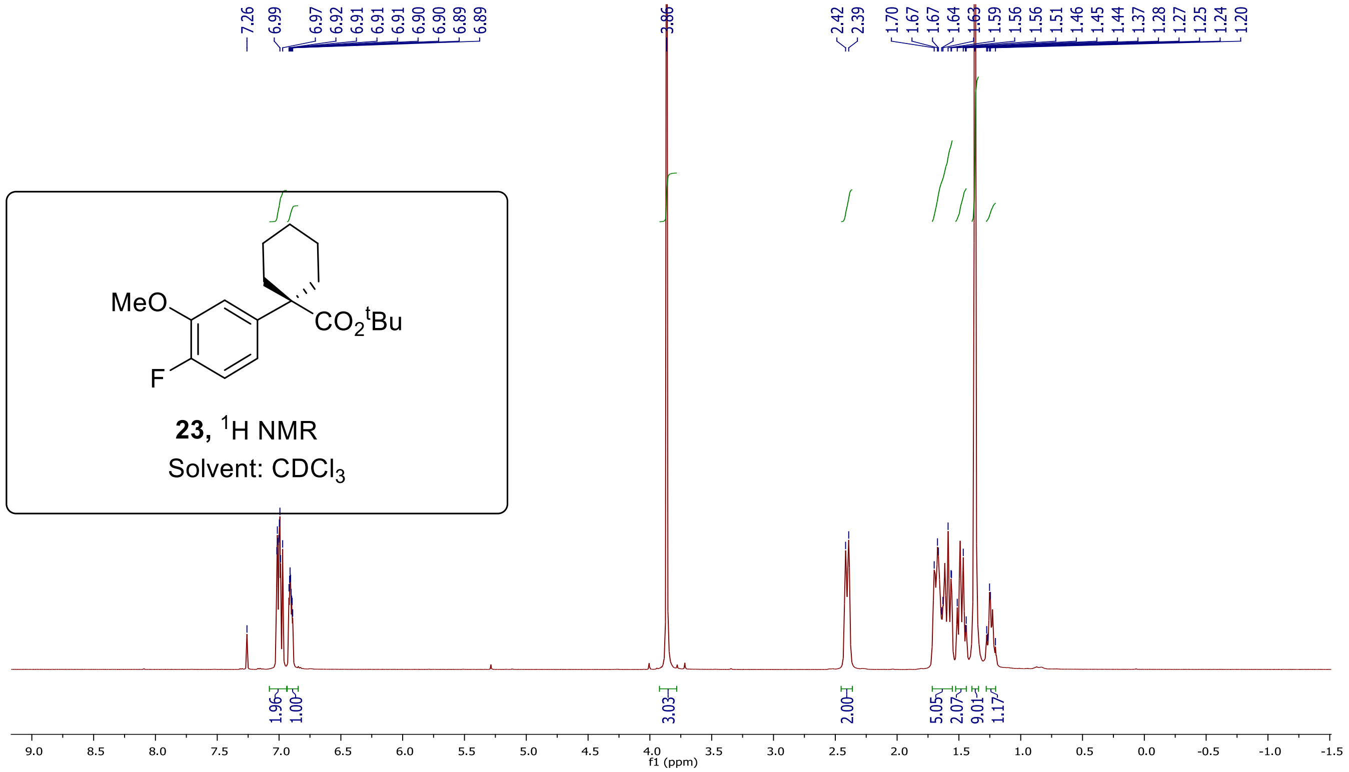

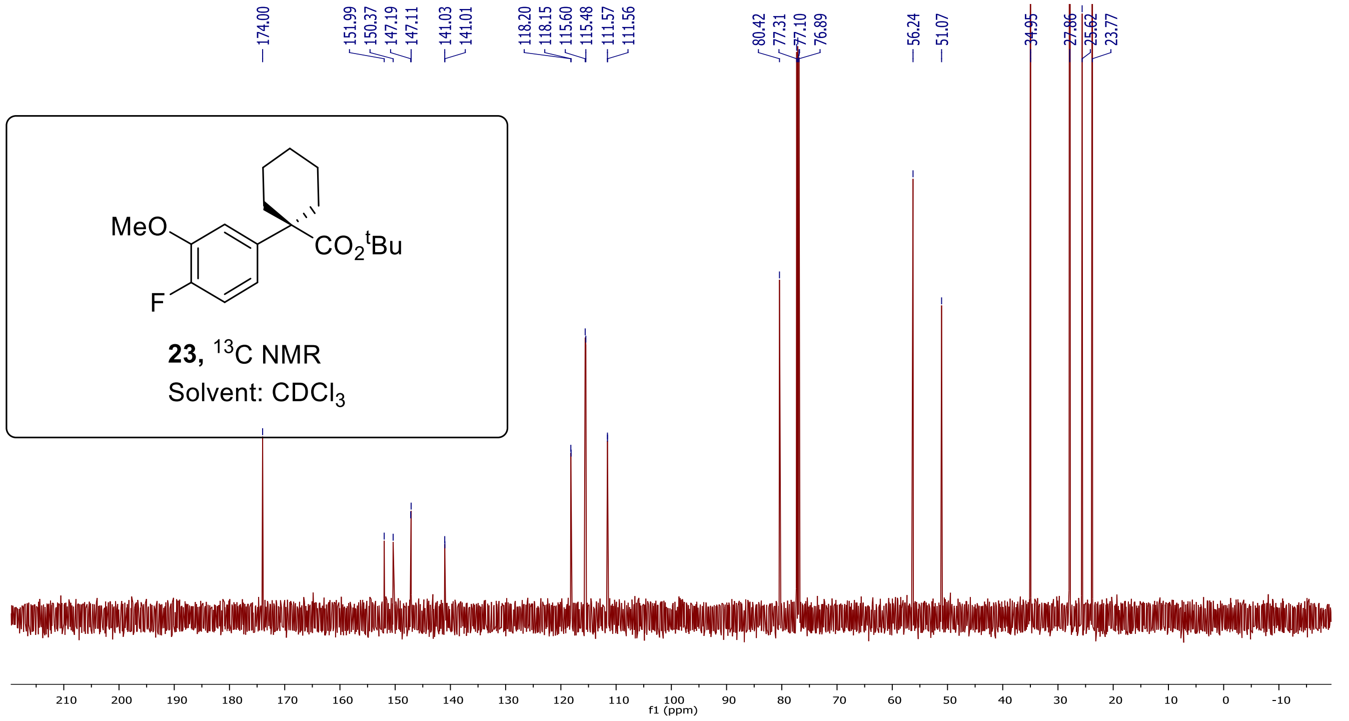

Supplementary Figure 141. NMR spectra of **23**

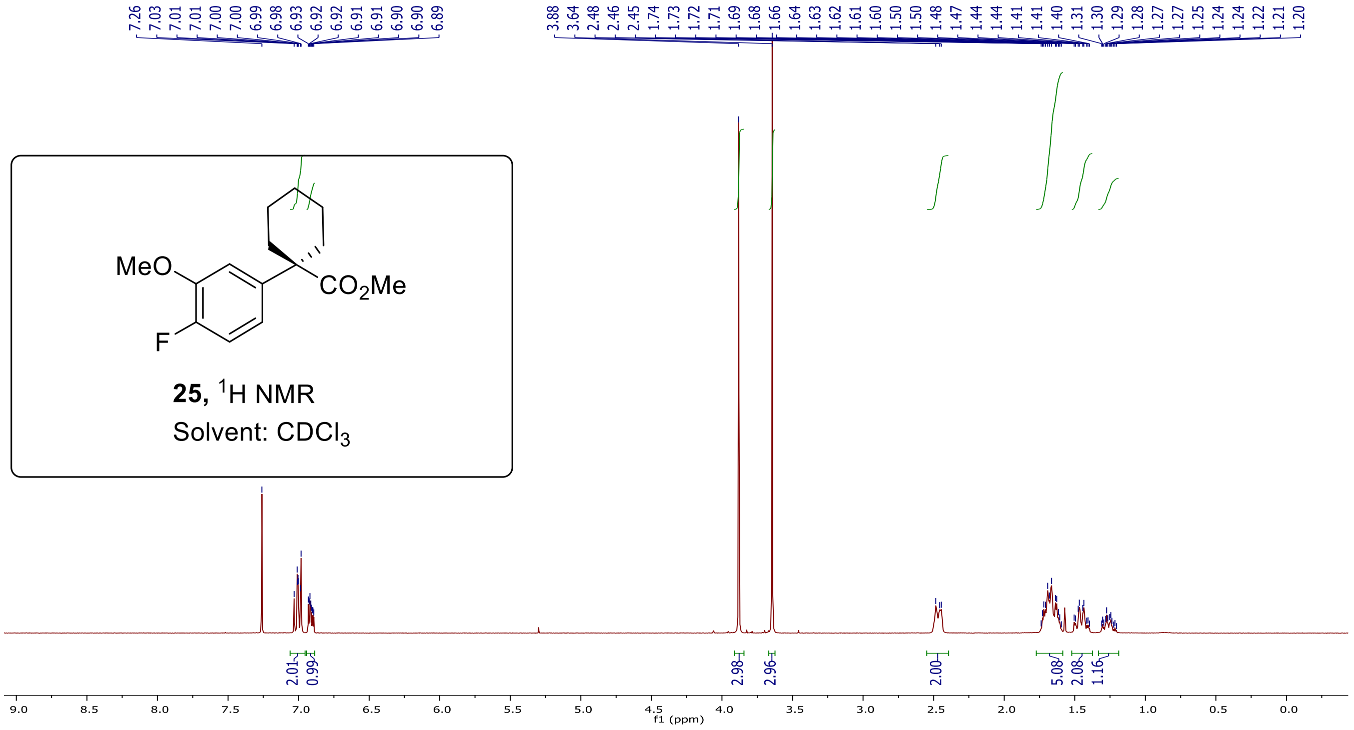

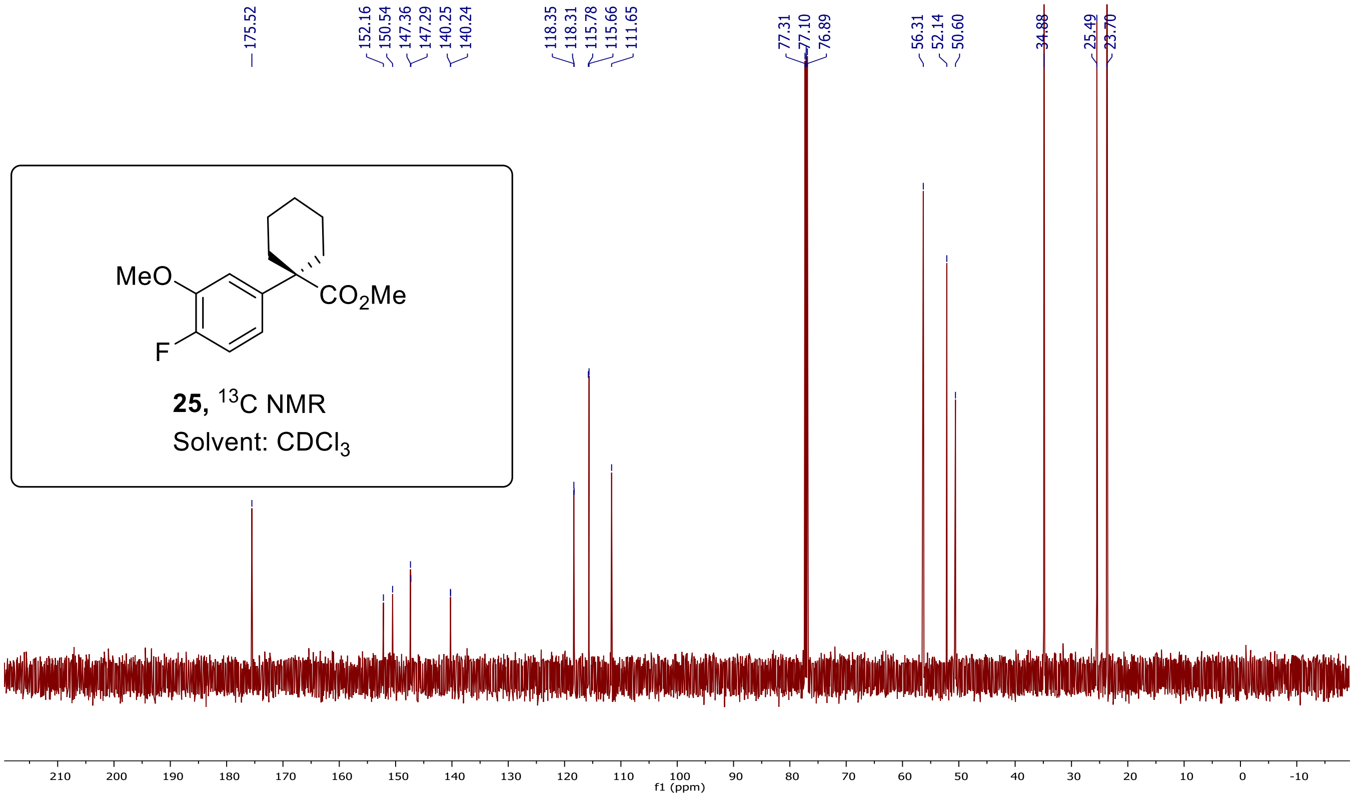

Supplementary Figure 142. NMR spectra of **25**

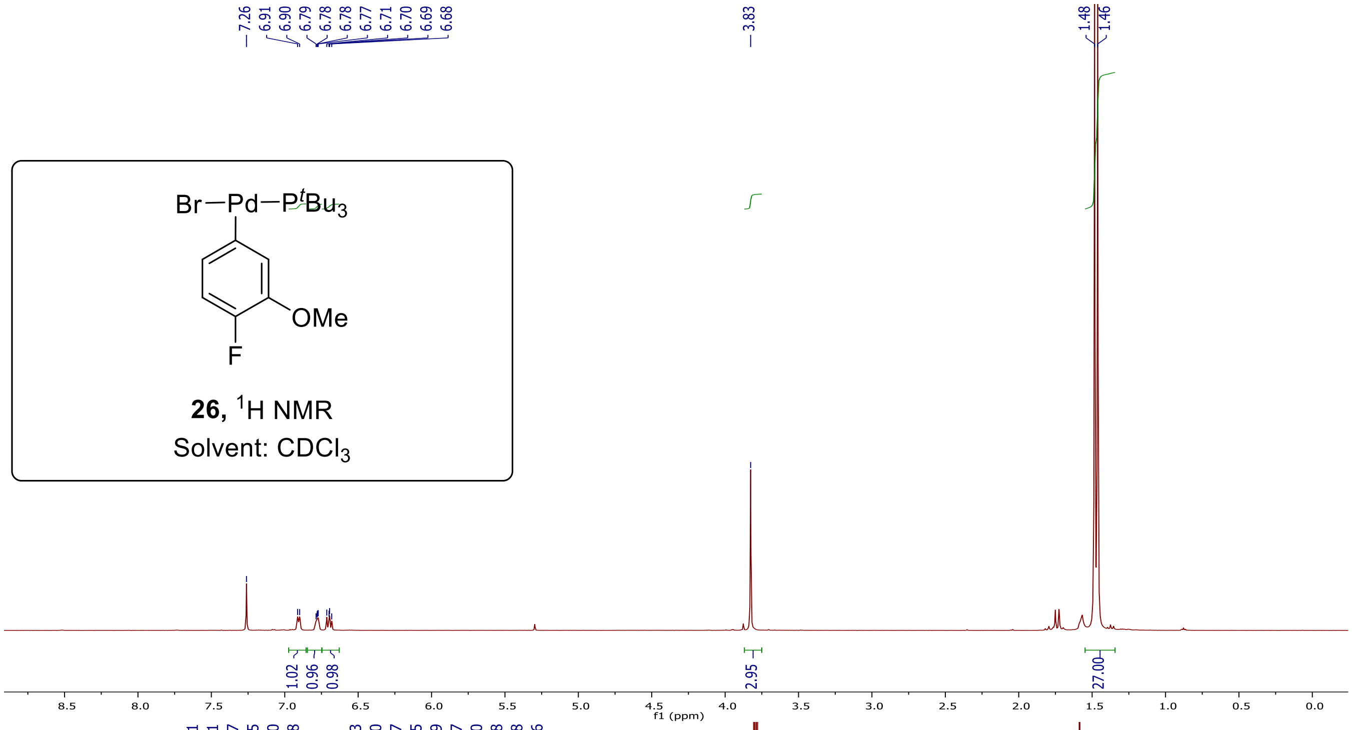

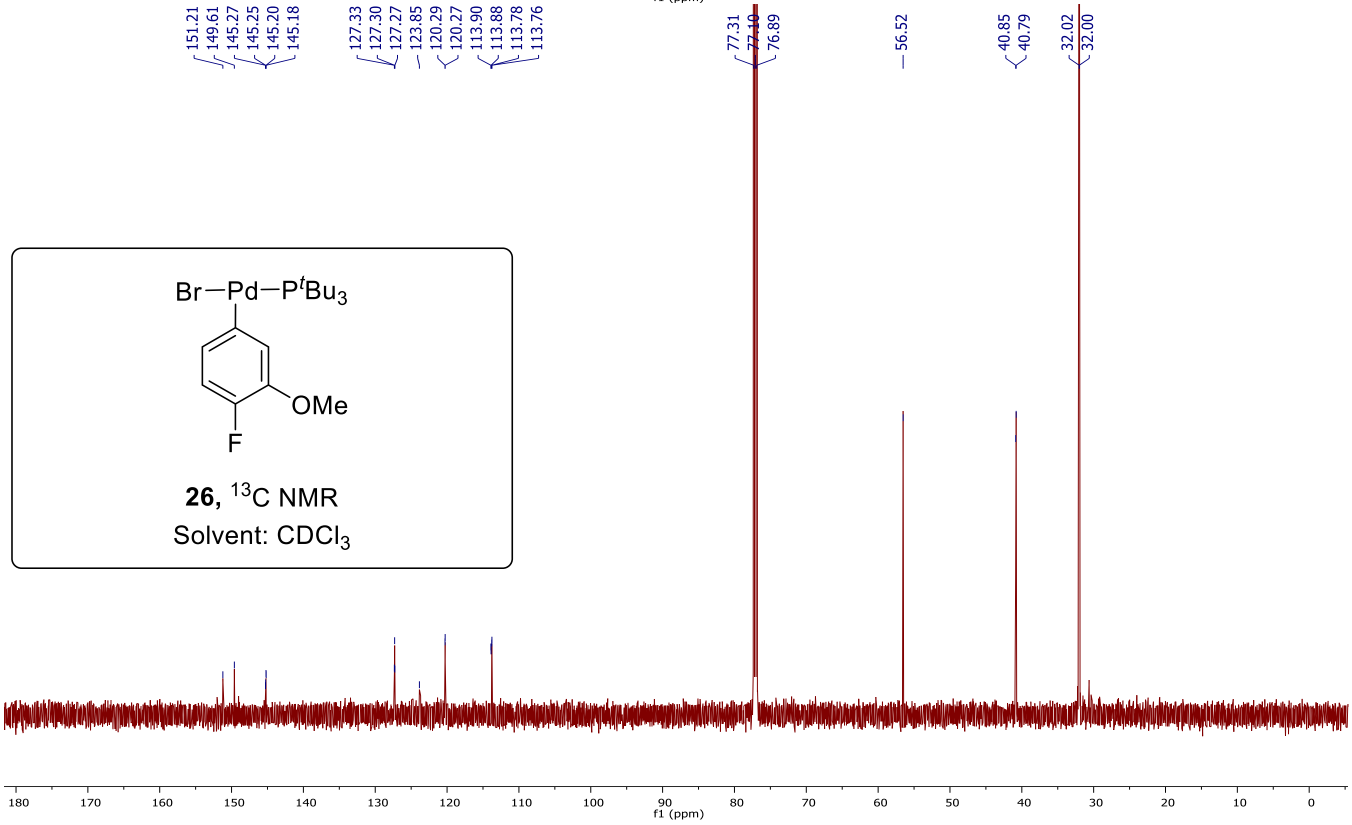

Supplementary Figure 143. NMR spectra of **26**

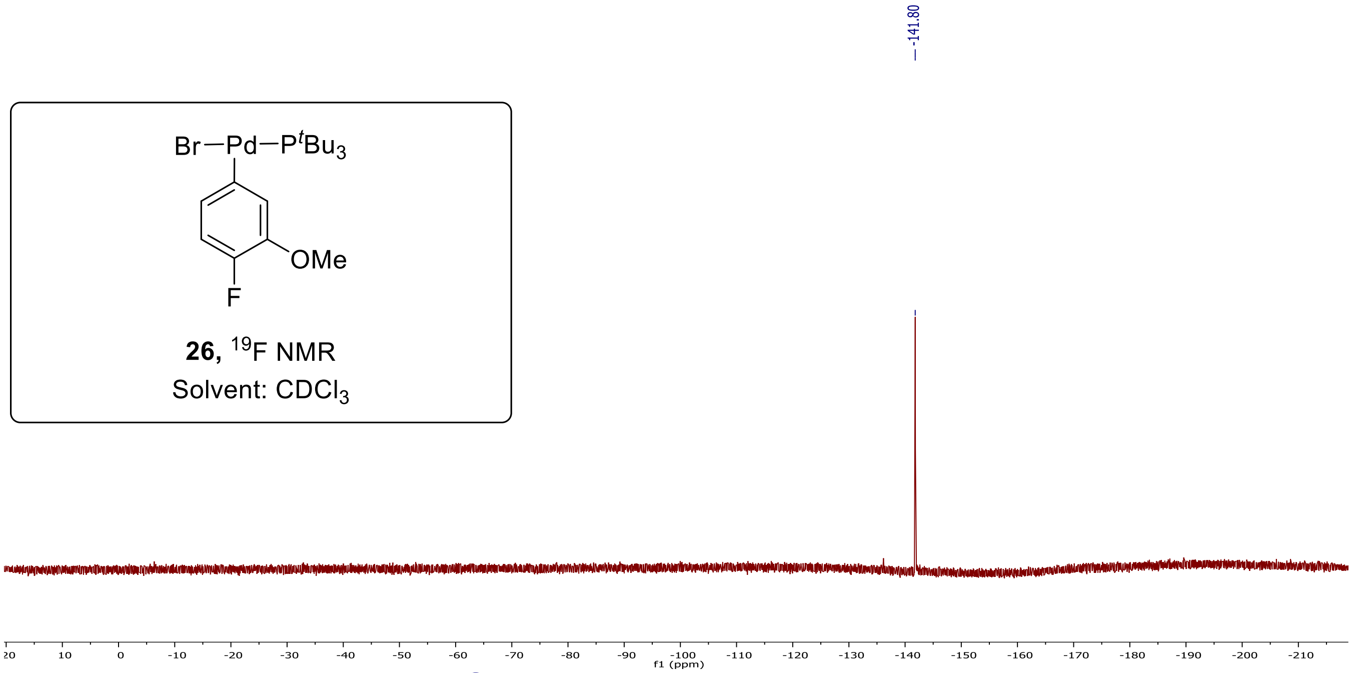

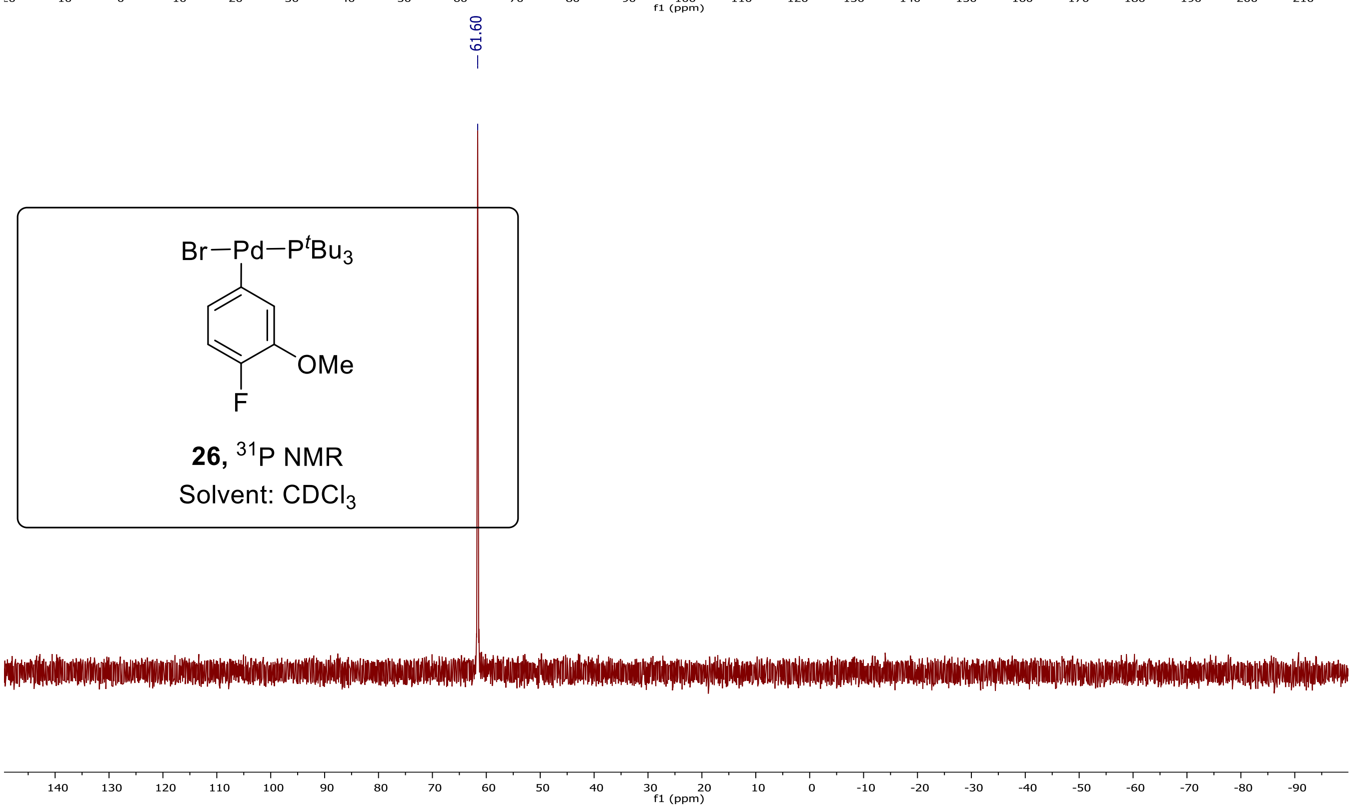

Supplementary Figure 144. NMR spectra of **26**

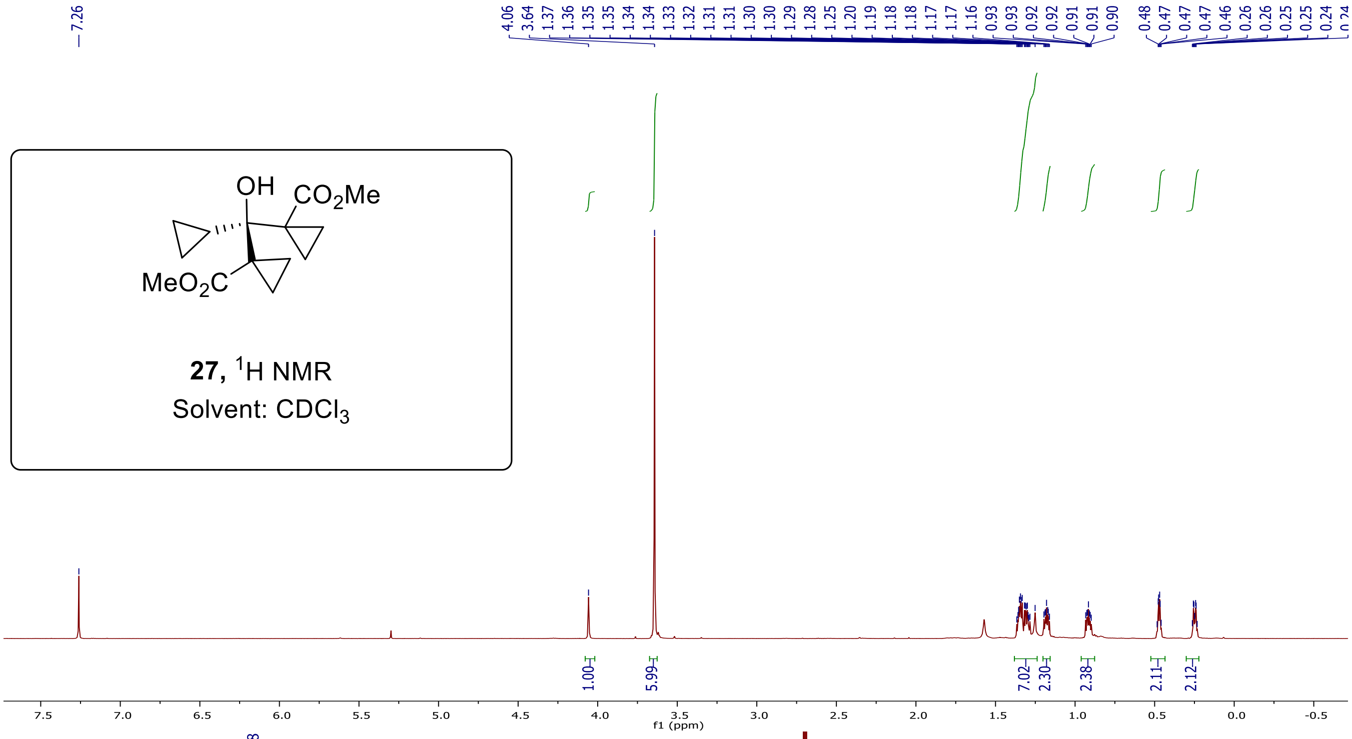

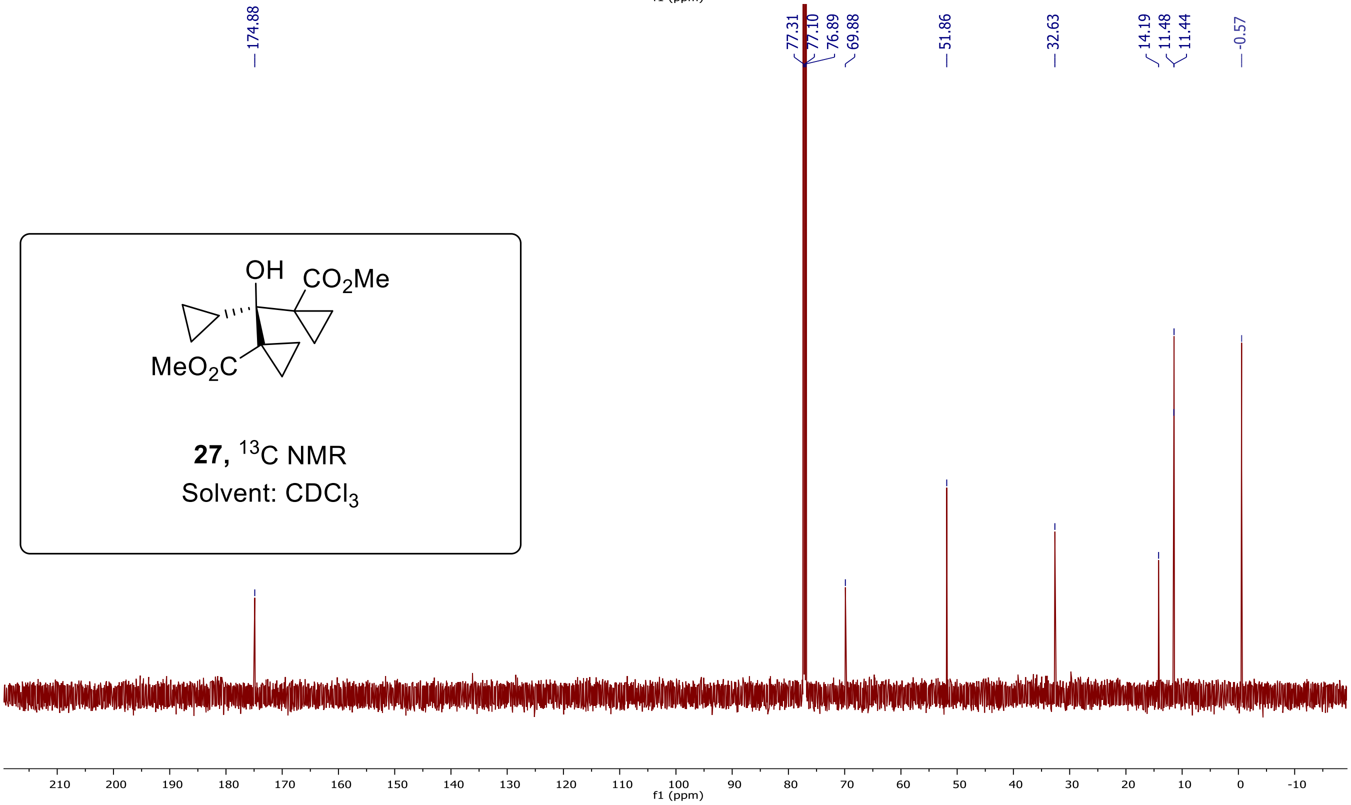

Supplementary Figure 145. NMR spectra of **27**

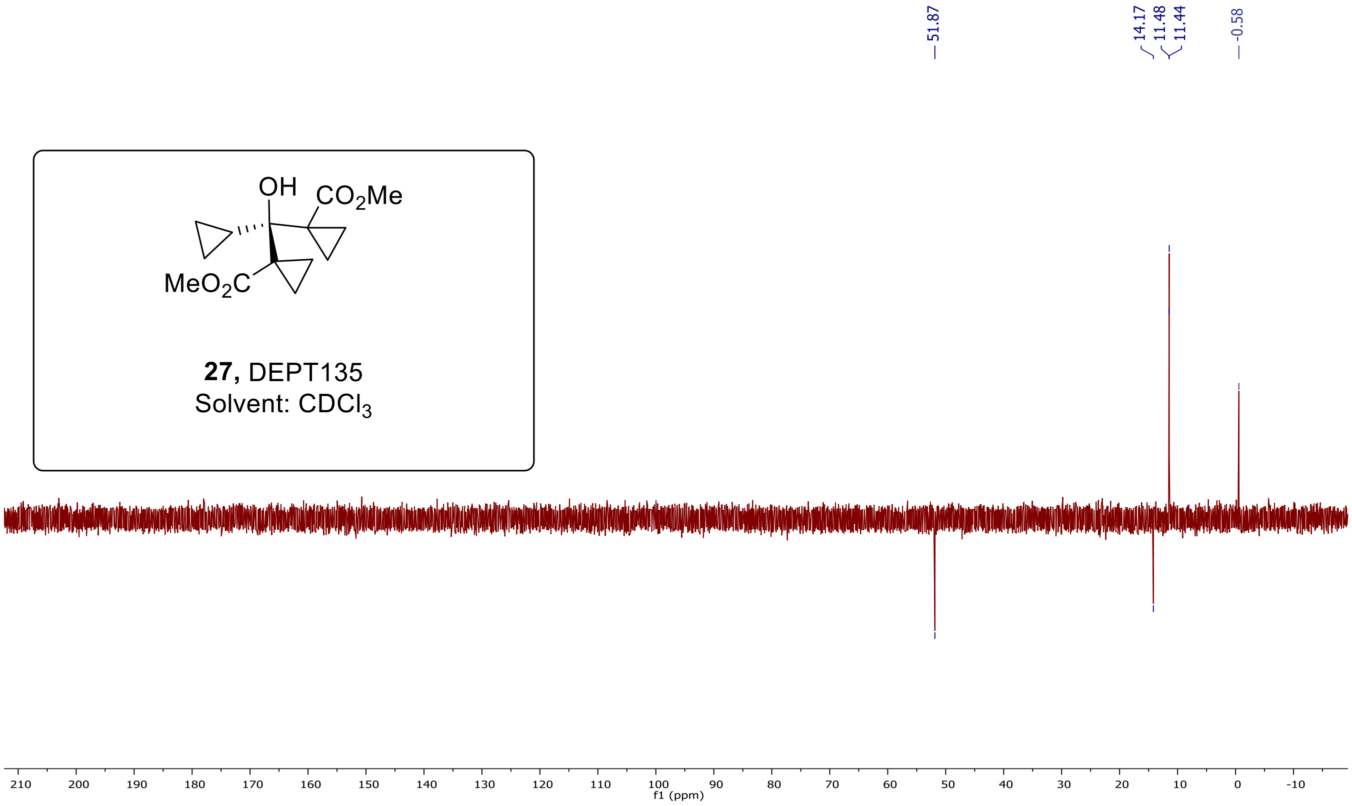

Supplementary Figure 146. DEPT135 spectrum of **27**

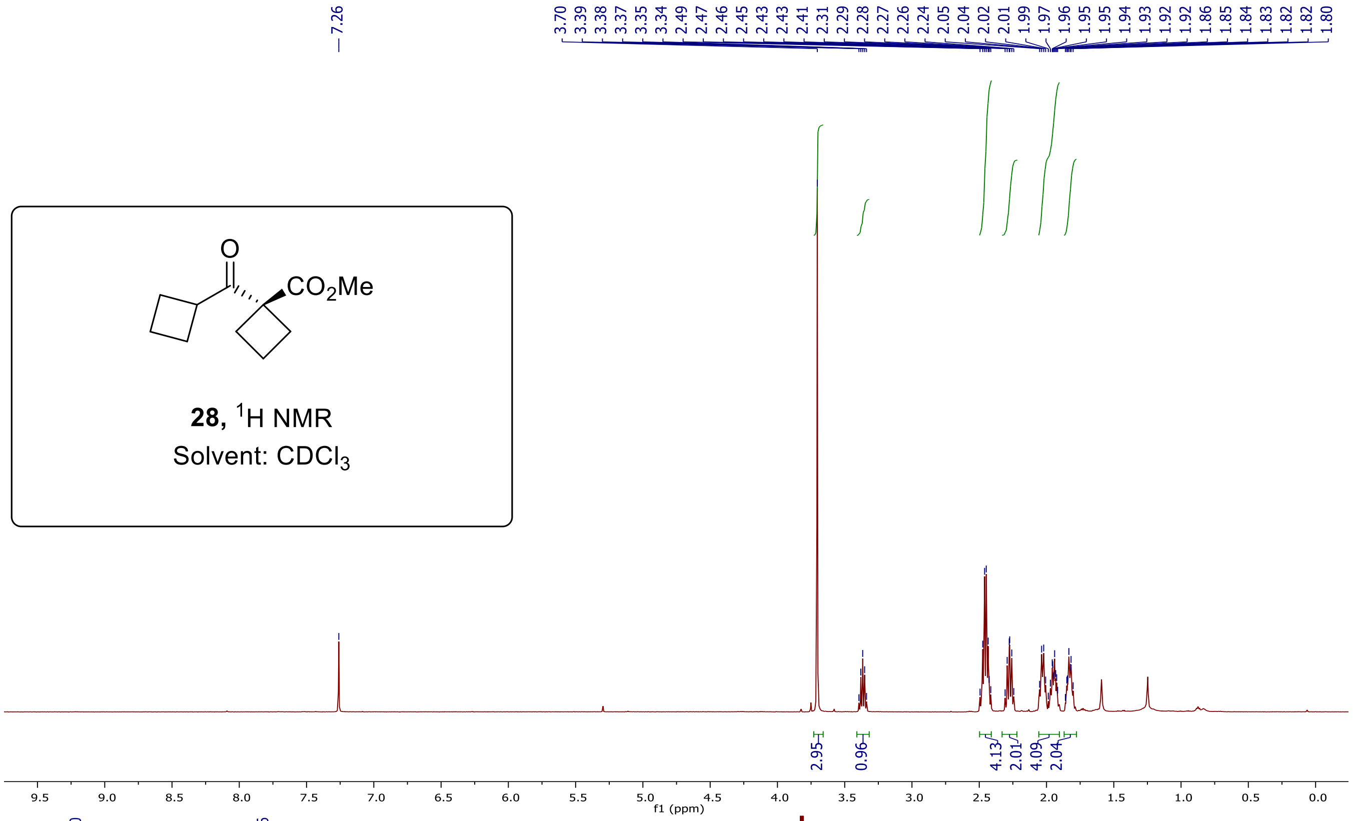

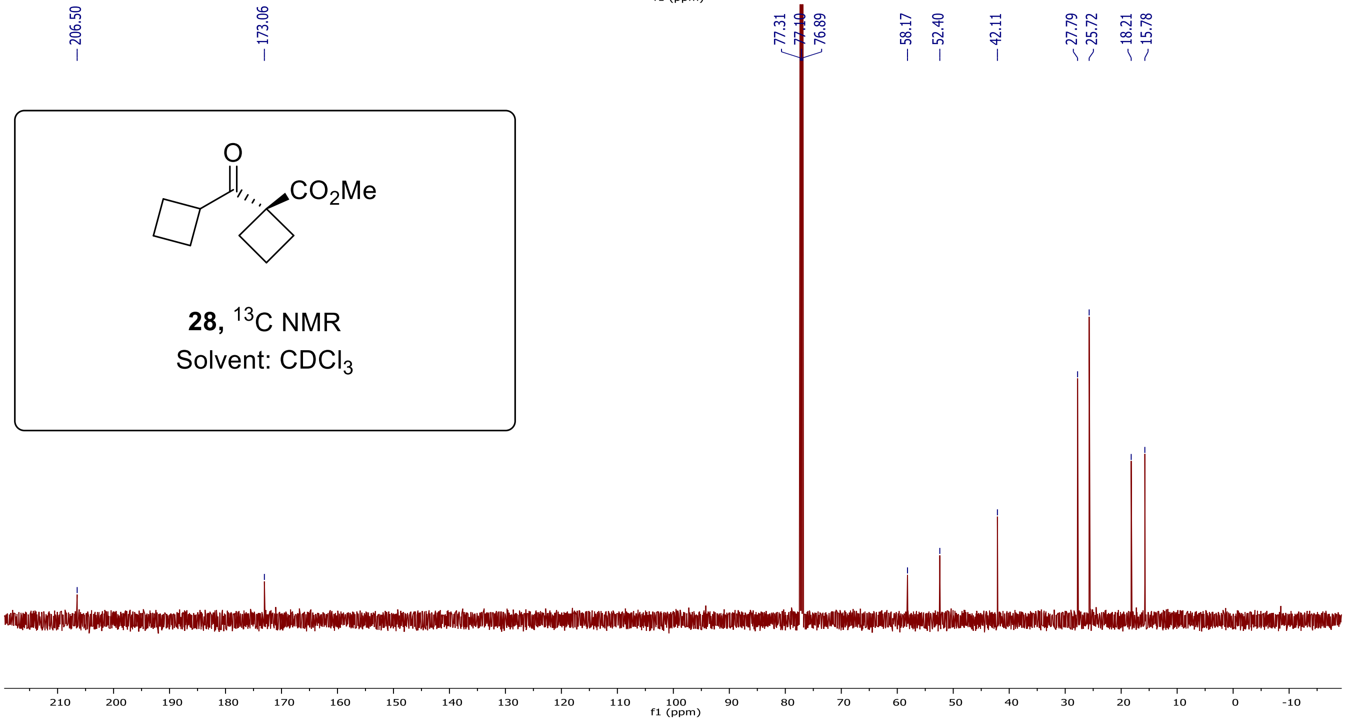

Supplementary Figure 147. NMR spectra of **28**

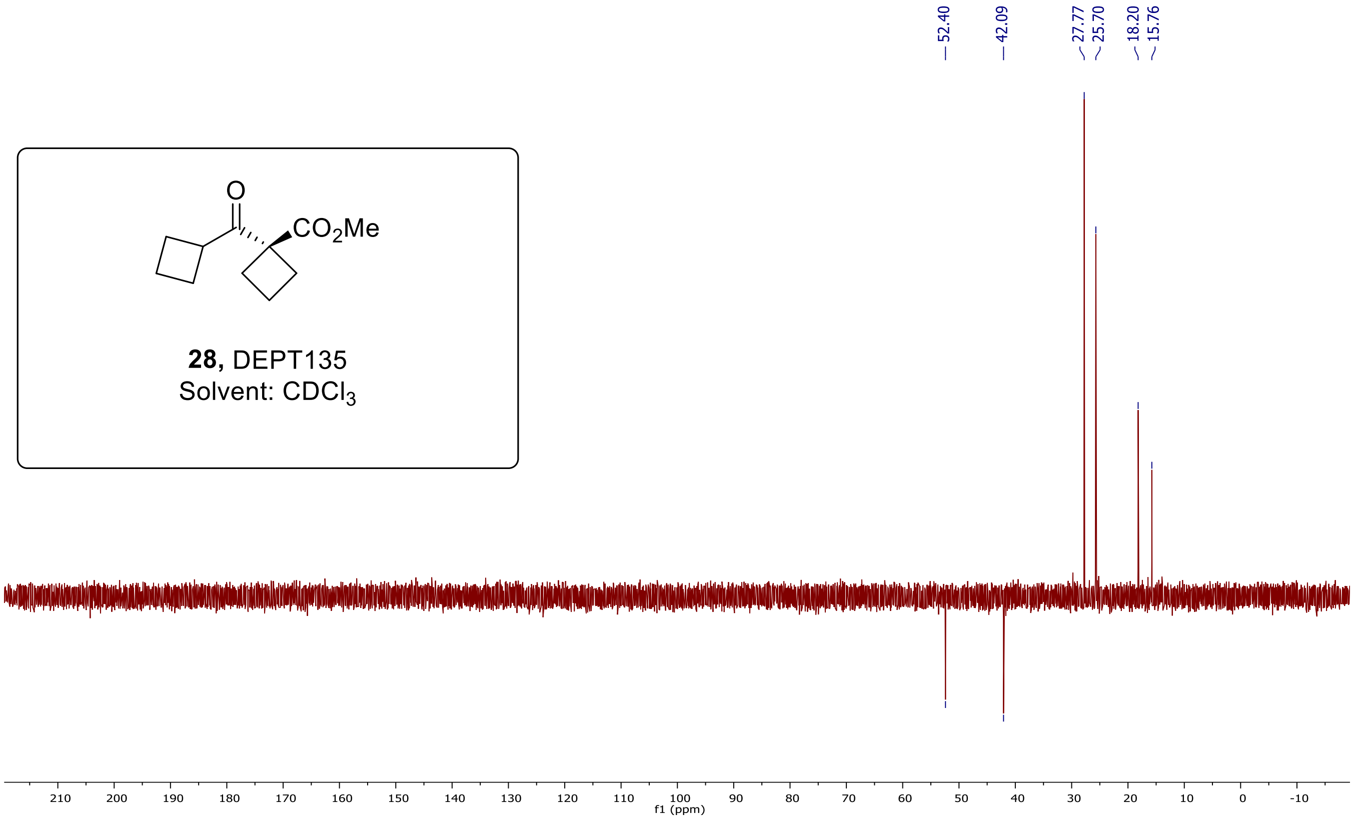

Supplementary Figure 148. DEPT135 spectrum of **28**

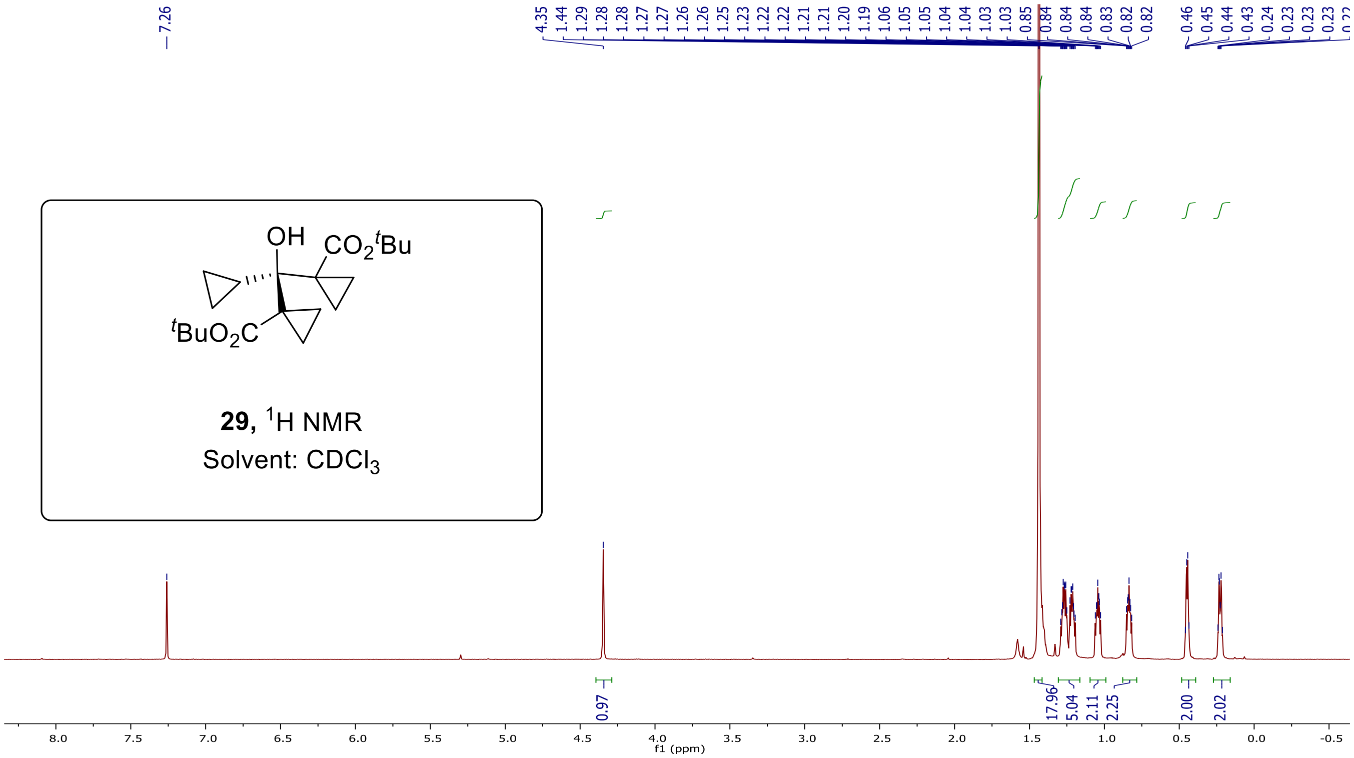

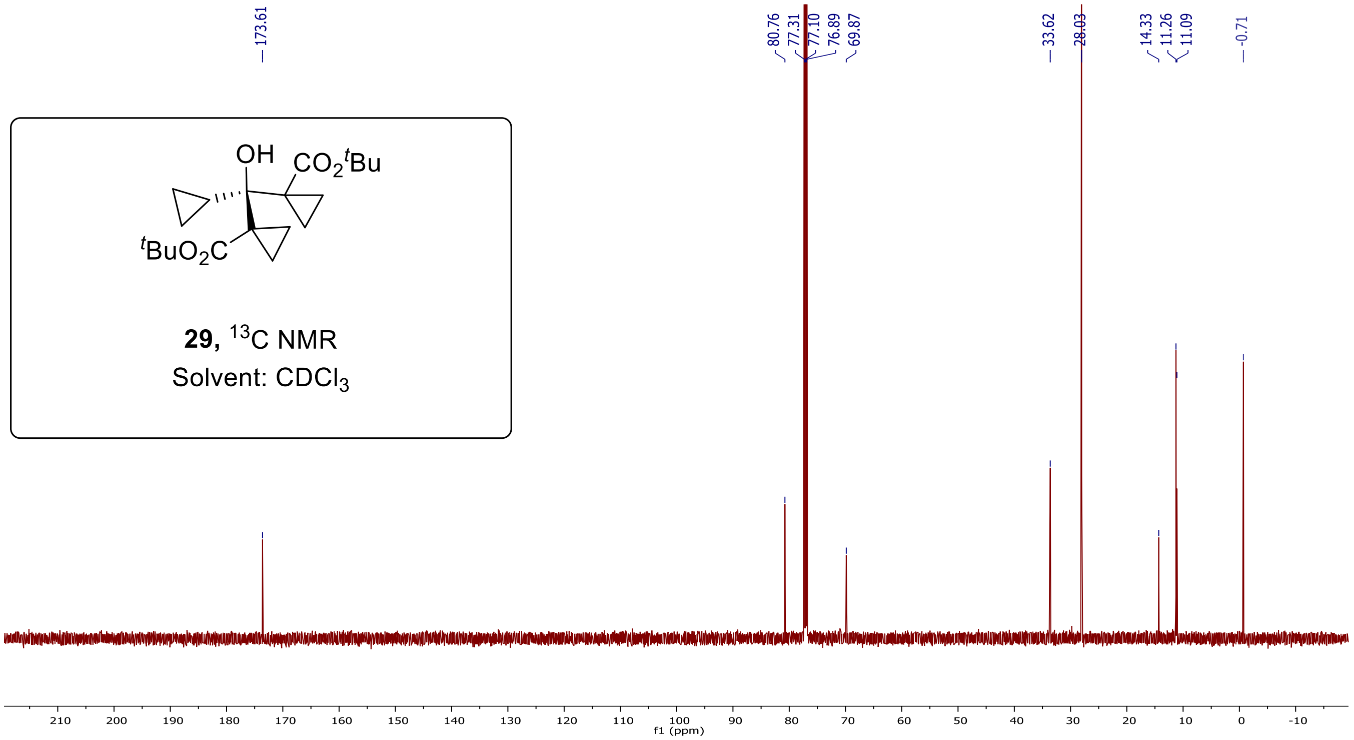

Supplementary Figure 149. NMR spectra of **29**

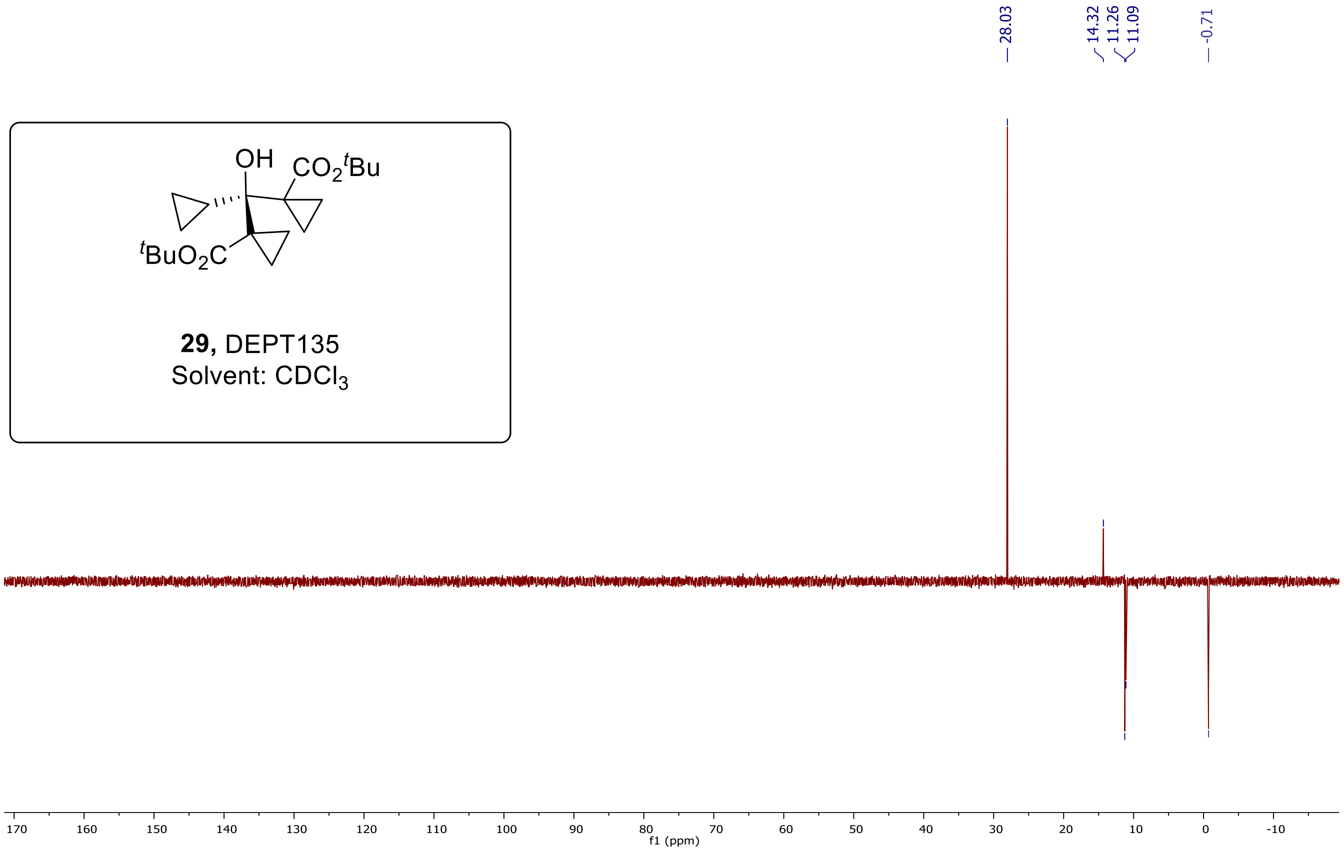

Supplementary Figure 150. DEPT135 spectrum of **29**
